# Deoxyfluorination of alcohols with 3,3-difluoro-1,2-diarylcyclopropenes

**DOI:** 10.1038/ncomms13320

**Published:** 2016-11-14

**Authors:** Lingchun Li, Chuanfa Ni, Fei Wang, Jinbo Hu

**Affiliations:** 1 Key Laboratory of Organofluorine Chemistry, Shanghai Institute of Organic Chemistry, Chinese Academy of Sciences, 345 Ling-Ling Road, Shanghai 200032, China

## Abstract

Aromatic cation activation is a useful strategy to promote deoxyfunctionalization; however, the deoxyfluorination of alcohols with cyclopropenium cation remains an unsolved problem due to the weak nucleophilicity of fluoride ion. Here we report the use of 3,3-difluoro-1,2-diarylcyclopropenes (CpFluors) as easily accessible and reactivity-tunable deoxyfluorination reagents. The electronic nature of CpFluors is critical for fluorination of monoalcohols via alkoxycyclopropenium cations, and CpFluors with electron-rich aryl substituents facilitate the transformation with high efficiency; however, selective monofluorination of 1,2- and 1,3-diols, which proceeds via cyclopropenone acetals, is less dependent on the electronic nature of CpFluors. Moreover, CpFluors are more sensitive to the electronic nature of alcohols than many other deoxyfluorination reagents, thus fluorination of longer diols can be achieved selectively at the relatively electron-rich position. This research not only unveils the first example of deoxyfluorination reagents that contain an all-carbon scaffold, but also sheds light on the divergent reactivity of cyclopropenium cation in deoxyfunctionalization of alcohols.

## Abstract (web summary)

Fluorination of organic molecules can dramatically change the properties and is commonly used for the synthesis of bioactive molecules. Here, the authors report the deoxyfluorination of alcohols and the selective monofluorination of diols using bench stable, tunable difluorocyclopropene reagents.

The introduction of fluorine atoms into organic molecules can induce marked changes in their chemical, physical and pharmacological properties, and fluorination has become a routine and powerful strategy in the design of new materials and drug candidates^1^,^2^,^3^. As a result, numerous efforts have been devoted to the development of fluorination methods^4^,^5^,^6^,^7^,^8^. Among them, direct deoxyfluorination of alcohols via nucleophilic fluorination of the *in situ*-generated activated intermediates, is a major approach to obtain alkyl fluorides, thanks to the abundance of natural and synthetic alcohols^8^,^9^,^10^,^11^,^12^ (Fig. 1a). Since the first report on the SF_4_-mediated deoxyfluorination reactions^13^, dozens of power-variable deoxyfluorination reagents have been developed^8^,^9^,^10^,^11^,^12^. Generally, these reagents can be divided into two categories: one is the sulfur fluorides and their derivatives (SF reagents)^14^,^15^,^16^,^17^,^18^ (Fig. 1b), and the other is the α-fluorinated alkylamines and their derivatives (NCF reagents)^19^,^20^,^21^,^22^ (Fig. 1c). The deoxyfluorination with these heteroatom-based reagents, however, suffers from disadvantages such as high cost, thermal instability, harsh reaction conditions or the requirement of external fluoride sources or bases. Moreover, these fluorination reagents rely on heteroatom-based motifs both as the fluoride carrier and as the C−O bond activator of alcohols. Carbon is the core of organic compounds and the use of an all-carbon moiety to activate a molecule is of great interest for both organic synthesis and mechanistic organic chemistry^23^,^24^. In this context, we have become interested in developing a new deoxyfluorination protocol using 3,3-difluorocyclopropenes as a class of novel fluorination reagents with an all-carbon scaffold (CCF reagents).

3,3-Difluorocyclopropenes are readily prepared by difluoromethylenation of the corresponding alkynes with many difluorocarbene sources^25^,^26^ (Fig. 1d); however, their defluorinative transformations have been only focused on the use of fluorine as a leaving group, thus fluoride is eliminated as a waste^27^,^28^,^29^,^30^,^31^,^32^. Although the deoxychlorination of alcohols with their analogues 3,3-dichlorocyclopropenes via cyclopropenium cation activation has been disclosed since 2009 (refs 33, 34), the deoxyfluorination with 3,3-difluorocyclopropenes is still challenging due to the weak nucleophilicity of fluoride ion.

Here we report our success on deoxyfluorination of alcohols using crystalline, thermally stable solid reagents 3,3-difluoro-1,2-diarylcyclopropenes (CpFluors, **1**) as reactivity-tunable fluorination reagents for the preparation of alkyl fluorides (Fig. 1d). The unique feature and potential application of this method are demonstrated in direct fluorination of monoalcohols through the cyclopropenium cation activation manner and acryloylative monofluorination of 1,2- and 1,3-diols through the cyclopropenone acetal activation manner. In the case of monoalcohols, tuning the electronic nature of the aryl substituents on CpFluors **1** is the key to suppress the competitive cyclopropenone acetal activation pathway, thus improving the efficiency of the desired deoxyfluorination. Furthermore, CpFluors are more sensitive to the electronic nature of alcohols than many other deoxyfluorination reagents, thus fluorination of longer diols can be achieved selectively at the relatively electron-rich position by using these new reagents.

## Results

### Reaction design

**Figure 1:**
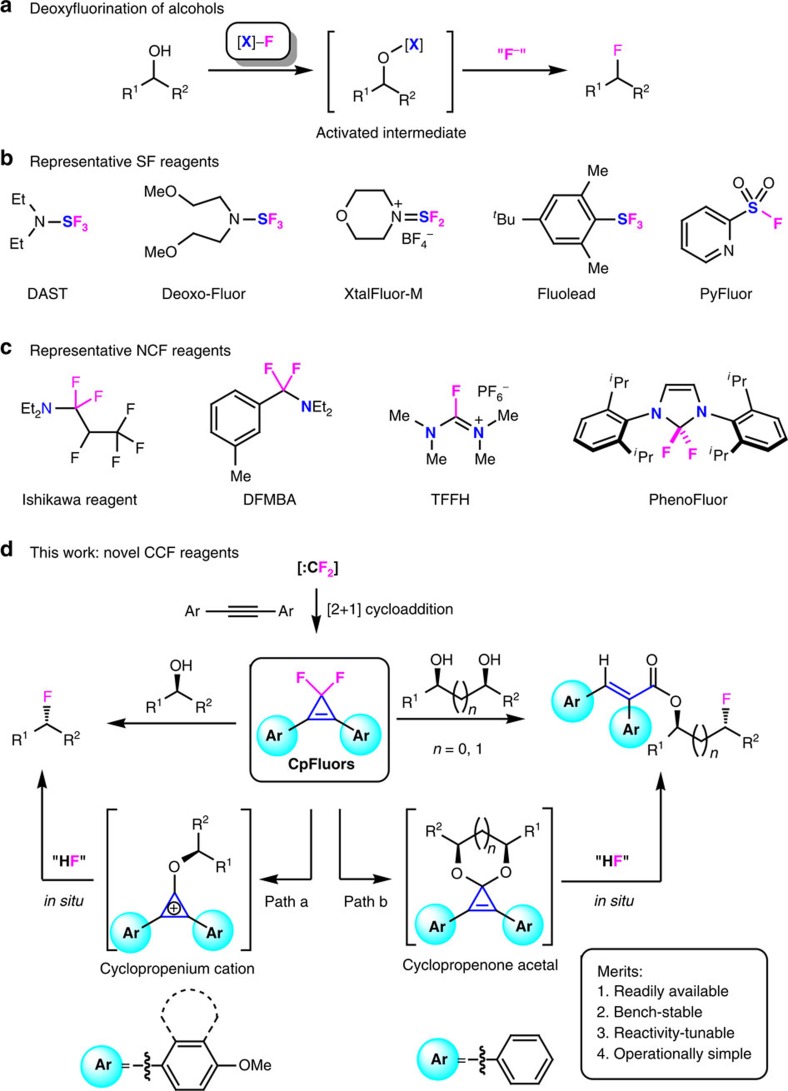
Methods for deoxyfluorination of alcohols. (**a**) General route for deoxyfluorination of alcohols, proceeding through the activation of alcohol followed by displacement with a fluoride ion. (**b**) Representative SF reagents, including aminosulfur trifluorides (such as diethylaminosulfur trifluoride (DAST) and Deoxo-Fluor)^14^,^15^, aminodifluorosulfinium salts (such as XtalFluor-M)^16^, arylsulfur trifluorides (such as Fluolead)^17^ and sulfonyl fluorides (such as PyFluor)^18^. (**c**) Representative NCF reagents, including perfluoroolefin-secondary amine adducts (such as Ishikawa reagent)^19^, difluorobenzylamines (such as *N*,*N*-diethyl-*a*,*a*-difluoro-(*m*-methylbenzyl)amine (DFMBA))^20^, fluoroiminium salts (such as tetramethylfluoroformamidinium hexafluorophosphate (TFFH))^21^ and difluoromethanediamines (such as PhenoFluor)^22^. (**d**) Our method using difluorocarbene-derived CCF reagents, 3,3-difluoro-1,2-diarylcyclopropenes (CpFluors). CpFluors are reactivity-tunable and are capable of fluorinating both monoalcohols (path a) and diols (path b) through different activation mode.

Previously, we were engaged in the development of novel difluorocarbene reagents for the difluoromethylation of heteroatom nucleophiles and difluoromethylenation of alkenes and alkynes^26^. During the course of our studies, we noticed that 3,3-difluorocyclopropenes can be hydrolyzed to cyclopropenones upon prolonged storage in a wet atmosphere and that their stability towards water is closely related to their molecular structures^35^,^36^,^37^. On the basis of these findings, we have initiated a program aiming to use the safe and readily available difluorocarbene reagents for deoxyfluorination by virtue of 3,3-difluorocyclopropenes. This research program was inspired by Lambert's deoxychlorination with 3,3-dichlorocyclopropenes, which utilizes aromatic cyclopropenium cations as the activator for the transformation of monoalcohols and carboxylic acids to alkyl chlorides and acyl chlorides, respectively^33^,^34^,^38^. We envisioned that under similar conditions, 3,3-difluorocyclopropenes may be used for deoxyfluorination of alcohols via nucleophilic attack of the alkoxycyclopropenium cation intermediate by the *in situ*-generated fluoride ion (Fig. 1d, path a).

### Initial attempt

At the onset, we chose 3,3-difluoro-1,2-diphenylcyclopropene (CpFluor **1a**) as the reagent to survey the suitable conditions for deoxyfluorination. After numerous trials and failures, we found that, unlike dichlorocyclopropenes^33^,^34^,^38^, CpFluor **1a** was only effective for the dehydroxyfluorination of carboxylic acids and benzyl alcohols^39^, and its fluorination of non-activated alcohols such as 3-phenylpropan-1-ol (**2a**) was unexpectedly difficult. Table 1 shows the representative conditions that were screened with **2a** as the model substrate. When the reaction was conducted in dichloromethane at room temperature, CpFluor **1a** was found to be inert towards alcohol **2a**, and at an elevated temperature (80 °C), **1a** readily reacts with glass (mainly SiO_2_) rather than **2a** due to the formation of HF (Table 1, entry 1). Therefore, a non-glass vessel should be used to avoid the undesired deoxyfluorination of glass. To our delight, when a polytetrafluoroethylene (PTFE) tube was used as the reaction vessel, the desired alkyl fluoride **3a** was produced in 42% yield (Table 1, entry 2). In terms of reaction solvents, no product **3a** was formed when THF was used, and acetonitrile provided only a very low yield (Table 1, entries 3 and 4). Other solvents such as toluene, chlorobenzene and 1,2-dichloroethane (DCE) were found to be much more efficient than THF and acetonitrile (Table 1, entries 5–7). These results indicate that Lewis basic solvents, which can serve as proton acceptors, are harmful to this reaction. To further improve the yield, we turned our attention to optimize the reactant ratio using DCE as the optimal solvent. However, to our great surprise, the highest yield of alkyl fluoride **3a** was only moderate even a large excess of CpFluor **1a** was used (entries 8–10). According to the conversion of **1a** and the yield of **3a**, we speculated that alcohol **2a** also took part in a side reaction. Moreover, we found that raising the temperature to 100 °C had little influence on the reaction, whereas lowering the temperature to 60 °C resulted in no desired reaction (entries 11 and 12).

### Modification of the reagent

**Figure 2:**
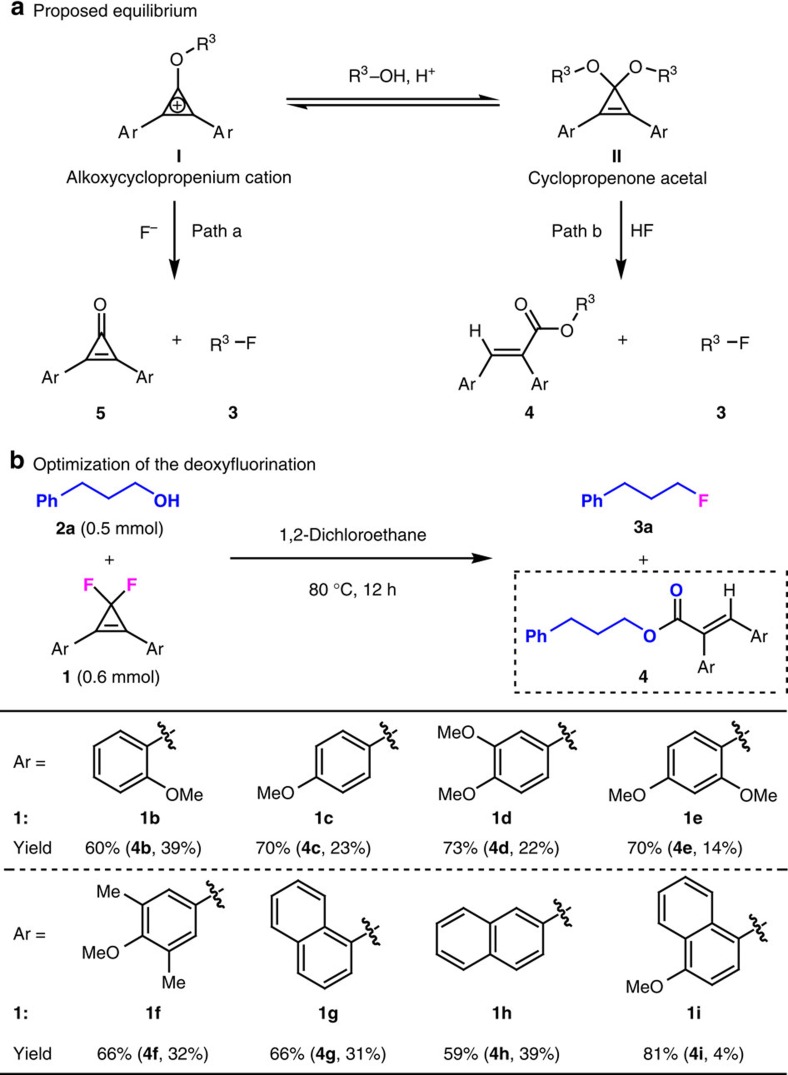
Substitution effect on the outcome of the deoxyfluorination. (**a**) A proposed competitive reaction pathway that arises from an equilibrium between alkoxycyclopropenium cation **I** and cyclopropenone acetal **II**. (**b**) Optimization on the deoxyfluorination via screening CpFluors with different aryl substitutents. Yields of **3a** were determined by ^19^F NMR spectroscopy using PhCF_3_ as an internal standard. Yields of **4** refer to the isolated yields.

To further improve the yield of alkyl fluoride **3a**, it is necessary to figure out and suppress the undesired competitive reaction pathway(s). An analysis of the crude reaction mixture with gas chromatography—mass spectrometry showed that 2,3-diphenylacrylate **4a** (Table 1) was formed as the major side product, along with a substantial amount of 2,3-diphenylcyclopropenone (**5a**). The identity of **4a** was confirmed by its separation (40% yield) and characterization. According to the above results, we proposed that the side product **4a** may arise from hydrogen fluoride-promoted ring-opening of a cyclopropenone acetal intermediate **II** that is in equilibrium with an alkoxycyclopropenium cation intermediate **I** (Fig. 2a)^40^,^41^,^42^. The occurrence of the cyclopropenone acetal pathway (path b) is attributed to the weak nucleophilicity of fluoride ion. The slow consumption of aromatic cation **I** by fluoride ion (path a) results in its accumulation and thus promotes the formation of acetal **II**. On the basis of this rationalization, we envisioned that the desired fluorination of alcohols via cyclopropenium cation might be significantly improved by tuning the electronic nature of CpFluors **1**. In other words, if we could further stabilize the cyclopropenium cation **I** (ref. 43), the formation of the cyclopropenone acetal intermediate **II** might be diminished, thus improving the desired fluorination reaction.

With aforementioned conceptions in mind, we made a survey on the deoxyfluorination of alcohol **2a** by using a series of structurally symmetrical 3,3-difluorocyclopropenes bearing various electron-rich aryl substituents under the same reaction conditions as those shown in Table 1, entry 7 (Fig. 2b). To our delight, when a methoxy group was introduced at the *ortho*-position of the phenyl group (**1b**), the yield of alkyl fluoride **3a** was enhanced to 60%, and changing the substitution pattern from *ortho*- to *para*-position could further improve the yield of **3a** to 70% (**1c**). However, compared with **1b** and **1c**, installing more than one electron-donating substituent on the phenyl ring has little influence on the chemical outcomes (**1d–1f**). The use of naphthyl groups instead of phenyl groups is also beneficial for the deoxyfluorination, and 1-naphthyl substituted **1g** gives a better result than 2-naphthyl substituted **1h** (**1g** and **1h**). Finally, we established that 4-methoxy-1-naphthyl-substituted difluorocyclopropene **1i** was the optimal reagent, giving the desired product **3a** in up to 81% yield with the generation of side product **4i** in only 4% yield (**1i**). Reducing the amount of **1i** (1.1 equivalents) and shortening the reaction time (8 h) gives an identical result (Supplementary Table 1). Note that the increase of the yield of alkyl fluoride **3** was always accompanied by the decrease of the yield of ester **4**, suggesting the existence of two tunable reaction pathways.

### Fluorination of monoalcohols

To evaluate the application scope of this deoxyfluorination protocol, we explored the transformation of structurally diverse monoalcohols by using CpFluor **1i** as a useful reagent under the optimized conditions (Table 2; Supplementary Table 1). The reaction of a broad range of primary (**3b**–**3g**) and secondary (**3j**–**3t**) monoalcohols proceeded smoothly to afford the corresponding alkyl fluorides in moderate to excellent yields. Chiral secondary alcohols were deoxyfluorinated to furnish products with inversion of configuration (**3n**–**3q**). Tertiary alcohols often give rise to poor results due to the predominant elimination reaction; however, as an exception, 1-adamantanol could be effectively deoxyfluorinated (**3i**), which is in accordance with the Bredt's rule^44^. Although fluoride ion is involved in this fluorination reaction, alkyl bromide does not undergo competing halex reaction (**3c**). Aldehyde and ketone functionalities, which readily undergo *gem*-difluorination with DAST and its derivatives^14^,^15^, can keep intact under our reaction conditions (**3n**, **3r** and **3t**). Other functionalities such as amide (**3n**), carbamate (**3o**) and sulfonamide (**3p**) are also tolerated under the present conditions. Benzylic alcohols are the most reactive and can be fluorinated with very high efficiency, and the carbonium ion-induced self-polymerization or elimination reaction was not observed (**3e** and **3m**)^17^. A homobenzylic alcohol also underwent smooth fluorination, albeit giving the alkyl fluoride in slightly lower yield due to the competitive elimination (**3f**). In addition to the good chemoselectivity and high stereospecificity, its ability for late-stage fluorination of complex molecules is another feature of this method. For examples, several steroids were selectively fluorinated at the hydroxyl group with high diastereoselectivity (**3r**–**3t**). Note that products **3r** and **3s** were obtained with retention of the configuration due to a homoallylic participation^45^. The *para*-methoxyphenyl-substituted CpFluor **1c** is also capable of fluorinating many alcohols (**3b**, **3k**, **3p** and **3r**), albeit usually in somewhat lower yields. However, although this method is compatible with several amide moieties, the substrates containing electron-rich amine groups, such as pyridine, cannot take part in reaction due to their inhibition effect on the initial activation of CpFluors, revealing a limitation of the additive-free deoxyfluorination method. We found that this limitation can be alleviated by the addition of an acid activator. For example, the reaction of alcohol **2h** with CpFluor **1c** in the presence of pyridine-hydrogen fluoride (Py·9HF) afforded the desired alkyl fluoride **3h** in moderate yield (54%).

### Fluorination of 1,2- and 1,3-diols

Selective fluorination of multiple alcohols, especially the symmetrical diols, using common deoxyfluorination reagents such as DAST, is a challenging synthetic task^46^,^47^. To date, available examples for selective deoxyfluorination of 1,2- and 1,3-diols mainly depend on the employment of sterically hindered reagents or reagents that can *in situ* generate stable hydroxyl protection groups^17^,^18^,^22^,^48^,^49^. On the basis of our finding that the competitive reaction of alcohol and fluoride ion towards alkoxycyclopropenium cation intermediate is tunable, we envisioned that the formation of a cyclic cyclopropenone acetal, by using 1,2- or 1,3-diols as substrates may lead to a productive monofluorination. Thus, the reactions of 2,2-dimethylpropane-1,3-diol (**6l**) with CpFluors **1a** and **1i** were examined under the optimized conditions for monoalcohols. To our delight, both of the reactions proceeded smoothly to afford the corresponding acryloylative monofluorination products selectively (see Supplementary Methods for deoxyfluorination of 1,2- and 1,3-diols). However, we found that CpFluor **1a** was more efficient than CpFluor **1i**, probably due to a relatively faster formation of the cyclic acetal intermediate from **1a**. By using **1a** as the reagent, we further investigated the scope of diols. As shown in Table 3, a series of symmetrical and unsymmetrical 1,2- and 1,3-diols were monofluorinated in high yields with good functional group tolerance. Functionalities such as alkyl halides (**7g** and **7m**) and alkenes (**7d** and **7h**) were intact during the reaction; however, a nitro substituent could retard the reaction significantly (**7n**). For unsymmetrical 1,2-diols, fluorination occurred preferentially at the less sterically hindered carbon, and the oxymethyl- and chloromethyl-substituted ones (**7d**–**7g**) were fluorinated more selectively than the simple alkyl-substituted ones (**7b** and **7c**). The transformation of unsymmetrical 1,3-diols also proceeded regioselectively (**7k**–**7p**). When 3-alkyl substituted 1,3-diol underwent the reaction, 3-position fluorination was predominated (**7o**); however, when a sugar derivative with 1,3-diol motif was subjected to this reaction, the primary alkyl fluoride was obtained in excellent yield (**7p**). The observed unusual preference of the fluoride ion for the secondary position in the case of **7o** can be attributed to the inductive and conformational effects present in the cyclic dioxanylium cation intermediate^50^. Moreover, fluorination at a chiral carbon proceeded with the inversion of the stereochemistry, as is indicated by single-crystal diffraction of product **7j** derived from *syn*-cyclohexane-1,2-diol (**6j**).

### Fluorination of longer diols

**Figure 3:**
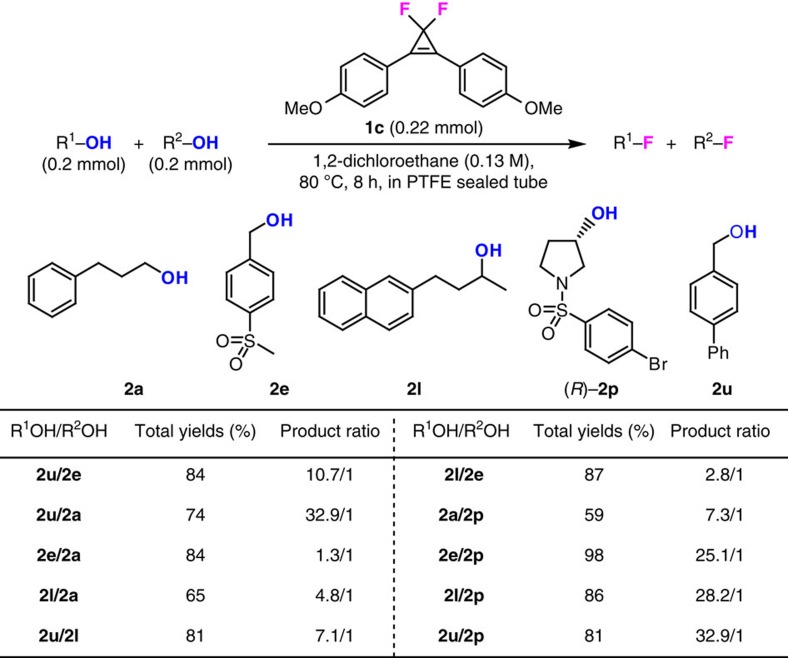
Competitive deoxyfluorination of monoalcohols. Competitive deoxyfluorination of primary and secondary alcohols with CpFluor **1c**. The yields and product ratios were determined by ^19^F NMR spectroscopy using PhCF_3_ as an internal standard.

To expand the synthetic application of our developed reagents, we also investigated the selective deoxyfluorination of longer diols, where the two hydroxyl groups are separated by several carbon centers. In this case, the formation of a cyclic acetal intermediate is disfavoured; therefore, the challenge associated with this synthetic goal is to distinguish two separated hydroxyl groups, especially when they possess similar steric hindrance. To evaluate the selective deoxyfluorination capability of our method, we first conducted the competitive fluorination of different monoalcohols with the much more easily accessible CpFluor **1c**, as the reagent under standard reaction conditions (Fig. 3). Among the primary alcohols tested, benzylic alcohols **2e** and **2u** are more active than the normal primary alcohol **2a**, and the reactivity of benzylic alcohols are tunable, with the relatively electron-rich **2u** being more reactive than the electron-deficient **2e**. To our great surprise, when comparing primary alcohol **2a** with secondary alcohol **2l**, we found that **2a** was less reactive, which is distinct from the expected reactivity order of typical S_N_2 reactions. Moreover, alcohol **2l** was found to be even more reactive than electron-deficient benzylic alcohol **2e**. These results indicate that it is the electronic nature of the alcohol rather than the steric hindrance of the alcohol that controls the selectivity of our deoxyfluorination reactions, at least for primary and secondary alcohols. This inference is further supported by the competitive reactions of electron-deficient 3-pyrrolidinol **2p** and other alcohols, with **2p** being the least reactive. Taking together, the reactivity of all the tested alcohols decreases in the following order: **2u**>**2l**>**2e**>**2a**>**2p**. This interesting reactivity feature of alcohols may arise from the unique activation mode of our deoxyfluorination method, where not only the electronic nature of the CpFluor reagents, but also the electronic nature of the alcohol substrates can influence the formation of the reactive alkoxycyclopropenium cation intermediate. The electron-rich alcohol can promote the formation and stabilization of the alkoxycyclopropenium cation, thus favoring deoxyfluorination.

**Figure 4:**
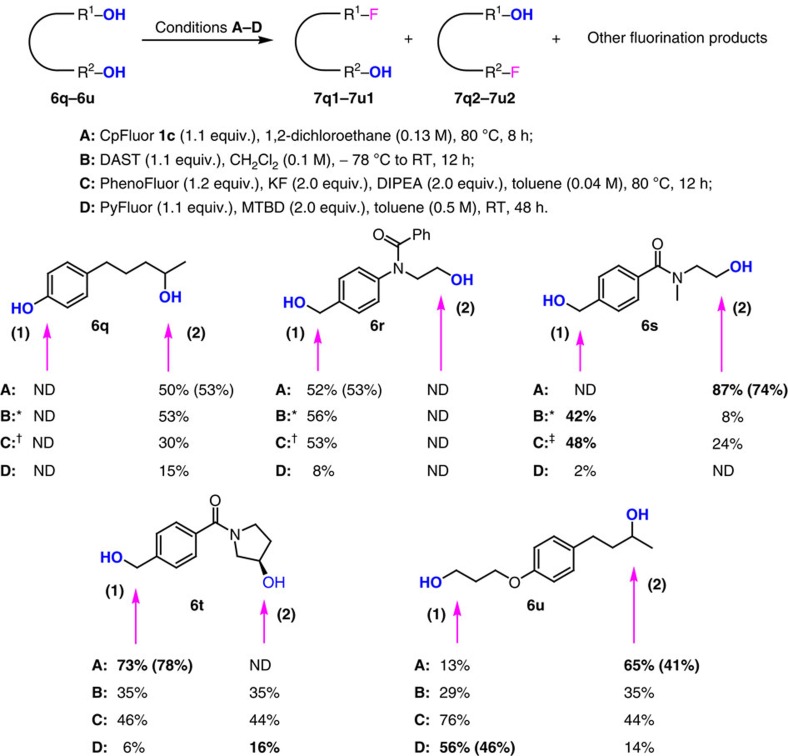
Selective deoxyfluorination of longer diols. Selective deoxyfluorination of longer diols, where the two hydroxyl groups are separated by several carbon centers. The total fluorination yields at the given position were determined by ^19^F NMR spectroscopy using PhCF_3_ as an internal standard. The yields in parentheses refer to isolated yields of the monofluorination products with the retention of the other hydroxyl group. *1.5 equiv. of DAST was used. ^†^1.5 equiv. of PhenoFluor was used. ^‡^The reaction was performed using 1.0 equiv. of PhenoFluor at RT for 48 h. DIPEA, *N*,*N*-diisopropylethylamine; MTBD, 7-methyl-1,5,7-triazabicyclo[4.4.0]dec-5-ene; ND, not detected.

Inspired by the above finding, we investigated the deoxyfluorination of several diols (or functionalized alcohol) with CpFluor **1c** and made a comparison with the results of other representative reagents DAST, PhenoFluor, as well as PyFluor (Fig. 4). By using CpFluor **1c** as the reagent, alcohols **6q**–**6u** were selectively fluorinated to give the monofluorinated compounds with retention of the other hydroxy group in moderate to good yields. Secondary alcohol **6q** with a phenolic hydroxyl group smoothly underwent deoxyfluorination at the alcoholic position to give the monofluorinated phenol **7q2**. Diols **6r** and **6s** with similar steric hindrances at both ends were selectively fluorinated at the relatively electron-rich position, affording the monofluorinated compounds **7r1** and **7s2** as the only products. In the case of diol **6t** bearing an electron-deficient benzyl alcohol moiety and an electron-deficient 3-pyrrolidinol moiety, deoxyfluorination occurred selectively at the benzylic position. A comparison between the results of **6s** and **6t** showed that in addition to the electronic effect, the stereoelectronic effect also can influence the selectivity of deoxyfluorination with CpFluor reagents. Moreover, in the case of diol **6u** containing both primary and acyclic secondary alcohol moieties, the relatively electron-rich secondary position preferred to be fluorinated, which is in accordance with the selectivity of intramolecular competitive reactions (**2a**/**2l**). Although the fluorination of **6q** and **6r** with DAST, PhenoFluor and PyFluor resulted in the same selectivity (not considering the yield) as CpFluor **1c** did, the fluorination of **6s**–**6u** with the three reagents afforded either opposite selectivity or poor selectivity. These preliminary results demonstrate that the chemical outcome of deoxyfluorination with various reagents is complicatedly influenced by both the steric hindrance and electronic nature of the alcohol substrates. Among the four kinds of reagents tested, CpFluor reagents are the most sensitive towards the electronic nature of the alcohols and are potentially useful for selective deoxyfluorination not otherwise achievable.

### Experimental investigation of reaction mechanism

**Figure 5:**
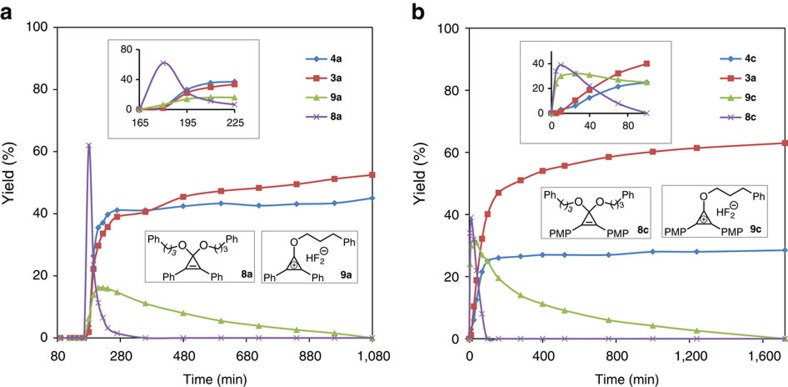
Mechanistic study on the deoxyfluorination of alcohols with CpFluors. CpFluors with electron-netural (**1a**) and electron-rich (**1c**) aryl substitutents were chosen to monitor the influence of electronic nature on the chemical outcome. Both reactions were perforformed in PTFE NMR tubes with DCE-*d*_4_ as the solvent. (**a**,**b**) Yield profiles of products and intermediates generated in the reactions of 3-phenylpropan-1-ol (**2a**, 1.0 equiv.) (**a**,**b**) with CpFluors **1a** (1.0 equiv.) (**a**) and **1c** (1.0 equiv.) (**b**) during the whole monitoring process. Insets on the top (**a**,**b**): the enlarged yield profiles at the early stage of the reactions. PMP, *p*-methoxyphenyl.

To better understand the reaction mechanism of CpFluors with different substitutions, we monitored the reaction of monoalcohol **2a** with CpFluor **1a** or **1c** in DCE-*d*_4_ by ^19^F and ^1^H nuclear magnetic resonance (NMR) spectroscopy (Fig. 5). CpFluor **1c** was chosen instead of **1i**, because the progress of the reaction with **1c** could be followed easily due to its good solubility.

For the reaction between alcohol **2a** and CpFluor **1a** (Fig. 5a; Supplementary Table 2), there was a significant induction period. During this period (*t*=0–165 min), the consumption of a small amount of **1a** and formation of HF were detected by ^19^F NMR analysis. The formation of trace HF is also supported by the ^1^H NMR chemical shift change of the alcoholic hydroxyl group (from 1.64 to 2.38 p.p.m.). After the induction period, a fast reaction between **1a** and **2a** gave intermediate **8a** and HF as the major products; however, only a small amount of alkyl fluoride **3a** was formed (*t*=165–180 min). According to the estimated molar ratio of consumed **1a** and formed **8a**, as well as the diagnostic ^1^H NMR spectrum of **8a** (see Supplementary Methods for investigation of reaction mechanism), the structure of observed **8a** is consistent with a cyclopropenone acetal. Subsequently (*t*=180–195 min), a fast consumption of **8a** led to a fast formation of alkyl fluoride **3a** and ester **4a**; meanwhile, a new intermediate that is assigned to alkoxycyclopropenium **9a** (ref. 33) was detected in substantial amount. As the reaction proceeded, a complete consumption of **8a** (*t*=195–300 min) and **9a** (*t*=210–1,080 min) furnished alkyl fluoride **3a** in moderate yield. It should be noted that during the first 300 min, alkyl fluoride **3a** and ester **4a** were produced at the similar rate. From these results, we confirmed that the fluorination of **2a** with the less electron-rich CpFluor **1a** proceeds predominantly through the ring-opening of a cyclopropenone acetal intermediate, and the cyclopropenium cation activation pathway (mainly occurred at the late stage of the reaction) contributed less to this transformation.

When monitoring the reaction between alcohol **2a** and CpFluor **1c** (Fig. 5b; Supplementary Table 3), no significant induction period was observed. At the initial stage (*t*=0–10 min), a very fast reaction between **2a** and **1c** afforded HF and a nearly 1:1 mixture of intermediates **8c** and **9c** as the major products. The structures of **8c** and **9c** were assigned on the basis of their diagnostic ^1^H NMR spectra (see Supplementary Methods for investigation of reaction mechanism). In the subsequent course, cyclopropenone acetal **8c** and alkoxycyclopropenium **9c** were converted to alkyl fluorides at different rates. Judging from the rate for the formation of alkyl fluoride **3a** and ester **4c**, acetal **8c** was consumed much faster than cyclopropenium **9c** (*t*=10–100 min versus *t*=10–1,720 min). These results suggest that changing the aryl substituents' electronic nature from electron neutral to electron rich not only facilitates the activation of CpFluors, but also significantly inhibits the formation of cyclopropenone acetal from alkoxycyclopropenium cation, thus improving the fluorination efficiency.

**Figure 6:**
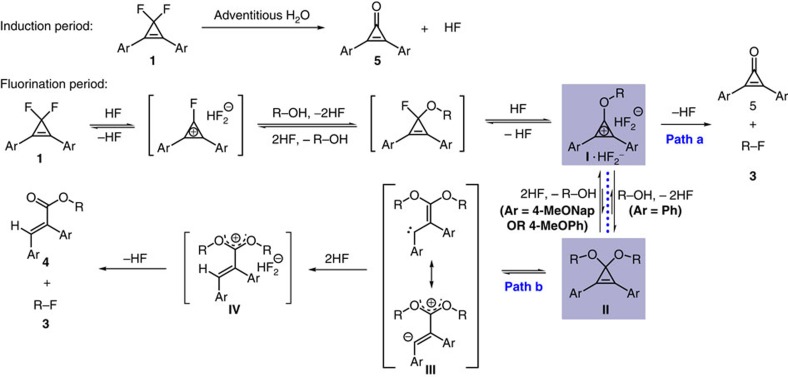
Proposed mechanism for deoxyfluorination of monoalcohols with electronically different CpFluors. CpFluor **1** undergoes a reaction with monoalcohols to afford intermediate **I**. The stability of **I** that is determined by the electronic nature of the aryl substituents can significantly influence its reaction pathway (path a or path b) to give the corresponding products.

According to above results and discussion, the proposed mechanism for the fluorination of monoalcohols with CpFluors is shown in Fig. 6. Under the activation of the trace amount of hydrogen fluoride that is probably generated from the hydrolysis of CpFluor **1** by adventitious water, CpFluor **1** undergoes a fast reaction with monoalcohols to afford intermediate **I**. Because the reaction of **I** with fluoride is slow, the stability of **I** that is determined by the electronic nature of the aryl substituents can significantly influence the fate of **I**. In the case of CpFluor **1** with electron-rich aryl substituents, such as 4-methoxynaphthalen-1-yl group, the stabilization of intermediate **I** benefits the fluorination of monoalcohols via nucleophilic displacement of the cyclopropenium oxide by fluoride (path a). When CpFluor **1** with less electron-rich substituents, such as phenyl group undergoes the reaction (path b), the formed less stable intermediate **I** further reacts with monoalcohols to give cyclopropenone acetal **II** as the more stable intermediate. The so-formed intermediate **II** is in equilibrium with intermediate **I** under the action of hydrogen fluoride and readily undergoes thermal-induced ring-opening reaction to give vinylcarben intermediate **III** (refs 40, 41, 42). Protonation of the vinylcarbene **III** by hydrogen fluoride followed by a fluoride attack delivers a mixture of alkyl fluoride **3** and acrylate **4**. The monofluorination of diols can be rationalized with the same depiction as shown in Fig. 6. In this case, the formation of the cyclic acetal of a diol is always favored regardless of the electronic nature of the CpFluors, and thus the fluorination proceeds through the second pathway (Fig. 6, path b).

## Discussion

We have developed a fluorination protocol by adapting readily available and sufficiently stable CpFluors as a novel class of deoxyfluorination reagents that contain an all-carbon scaffold. We found that the electronic nature of the aryl substituents on the *gem*-difluorocyclopropene scaffold is critical for the transformation of monoalcohols. CpFluors with electron-rich aryl substituents furnish the deoxyfluorination with high efficiency through a stable alkoxycyclopropenium intermediate, and the spectroscopic investigations have disclosed the mechanistic pathways of this unprecedented deoxyfluorination reaction. On the other hand, the selective fluorination of 1,2- and 1,3-diols proceeds through a stable cyclopropenone acetal intermediate and is less dependent on the electronic nature of CpFluors. Furthermore, the monofluorination via alkoxycyclopropenium cation is sensitive to the electronic nature of the alcohols, thus deoxyfluorination of longer diols can be achieved selectively at the relatively electron-rich position rather than the less sterically hindered position. This research not only provides a practical method for the fluorination of various alcohols, using reagents that can be readily prepared from easily accessible alkynes and difluorocarbene reagents, but also sheds light on the divergent reactivity of cyclopropenium cations in the transformation of alcohols. We believe that in selective deoxyfluorination of multiple alcohols, CpFluors are complementary to other reagents, such as DAST, PhenoFluor and PyFluor, and should be as important as these reagents. Further research on the synthetic application of CpFluors is underway.

## Methods

### General

The general procedures for deoxyfluorination of monoalcohols **2** with CpFluor **1i** are as follows. In a typical experiment, into a dried polytetrafluoroethylene tube equipped with a magnetic stir bar were sequentially added octadecan-1-ol (**2b**, 54.1 mg, 0.2 mmol), CpFluor **1i** (85.4 mg, 0.22 mmol) and DCE (1.5 ml). The tube was sealed and immersed in an oil bath at 80 °C. After being stirred for 8 h, the reaction mixture was cooled to room temperature and precipitated with ether (6 ml). The precipitate (mainly 2,3-bis(4-methoxynaphthalen-1-yl)cycloprop-2-enone) was filtered and washed with ether. The filtrate was concentrated under reduced pressure and the residue was purified by flash chromatography on silica gel to give alkyl fluoride **3b** (51.3 mg, 93%). The deoxyfluorination of 1,2- and 1,3-diols **6a-6p** with CpFluor **1a**, as well as longer diols **6q**–**6u** with CpFluor **1c** were carried out similarly and the procedures are presented in Supplementary Methods.

CpFluors **1a**-**1e** were prepared from diarylalkynes and (chlorodifluoromethyl)trimethylsilane according to procedures presented in Supplementary Methods.

### Data availability

The authors declare that the data supporting the findings of this study are available within the article and its Supplementary Information files. For the experimental procedures, and spectroscopic and physical data of compounds, see Supplementary Methods. For NMR analysis of the compounds in this article, see Supplementary Figs 1–222 and Fig 227. The CCDC 1443649, CCDC 1443627, CCDC 1443644 and CCDC 1443654 contain the crystallographic data for compounds **3m**, **3r**, **3t** and **7j**, respectively (Supplementary Figs 223–226; Supplementary Tables 4–19). These data can be obtained free of charge from the Cambridge Crystallographic Data Center (www.ccdc.cam.ac.uk).

## Additional information

**How to cite this article:** Li, L. *et al.* Deoxyfluorination of alcohols with 3,3-difluoro-1,2-diarylcyclopropenes. *Nat. Commun.* **7,** 13320 doi: 10.1038/ncomms13320 (2016).

**Publisher's note:** Springer Nature remains neutral with regard to jurisdictional claims in published maps and institutional affiliations.

## Supplementary Material

Supplementary Information Supplementary Figures 1-227, Supplementary Tables 1-19, Supplementary Methods and Supplementary References.

## Figures and Tables

**Table 1:**
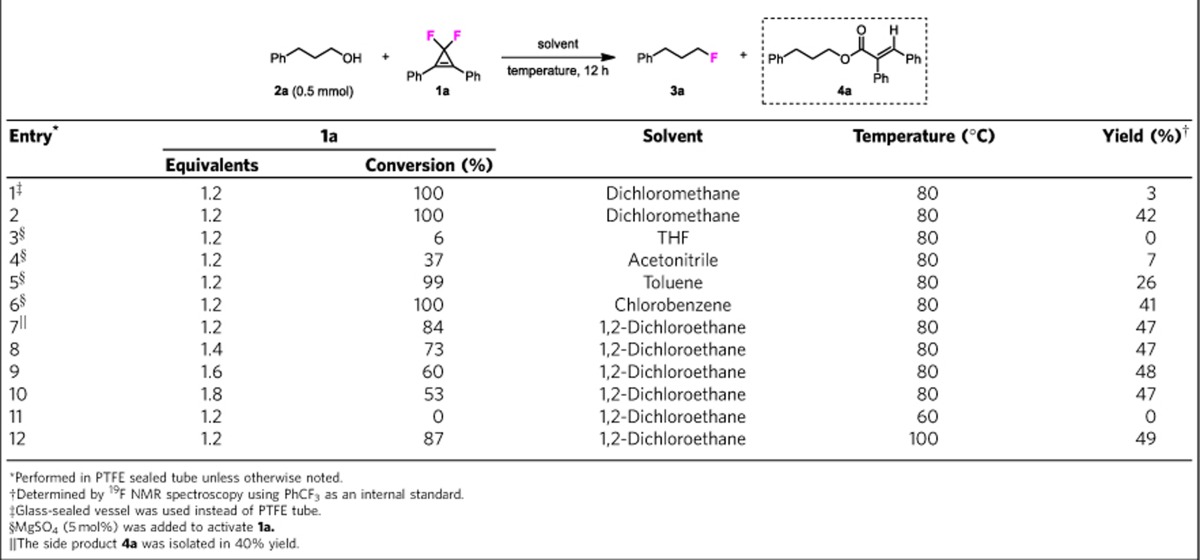
Initial deoxyfluorination of alcohols with CpFluor 1a.

**Table 2:**
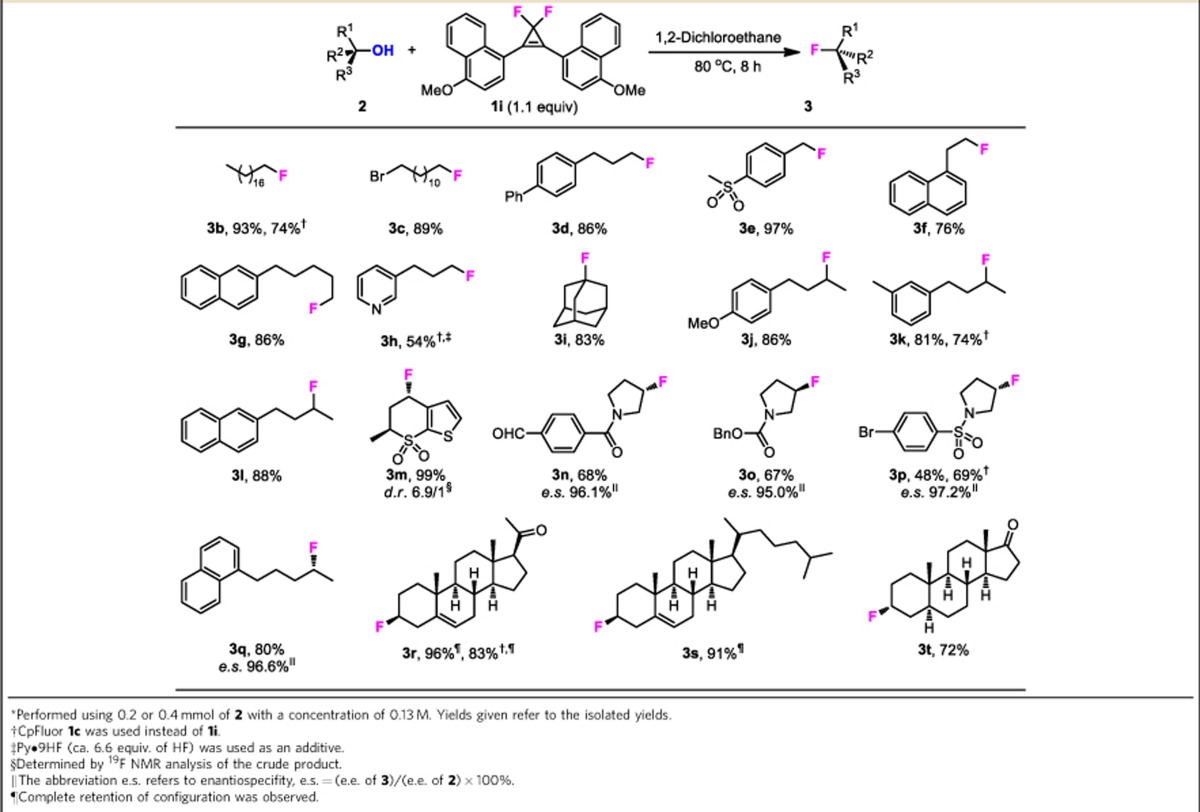
Deoxyfluorination of monoalcohols with CpFluor 1i*.

**Table 3:**
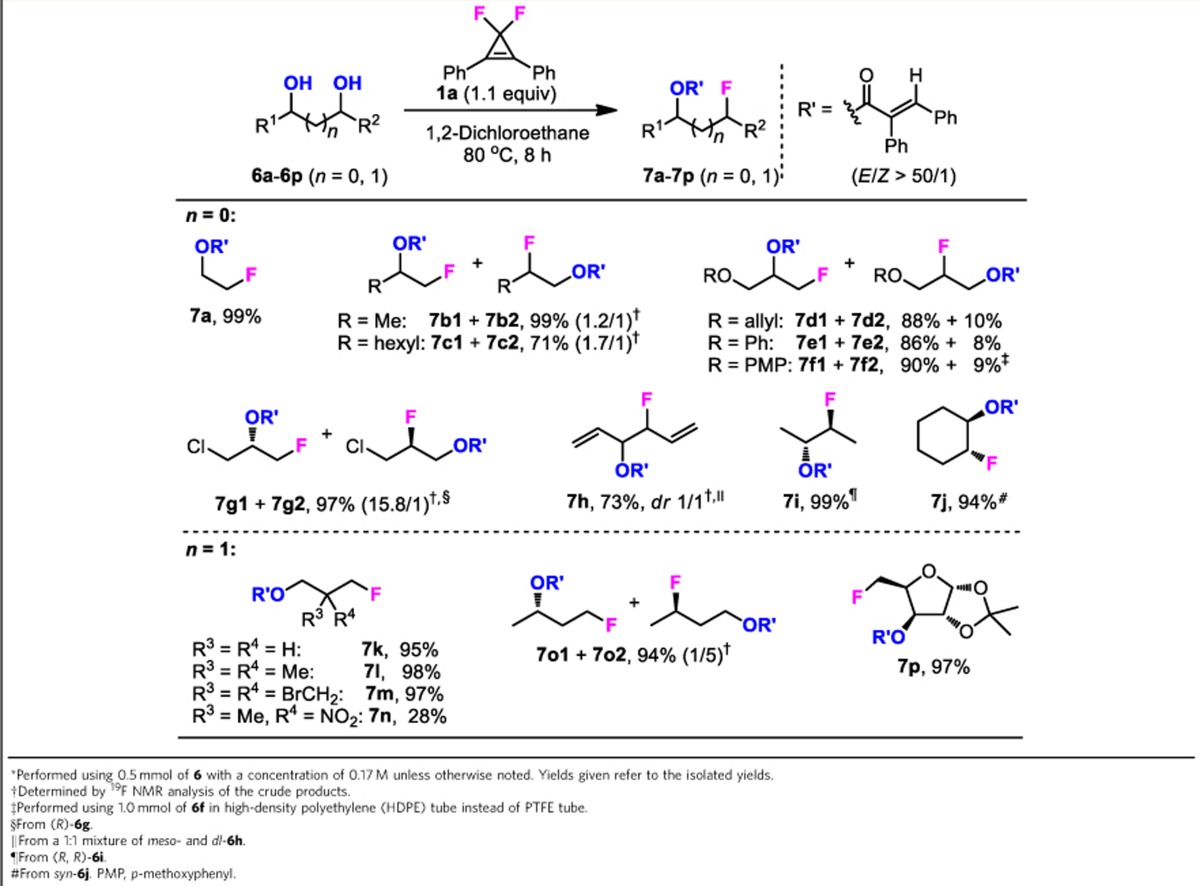
Deoxyfluorination of 1,2- and 1,3-diols with CpFluor 1a*.
