# Supplementary material for: Deoxyfluorination of alcohols with 3,3-difluoro-1,2-diarylcyclopropenes
Source: Nat Commun. 2016 Nov 14;7:13320. doi: 10.1038/ncomms13320 (PMC5114535; doi:10.1038/ncomms13320)
Supplement: Supplementary Information — Supplementary Figures 1-227, Supplementary Tables 1-19, Supplementary Methods and Supplementary References. [file ncomms13320-s1.pdf]

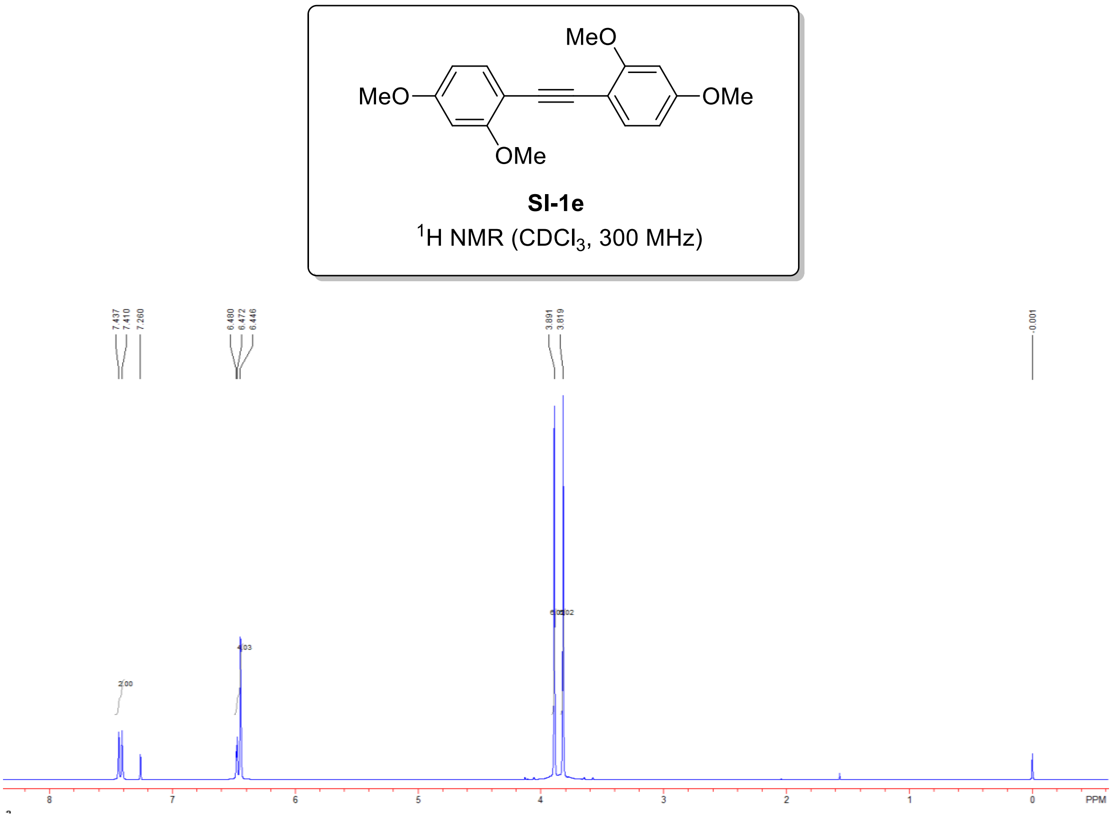

Supplementary Figure 1. ^{1}H NMR Spectrum of SI-1e

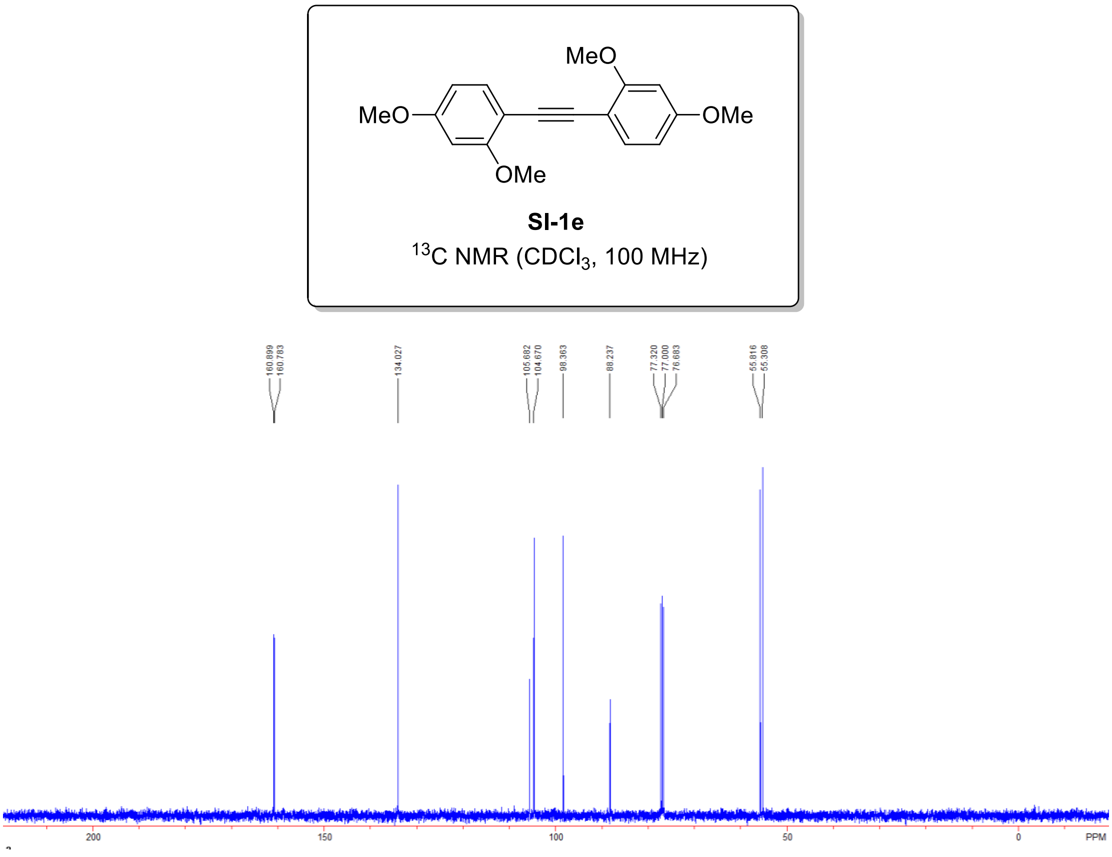

Supplementary Figure 2. ^{13}C NMR Spectrum of SI-1e

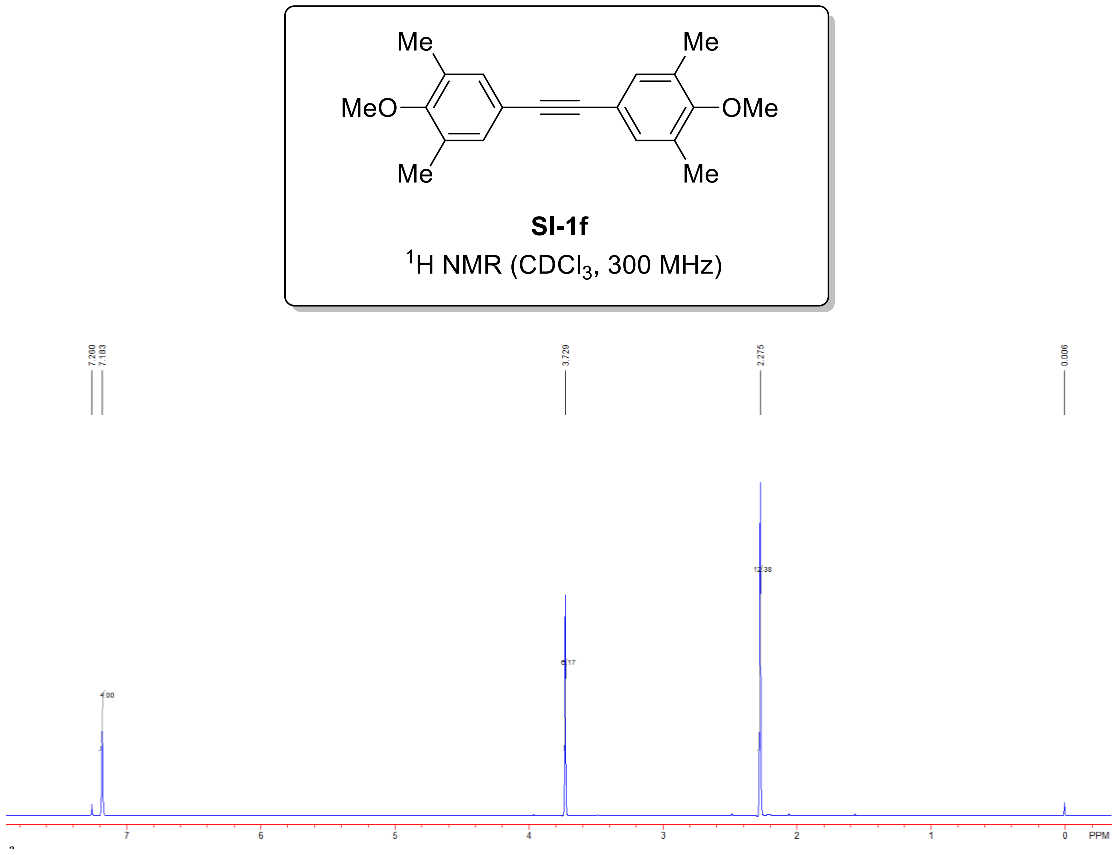

Supplementary Figure 3. ^{1}**H NMR Spectrum of SI-1f**

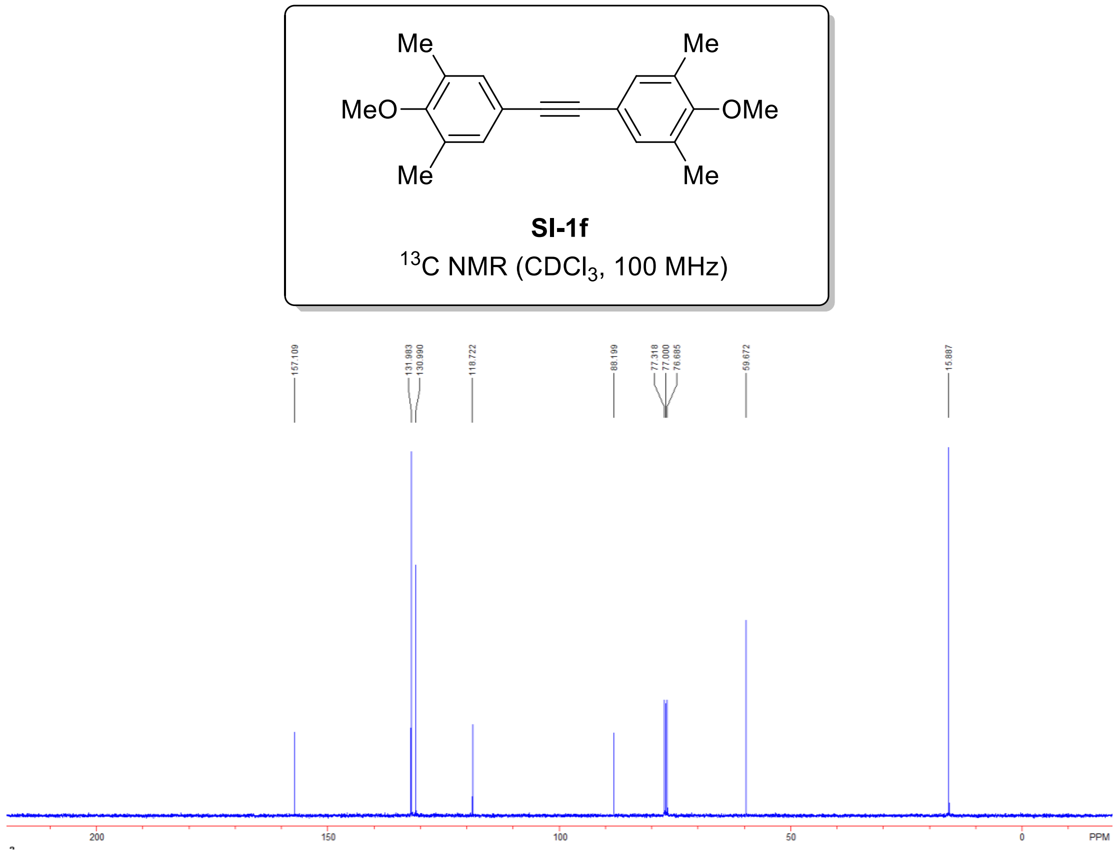

Supplementary Figure 4. ^{13}**C NMR Spectrum of SI-1f**

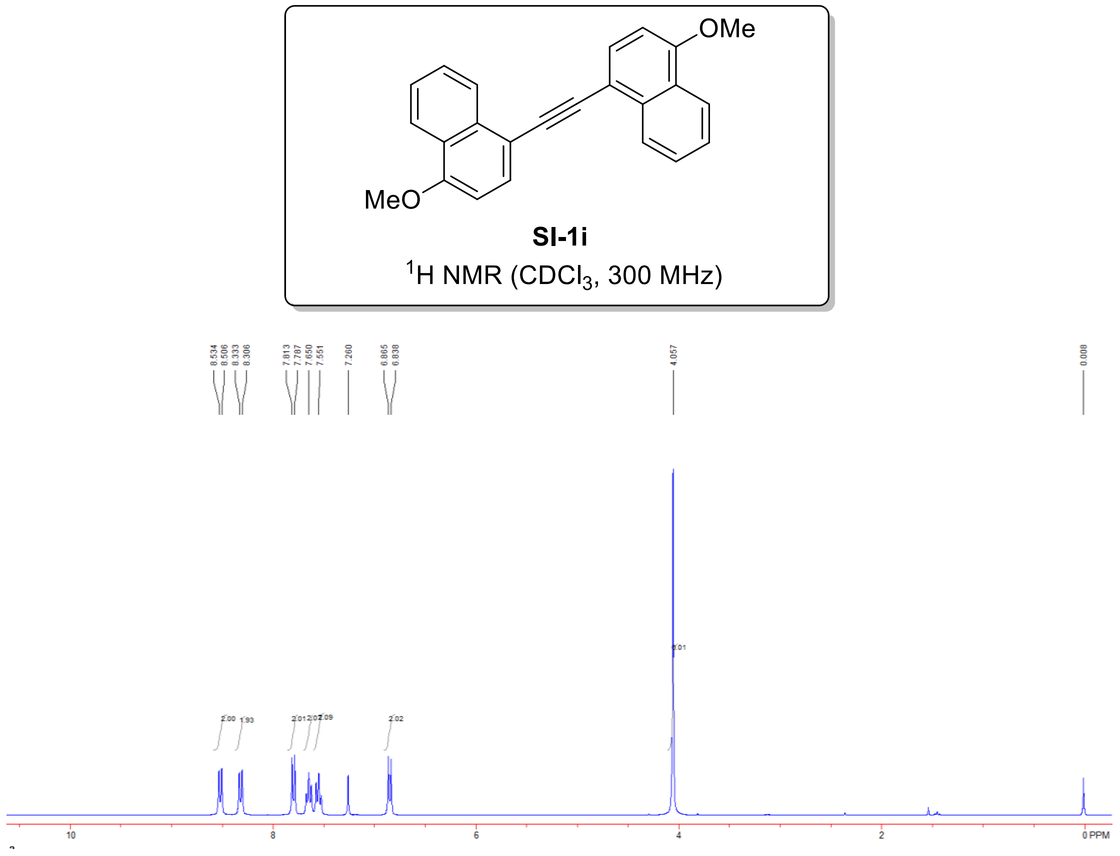

Supplementary Figure 5. ^{1}H NMR Spectrum of SI-1i

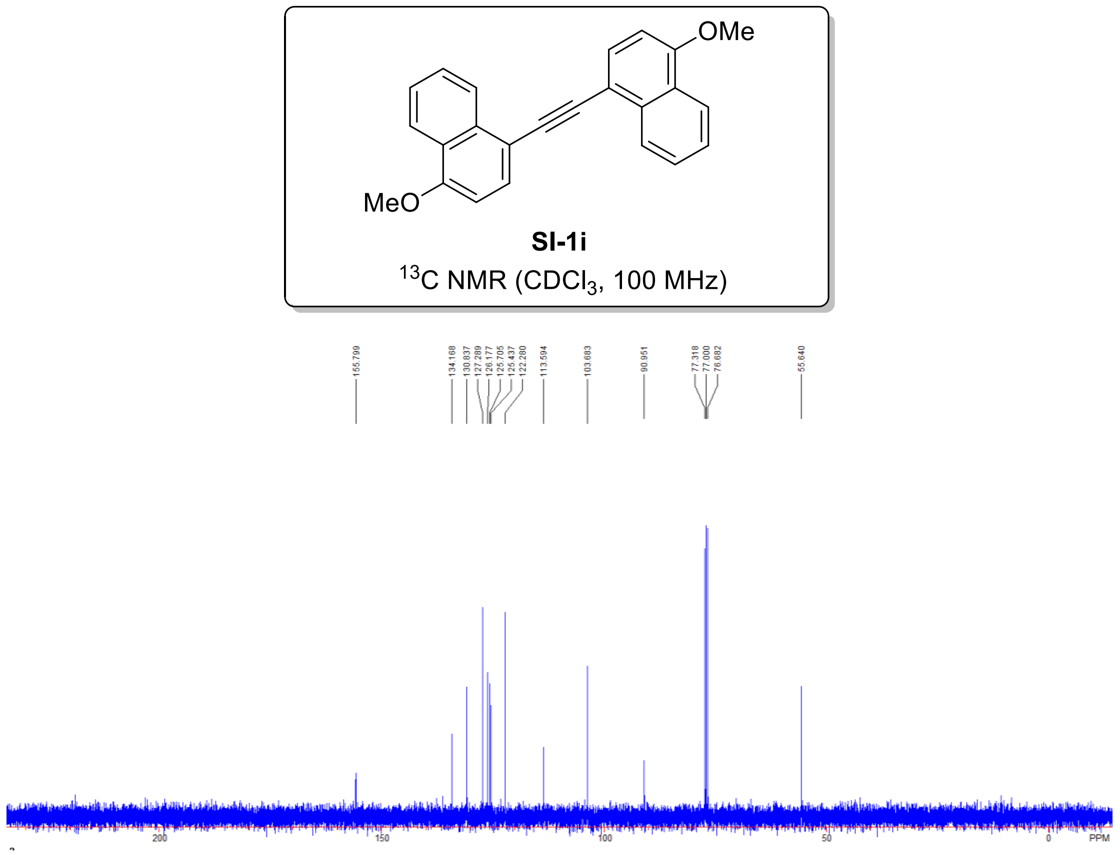

Supplementary Figure 6. ^{13}C NMR Spectrum of SI-1i

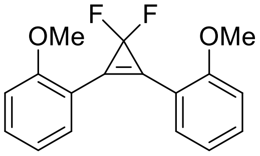

1b

^{1}H NMR ($CDCl_3$, 400 MHz)

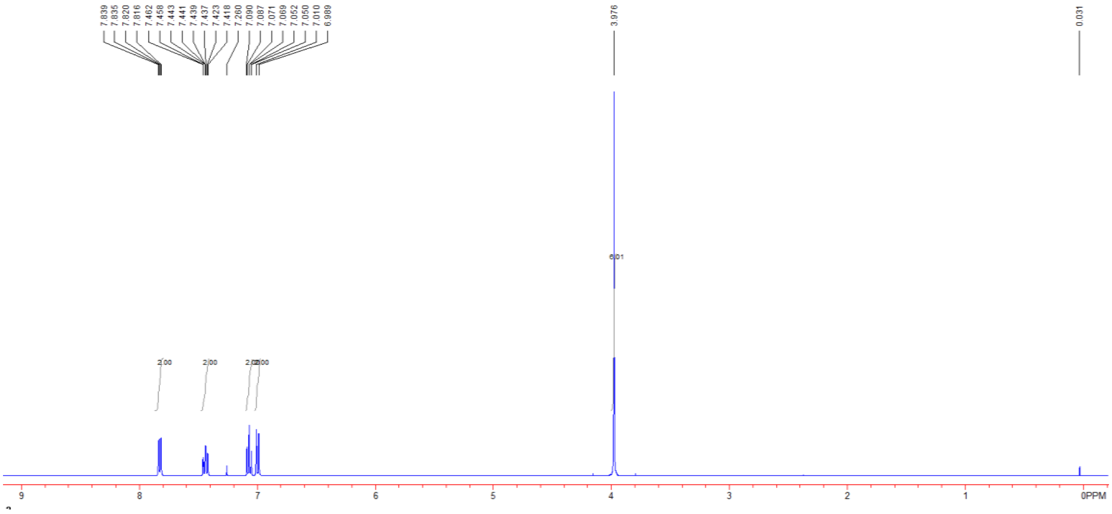

Supplementary Figure 7. ^{1}H NMR Spectrum of 1b

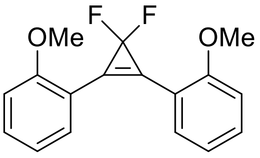

1b

^{19}F NMR ($CDCl_3$, 376 MHz)

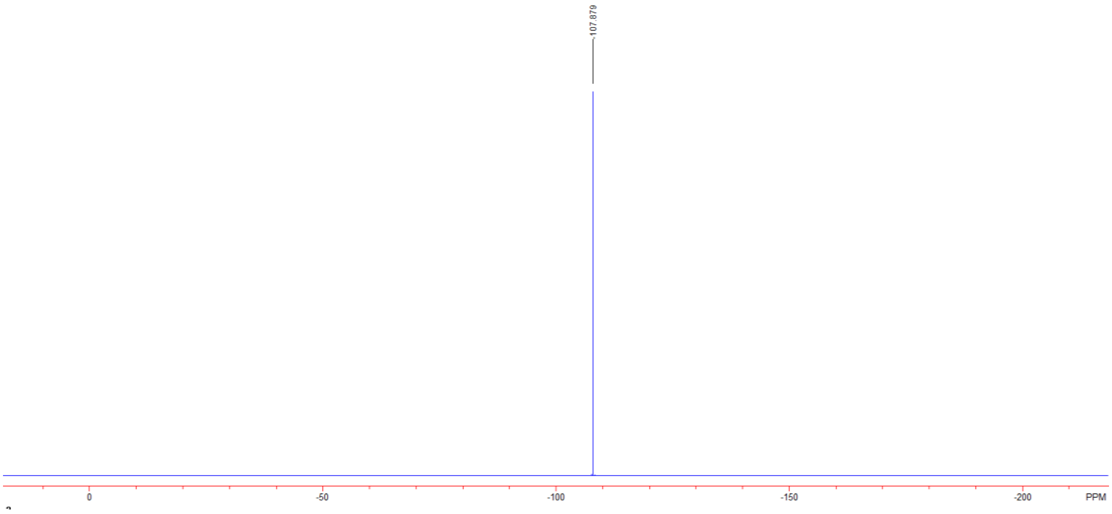

Supplementary Figure 8. ^{19}F NMR Spectrum of 1b

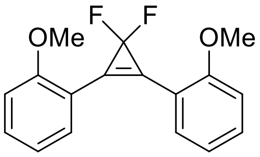

1b

^{13}C NMR ($CDCl_3$, 100 MHz)

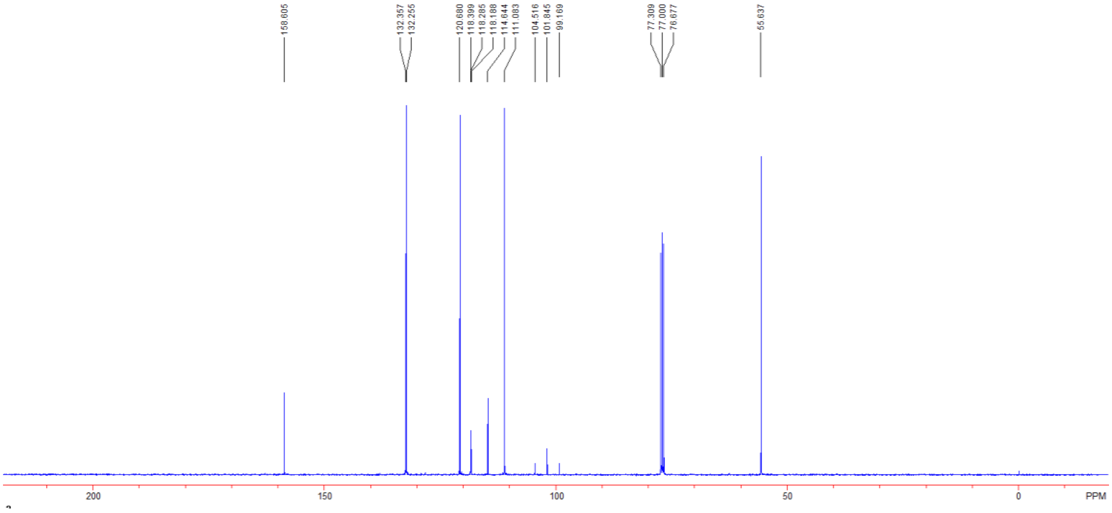

Supplementary Figure 9. ^{13}C NMR Spectrum of 1b

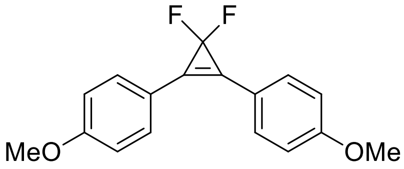

1c

^{1}H NMR ($CDCl_3$, 400 MHz)

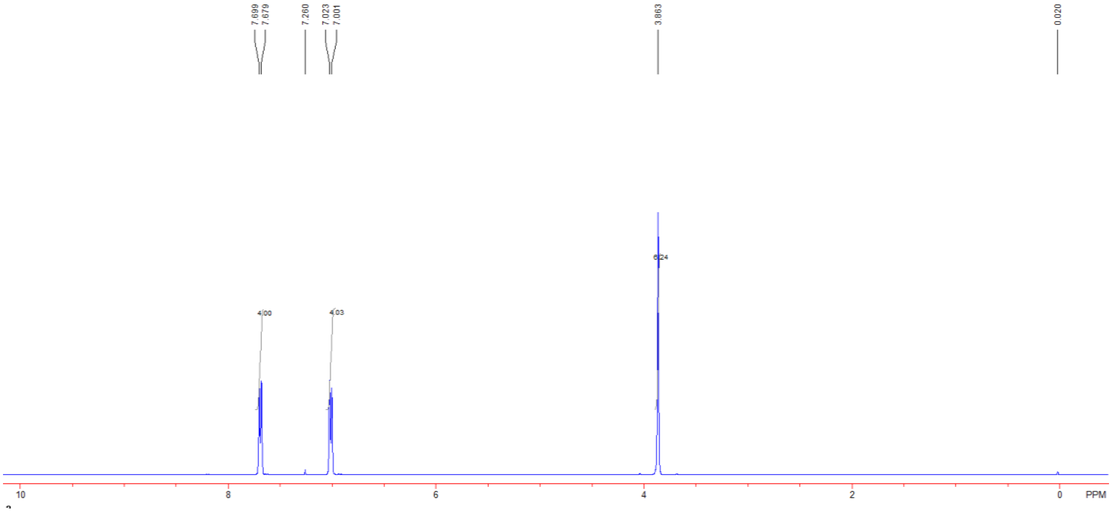

Supplementary Figure 10. ^{1}H NMR Spectrum of 1c

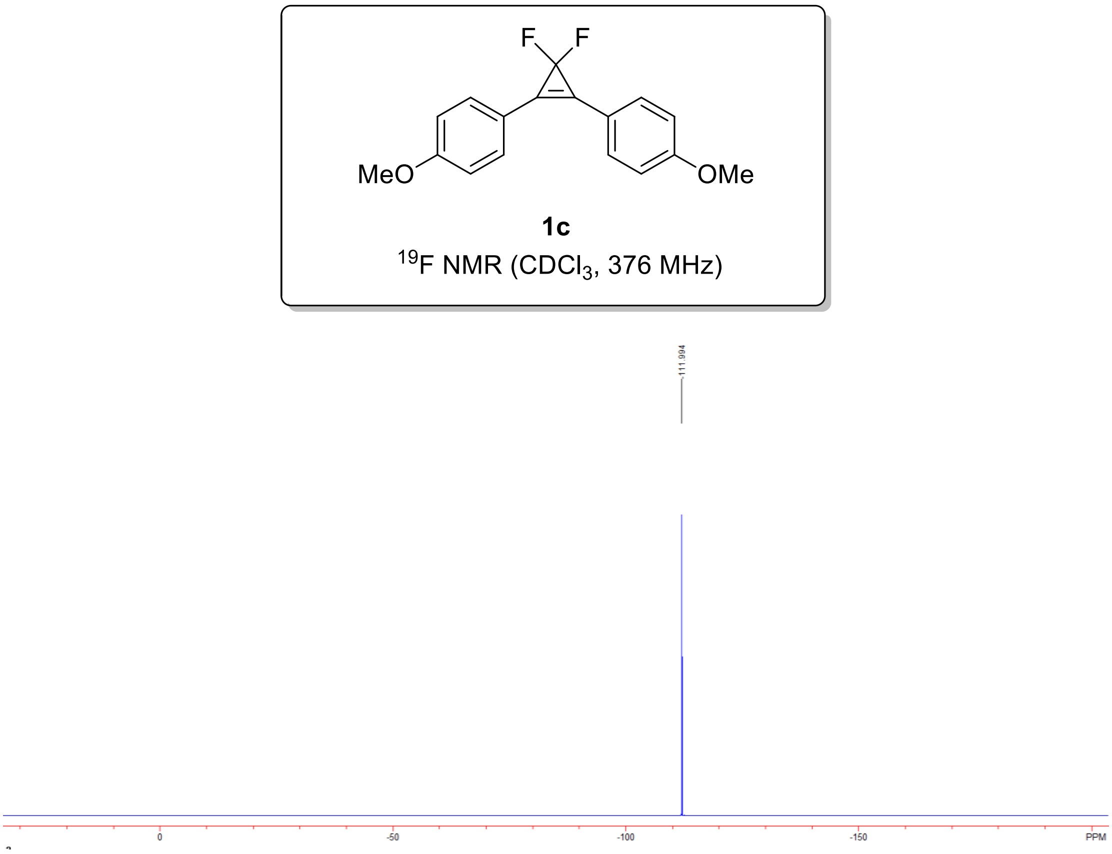

Supplementary Figure 11. ^{19}F NMR Spectrum of 1c

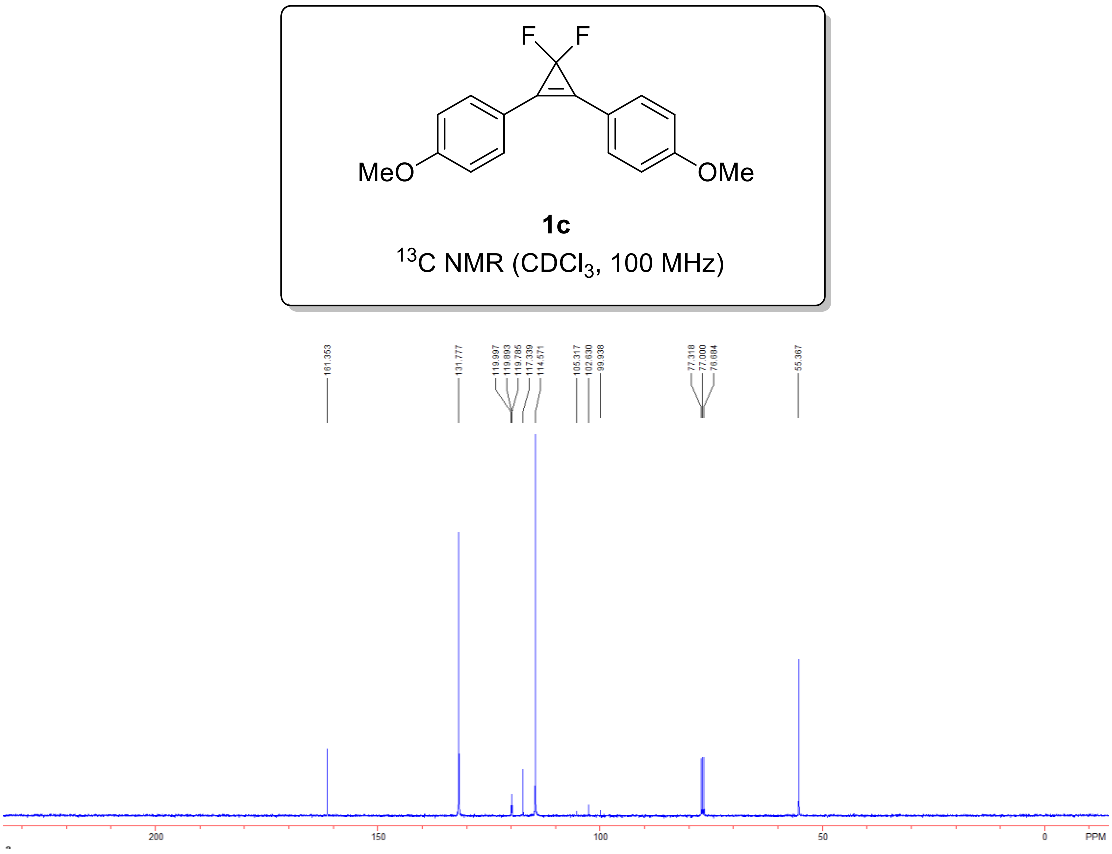

Supplementary Figure 12. ^{13}C NMR Spectrum of 1c

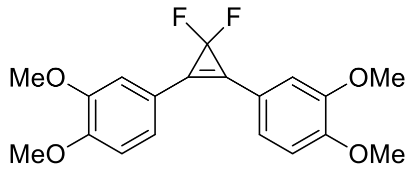

1d

^{1}H NMR ($CDCl_3$, 400 MHz)

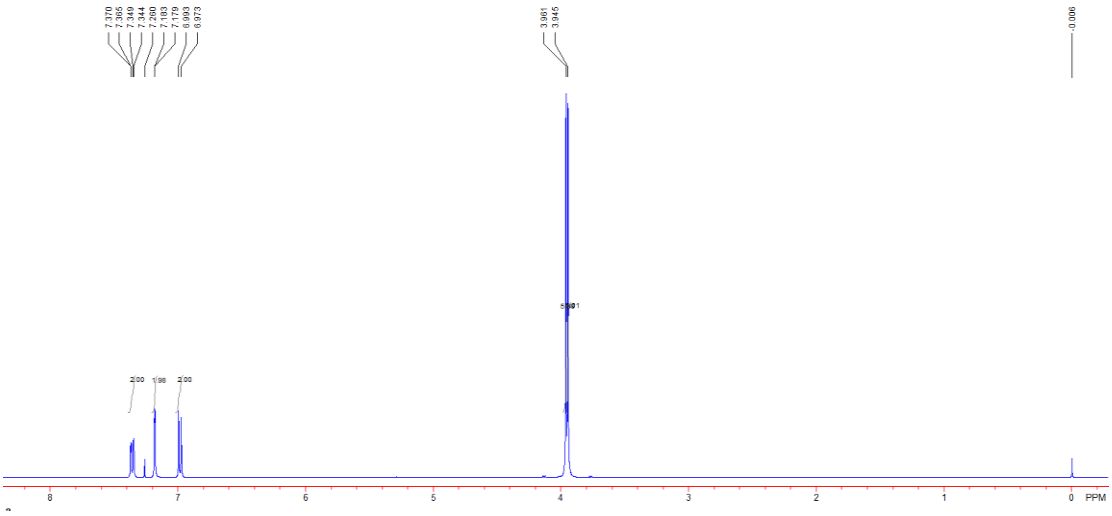

Supplementary Figure 13. ^{1}H NMR Spectrum of 1d

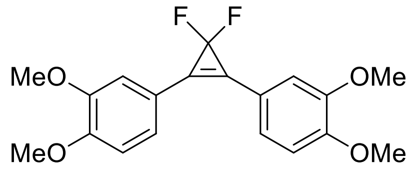

1d

^{19}F NMR ($CDCl_3$, 376 MHz)

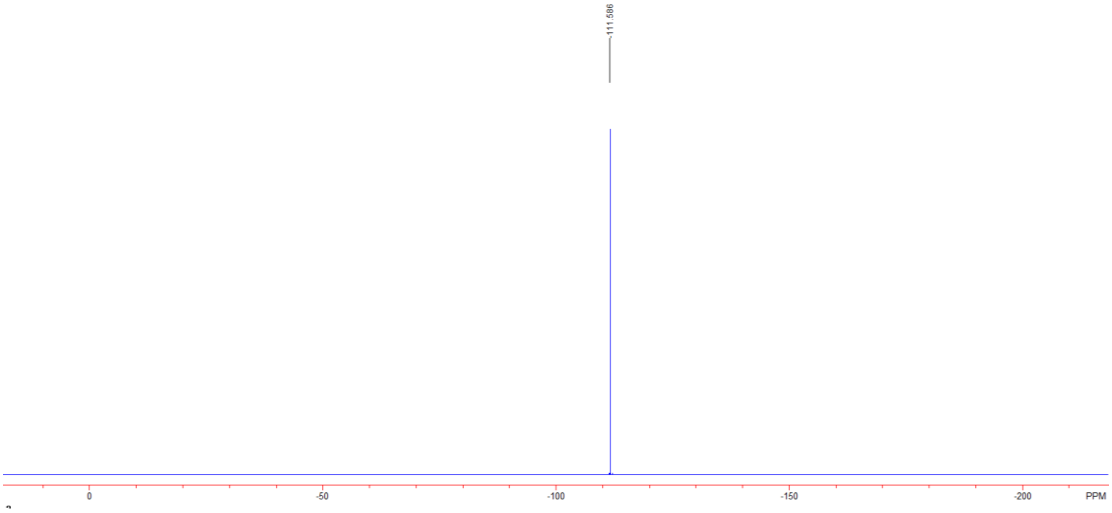

Supplementary Figure 14. ^{19}F NMR Spectrum of 1d

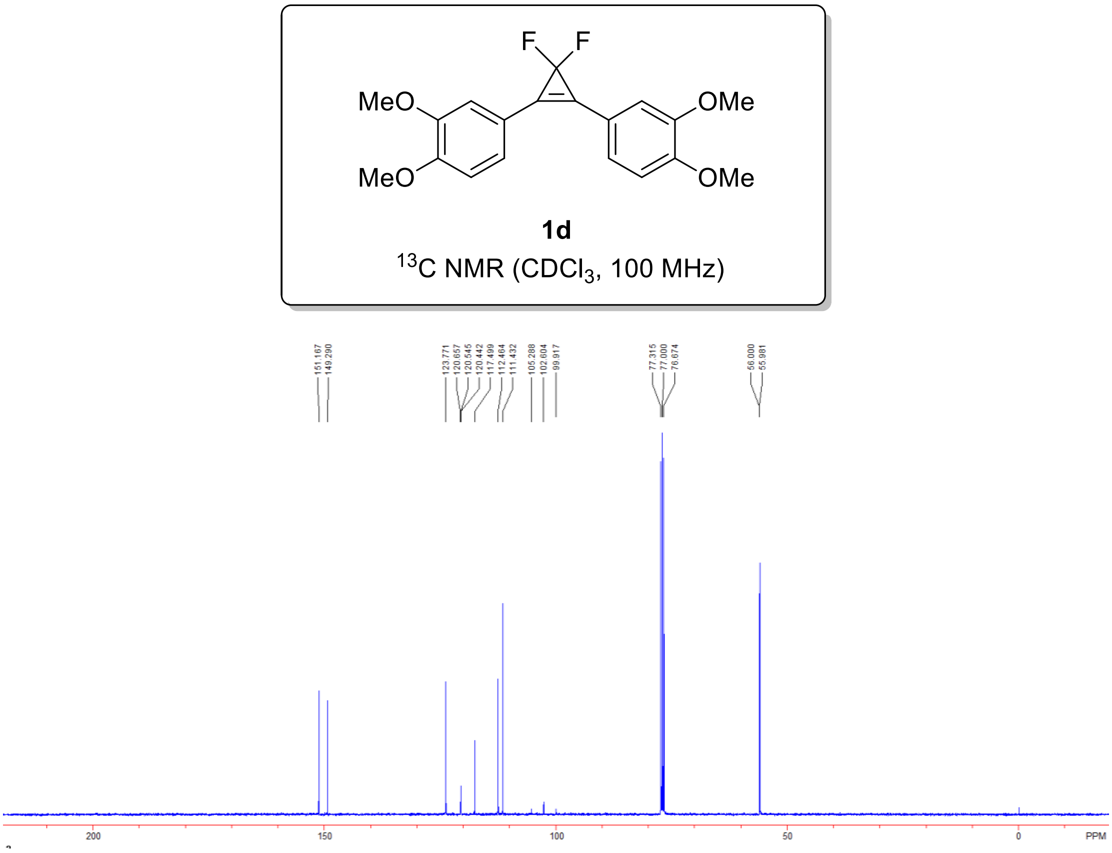

Supplementary Figure 15. ^{13}C NMR Spectrum of 1d

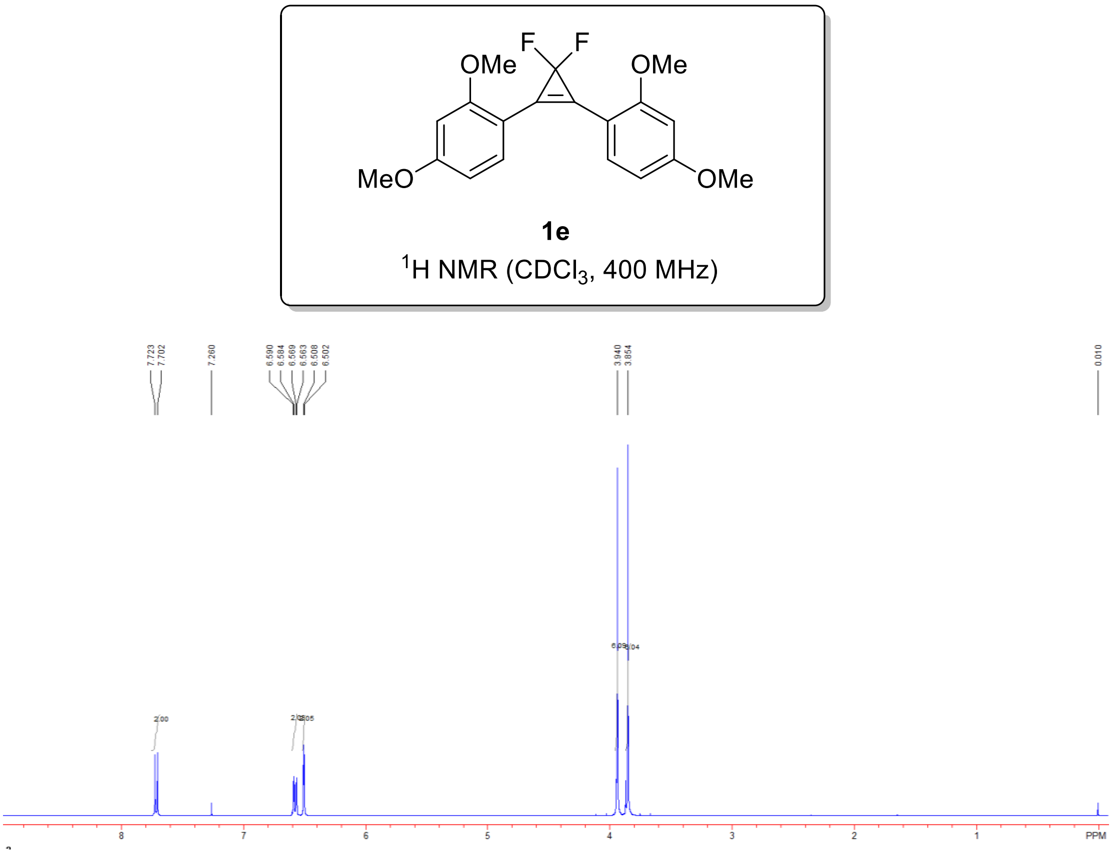

Supplementary Figure 16. ^{1}H NMR Spectrum of 1e

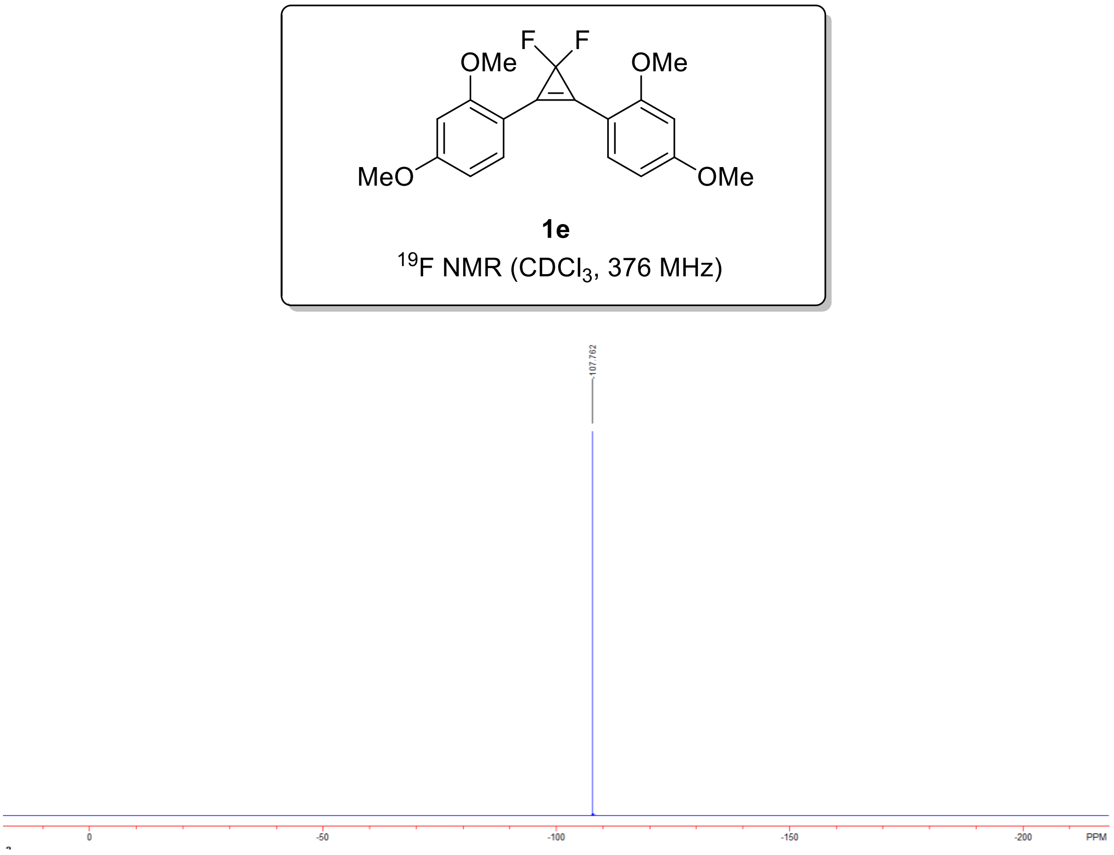

Supplementary Figure 17. ^{19}**F NMR Spectrum of 1e**

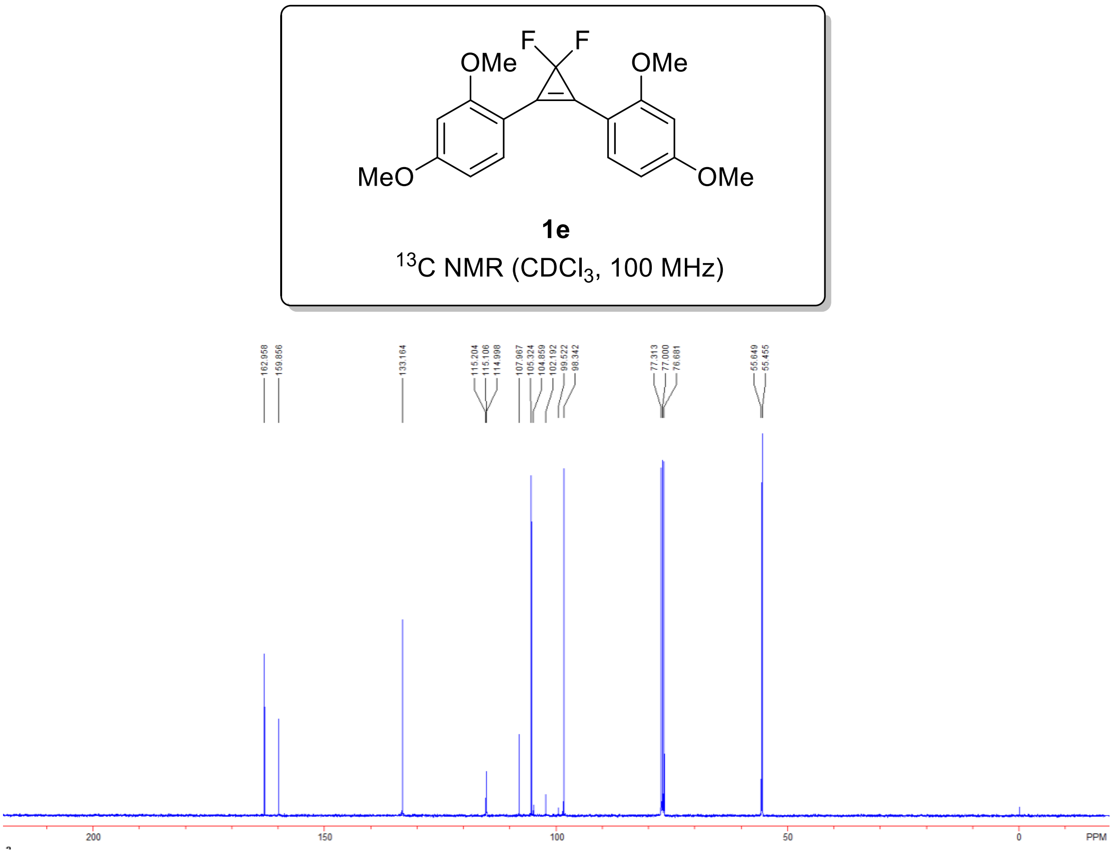

Supplementary Figure 18. ^{13}**C NMR Spectrum of 1e**

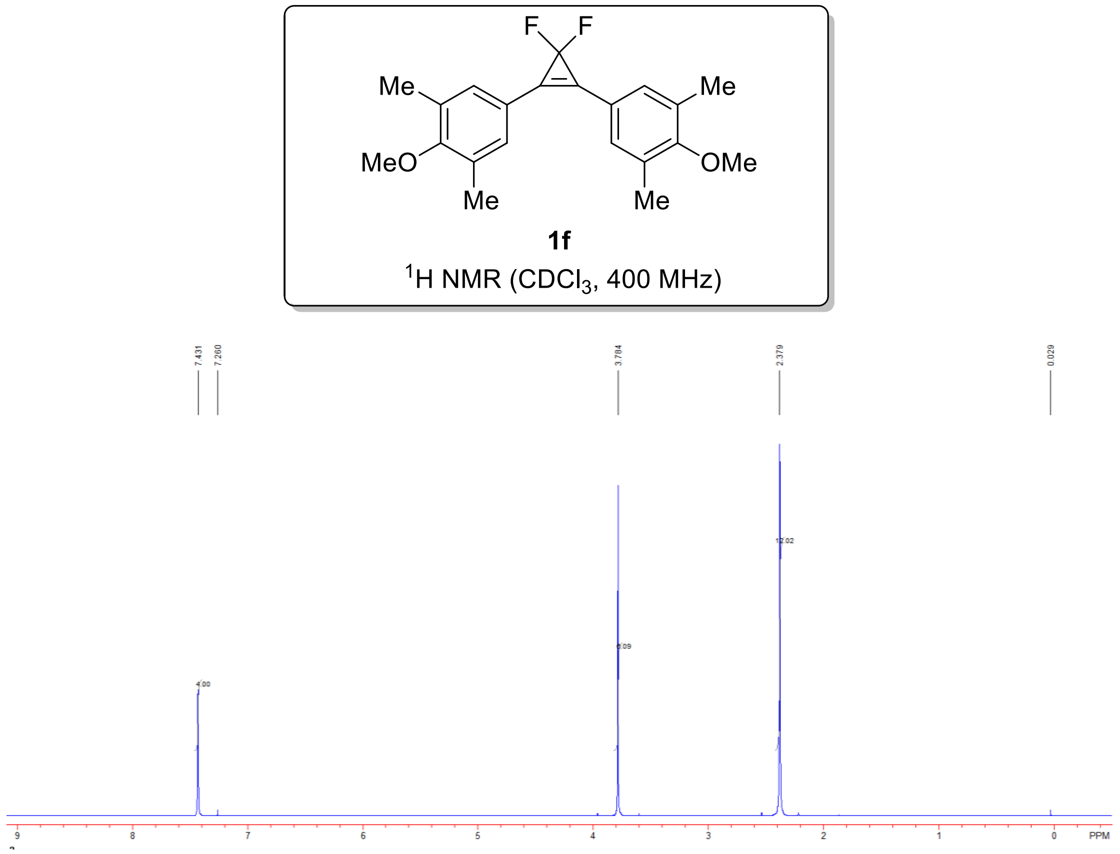

Supplementary Figure 19. ^{1}H NMR Spectrum of 1f

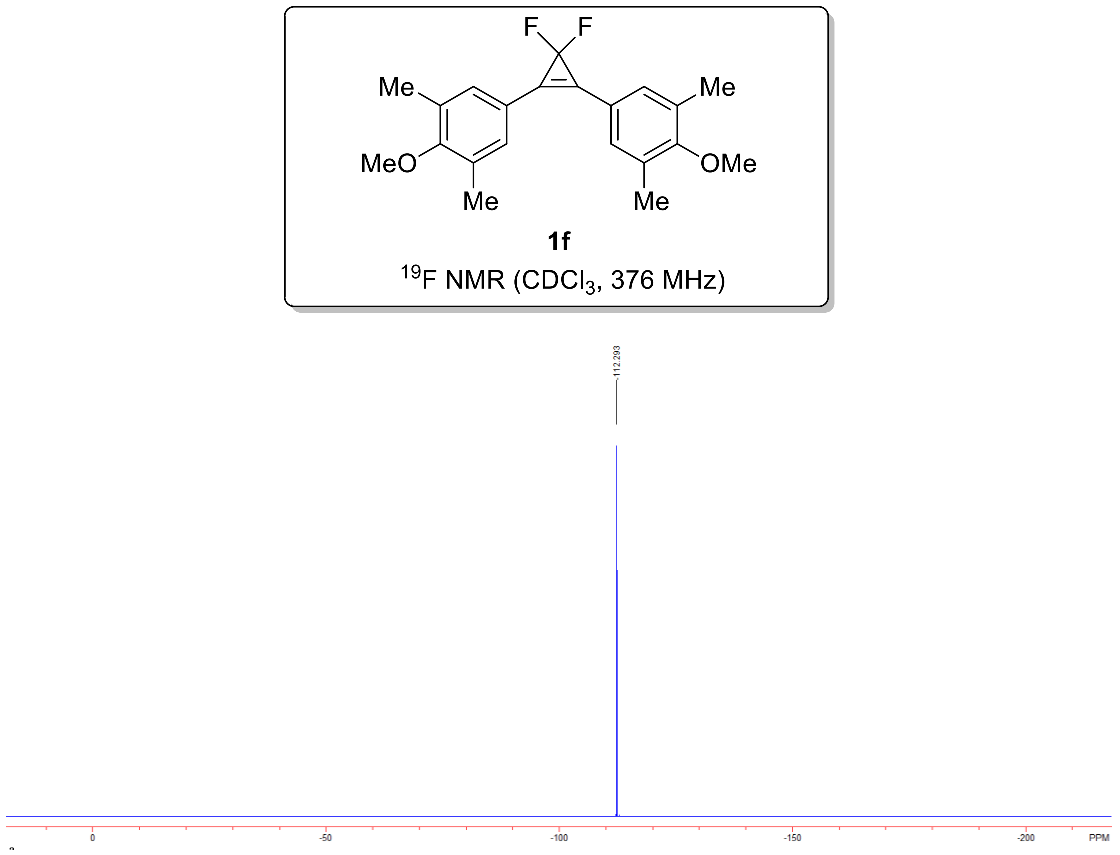

Supplementary Figure 20. ^{19}F NMR Spectrum of 1f

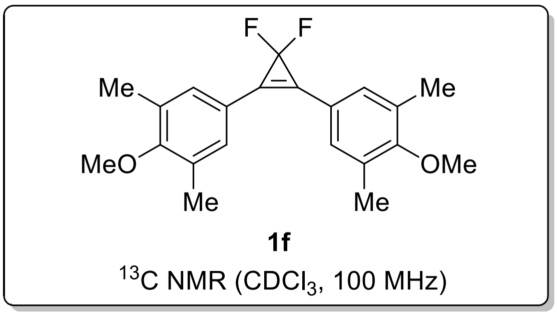

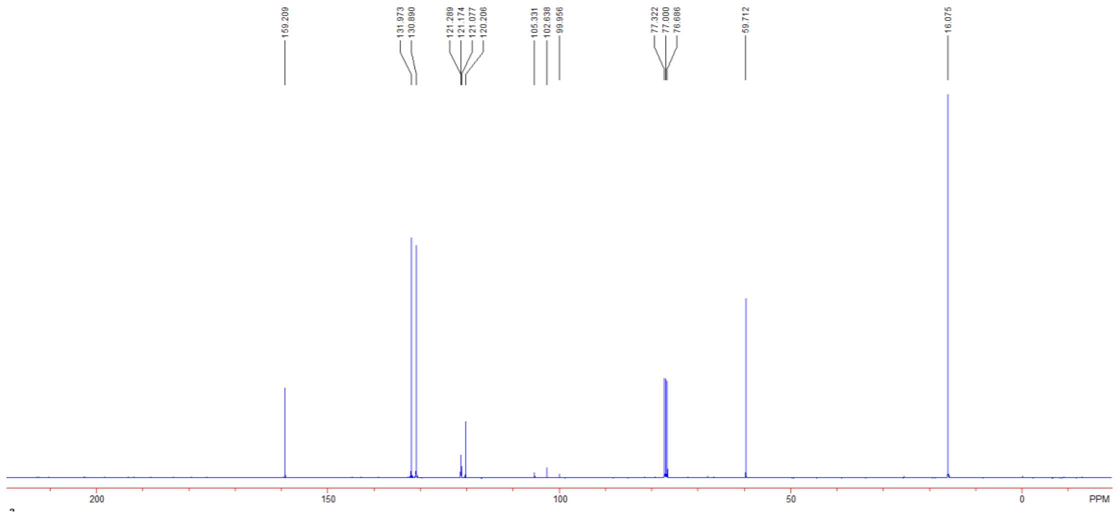

Supplementary Figure 21. ^{13}C NMR Spectrum of 1f

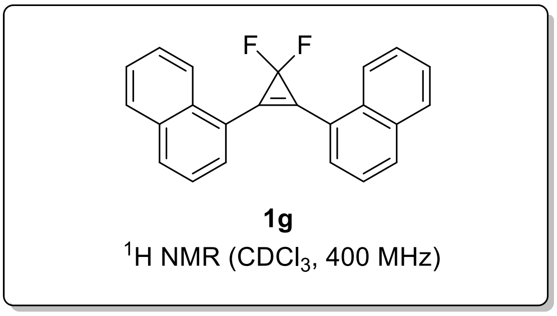

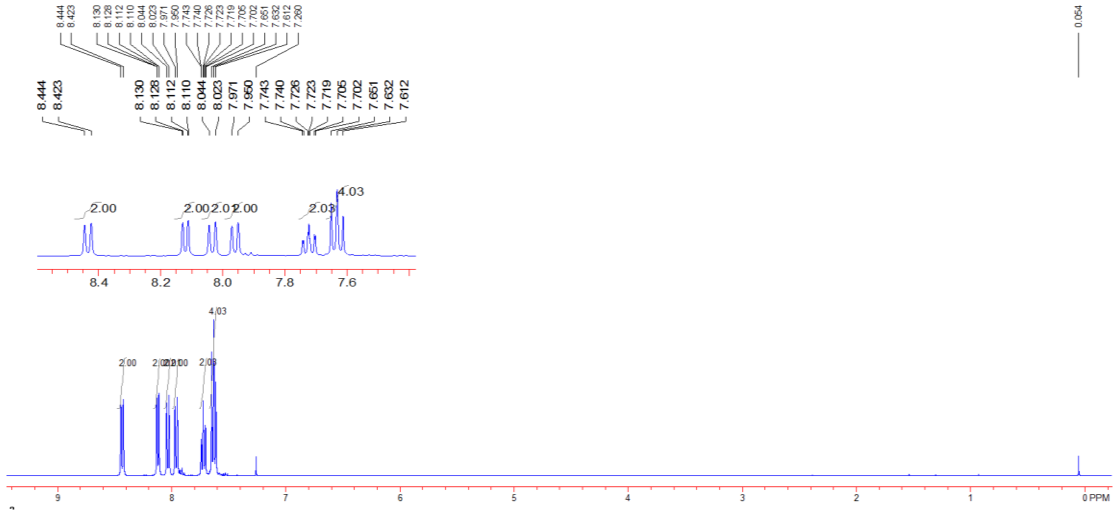

Supplementary Figure 22. ^{1}H NMR Spectrum of 1g

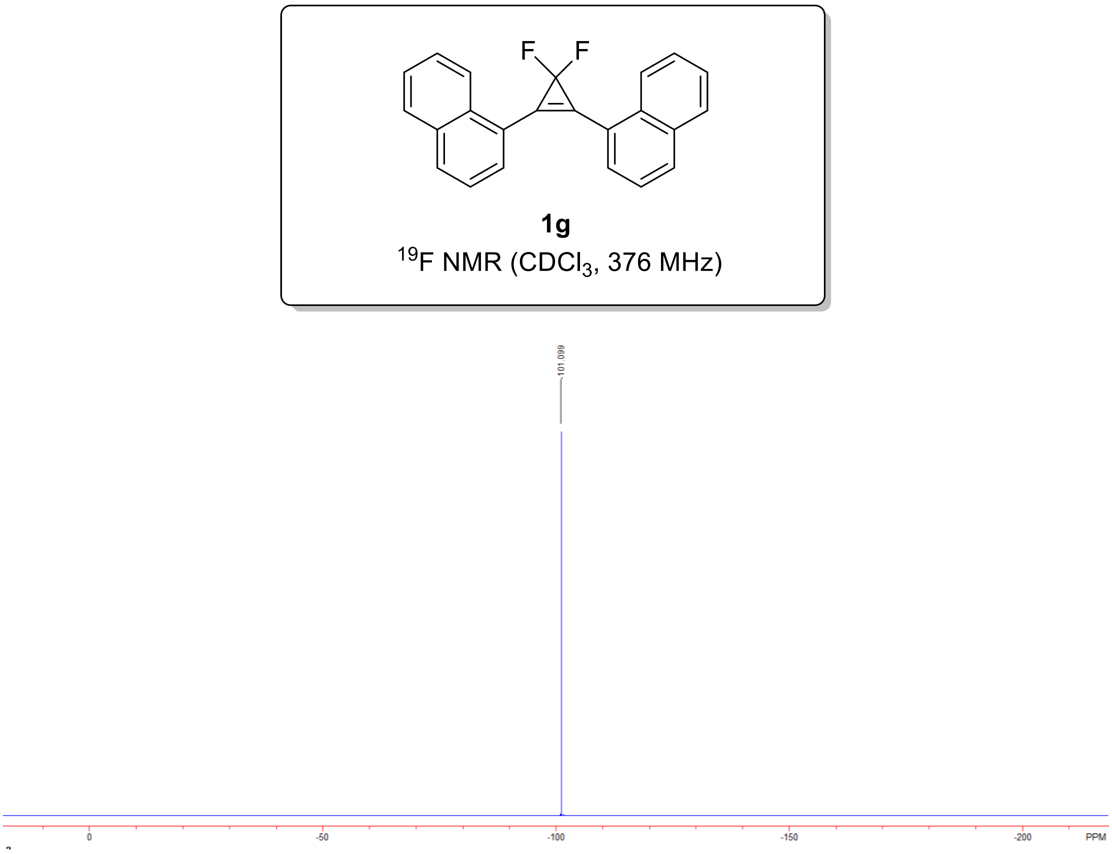

Supplementary Figure 23. ^{19}F NMR Spectrum of 1g

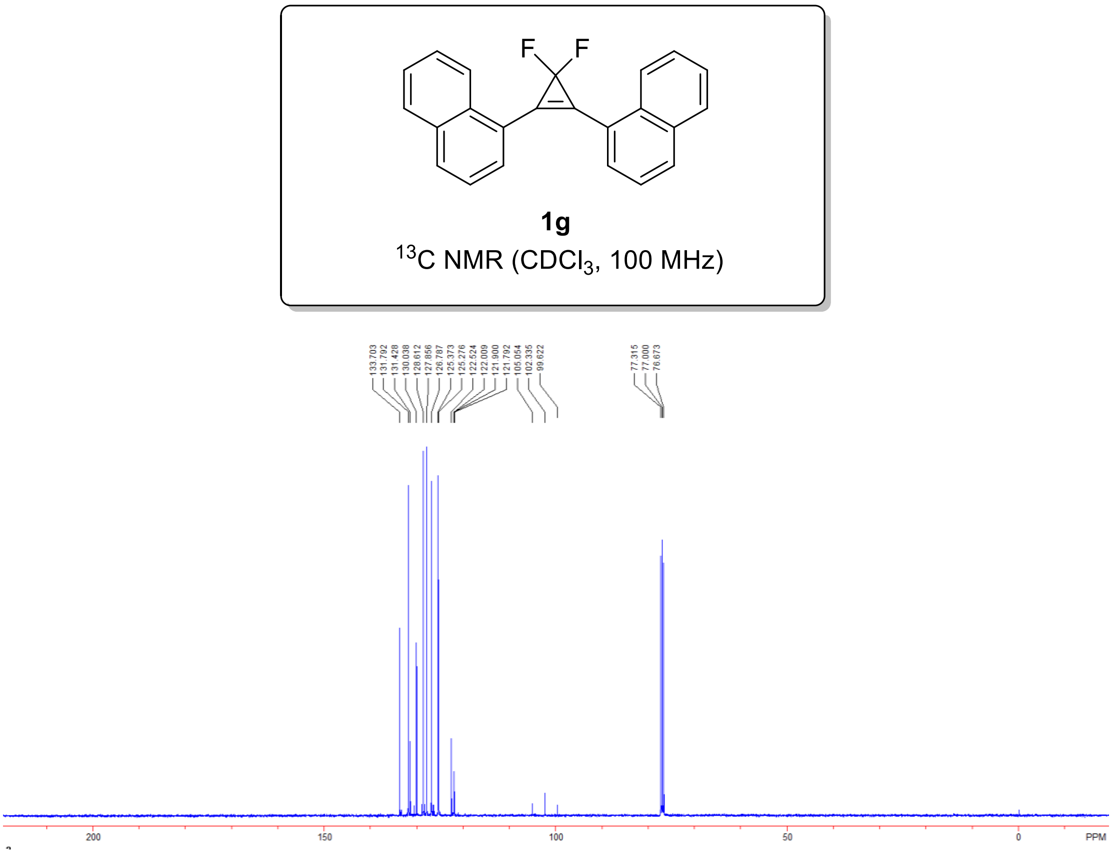

Supplementary Figure 24. ^{13}C NMR Spectrum of 1g

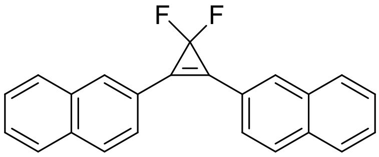

1h

^1H NMR ($CDCl_3$, 400 MHz)

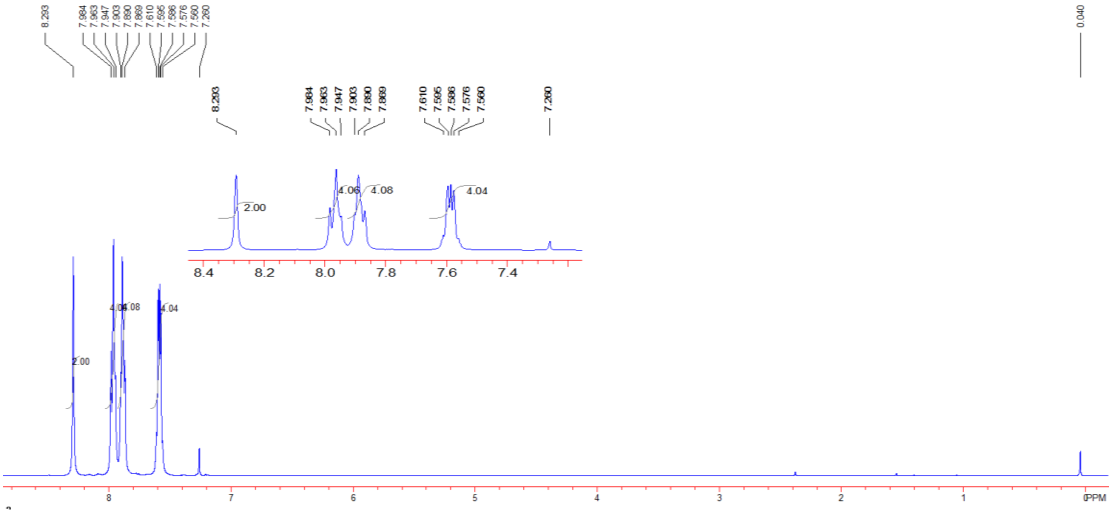

Supplementary Figure 25. ^{1}H NMR Spectrum of 1h

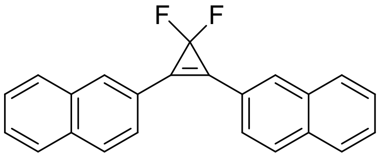

1h

^{19}F NMR ($CDCl_3$, 376 MHz)

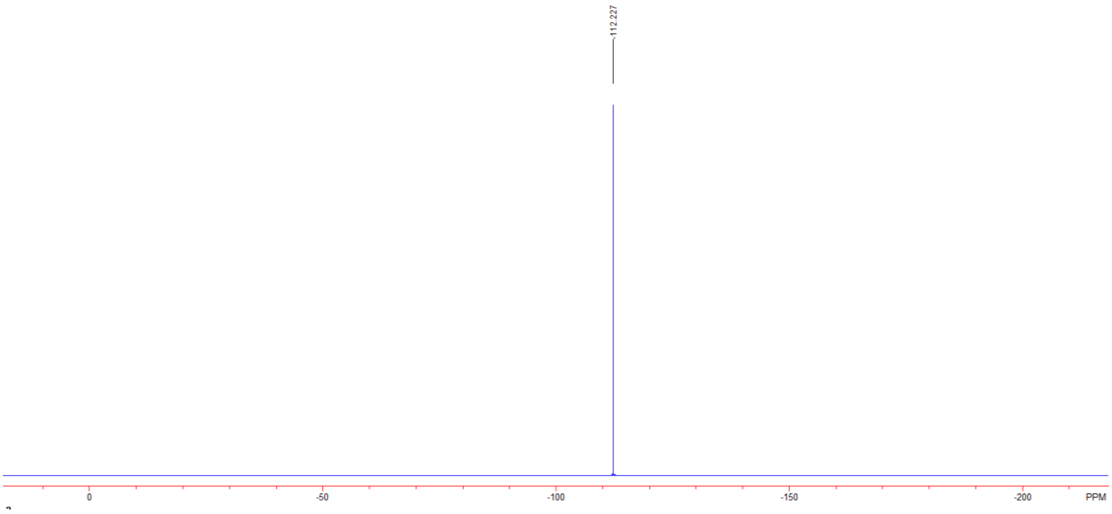

Supplementary Figure 26. ^{19}F NMR Spectrum of 1h

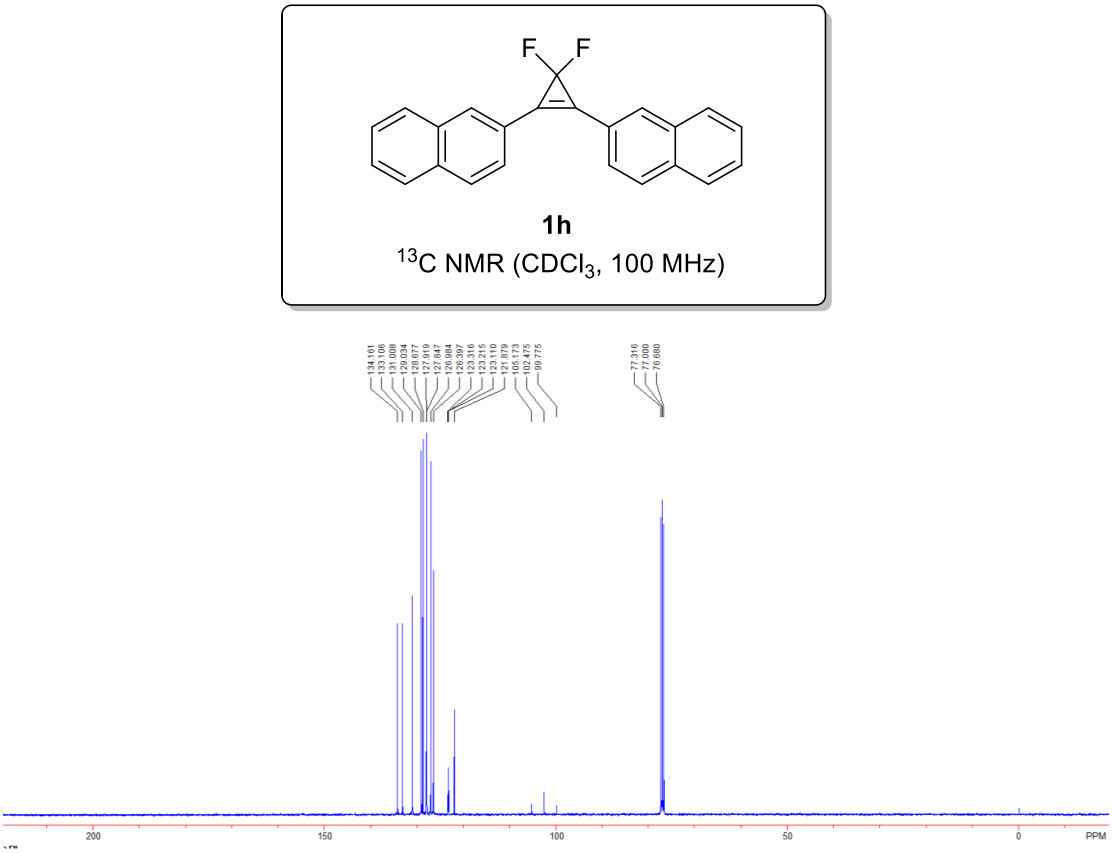

Supplementary Figure 27. ^{13}C NMR Spectrum of 1h

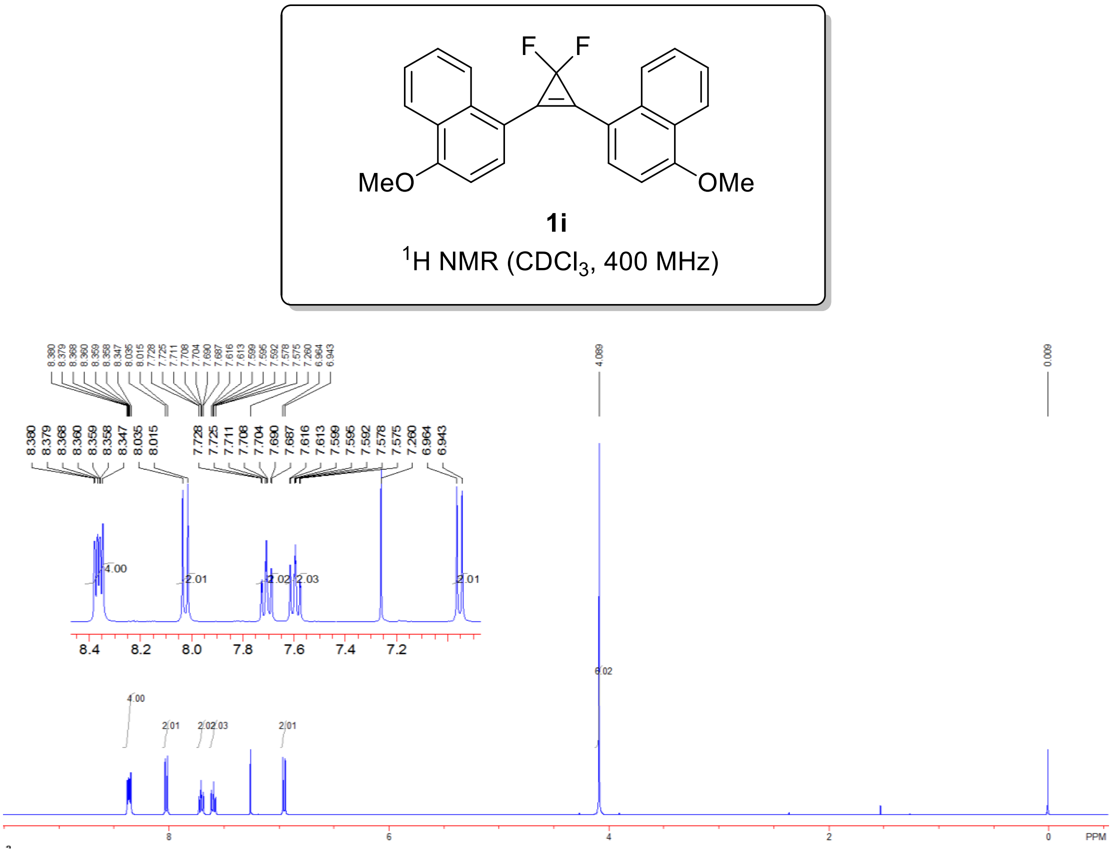

Supplementary Figure 28. ^{1}H NMR Spectrum of 1i

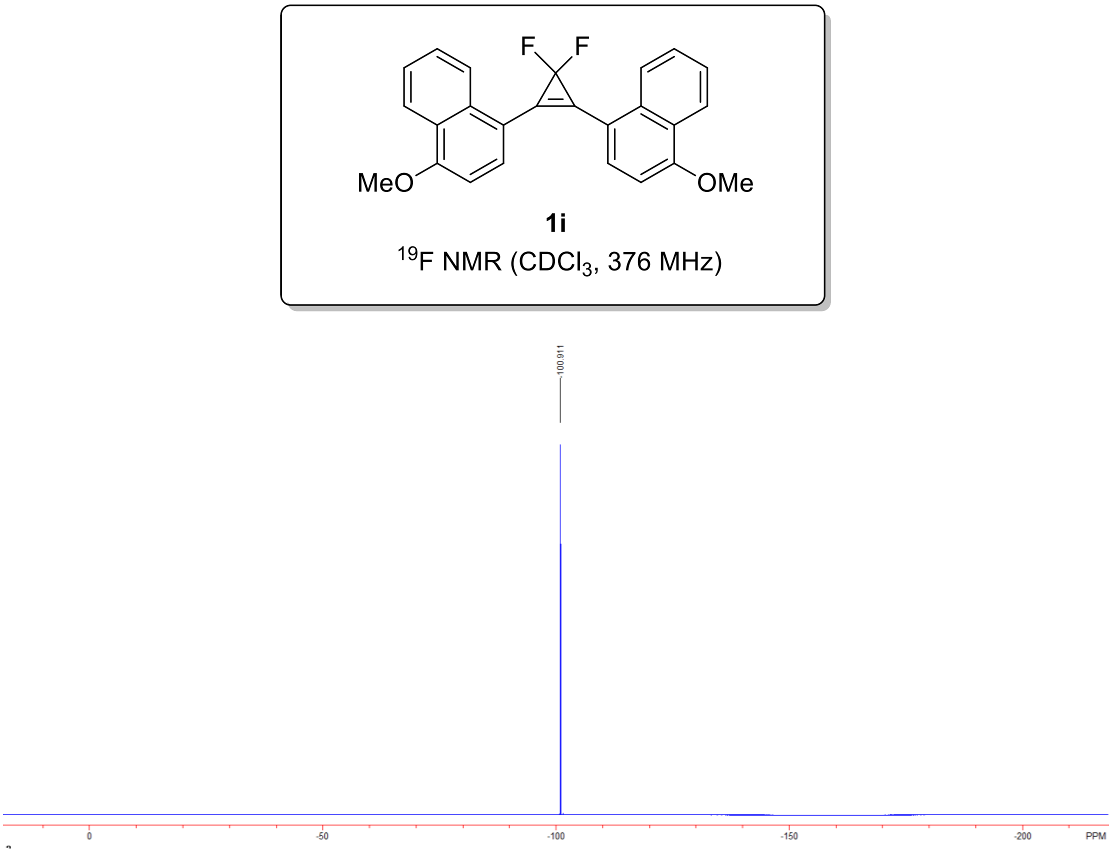

Supplementary Figure 29. ^{19}**F NMR Spectrum of 1i**

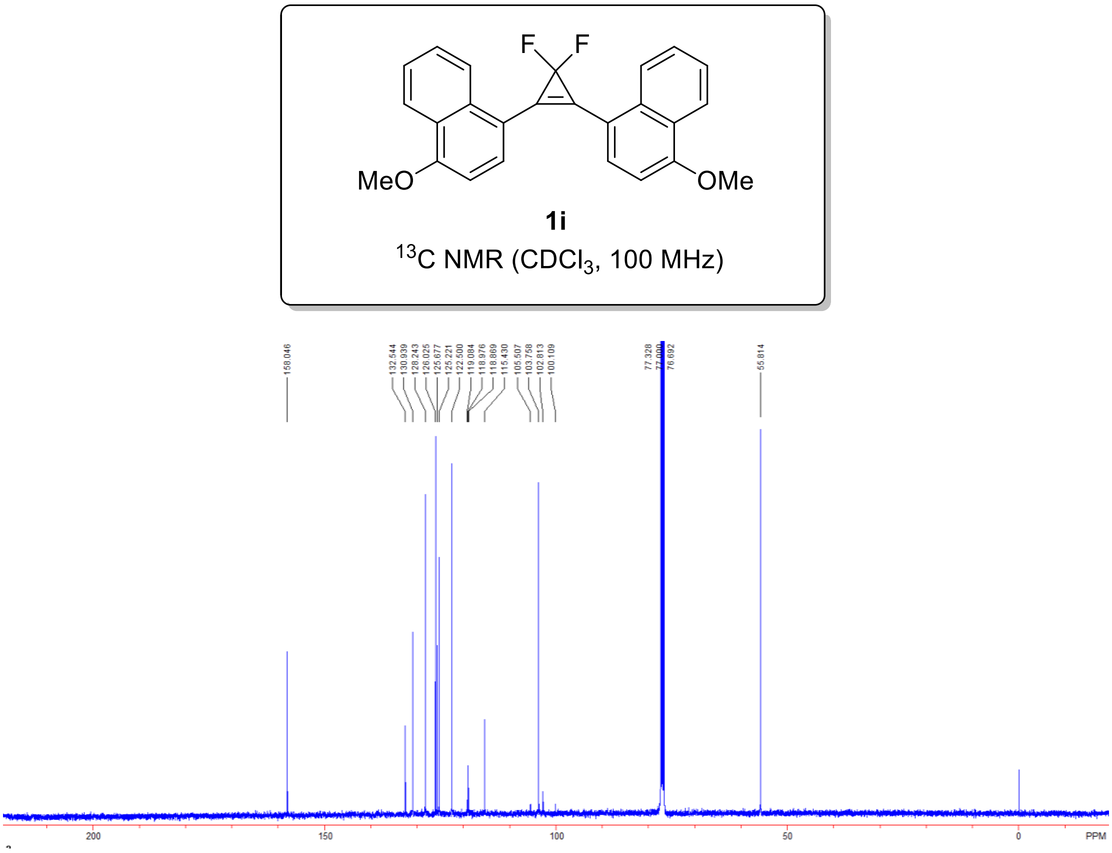

Supplementary Figure 30. ^{13}**C NMR Spectrum of 1i**

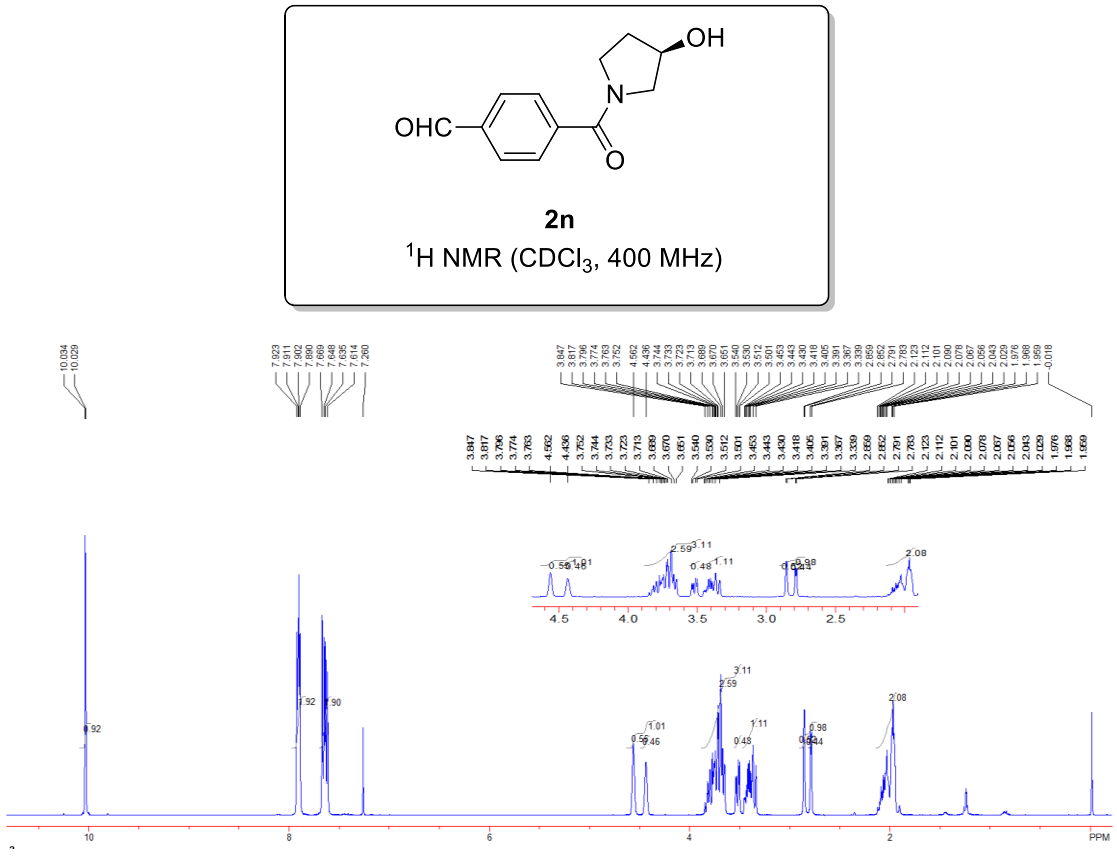

Supplementary Figure 31. ^{1}H NMR Spectrum of 2n

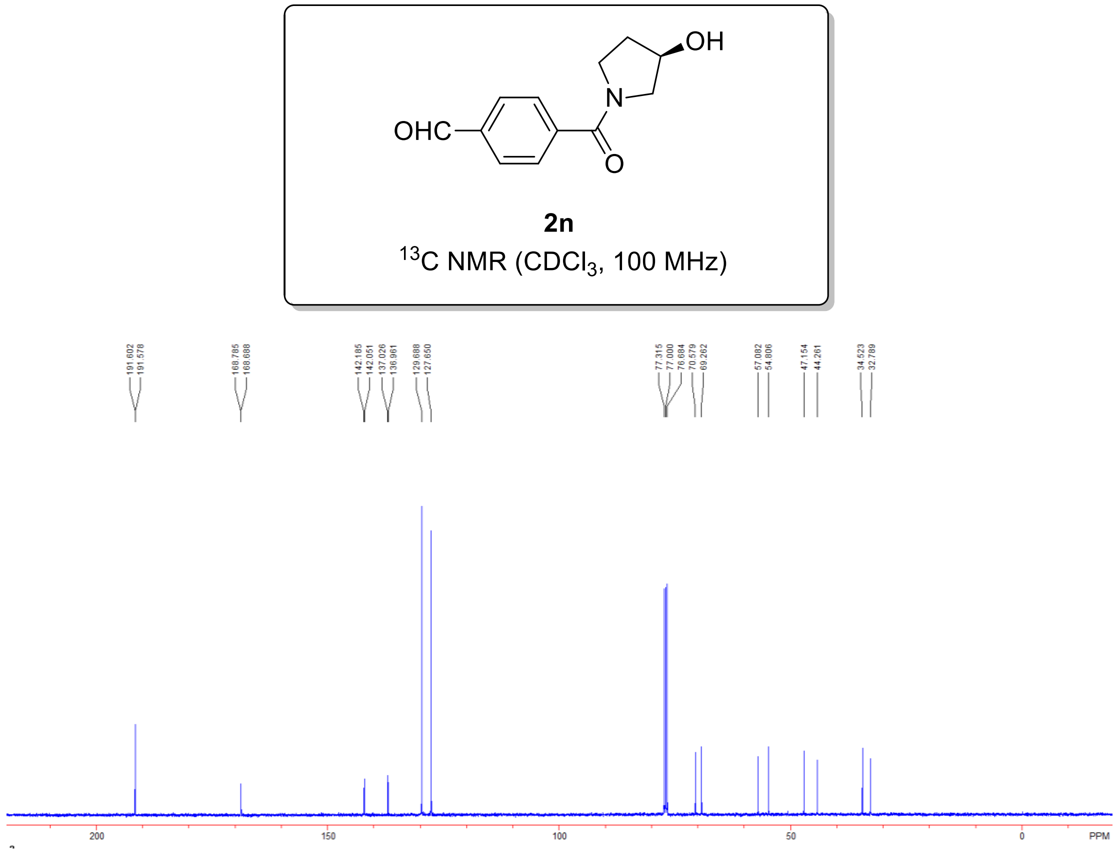

Supplementary Figure 32. ^{13}C NMR Spectrum of 2n

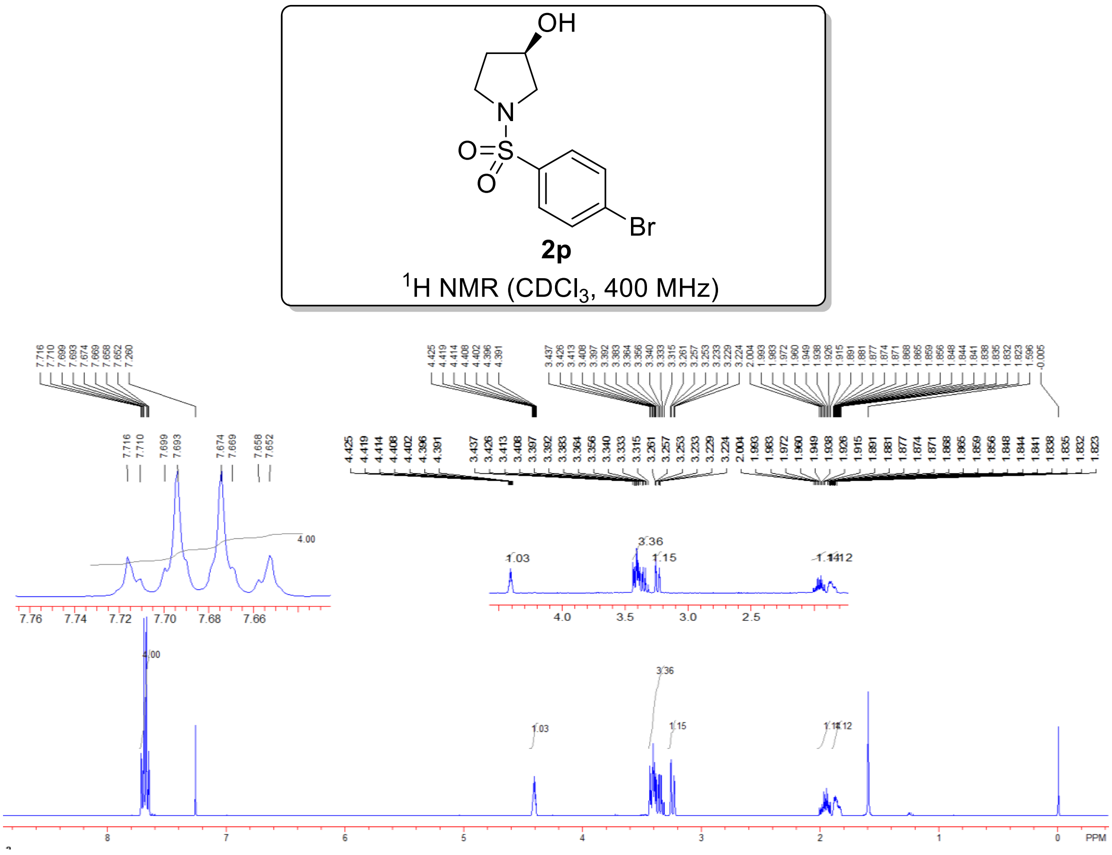

Supplementary Figure 33. ^{1}**H NMR Spectrum of 2p**

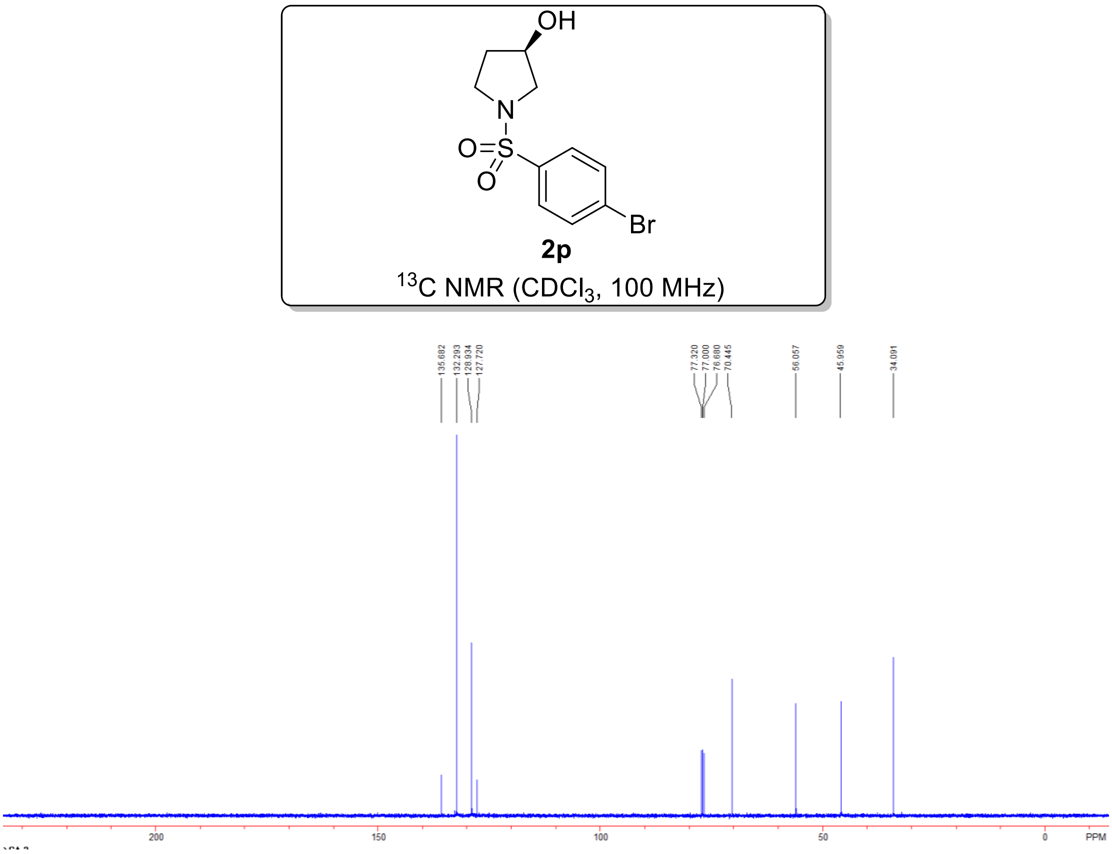

Supplementary Figure 34. ^{13}**C NMR Spectrum of 2p**

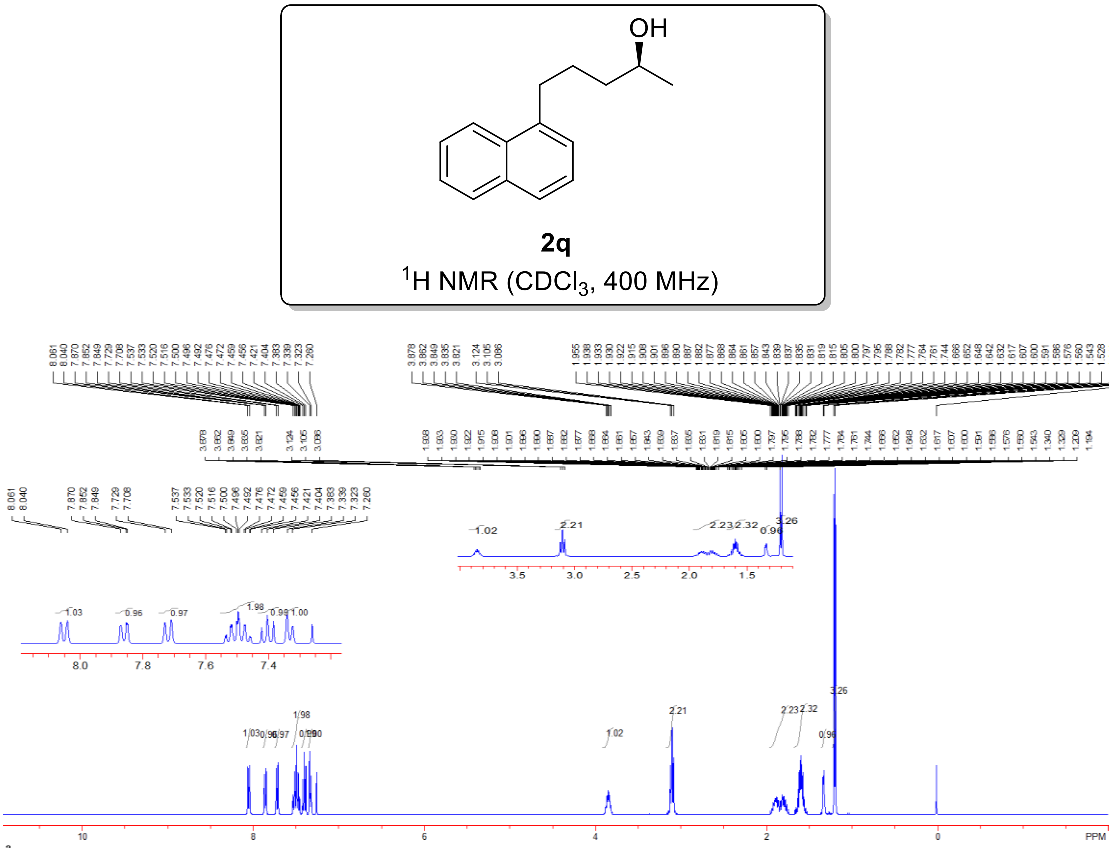

Supplementary Figure 35. ^{1}H NMR Spectrum of 2q

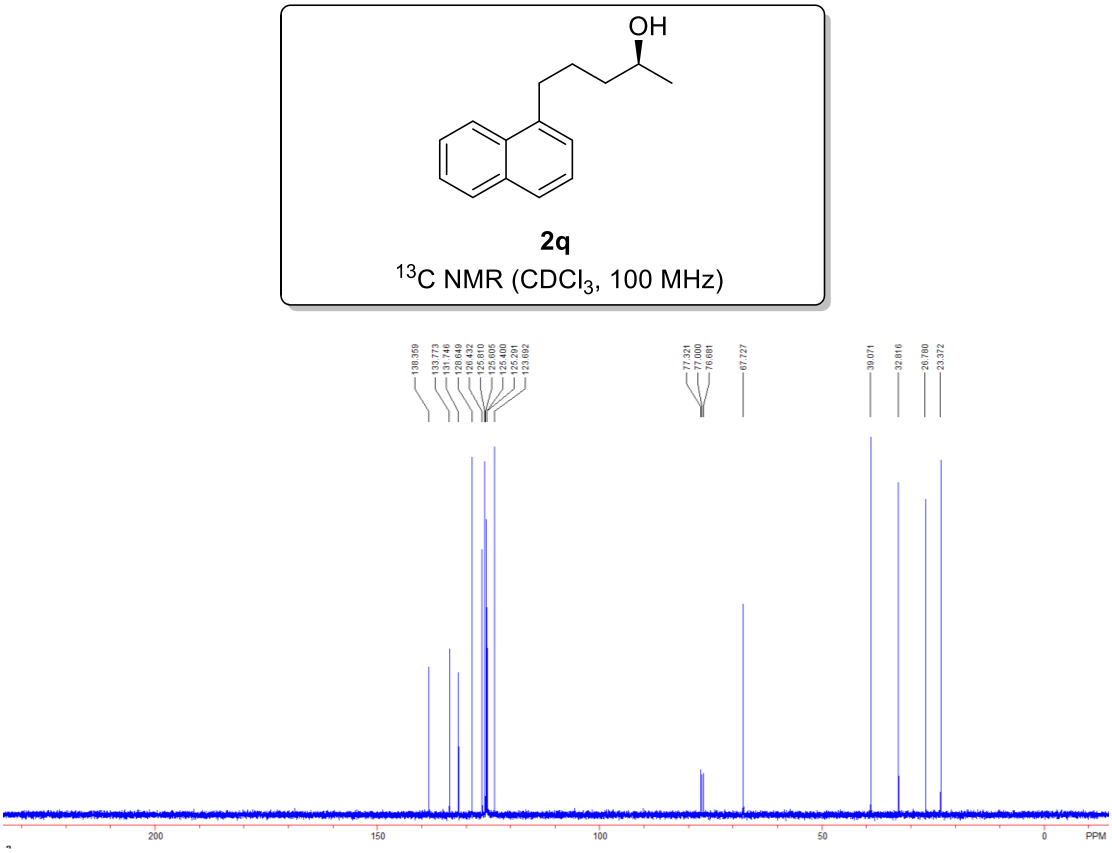

Supplementary Figure 36. ^{13}C NMR Spectrum of 2q

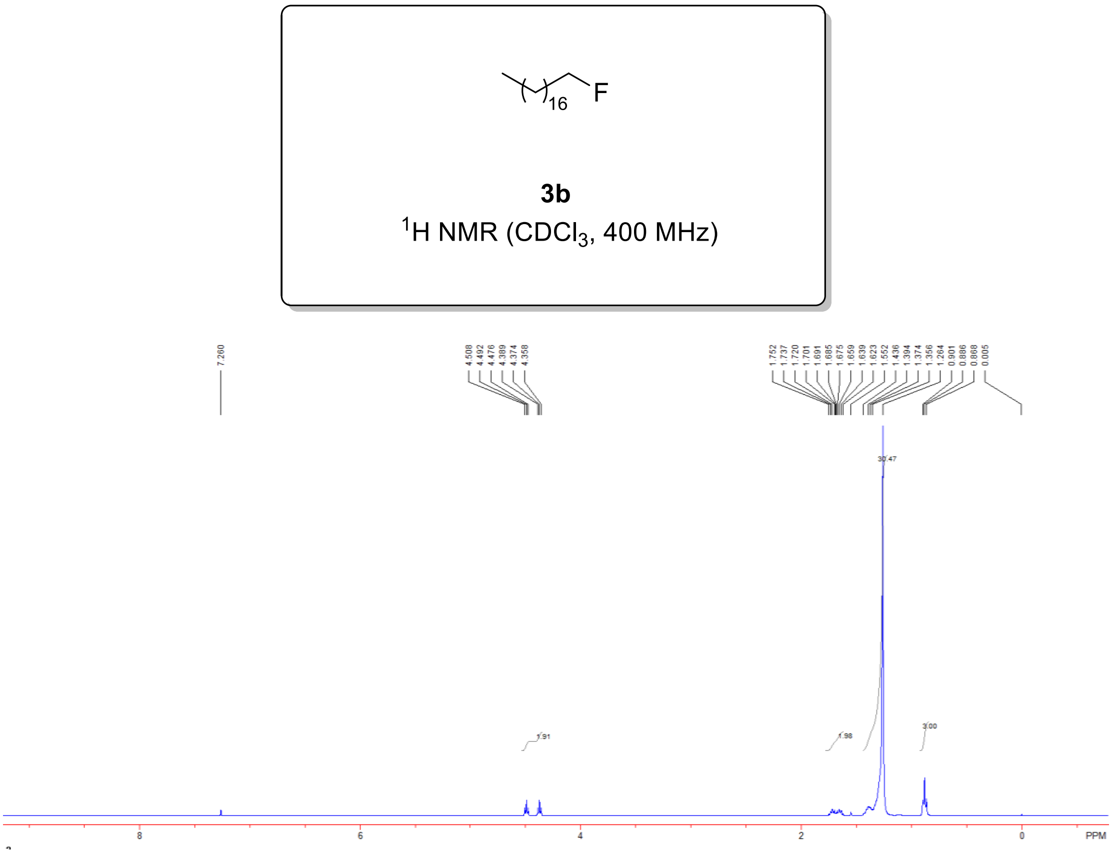

Supplementary Figure 37. ^{1}H NMR Spectrum of 3b

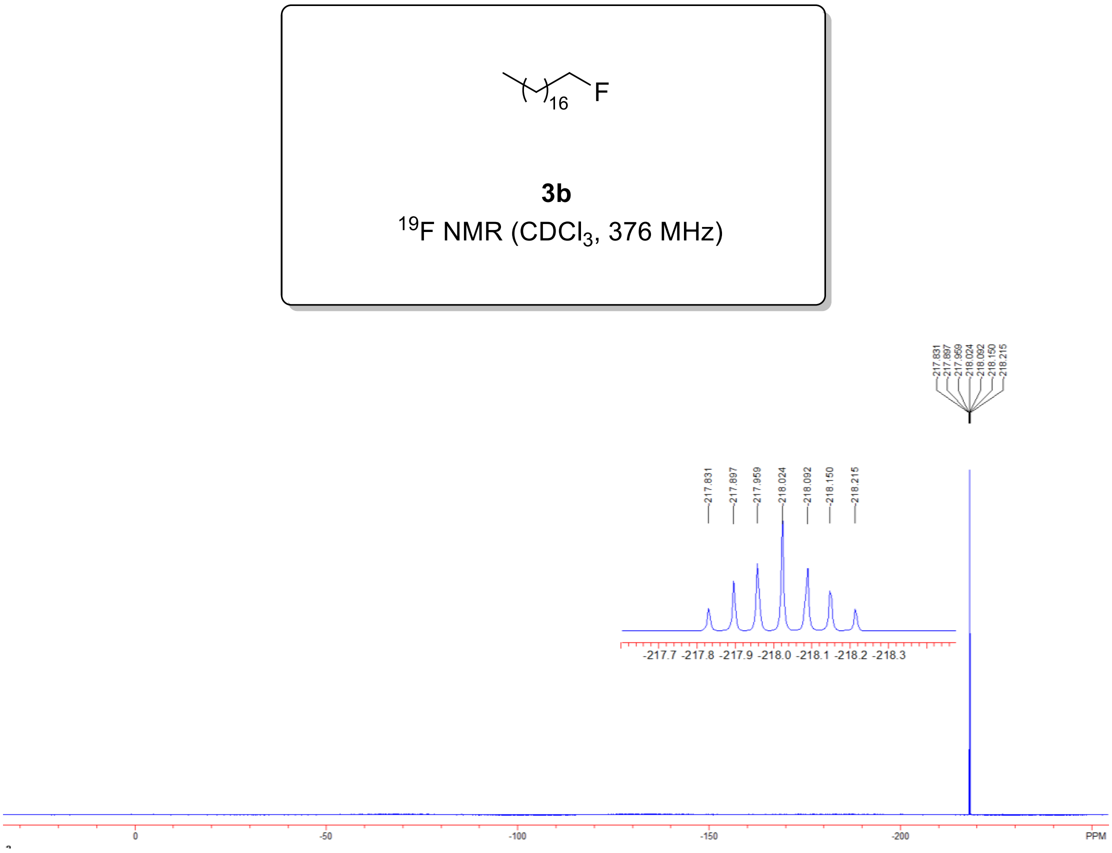

Supplementary Figure 38. ^{19}F NMR Spectrum of 3b

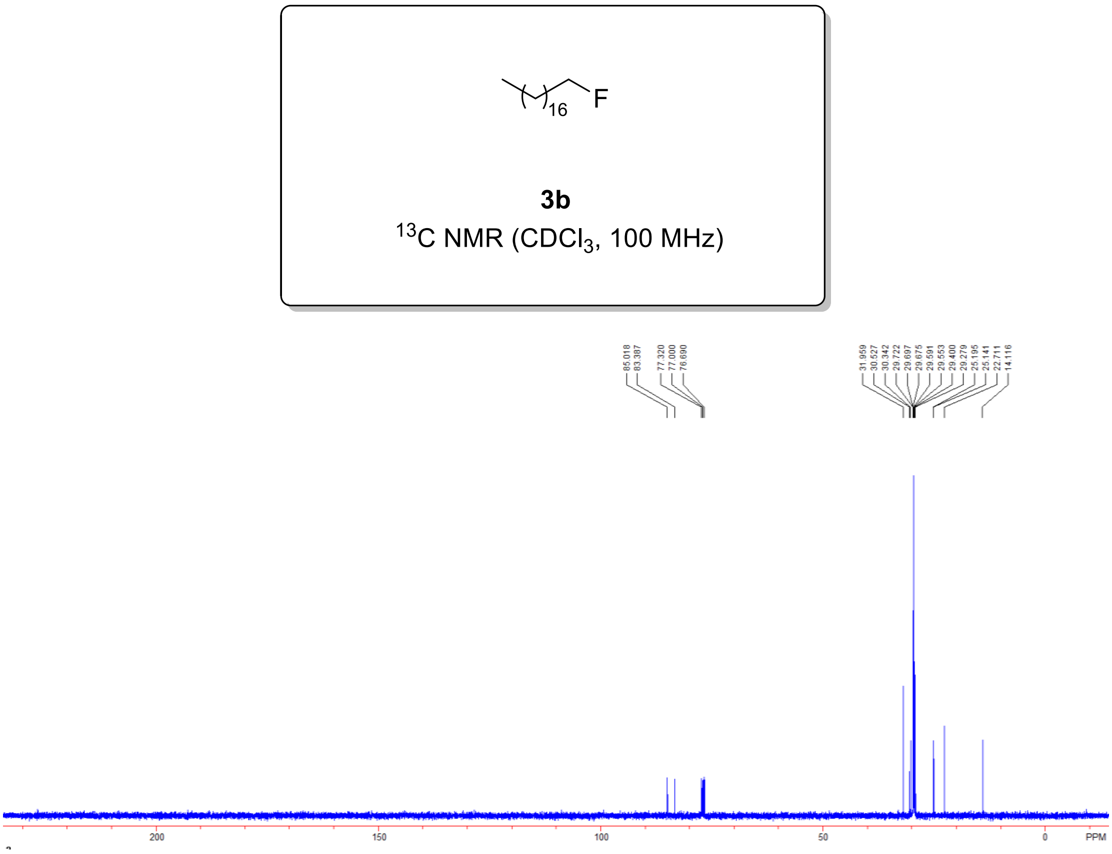

Supplementary Figure 39. ^{13}C NMR Spectrum of 3b

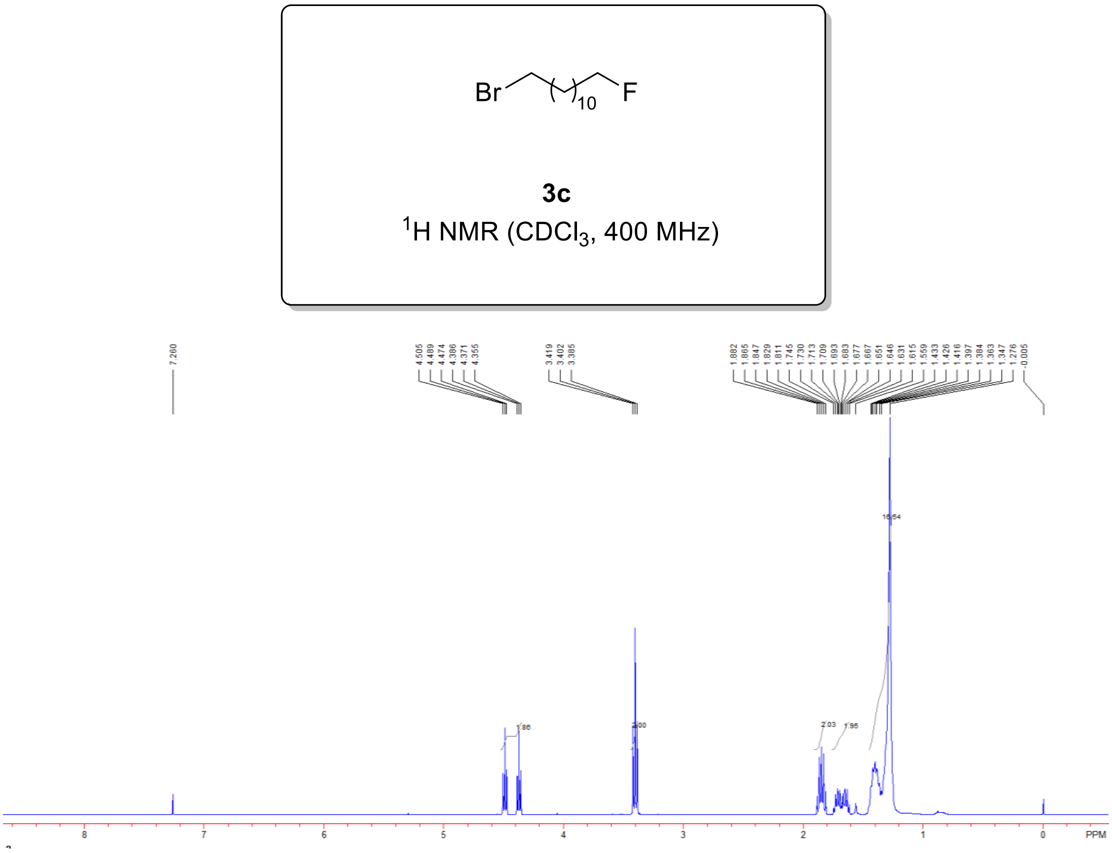

Supplementary Figure 40. ^{1}H NMR Spectrum of 3c

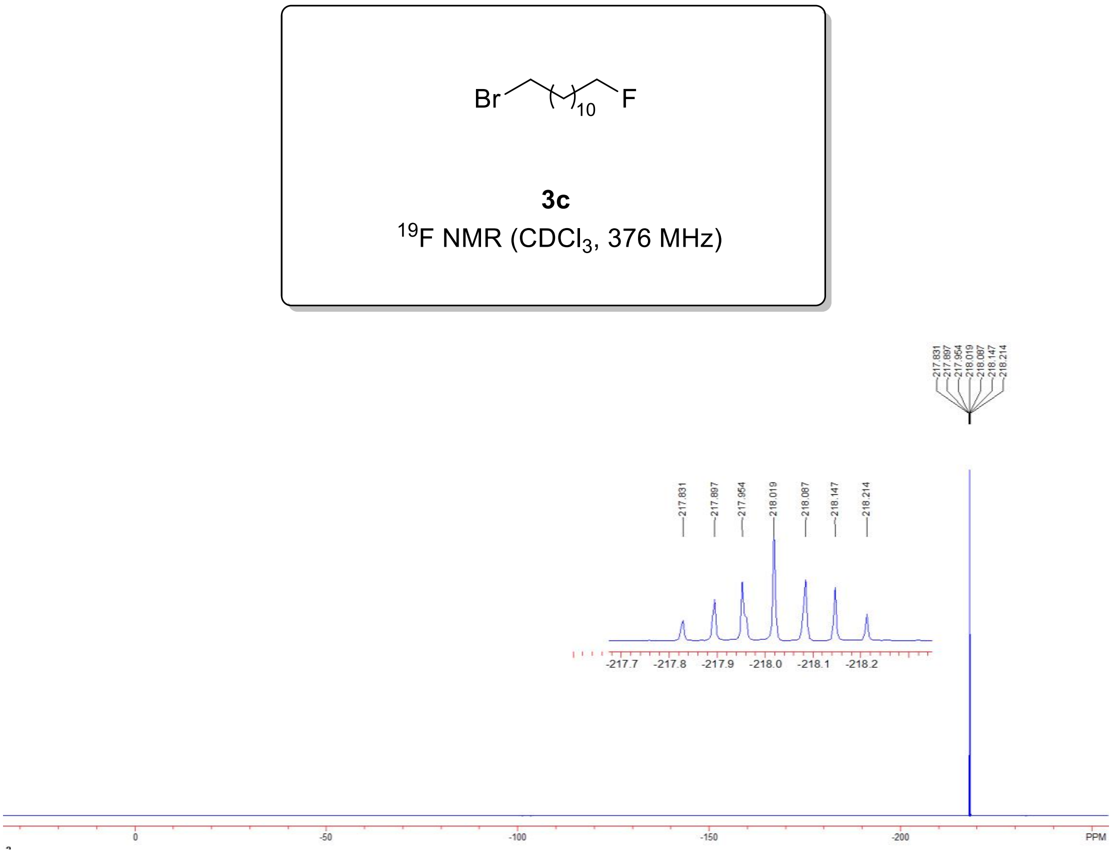

Supplementary Figure 41. ^{19}**F NMR Spectrum of 3c**

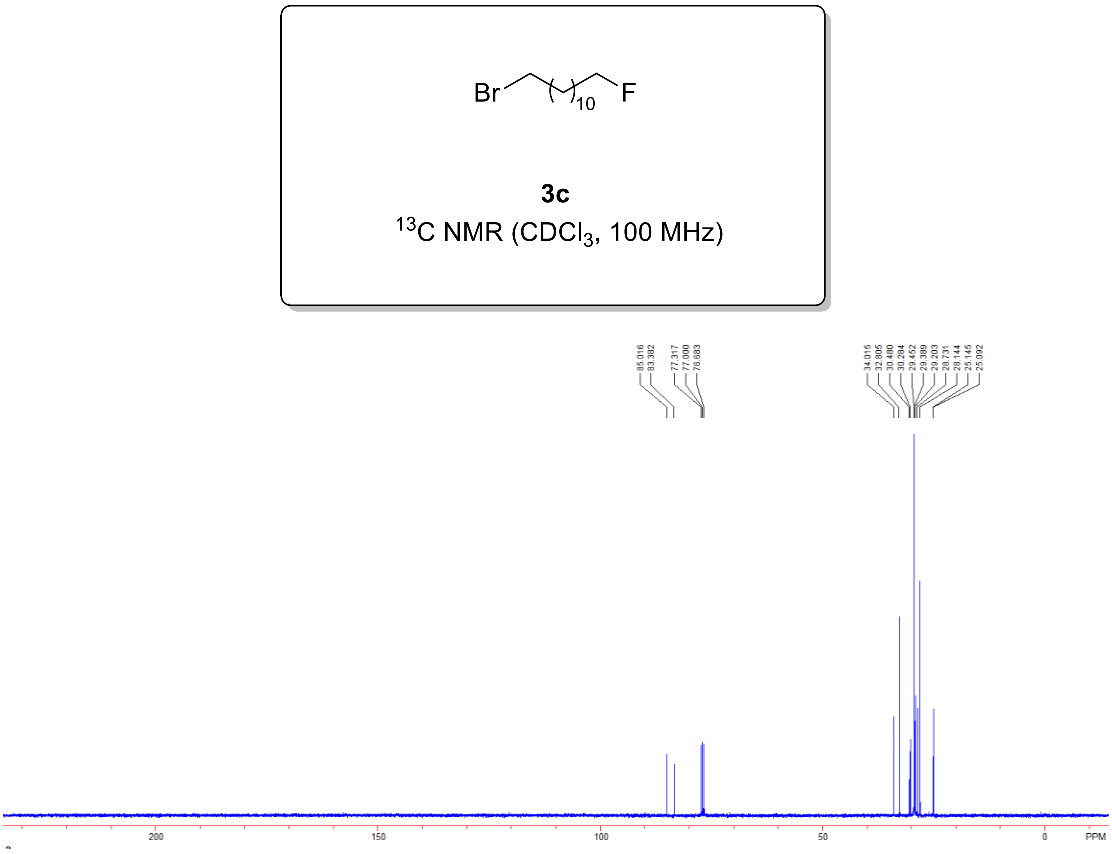

Supplementary Figure 42. ^{13}**C NMR Spectrum of 3c**

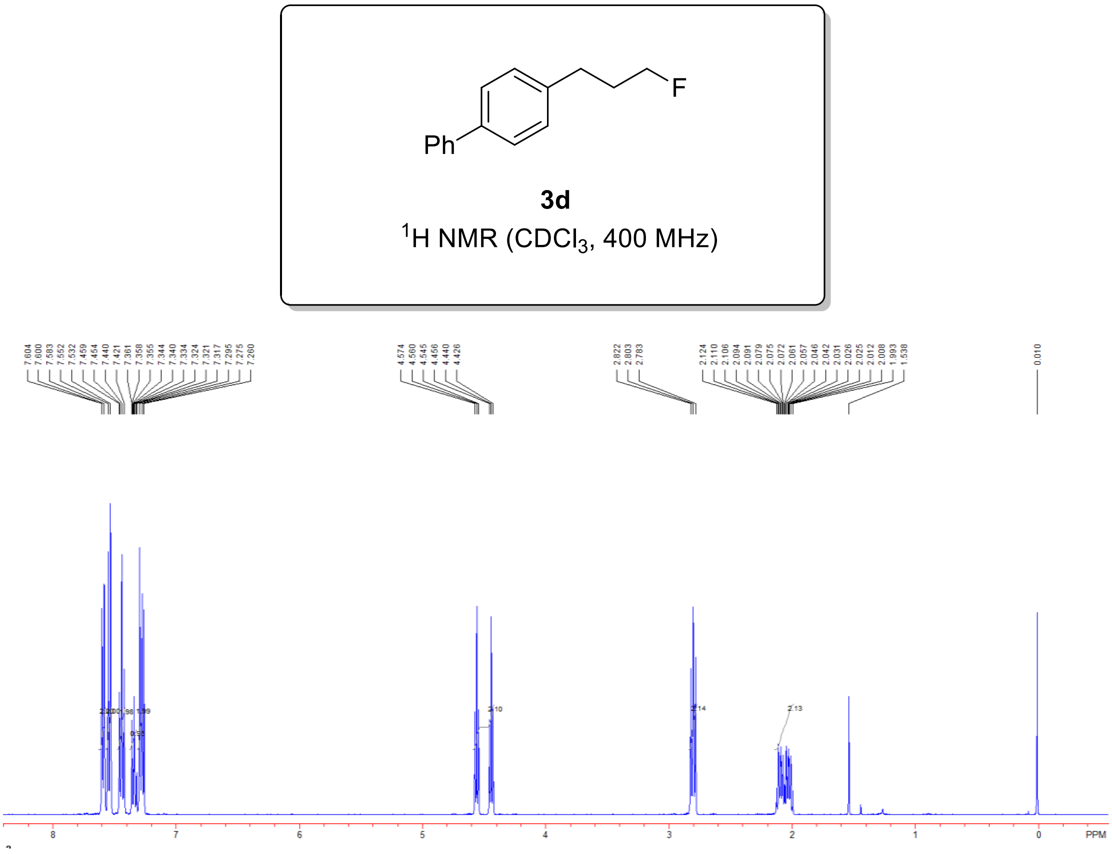

Supplementary Figure 43. ^{1}H NMR Spectrum of 3d

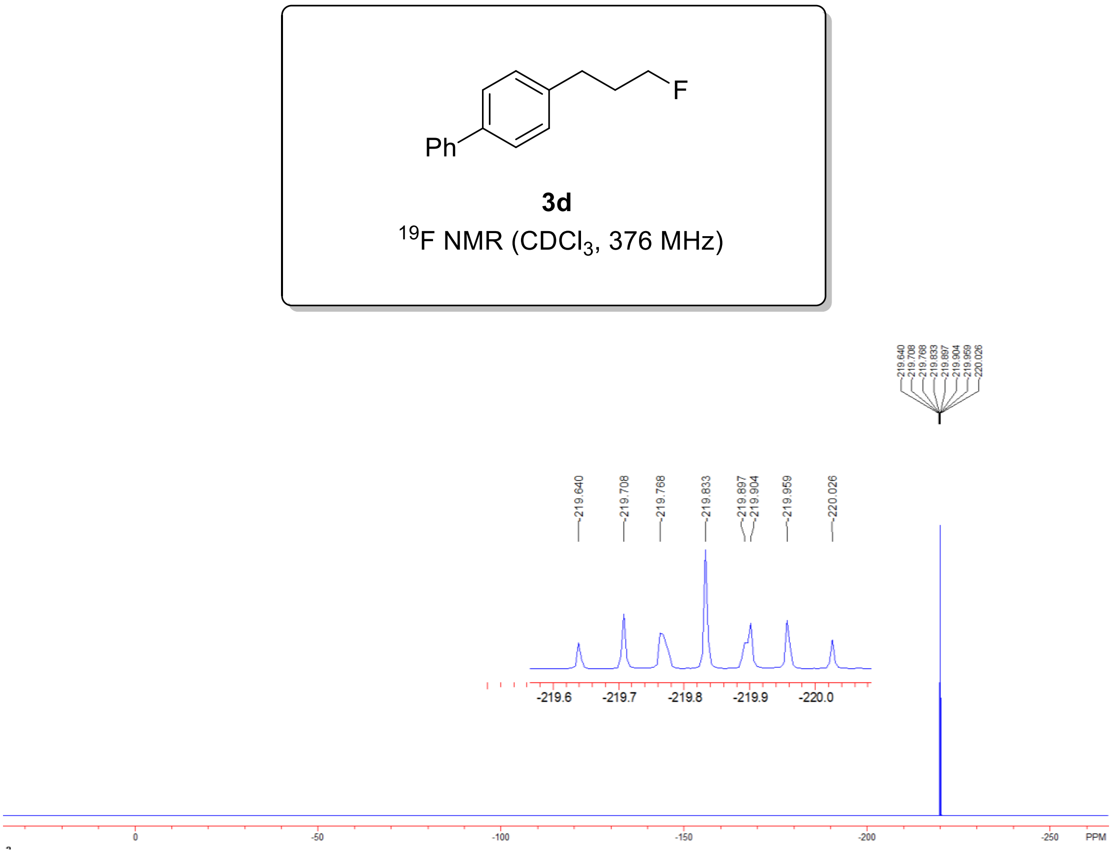

Supplementary Figure 44. ^{19}F NMR Spectrum of 3d

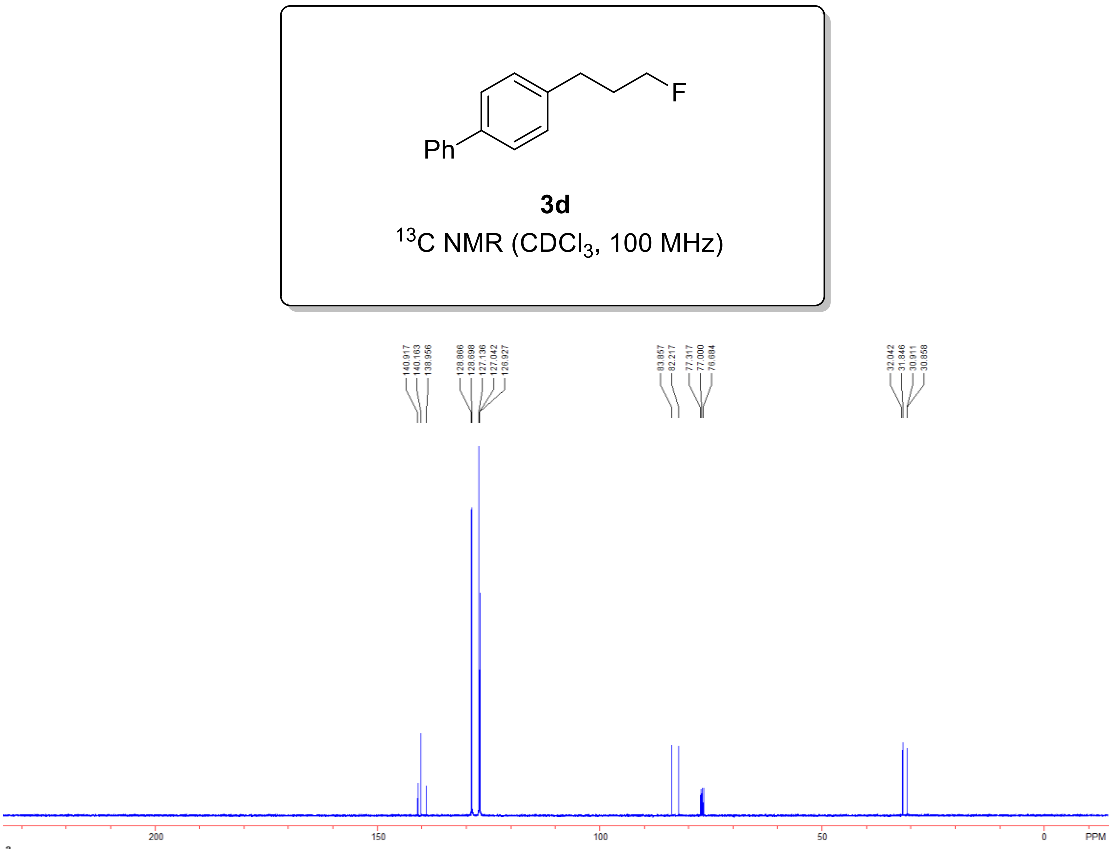

Supplementary Figure 45. ^{13}C NMR Spectrum of 3d

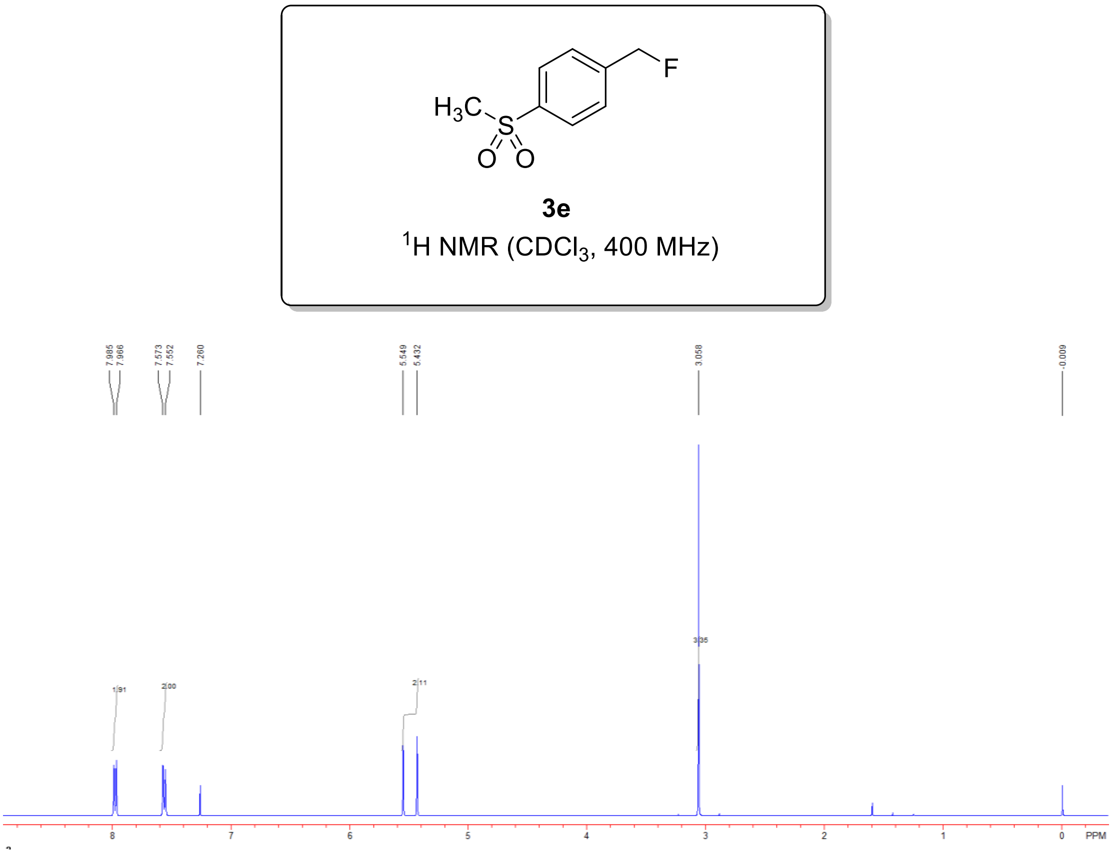

Supplementary Figure 46. ^{1}H NMR Spectrum of 3e

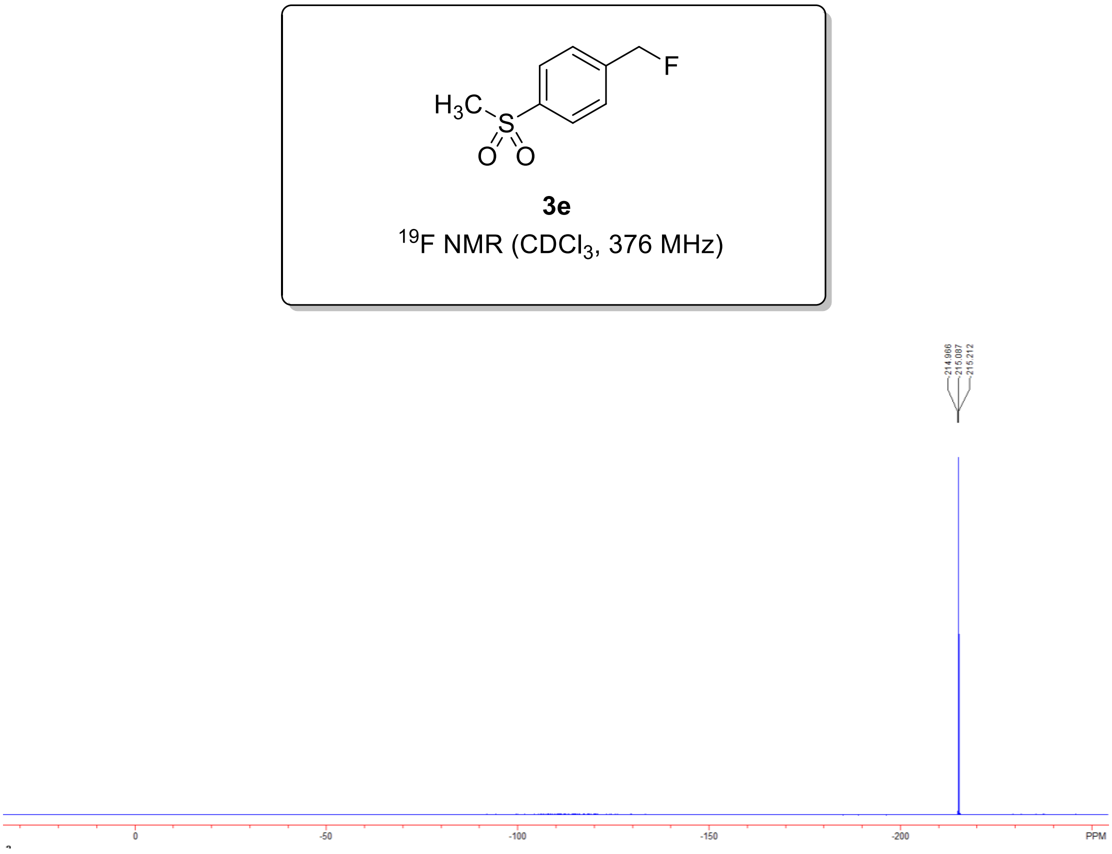

Supplementary Figure 47. ^{19}F NMR Spectrum of 3e

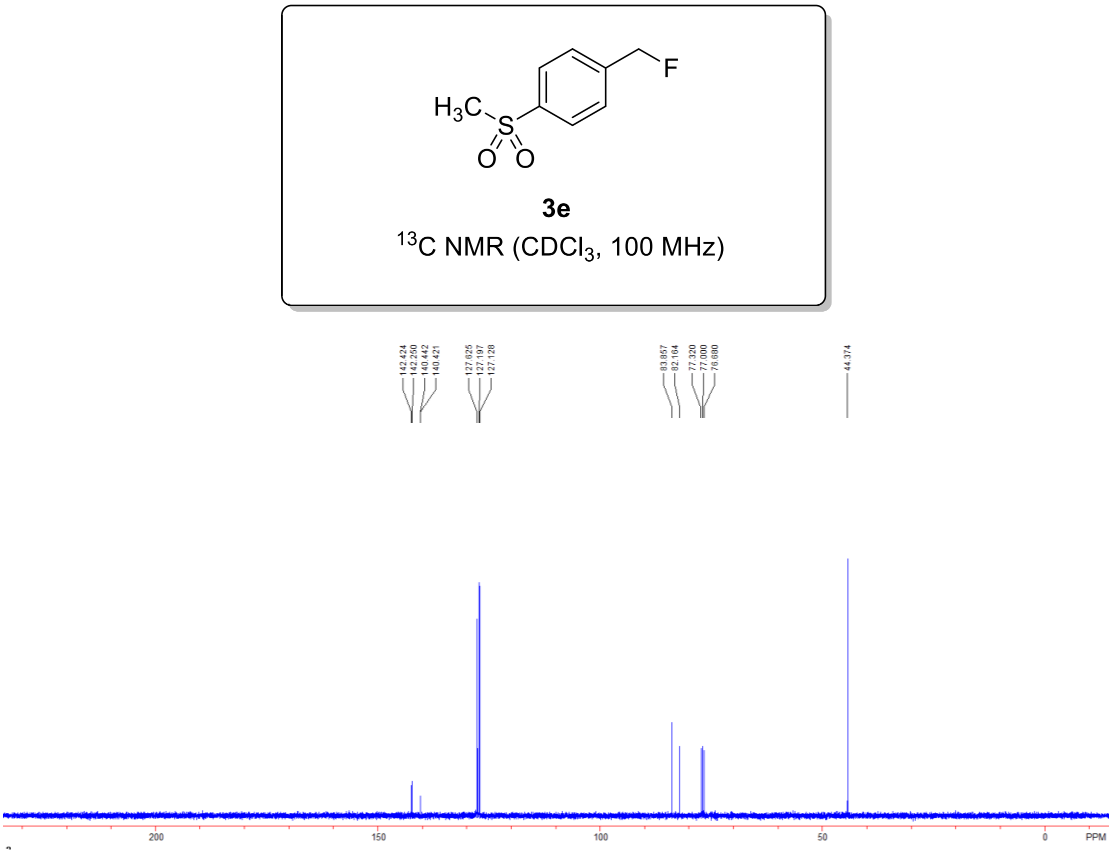

Supplementary Figure 48. ^{13}C NMR Spectrum of 3e

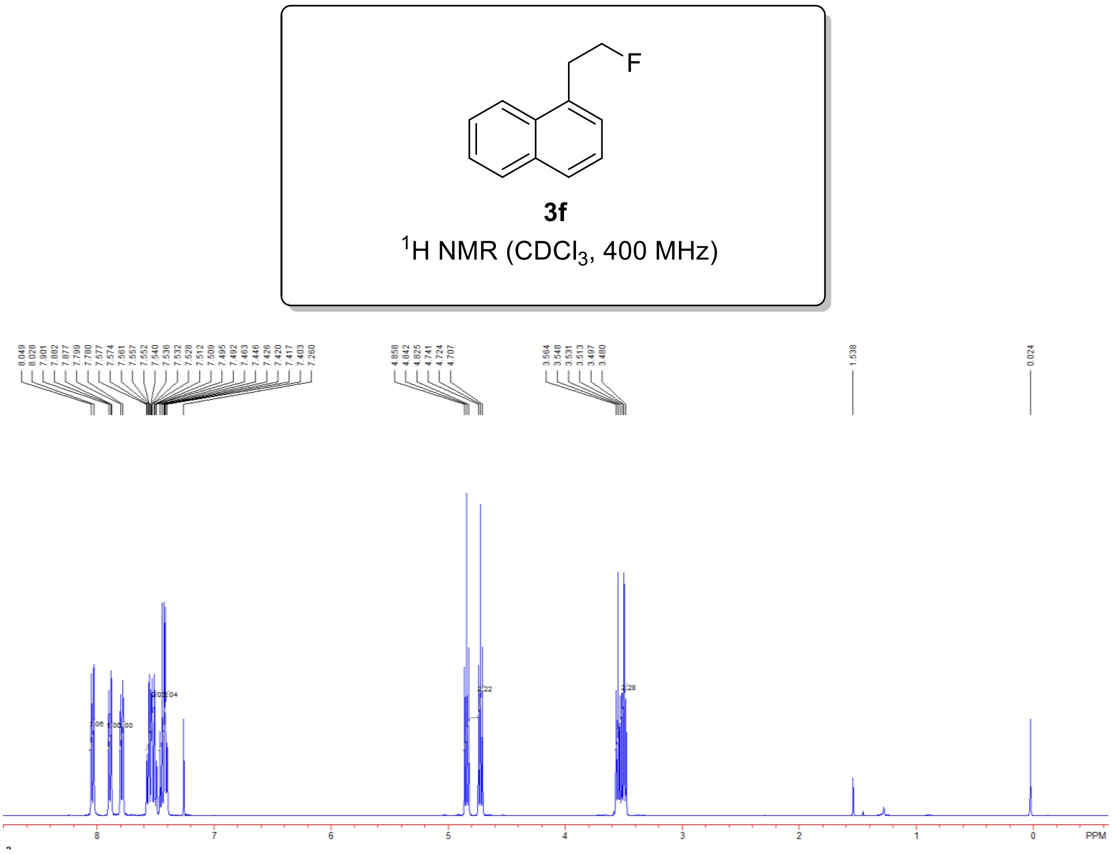

Supplementary Figure 49. ^{1}H NMR Spectrum of 3f

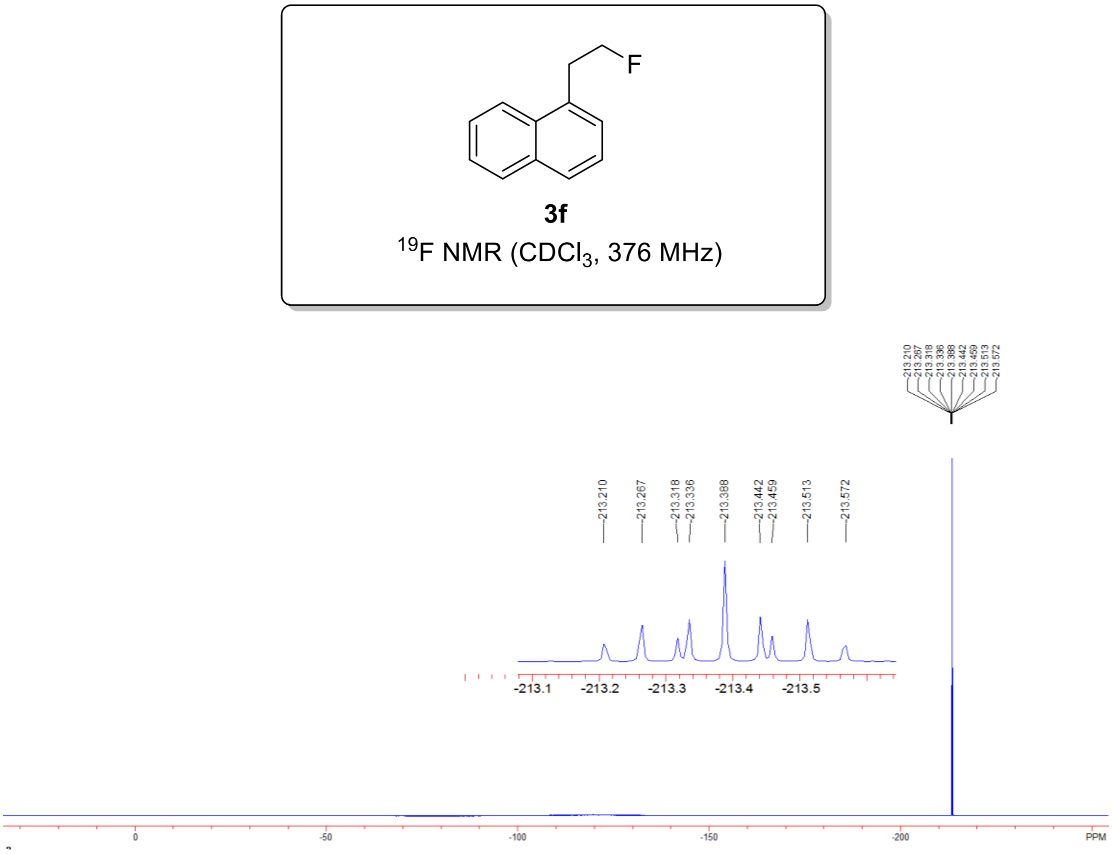

Supplementary Figure 50. ^{19}F NMR Spectrum of 3f

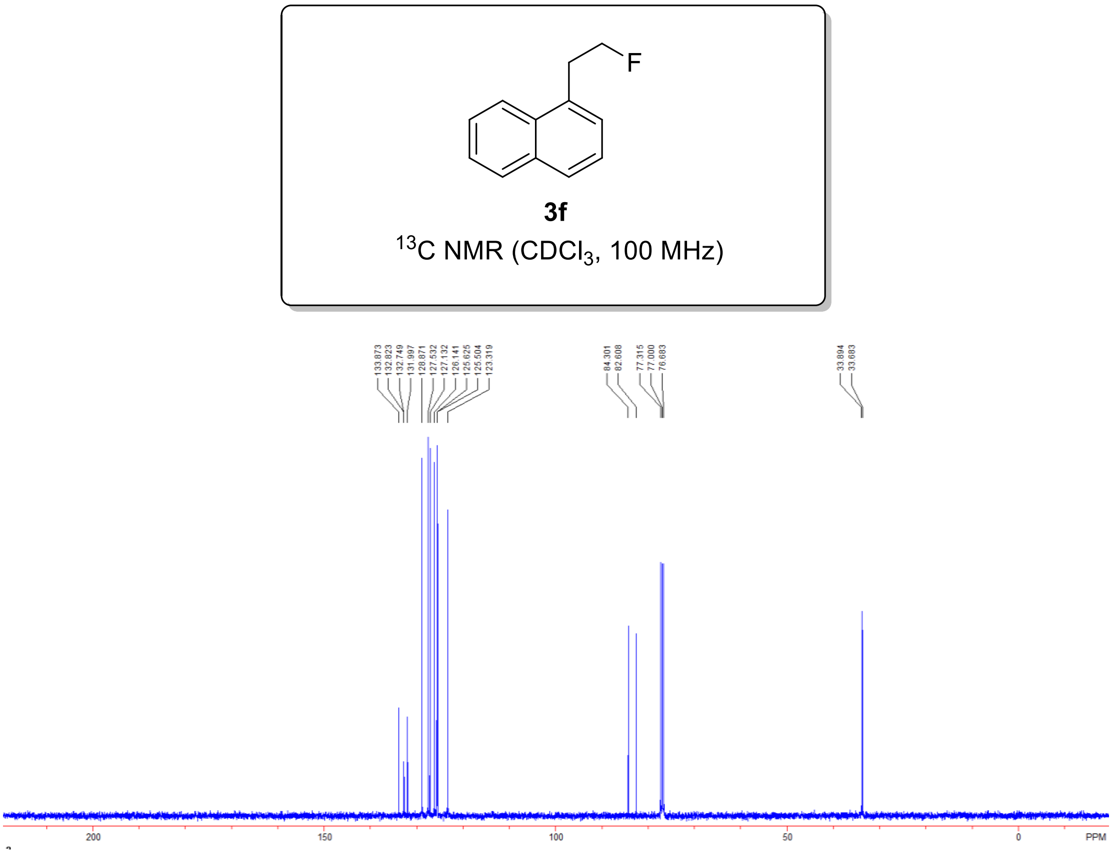

Supplementary Figure 51. ^{13}C NMR Spectrum of 3f

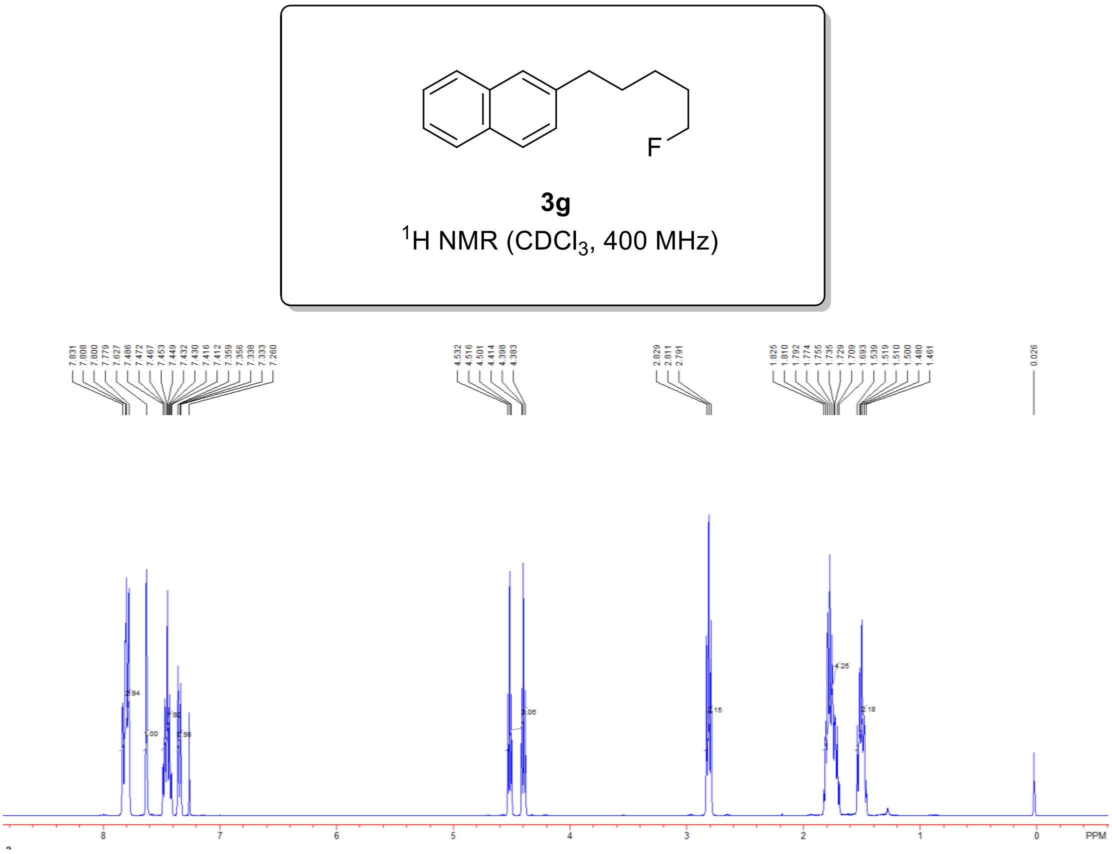

Supplementary Figure 52. ^{1}H NMR Spectrum of 3g

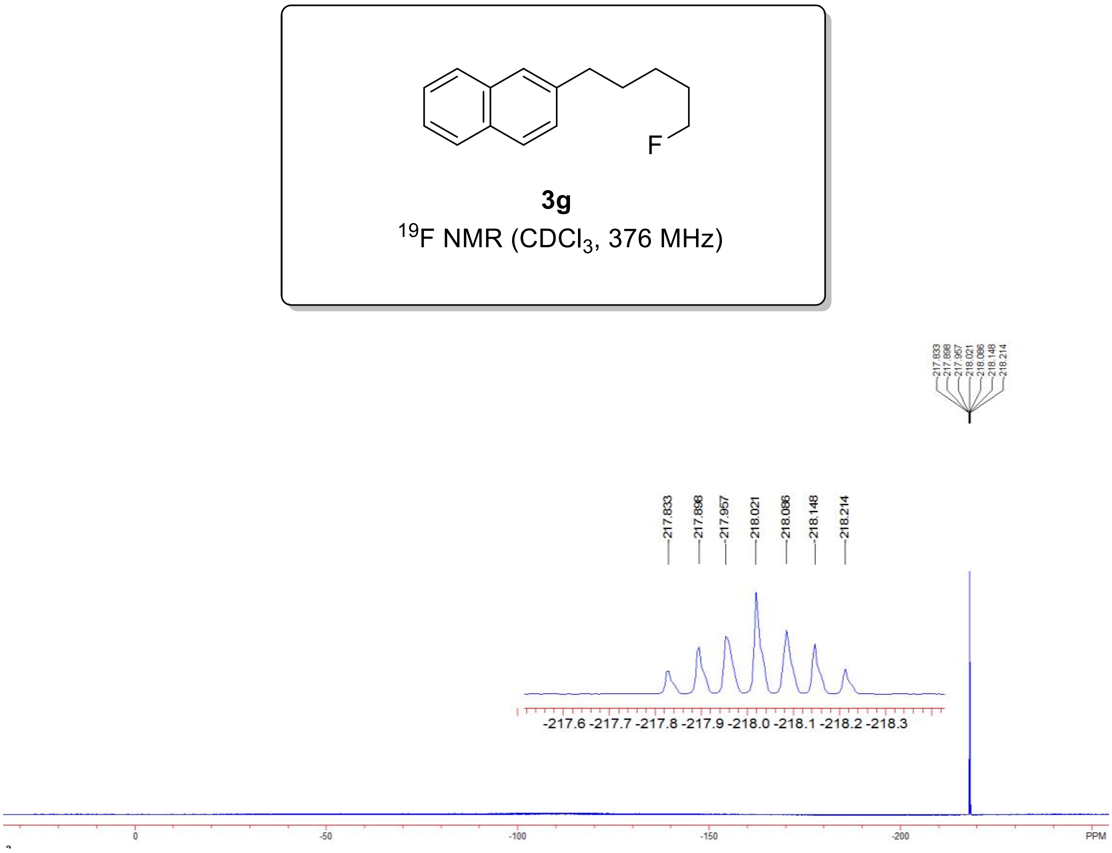

Supplementary Figure 53. ^{19}F NMR Spectrum of 3g

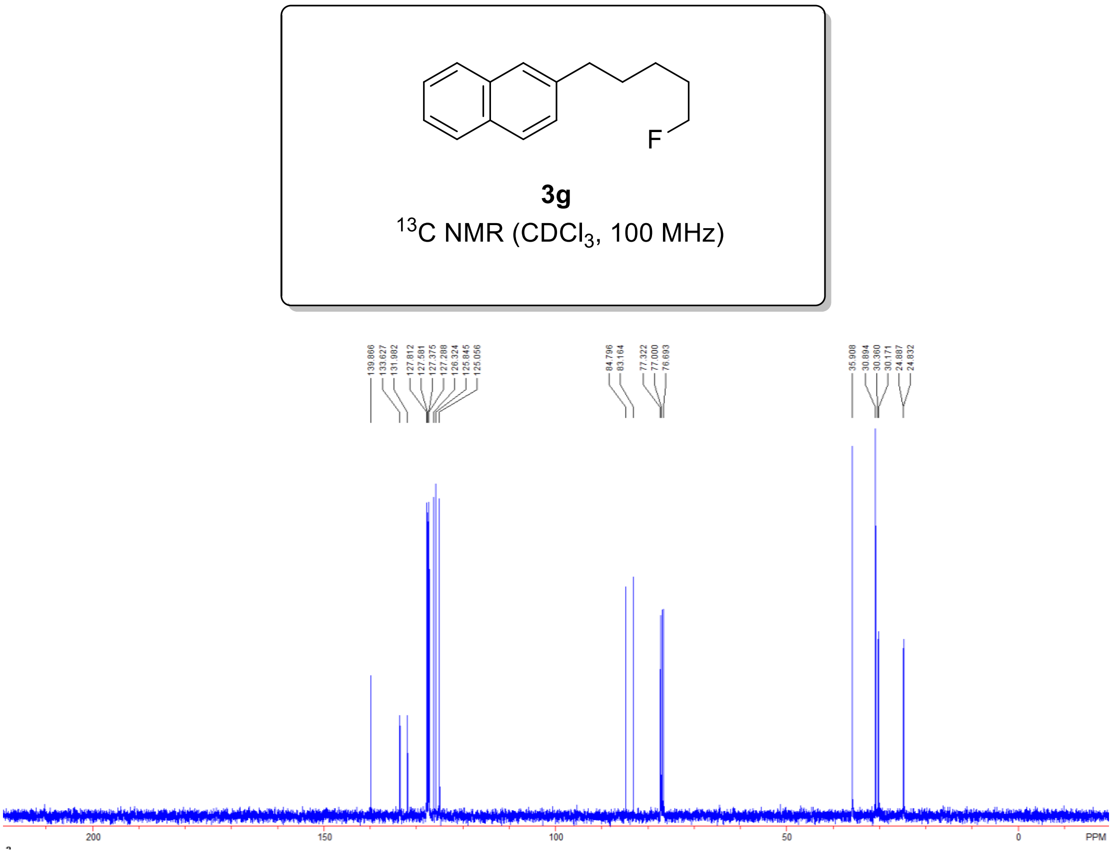

Supplementary Figure 54. ^{13}C NMR Spectrum of 3g

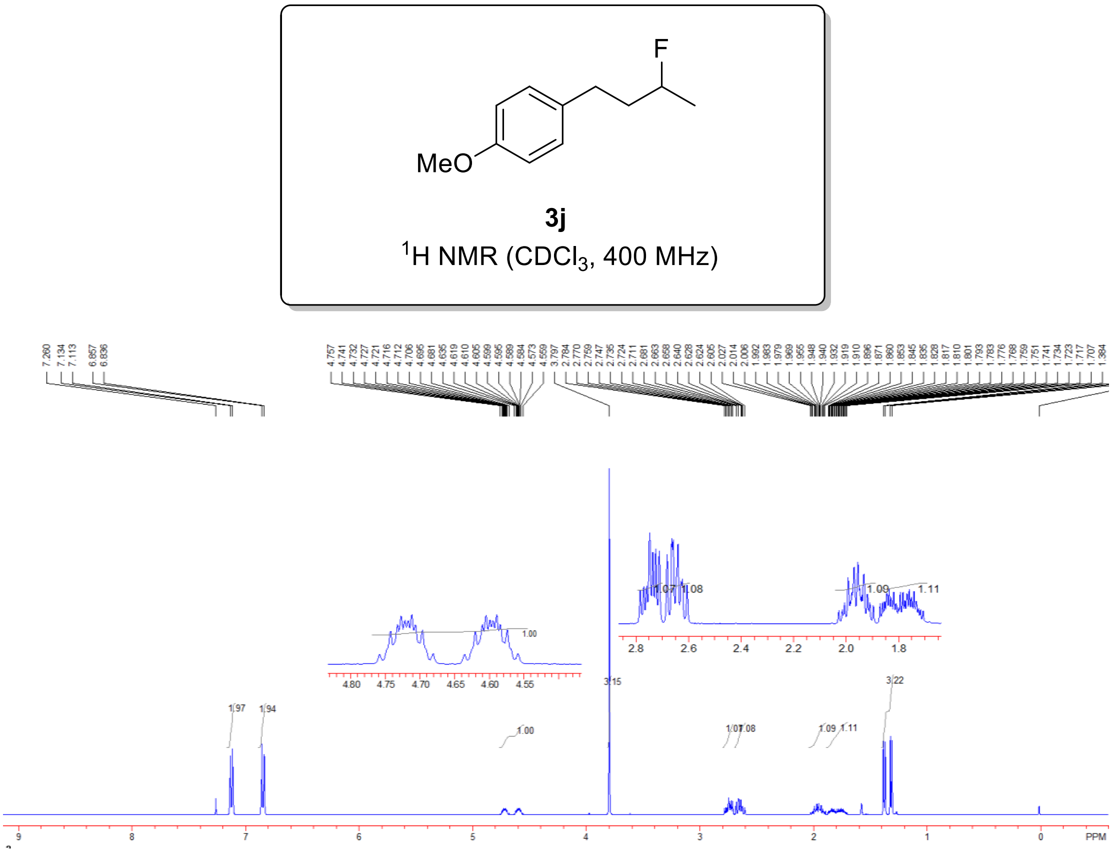

Supplementary Figure 55. ^{1}**H NMR Spectrum of 3j**

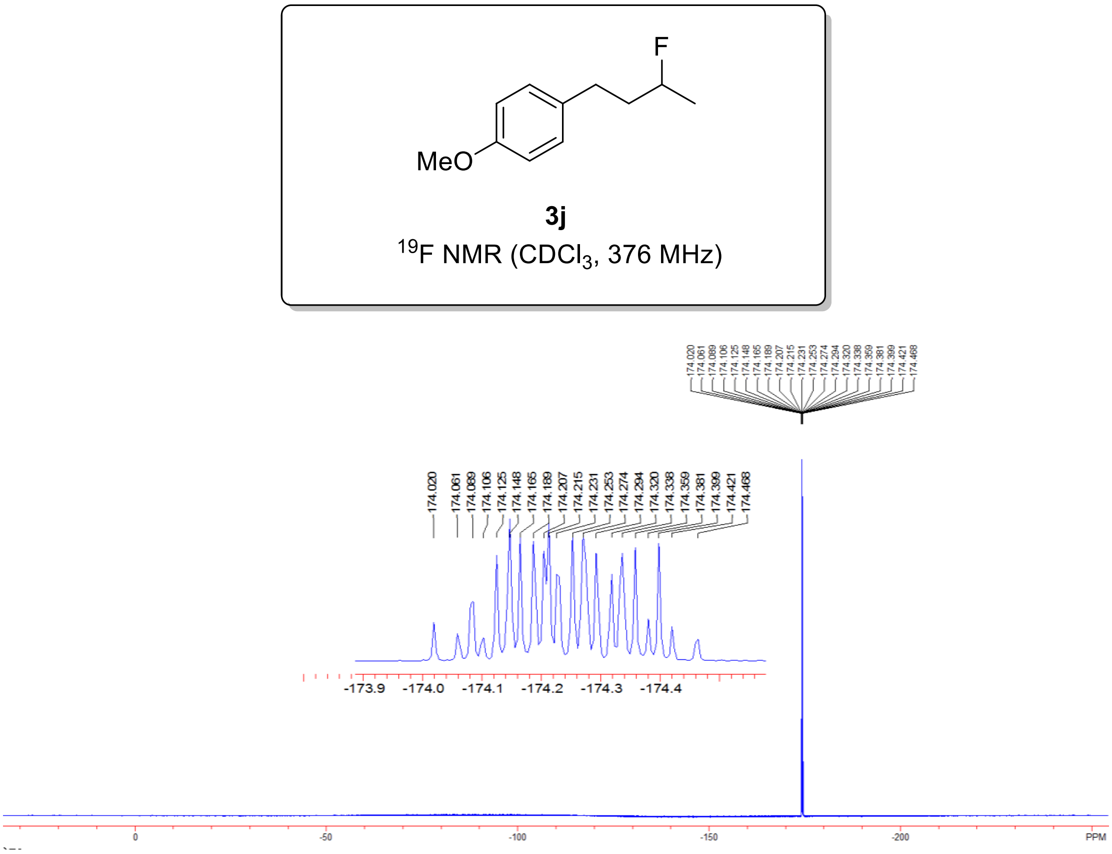

Supplementary Figure 56. ^{19}**F NMR Spectrum of 3j**

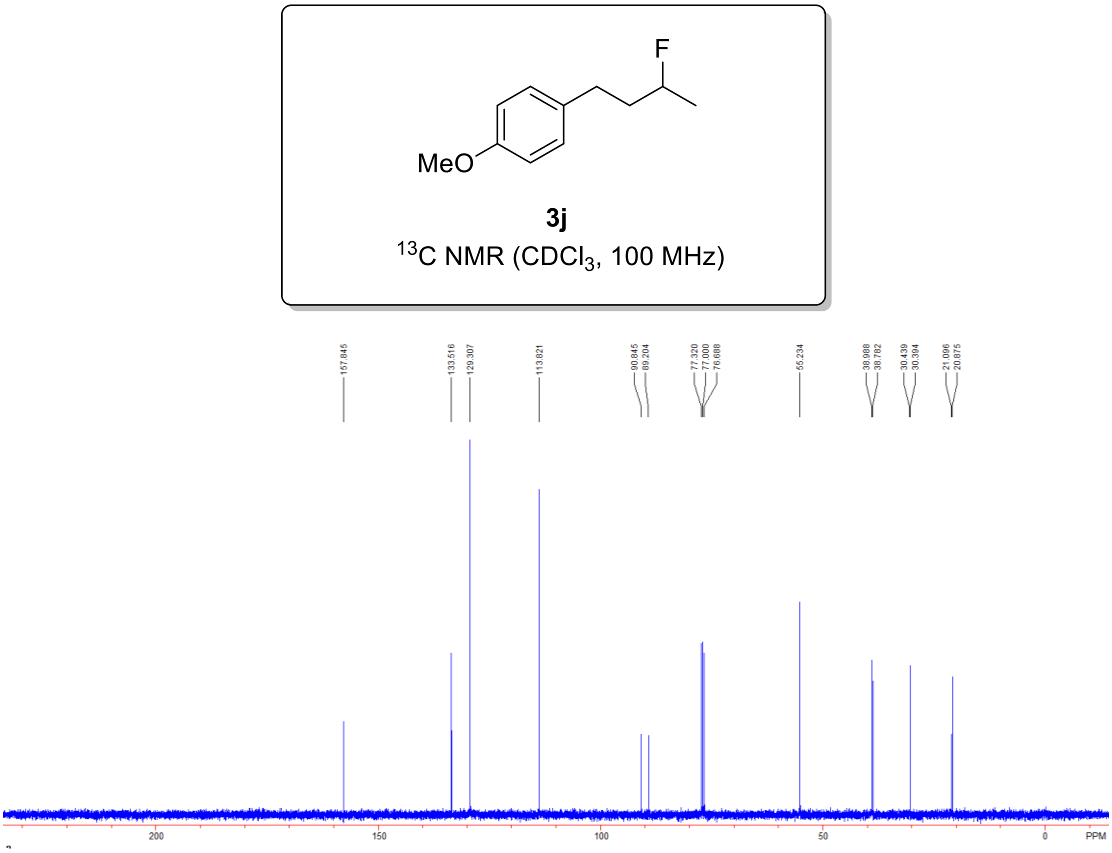

Supplementary Figure 57. ^{13}C NMR Spectrum of 3j

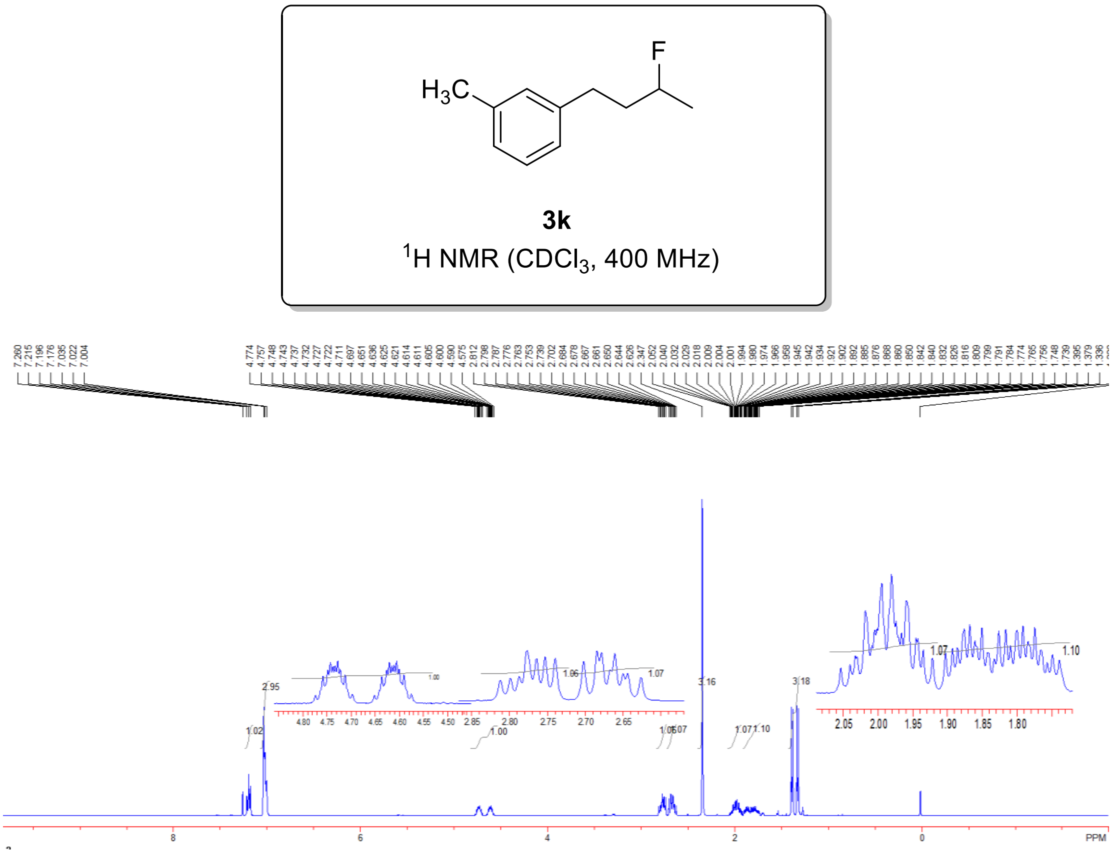

Supplementary Figure 58. ^{1}H NMR Spectrum of 3k

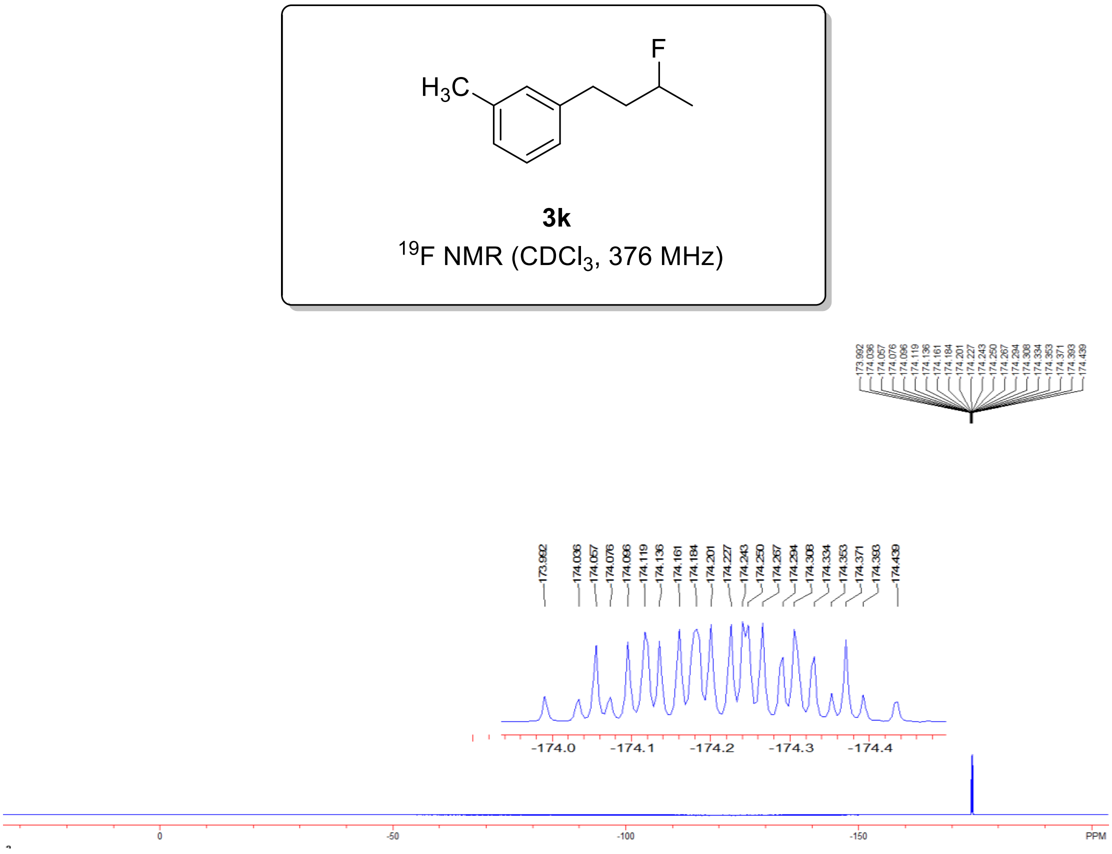

Supplementary Figure 59. ^{19}F NMR Spectrum of 3k

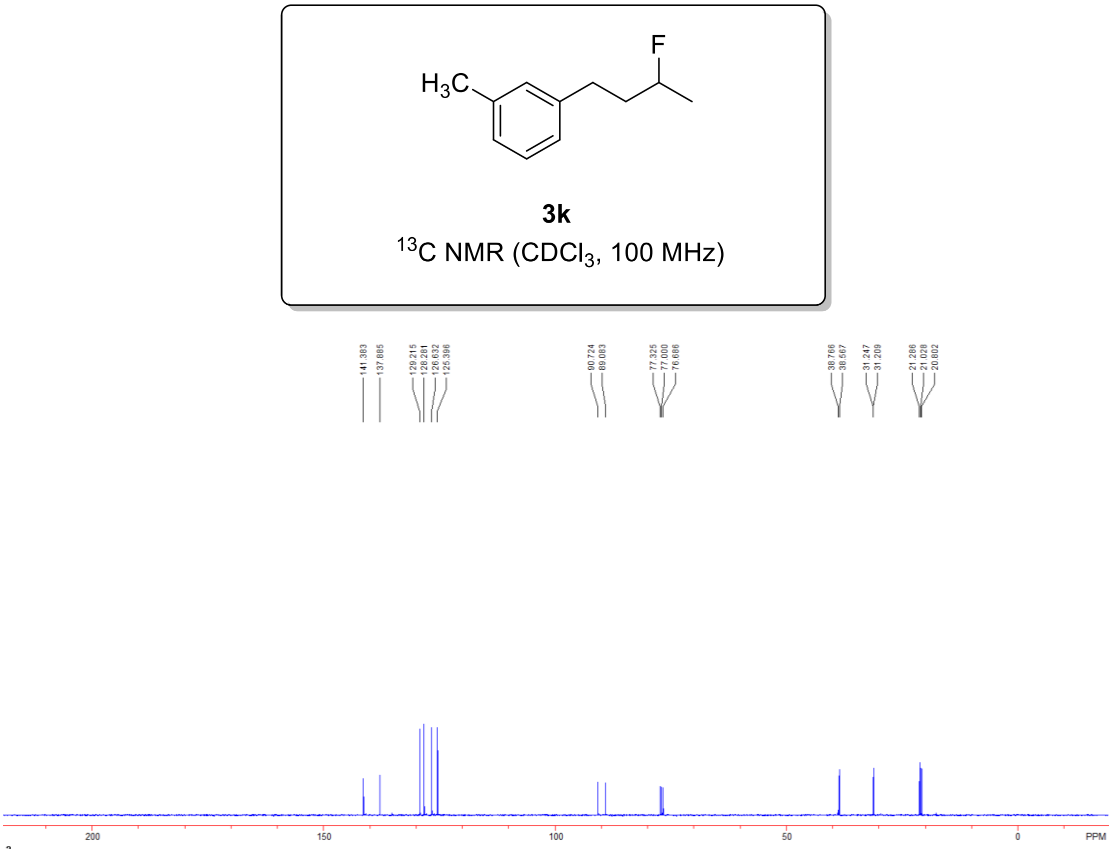

Supplementary Figure 60. ^{13}C NMR Spectrum of 3k

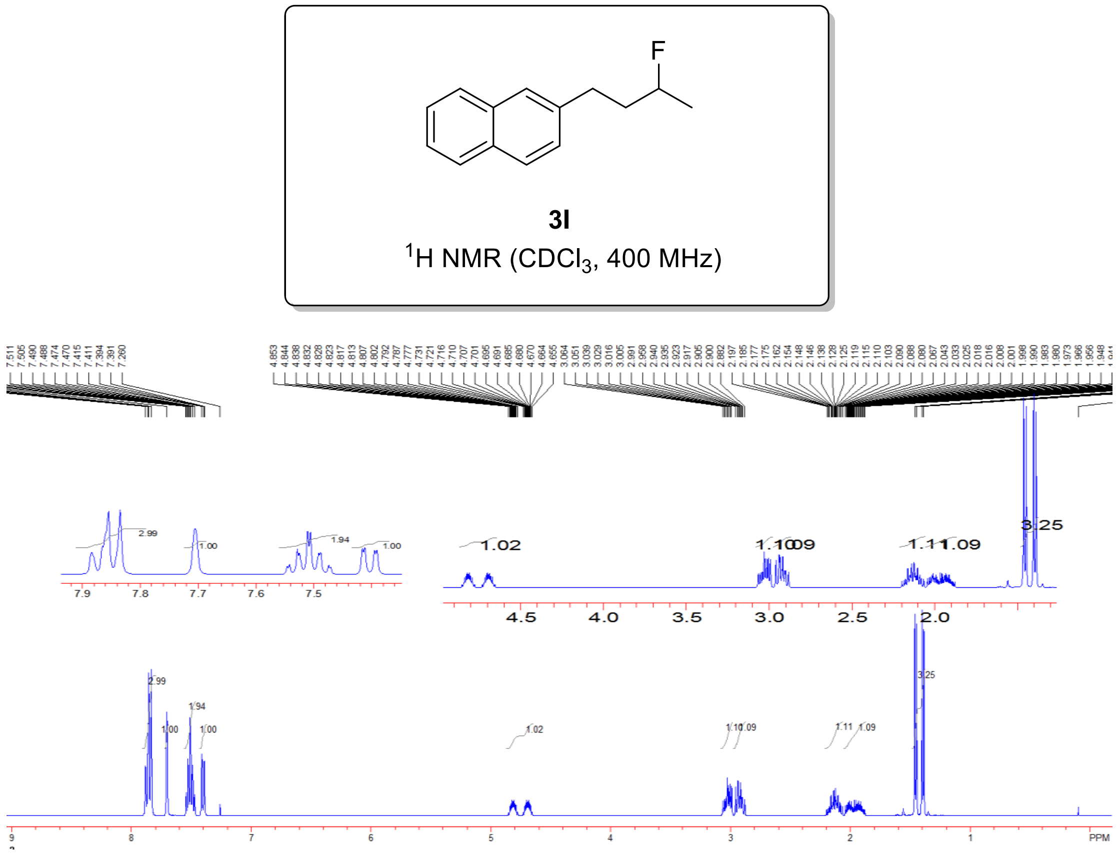

Supplementary Figure 61. ^{1}H NMR Spectrum of 3l

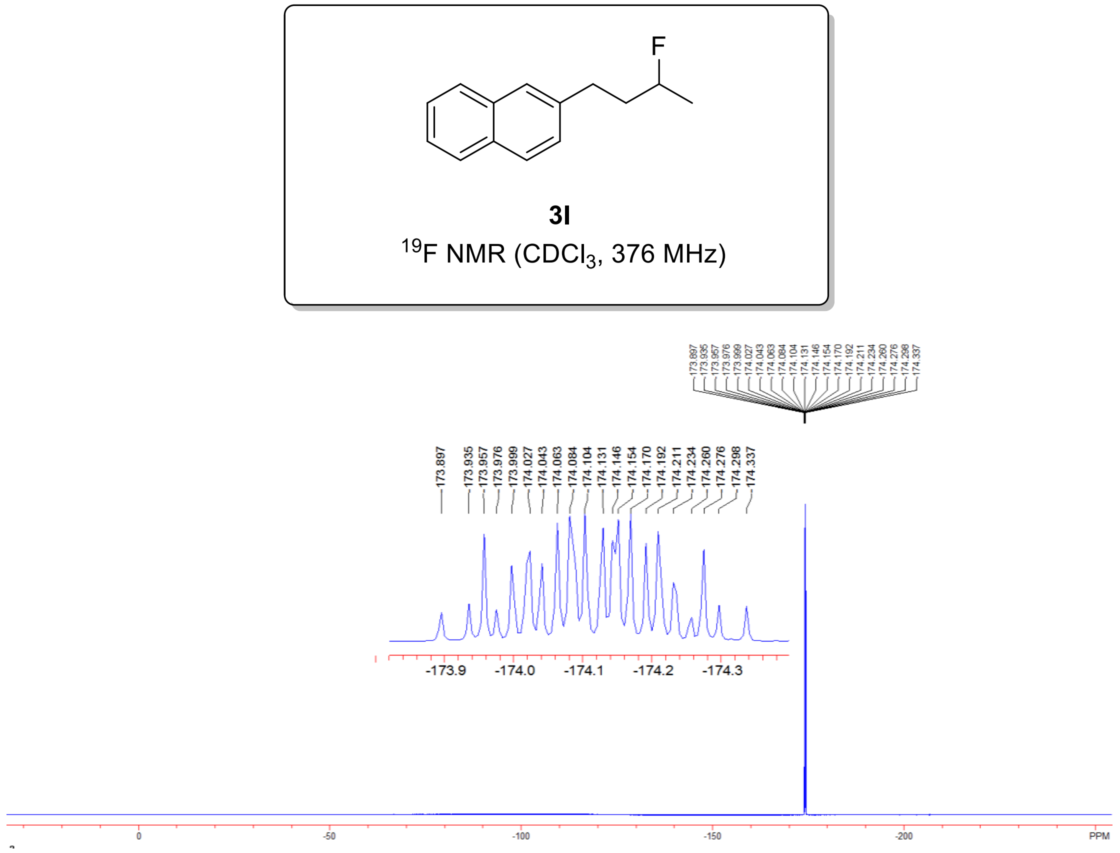

Supplementary Figure 62. ^{19}F NMR Spectrum of 3l

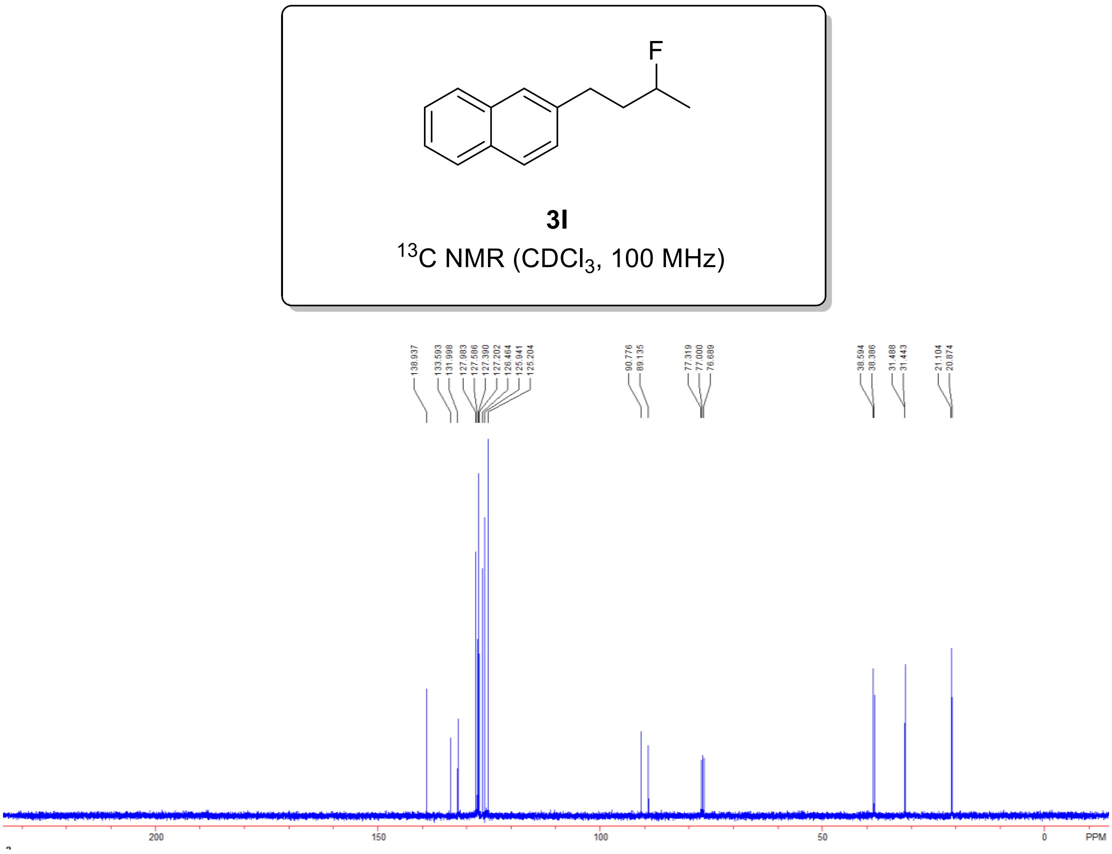

Supplementary Figure 63. ^{13}**C NMR Spectrum of 3l**

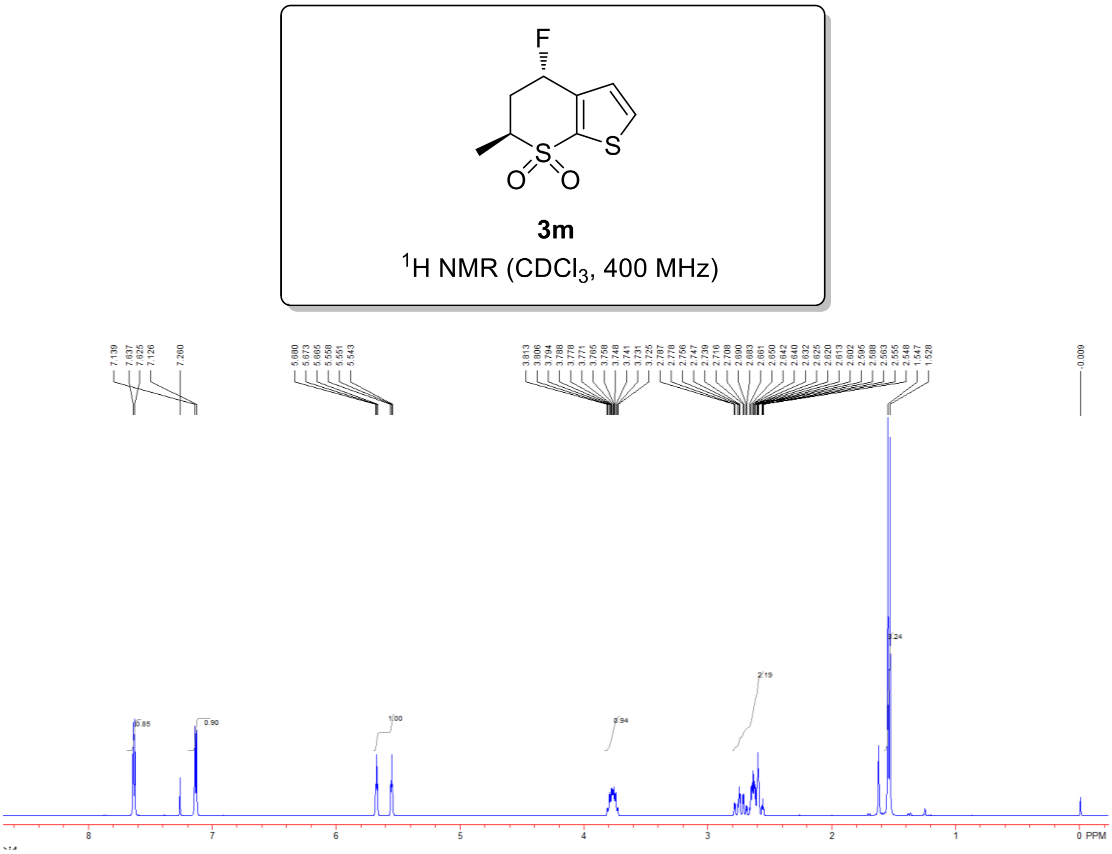

Supplementary Figure 64. ^{1}**H NMR Spectrum of 3m**

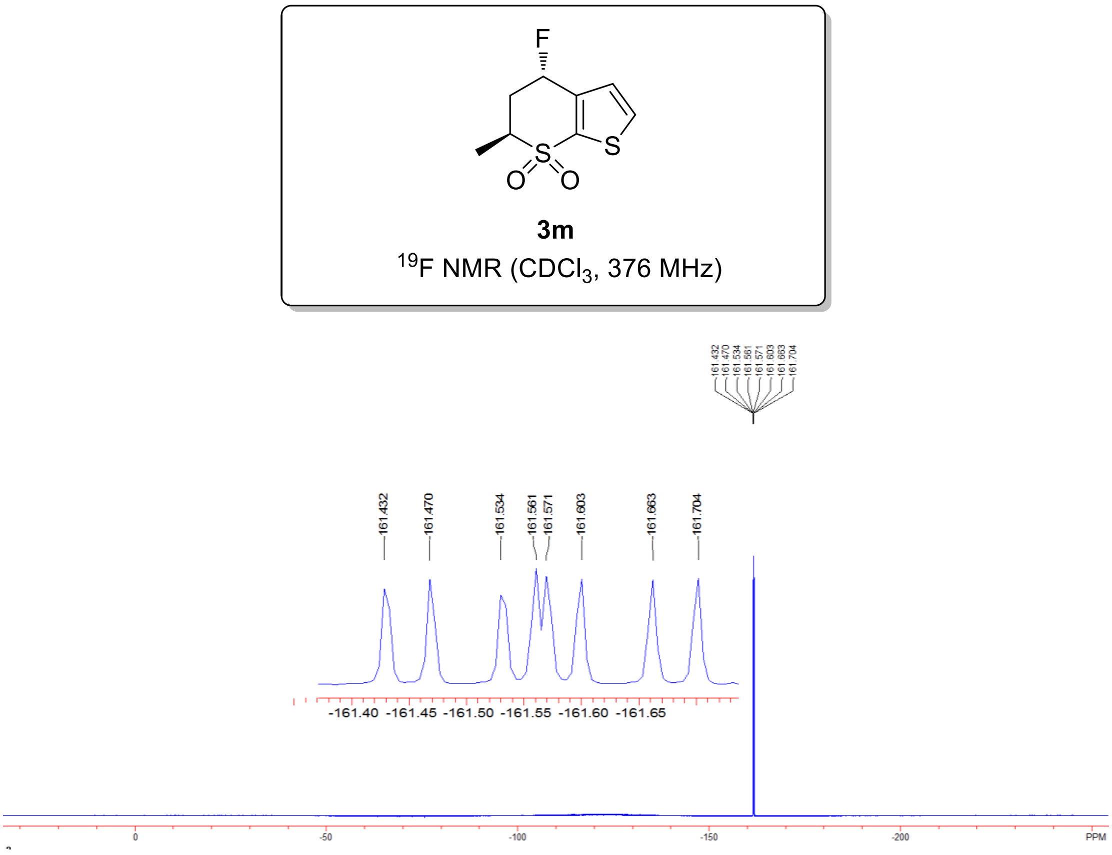

Supplementary Figure 65. ^{19}F NMR Spectrum of 3m

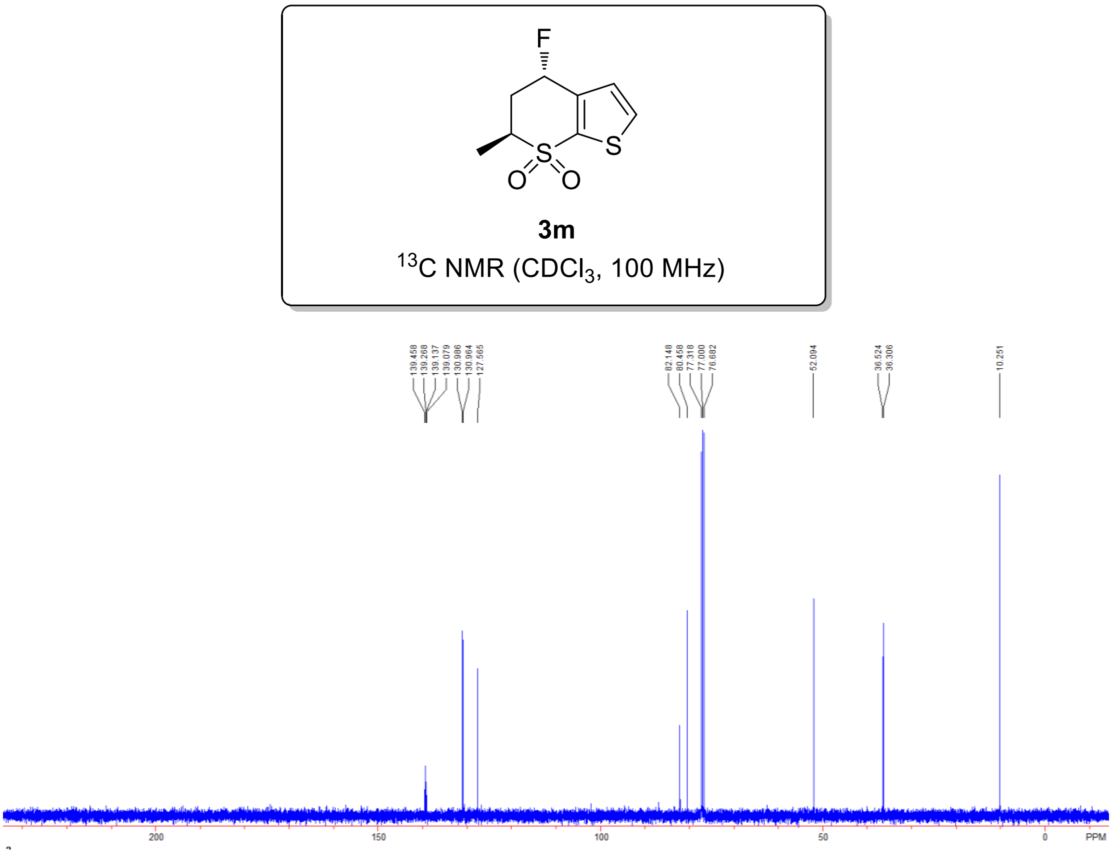

Supplementary Figure 66. ^{13}C NMR Spectrum of 3m

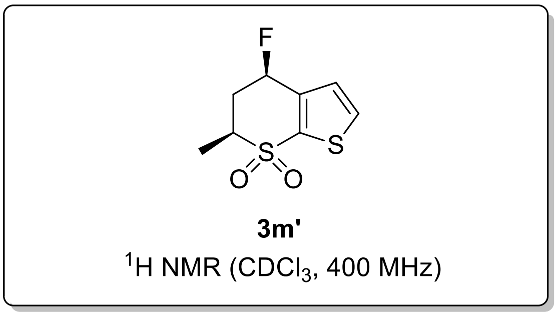

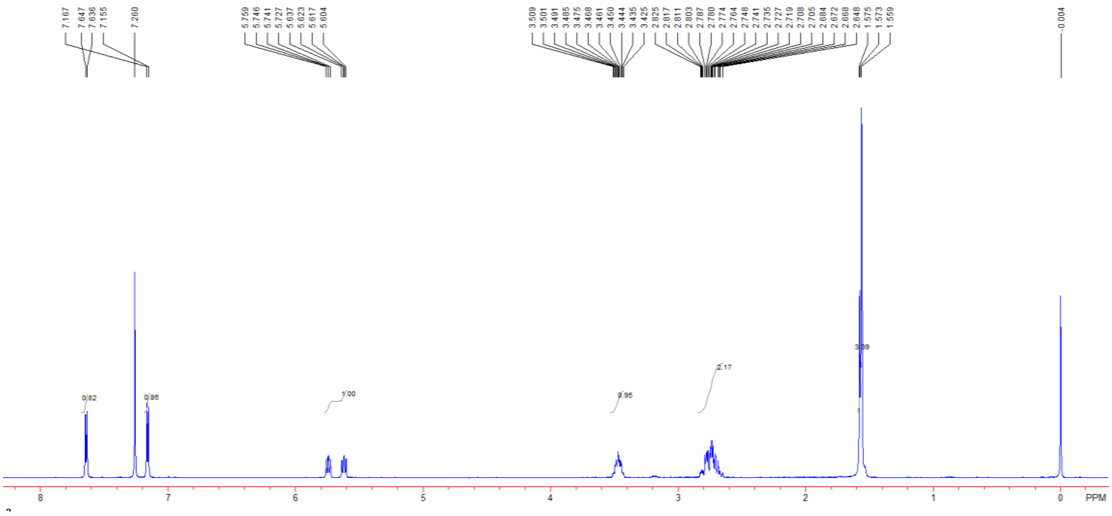

Supplementary Figure 67. ^{1}H NMR Spectrum of 3m'

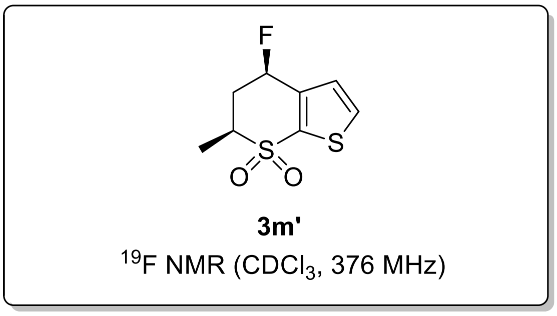

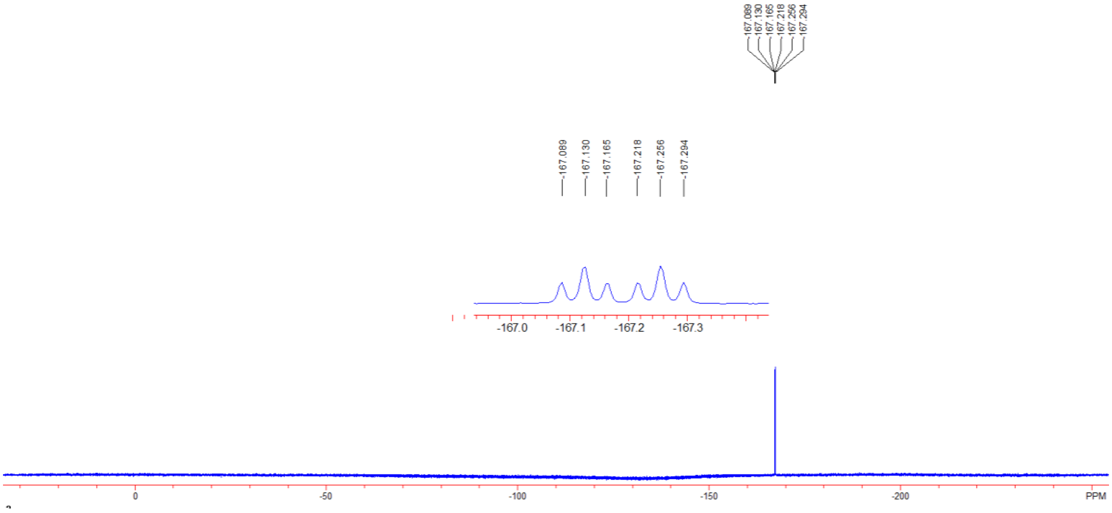

Supplementary Figure 68. ^{19}F NMR Spectrum of 3m'

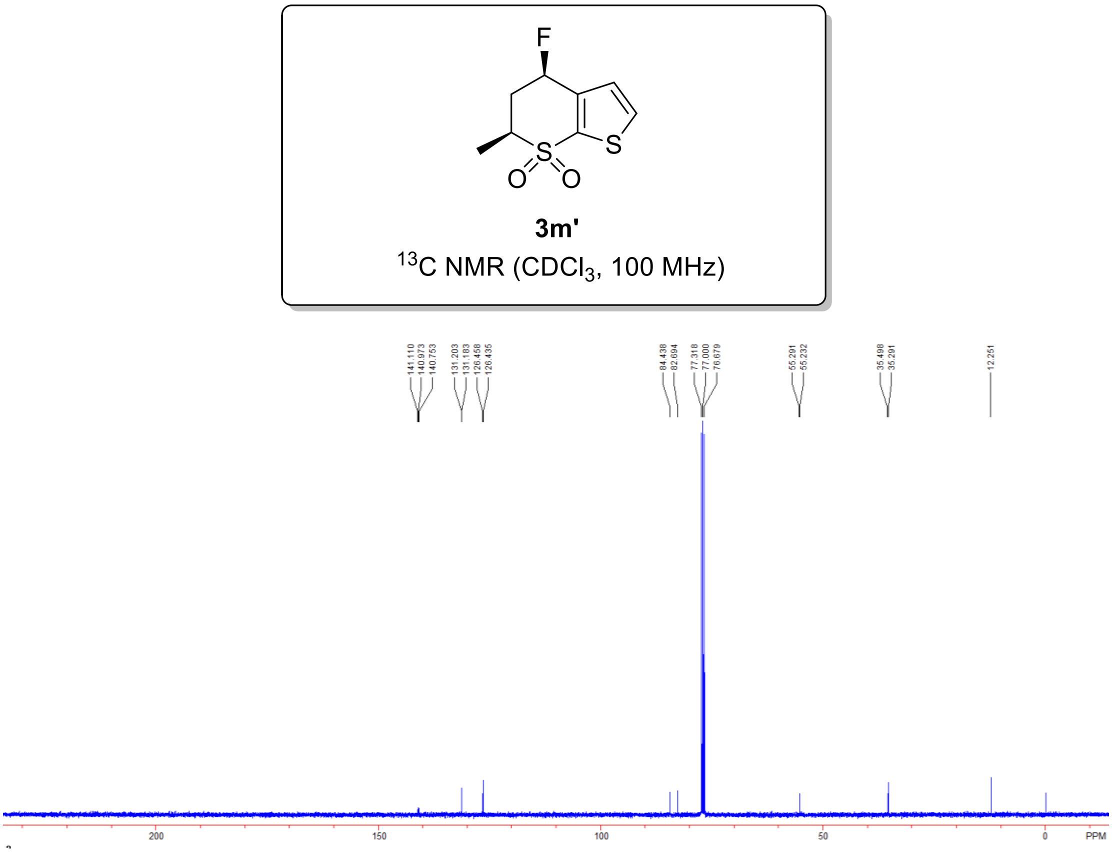

Supplementary Figure 69. ^{13}C NMR Spectrum of 3m'

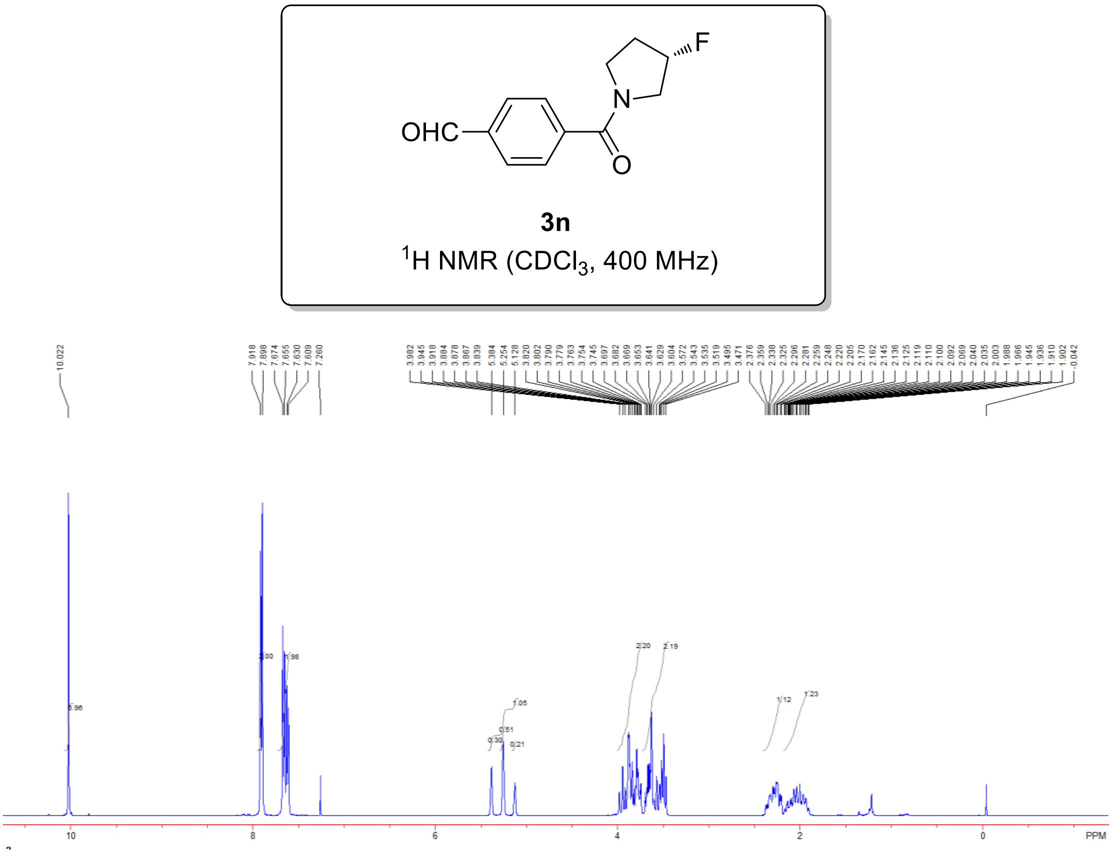

Supplementary Figure 70. ^{1}H NMR Spectrum of 3n

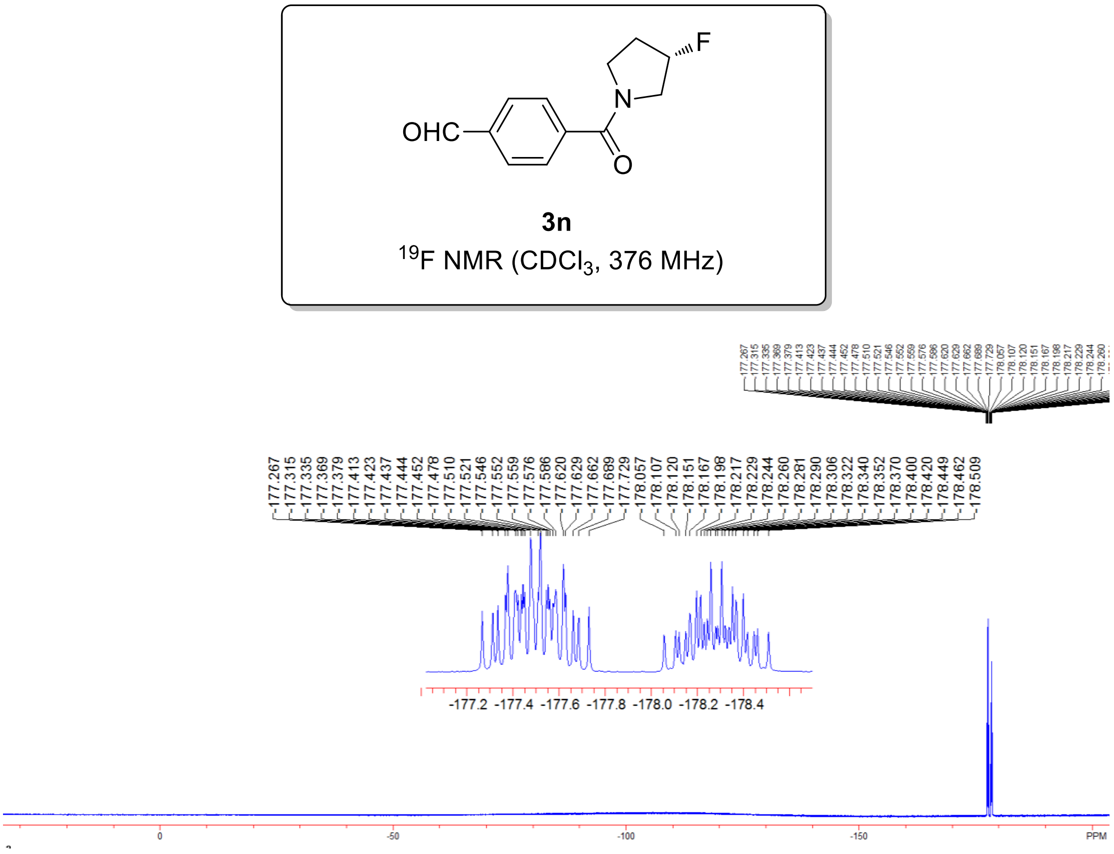

Supplementary Figure 71. ^{19}F NMR Spectrum of 3n

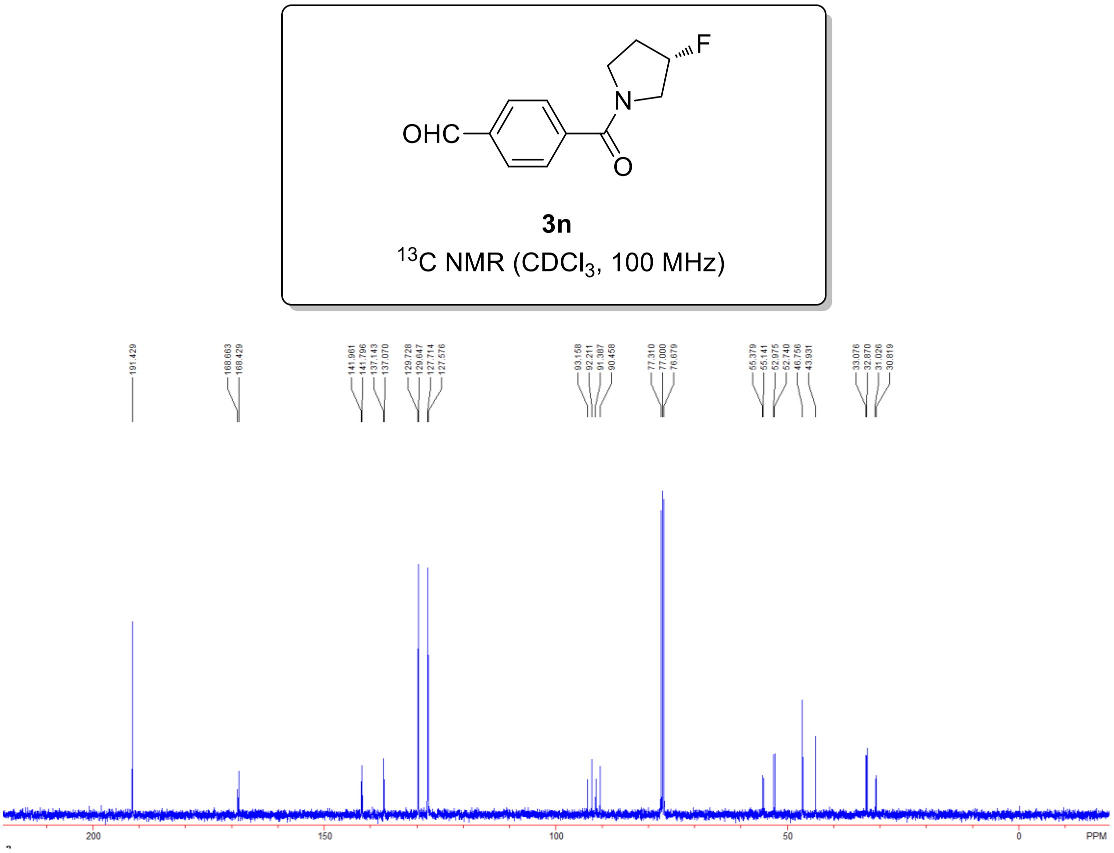

Supplementary Figure 72. ^{13}C NMR Spectrum of 3n

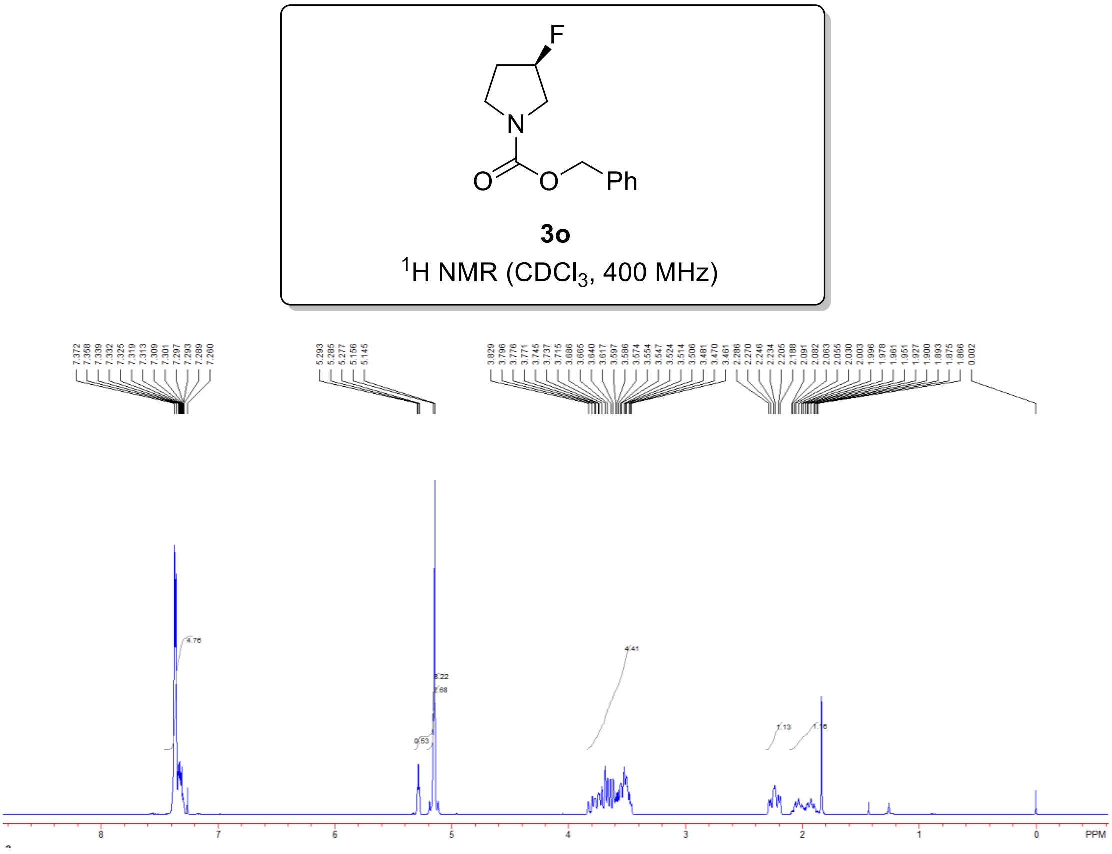

Supplementary Figure 73. ^{1}H NMR Spectrum of 3o

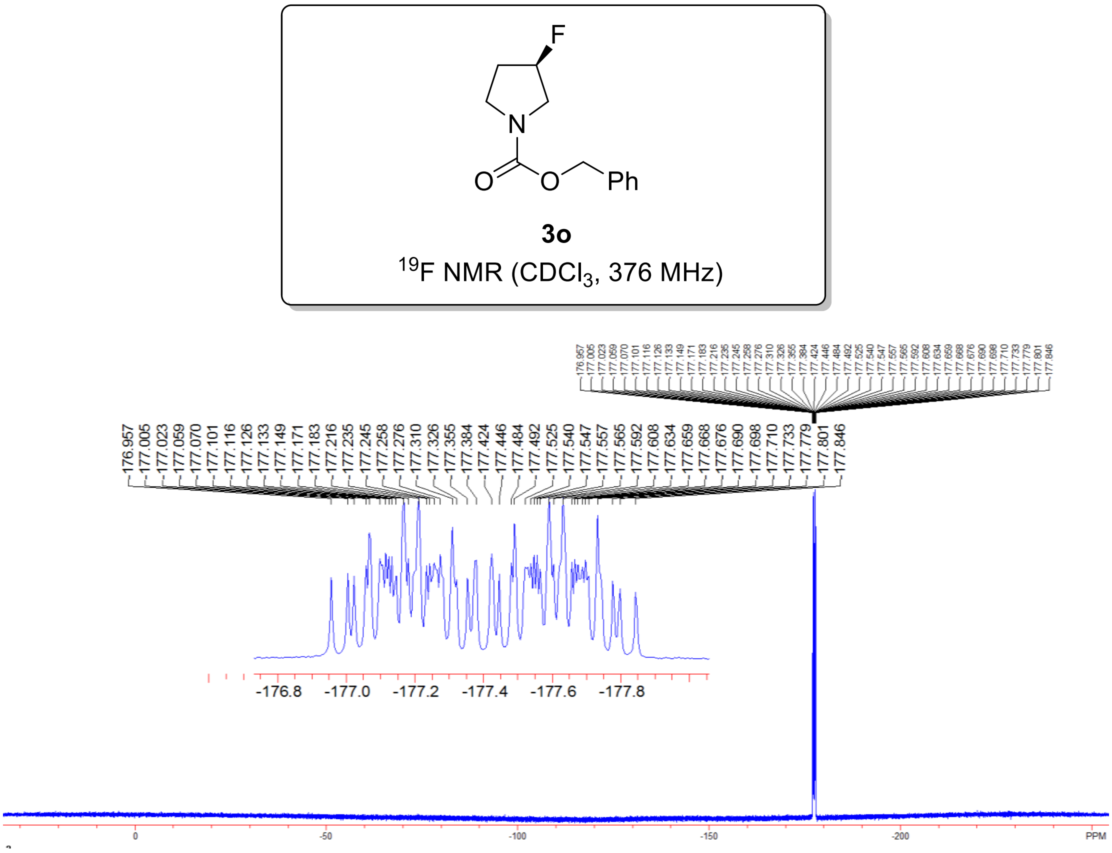

Supplementary Figure 74. ^{19}F NMR Spectrum of 3o

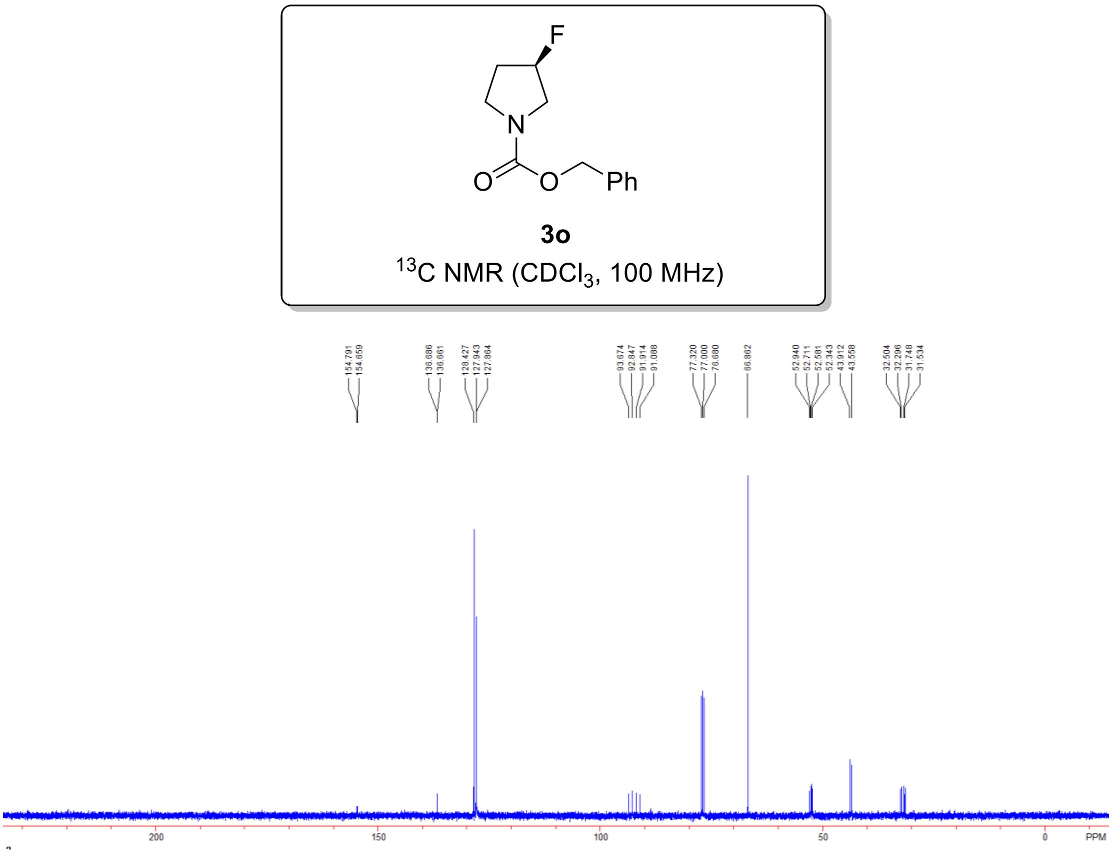

Supplementary Figure 75. ^{13}C NMR Spectrum of 3o

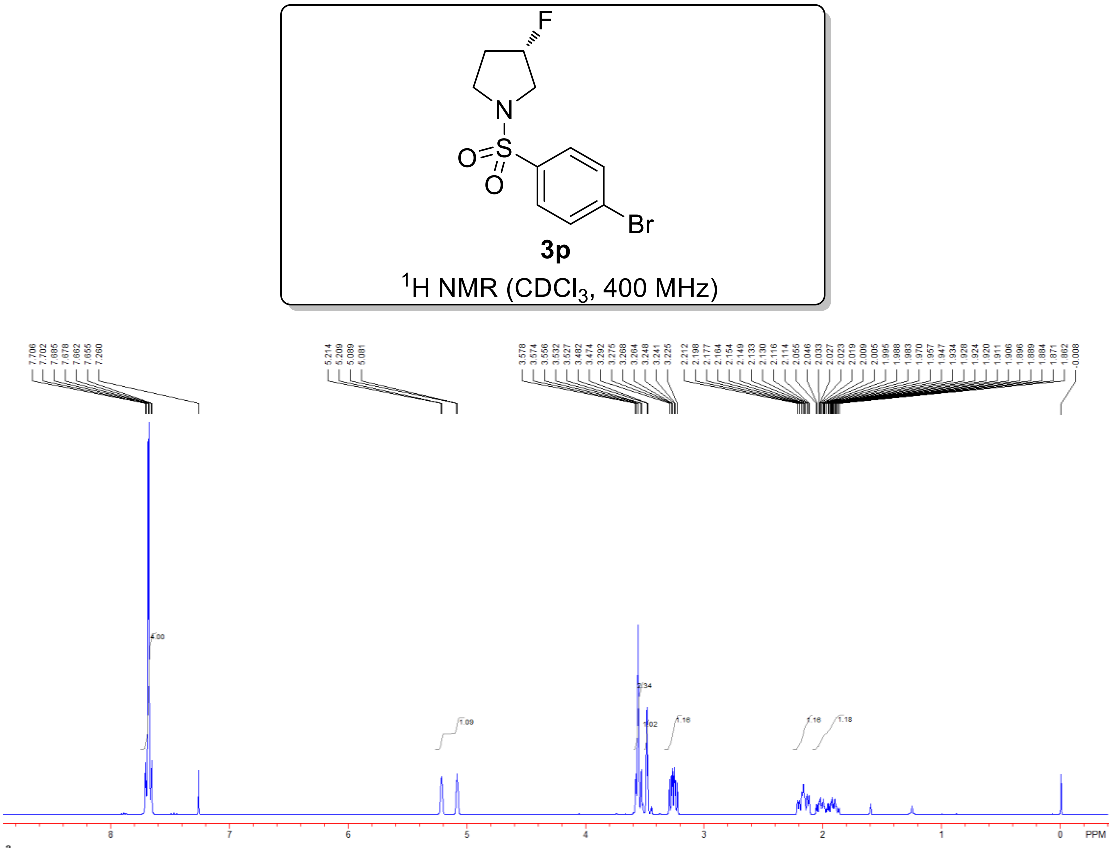

Supplementary Figure 76. ^{1}H NMR Spectrum of 3p

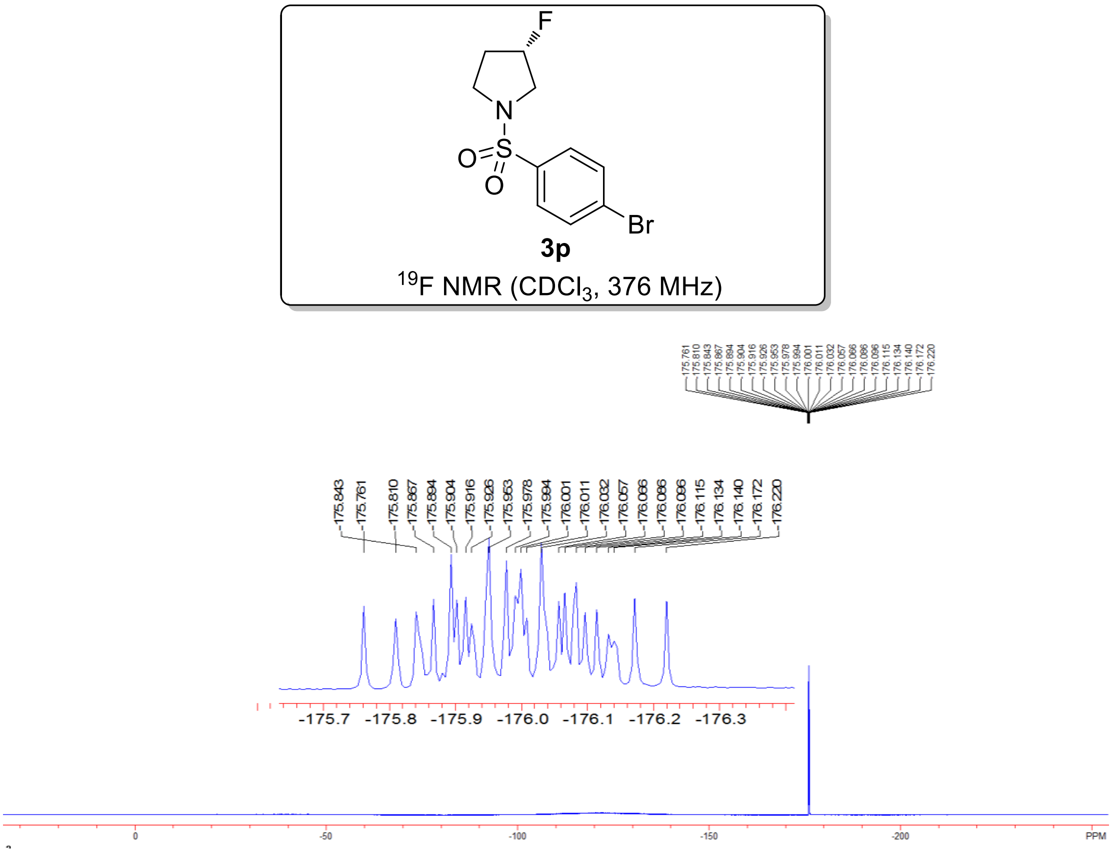

Supplementary Figure 77. ^{19}F NMR Spectrum of 3p

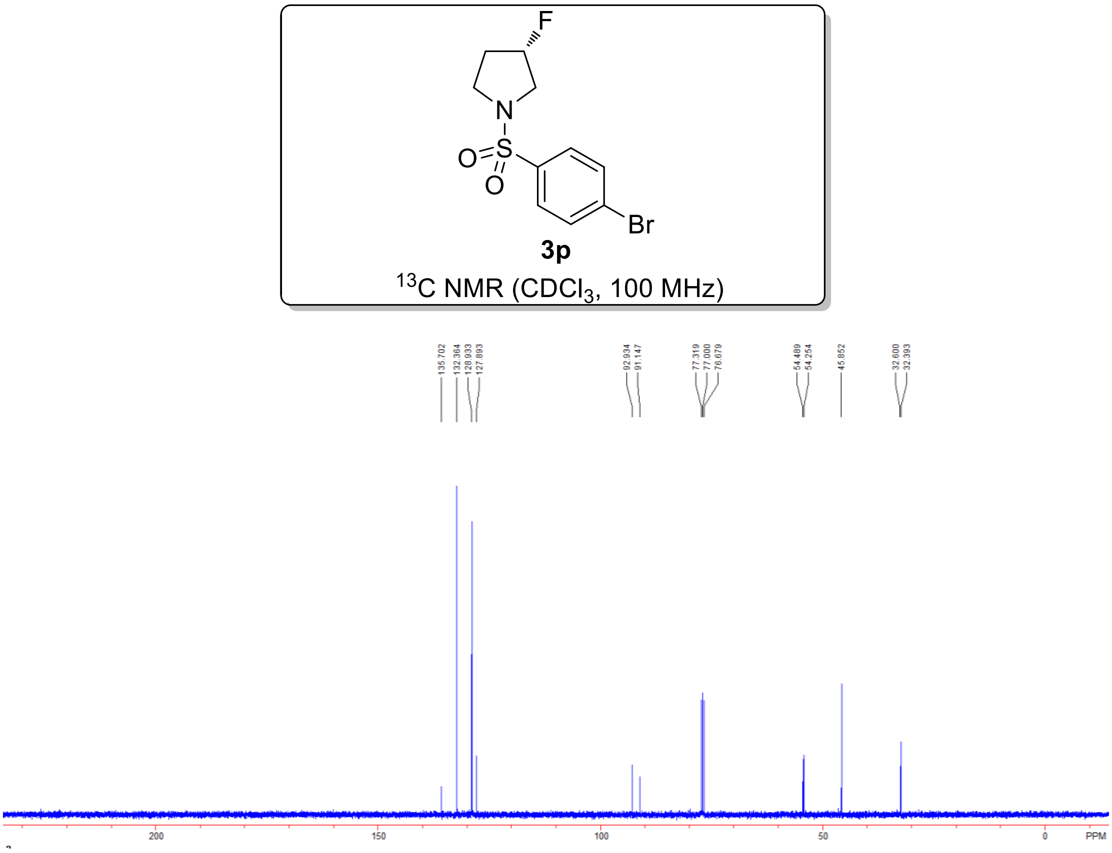

Supplementary Figure 78. ^{13}C NMR Spectrum of 3p

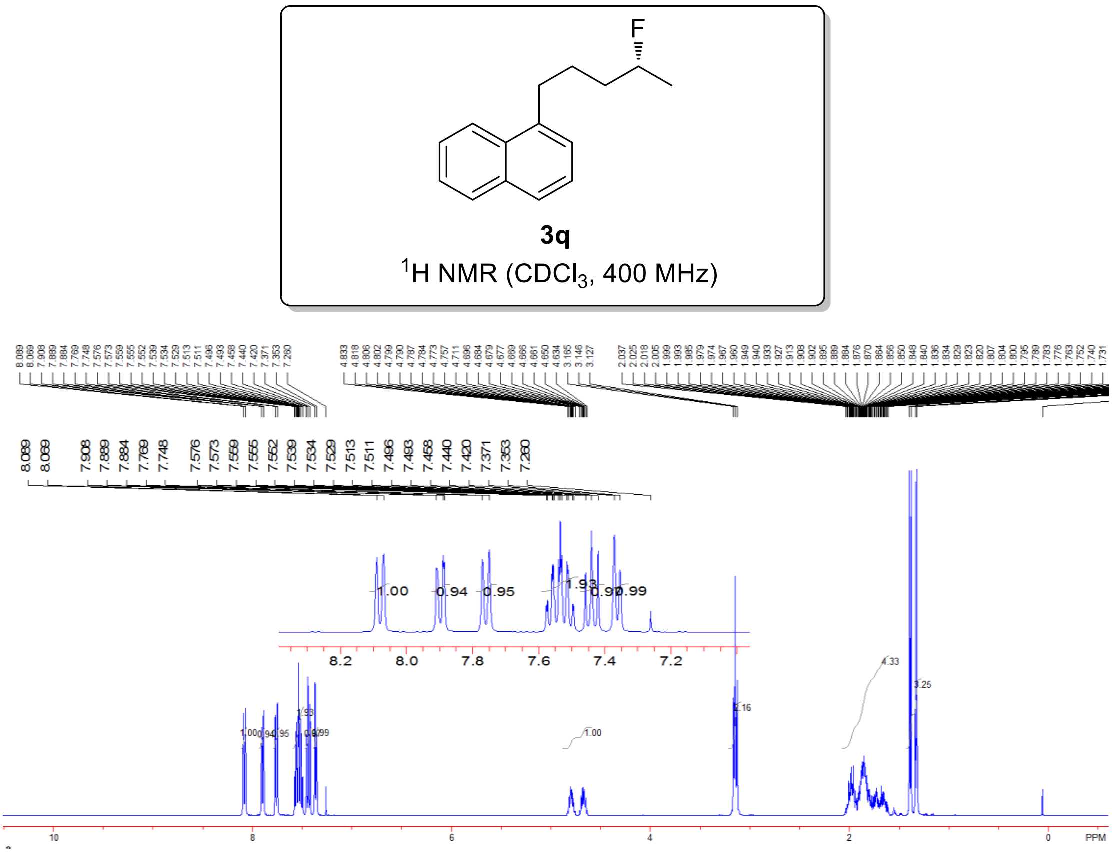

Supplementary Figure 79. ^{1}H NMR Spectrum of 3q

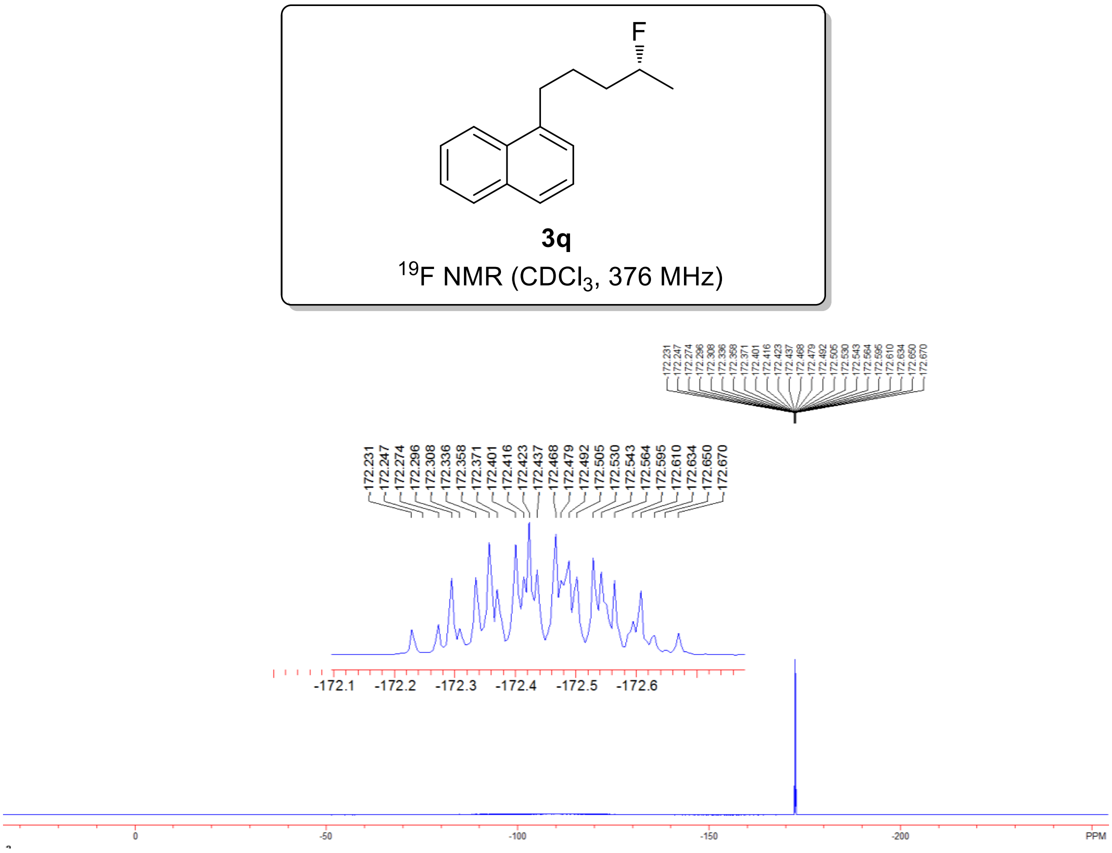

Supplementary Figure 80. ^{19}F NMR Spectrum of 3q

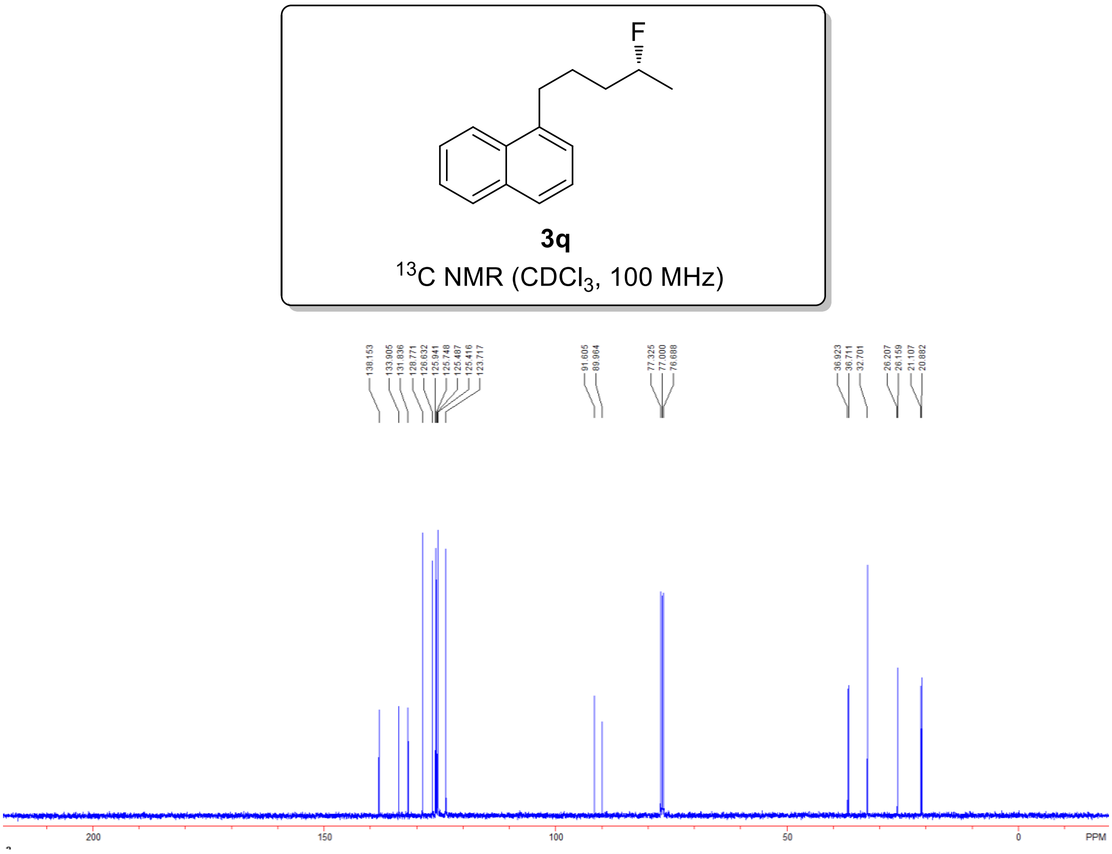

Supplementary Figure 81. ^{13}C NMR Spectrum of 3q

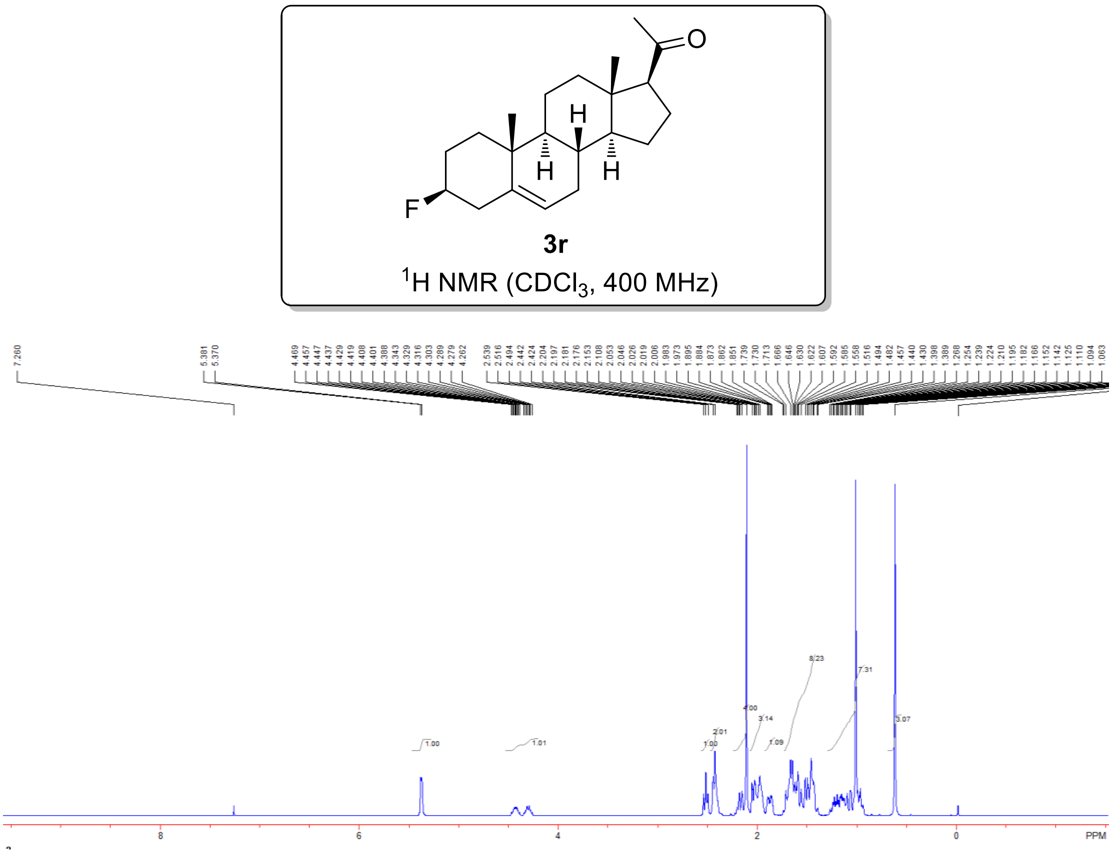

Supplementary Figure 82. ^{1}H NMR Spectrum of 3r

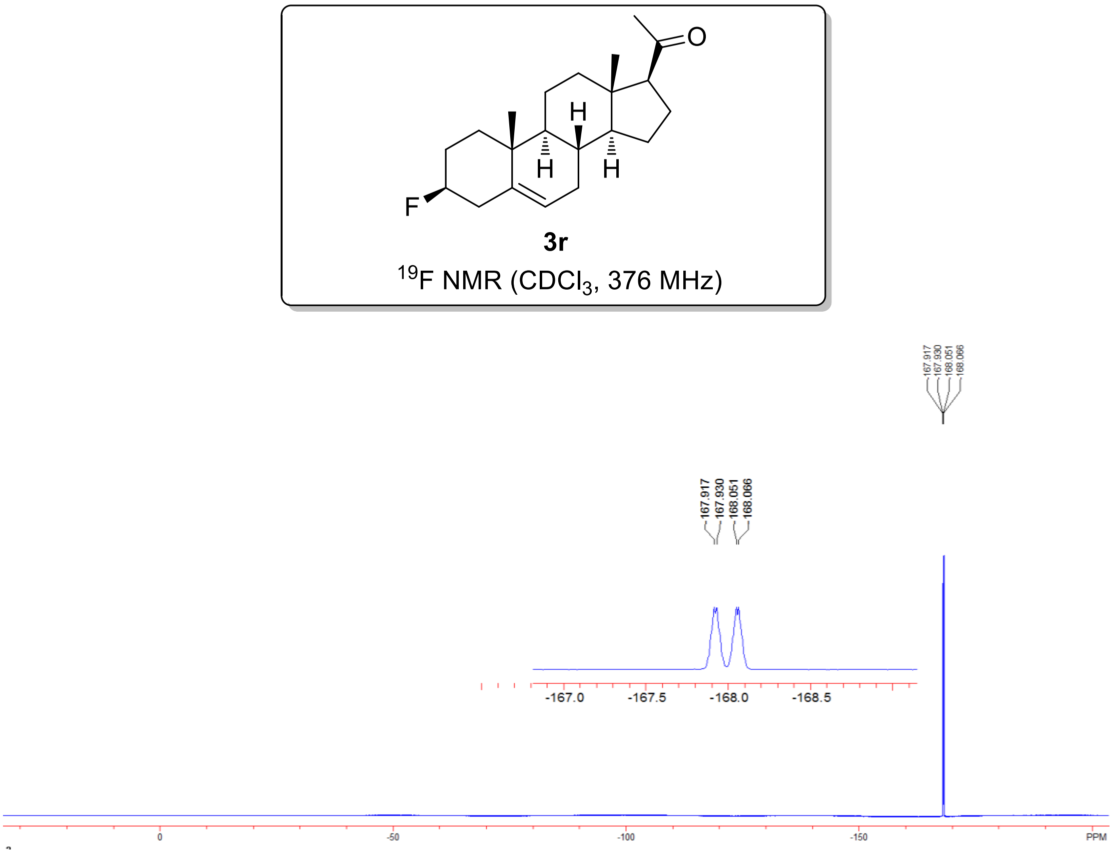

Supplementary Figure 83. ^{19}F NMR Spectrum of 3r

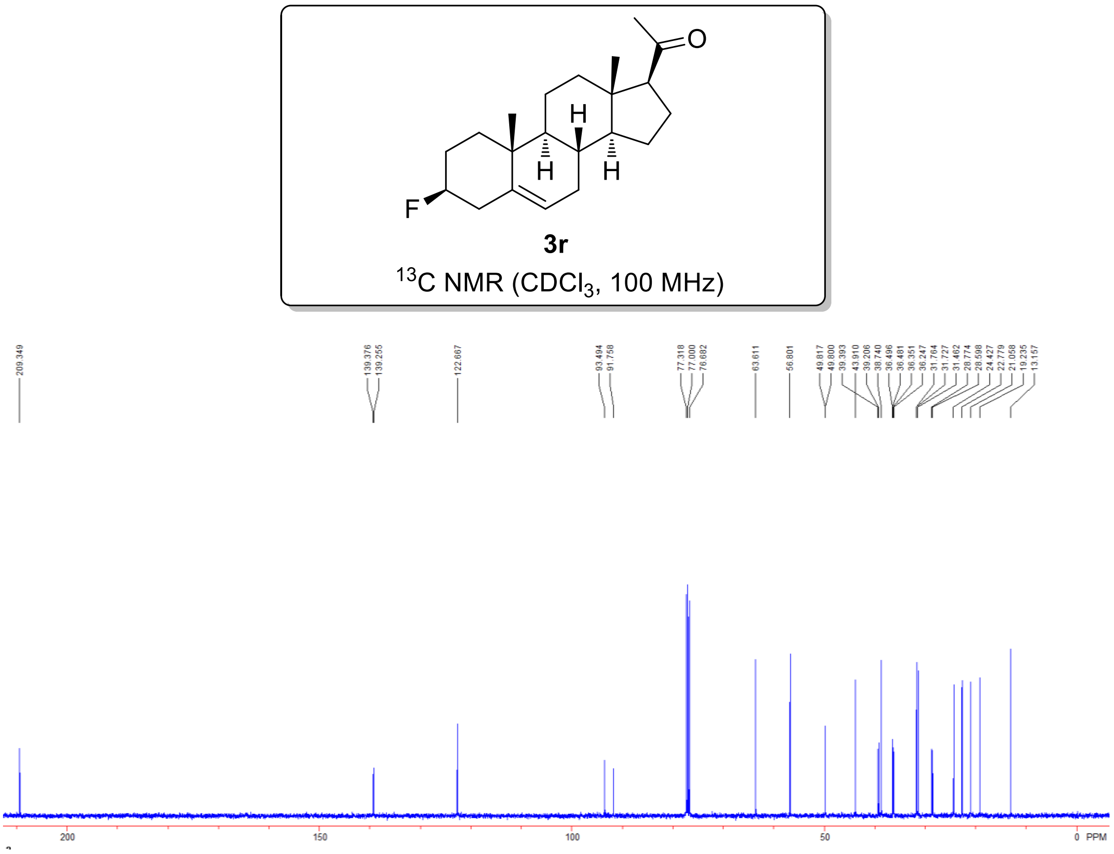

Supplementary Figure 84. ^{13}C NMR Spectrum of 3r

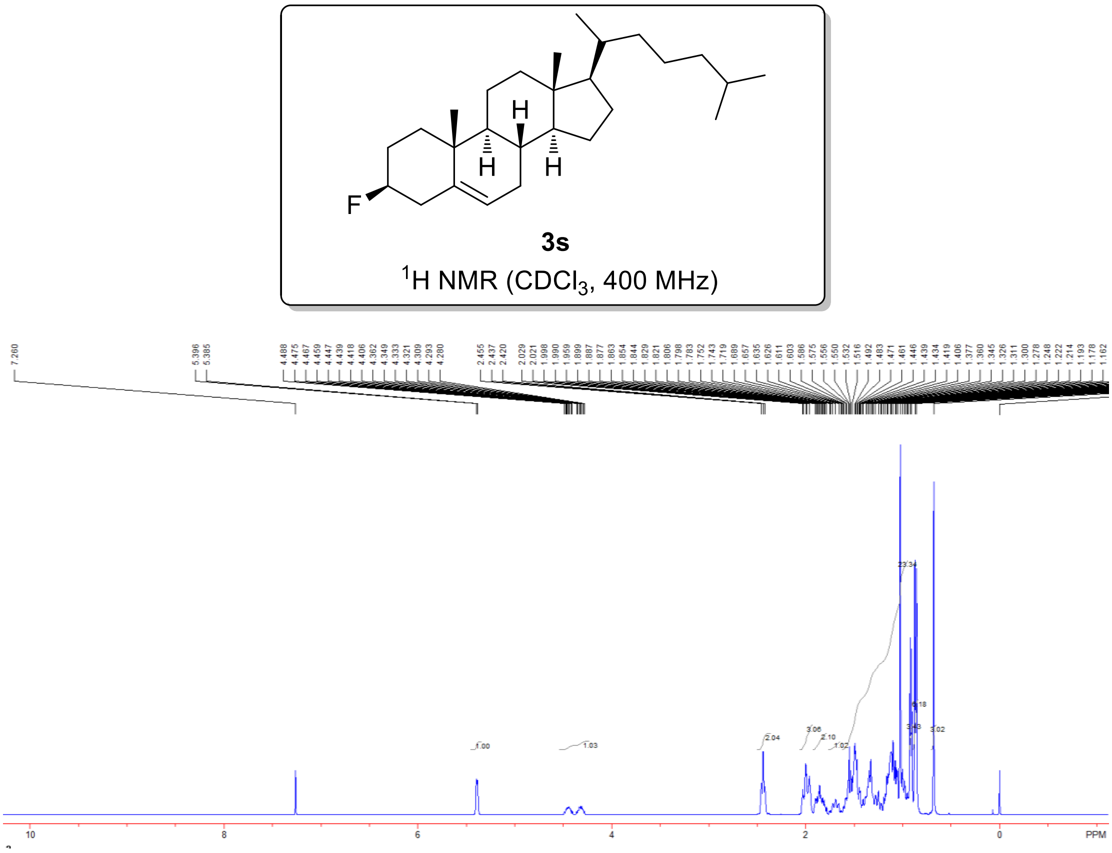

Supplementary Figure 85. ^{1}H NMR Spectrum of 3s

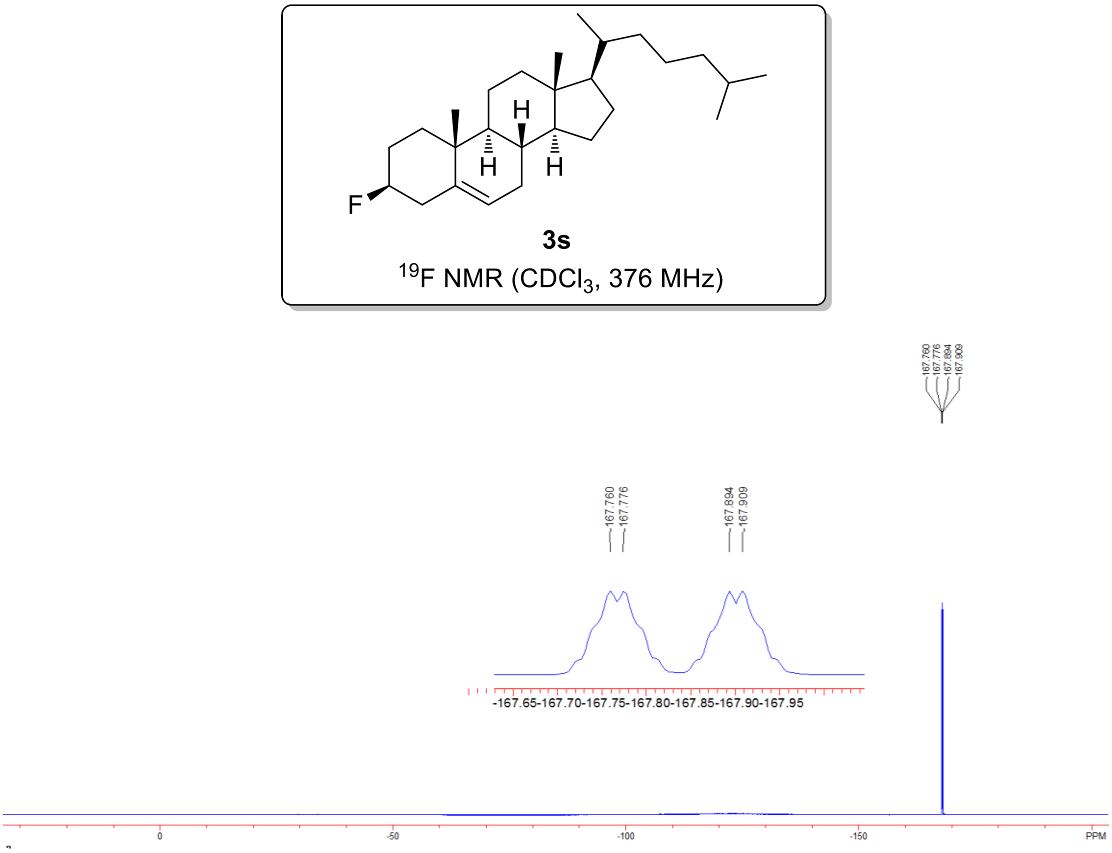

Supplementary Figure 86. ^{19}F NMR Spectrum of 3s

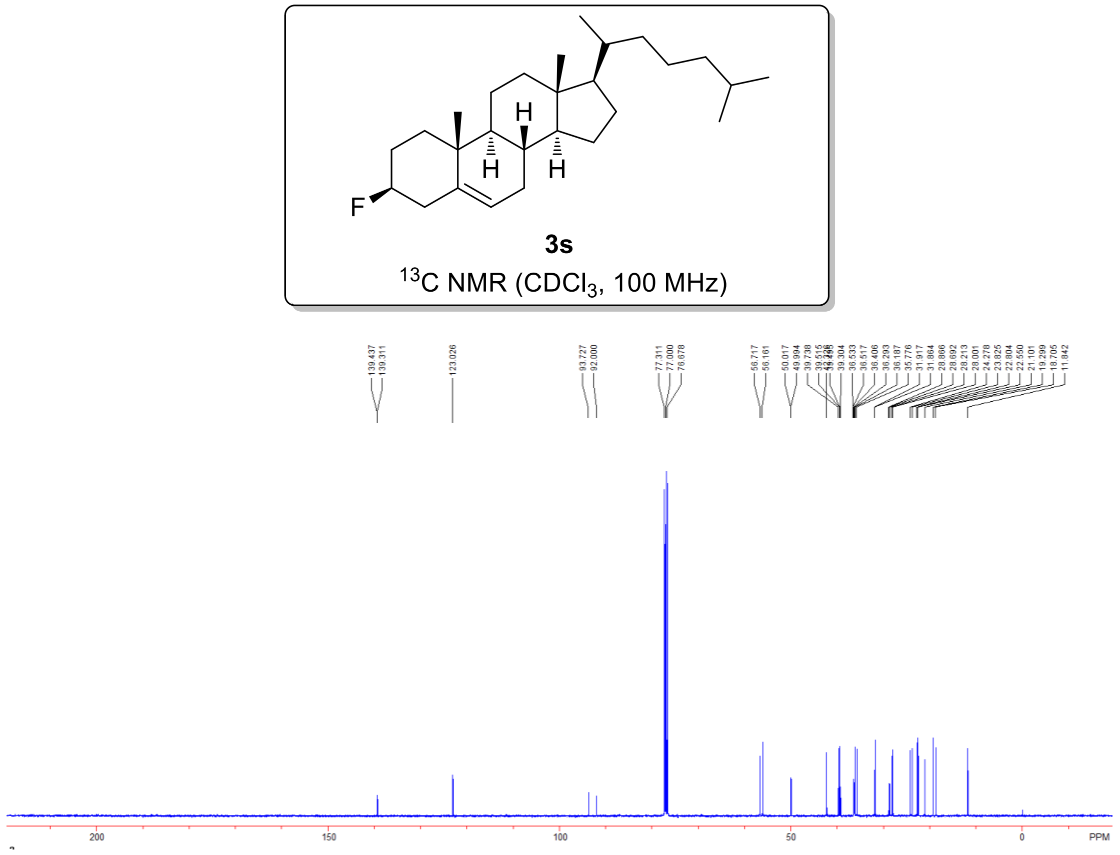

Supplementary Figure 87. ^{13}C NMR Spectrum of 3s

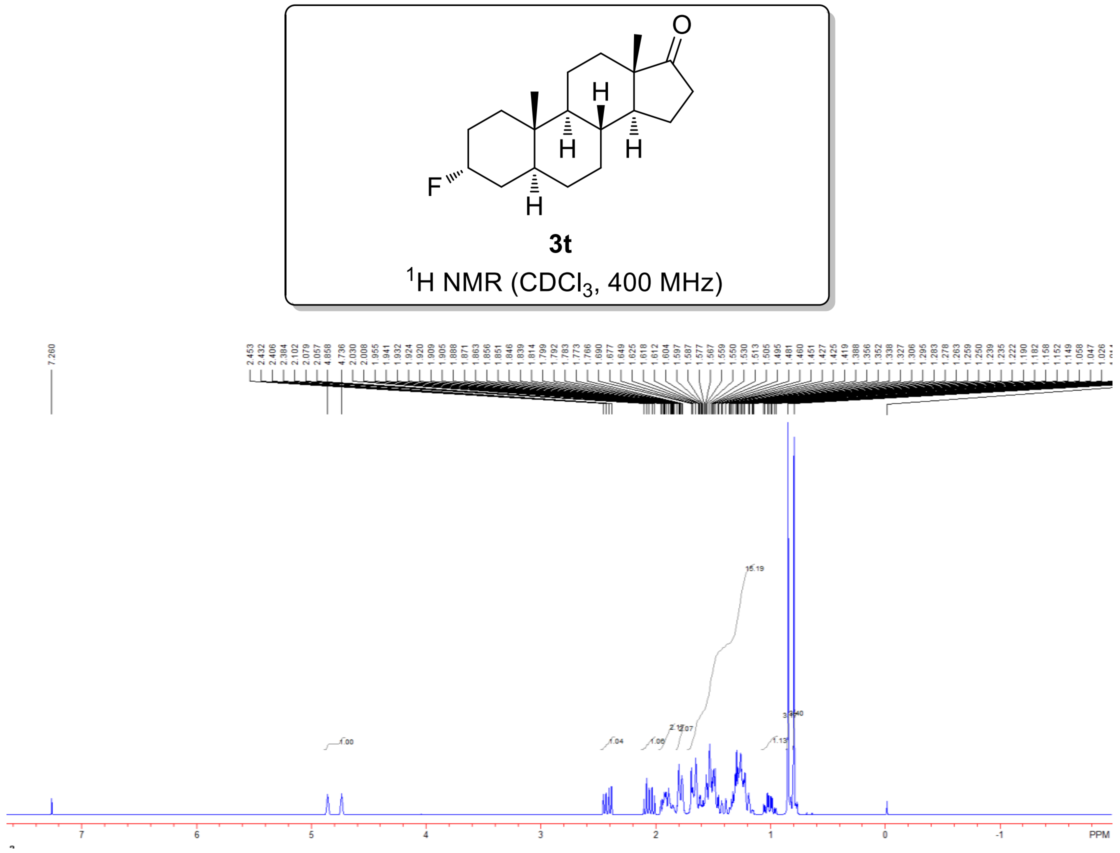

Supplementary Figure 88. ^{1}H NMR Spectrum of 3t

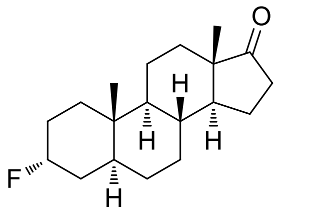

3t

^{19}F NMR ($CDCl_3$, 376 MHz)

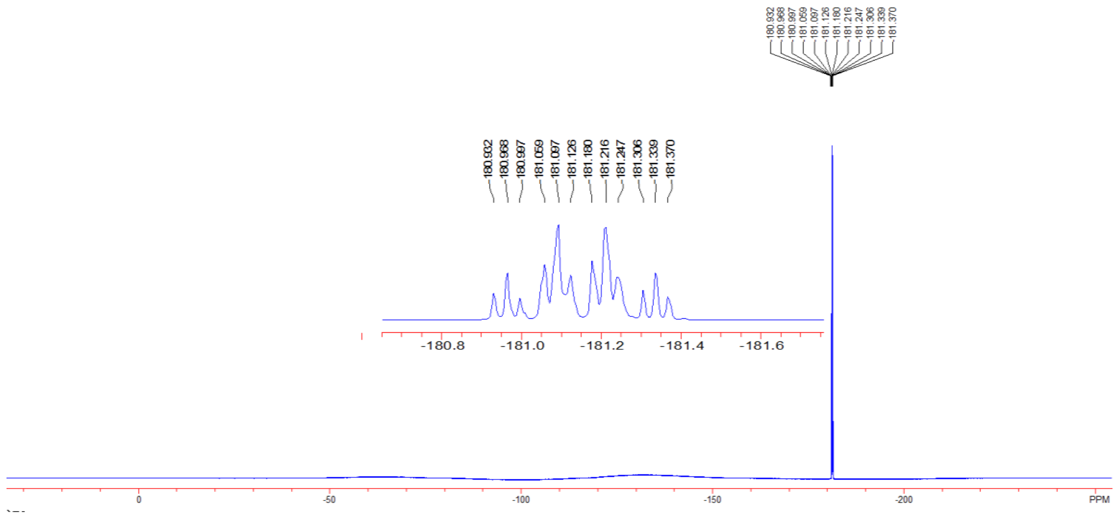

Supplementary Figure 89. ^{19}F NMR Spectrum of 3t

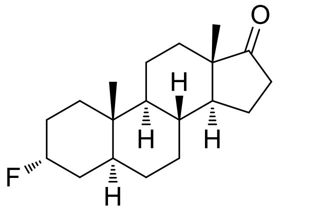

3t

^{13}C NMR ($CDCl_3$, 100 MHz)

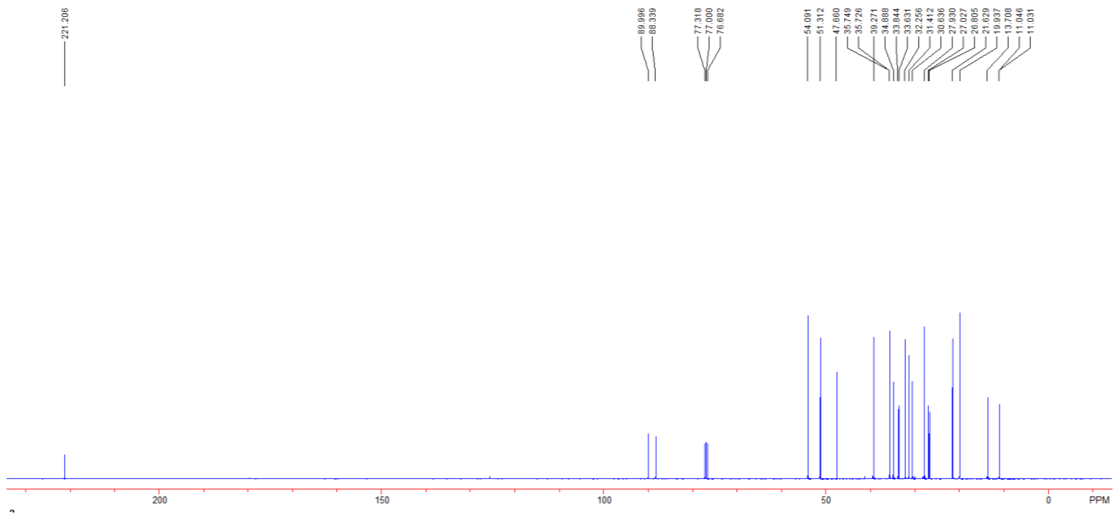

Supplementary Figure 90. ^{13}C NMR Spectrum of 3t

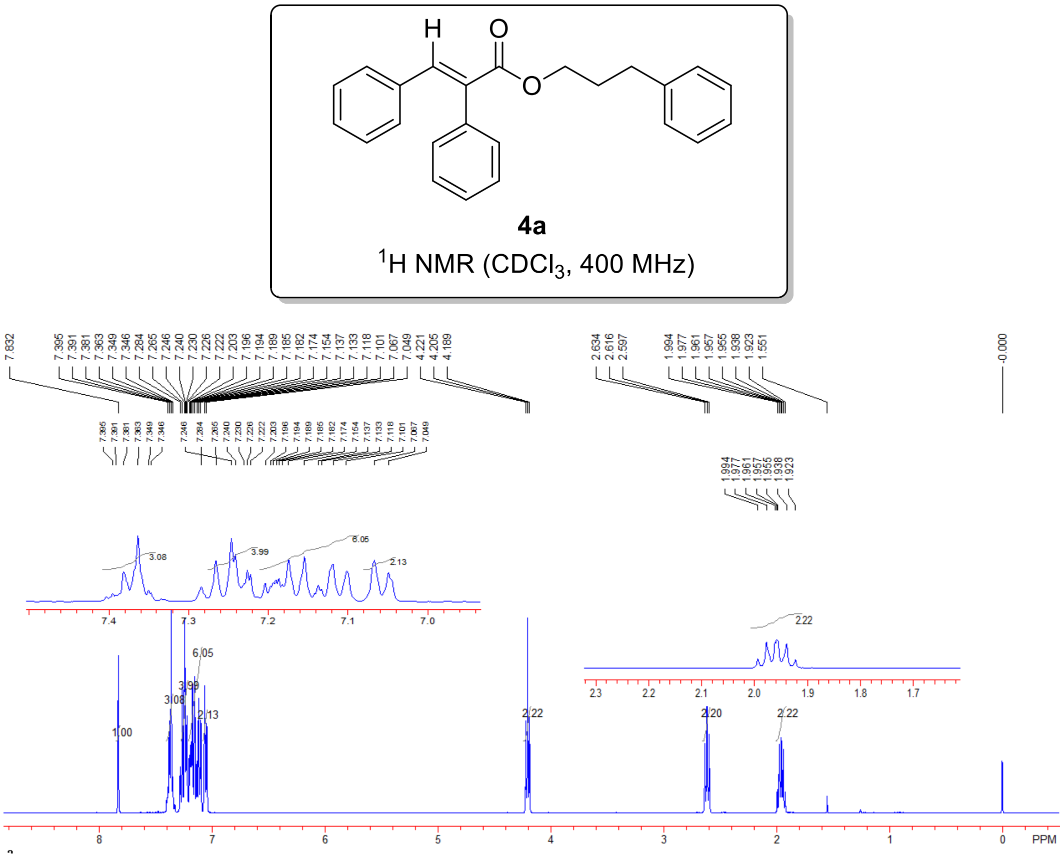

Supplementary Figure 91. ^{1}H NMR Spectrum of 4a

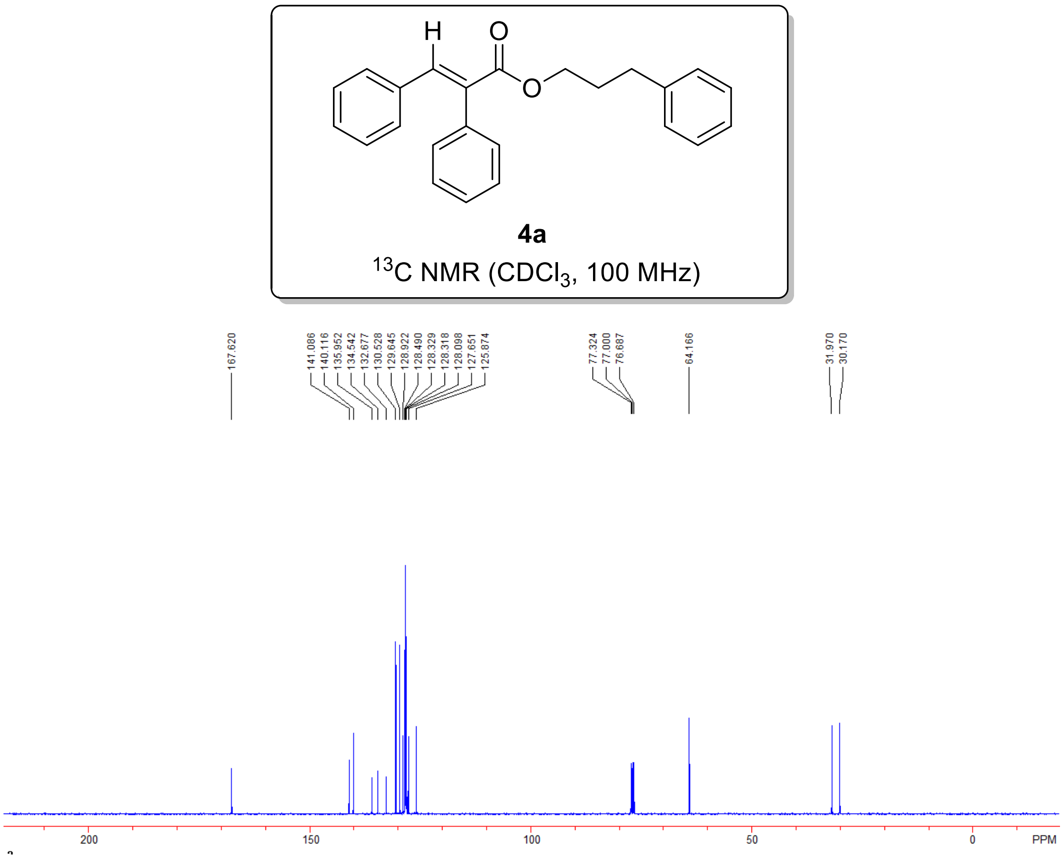

Supplementary Figure 92. ^{13}C NMR Spectrum of 4a

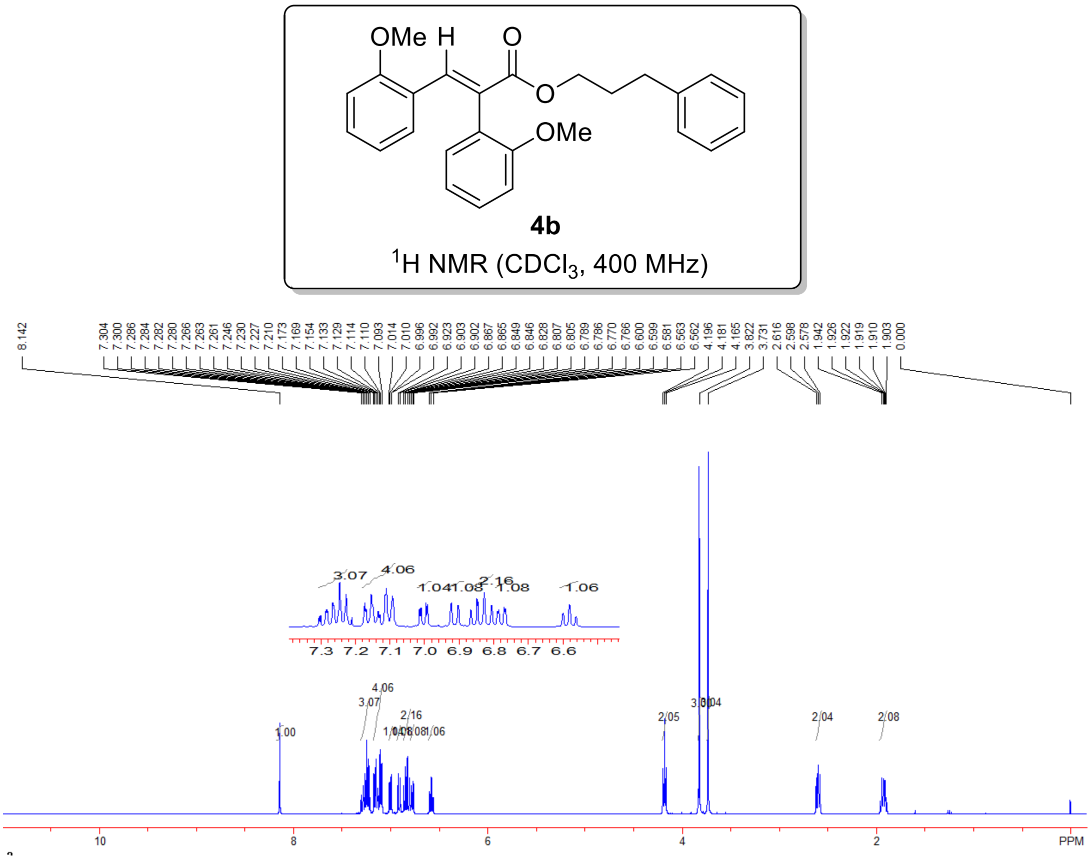

Supplementary Figure 93. ^{1}H NMR Spectrum of 4b

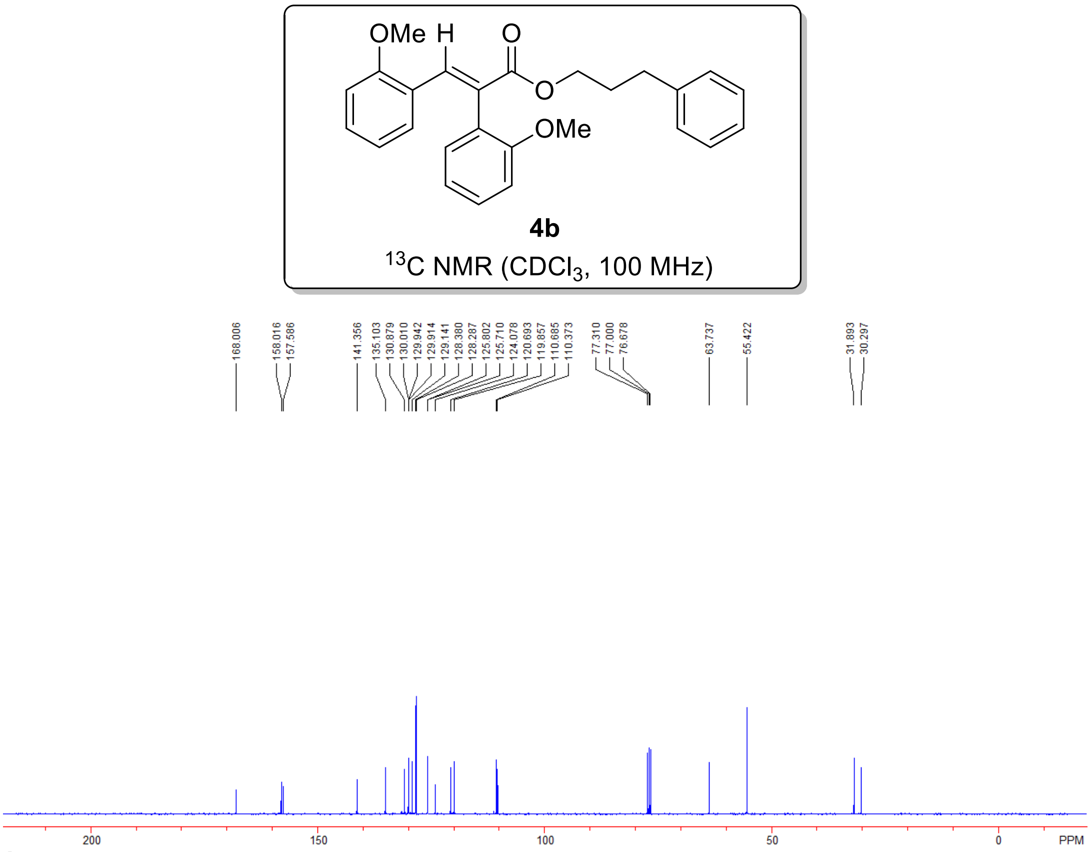

Supplementary Figure 94. ^{13}C NMR Spectrum of 4b

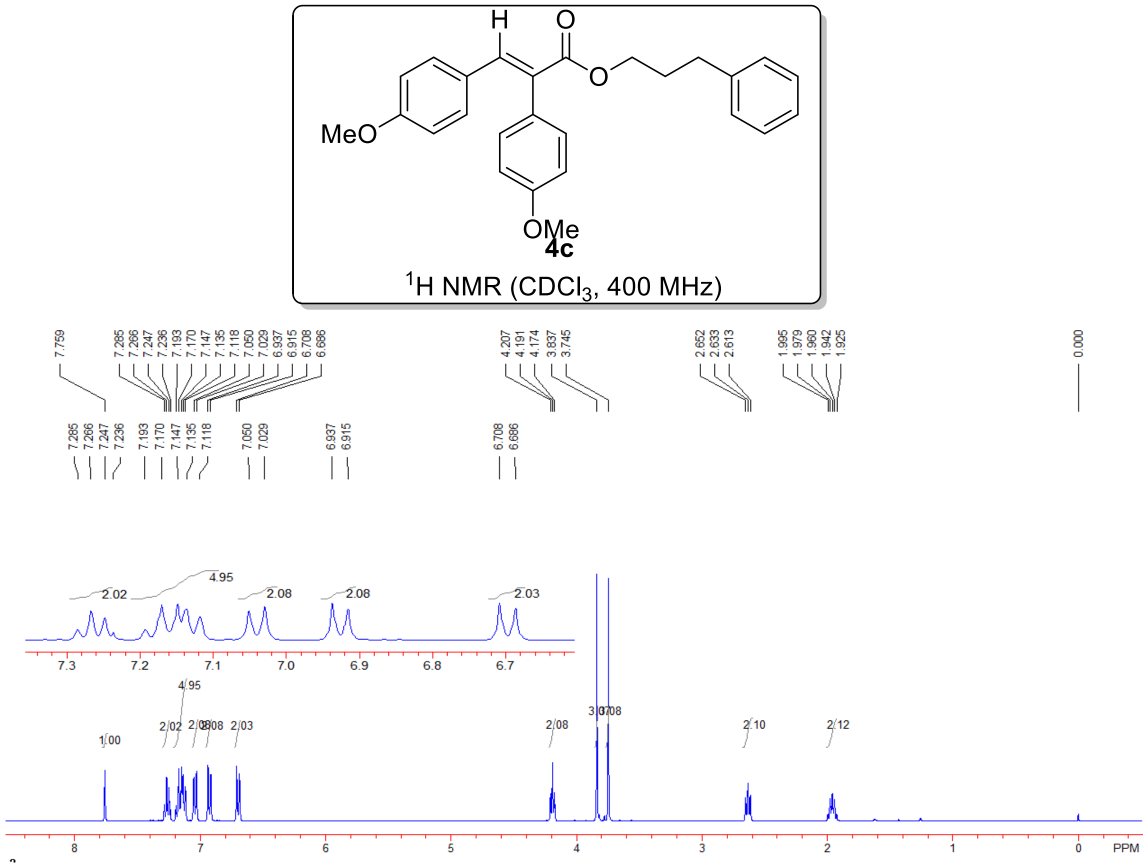

Supplementary Figure 95. ^{1}H NMR Spectrum of 4c

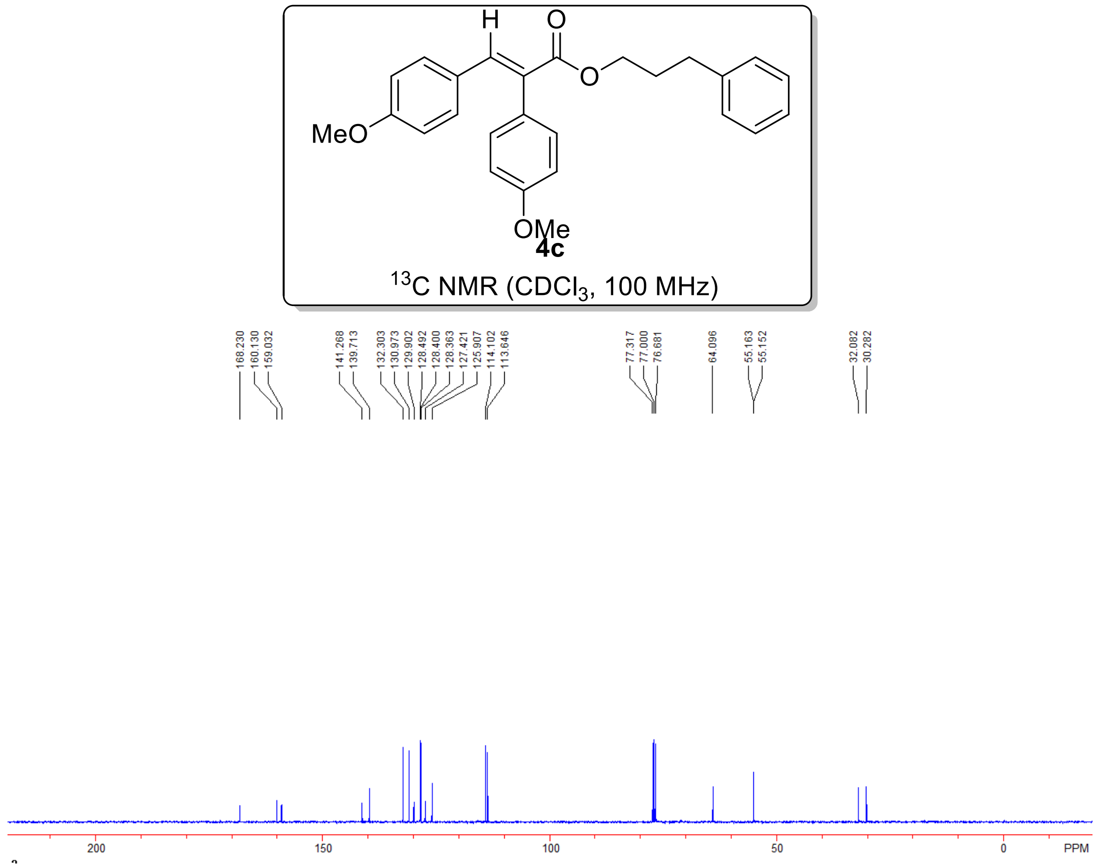

Supplementary Figure 96. ^{13}C NMR Spectrum of 4c

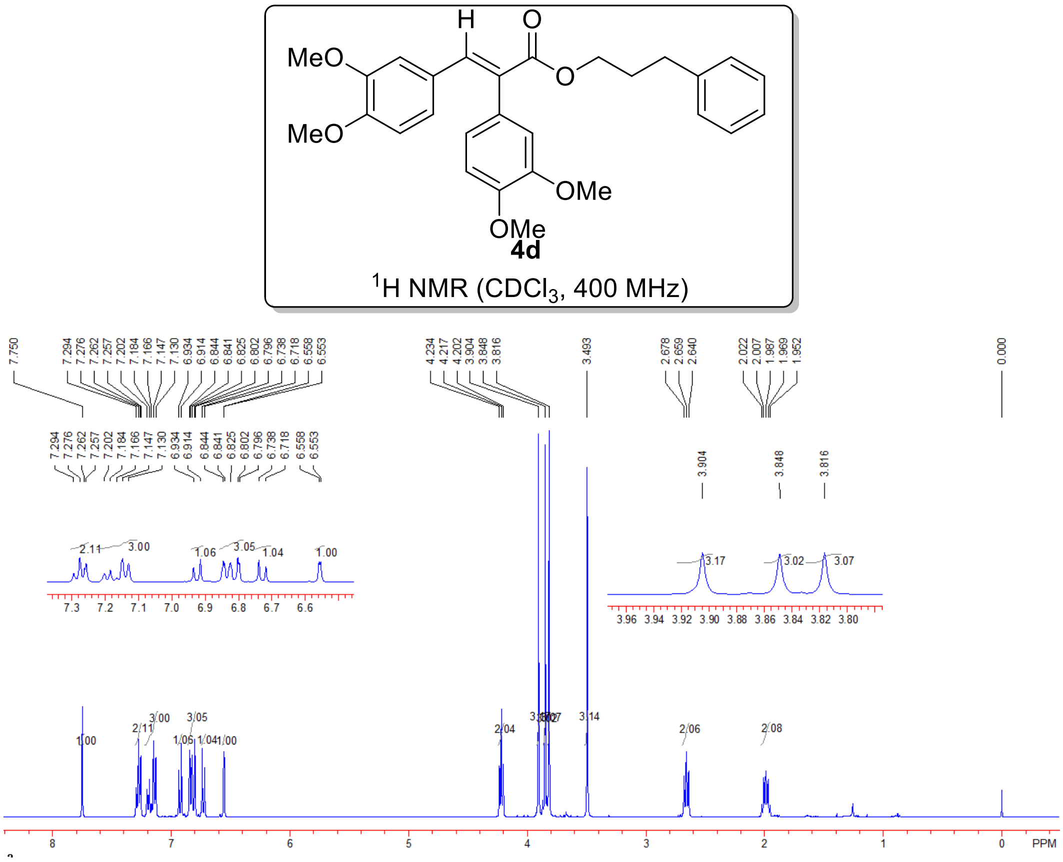

Supplementary Figure 97. ^{1}H NMR Spectrum of 4d

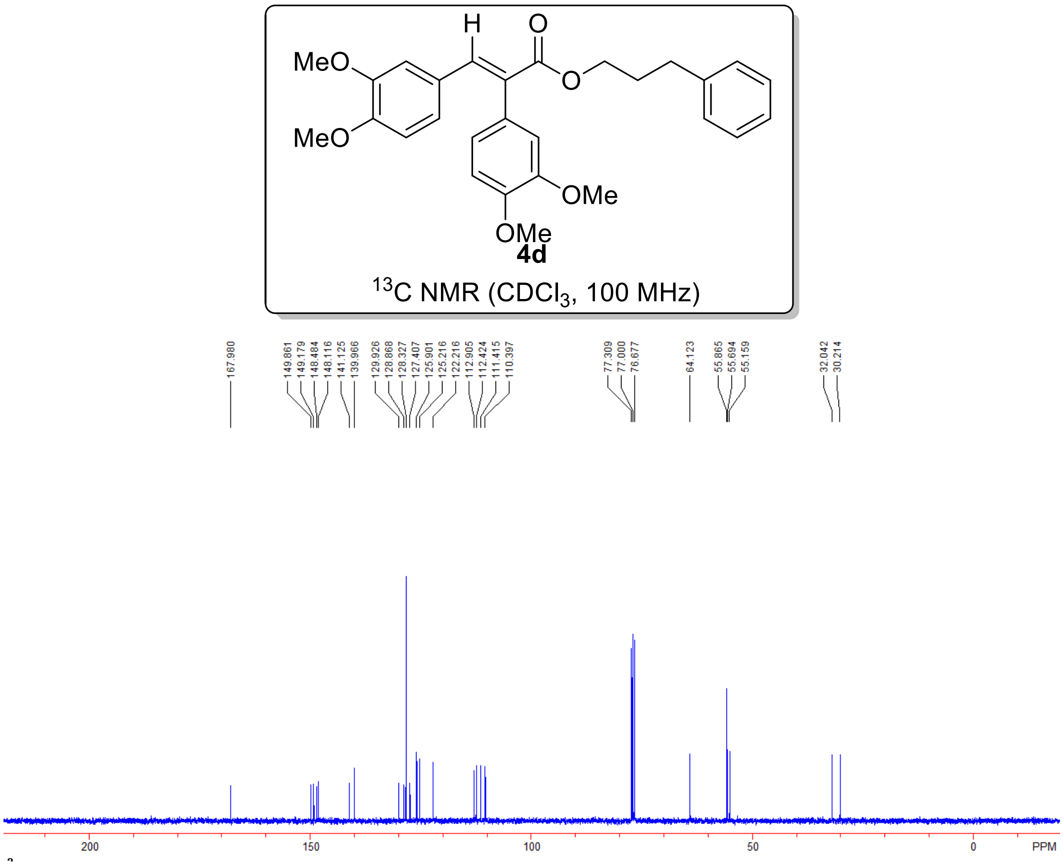

Supplementary Figure 98. ^{13}C NMR Spectrum of 4d

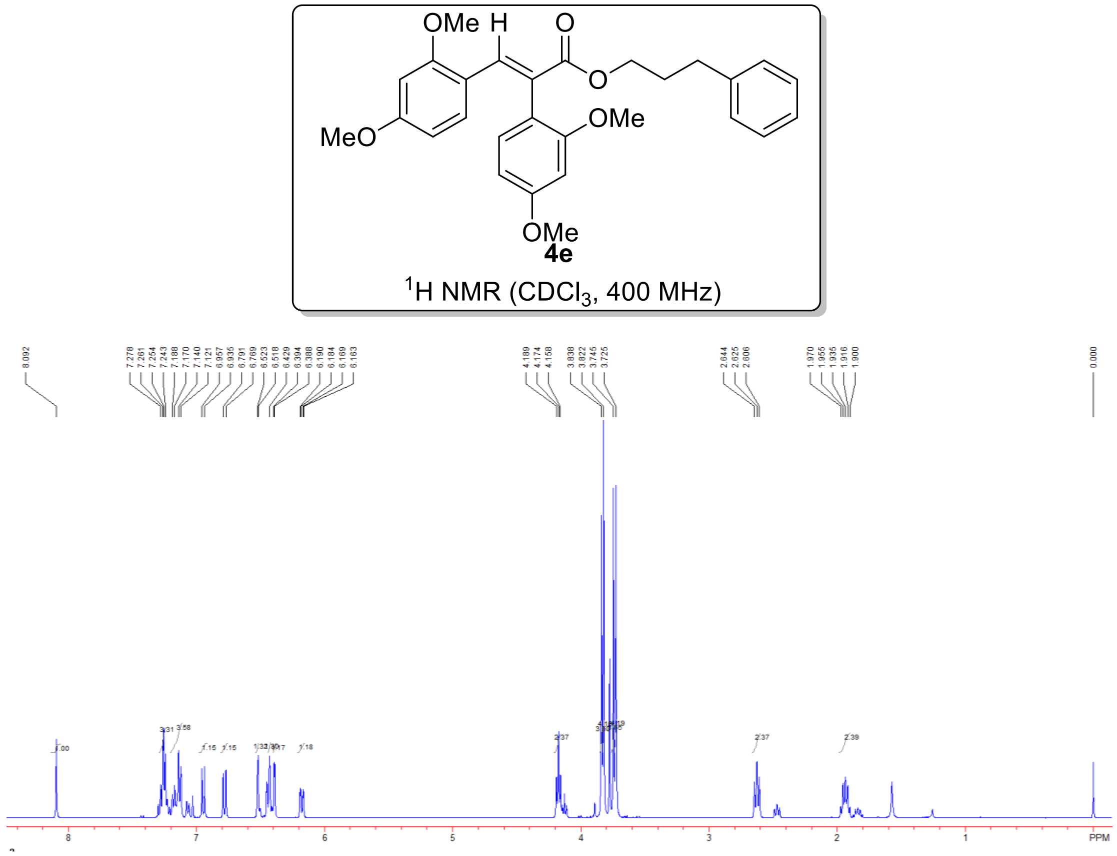

Supplementary Figure 99. ^{1}H NMR Spectrum of 4e

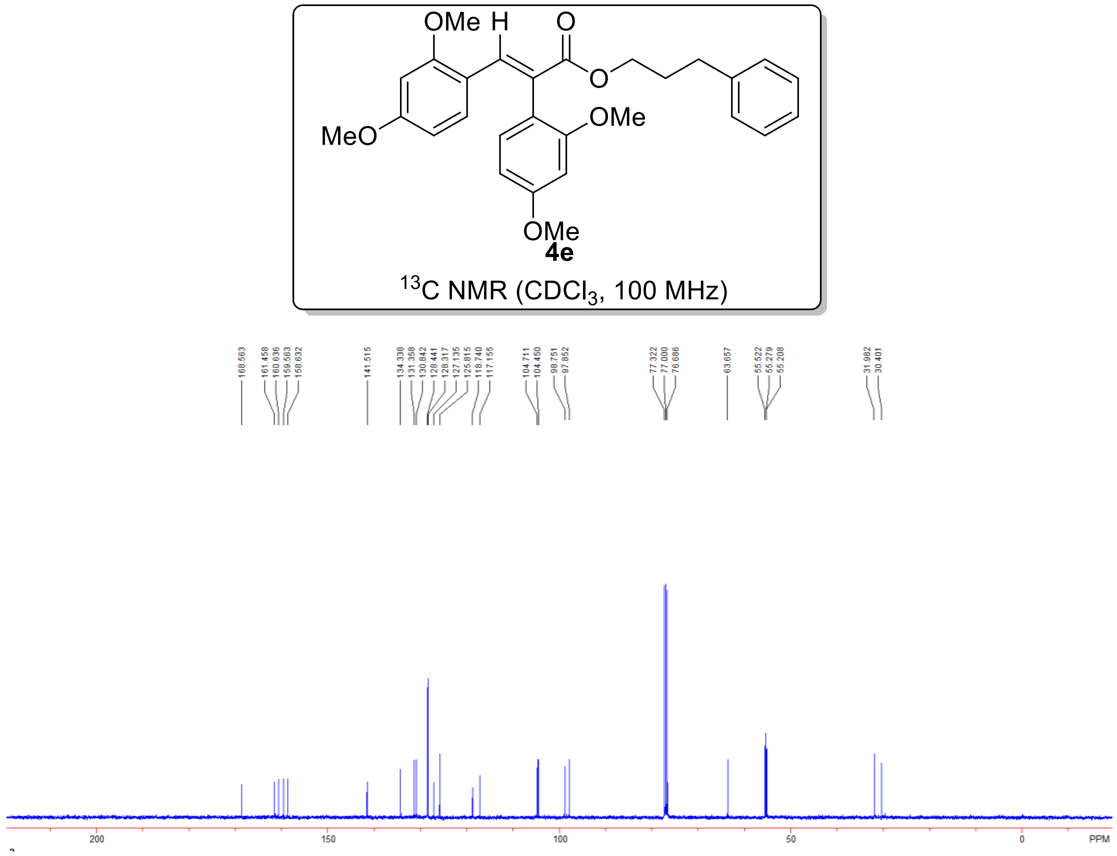

Supplementary Figure 100. ^{13}C NMR Spectrum of 4e

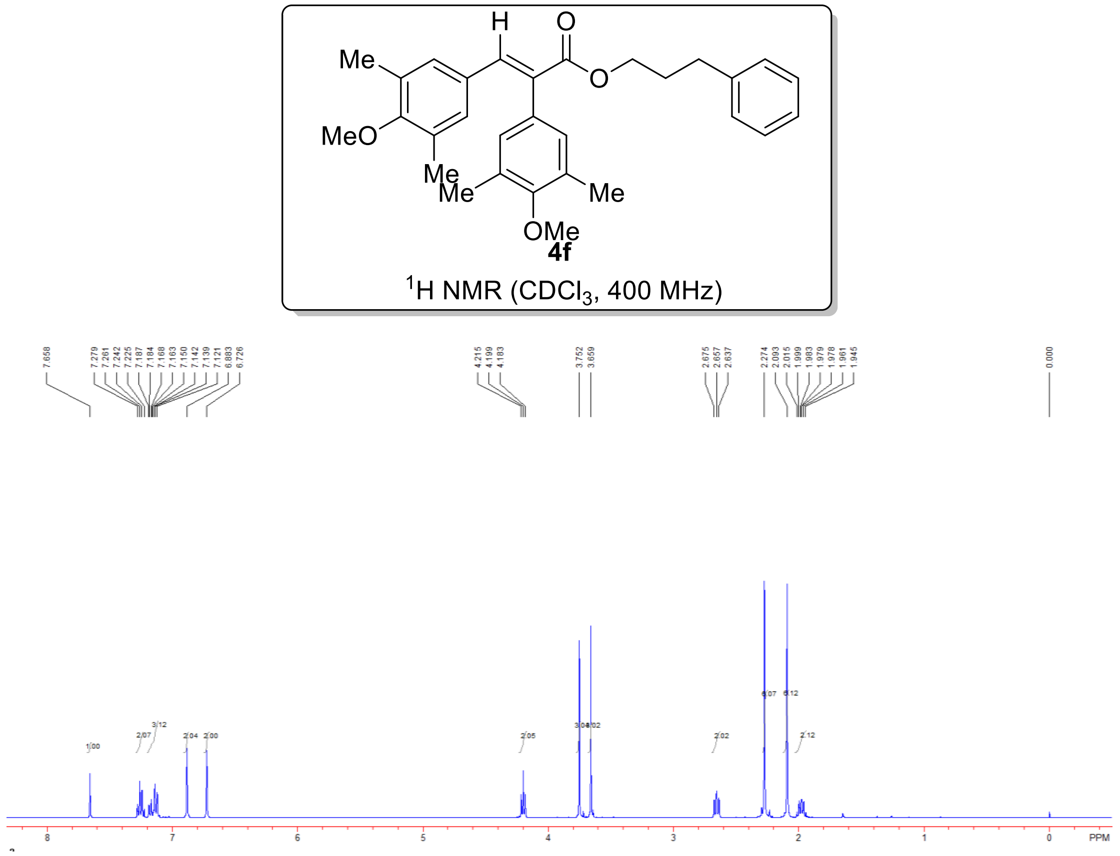

Supplementary Figure 101. ^{1}H NMR Spectrum of 4f

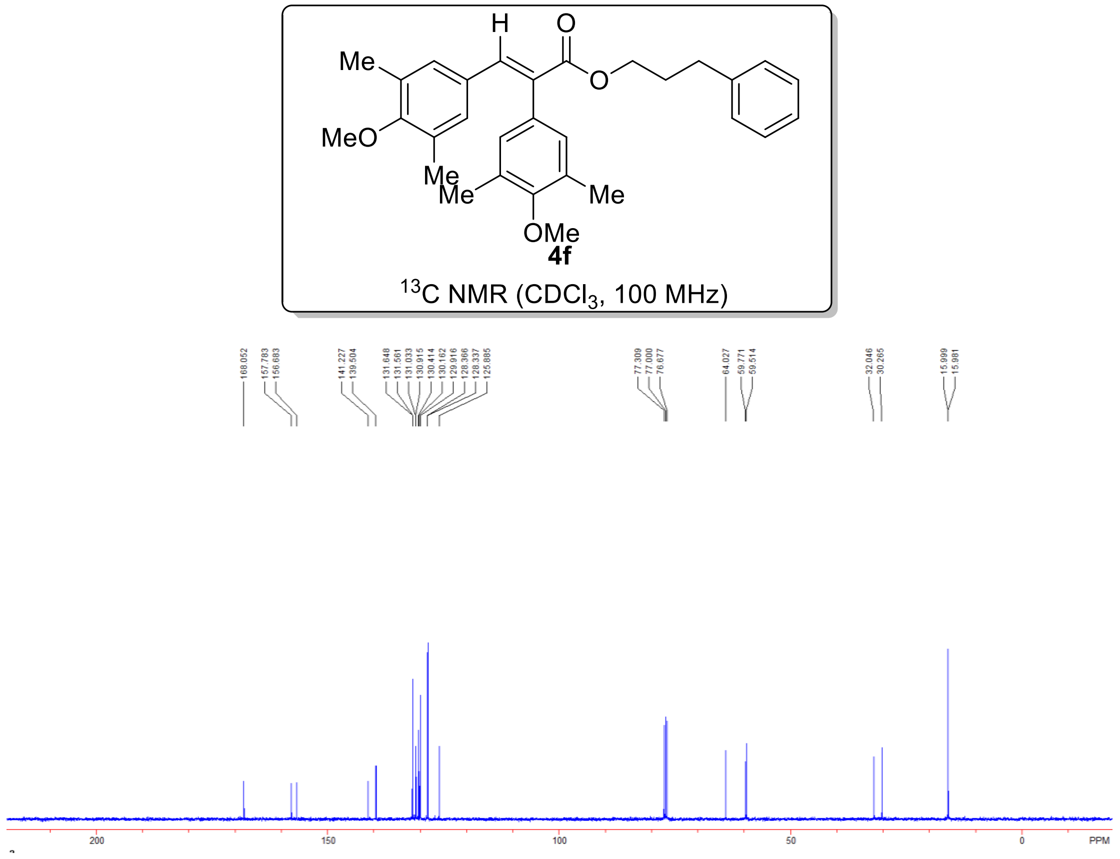

Supplementary Figure 102. ^{13}C NMR Spectrum of 4f

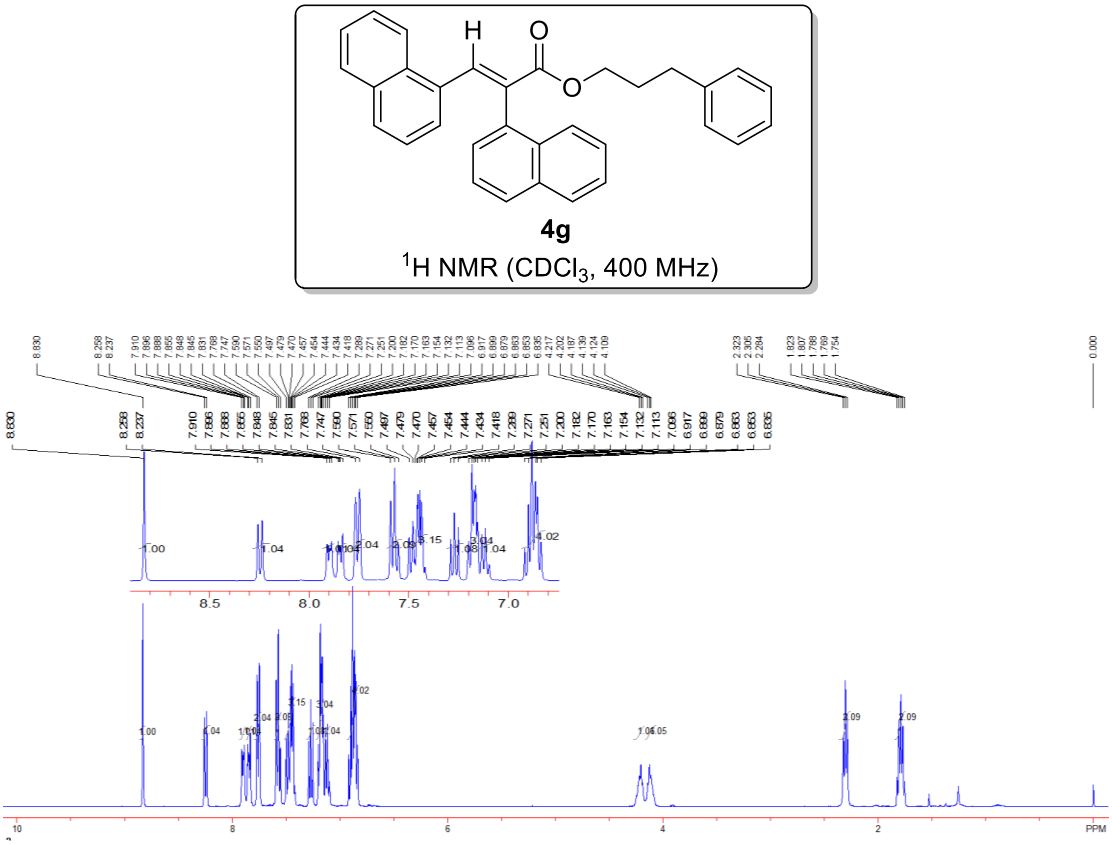

Supplementary Figure 103. ^{1}**H NMR Spectrum of 4g**

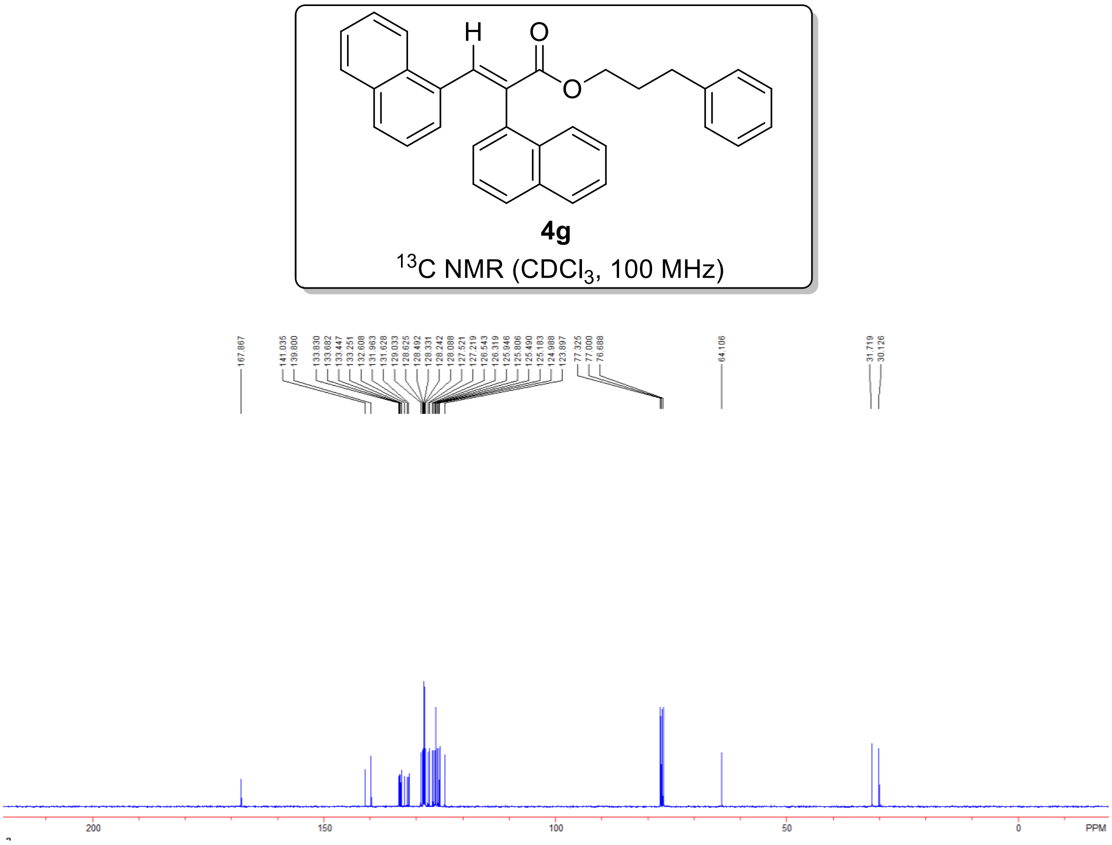

Supplementary Figure 104. ^{13}**C NMR Spectrum of 4g**

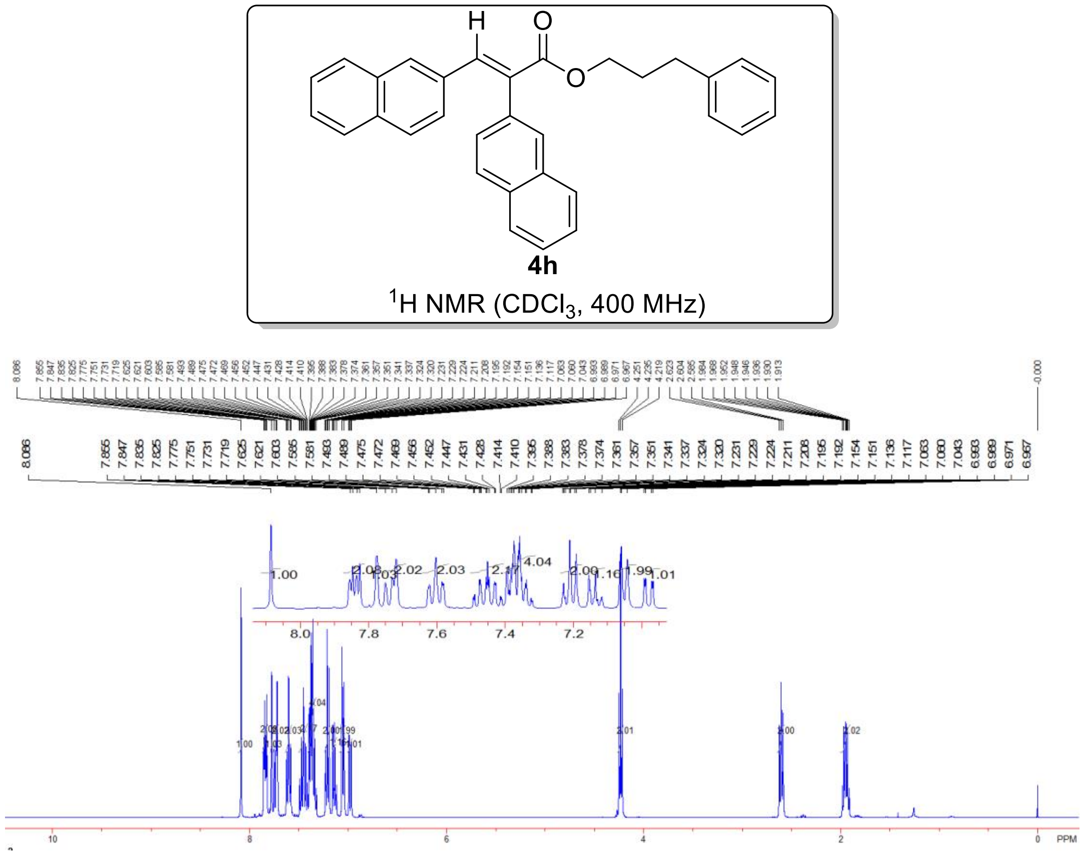

Supplementary Figure 105. ^{1}H NMR Spectrum of 4h

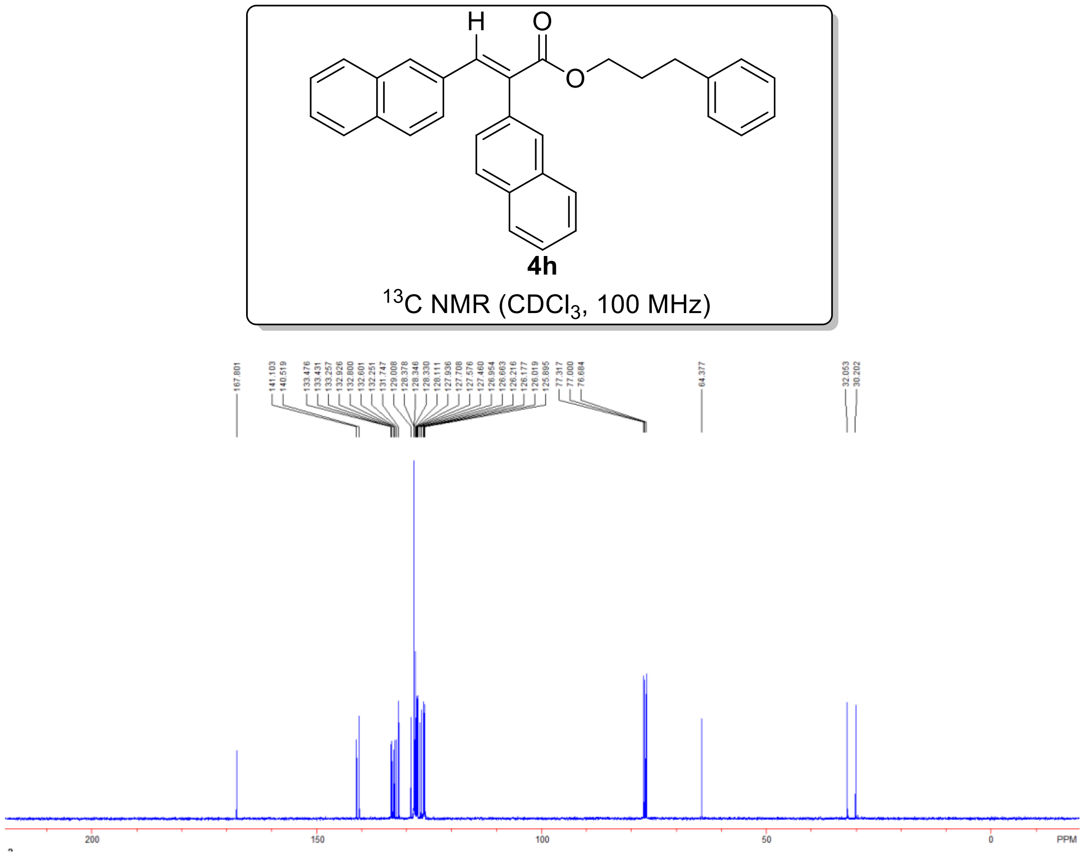

Supplementary Figure 106. ^{13}C NMR Spectrum of 4h

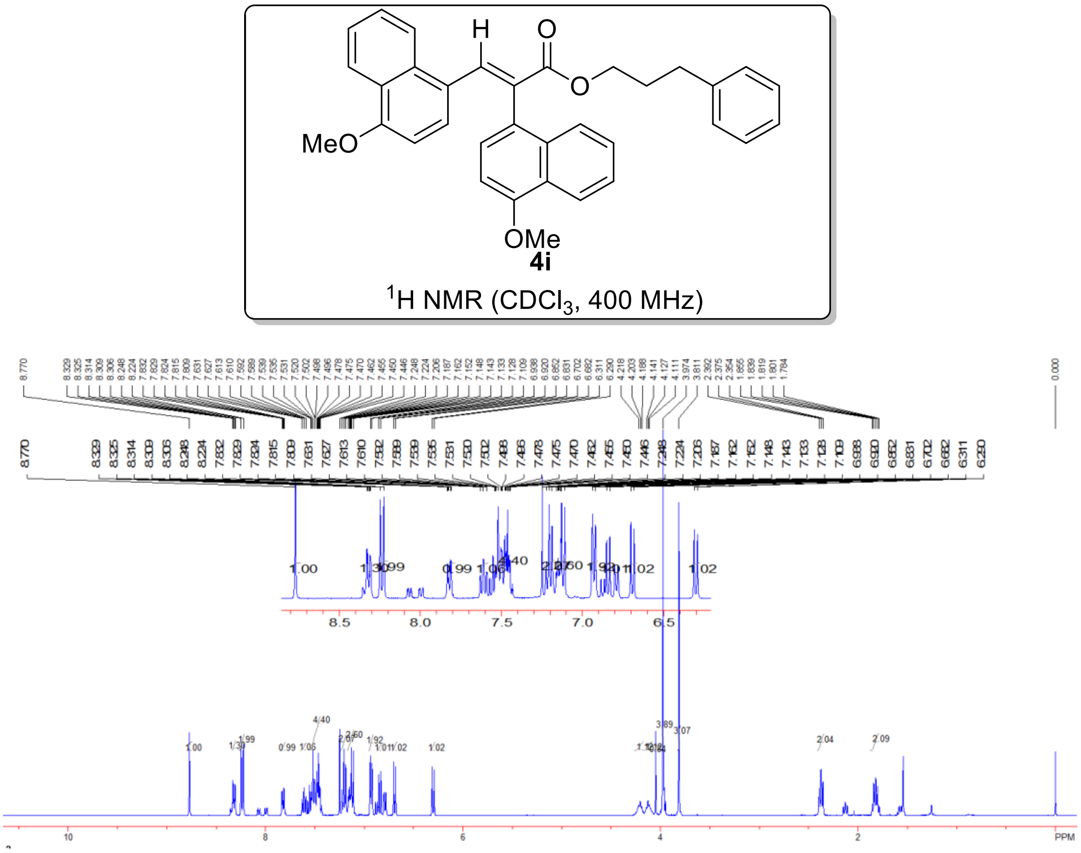

Supplementary Figure 107. ^{1}**H NMR Spectrum of 4i**

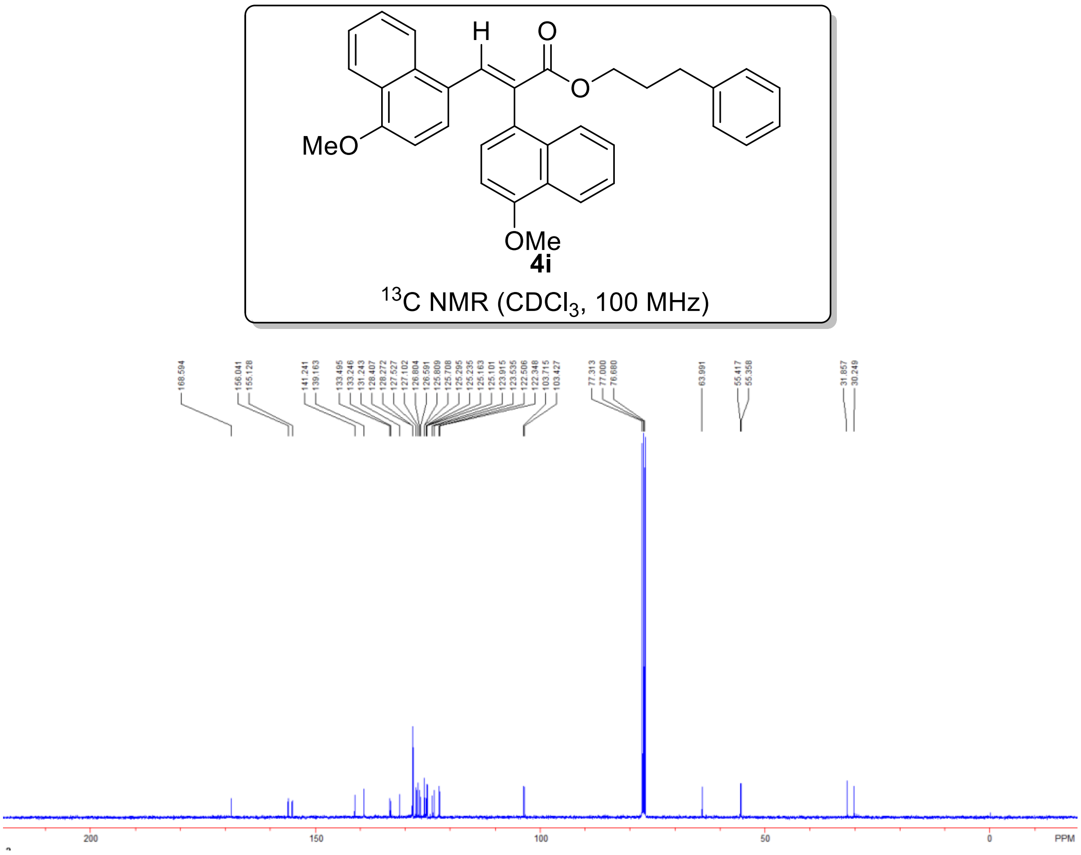

Supplementary Figure 108. ^{13}**C NMR Spectrum of 4i**

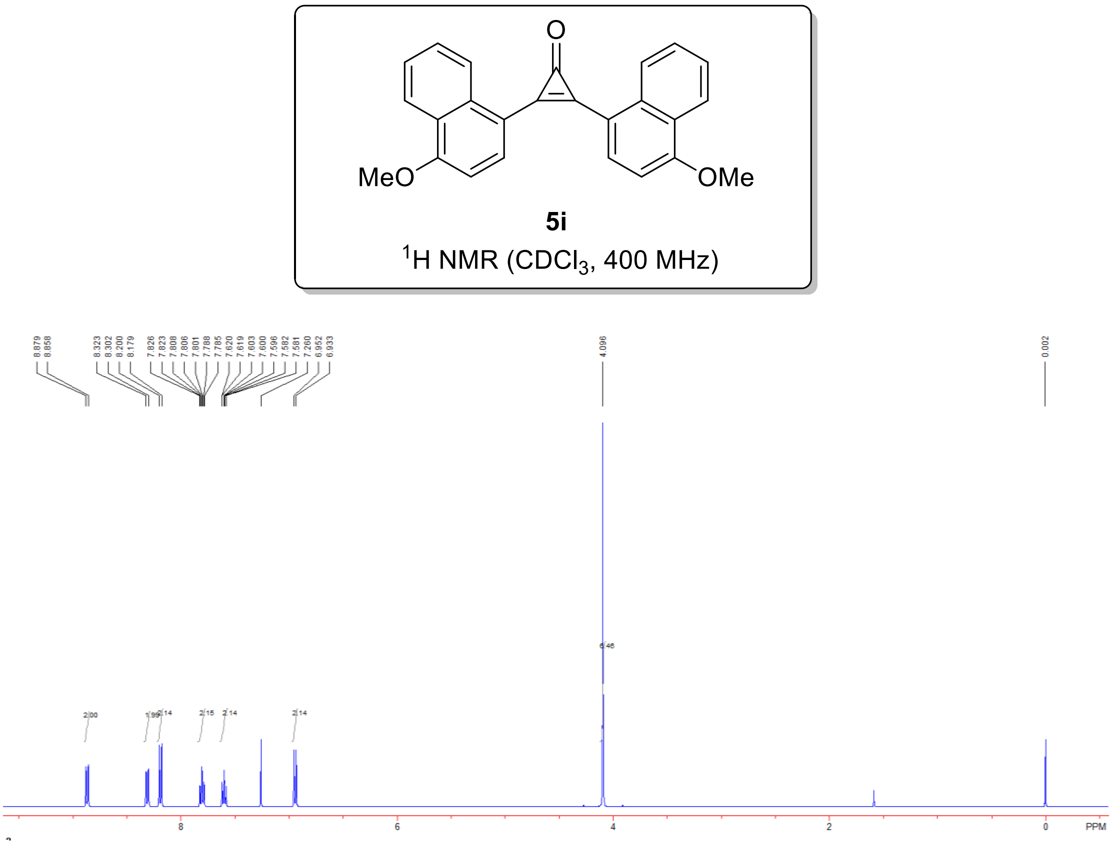

Supplementary Figure 109. ^{1}H NMR Spectrum of 5i

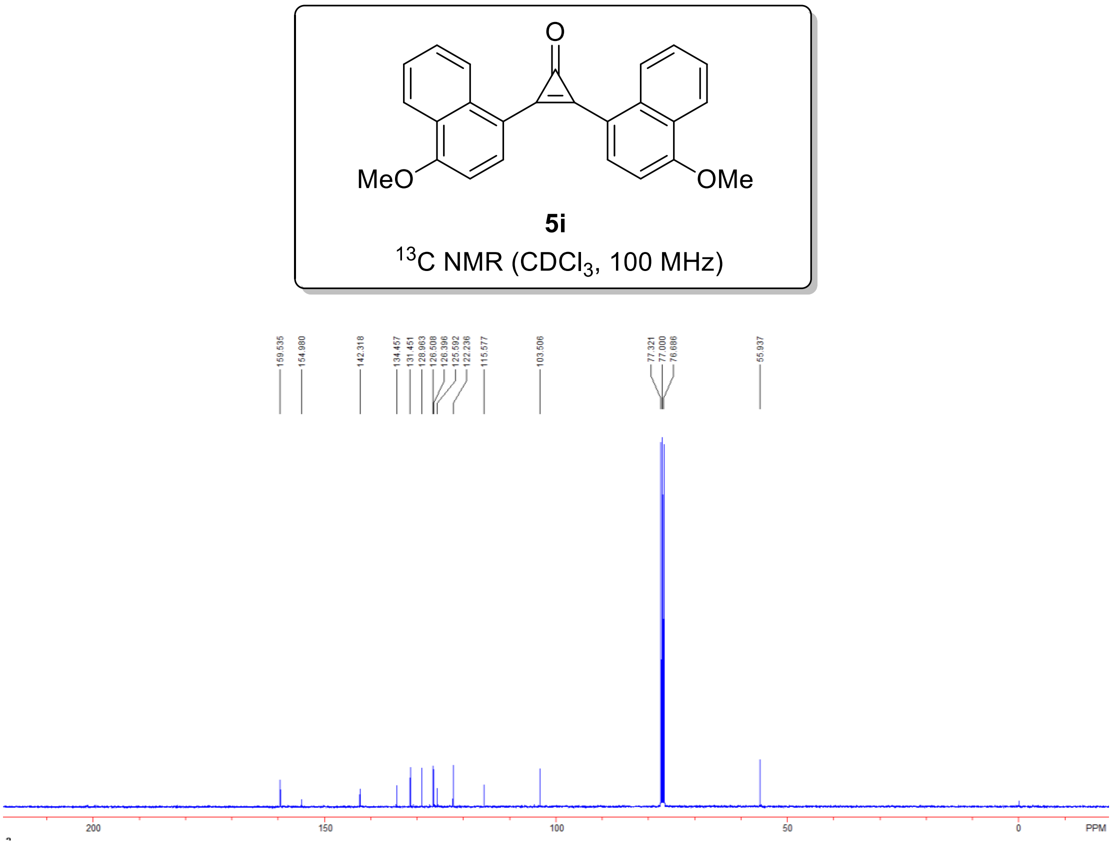

Supplementary Figure 110. ^{13}C NMR Spectrum of 5i

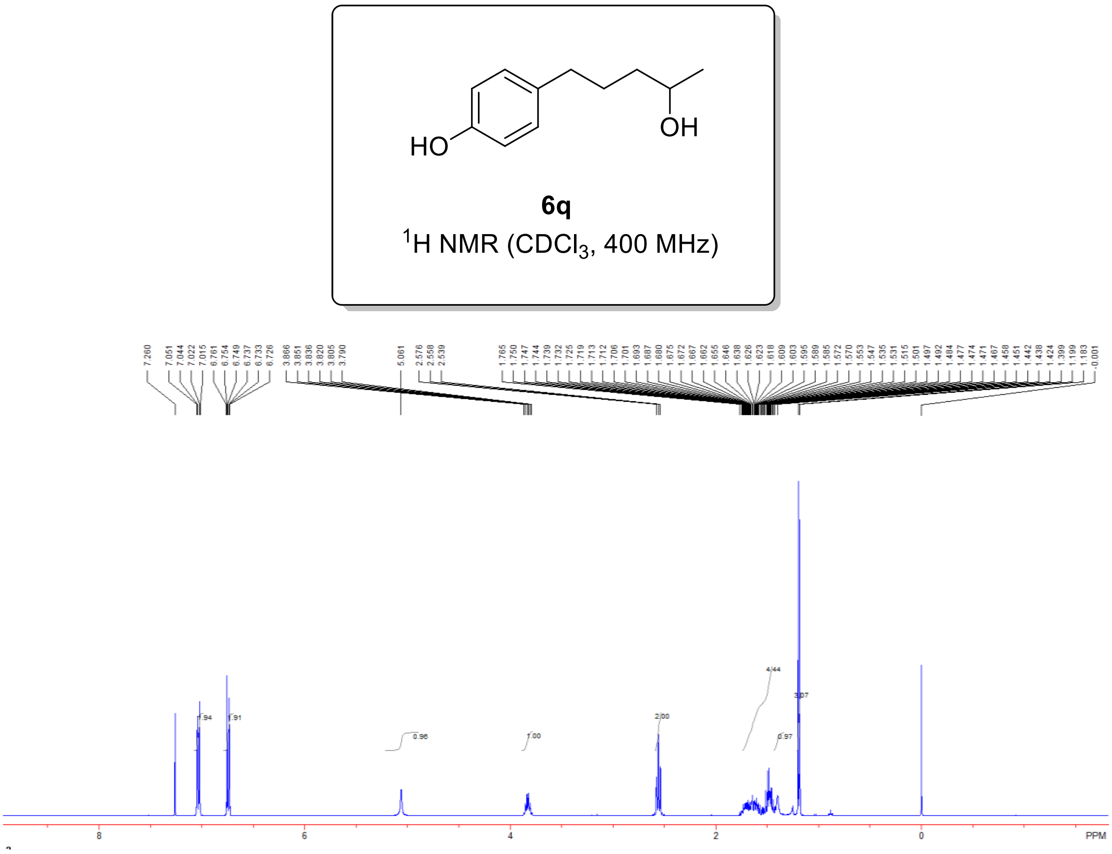

Supplementary Figure 111. ^{1}H NMR Spectrum of 6q

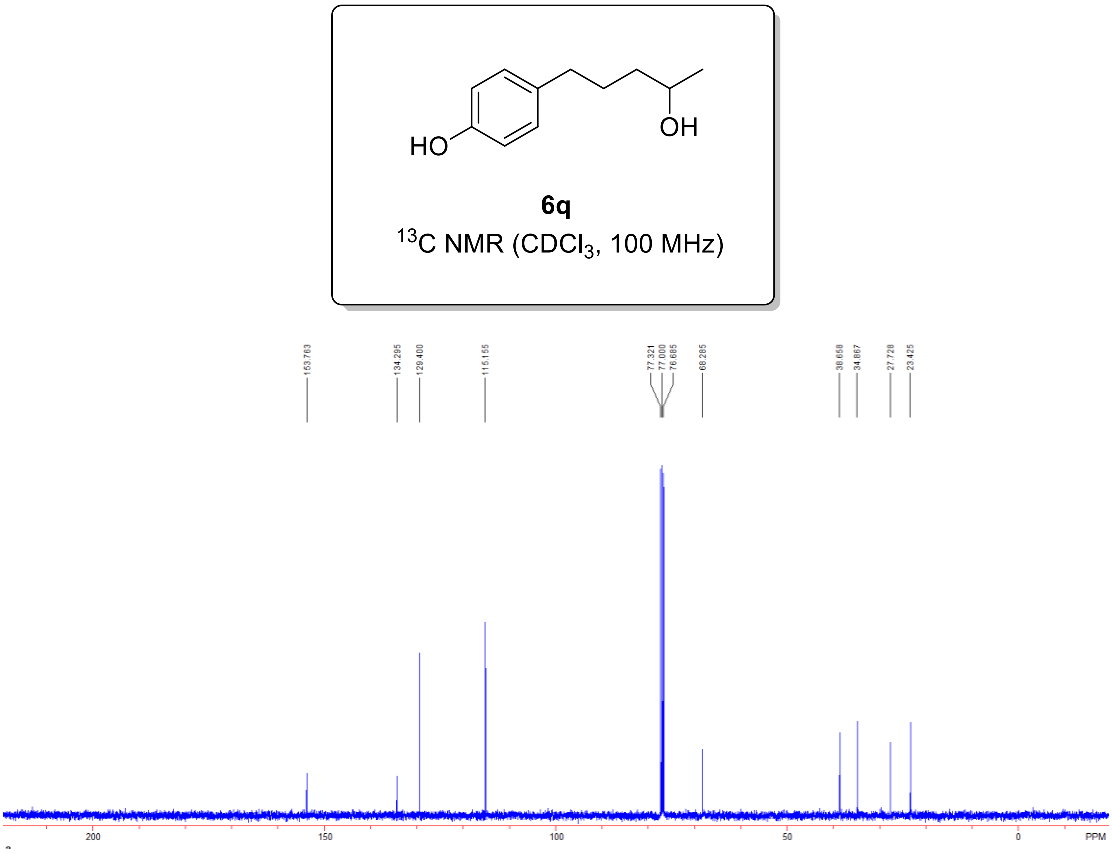

Supplementary Figure 112. ^{13}C NMR Spectrum of 6q

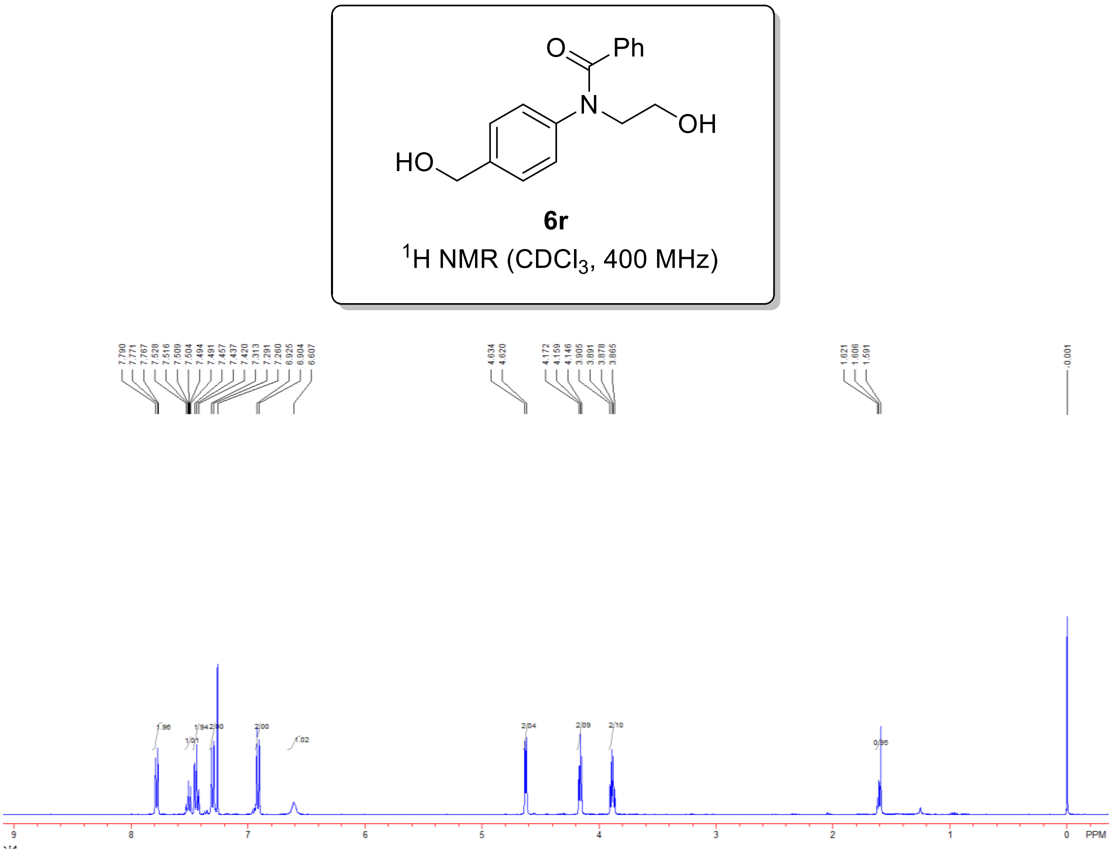

Supplementary Figure 113. ^{1}H NMR Spectrum of 6r

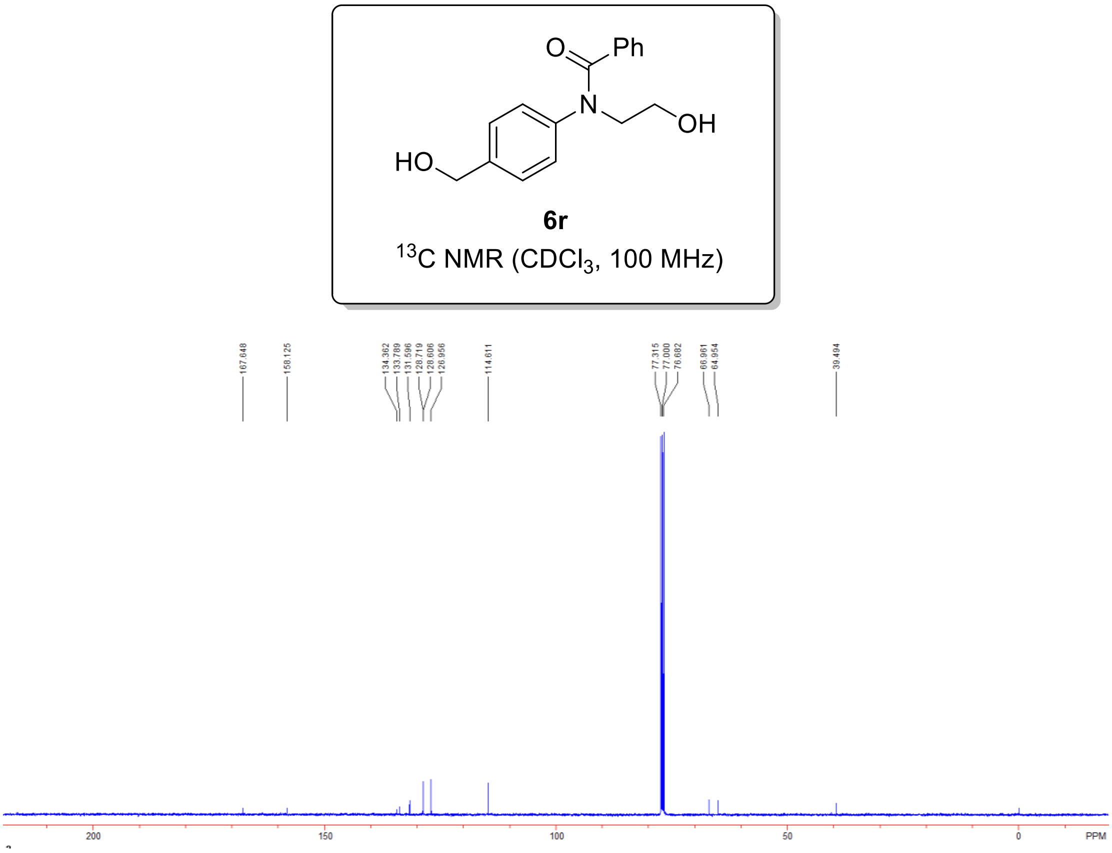

Supplementary Figure 114 ^{13}C NMR Spectrum of 6r

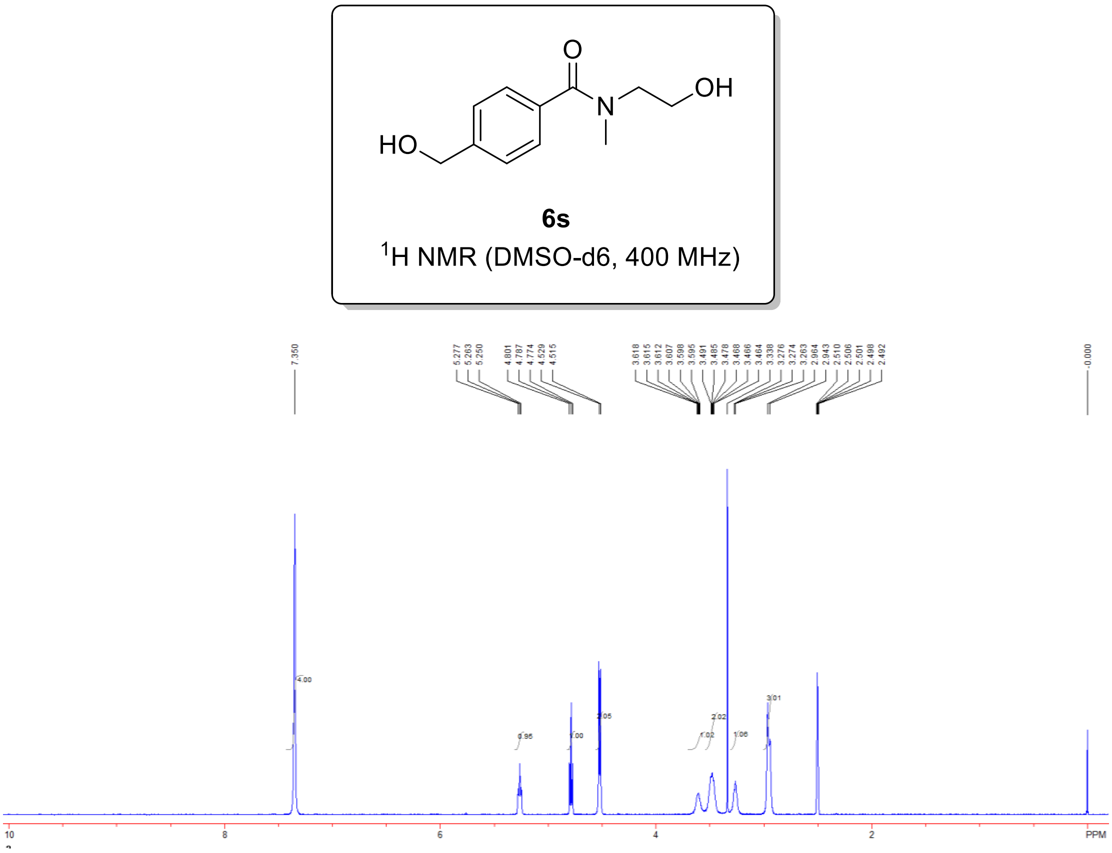

Supplementary Figure 115. ^{1}H NMR Spectrum of 6s

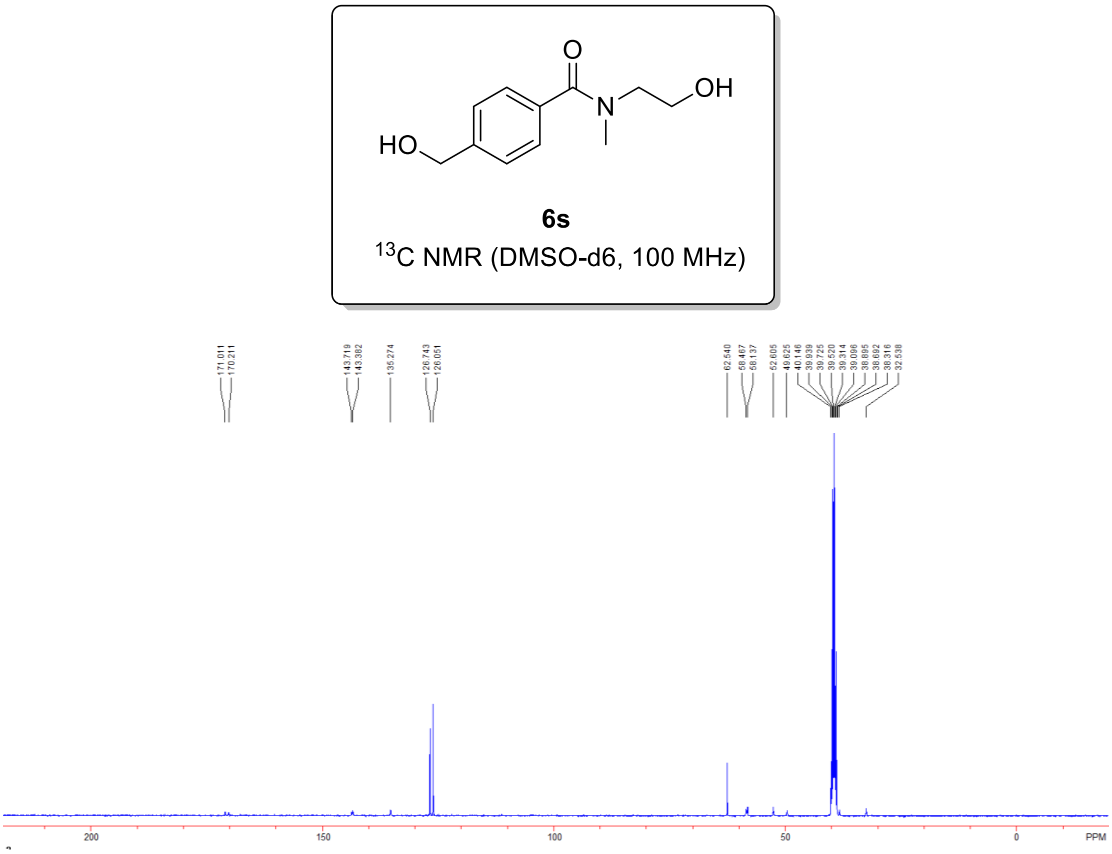

Supplementary Figure 116. ^{13}C NMR Spectrum of 6s

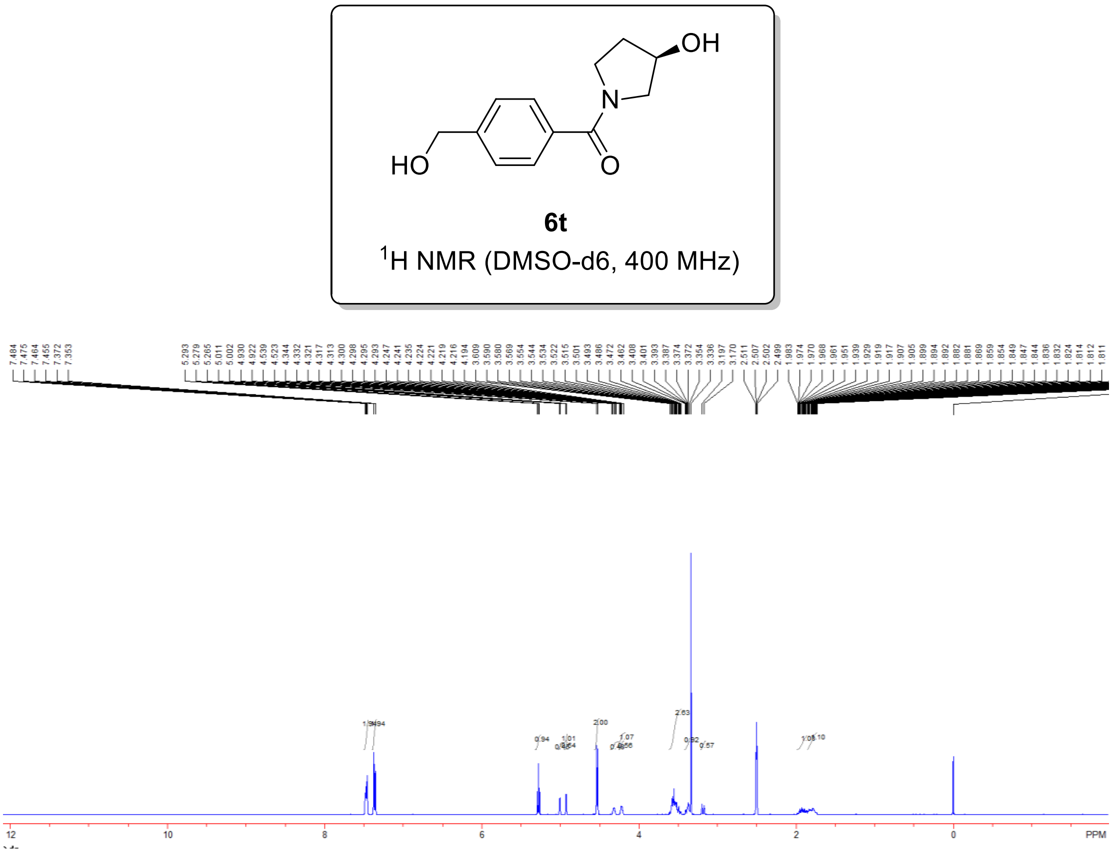

Supplementary Figure 117. ^{1}**H NMR Spectrum of 6t**

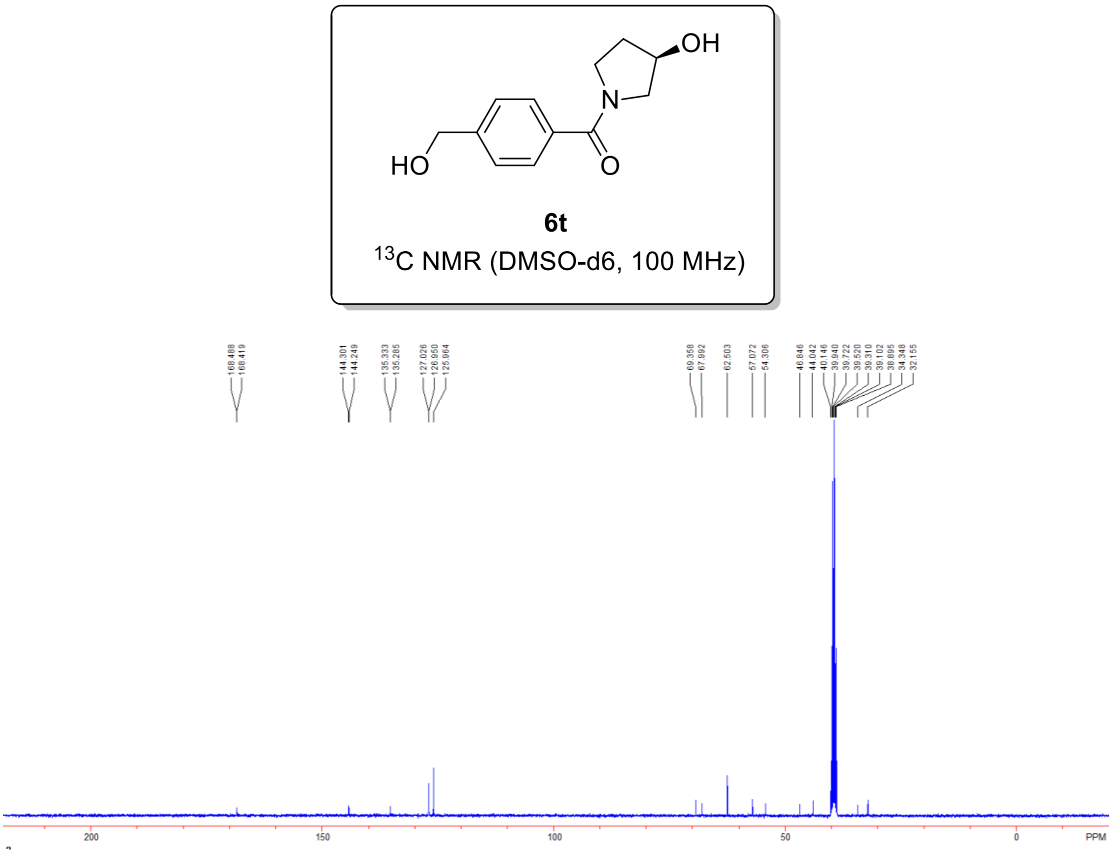

Supplementary Figure 118. ^{13}**C NMR Spectrum of 6t**

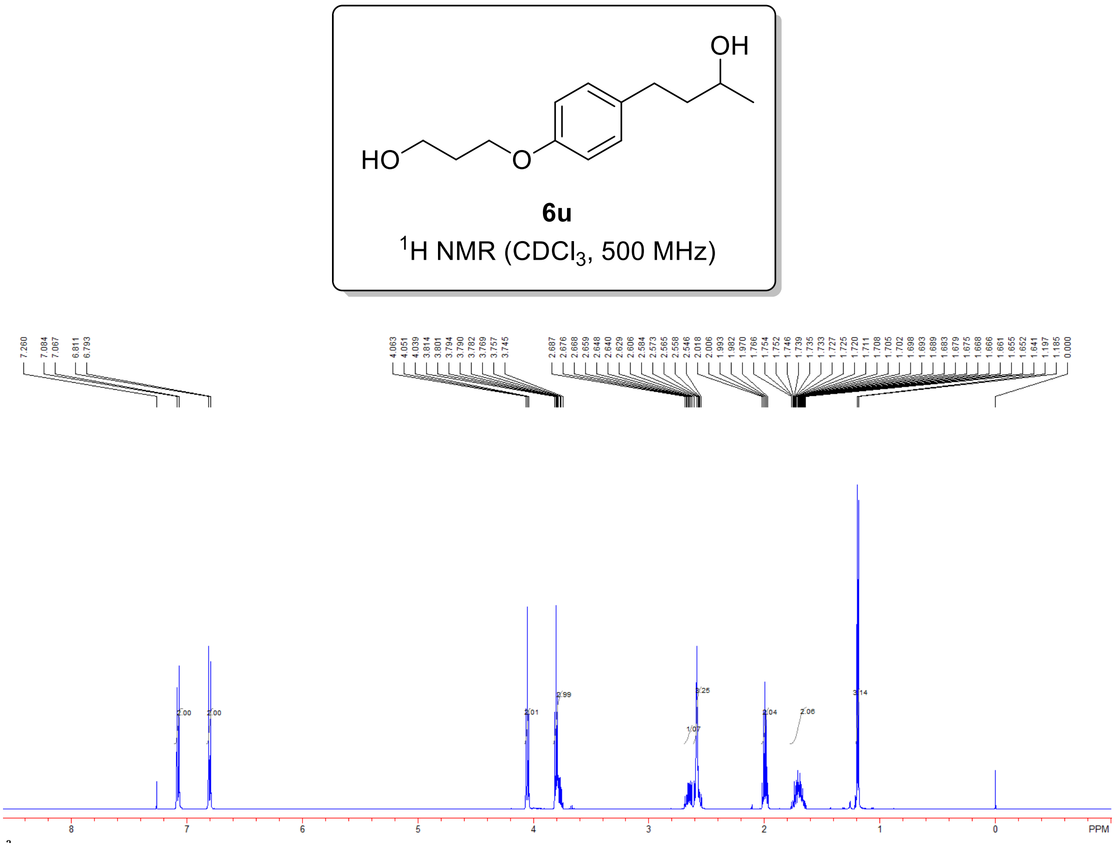

Supplementary Figure 119. ^{1}H NMR Spectrum of 6u

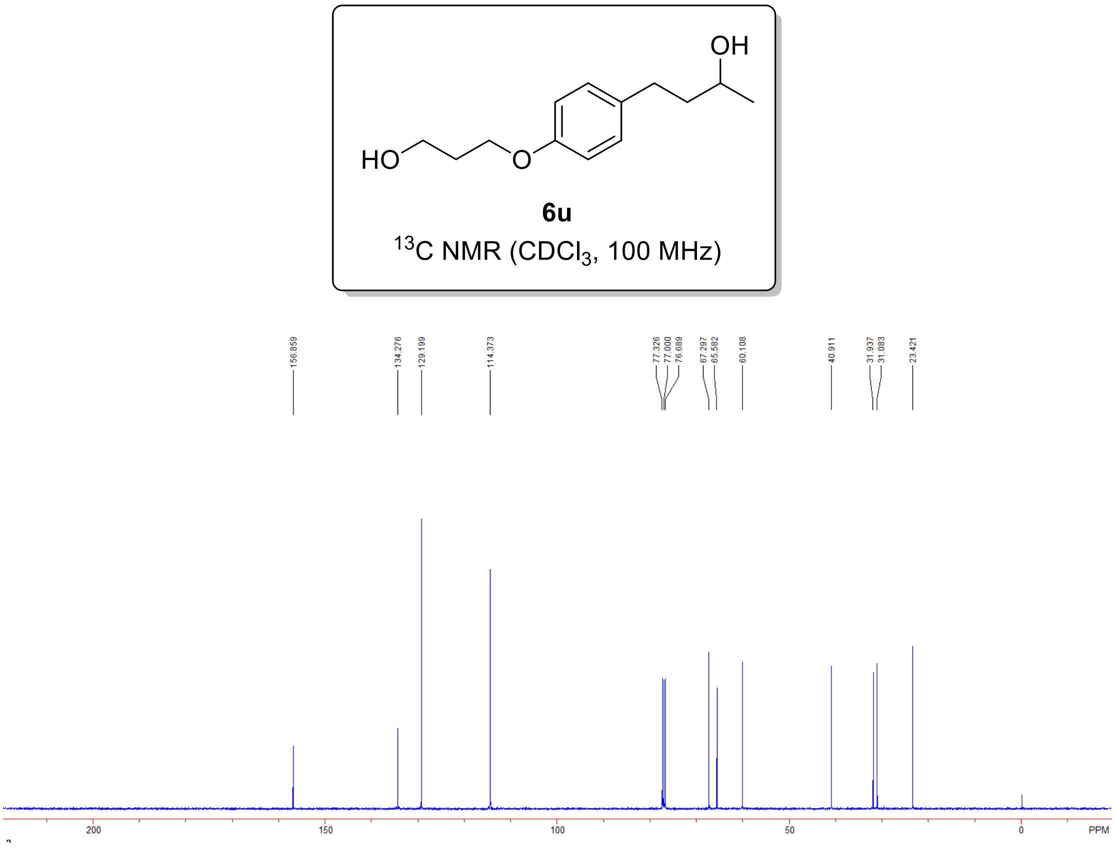

Supplementary Figure 120. ^{13}C NMR Spectrum of 6u

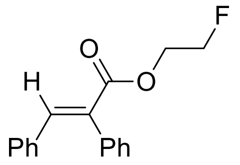

7a

^{1}H NMR ($CDCl_3$, 400 MHz)

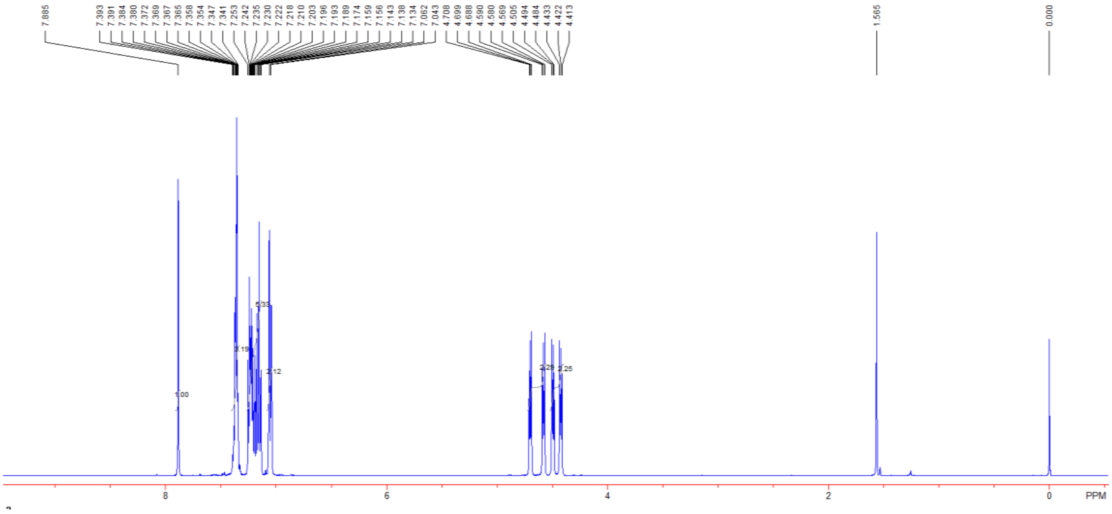

Supplementary Figure 121. ^{1}H NMR Spectrum of 7a

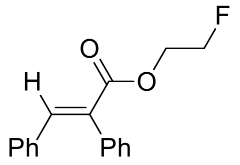

7a

^{19}F NMR ($CDCl_3$, 376 MHz)

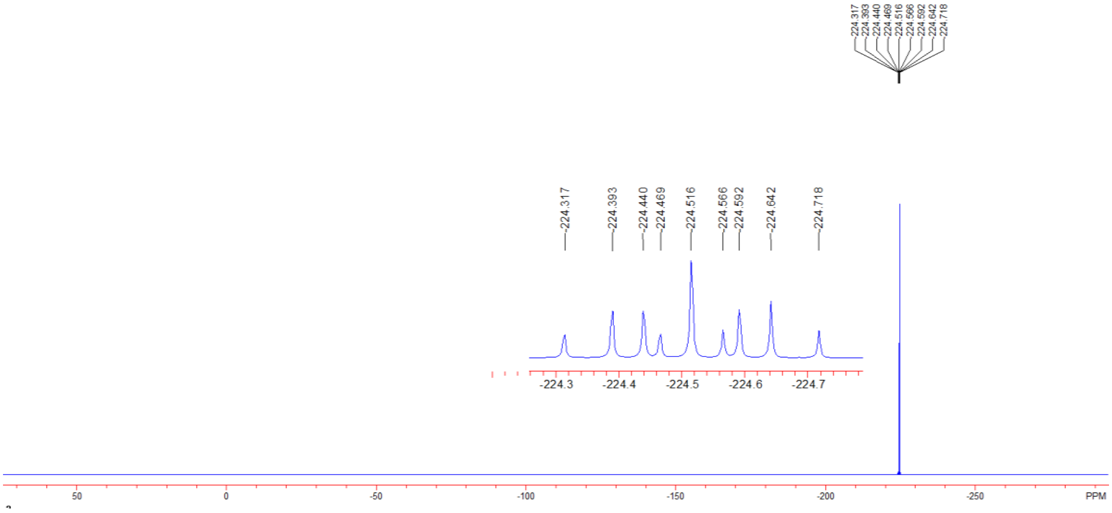

Supplementary Figure 122. ^{19}F NMR Spectrum of 7a

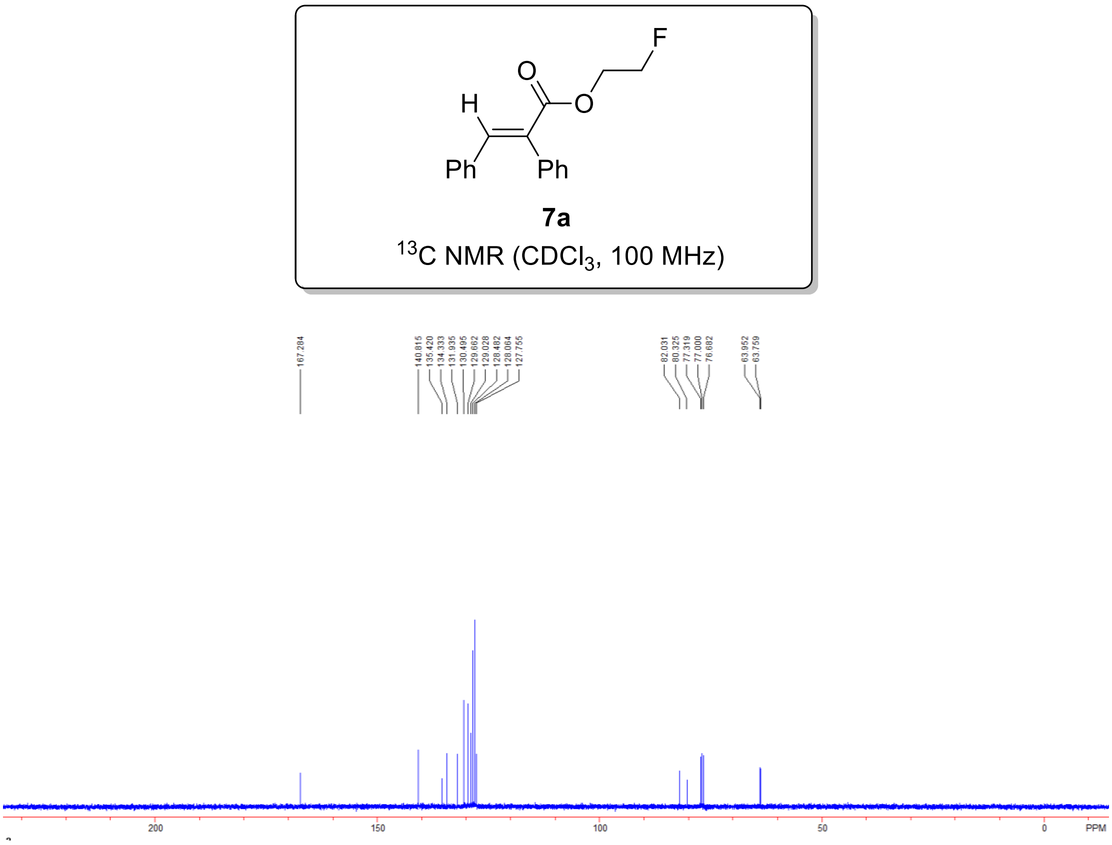

Supplementary Figure 123. ^{13}C NMR Spectrum of 7a

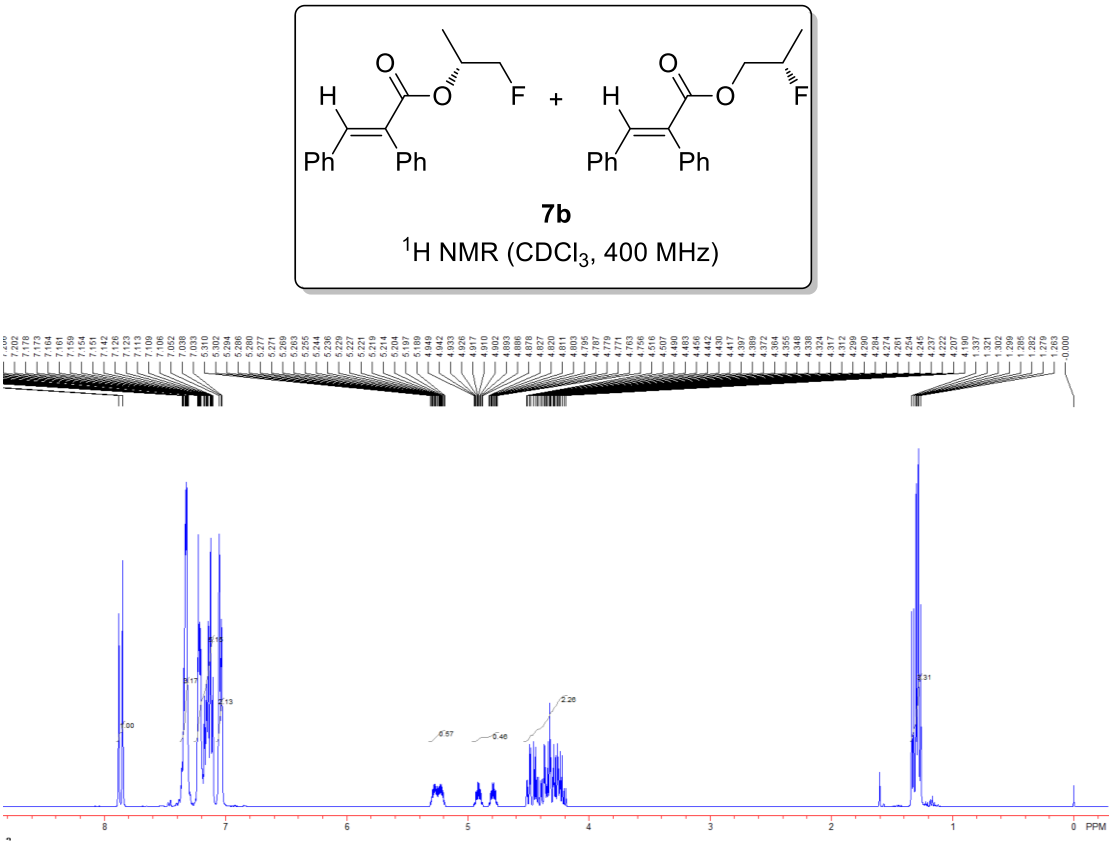

Supplementary Figure 124. ^{1}H NMR Spectrum of 7b

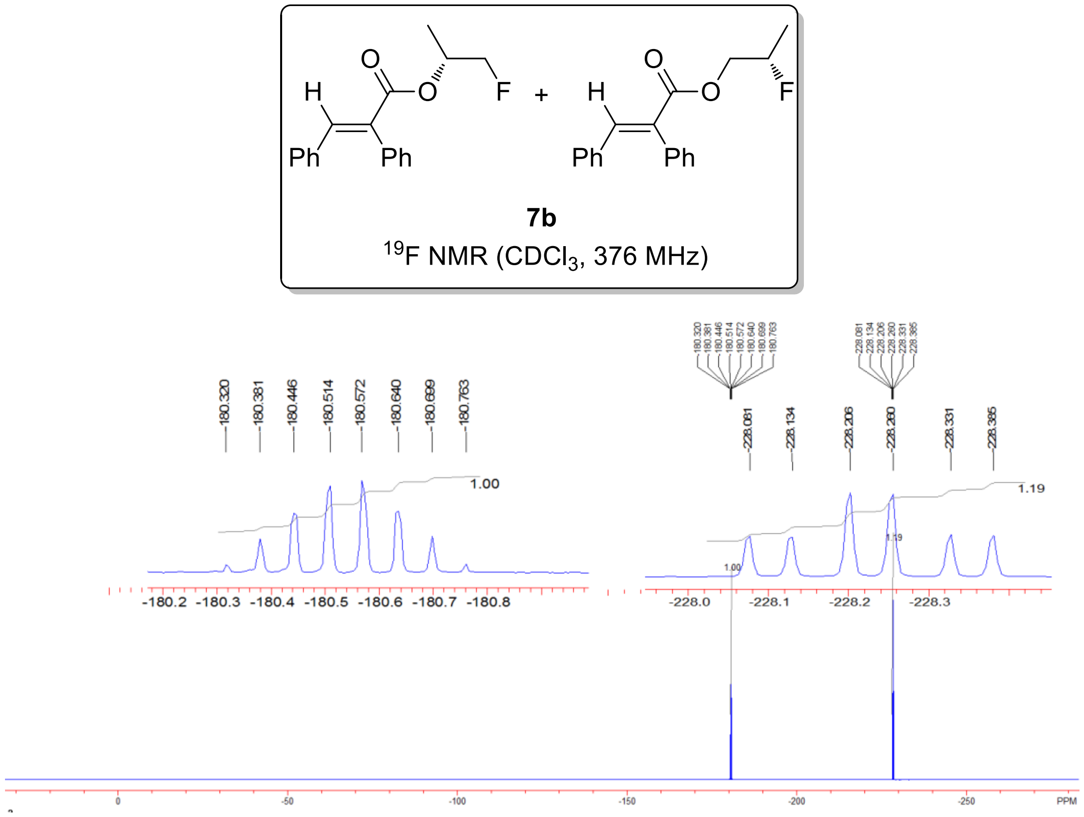

Supplementary Figure 125. ^{19}**F NMR Spectrum of 7b**

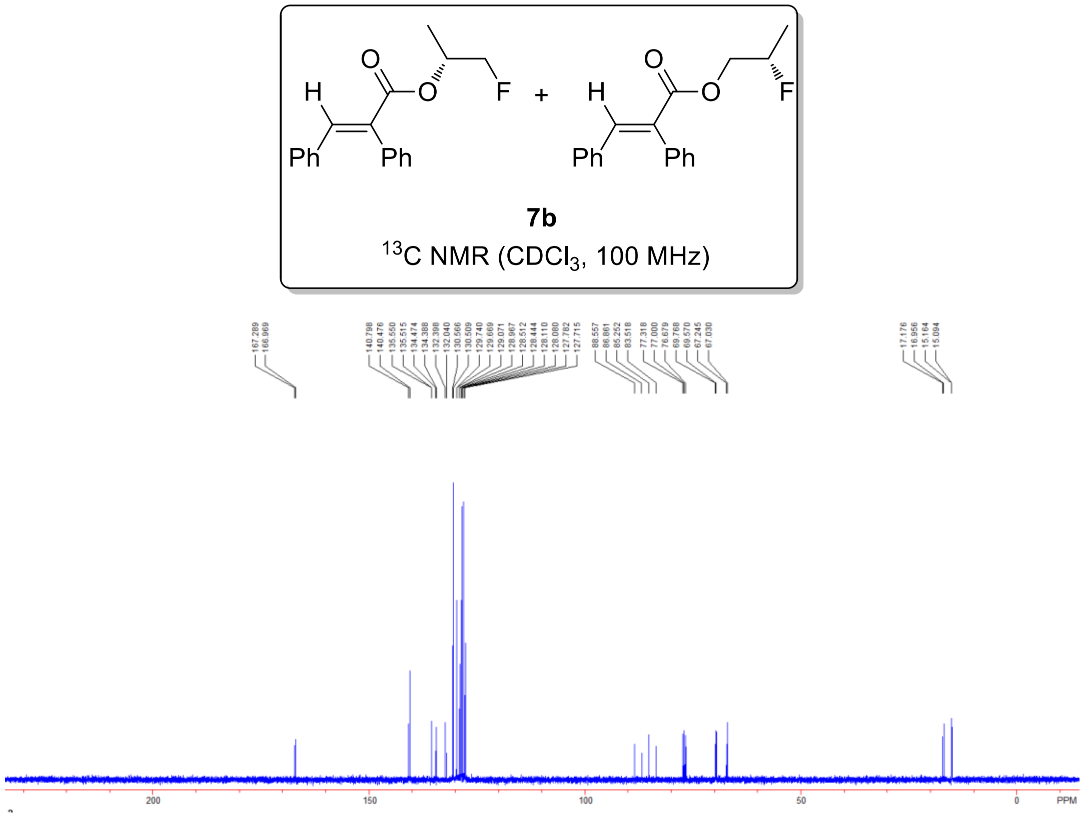

Supplementary Figure 126. ^{13}**C NMR Spectrum of 7b**

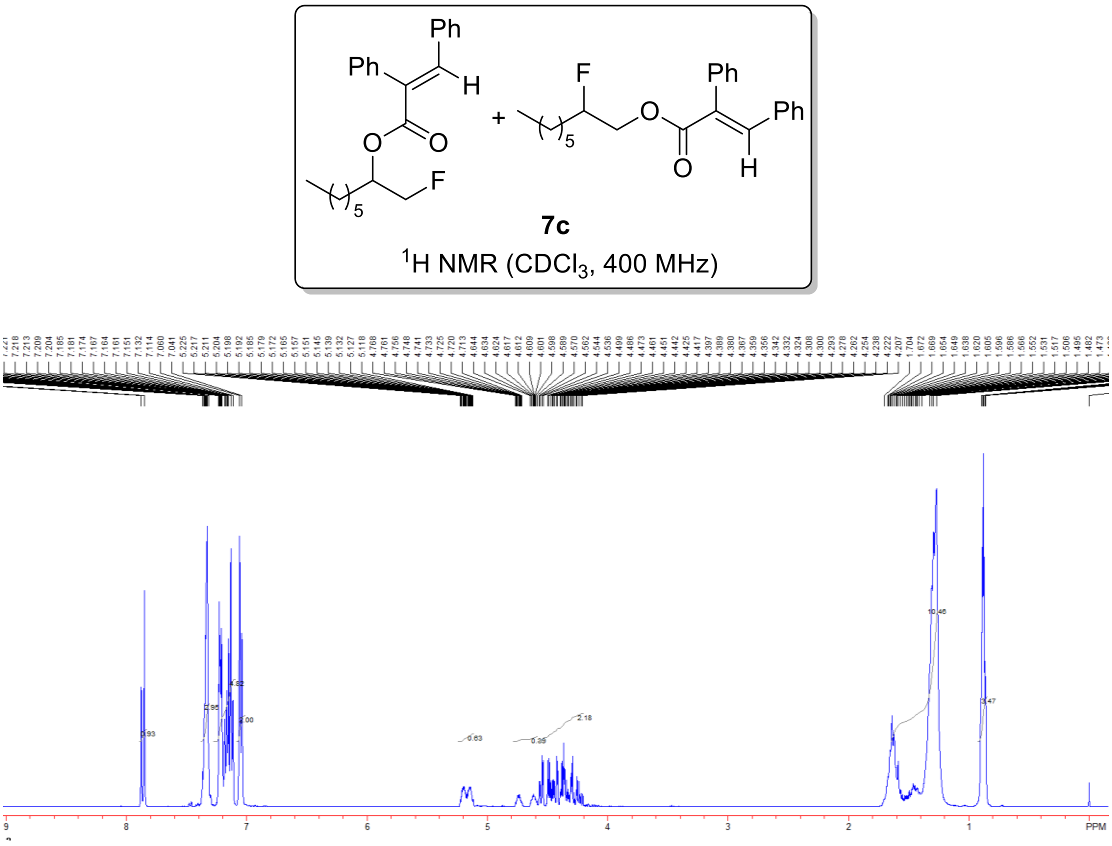

Supplementary Figure 127. ^{1}H NMR Spectrum of 7c

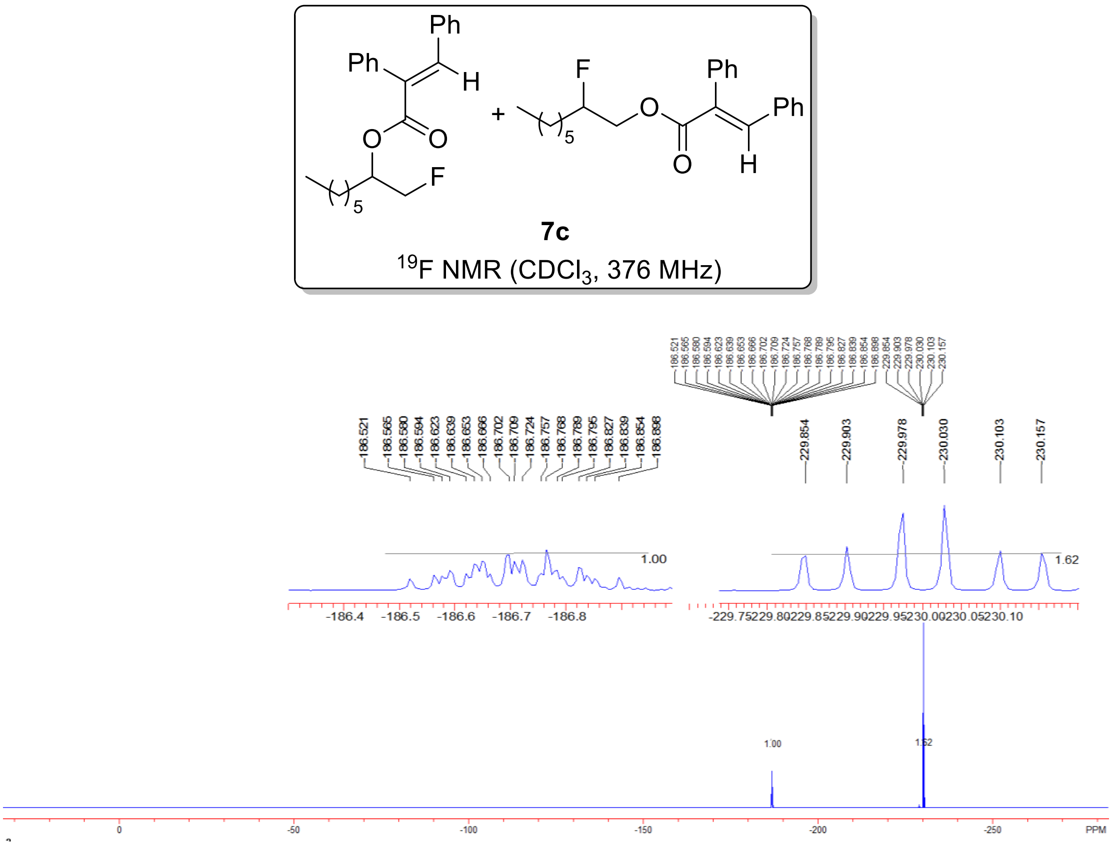

Supplementary Figure 128. ^{19}F NMR Spectrum of 7c

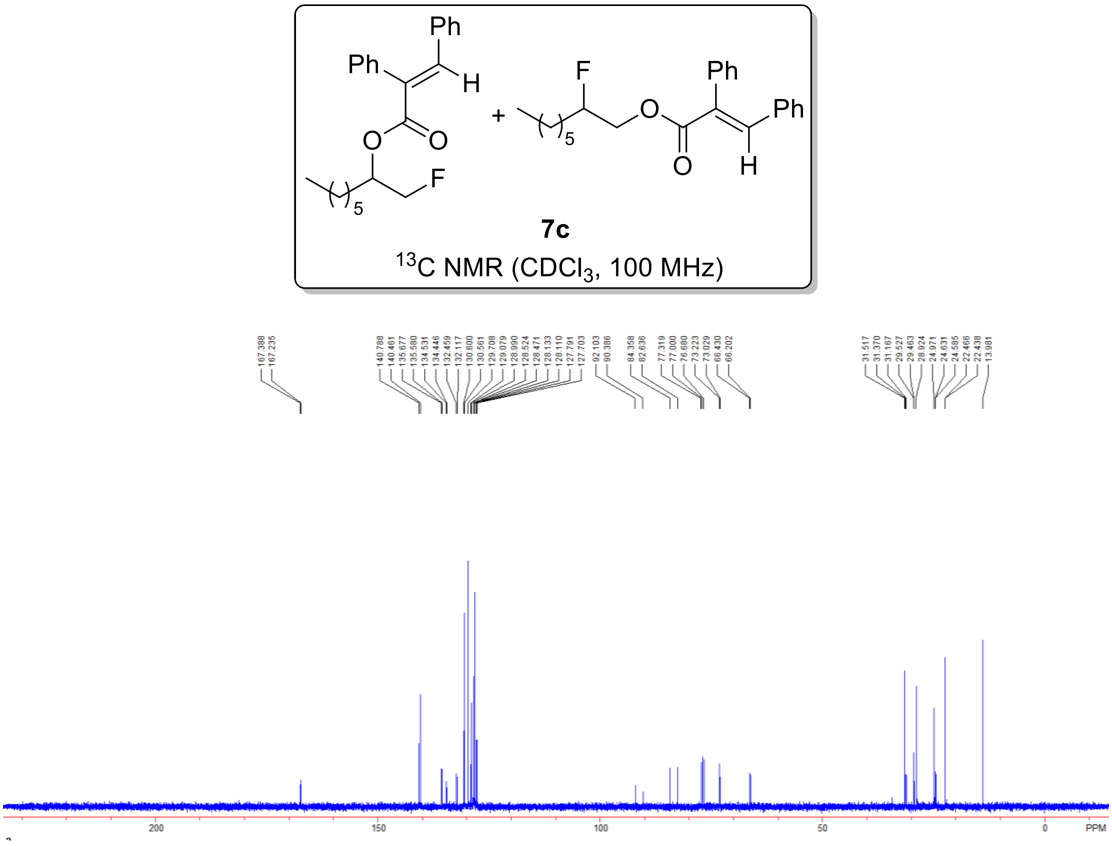

Supplementary Figure 129. ^{13}C NMR Spectrum of 7c

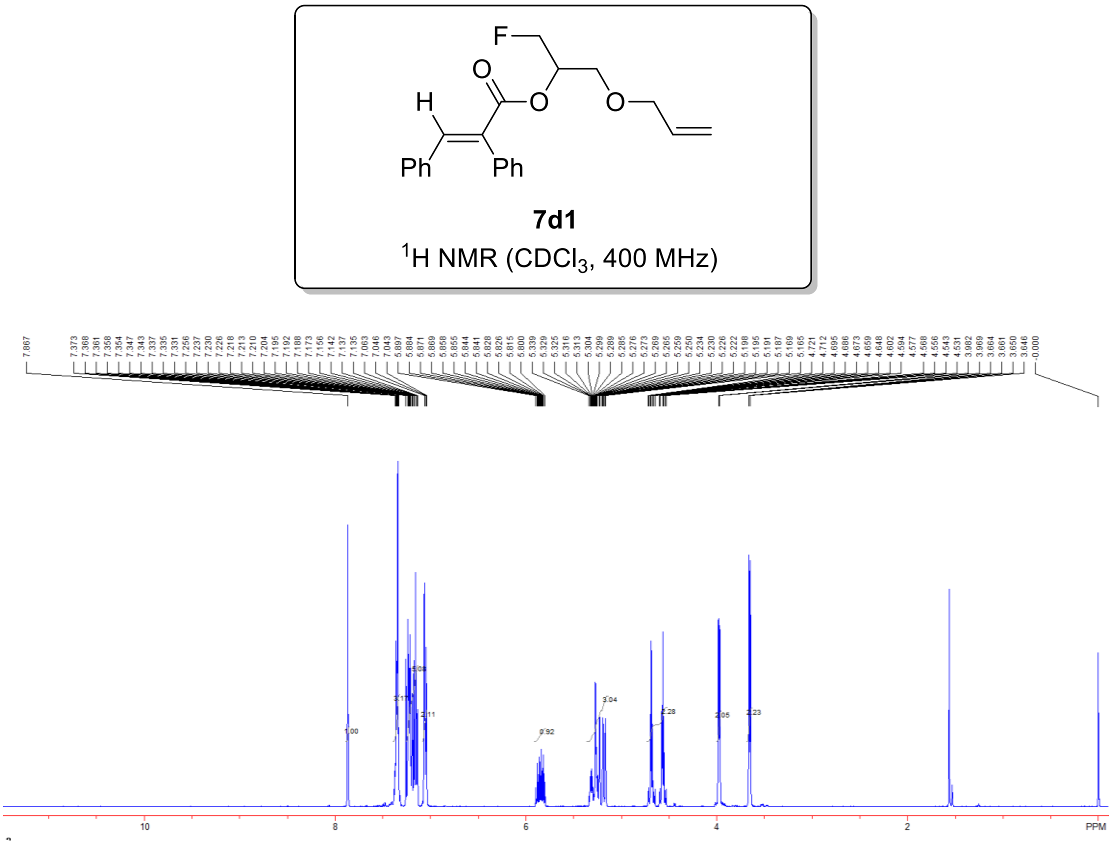

Supplementary Figure 130. ^{1}H NMR Spectrum of 7d1

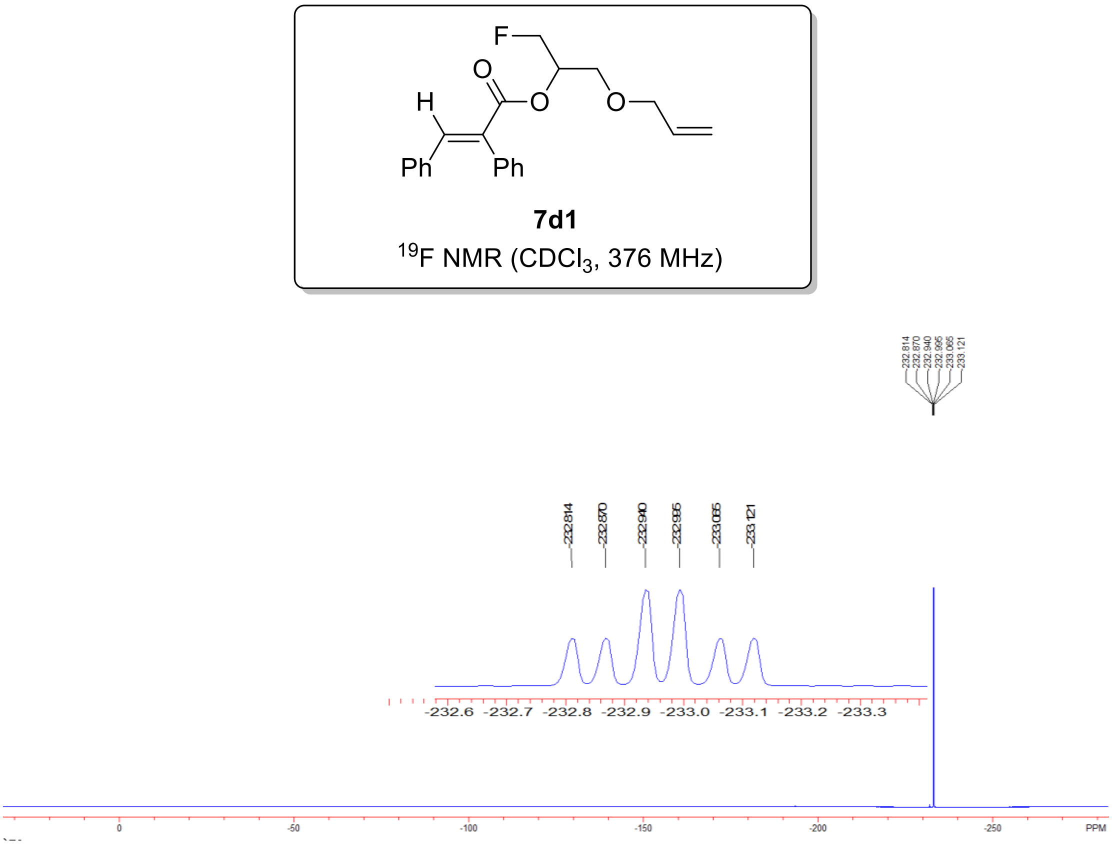

Supplementary Figure 131. ^{19}**F NMR Spectrum of 7d1**

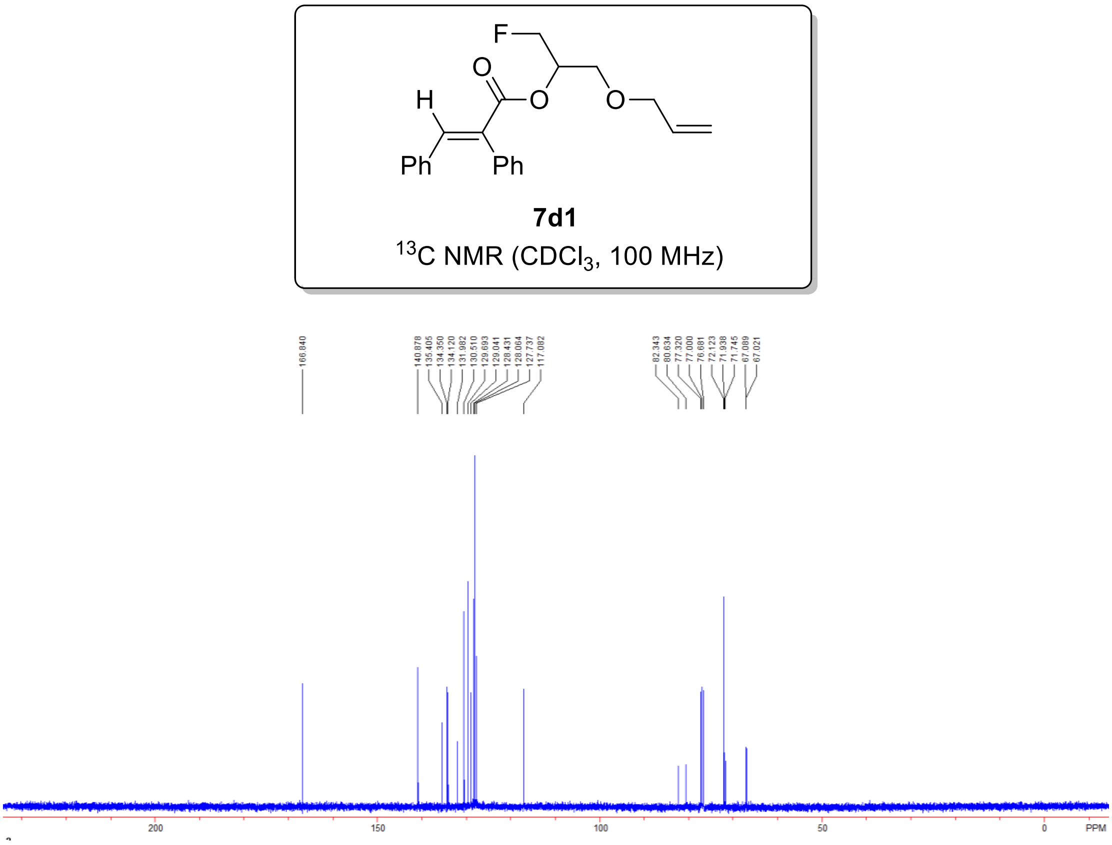

Supplementary Figure 132. ^{13}**C NMR Spectrum of 7d1**

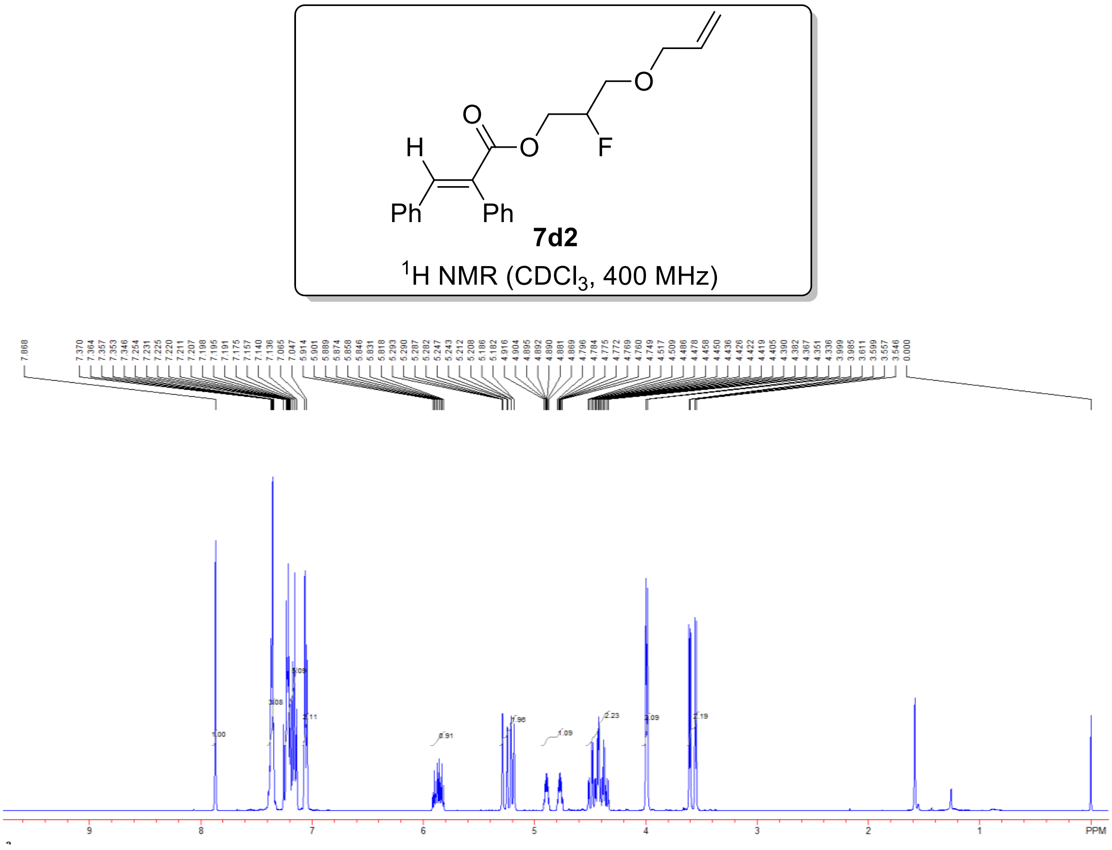

Supplementary Figure 133. ^{1}**H NMR Spectrum of 7d2**

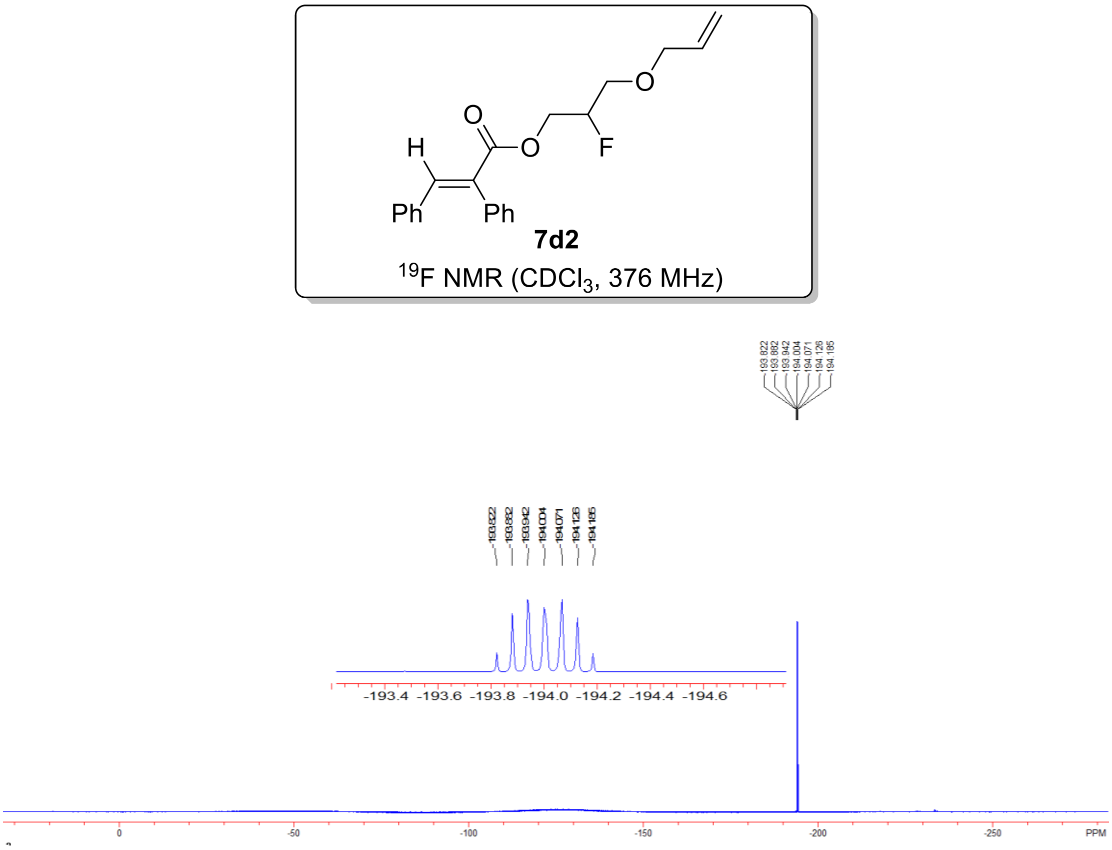

Supplementary Figure 134. ^{19}**F NMR Spectrum of 7d2**

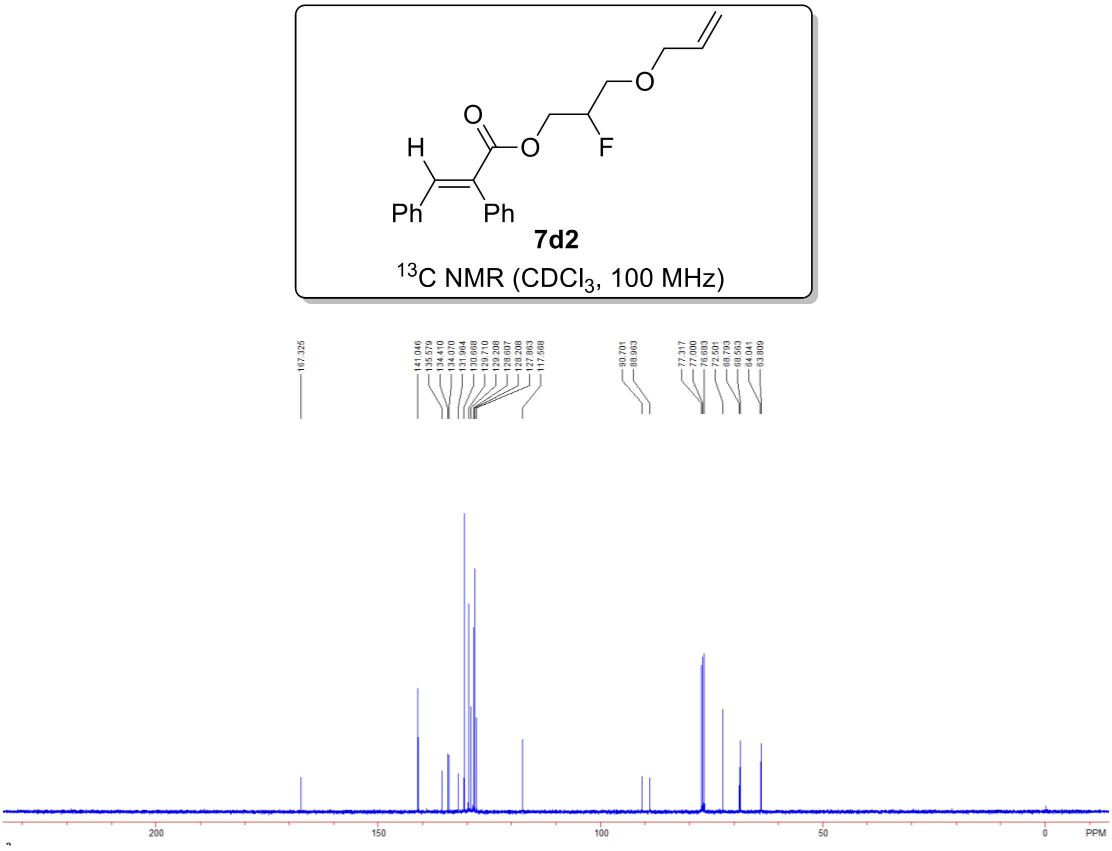

Supplementary Figure 135. ^{13}C NMR Spectrum of 7d2

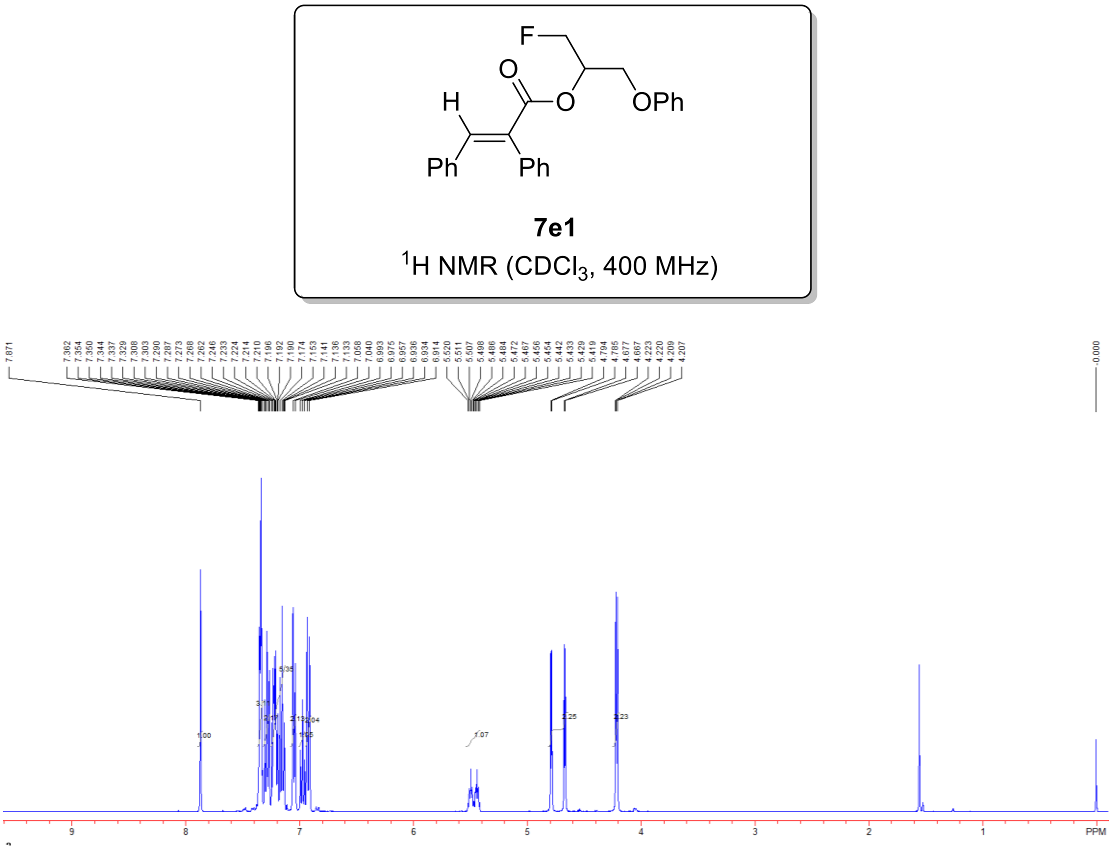

Supplementary Figure 136. ^{1}H NMR Spectrum of 7e1

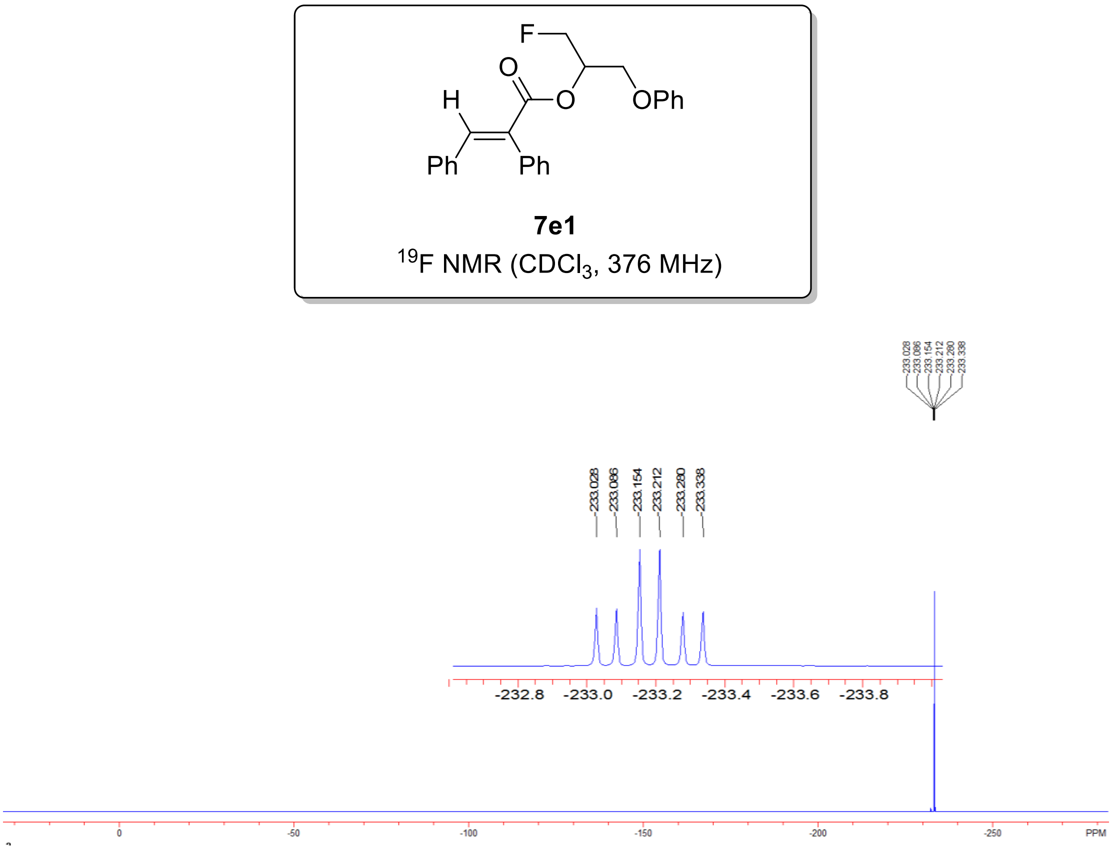

Supplementary Figure 137. ^{19}F NMR Spectrum of 7e1

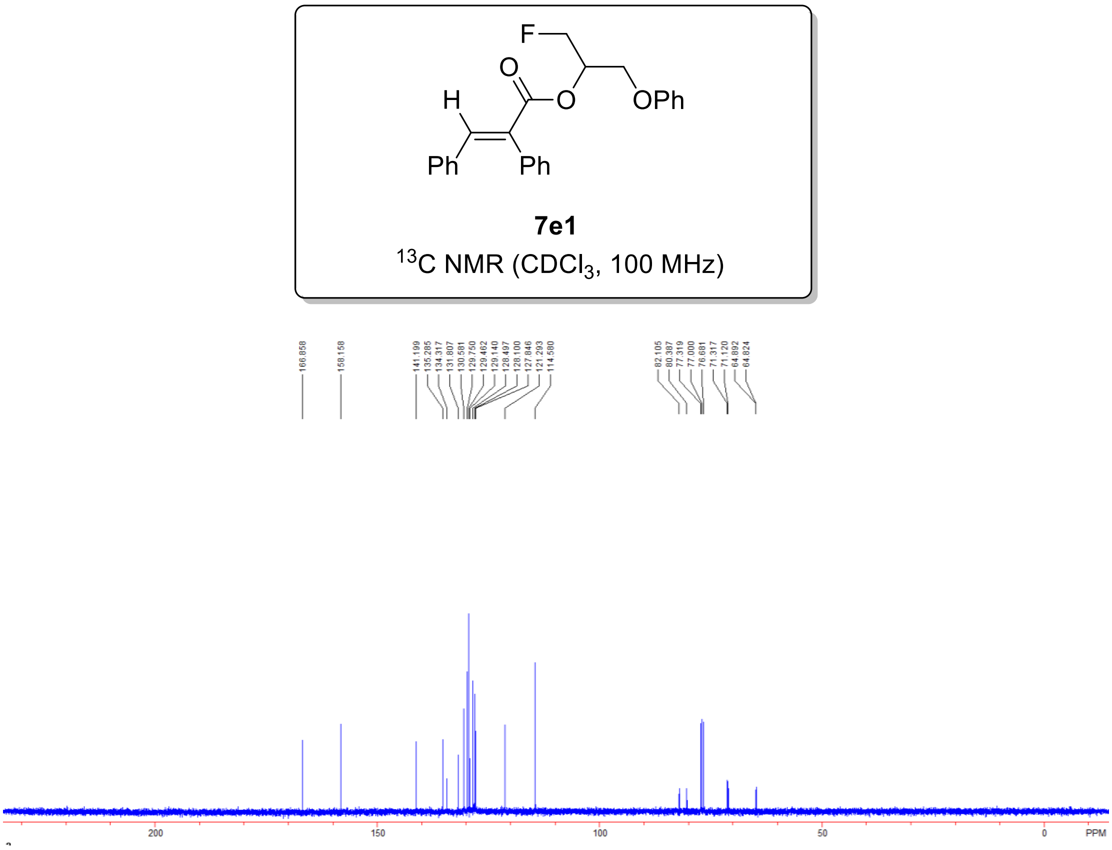

Supplementary Figure 138. ^{13}C NMR Spectrum of 7e1

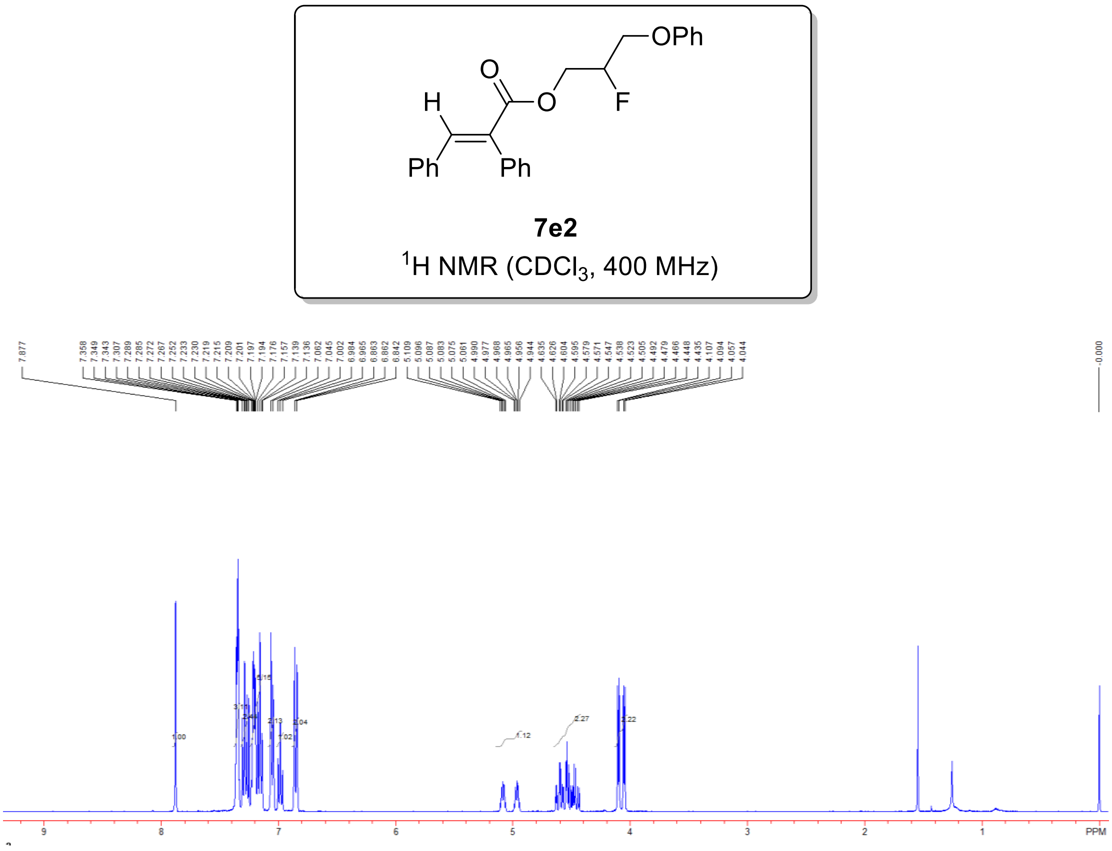

Supplementary Figure 139. ^{1}H NMR Spectrum of 7e2

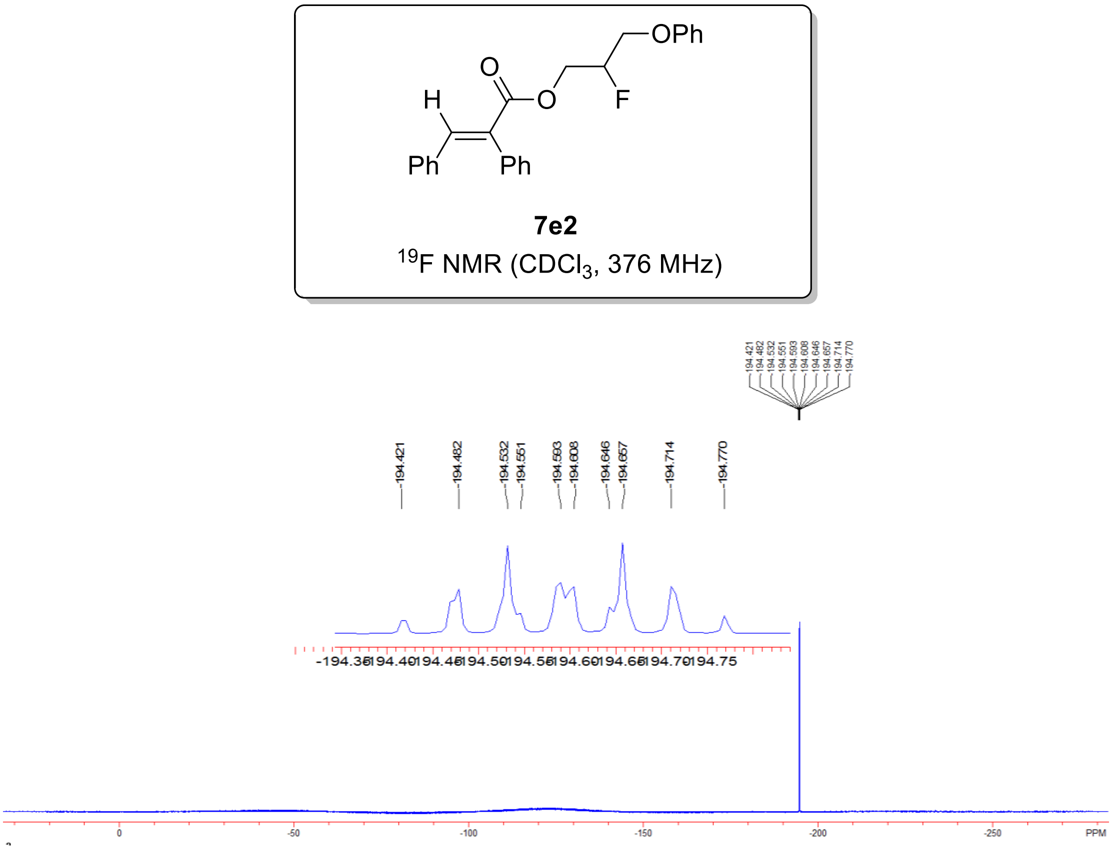

Supplementary Figure 140. ^{19}F NMR Spectrum of 7e2

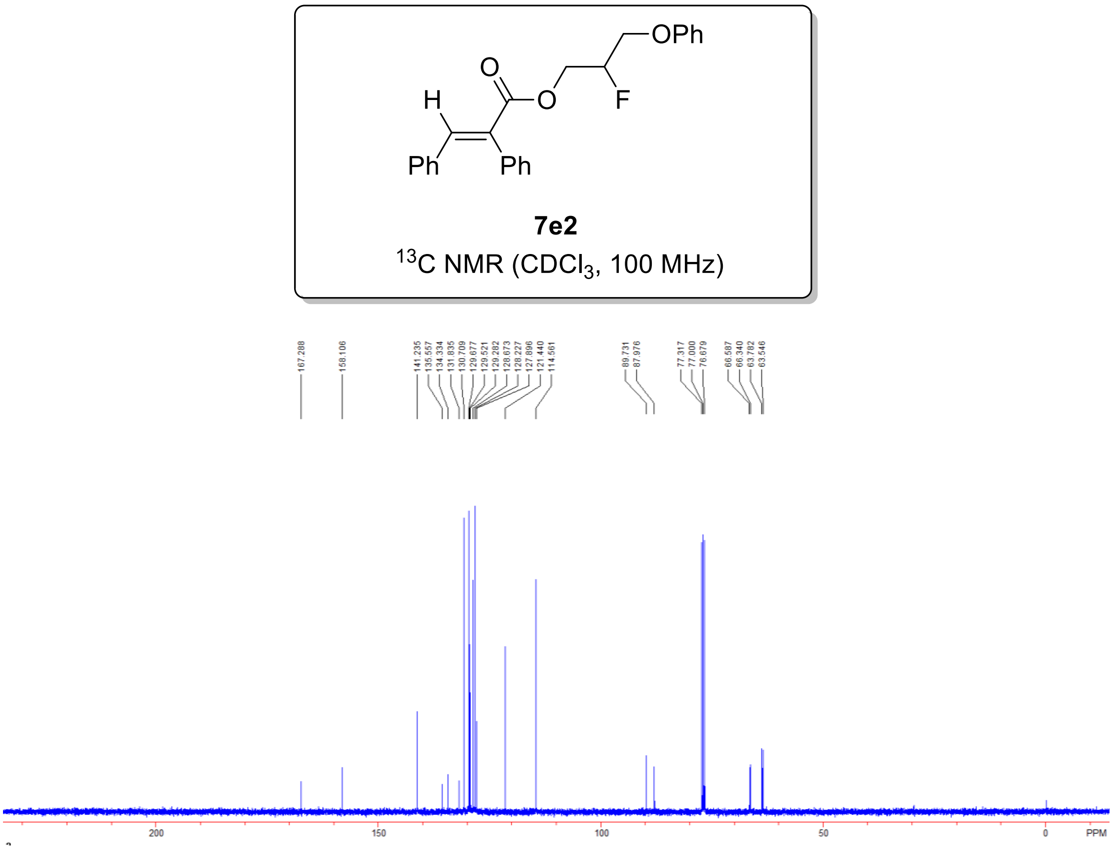

Supplementary Figure 141. ^{13}C NMR Spectrum of 7e2

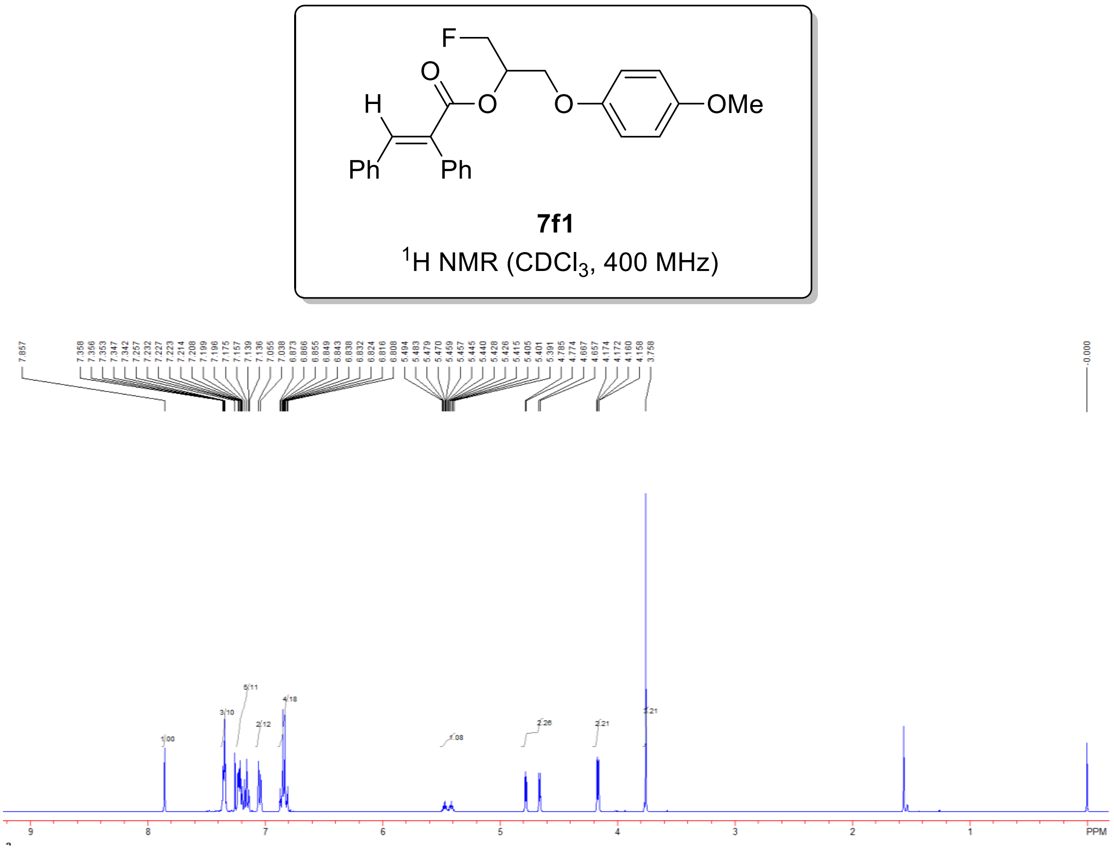

Supplementary Figure 142. ^{1}H NMR Spectrum of 7f1

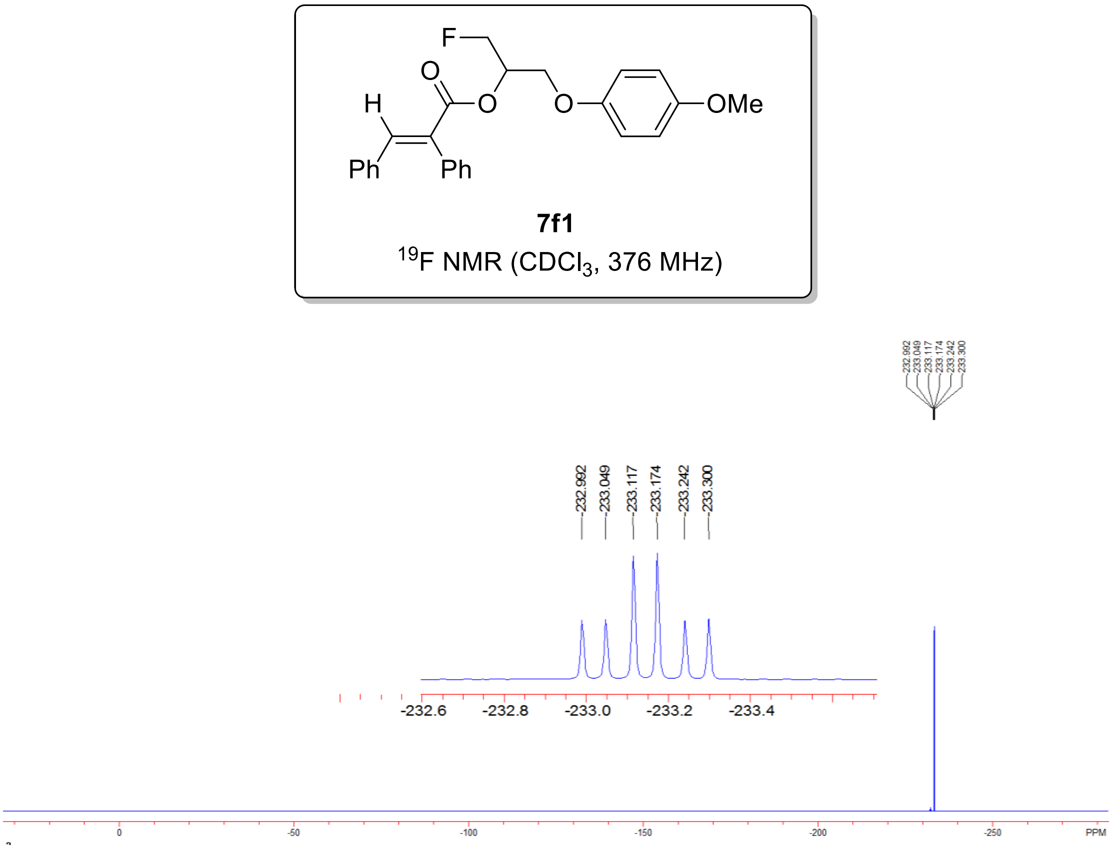

Supplementary Figure 143. ^{19}F NMR Spectrum of 7f1

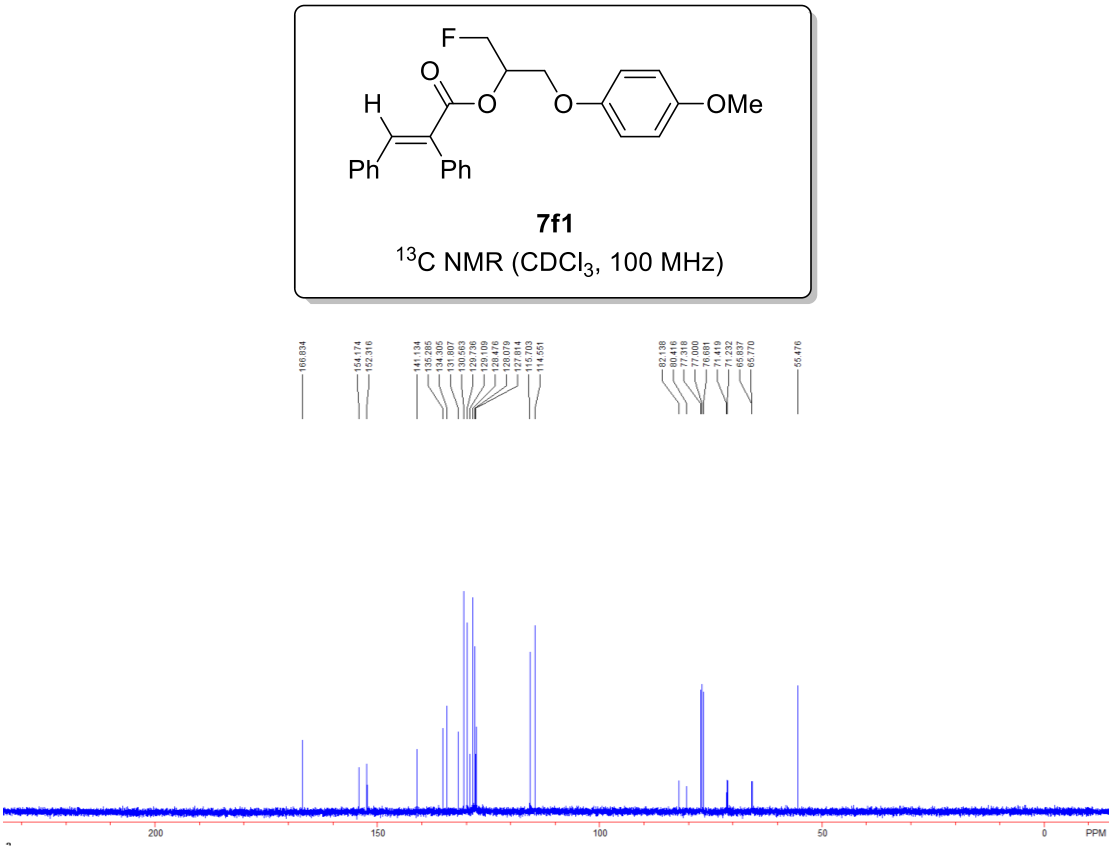

Supplementary Figure 144. ^{13}C NMR Spectrum of 7f1

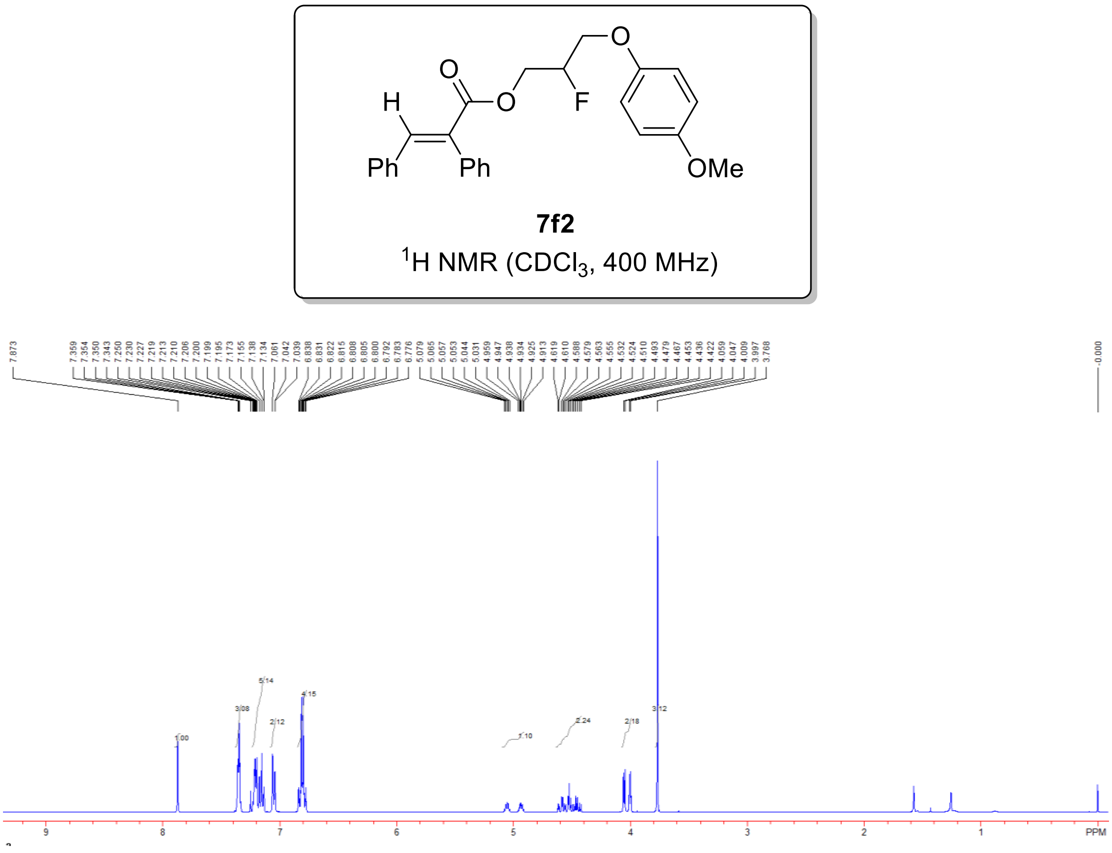

Supplementary Figure 145. ^{1}H NMR Spectrum of 7f2

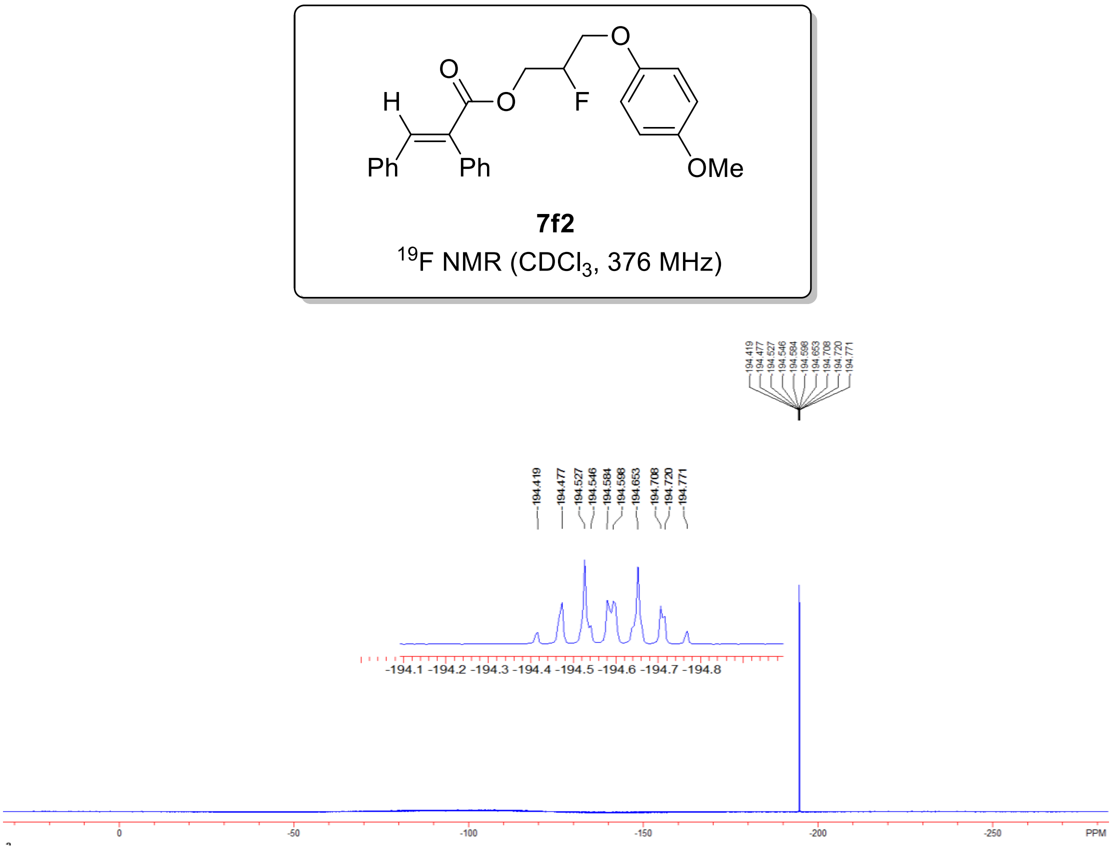

Supplementary Figure 146. ^{19}F NMR Spectrum of 7f2

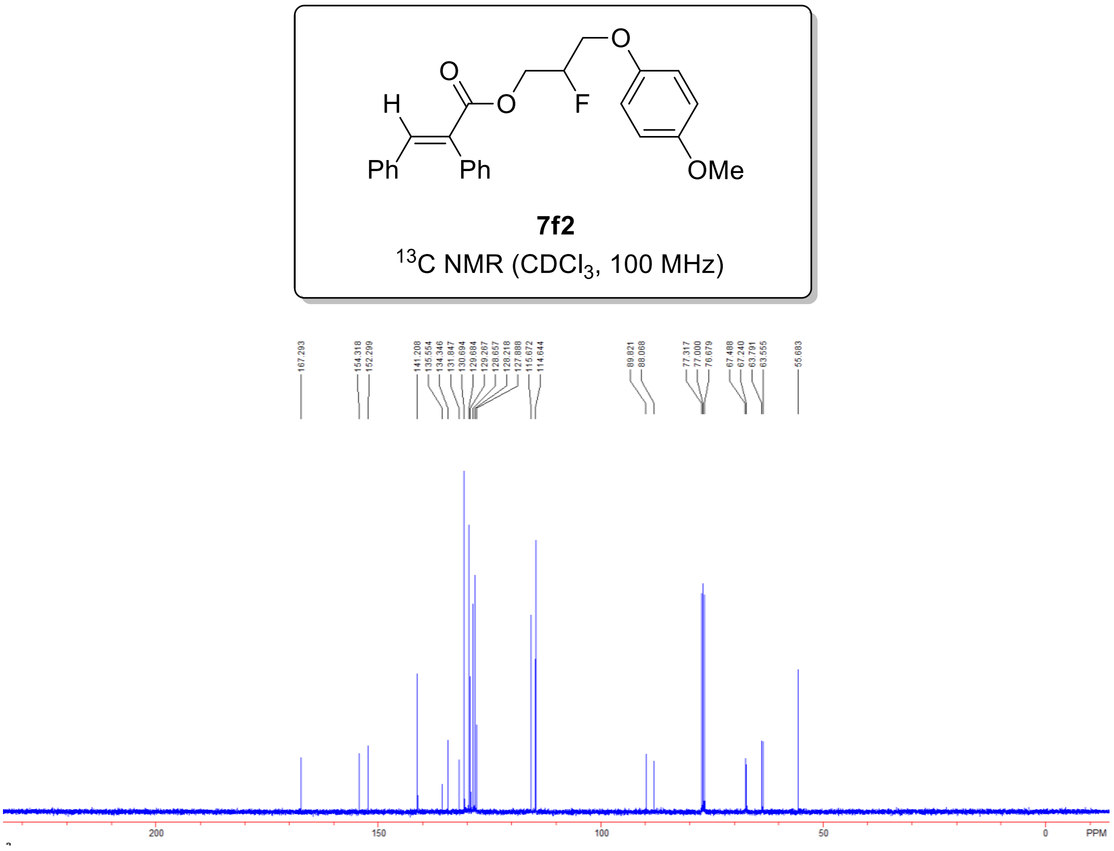

Supplementary Figure 147. ^{13}C NMR Spectrum of 7f2

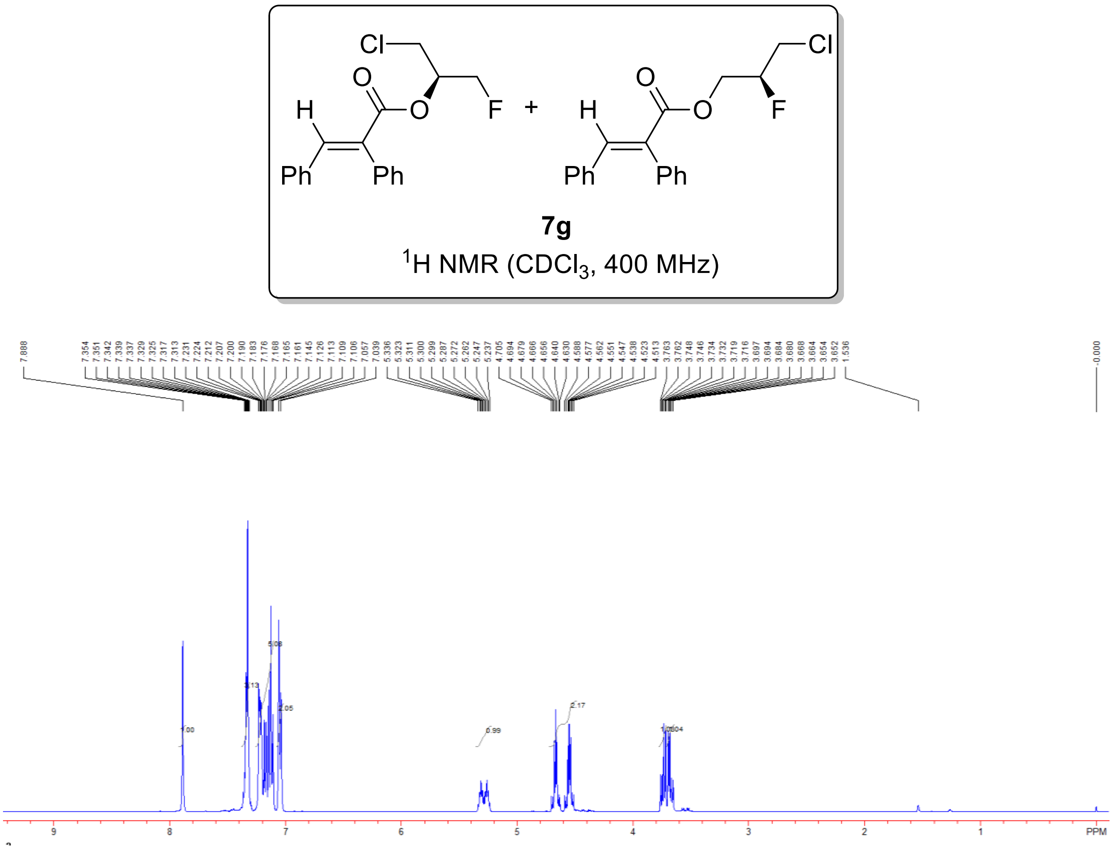

Supplementary Figure 148. ^{1}H NMR Spectrum of 7g

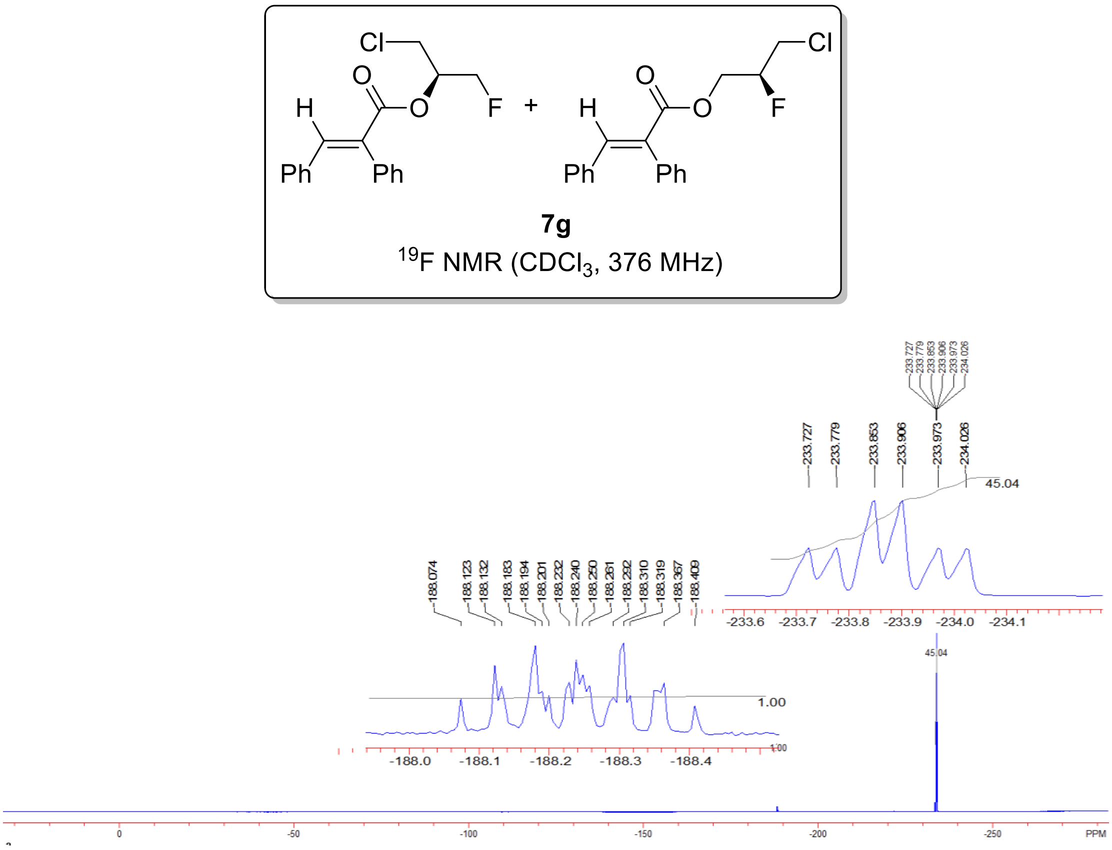

Supplementary Figure 149. ^{19}F NMR Spectrum of 7g

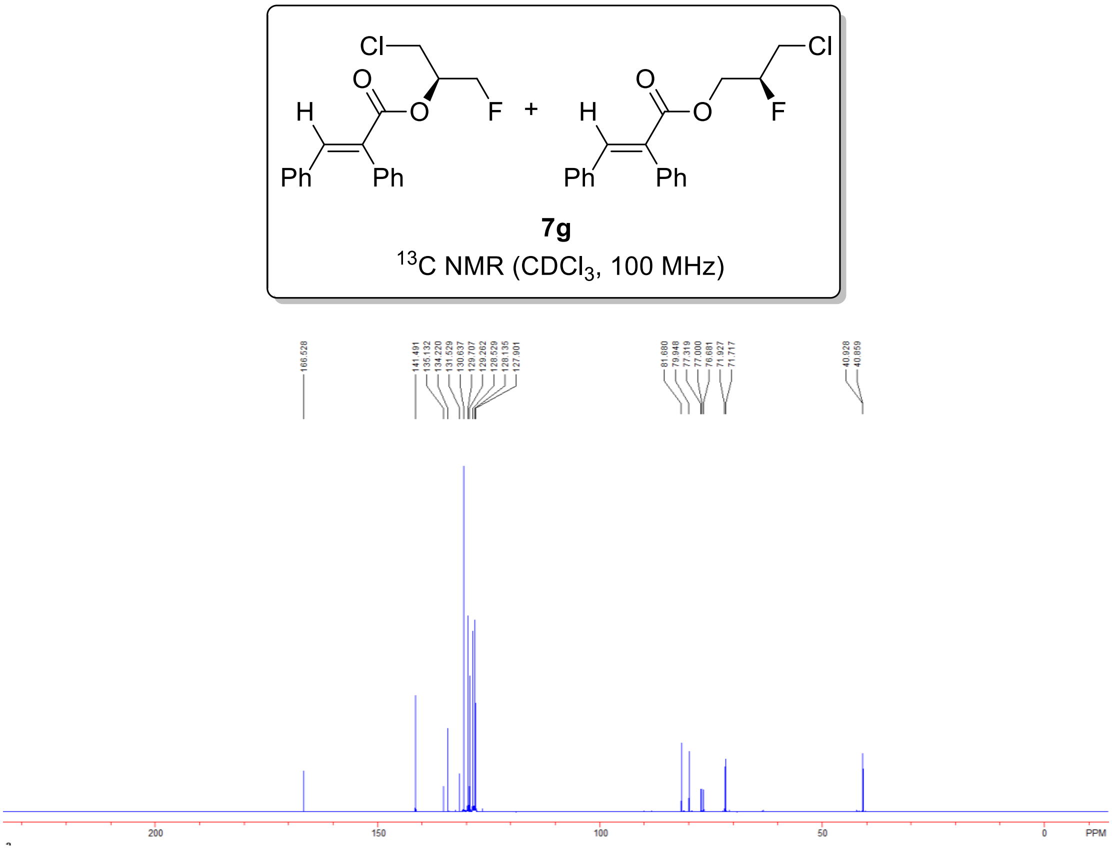

Supplementary Figure 150. ^{13}C NMR Spectrum of 7g

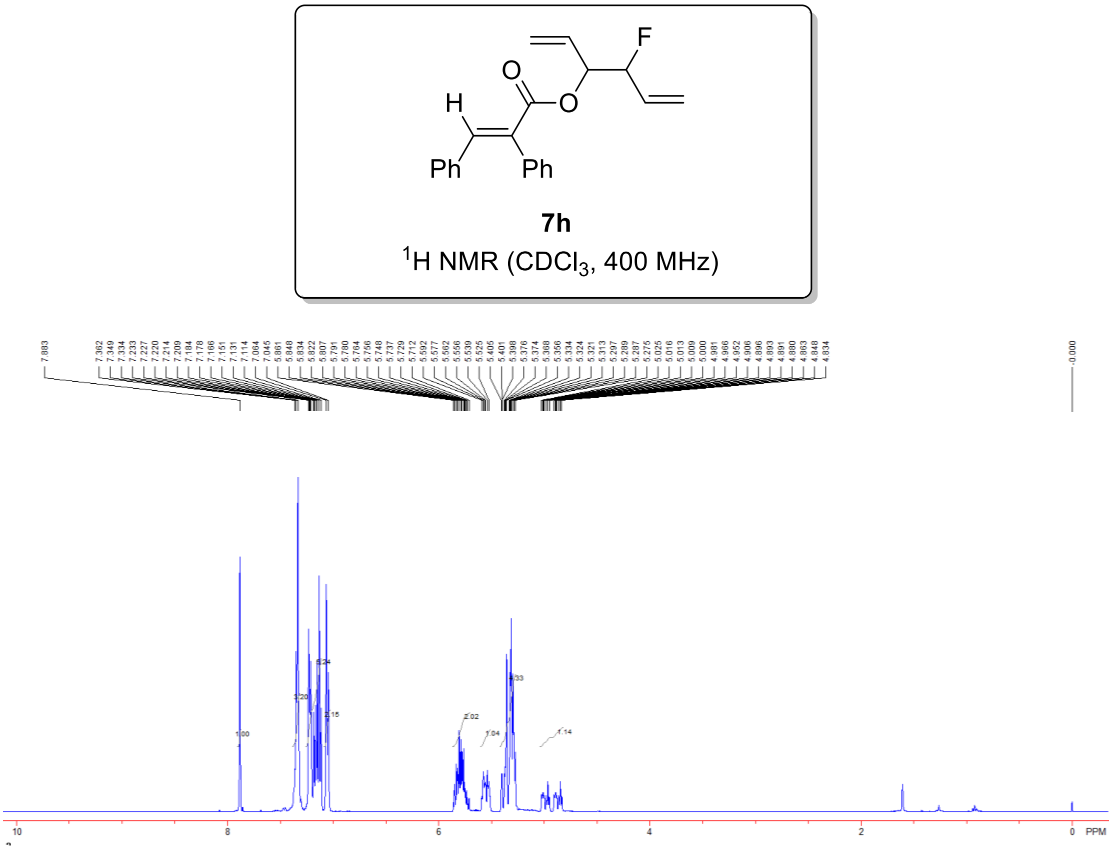

Supplementary Figure 151. ^{1}H NMR Spectrum of 7h

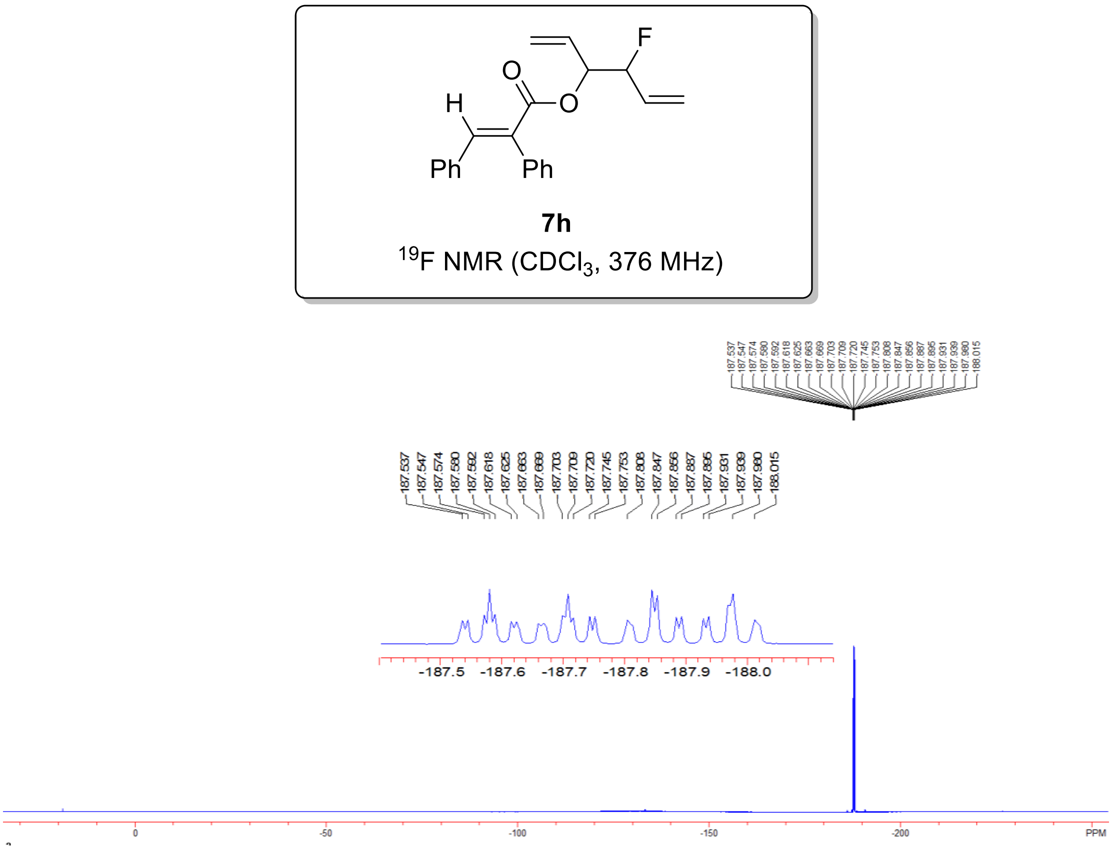

Supplementary Figure 152. ^{19}F NMR Spectrum of 7h

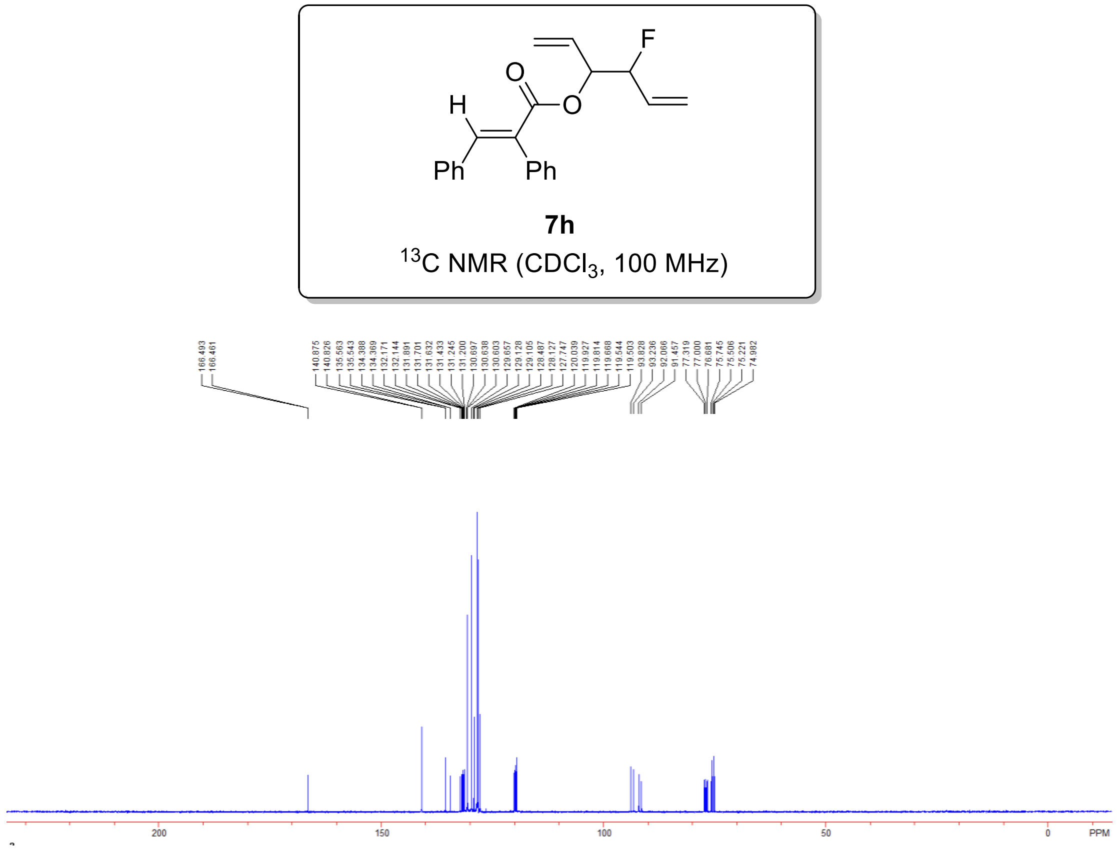

Supplementary Figure 153. ^{13}C NMR Spectrum of 7h

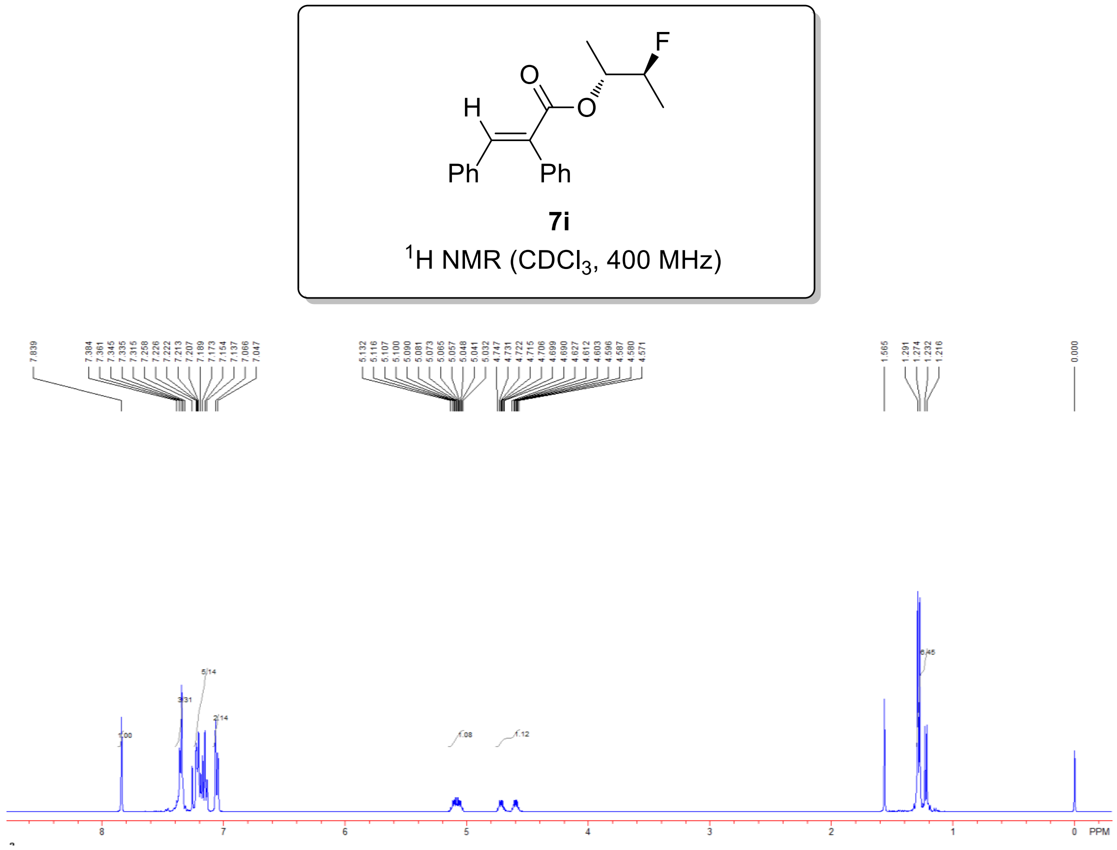

Supplementary Figure 154. ^{1}H NMR Spectrum of 7i

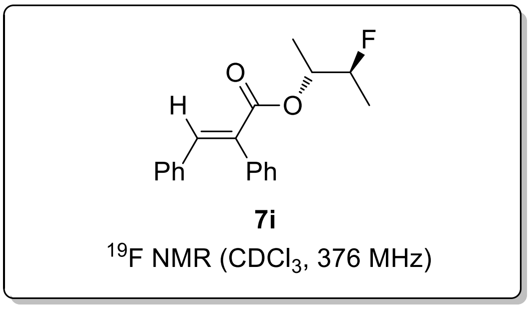

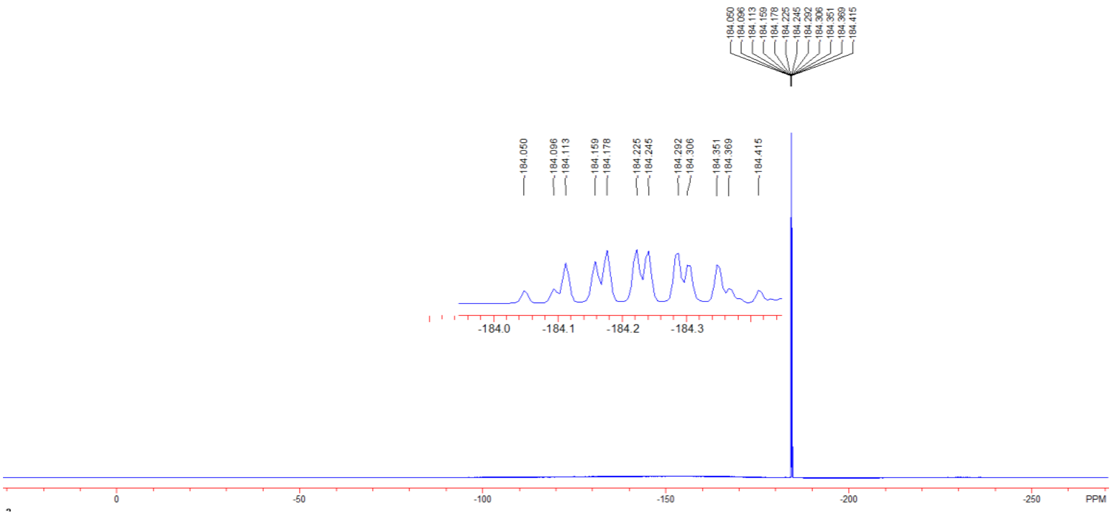

Supplementary Figure 155. ^{19}F NMR Spectrum of 7i

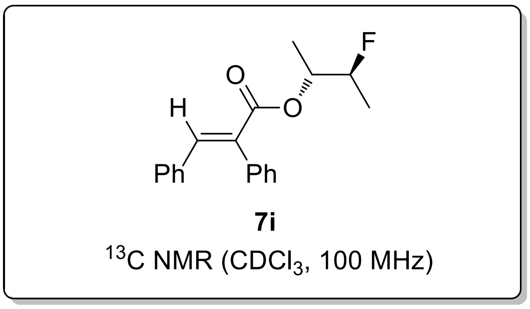

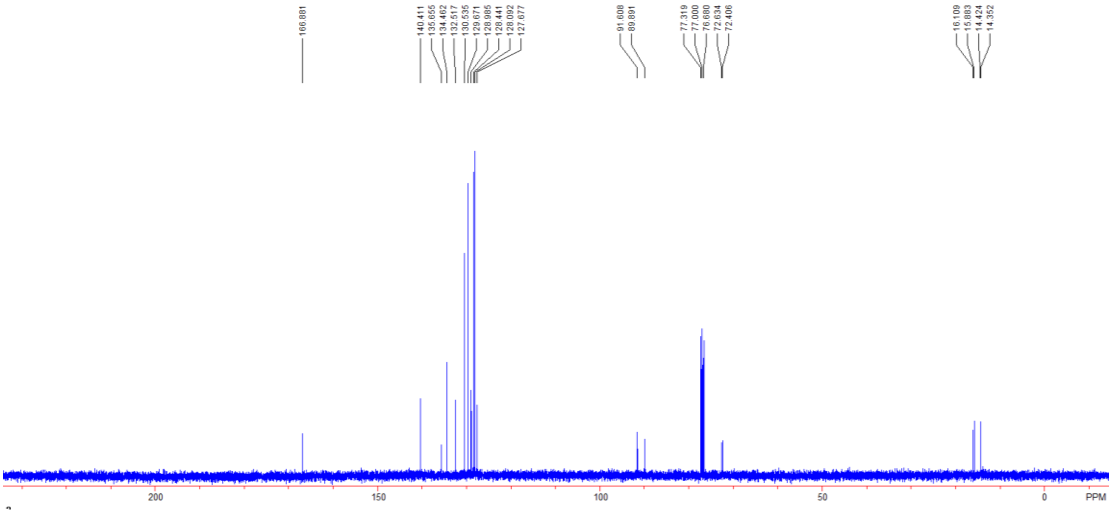

Supplementary Figure 156. ^{13}C NMR Spectrum of 7i

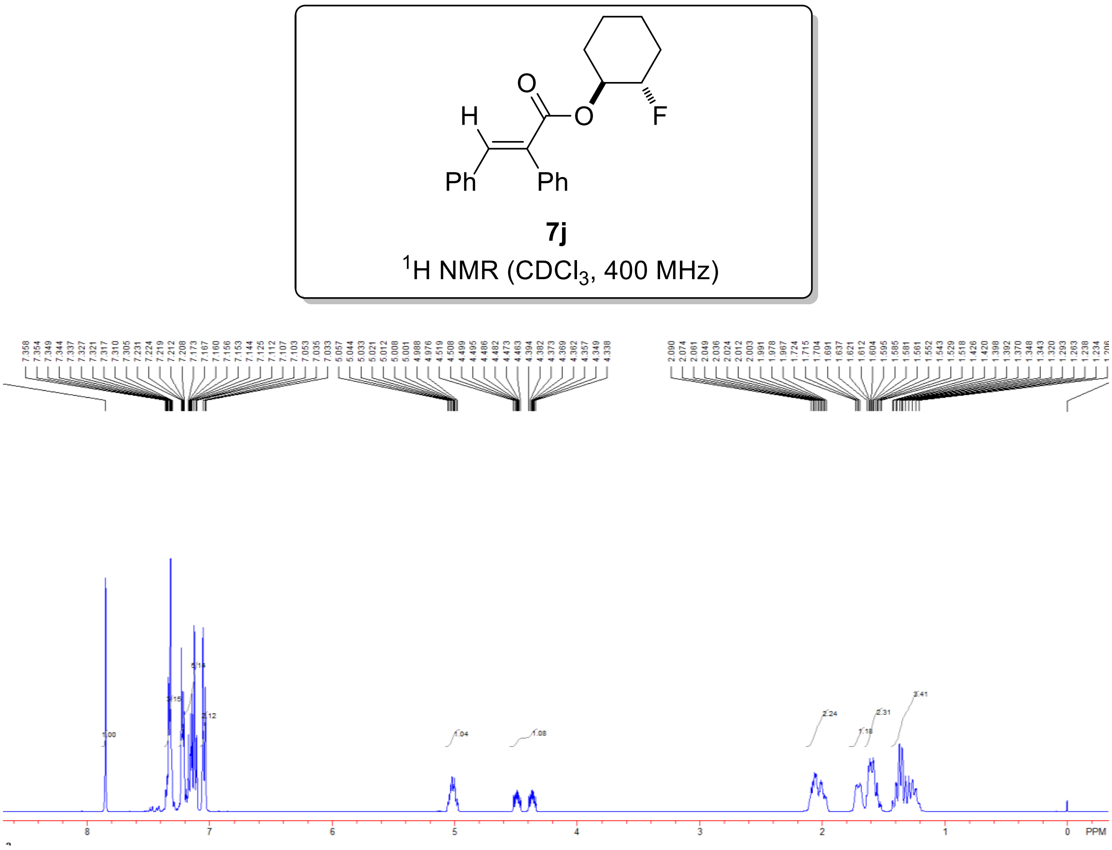

Supplementary Figure 157. ^{1}H NMR Spectrum of 7j

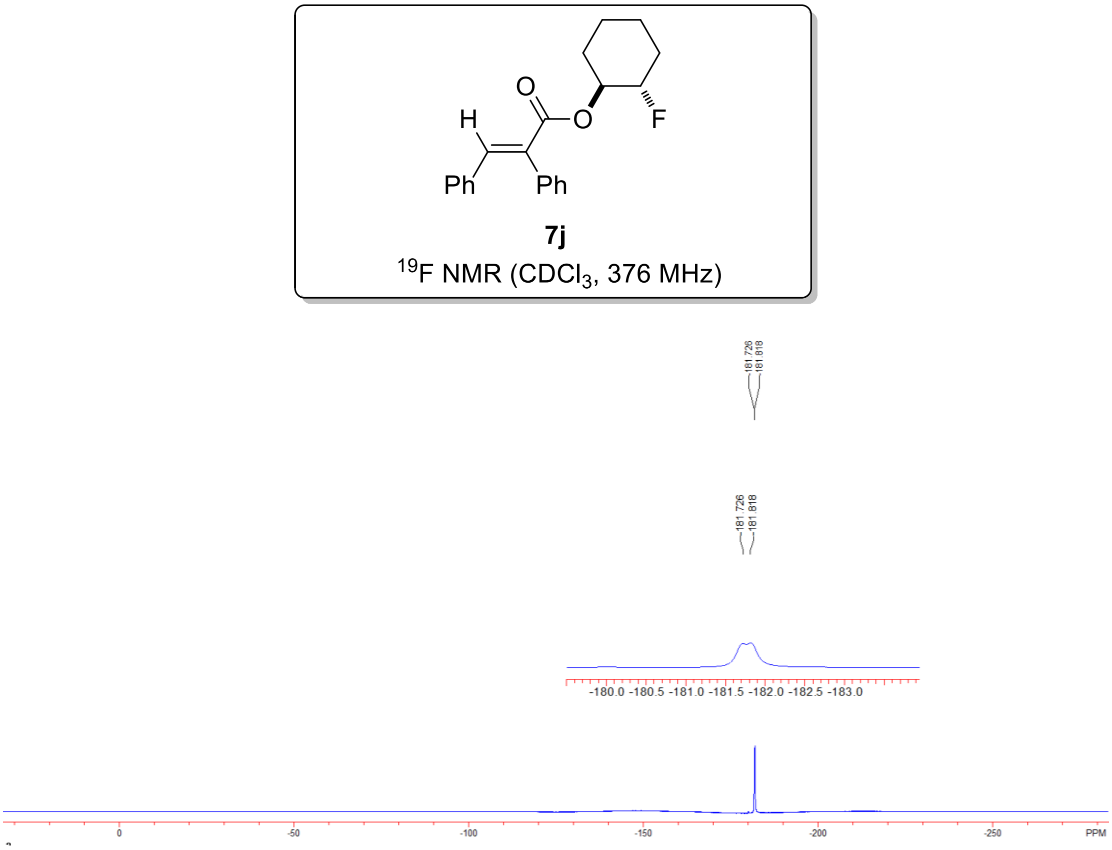

Supplementary Figure 158. ^{19}F NMR Spectrum of 7j

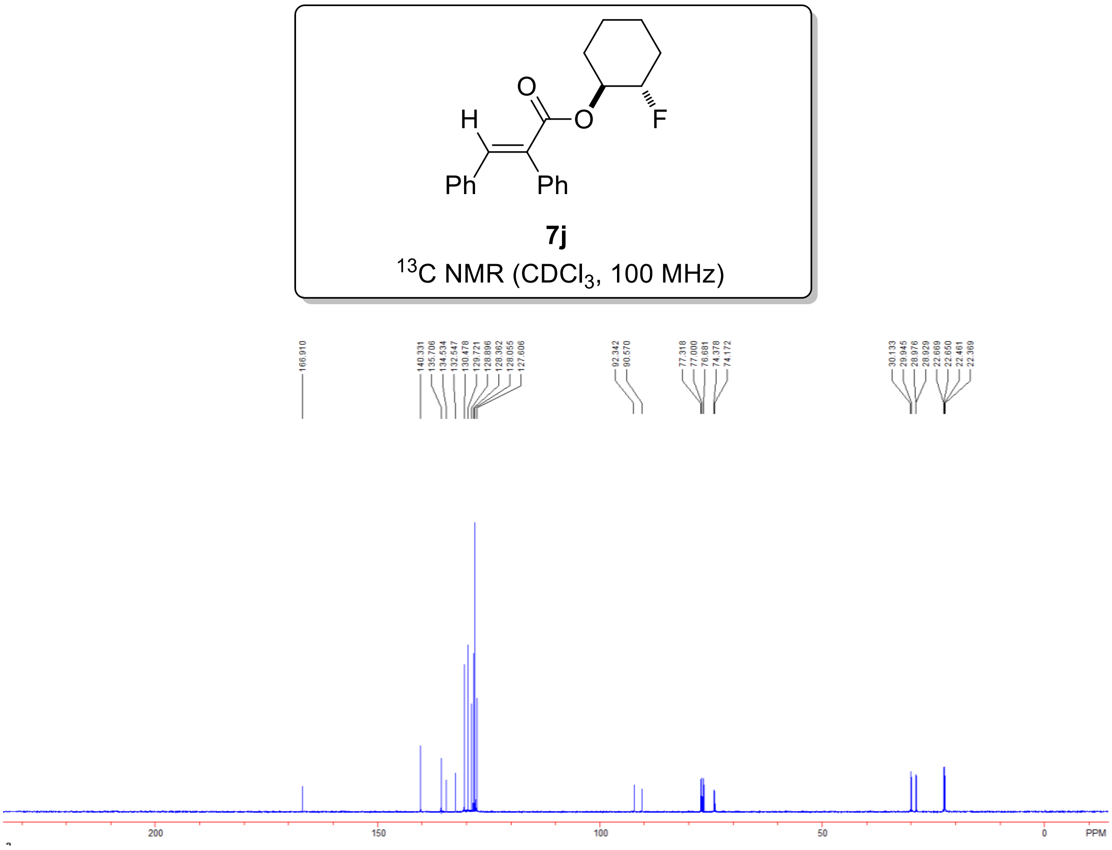

Supplementary Figure 159. ^{13}C NMR Spectrum of 7j

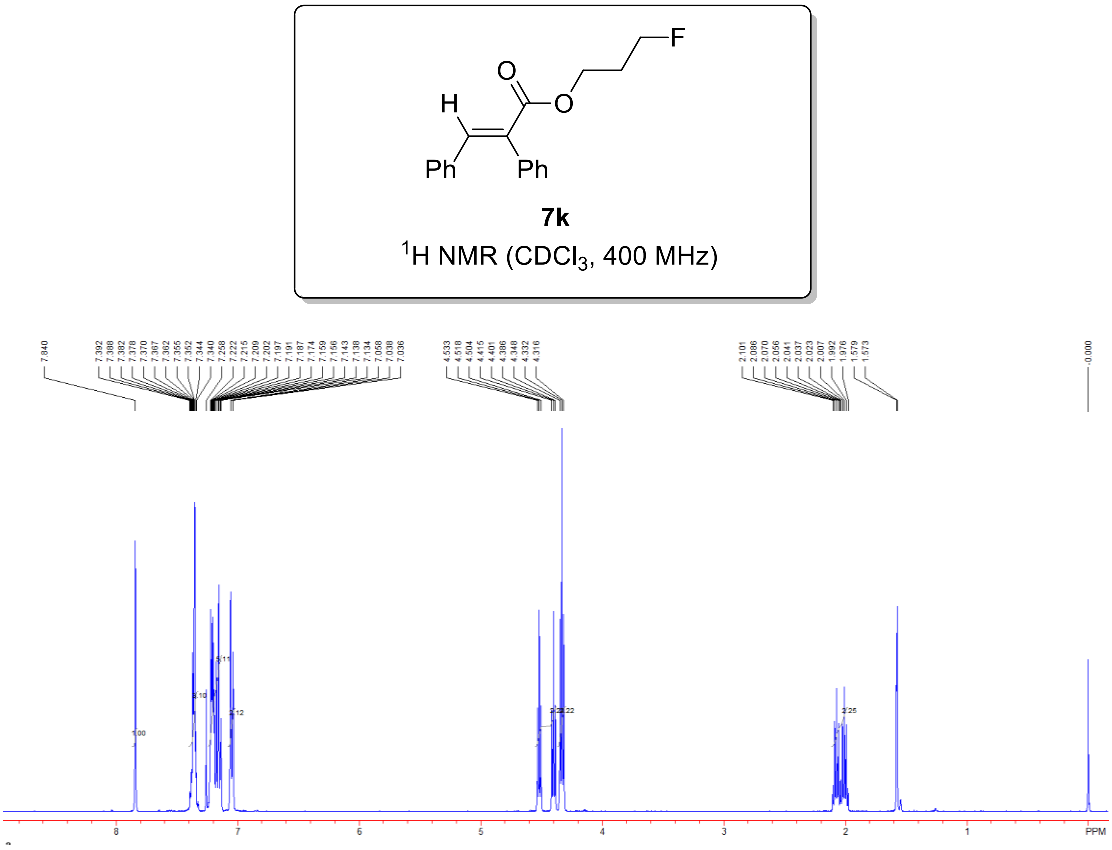

Supplementary Figure 160. ^{1}H NMR Spectrum of 7k

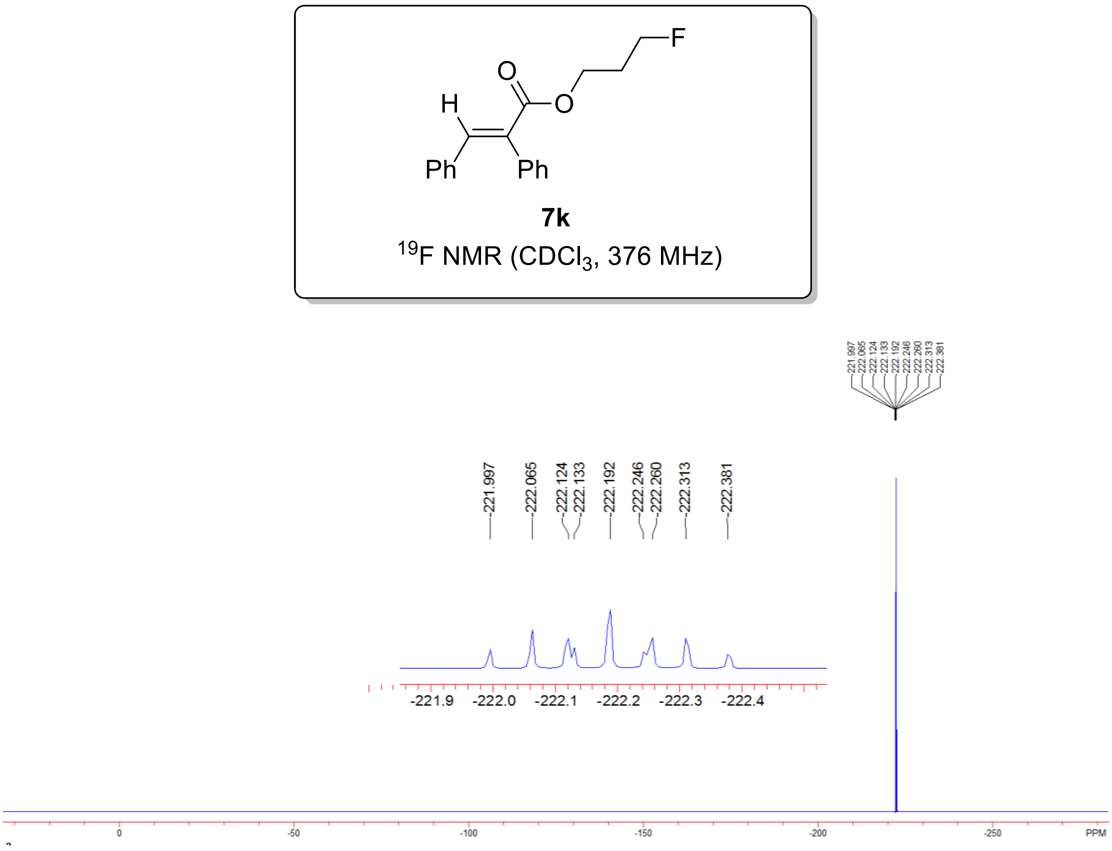

Supplementary Figure 161. ^{19}**F NMR Spectrum of 7k**

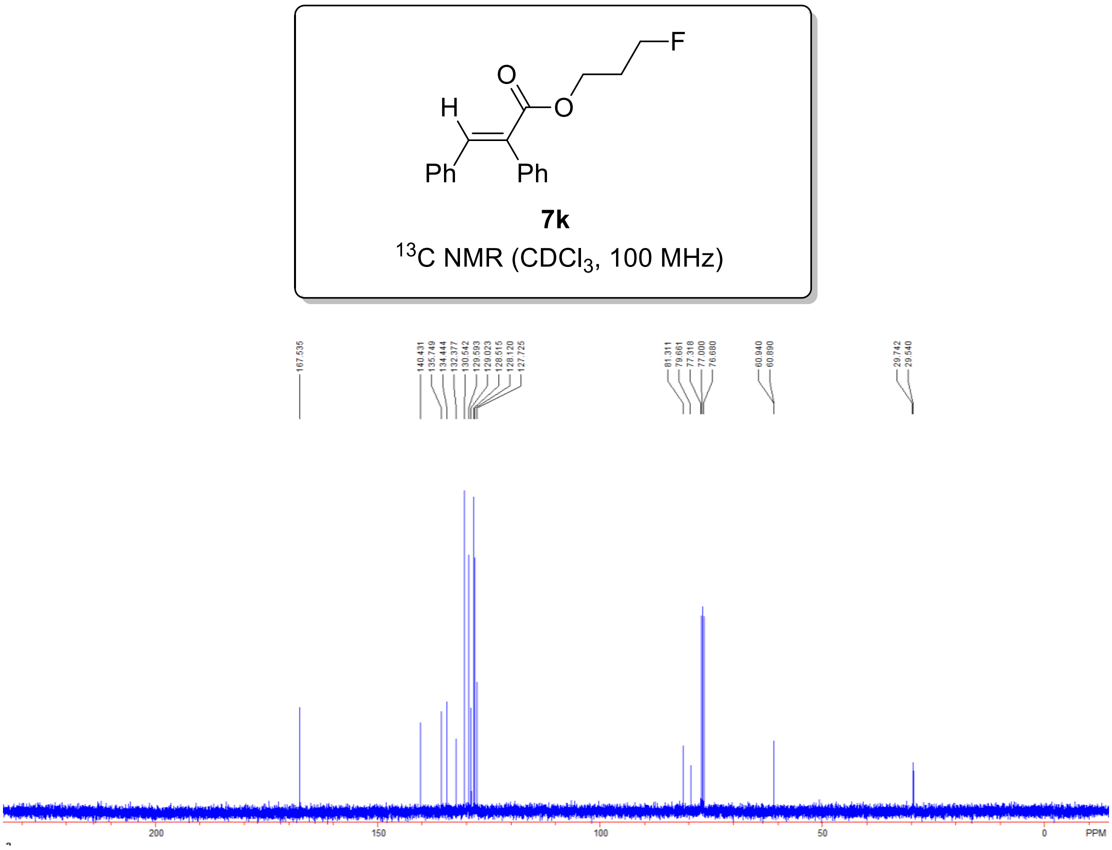

Supplementary Figure 162. ^{13}**C NMR Spectrum of 7k**

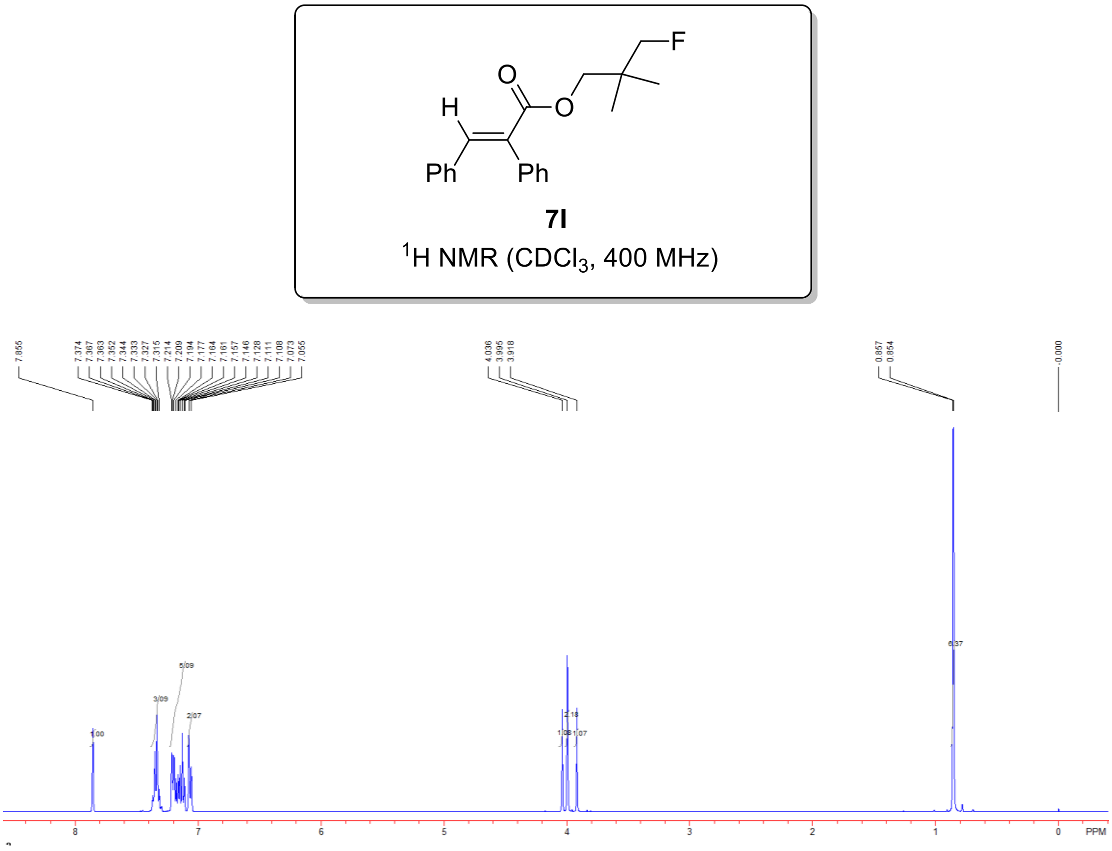

Supplementary Figure 163. ^{1}H NMR Spectrum of 7l

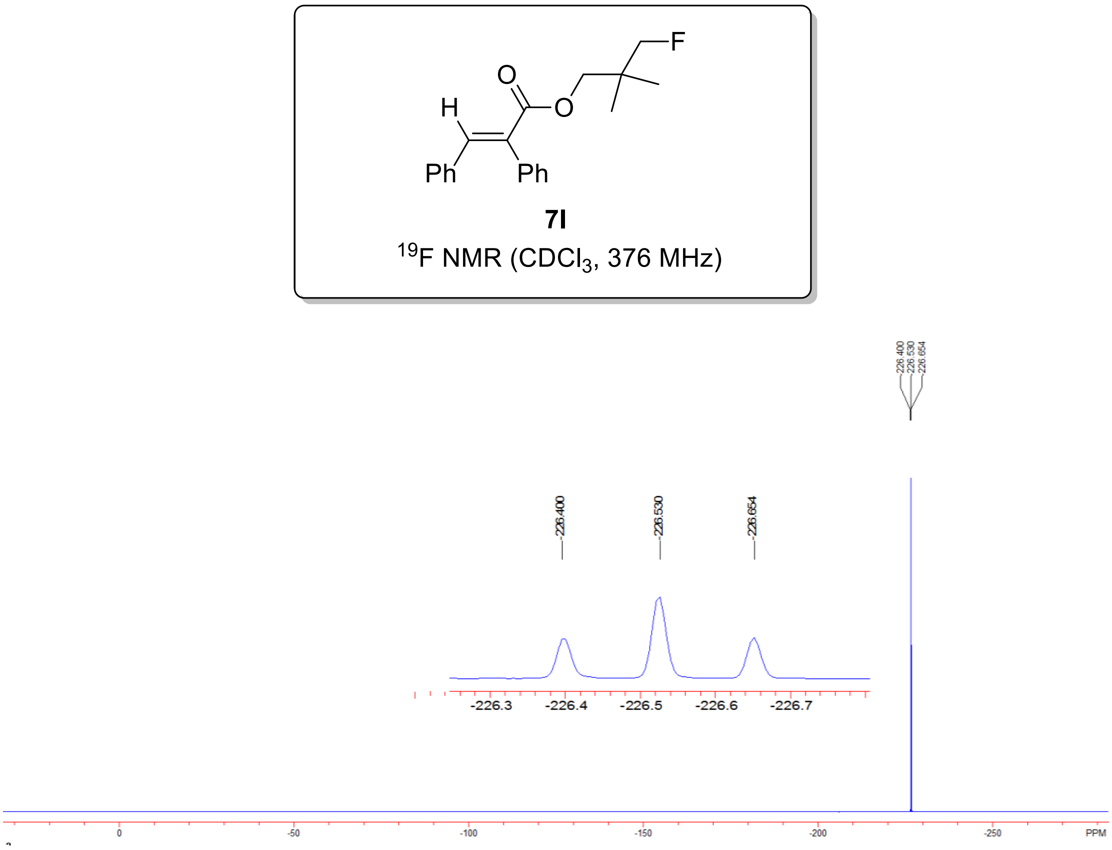

Supplementary Figure 164. ^{19}F NMR Spectrum of 7l

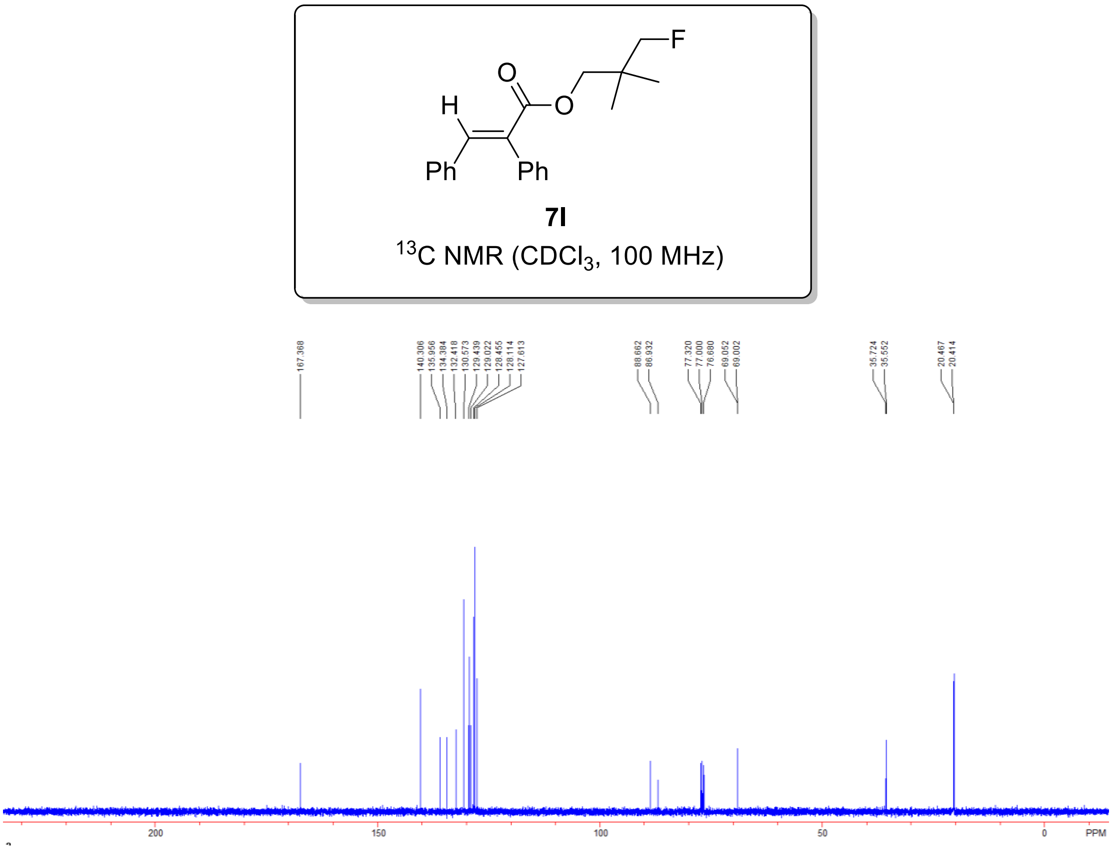

Supplementary Figure 165. ^{13}**C NMR Spectrum of 7l**

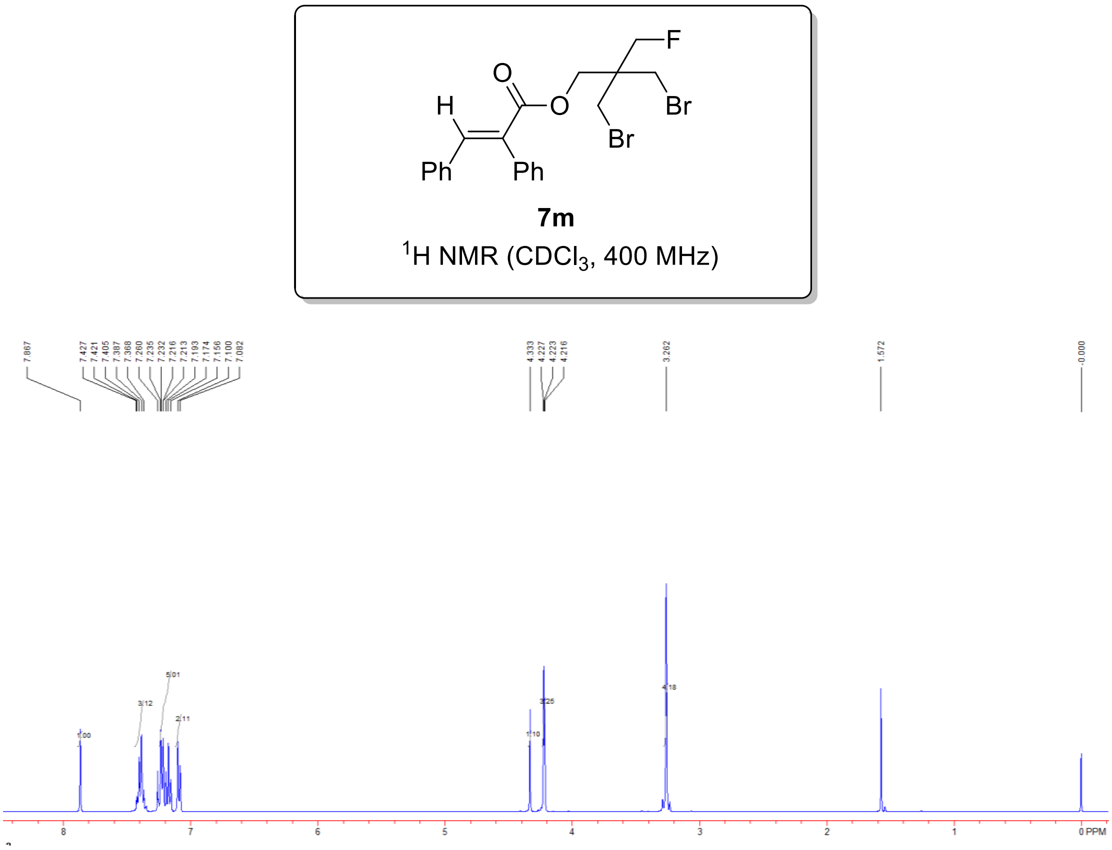

Supplementary Figure 166. ^{1}**H NMR Spectrum of 7m**

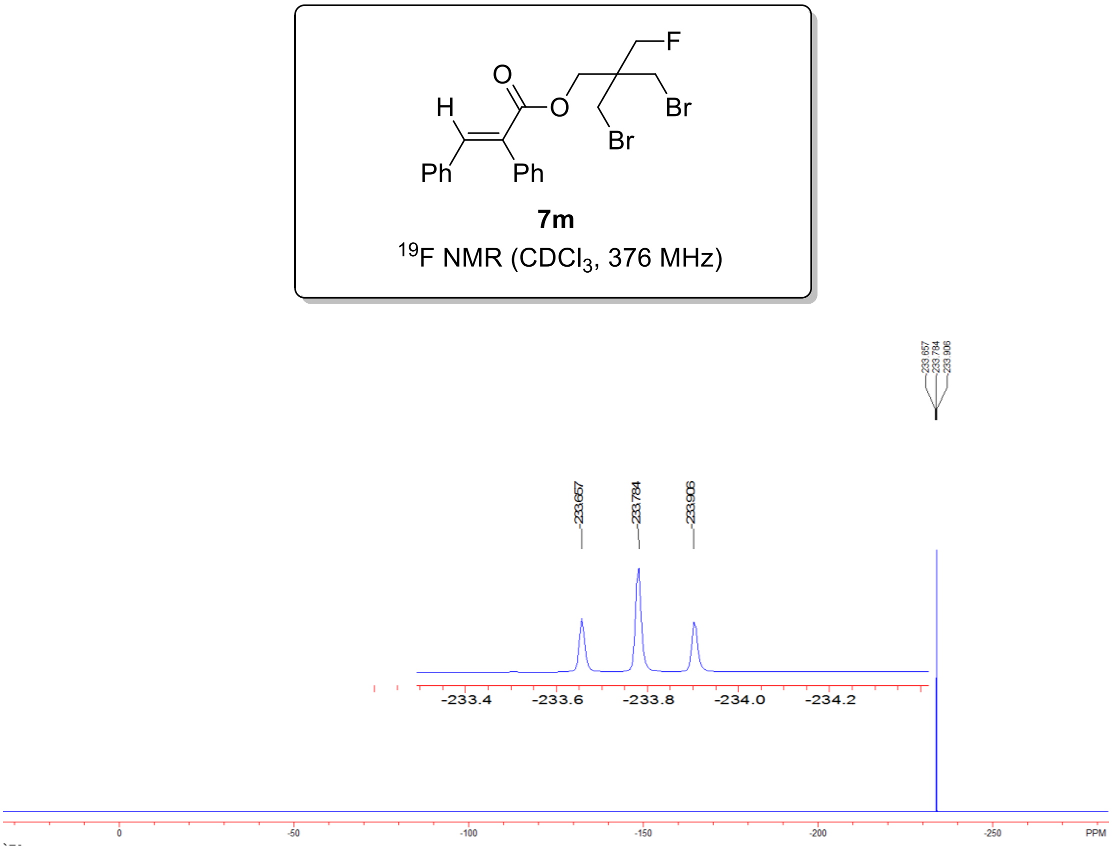

Supplementary Figure 167. ^{19}**F NMR Spectrum of 7m**

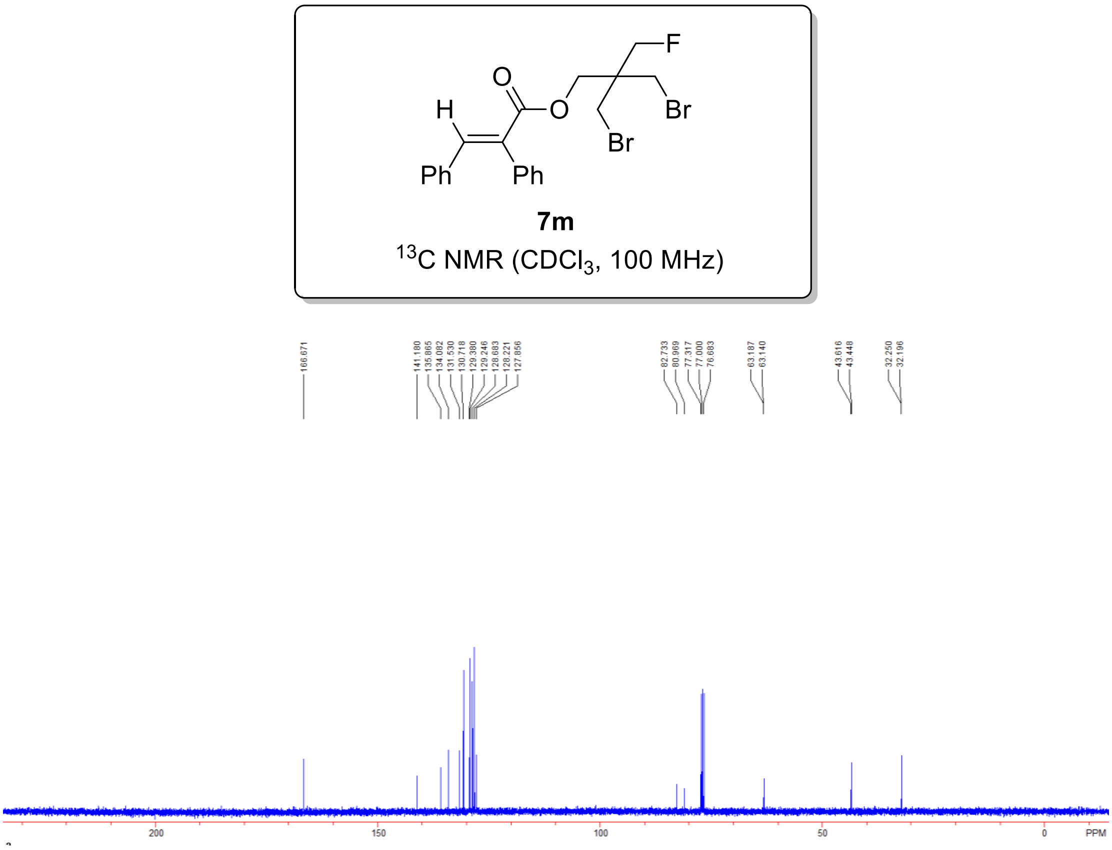

Supplementary Figure 168. ^{13}**C NMR Spectrum of 7m**

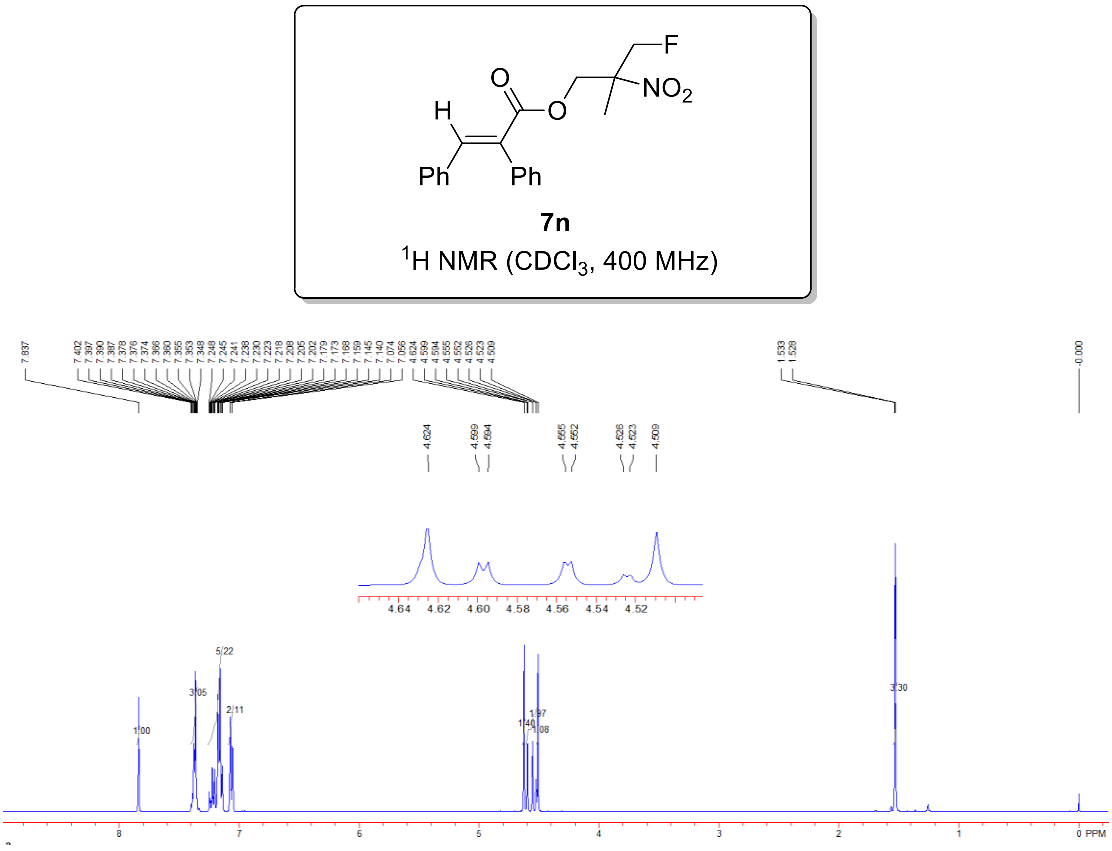

Supplementary Figure 169. ^{1}H NMR Spectrum of 7n

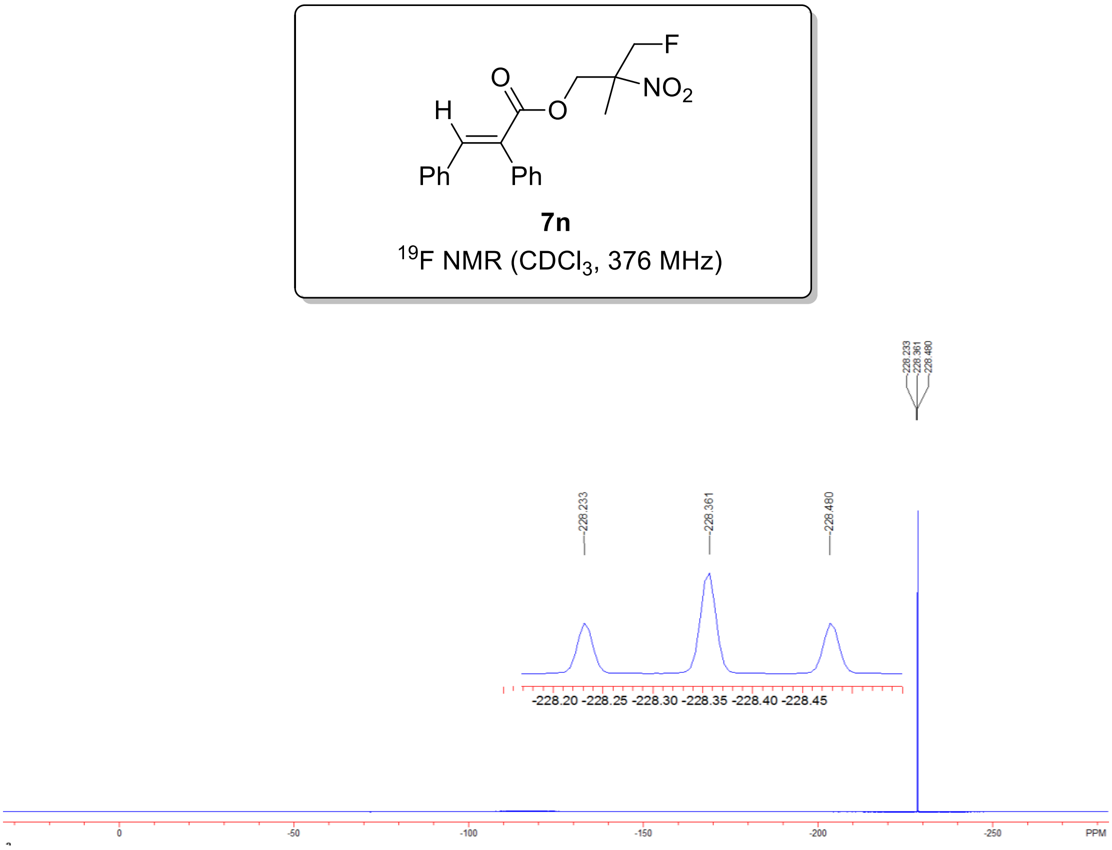

Supplementary Figure 170. ^{19}F NMR Spectrum of 7n

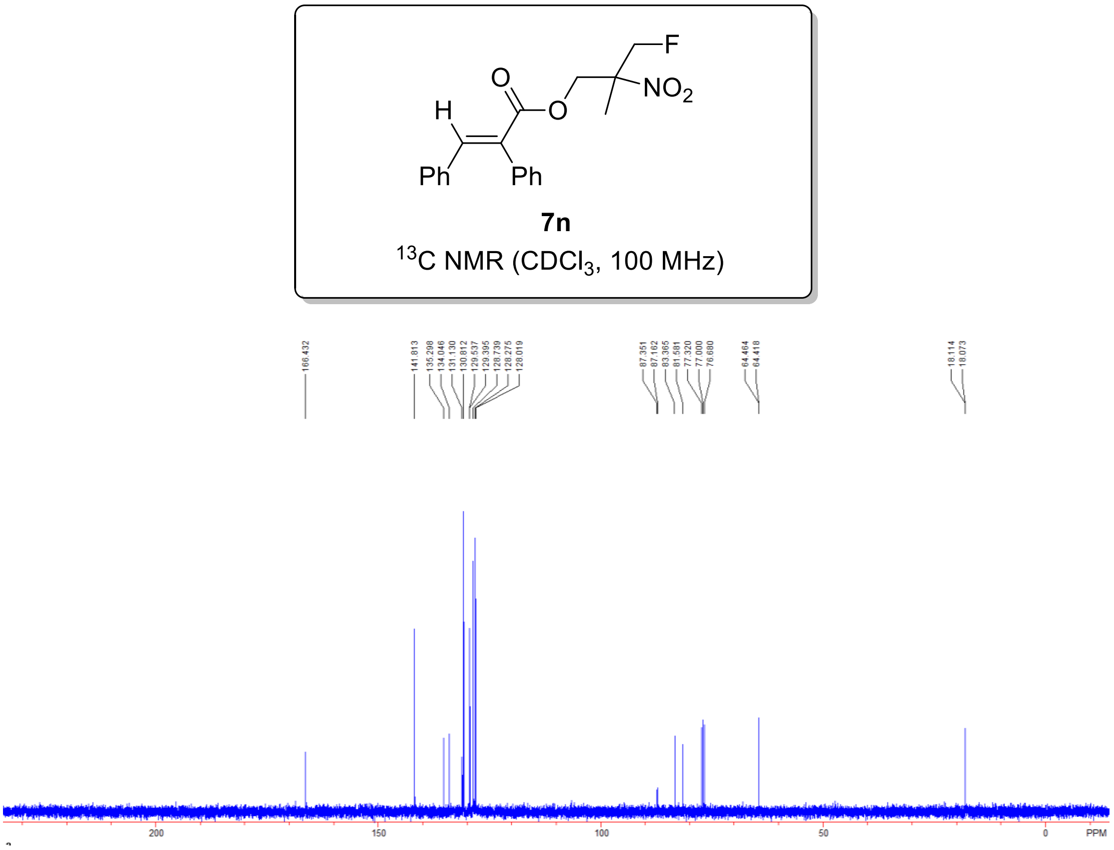

Supplementary Figure 171. ^{13}C NMR Spectrum of 7n

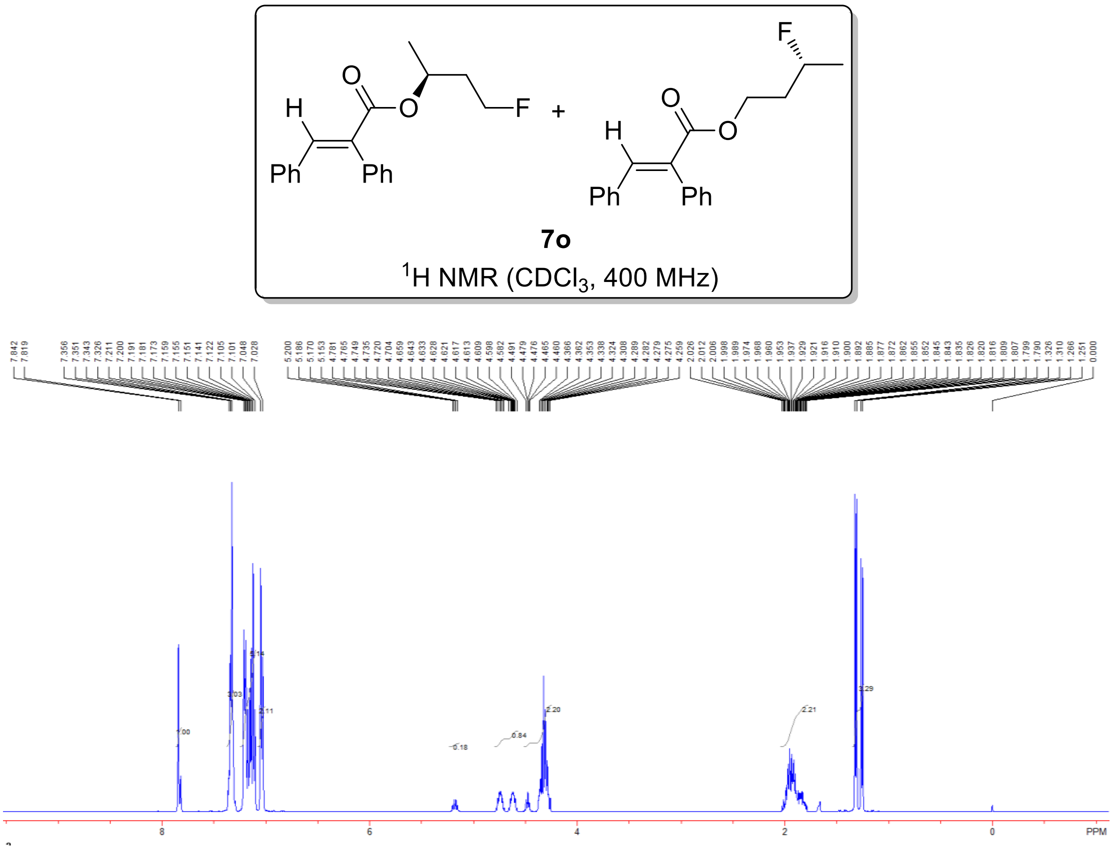

Supplementary Figure 172. ^{1}H NMR Spectrum of 7o

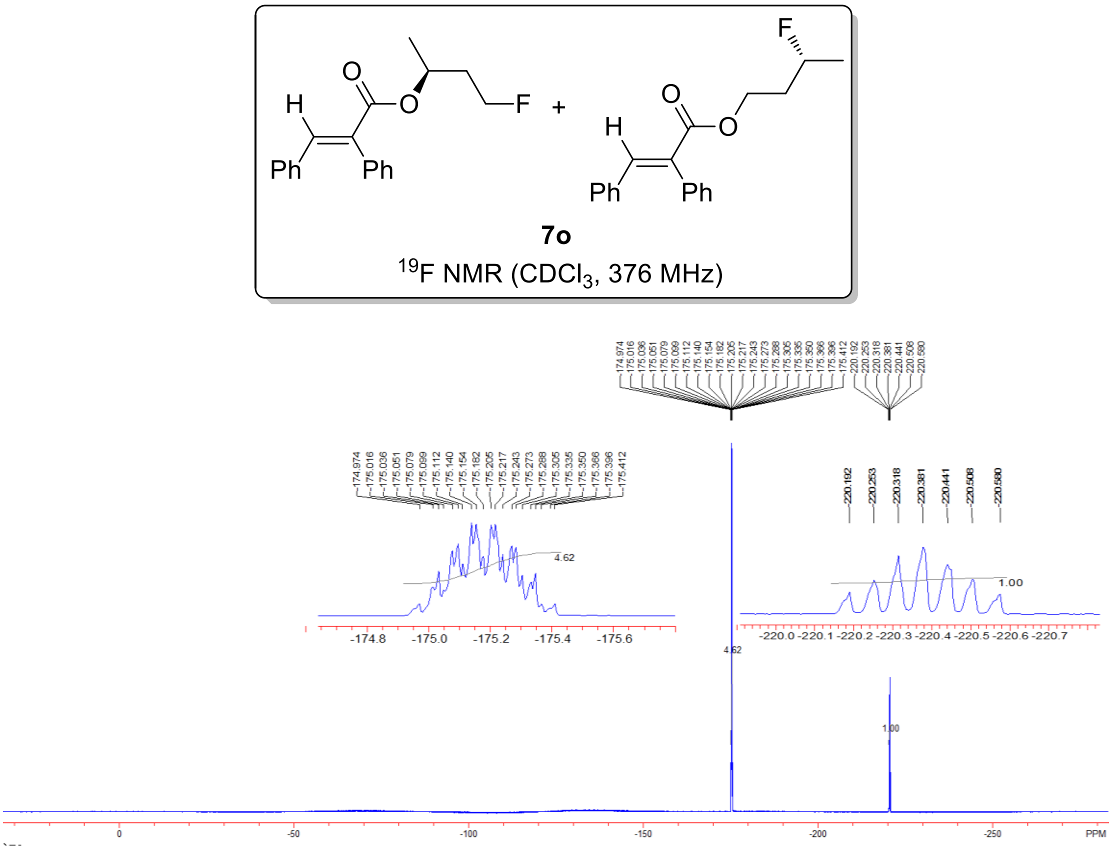

Supplementary Figure 173. ^{19}F NMR Spectrum of 7o

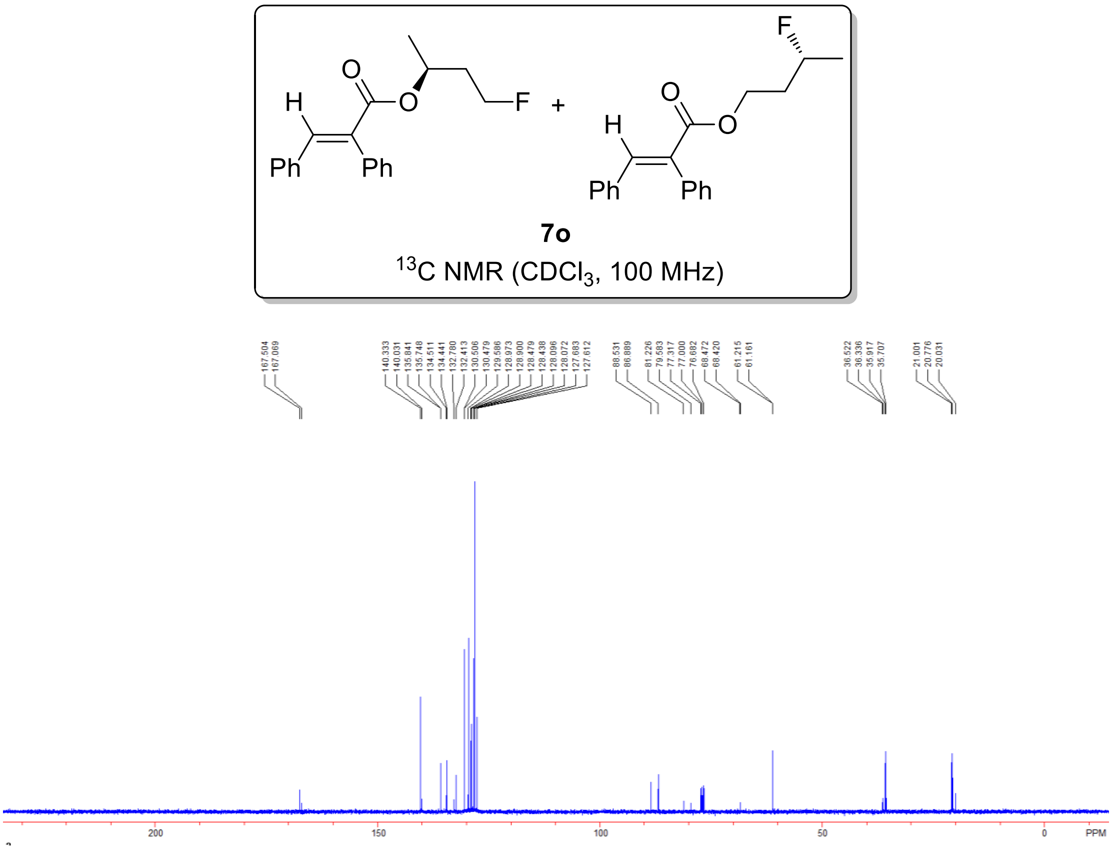

Supplementary Figure 174. ^{13}C NMR Spectrum of 7o

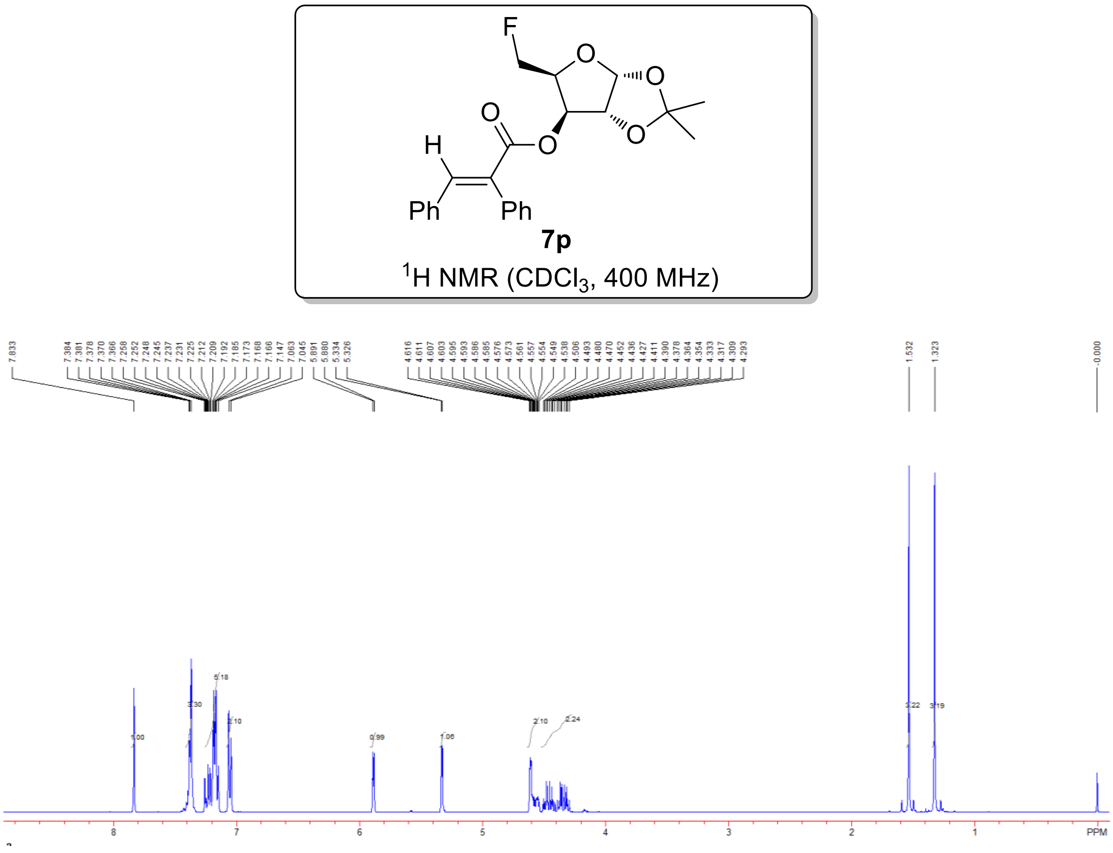

Supplementary Figure 175. ^{1}H NMR Spectrum of 7p

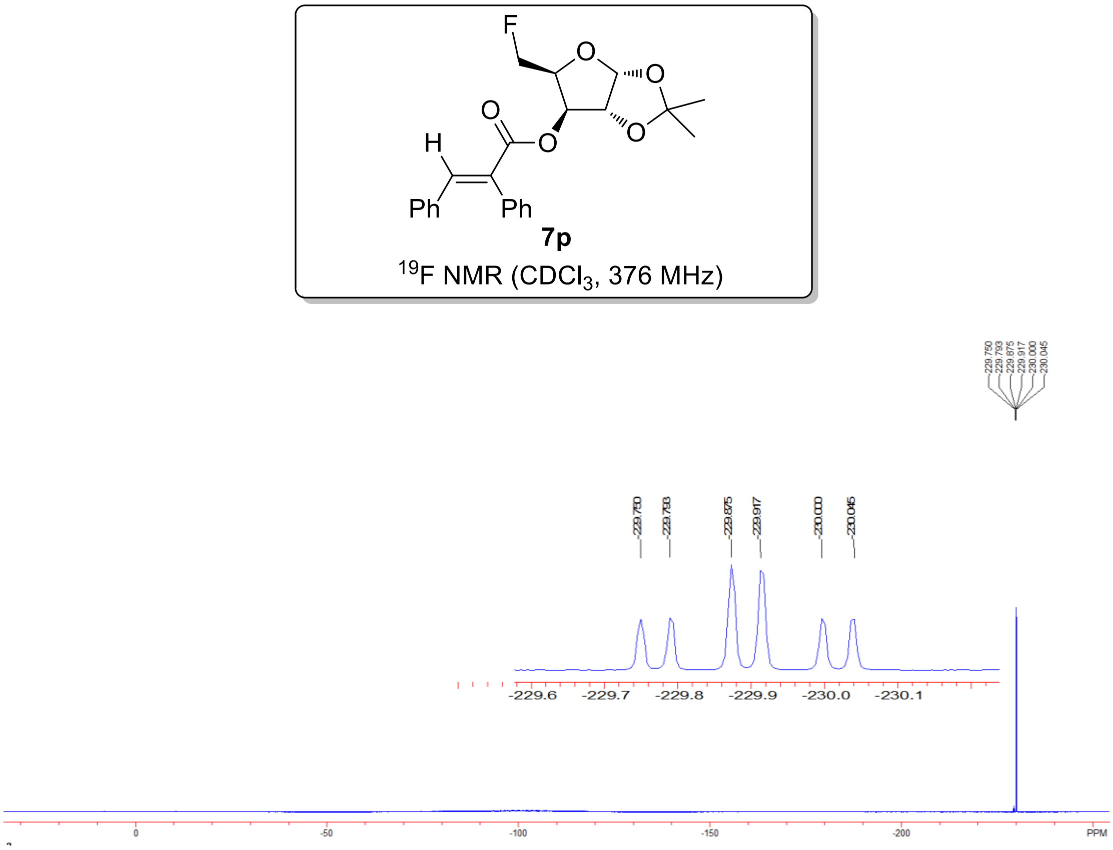

Supplementary Figure 176. ^{19}F NMR Spectrum of 7p

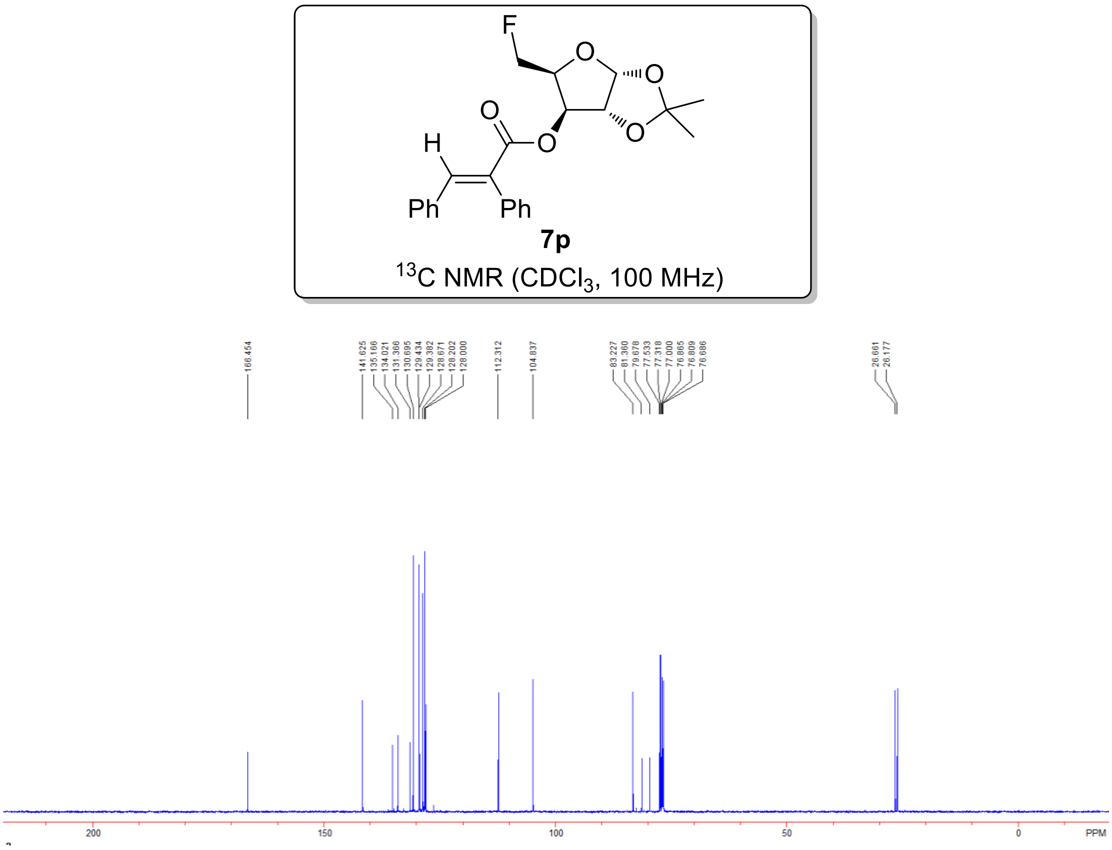

Supplementary Figure 177. ^{13}C NMR Spectrum of 7p

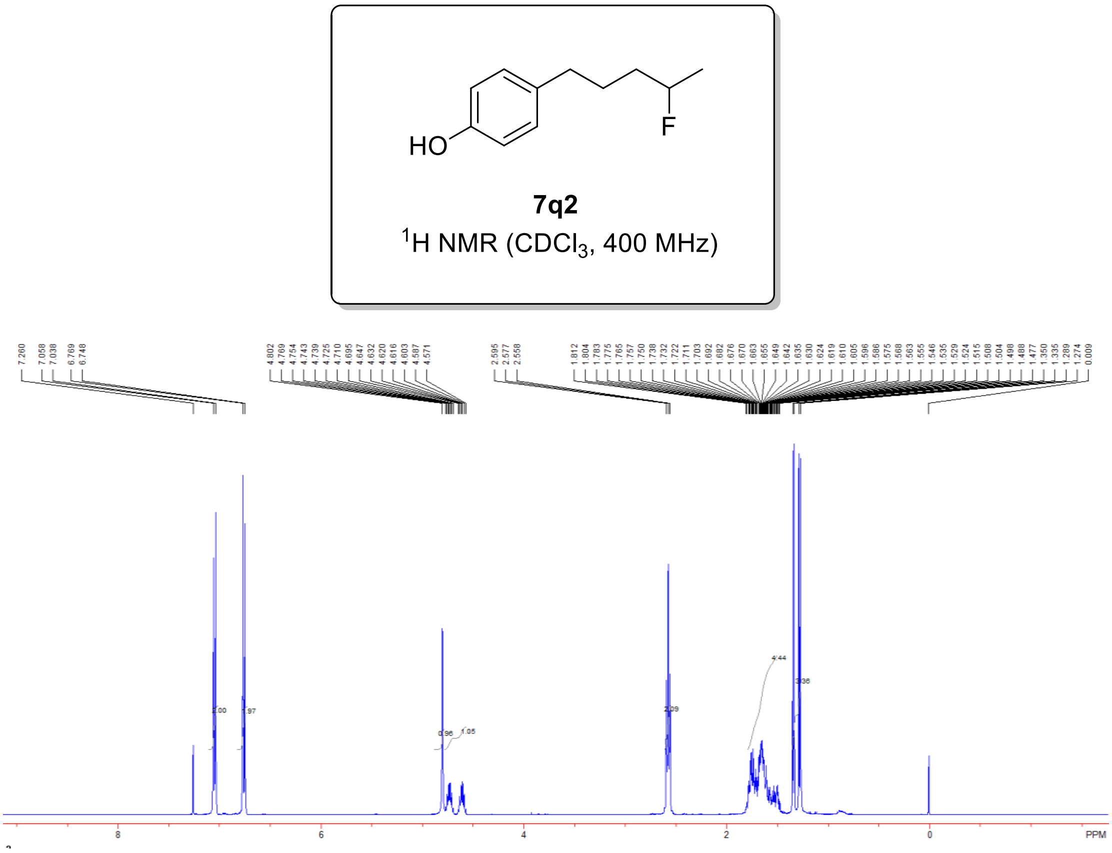

Supplementary Figure 178. ^{1}H NMR Spectrum of 7q2

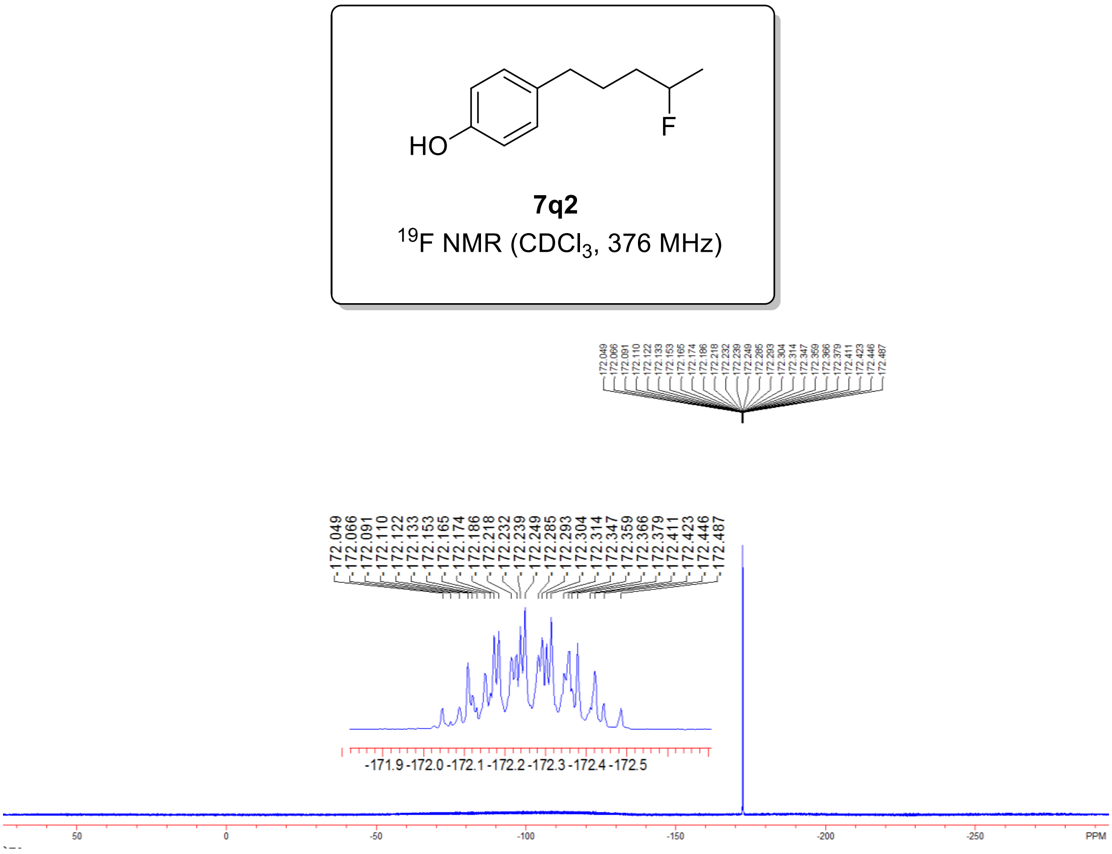

Supplementary Figure 179. ^{19}F NMR Spectrum of 7q2

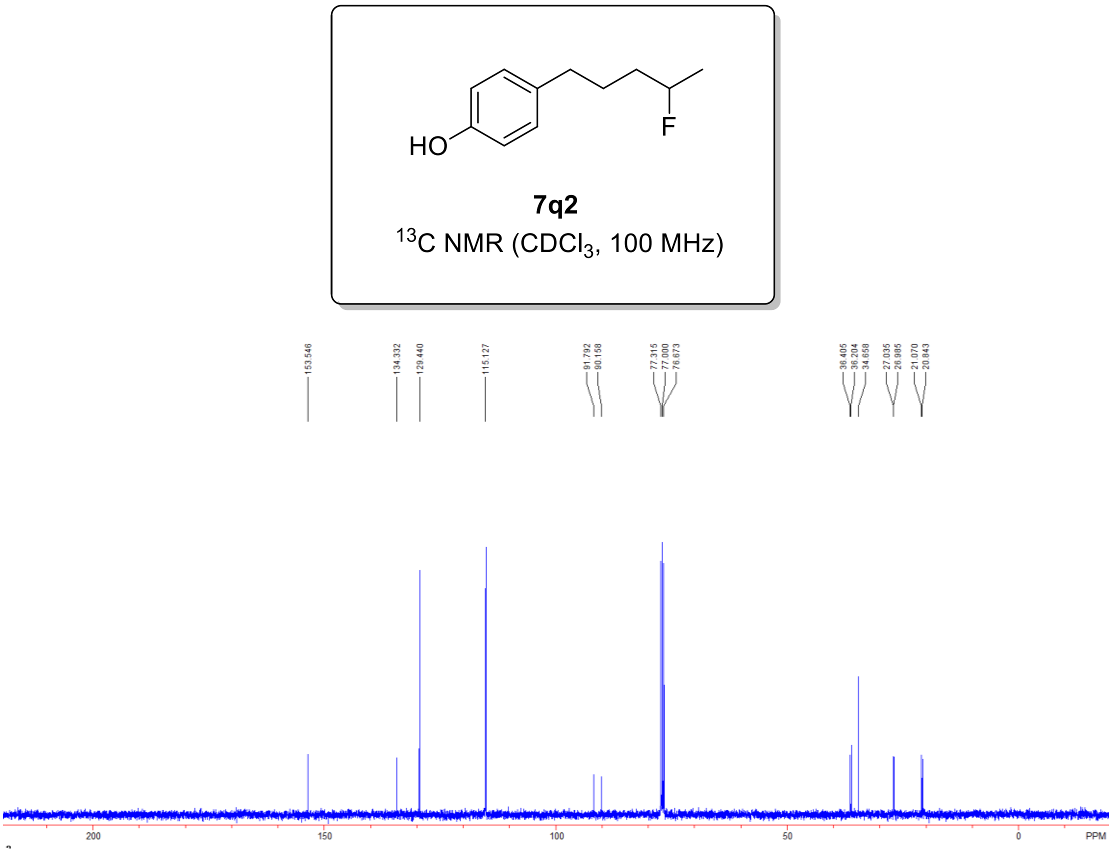

Supplementary Figure 180. ^{13}C NMR Spectrum of 7q2

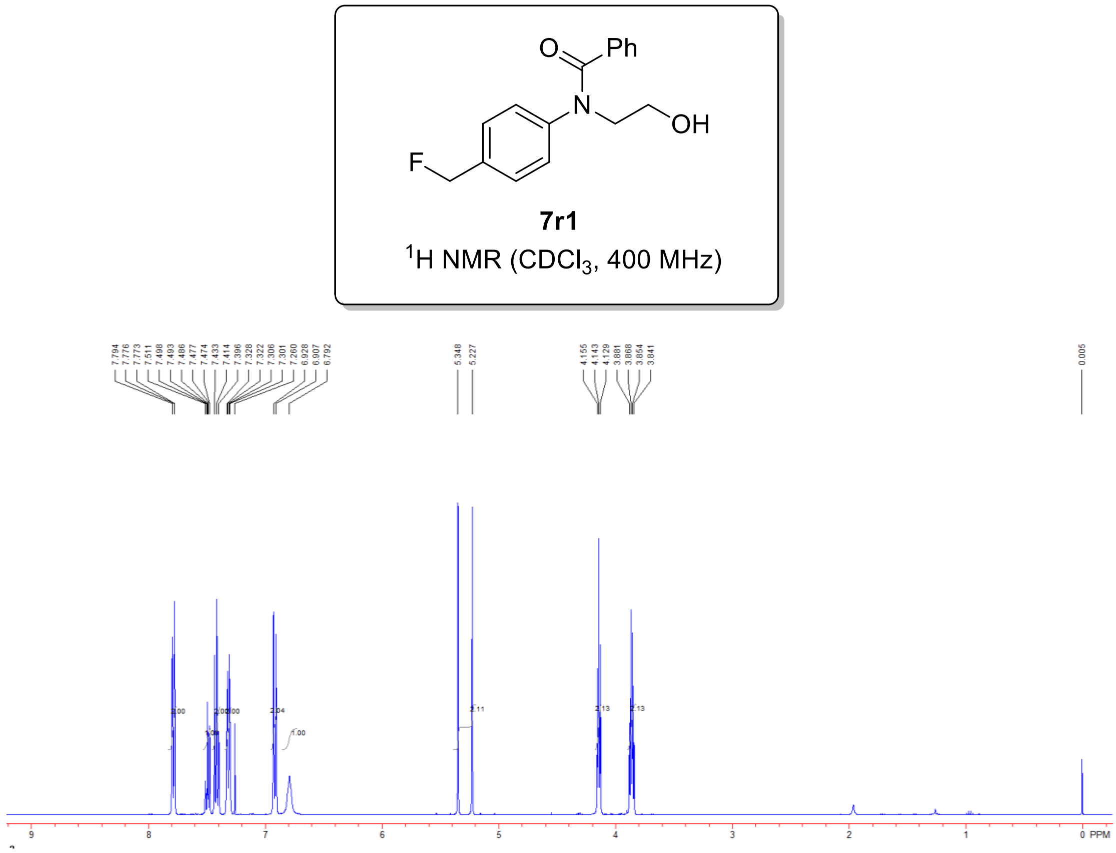

Supplementary Figure 181. ^{1}**H NMR Spectrum of 7r1**

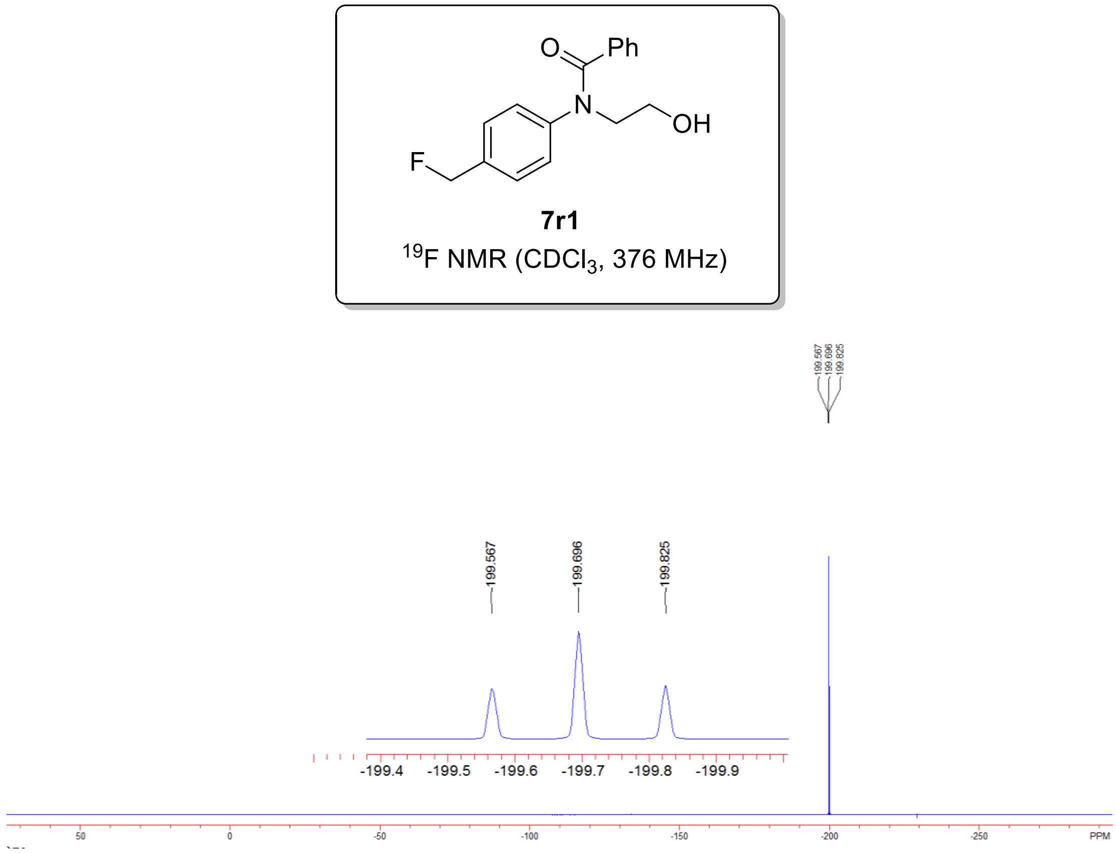

Supplementary Figure 182. ^{19}**F NMR Spectrum of 7r1**

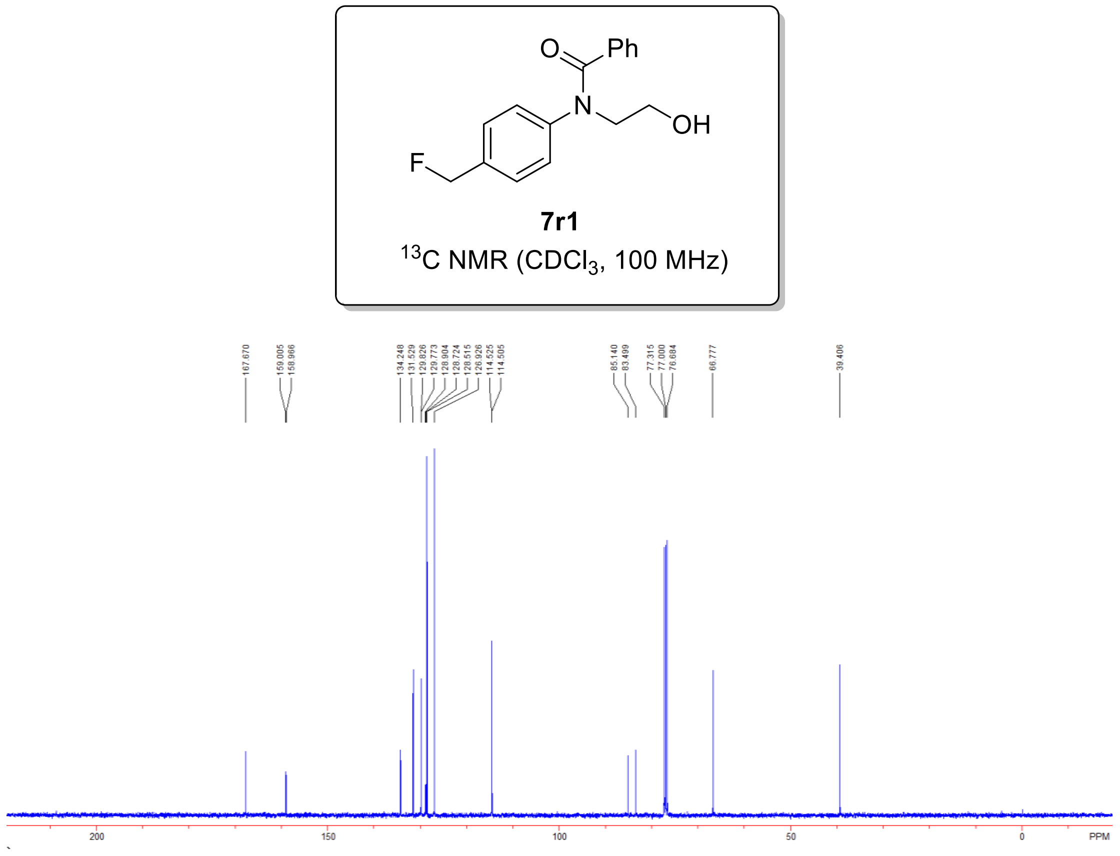

Supplementary Figure 183. ^{13}C NMR Spectrum of 7r1

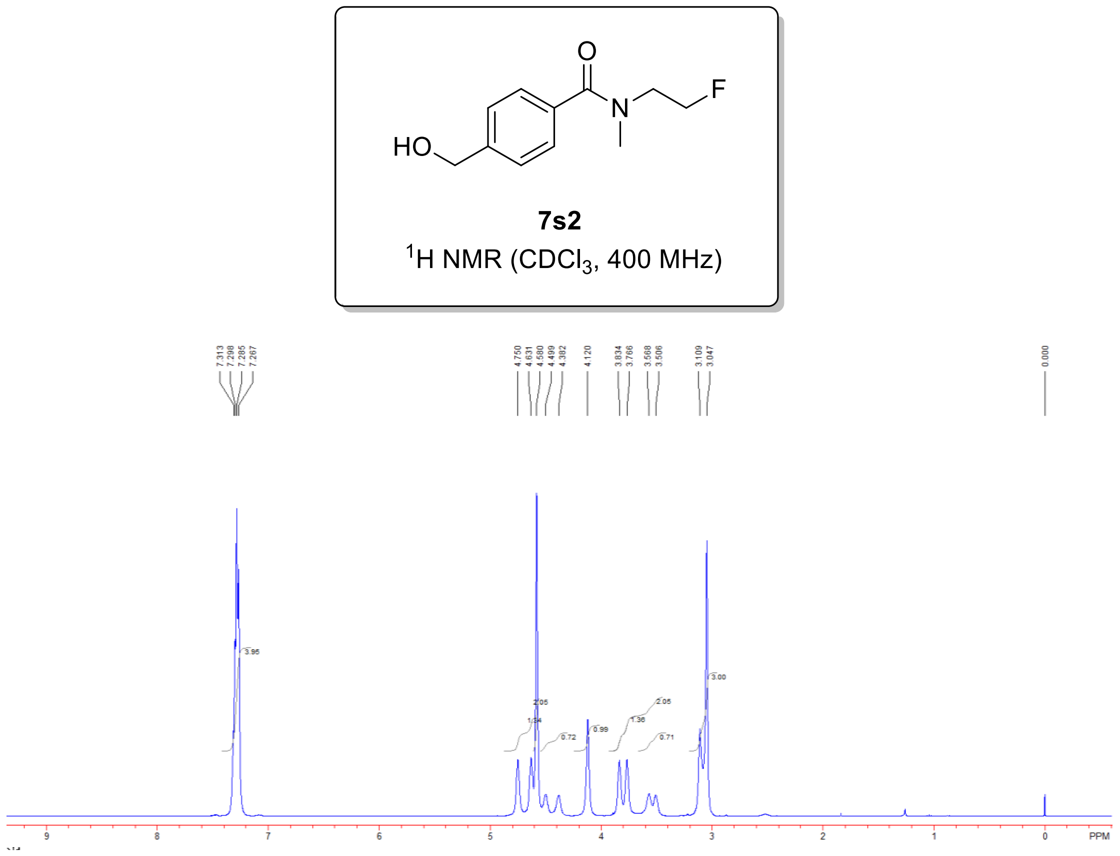

Supplementary Figure 184. ^{1}H NMR Spectrum of 7s2

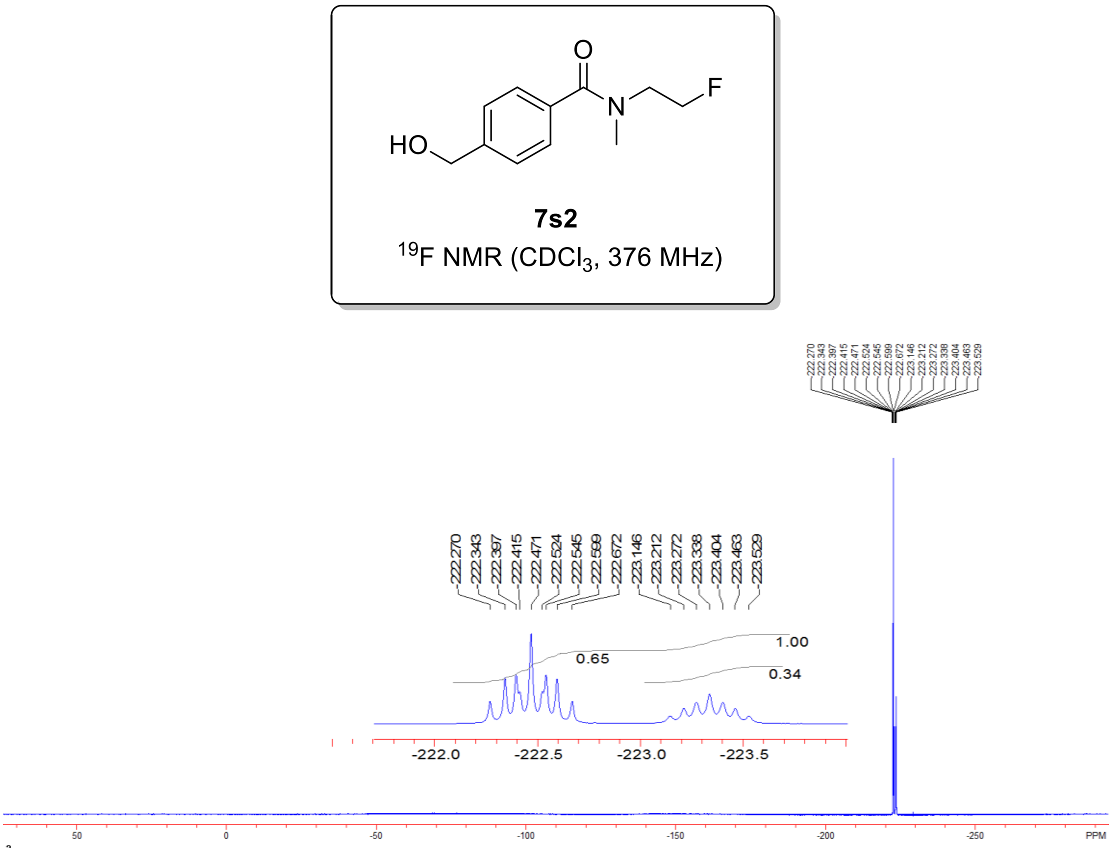

Supplementary Figure 185. ^{19}F NMR Spectrum of 7s2

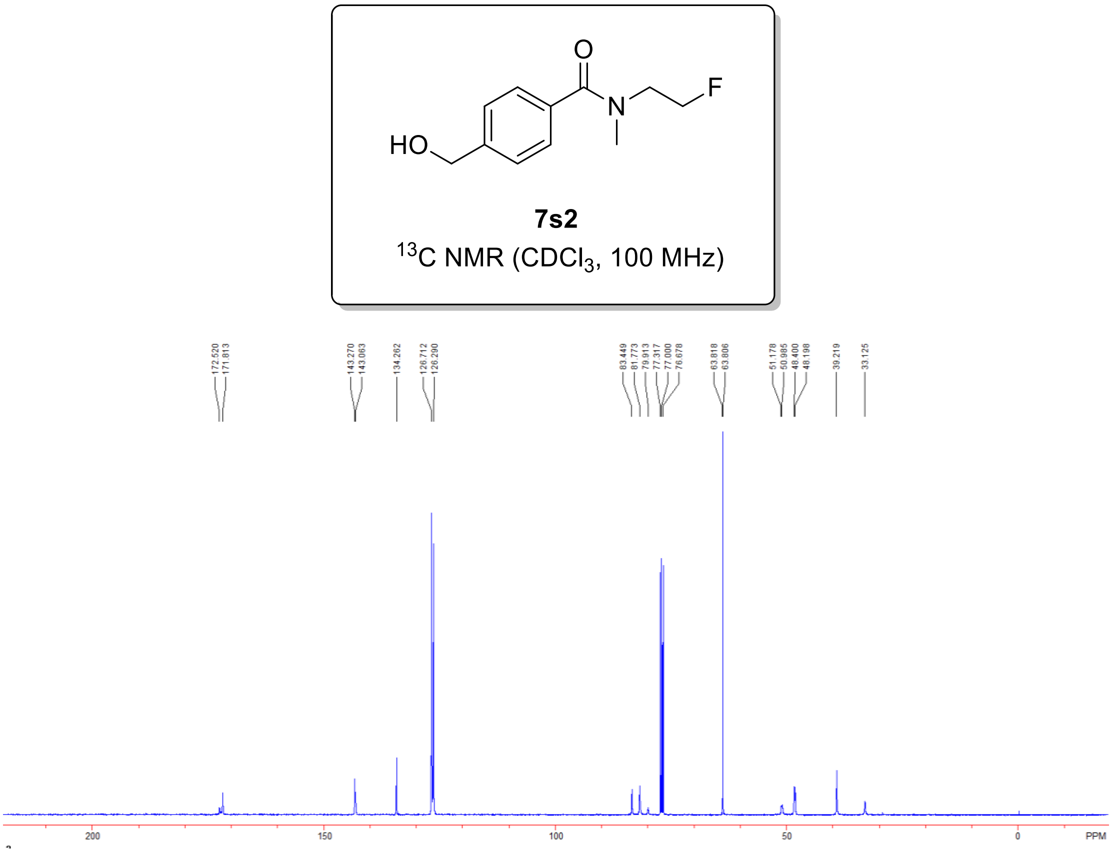

Supplementary Figure 186. ^{13}C NMR Spectrum of 7s2

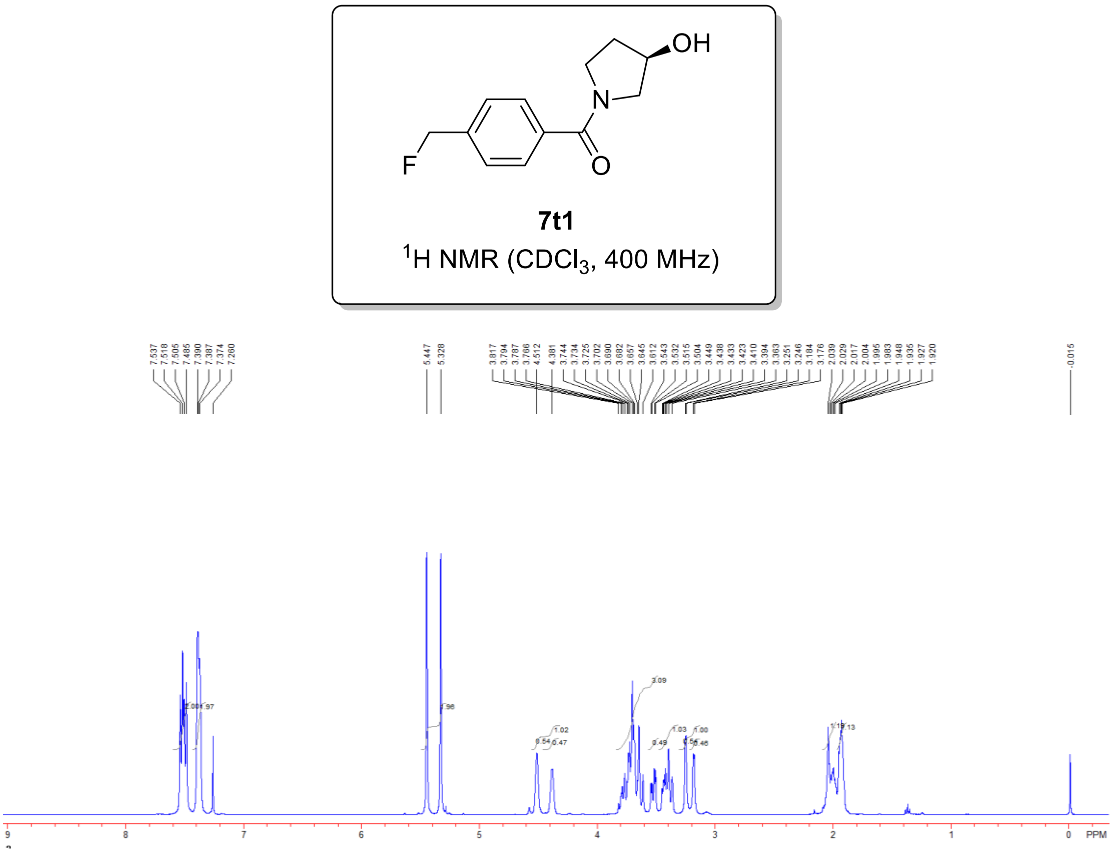

Supplementary Figure 187. ^{1}H NMR Spectrum of 7t1

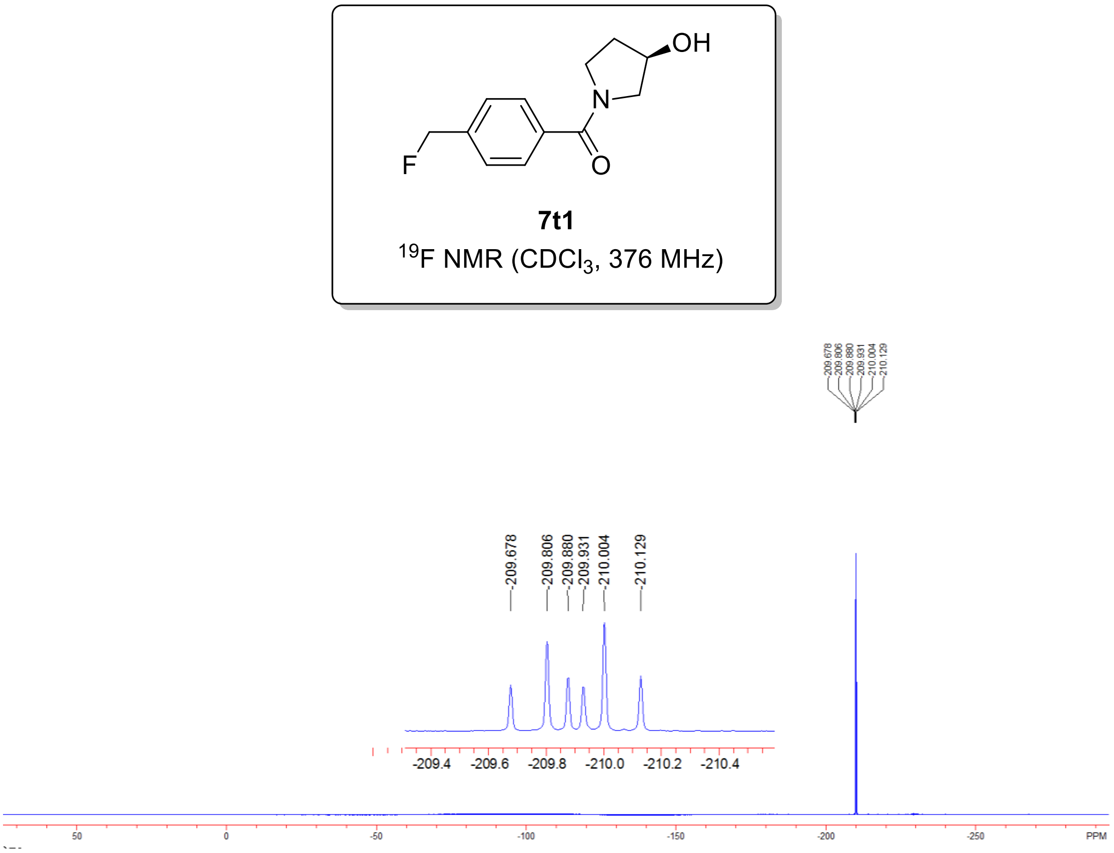

Supplementary Figure 188. ^{19}F NMR Spectrum of 7t1

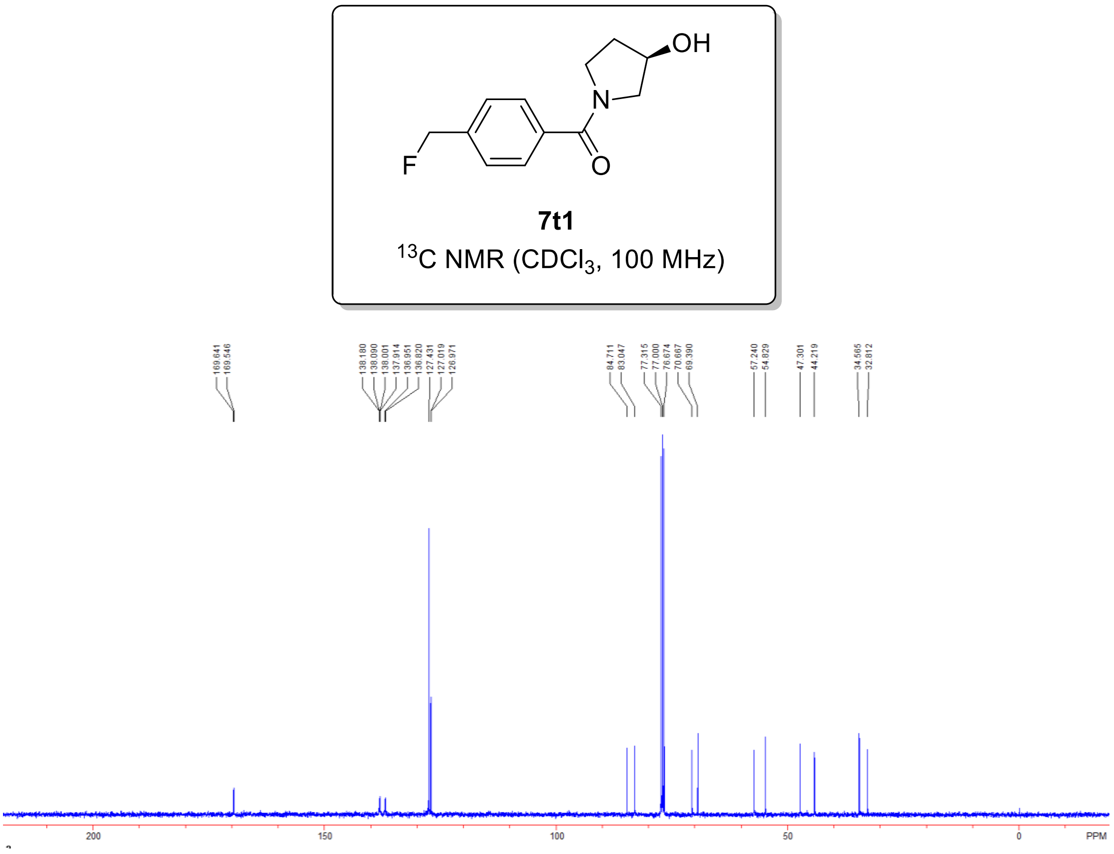

Supplementary Figure 189. ^{13}C NMR Spectrum of 7t1

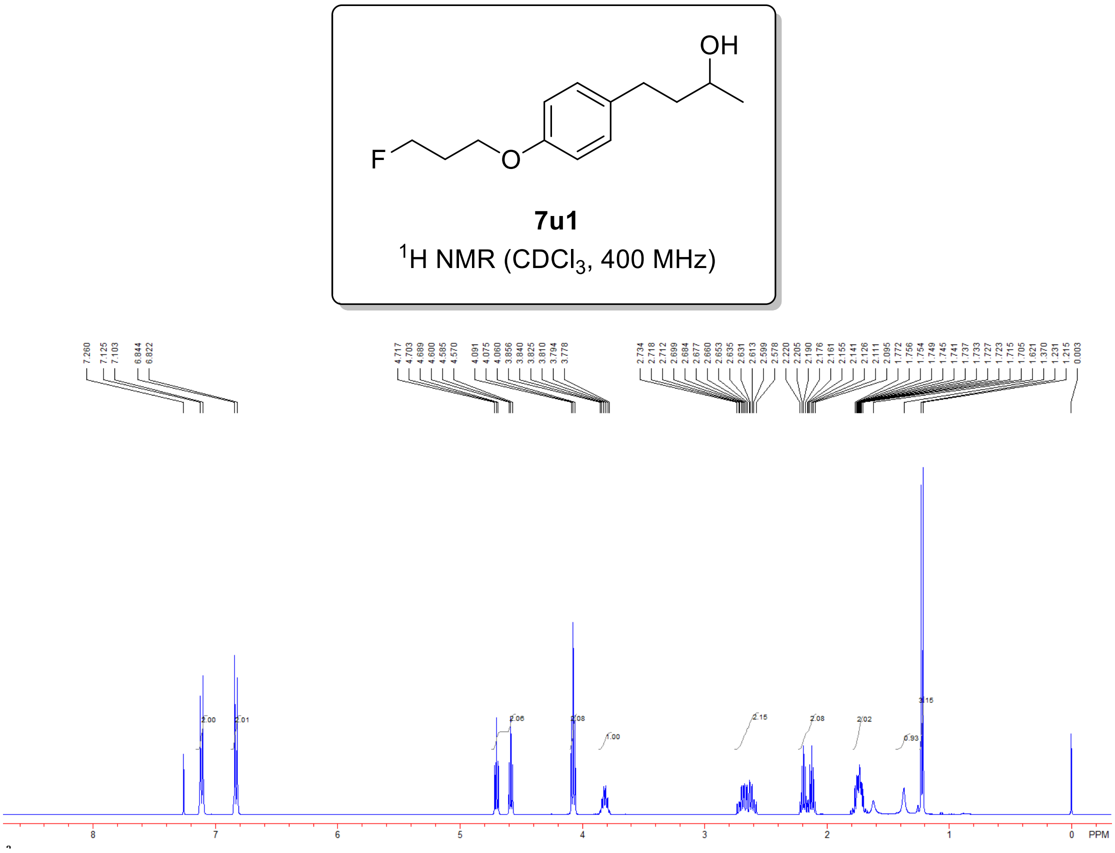

Supplementary Figure 190. ^{1}H NMR Spectrum of 7u1

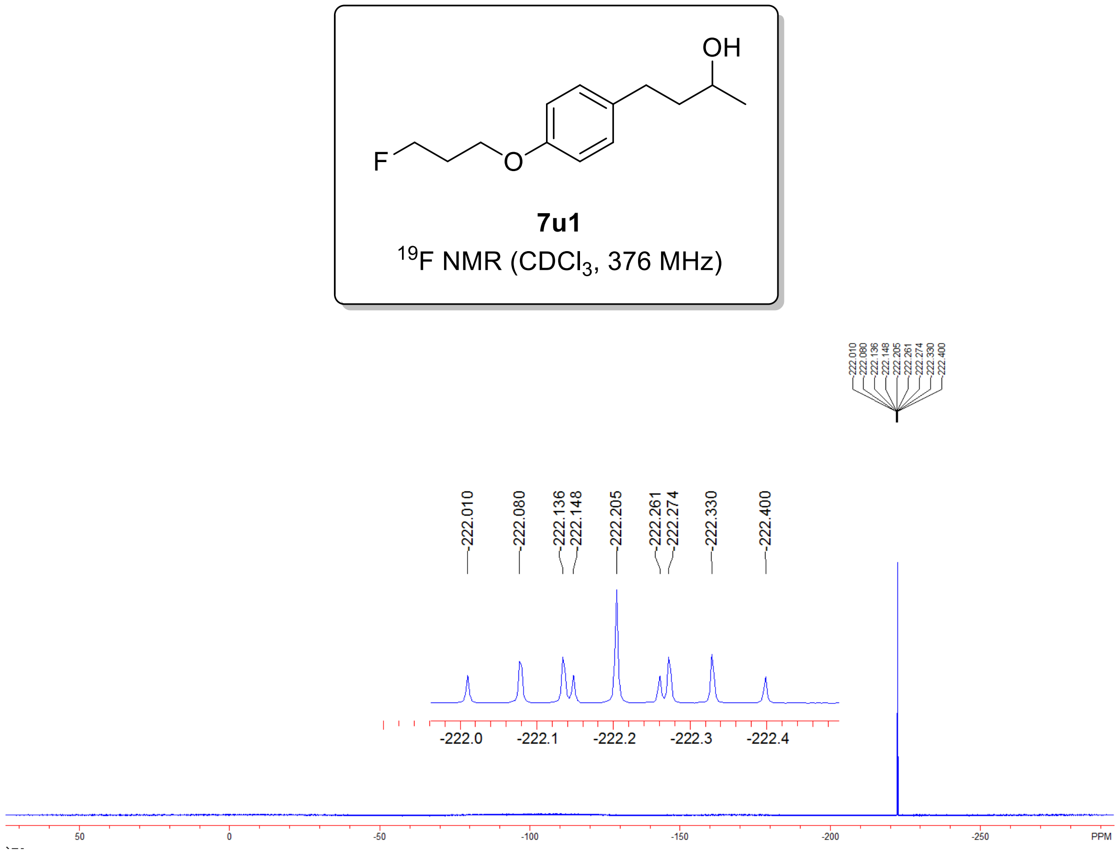

Supplementary Figure 191. ^{19}**F NMR Spectrum of 7u1**

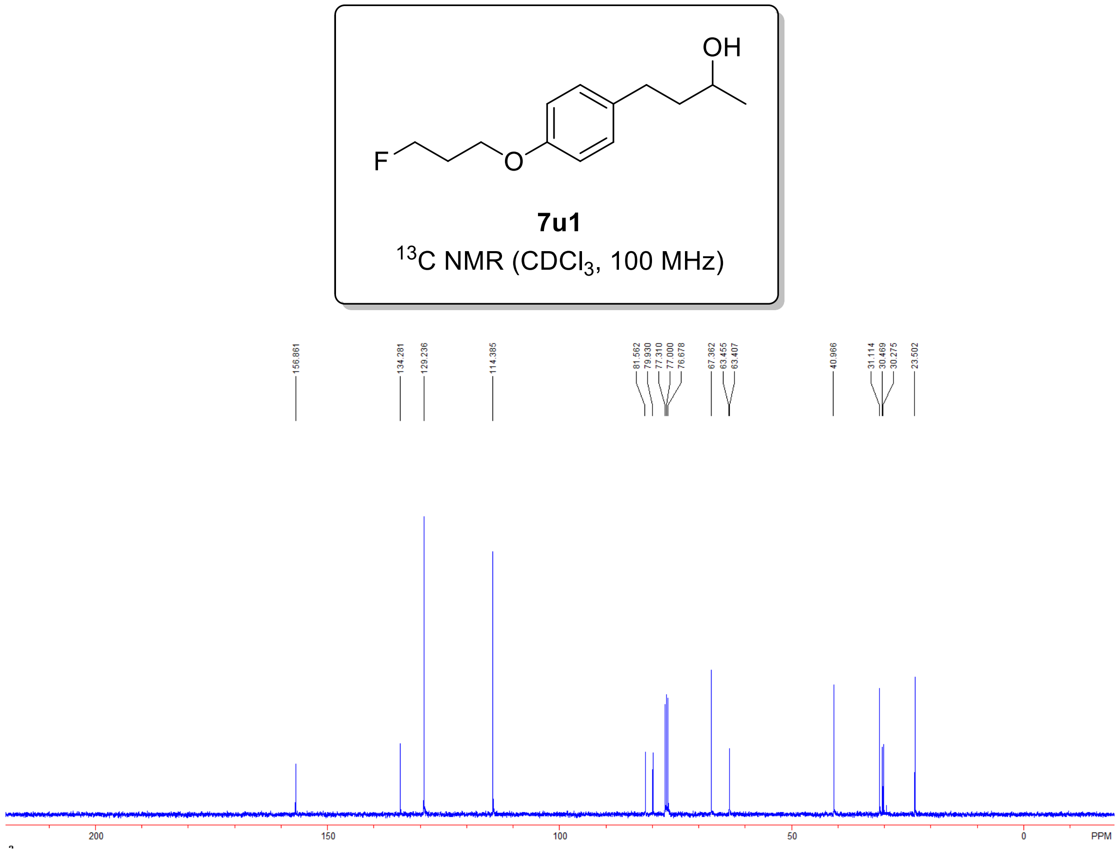

Supplementary Figure 192. ^{13}**C NMR Spectrum of 7u1**

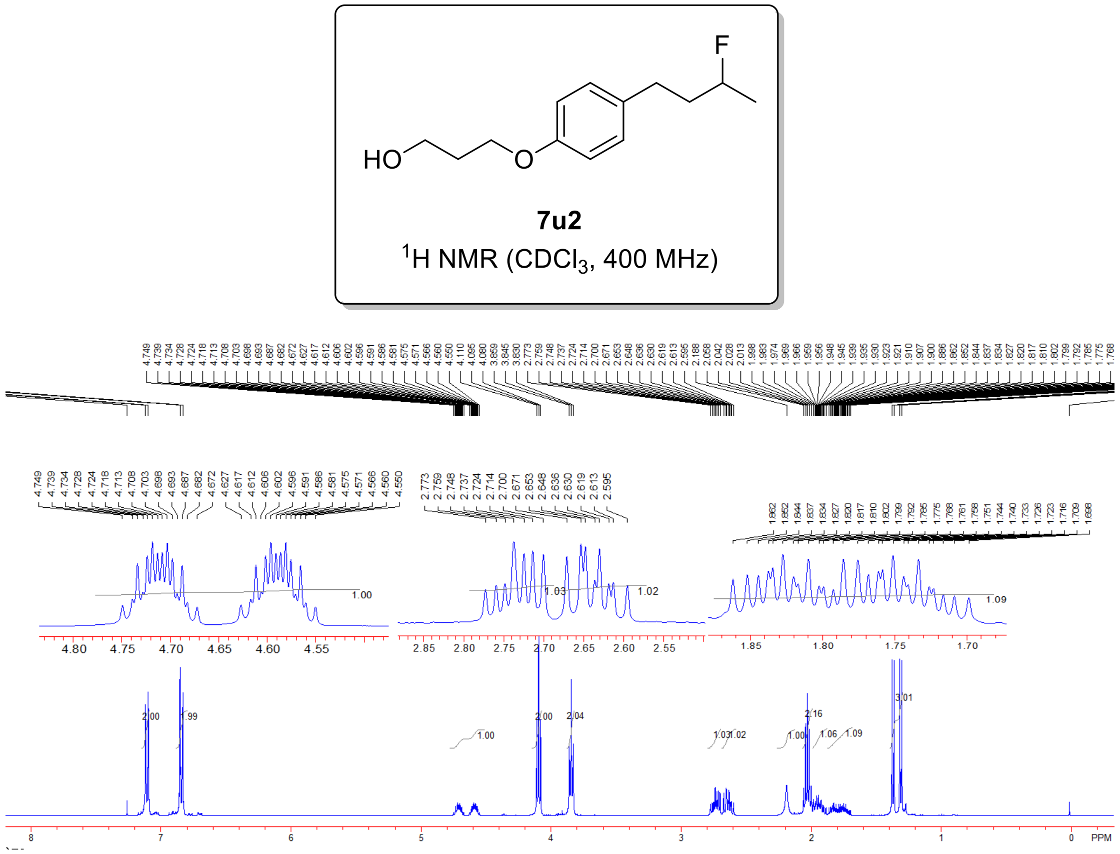

Supplementary Figure 193. ^{1}H NMR Spectrum of 7u2

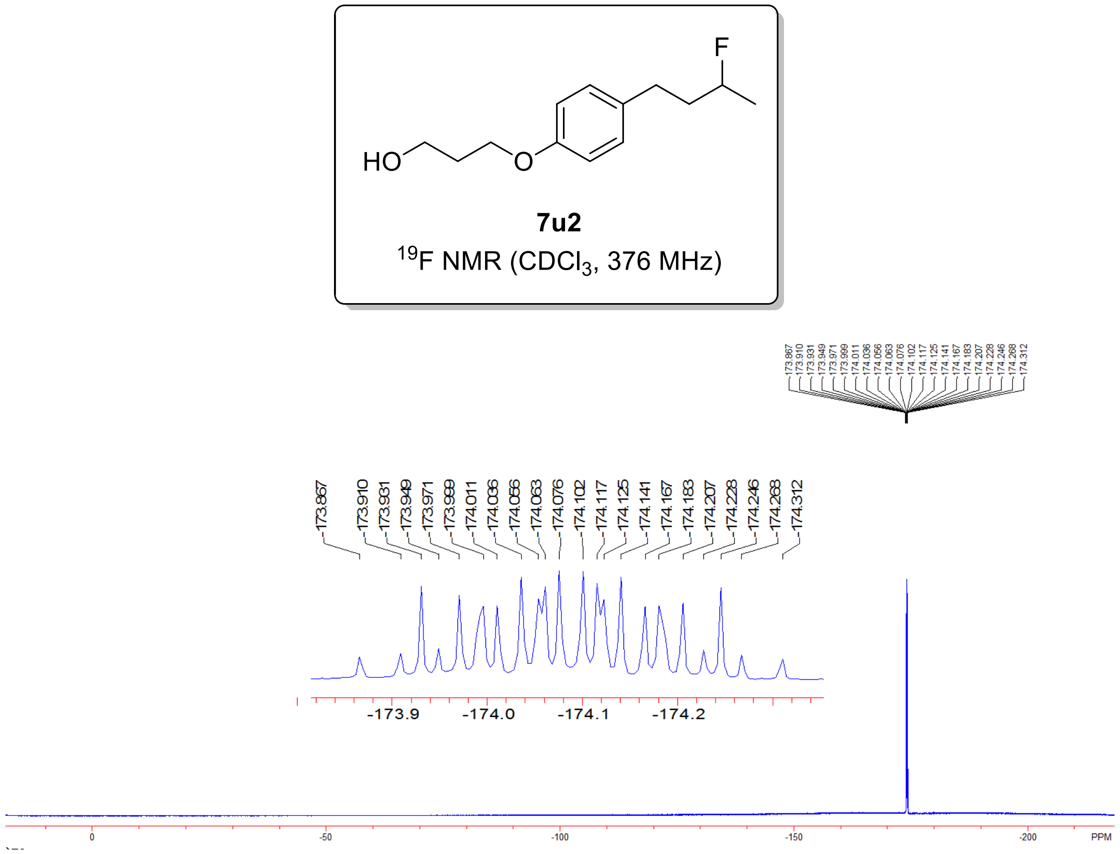

Supplementary Figure 194. ^{19}F NMR Spectrum of 7u2

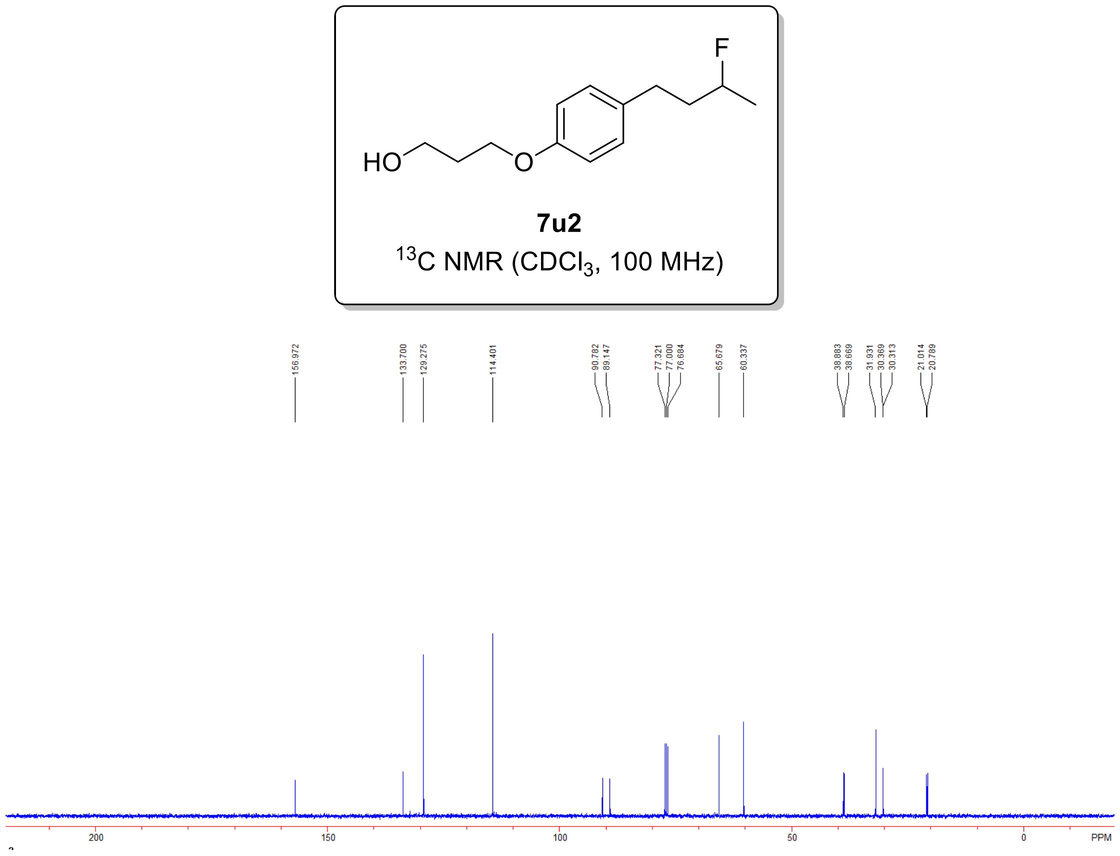

Supplementary Figure 195. ^{13}**C NMR Spectrum of 7u2**

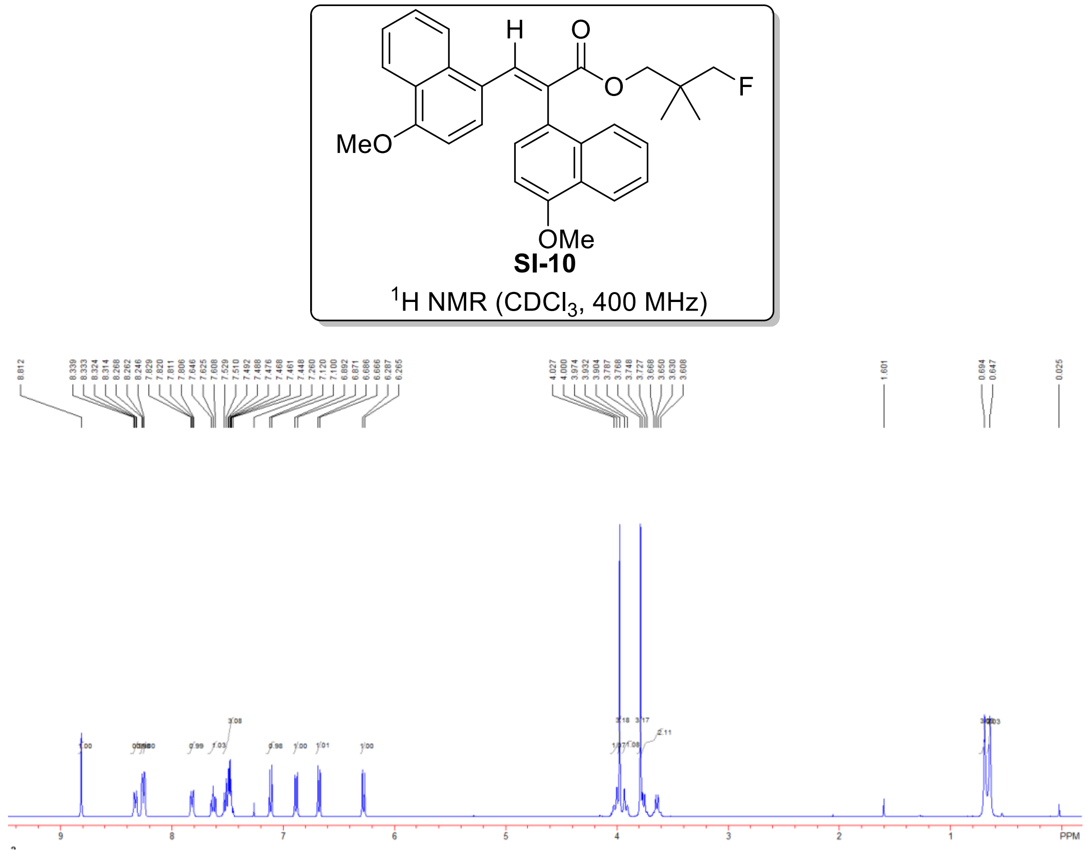

Supplementary Figure 196. ^{1}**H NMR Spectrum of SI-10**

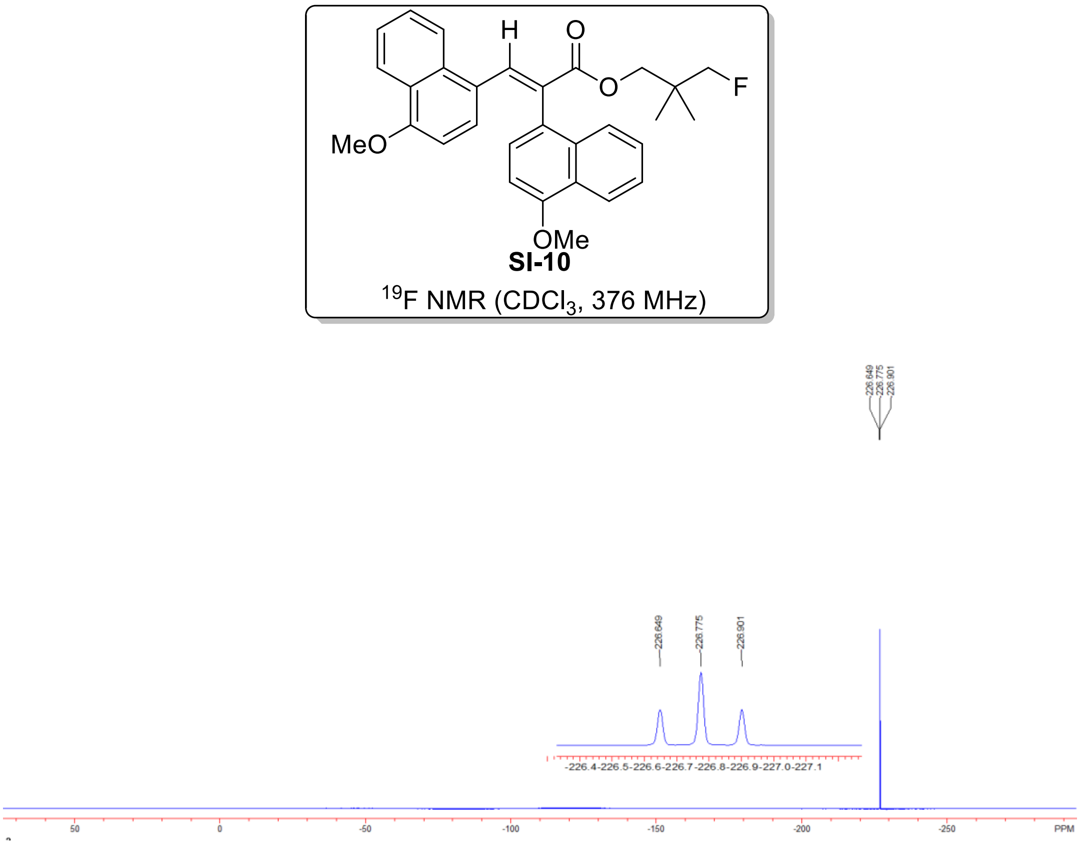

Supplementary Figure 197. ^{19}**F NMR Spectrum of SI-10**

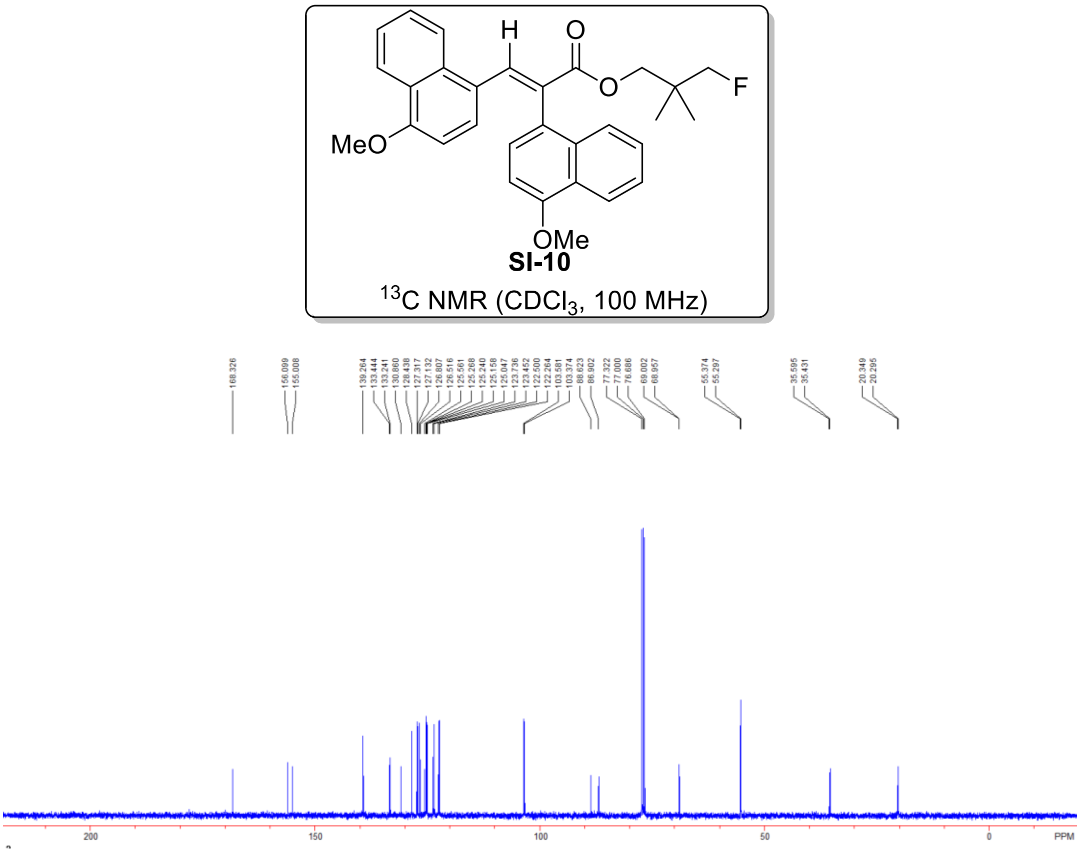

Supplementary Figure 198. ^{13}**C NMR Spectrum of SI-10**

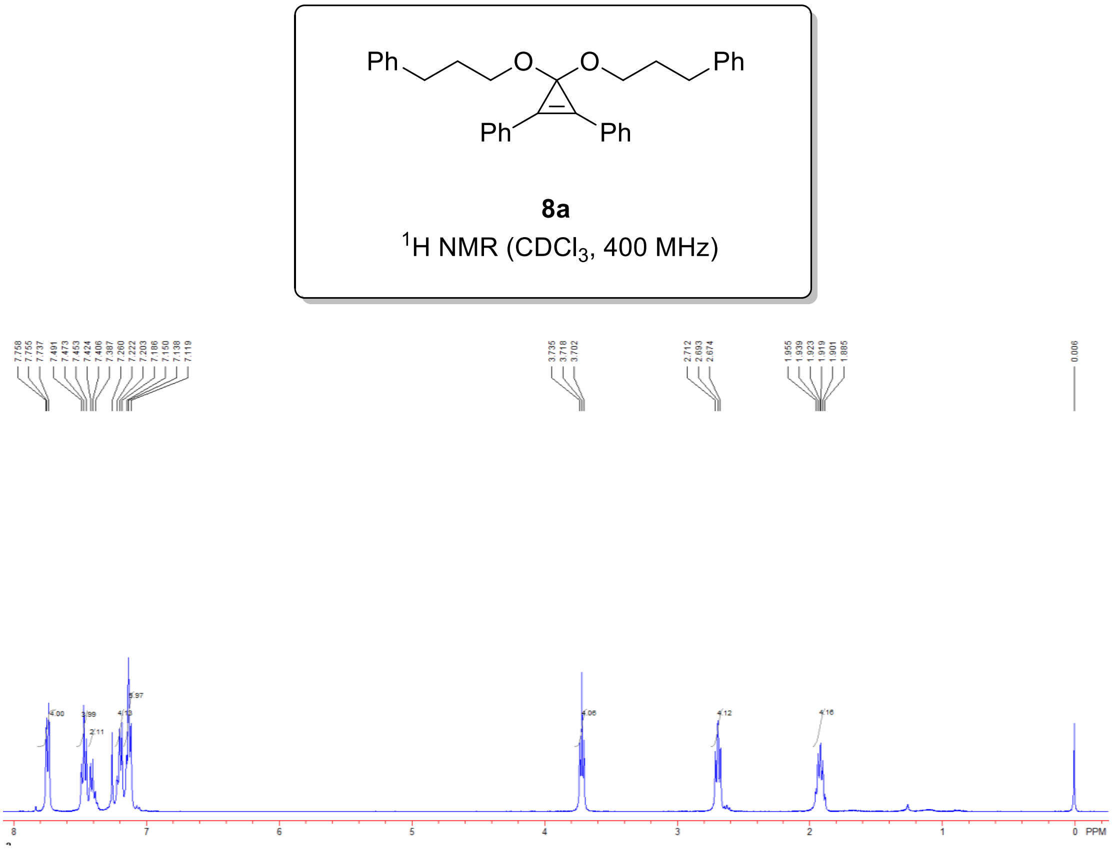

Supplementary Figure 199. ^{1}H NMR Spectrum of 8a

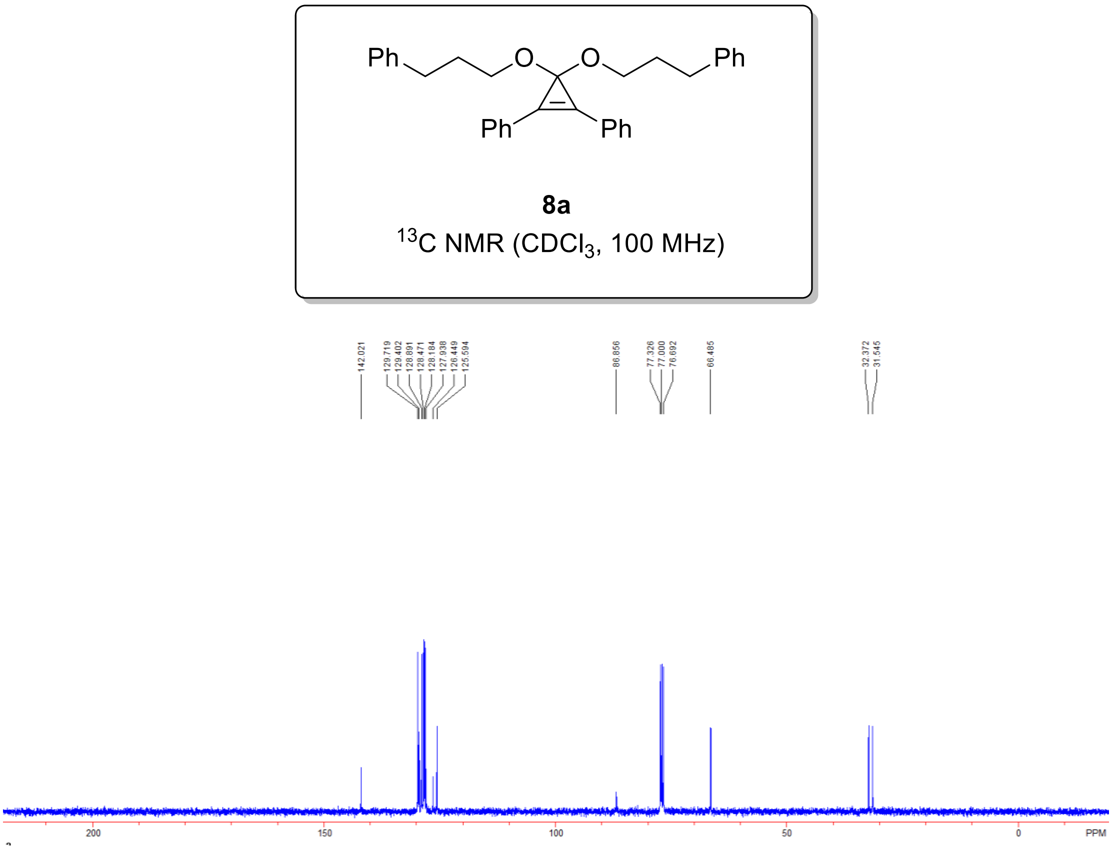

Supplementary Figure 200. ^{13}C NMR Spectrum of 8a

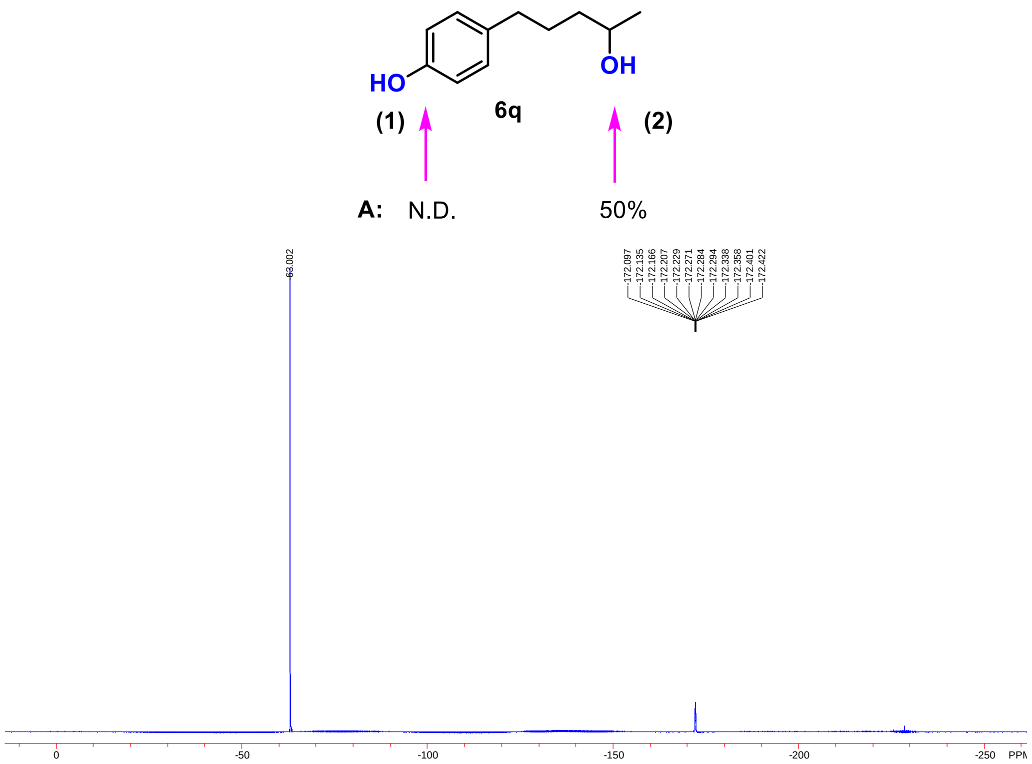

Supplementary Figure 201. ^{19}**F NMR Spectrum of (6q + CpFluor 1c) (Crude)**

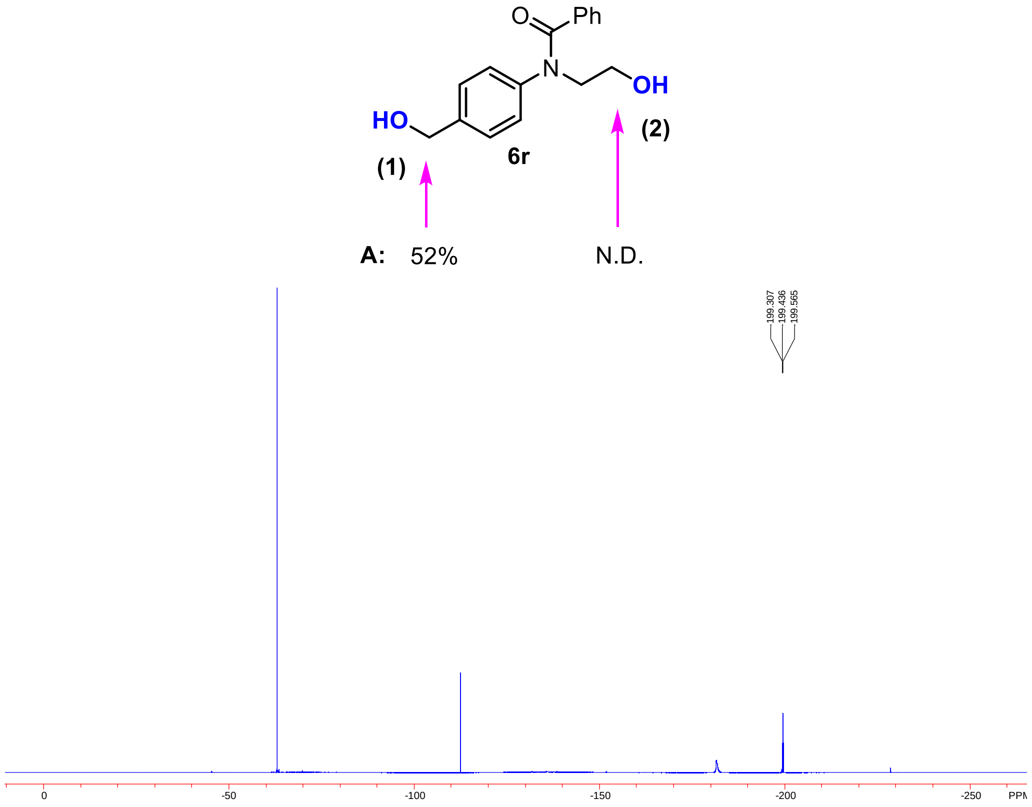

Supplementary Figure 202. ^{19}**F NMR Spectrum of (6r + CpFluor 1c) (Crude)**

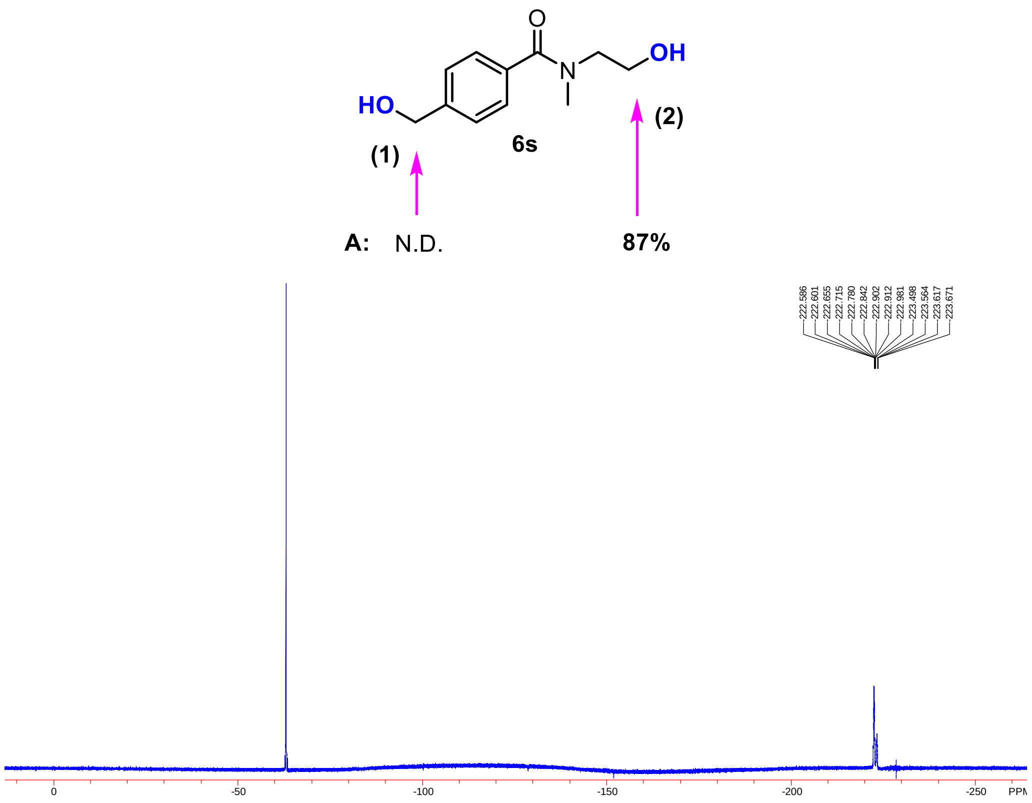

Supplementary Figure 203. ^{19}F NMR Spectrum of (6s + CpFluor 1c) (Crude)

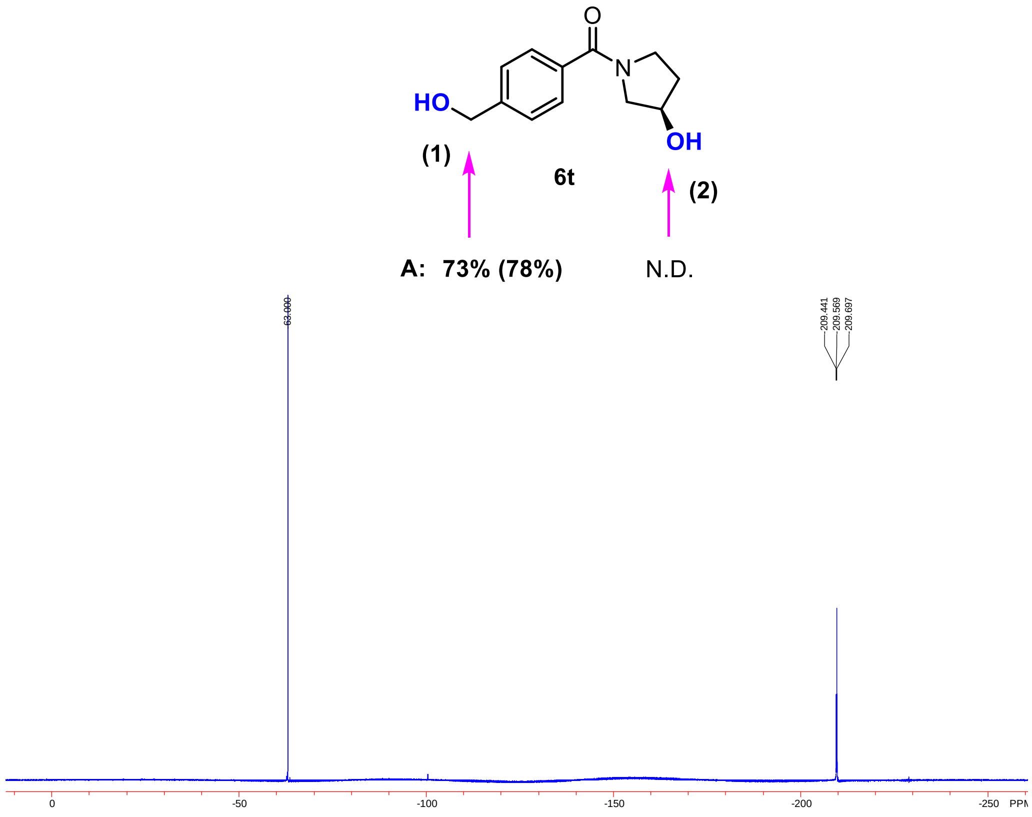

Supplementary Figure 204. ^{19}F NMR Spectrum of (6t + CpFluor 1c) (Crude)

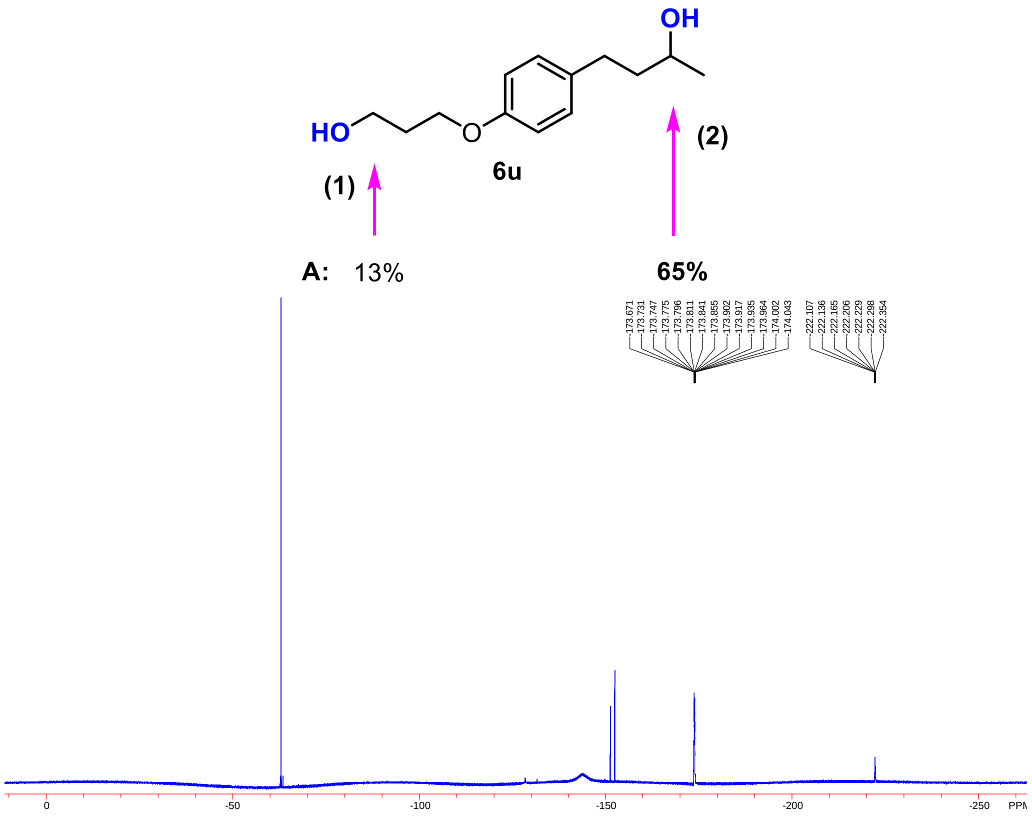

Supplementary Figure 205. ^{19}**F NMR Spectrum of (6u + CpFluor 1c) (Crude)**

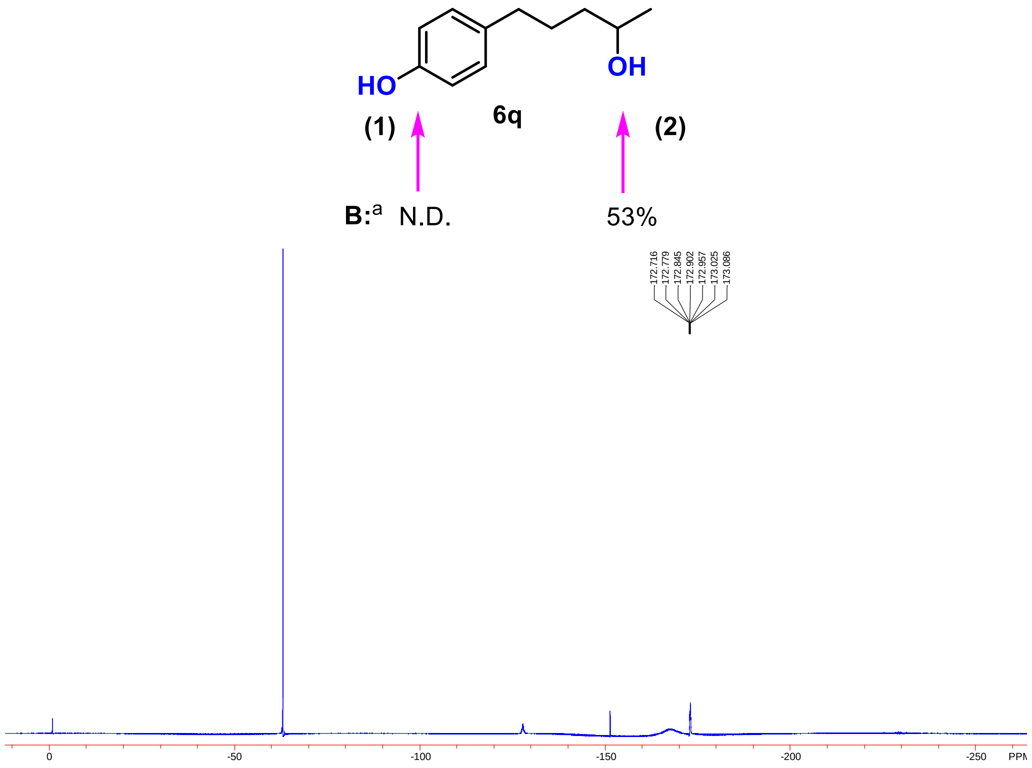

Supplementary Figure 206. ^{19}**F NMR Spectrum of (6q + DAST) (Crude)**

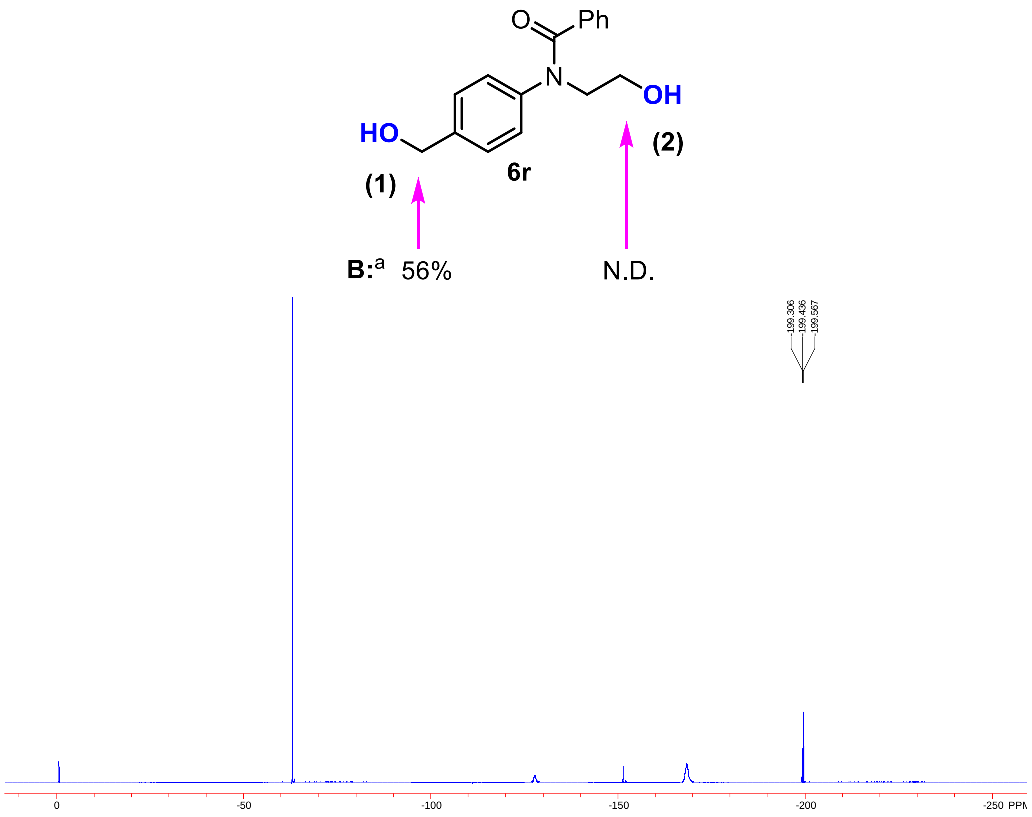

Supplementary Figure 207. ^{19}**F NMR Spectrum of (6r + DAST) (Crude)**

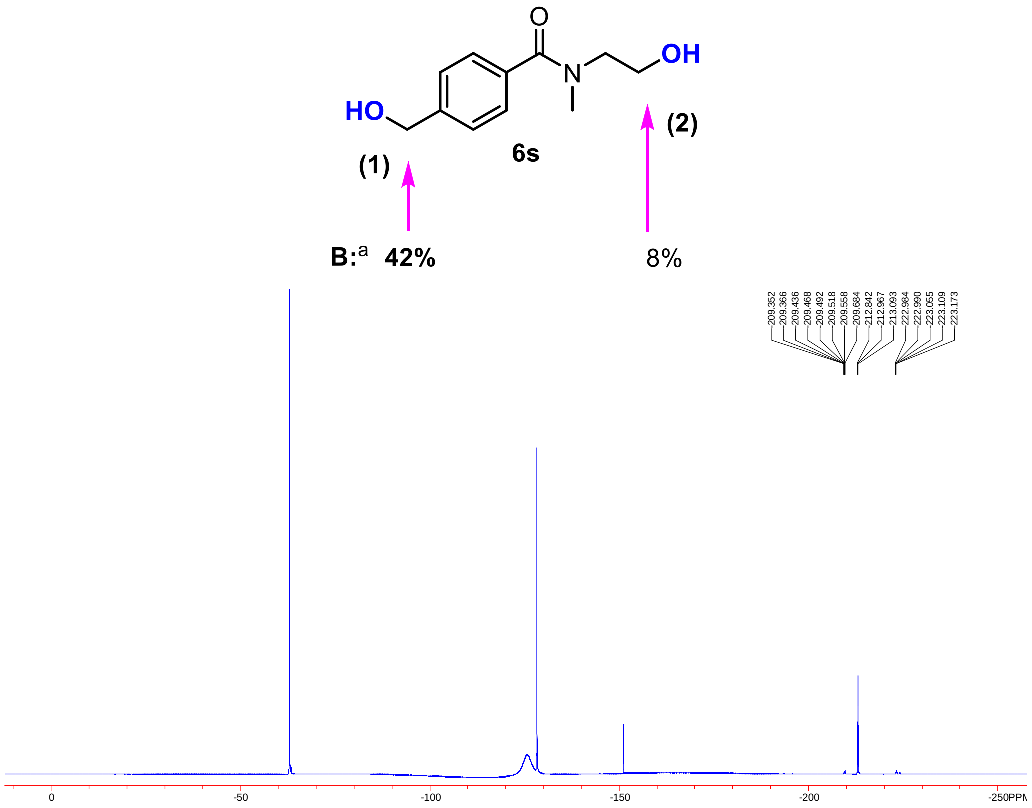

Supplementary Figure 208. ^{19}**F NMR Spectrum of (6s + DAST) (Crude)**

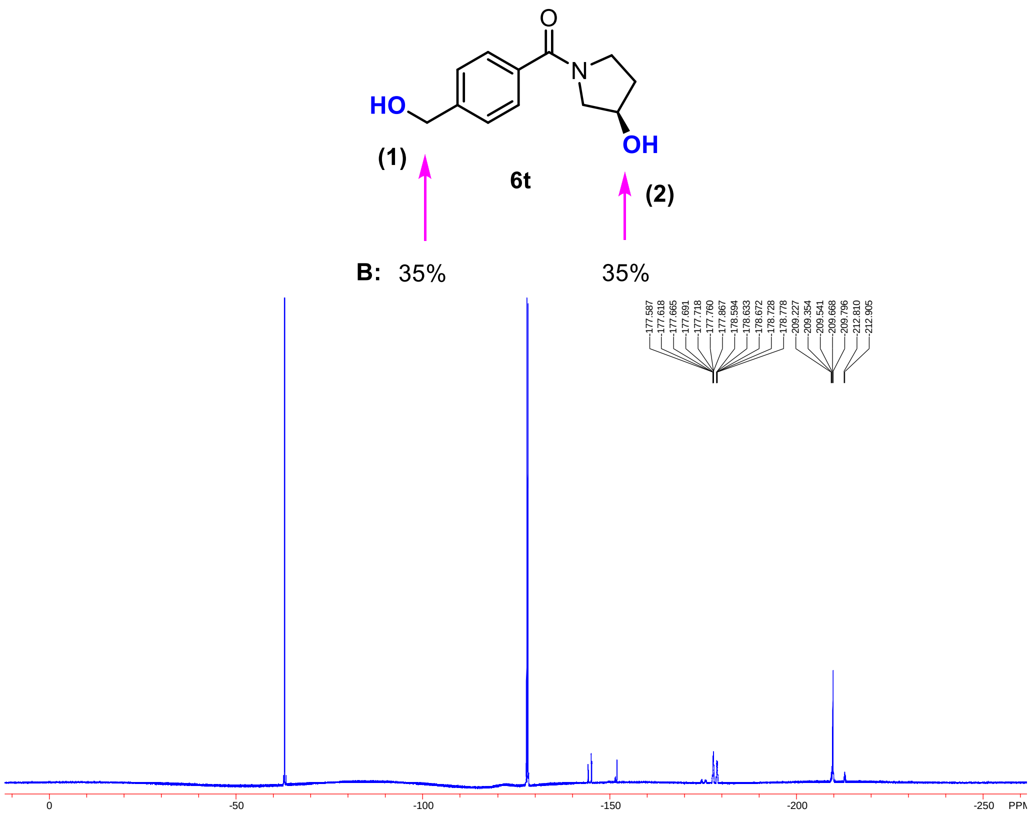

Supplementary Figure 209. ^{19}F NMR Spectrum of (6t + DAST) (Crude)

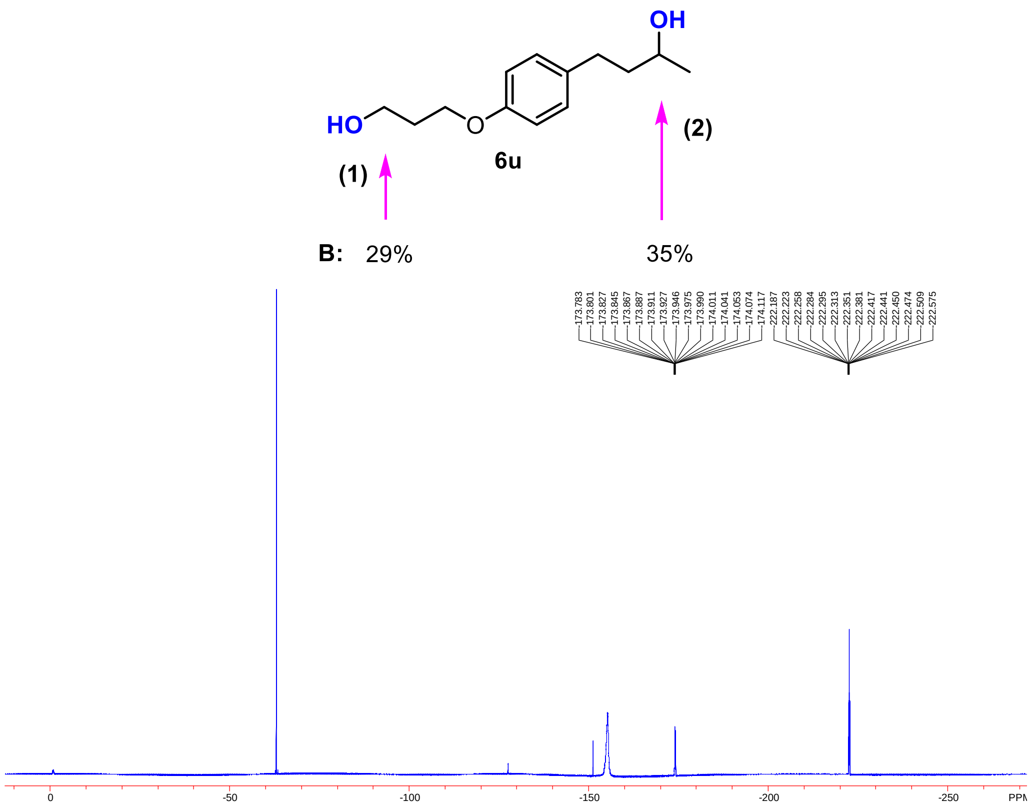

Supplementary Figure 210. ^{19}F NMR Spectrum of (6u + DAST) (Crude)

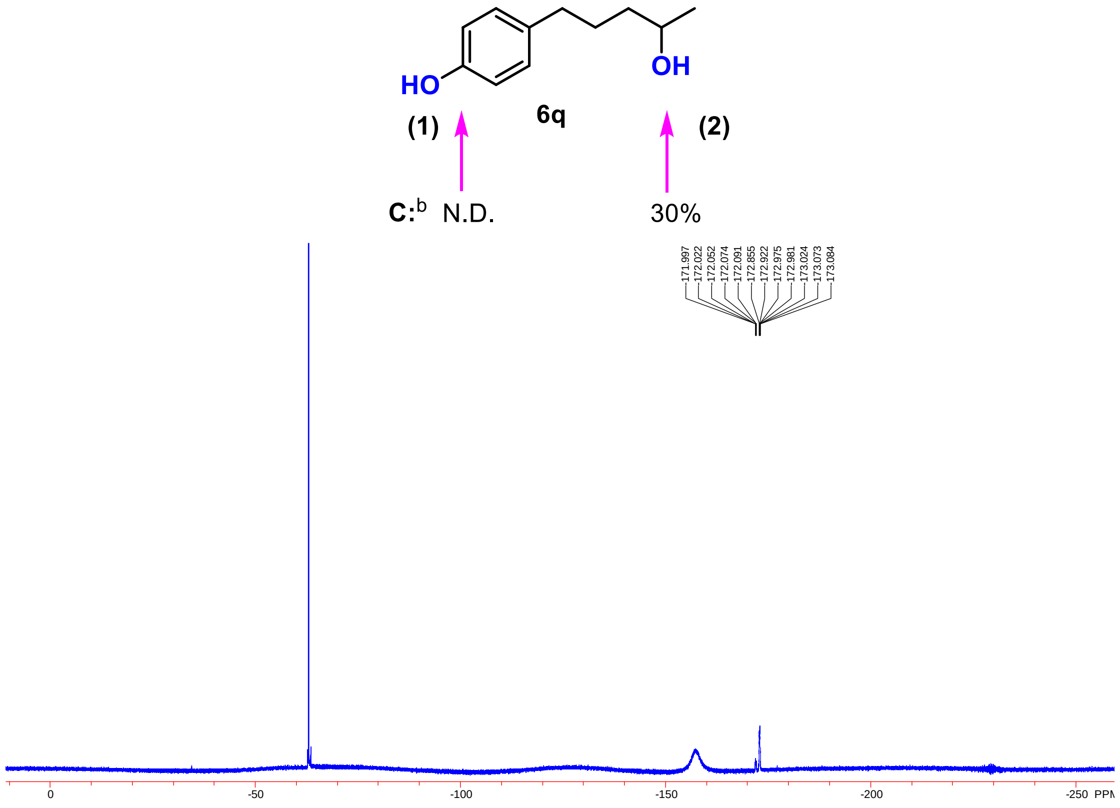

Supplementary Figure 211. ^{19}F NMR Spectrum of (6q + PhenoFluor) (Crude)

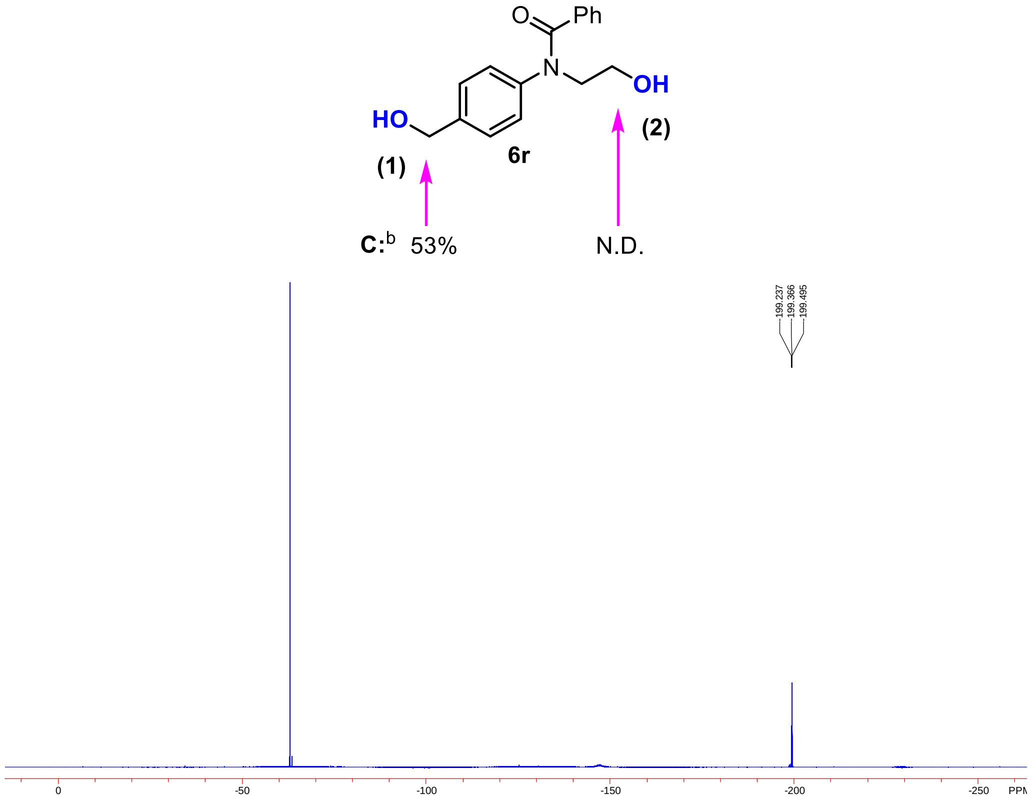

Supplementary Figure 212. ^{19}F NMR Spectrum of (6r + PhenoFluor) (Crude)

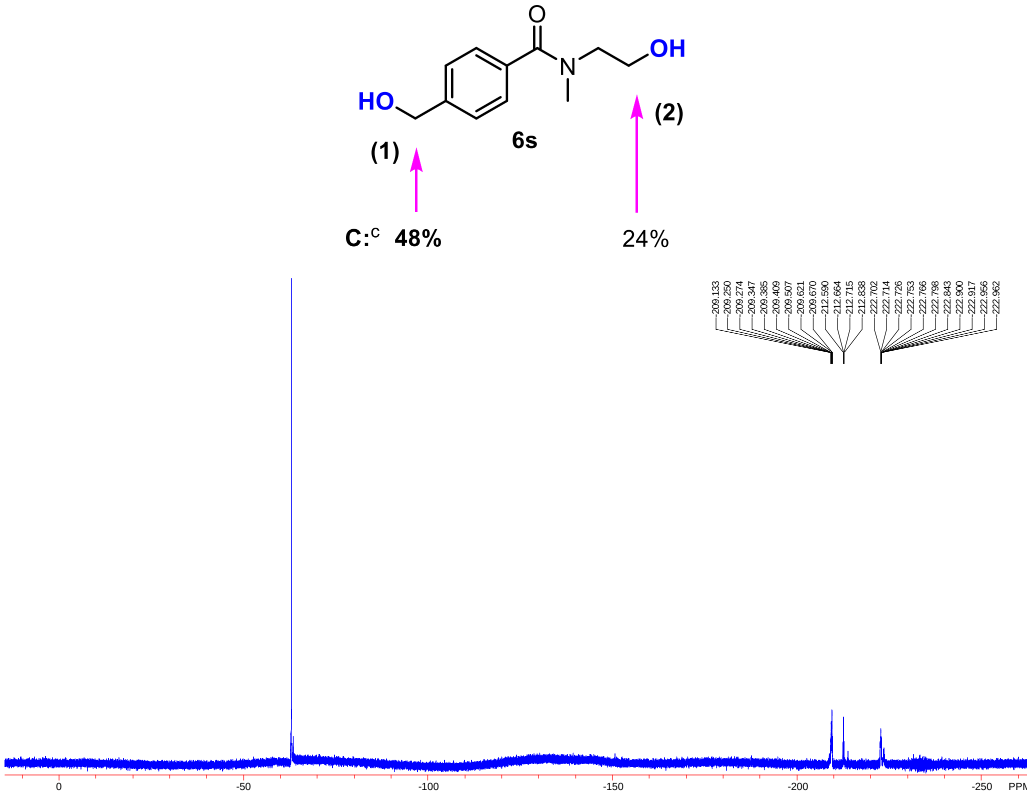

Supplementary Figure 213. ^{19}**F NMR Spectrum of (6s + PhenoFluor) (Crude)**

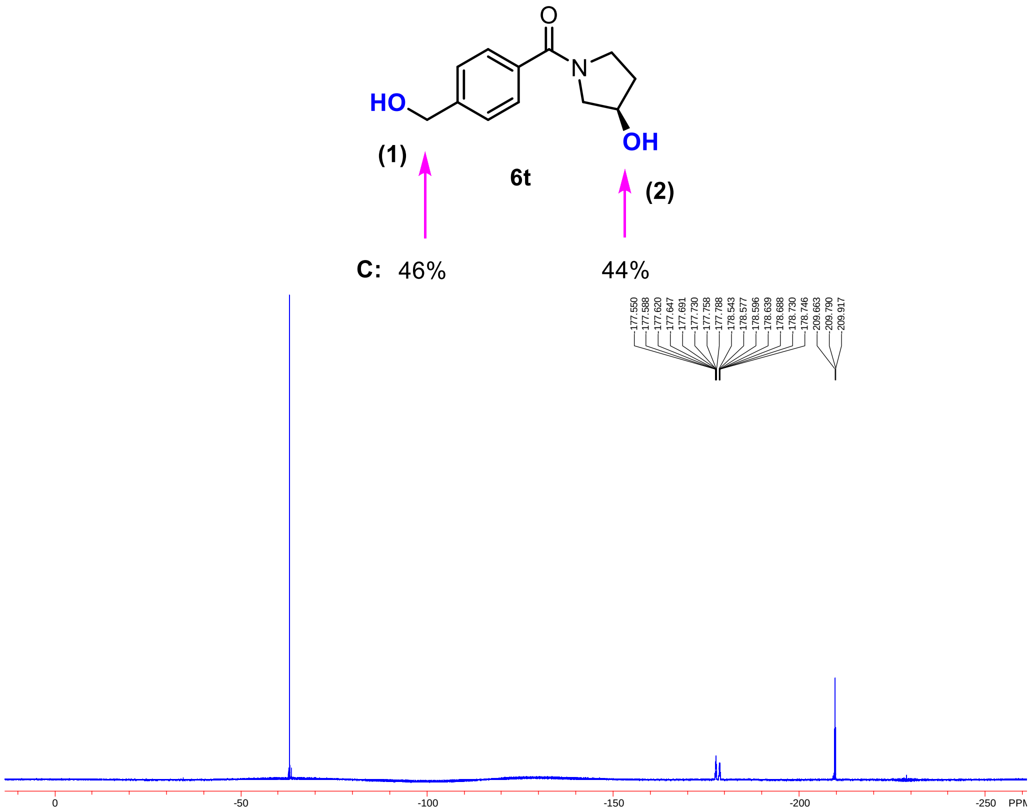

Supplementary Figure 214. ^{19}**F NMR Spectrum of (6t + PhenoFluor) (Crude)**

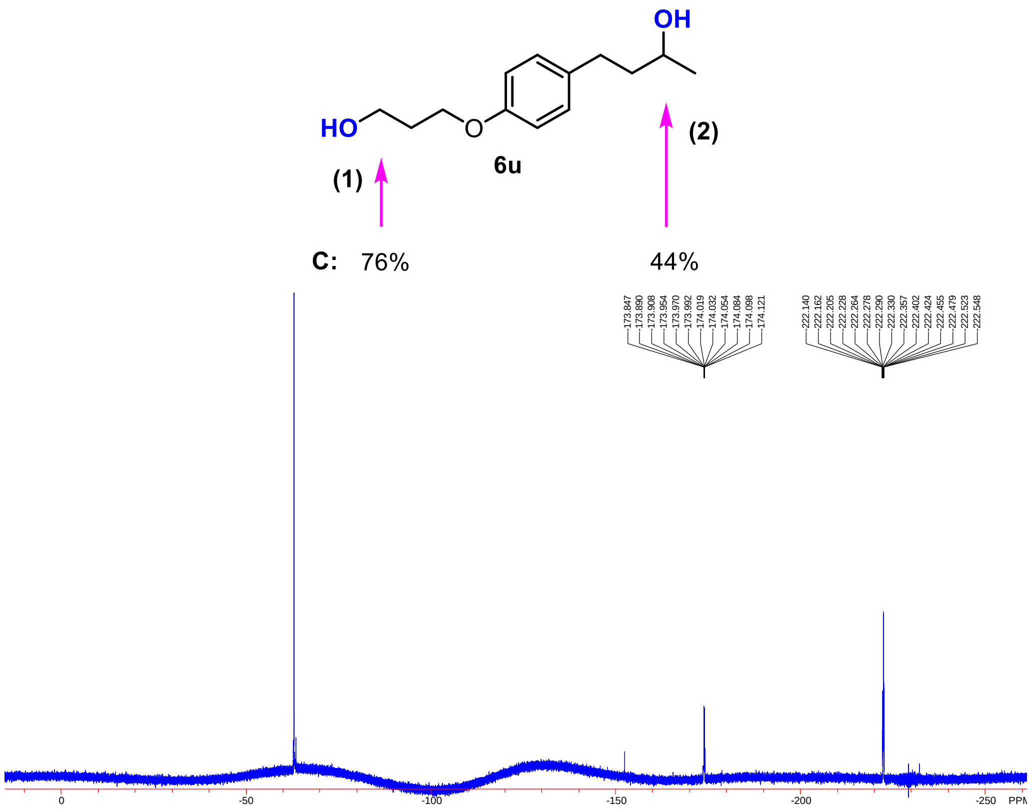

Supplementary Figure 215. ^{19}F NMR Spectrum of (6u + PhenoFluor) (Crude)

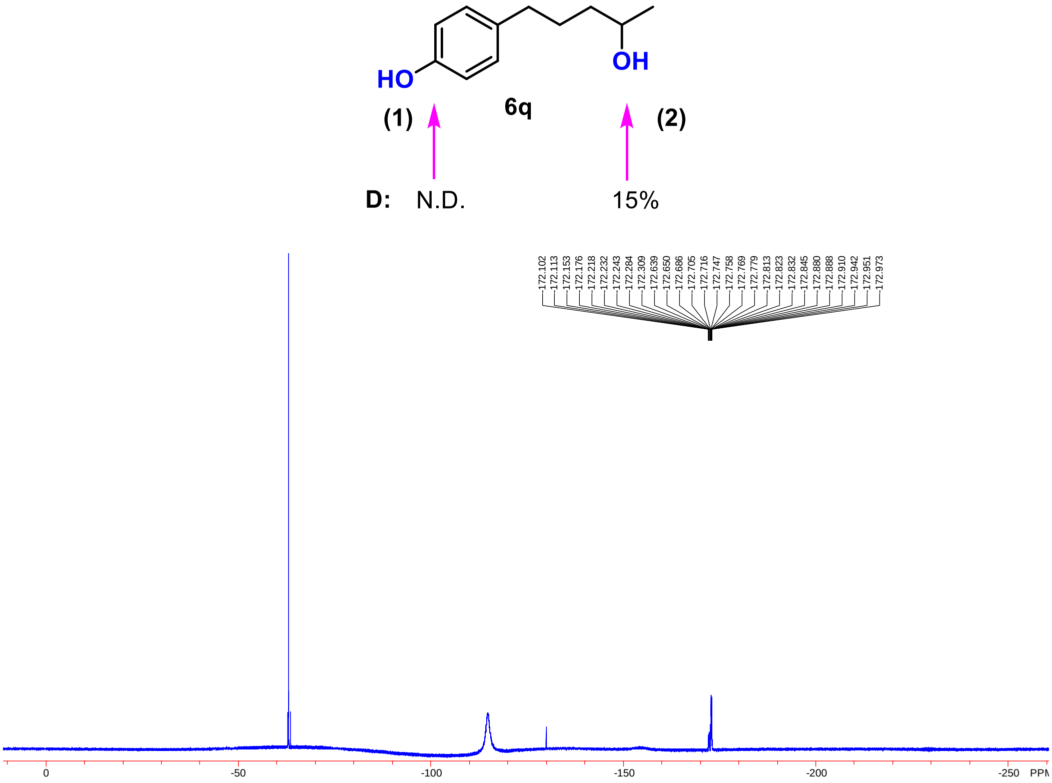

Supplementary Figure 216. ^{19}F NMR Spectrum of (6q + PyFluor) (Crude)

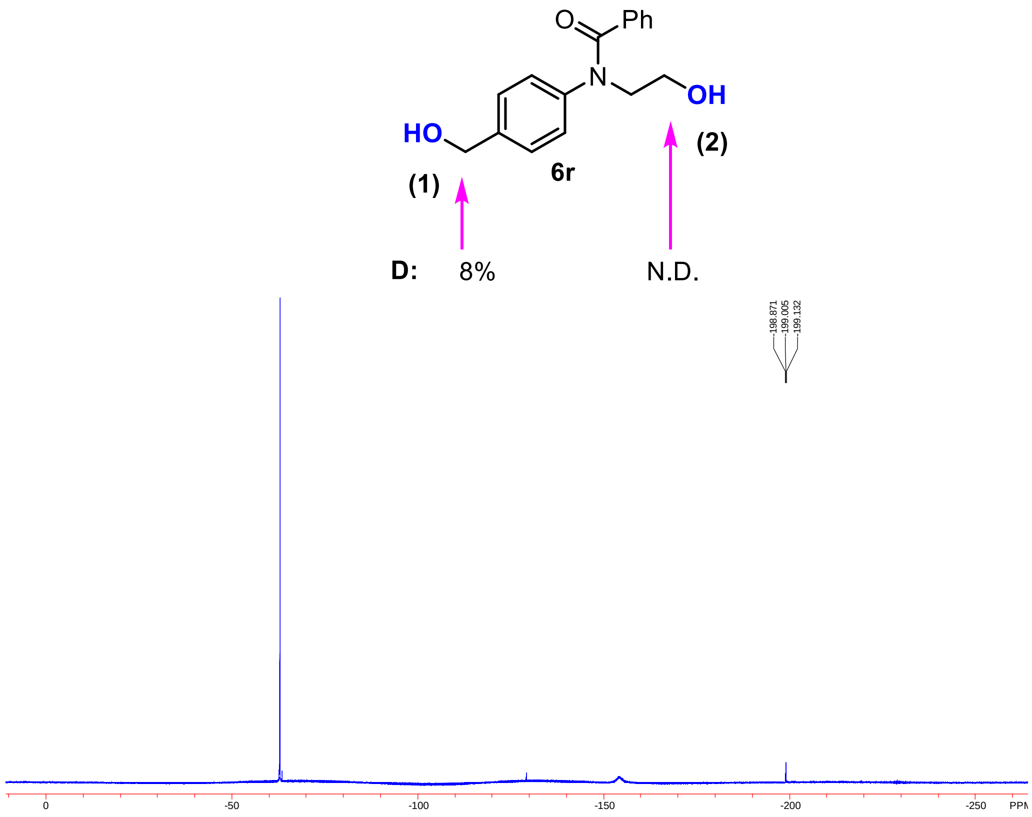

Supplementary Figure 217. ^{19}**F NMR Spectrum of (6r + PyFluor) (Crude)**

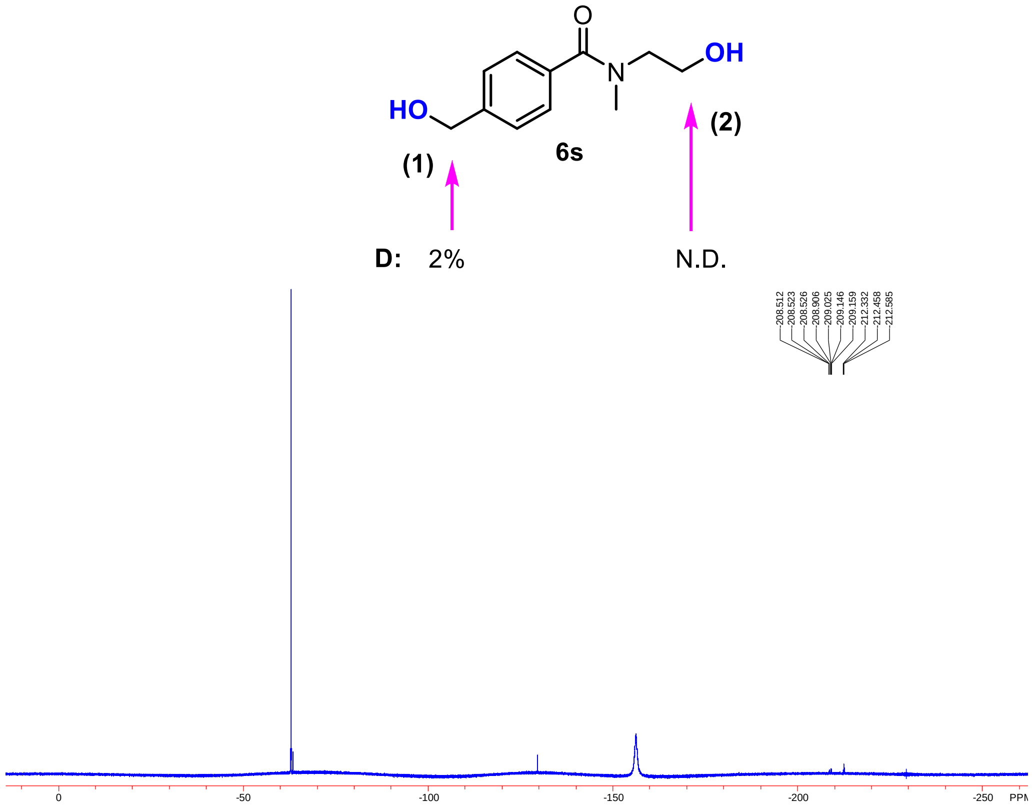

Supplementary Figure 218. ^{19}**F NMR Spectrum of (6s + PyFluor) (Crude)**

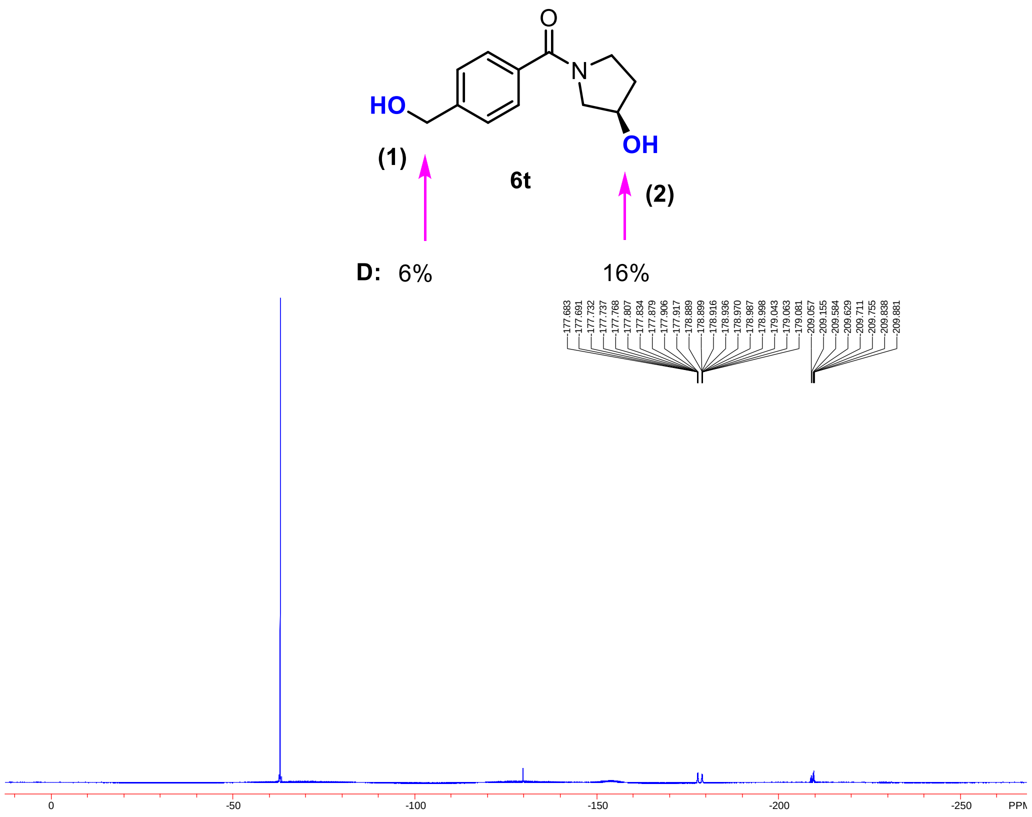

Supplementary Figure 219. ^{19}**F NMR Spectrum of (6t + PyFluor) (Crude)**

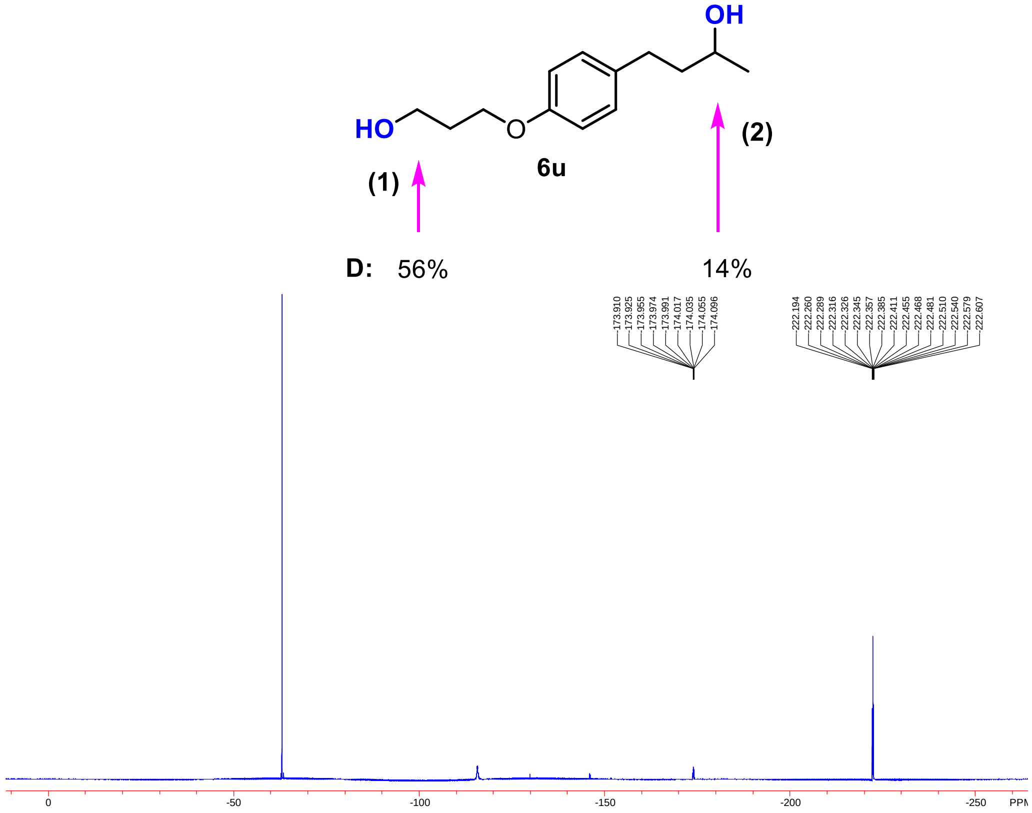

Supplementary Figure 220. ^{19}**F NMR Spectrum of (6u + PyFluor) (Crude)**

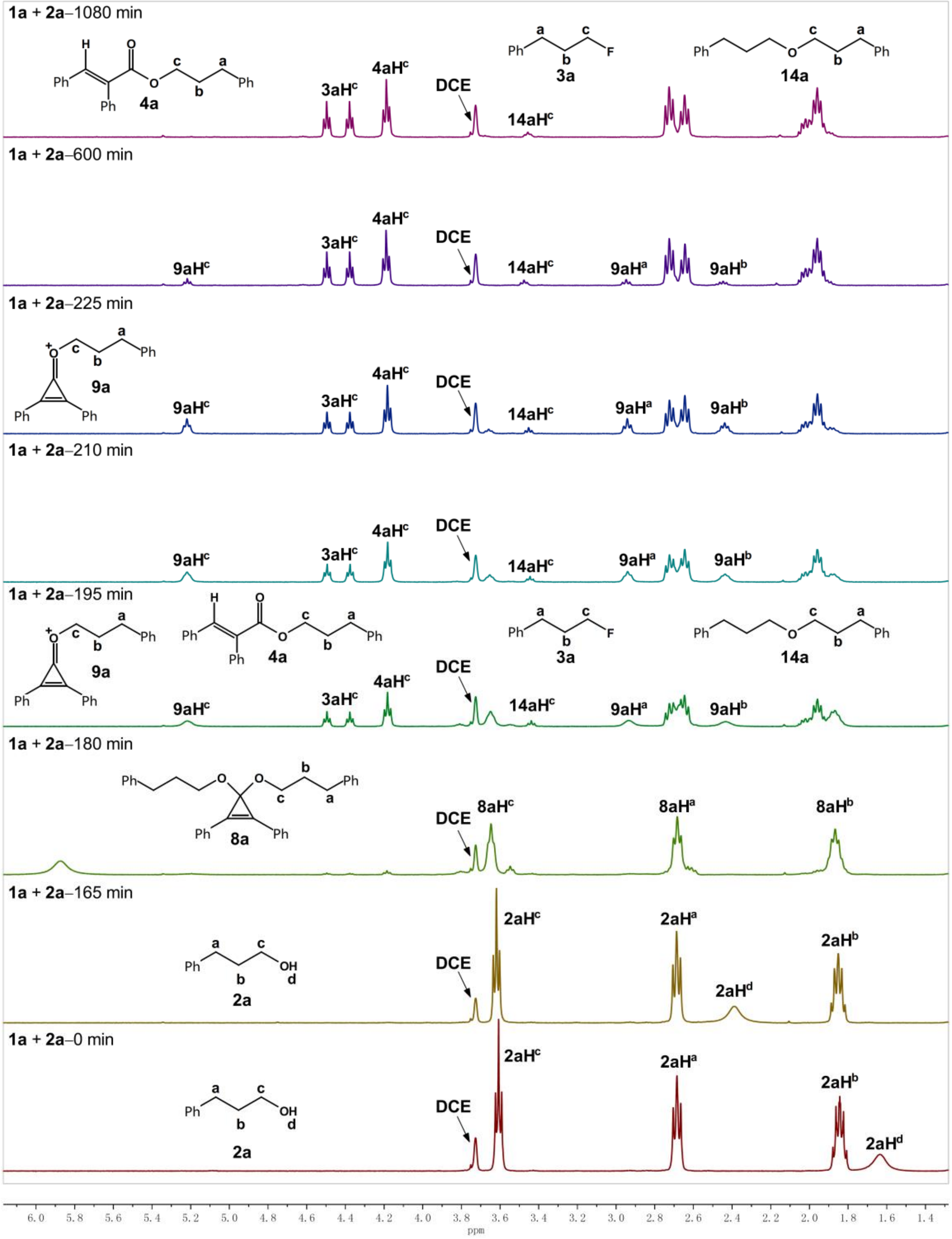

Supplementary Figure 221. Representational ^{1}H NMR Spectroscopy of the Reaction between Alcohol 2a and CpFluor 1a. Only the characteristic peaks are displayed.

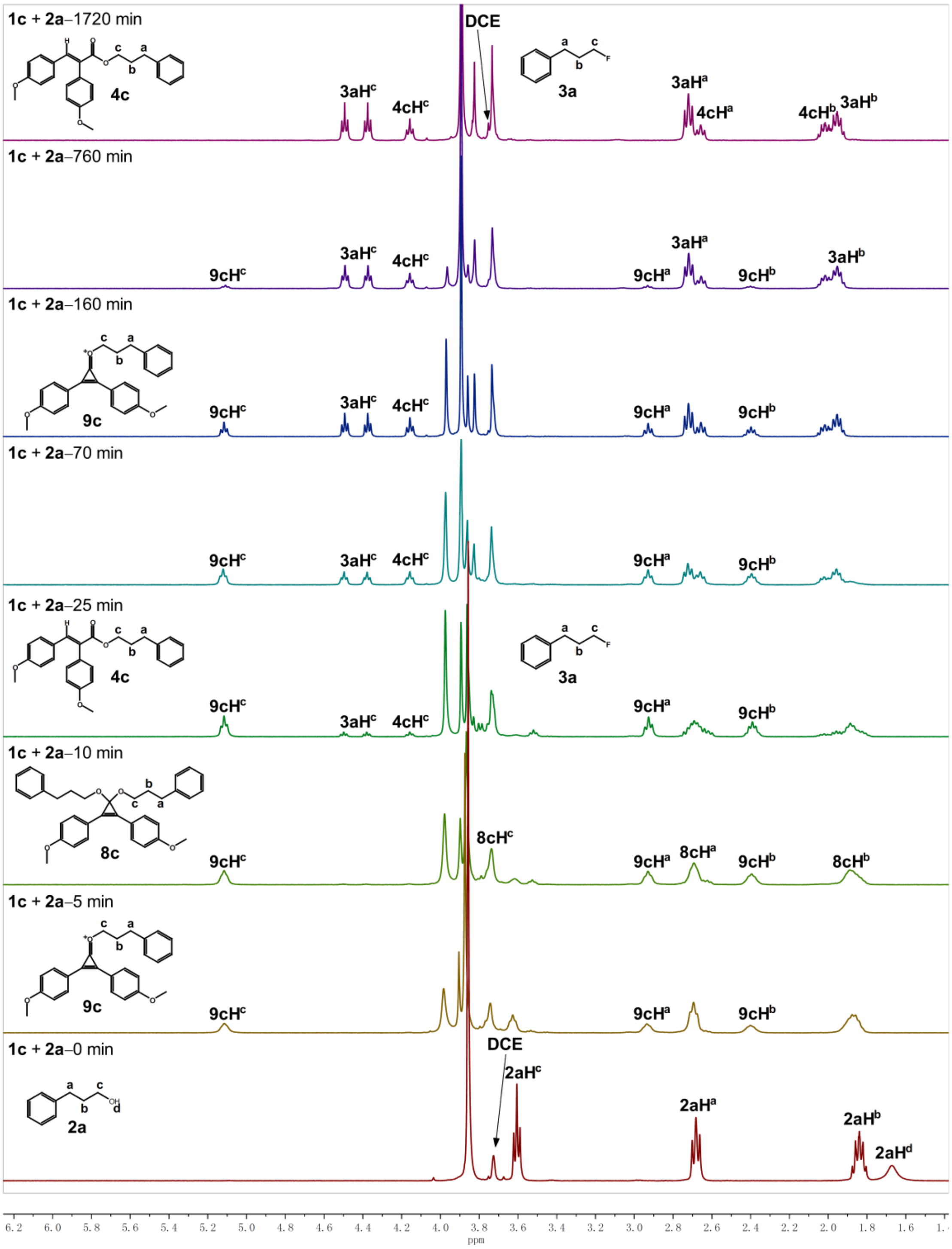

Supplementary Figure 222. Representational ^{1}H NMR Spectroscopy of the Reaction between Alcohol 2a and CpFluor 1c. Only the characteristic peaks are displayed.

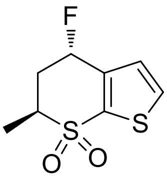

3m

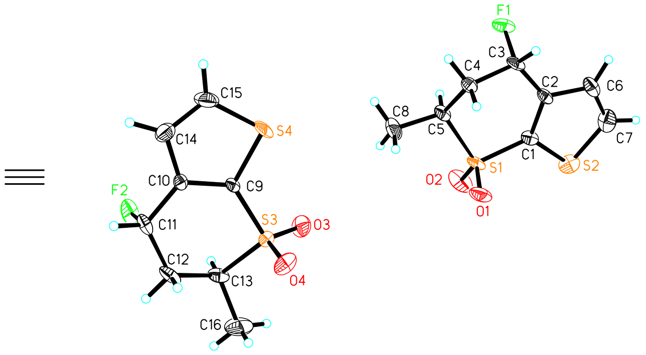

Supplementary Figure 223. ORTEP image of the X-ray crystal structure of 3m

The crystallographic coordinates have been deposited with the Cambridge Crystallographic Data Centre; deposition No.: CCDC 1443649. These data can be obtained free of charge via from the Cambridge Crystallographic Data Centre, 12 Union Road, Cambridge CB2 1EZ, UK; fax: (+44)1223-336-033; via www.ccdc.cam.ac.uk/conts/retrieving.html or deposit@ccdc.cam.ac.uk.

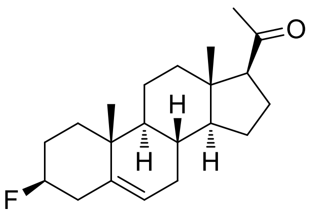

3r

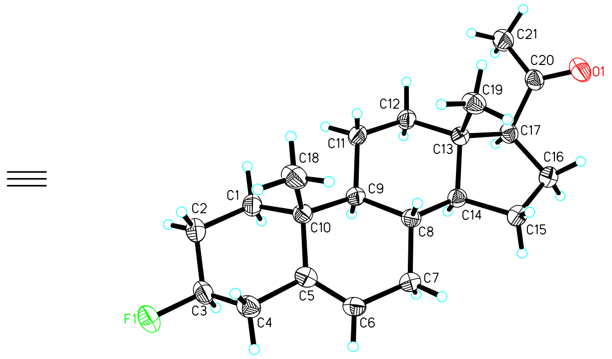

Supplementary Figure 224. ORTEP image of the X-ray crystal structure of 3r

The crystallographic coordinates have been deposited with the Cambridge Crystallographic Data Centre; deposition No.: CCDC 1443627. These data can be obtained free of charge via from the Cambridge Crystallographic Data Centre, 12 Union Road, Cambridge CB2 1EZ, UK; fax: (+44)1223-336-033; via www.ccdc.cam.ac.uk/conts/retrieving.html or deposit@ccdc.cam.ac.uk.

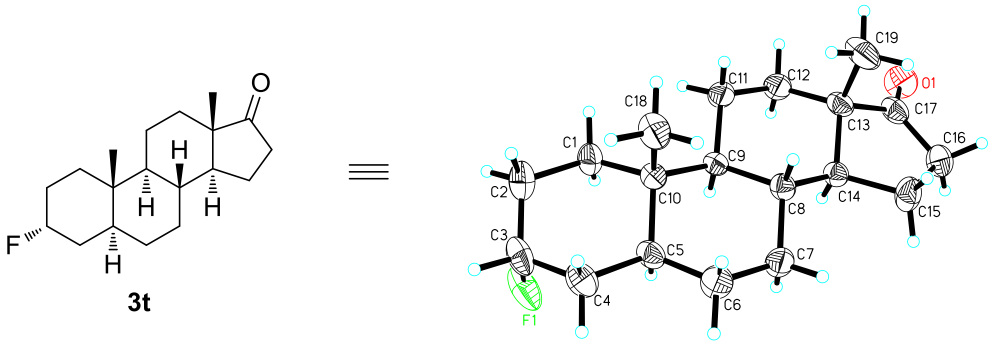

Supplementary Figure 225. ORTEP image of the X-ray crystal structure of 3t

The crystallographic coordinates have been deposited with the Cambridge Crystallographic Data Centre; deposition No.: CCDC 1443644. These data can be obtained free of charge via from the Cambridge Crystallographic Data Centre, 12 Union Road, Cambridge CB2 1EZ, UK; fax: (+44)1223-336-033; via www.ccdc.cam.ac.uk/conts/retrieving.html or deposit@ccdc.cam.ac.uk.

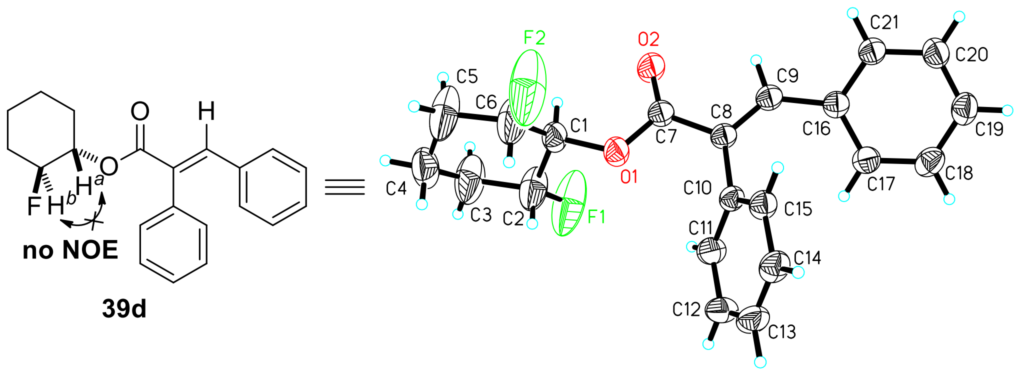

Supplementary Figure 226. ORTEP image of the X-ray crystal structure of 7j

The crystallographic coordinates have been deposited with the Cambridge Crystallographic Data Centre; deposition No.: CCDC 1443654. These data can be obtained free of charge via from the Cambridge Crystallographic Data Centre, 12 Union Road, Cambridge CB2 1EZ, UK; fax: (+44)1223-336-033; via www.ccdc.cam.ac.uk/conts/retrieving.html or deposit@ccdc.cam.ac.uk.

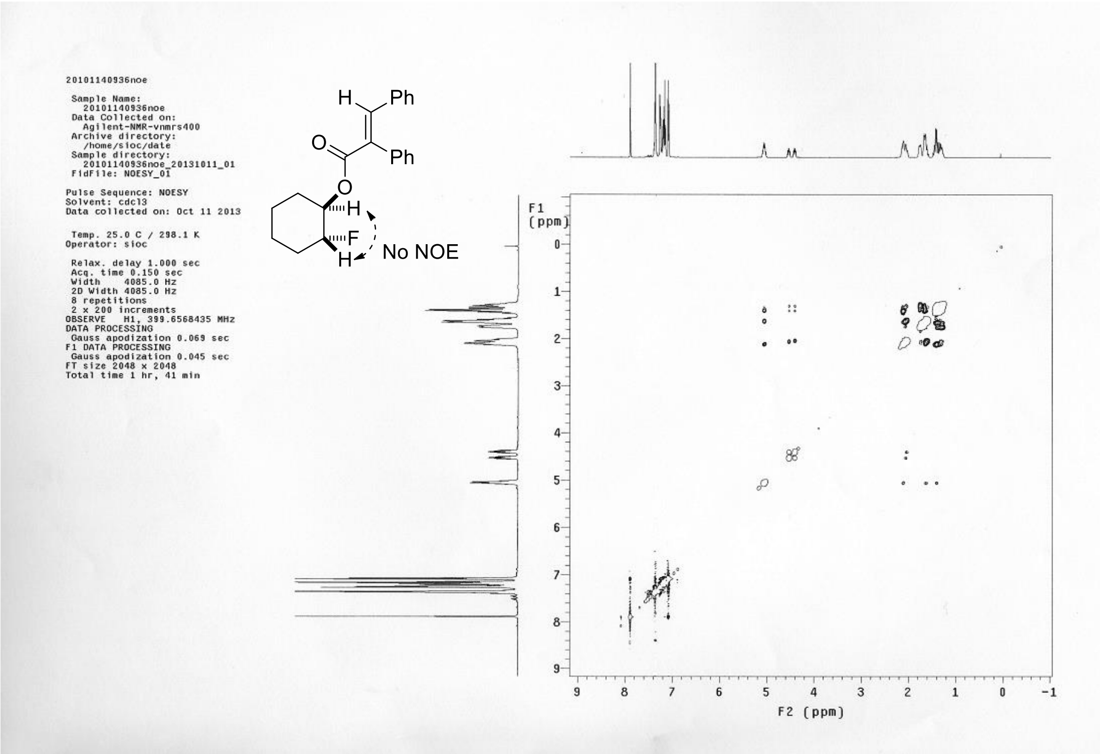

Supplementary Figure 227. NOESY Spectrum of 7j

Supplementary Table 1. Survey of Reaction Conditions for the Deoxyfluorination of Alcohols 2 with CpFluors 1i

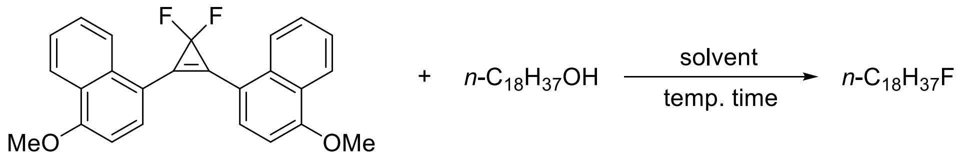

1i **2b** **3b**

Entry	Reactant ratio[a] **1 : 2**	Solvent	Temp (°C)	Time (h)	Yield (%)[b]
1	1.2 : 1.0	Trifluoromethylbenzene	100	12	87
2	1.2 : 1.0	Toluene	100	12	87
3	1.2 : 1.0	DCE	100	12	94
4	1.2 : 1.0	DCE	80	12	91
**5**	**1.1 : 1.0**	**DCE**	**80**	**8**	**93**
6	1.1 : 1.0	DCE	80	6	92
7	1.1 : 1.0	DCE	80	3	81
8	1.2 : 1.0	Chlorobenzene	100	12	94
9	1.1 : 1.0	Chlorobenzene	100	12	94
10	1.1 : 1.0	Chlorobenzene	100	5	92
11	1.1 : 1.0	Chlorobenzene	100	3	96
12[c]	1.1 : 1.0	Chlorobenzene	100	1.5	97
13	1.1 : 1.0	Chlorobenzene	100	0.5	77

[a] Molar ratio. [b] Isolated yield. [c] This condition was not suitable for 2° alcohols due to the enhanced side elimination reaction.

Supplementary Table 2. NMR Study of the Reaction between Monoalcohol 2a and CpFluor 1a

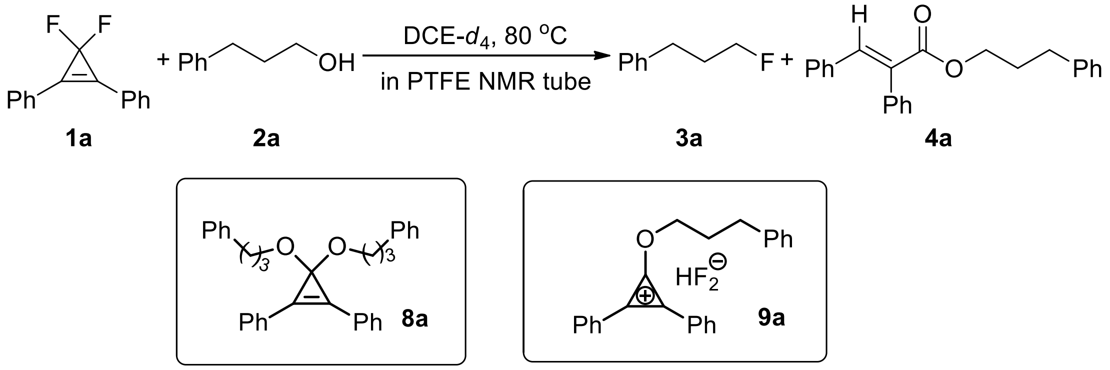

Time (min)	Conversion (%)	Yield (%)				
	1a	[HF]	8a	9a	3a	4a
0	0	0	0	0	0	0
30	0	0	0	0	0	0
60	0	0	0	0	0	0
90	0	0	0	0	0	0
120	0.3	1	0	0	0	0
135	0.8	1.2	0	0	0	0
150	2.2	2.7	0	0	0	0
165	4.6	5.3	0	0	0	0
180	39.9	31.9	62	6.3	1.9	3.8
195	74	50.1	23.6	13.9	22.2	26.4
210	84.6	56.1	11.3	16.1	29.7	35.5
225	89.1	57.7	6.5	15.1	33.6	37.1
240	91.1	58.4	3.2	15.9	35.7	39.7
270	93.1	57.4	1.5	14.7	39.0	41.1
300	93.9	56.9	0	13.5	39.3	40.5
360	95	54.4	0	11.1	40.6	41.1
480	96.4	49.0	0	8	45.4	42.4
600	97.2	43.8	0	5.5	47.3	43.3
720	97.9	39.7	0	3.9	48.3	42.6
840	98.5	35.0	0	2.6	49.5	43.1
960	99.1	31.9	0	1.5	51.2	43.4
1080	99.5	29.2	0	0	52.5	45

Supplementary Table 3. NMR Study of the Reaction between Monoalcohol 2a and CpFluor 1c

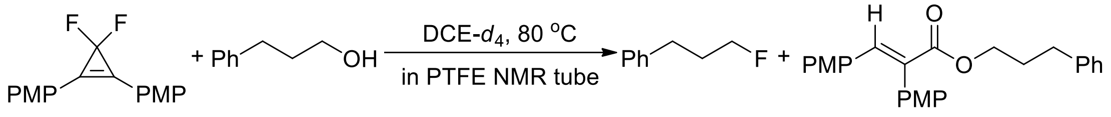

1c **2a** **3a** **4c**

(PMP = *p*-methoxyphenyl)

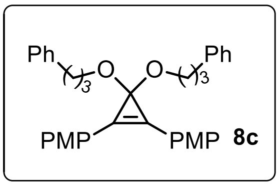

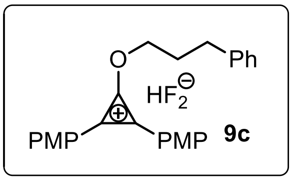

Time (min)	Conversion (%)	Yield (%)				
	**1c**	[HF]	**8c**	**9c**	**3a**	**4c**
0	0	0	0	0	0	0
5	54.2	53.6	34	24	0	0
10	68.6	66.0	39	30	1.2	3
25	78.2	65.2	32	32	10.4	6
40	81.6	62.8	22	31	18.8	12.5
70	85.8	59.6	8	27	32.2	21.5
100	88.5	58.4	0	25	40.1	25
160	90.3	57.0	0	19.5	47.0	26
280	91.3	52.4	0	14	51.0	26.5
400	92	50.2	0	11.2	54.0	27
520	93.7	49.2	0	9.1	55.7	27
760	95.7	42.2	0	6	58.5	27
1000	97.8	36.6	0	4.2	60.2	28
1240	99.1	34.8	0	2.6	61.4	28
1720	100	23.1	0	0	63.0	28.5

Supplementary Table 4. Crystal data and structure refinement for 3m

Empirical formula	$C_8H_9FO_2S_2$
Formula weight	220.27
Temperature	296(2) K
Wavelength	1.54178 Å
Crystal system, space group	Orthorhombic, P 21 21 21
Unit cell dimensions	a = 7.8082(16) Å α = 90°
	b = 9.3493(19) Å β = 90°
	c = 26.310(5) Å γ = 90°
Volume	1920.6(7) Å^3
Z, Calculated density	8, 1.524 Mg/m^3
Absorption coefficient	4.903 mm^{-1}
F(000)	912
Crystal size	0.36 × 0.25 × 0.21 mm^3
Theta range for data collection	3.36 to 67.39^o
Limiting indices	-9<=h<=8, -9<=k<=11, -31<=l<=31
Reflections collected / unique	8699 / 3380 [R(int) = 0.0488]
Completeness to theta = 67.39	99.2 %
Absorption correction	Semi-empirical from equivalents
Max. and min. transmission	0.7529 and 0.2816
Refinement method	Full-matrix least-squares on F^2
Data / restraints / parameters	3380 / 0 / 236
Goodness-of-fit on F^2	1.098
Final R indices [I>2sigma(I)]	R1 = 0.0554, wR2 = 0.1382
R indices (all data)	R1 = 0.0557, wR2 = 0.1388
Absolute structure parameter	0.08(2)
Extinction coefficient	0.0231(14)
Largest diff. peak and hole	0.822 and -0.947 e.Å^{-3}

Supplementary Table 5. Atomic coordinates ($\times 10^4$) and equivalent isotropic displacement parameters (Å^2 $\times 10^3$) for **3m**

	x	y	z	U(eq)
S(1)	6907(1)	7932(1)	8978(1)	35(1)
S(2)	9864(2)	9789(1)	9305(1)	51(1)
S(3)	2420(1)	2203(1)	8191(1)	27(1)
S(4)	-207(1)	3799(1)	8800(1)	47(1)
O(1)	7356(5)	6546(3)	9174(1)	55(1)
O(2)	7370(6)	8260(4)	8463(1)	70(1)
O(3)	3299(4)	3525(3)	8106(1)	54(1)
O(4)	3113(4)	1246(3)	8566(1)	49(1)
F(1)	4211(4)	10744(3)	9816(1)	66(1)
F(2)	-1488(4)	1548(3)	7300(1)	58(1)
C(1)	7799(5)	9211(3)	9377(1)	31(1)
C(2)	6995(5)	9813(3)	9783(1)	32(1)
C(3)	5191(5)	9516(4)	9927(1)	37(1)
C(4)	4371(5)	8263(4)	9657(1)	34(1)
C(5)	4673(5)	8247(4)	9083(1)	34(1)
C(6)	8114(6)	10776(4)	10043(2)	48(1)
C(7)	9662(6)	10851(5)	9829(2)	58(1)
C(8)	3652(7)	7083(6)	8819(2)	63(1)
C(9)	294(4)	2556(3)	8352(1)	23(1)
C(10)	-1097(4)	1845(3)	8171(1)	26(1)
C(11)	-1005(5)	786(4)	7744(1)	37(1)
C(12)	733(6)	169(4)	7661(2)	45(1)
C(13)	2165(5)	1271(4)	7607(1)	37(1)
C(14)	-2595(5)	2295(5)	8419(2)	47(1)
C(15)	-2306(6)	3348(5)	8763(2)	56(1)

C(16)	3895(8)	615(7)	7463(2)	73(2)
H(3B)	5143	9343	10294	45
H(4B)	3147	8282	9721	40
H(4C)	4818	7383	9801	40
H(5A)	4358	9179	8940	41
H(6A)	7798	11295	10329	58
H(7A)	10542	11423	9953	69
H(8A)	2451	7245	8873	94
H(8B)	3965	6169	8957	94
H(8C)	3893	7100	8462	94
H(11A)	-1825	11	7806	44
H(12A)	704	-412	7355	53
H(12B)	1003	-458	7943	53
H(13A)	1839	1961	7344	45
H(14A)	-3672	1911	8355	56
H(15A)	-3162	3775	8957	67
H(16A)	3785	106	7148	110
H(16B)	4254	-34	7725	110
H(16C)	4731	1361	7426	110

Supplementary Table 6. Bond lengths [Å] and angles [^{o}] for **3m**

S(1)-O(2)	1.434(3)	C(5)-H(5A)	0.9800
S(1)-O(1)	1.439(3)	C(6)-C(7)	1.335(7)
S(1)-C(1)	1.738(4)	C(6)-H(6A)	0.9300
S(1)-C(5)	1.790(4)	C(7)-H(7A)	0.9300
S(2)-C(7)	1.707(6)	C(8)-H(8A)	0.9600
S(2)-C(1)	1.711(4)	C(8)-H(8B)	0.9600
S(3)-O(3)	1.432(3)	C(8)-H(8C)	0.9600

S(3)-O(4)	1.436(3)	C(9)-C(10)	1.359(4)
S(3)-C(9)	1.745(3)	C(10)-C(14)	1.405(5)
S(3)-C(13)	1.778(4)	C(10)-C(11)	1.498(4)
S(4)-C(15)	1.695(5)	C(11)-C(12)	1.490(6)
S(4)-C(9)	1.702(3)	C(11)-H(11A)	0.9800
F(1)-C(3)	1.410(4)	C(12)-C(13)	1.528(6)
F(2)-C(11)	1.420(4)	C(12)-H(12A)	0.9700
C(1)-C(2)	1.360(5)	C(12)-H(12B)	0.9700
C(2)-C(6)	1.429(5)	C(13)-C(16)	1.531(6)
C(2)-C(3)	1.485(6)	C(13)-H(13A)	0.9800
C(3)-C(4)	1.512(5)	C(14)-C(15)	1.357(7)
C(3)-H(3B)	0.9800	C(14)-H(14A)	0.9300
C(4)-C(5)	1.530(4)	C(15)-H(15A)	0.9300
C(4)-H(4B)	0.9700	C(16)-H(16A)	0.9600
C(4)-H(4C)	0.9700	C(16)-H(16B)	0.9600
C(5)-C(8)	1.516(6)	C(16)-H(16C)	0.9600

O(2)-S(1)-O(1)	118.1(2)	S(2)-C(1)-S(1)	121.83(19)
O(2)-S(1)-C(1)	108.8(2)	C(1)-C(2)-C(6)	110.7(4)
O(1)-S(1)-C(1)	107.76(17)	C(1)-C(2)-C(3)	124.1(3)
O(2)-S(1)-C(5)	110.9(2)	C(6)-C(2)-C(3)	125.1(3)
O(1)-S(1)-C(5)	109.29(19)	F(1)-C(3)-C(2)	108.1(3)
C(1)-S(1)-C(5)	100.62(16)	F(1)-C(3)-C(4)	107.7(3)
C(7)-S(2)-C(1)	90.4(2)	C(2)-C(3)-C(4)	115.2(3)
O(3)-S(3)-O(4)	117.68(19)	F(1)-C(3)-H(3B)	108.6
O(3)-S(3)-C(9)	109.31(17)	C(2)-C(3)-H(3B)	108.6
O(4)-S(3)-C(9)	108.11(15)	C(4)-C(3)-H(3B)	108.6
O(3)-S(3)-C(13)	109.95(19)	C(3)-C(4)-C(5)	114.0(3)
O(4)-S(3)-C(13)	109.27(18)	C(3)-C(4)-H(4B)	108.8

C(9)-S(3)-C(13)	101.23(16)	C(5)-C(4)-H(4B)	108.8
C(15)-S(4)-C(9)	90.72(18)	C(3)-C(4)-H(4C)	108.8
C(2)-C(1)-S(2)	113.1(3)	C(5)-C(4)-H(4C)	108.8
C(2)-C(1)-S(1)	125.0(3)	H(4B)-C(4)-H(4C)	107.7
C(8)-C(5)-C(4)	112.2(3)	F(2)-C(11)-H(11A)	109.5
C(8)-C(5)-S(1)	108.9(3)	C(12)-C(11)-H(11A)	109.5
C(4)-C(5)-S(1)	107.7(2)	C(10)-C(11)-H(11A)	109.5
C(8)-C(5)-H(5A)	109.3	C(11)-C(12)-C(13)	114.8(3)
C(4)-C(5)-H(5A)	109.3	C(11)-C(12)-H(12A)	108.6
S(1)-C(5)-H(5A)	109.3	C(13)-C(12)-H(12A)	108.6
C(7)-C(6)-C(2)	112.6(4)	C(11)-C(12)-H(12B)	108.6
C(7)-C(6)-H(6A)	123.7	C(13)-C(12)-H(12B)	108.6
C(2)-C(6)-H(6A)	123.7	H(12A)-C(12)-H(12B)	107.6
C(6)-C(7)-S(2)	113.2(3)	C(12)-C(13)-C(16)	113.5(4)
C(6)-C(7)-H(7A)	123.4	C(12)-C(13)-S(3)	109.5(2)
S(2)-C(7)-H(7A)	123.4	C(16)-C(13)-S(3)	108.1(3)
C(5)-C(8)-H(8A)	109.5	C(12)-C(13)-H(13A)	108.6
C(5)-C(8)-H(8B)	109.5	C(16)-C(13)-H(13A)	108.6
H(8A)-C(8)-H(8B)	109.5	S(3)-C(13)-H(13A)	108.6
C(5)-C(8)-H(8C)	109.5	C(15)-C(14)-C(10)	112.9(4)
H(8A)-C(8)-H(8C)	109.5	C(15)-C(14)-H(14A)	123.6
H(8B)-C(8)-H(8C)	109.5	C(10)-C(14)-H(14A)	123.6
C(10)-C(9)-S(4)	113.2(2)	C(14)-C(15)-S(4)	112.3(3)
C(10)-C(9)-S(3)	125.7(2)	C(14)-C(15)-H(15A)	123.8
S(4)-C(9)-S(3)	121.01(18)	S(4)-C(15)-H(15A)	123.8
C(9)-C(10)-C(14)	110.9(3)	C(13)-C(16)-H(16A)	109.5
C(9)-C(10)-C(11)	123.2(3)	C(13)-C(16)-H(16B)	109.5
C(14)-C(10)-C(11)	125.9(3)	H(16A)-C(16)-H(16B)	109.5
F(2)-C(11)-C(12)	108.3(3)	C(13)-C(16)-H(16C)	109.5

F(2)-C(11)-C(10)	105.8(3)	H(16A)-C(16)-H(16C)	109.5
C(12)-C(11)-C(10)	114.2(3)	H(16B)-C(16)-H(16C)	109.5

Supplementary Table 7. Torsion angles [^{o}] for **3m**

C(7)-S(2)-C(1)-C(2)	0.3(3)
C(7)-S(2)-C(1)-S(1)	-177.3(2)
O(2)-S(1)-C(1)-C(2)	138.9(3)
O(1)-S(1)-C(1)-C(2)	-92.0(3)
C(5)-S(1)-C(1)-C(2)	22.4(3)
O(2)-S(1)-C(1)-S(2)	-43.8(3)
O(1)-S(1)-C(1)-S(2)	85.3(2)
C(5)-S(1)-C(1)-S(2)	-160.3(2)
S(2)-C(1)-C(2)-C(6)	-0.1(4)
S(1)-C(1)-C(2)-C(6)	177.4(3)
S(2)-C(1)-C(2)-C(3)	179.0(3)
S(1)-C(1)-C(2)-C(3)	-3.5(5)
C(1)-C(2)-C(3)-F(1)	-108.5(4)
C(6)-C(2)-C(3)-F(1)	70.4(4)
C(1)-C(2)-C(3)-C(4)	11.9(5)
C(6)-C(2)-C(3)-C(4)	-169.1(3)
F(1)-C(3)-C(4)-C(5)	74.2(4)
C(2)-C(3)-C(4)-C(5)	-46.4(4)
C(3)-C(4)-C(5)-C(8)	-172.9(3)
C(3)-C(4)-C(5)-S(1)	67.3(3)
O(2)-S(1)-C(5)-C(8)	72.9(3)
O(1)-S(1)-C(5)-C(8)	-58.9(3)
C(1)-S(1)-C(5)-C(8)	-172.2(3)
O(2)-S(1)-C(5)-C(4)	-165.2(3)

O(1)-S(1)-C(5)-C(4)	62.9(3)
C(1)-S(1)-C(5)-C(4)	-50.3(3)
C(1)-C(2)-C(6)-C(7)	-0.2(5)
C(3)-C(2)-C(6)-C(7)	-179.3(3)
C(2)-C(6)-C(7)-S(2)	0.5(5)
C(1)-S(2)-C(7)-C(6)	-0.4(4)
C(15)-S(4)-C(9)-C(10)	1.4(3)
C(15)-S(4)-C(9)-S(3)	-174.4(2)
O(3)-S(3)-C(9)-C(10)	136.7(3)
O(4)-S(3)-C(9)-C(10)	-94.1(3)
C(13)-S(3)-C(9)-C(10)	20.7(3)
O(3)-S(3)-C(9)-S(4)	-48.0(2)
O(4)-S(3)-C(9)-S(4)	81.2(2)
C(13)-S(3)-C(9)-S(4)	-163.99(19)
S(4)-C(9)-C(10)-C(14)	-2.2(4)
S(3)-C(9)-C(10)-C(14)	173.5(3)
S(4)-C(9)-C(10)-C(11)	174.9(2)
S(3)-C(9)-C(10)-C(11)	-9.4(4)
C(9)-C(10)-C(11)-F(2)	-97.6(4)
C(14)-C(10)-C(11)-F(2)	79.1(4)
C(9)-C(10)-C(11)-C(12)	21.4(4)
C(14)-C(10)-C(11)-C(12)	-161.9(4)
F(2)-C(11)-C(12)-C(13)	65.9(4)
C(10)-C(11)-C(12)-C(13)	-51.6(4)
C(11)-C(12)-C(13)-C(16)	-173.6(3)
C(11)-C(12)-C(13)-S(3)	65.5(4)
O(3)-S(3)-C(13)-C(12)	-160.3(3)
O(4)-S(3)-C(13)-C(12)	69.1(3)
C(9)-S(3)-C(13)-C(12)	-44.8(3)

O(3)-S(3)-C(13)-C(16)	75.6(4)
O(4)-S(3)-C(13)-C(16)	-54.9(4)
C(9)-S(3)-C(13)-C(16)	-168.8(3)
C(9)-C(10)-C(14)-C(15)	2.0(5)
C(11)-C(10)-C(14)-C(15)	-175.0(4)
C(10)-C(14)-C(15)-S(4)	-0.9(5)
C(9)-S(4)-C(15)-C(14)	-0.3(4)

Supplementary Table 8. Crystal data and structure refinement for **3r**

Empirical formula	$C_{21}H_{31}FO$
Formula weight	318.46
Temperature	296(2) K
Wavelength	1.54178 Å
Crystal system, space group	Monoclinic, P 21
Unit cell dimensions	a = 12.145(2) Å α = 90°
	b = 12.015(2) Å β = 100.79(3)^o
	c = 12.325(3) Å γ = 90°
Volume	1766.8(6) Å^3
Z, Calculated density	4, 1.197 Mg/m^3
Absorption coefficient	0.614 mm^{-1}
F(000)	696
Crystal size	0.31 × 0.25 × 0.23 mm^3
Theta range for data collection	4.69 to 66.89^o
Limiting indices	-14<=h<=12, -13<=k<=14, -14<=l<=14
Reflections collected / unique	8645 / 4919 [R(int) = 0.0178]
Completeness to theta = 66.89	96.5 %
Absorption correction	Semi-empirical from equivalents
Max. and min. transmission	0.7529 and 0.5561
Refinement method	Full-matrix least-squares on F^2
Data / restraints / parameters	4919 / 1 / 416
Goodness-of-fit on F^2	1.031
Final R indices [I>2sigma(I)]	R1 = 0.0376, wR2 = 0.1032
R indices (all data)	R1 = 0.0380, wR2 = 0.1037
Absolute structure parameter	-0.06(15)
Extinction coefficient	0.0023(3)
Largest diff. peak and hole	0.177 and -0.163 e.Å^{-3}

Supplementary Table 9. Atomic coordinates ($\times 10^4$) and equivalent isotropic displacement parameters ($Å^2 \times 10^3$) for **3r**

	x	y	z	U(eq)
O(1)	1961(1)	-530(2)	16375(1)	74(1)
O(1A)	4702(1)	557(2)	-4526(1)	68(1)
F(1)	-59(1)	1595(1)	6561(1)	68(1)
F(1A)	7442(1)	19(2)	5494(1)	82(1)
C(1)	-445(2)	340(2)	9218(2)	47(1)
C(1A)	6367(2)	-884(2)	2611(2)	57(1)
C(2)	-471(2)	406(2)	7970(2)	52(1)
C(2A)	6479(2)	-860(2)	3869(2)	63(1)
C(3)	-32(2)	1519(2)	7709(2)	49(1)
C(3A)	7308(2)	8(3)	4329(2)	65(1)
C(4)	1152(2)	1706(2)	8313(2)	49(1)
C(4A)	6935(2)	1152(2)	3889(2)	59(1)
C(5)	1228(1)	1584(2)	9553(1)	41(1)
C(5A)	6757(2)	1157(2)	2636(2)	45(1)
C(6)	1710(2)	2364(2)	10237(2)	49(1)
C(6A)	7225(2)	1933(2)	2104(2)	53(1)
C(7)	1838(2)	2320(2)	11472(2)	51(1)
C(7A)	7092(2)	2024(2)	878(2)	50(1)
C(8)	1613(1)	1171(2)	11900(1)	37(1)
C(8A)	6164(1)	1285(2)	247(1)	38(1)
C(9)	581(1)	655(2)	11160(1)	37(1)
C(9A)	6167(1)	149(2)	824(1)	37(1)
C(10)	726(1)	527(2)	9937(1)	38(1)
C(10A)	6011(1)	240(2)	2045(1)	39(1)
C(11)	212(2)	-428(2)	11642(2)	52(1)

C(11A)	5339(2)	-664(2)	138(2)	48(1)
C(12)	91(2)	-342(2)	12857(2)	48(1)
C(12A)	5481(2)	-779(2)	-1066(2)	45(1)
C(13)	1171(1)	100(2)	13572(1)	38(1)
C(13A)	5439(1)	352(2)	-1637(1)	38(1)
C(14)	1419(1)	1230(2)	13086(1)	38(1)
C(14A)	6334(1)	1094(2)	-934(1)	39(1)
C(15)	2310(2)	1756(2)	13973(2)	49(1)
C(15A)	6452(2)	2089(2)	-1669(2)	52(1)
C(16)	1979(2)	1387(2)	15064(2)	48(1)
C(16A)	6233(2)	1614(2)	-2847(2)	58(1)
C(17)	1082(1)	470(2)	14770(1)	41(1)
C(17A)	5878(2)	393(2)	-2750(2)	44(1)
C(18)	1494(2)	-462(2)	9787(2)	56(1)
C(18A)	4788(2)	497(2)	2132(2)	53(1)
C(19)	2139(2)	-732(2)	13629(2)	51(1)
C(19A)	4251(2)	849(2)	-1805(2)	51(1)
C(20)	1176(2)	-457(2)	15625(1)	46(1)
C(20A)	5089(2)	-41(2)	-3764(2)	50(1)
C(21)	243(2)	-1282(2)	15521(2)	62(1)
C(21A)	4822(2)	-1256(2)	-3815(2)	67(1)
H(1A)	-716	-387	9389	56
H(1B)	-956	893	9415	56
H(1AA)	5820	-1445	2312	68
H(1AB)	7081	-1104	2432	68
H(2A)	-12	-180	7747	62
H(2B)	-1233	310	7570	62
H(2AA)	5758	-695	4063	75
H(2AB)	6726	-1581	4176	75

H(3A)	-515	2100	7925	58
H(3AA)	8030	-168	4124	77
H(4A)	1395	2446	8149	59
H(4B)	1646	1170	8061	59
H(4AA)	7500	1698	4185	70
H(4AB)	6242	1353	4124	70
H(6A)	1992	2986	9931	58
H(6AA)	7671	2462	2528	64
H(7A)	1323	2848	11703	62
H(7B)	2593	2545	11802	62
H(7AA)	7795	1826	664	60
H(7AB)	6930	2792	664	60
H(8A)	2265	695	11879	44
H(8AA)	5438	1647	230	46
H(9A)	-33	1185	11152	44
H(9AA)	6915	-166	848	45
H(11A)	755	-1004	11577	62
H(11B)	-501	-656	11206	62
H(11C)	5431	-1391	484	57
H(11D)	4581	-415	149	57
H(12A)	-526	154	12917	57
H(12B)	-81	-1069	13122	57
H(12C)	6194	-1134	-1089	54
H(12D)	4891	-1250	-1460	54
H(14A)	742	1679	13061	46
H(14B)	7040	681	-867	47
H(15A)	2300	2560	13908	58
H(15B)	3051	1486	13922	58
H(15C)	7198	2406	-1484	62

H(15D)	5907	2661	-1591	62
H(16A)	1679	2010	15414	58
H(16B)	2627	1099	15567	58
H(16C)	6907	1656	-3162	70
H(16D)	5643	2029	-3316	70
H(17A)	348	822	14730	49
H(17B)	6560	-61	-2655	53
H(18A)	1566	-512	9026	84
H(18B)	1174	-1138	10003	84
H(18C)	2220	-350	10238	84
H(18D)	4726	544	2896	79
H(18E)	4309	-87	1782	79
H(18F)	4565	1192	1773	79
H(19A)	2194	-963	12895	76
H(19B)	2000	-1370	14053	76
H(19C)	2827	-385	13974	76
H(19D)	3981	819	-1122	77
H(19E)	3761	428	-2357	77
H(19F)	4271	1609	-2041	77
H(21A)	410	-1822	16103	93
H(21B)	160	-1650	14819	93
H(21C)	-442	-905	15574	93
H(21D)	4319	-1418	-4494	101
H(21E)	4476	-1454	-3203	101
H(21F)	5501	-1675	-3781	101

Supplementary Table 10. Bond lengths [Å] and angles [^{o}] for **3r**

O(1)-C(20)	1.201(2)	C(11)-H(11A)	0.9700

O(1A)-C(20A)	1.206(3)	C(11)-H(11B)	0.9700
F(1)-C(3)	1.412(2)	C(11A)-C(12A)	1.531(2)
F(1A)-C(3A)	1.415(2)	C(11A)-H(11C)	0.9700
C(1)-C(2)	1.535(2)	C(11A)-H(11D)	0.9700
C(1)-C(10)	1.544(2)	C(12)-C(13)	1.532(2)
C(1)-H(1A)	0.9700	C(12)-H(12A)	0.9700
C(1)-H(1B)	0.9700	C(12)-H(12B)	0.9700
C(1A)-C(2A)	1.532(3)	C(12A)-C(13A)	1.527(3)
C(1A)-C(10A)	1.544(3)	C(12A)-H(12C)	0.9700
C(1A)-H(1AA)	0.9700	C(12A)-H(12D)	0.9700
C(1A)-H(1AB)	0.9700	C(13)-C(19)	1.535(2)
C(2)-C(3)	1.497(3)	C(13)-C(14)	1.537(3)
C(2)-H(2A)	0.9700	C(13)-C(17)	1.564(2)
C(2)-H(2B)	0.9700	C(13A)-C(19A)	1.538(2)
C(2A)-C(3A)	1.487(4)	C(13A)-C(14A)	1.541(2)
C(2A)-H(2AA)	0.9700	C(13A)-C(17A)	1.563(2)
C(2A)-H(2AB)	0.9700	C(14)-C(15)	1.523(3)
C(3)-C(4)	1.507(3)	C(14)-H(14A)	0.9800
C(3)-H(3A)	0.9800	C(14A)-C(15A)	1.523(3)
C(3A)-C(4A)	1.515(4)	C(14A)-H(14B)	0.9800
C(3A)-H(3AA)	0.9800	C(15)-C(16)	1.538(3)
C(4)-C(5)	1.521(2)	C(15)-H(15A)	0.9700
C(4)-H(4A)	0.9700	C(15)-H(15B)	0.9700
C(4)-H(4B)	0.9700	C(15A)-C(16A)	1.537(3)
C(4A)-C(5A)	1.518(3)	C(15A)-H(15C)	0.9700
C(4A)-H(4AA)	0.9700	C(15A)-H(15D)	0.9700
C(4A)-H(4AB)	0.9700	C(16)-C(17)	1.545(3)
C(5)-C(6)	1.322(3)	C(16)-H(16A)	0.9700
C(5)-C(10)	1.523(3)	C(16)-H(16B)	0.9700

C(5A)-C(6A)	1.327(3)	C(16A)-C(17A)	1.541(3)
C(5A)-C(10A)	1.523(3)	C(16A)-H(16C)	0.9700
C(6)-C(7)	1.502(3)	C(16A)-H(16D)	0.9700
C(6)-H(6A)	0.9300	C(17)-C(20)	1.523(3)
C(6A)-C(7A)	1.493(3)	C(17)-H(17A)	0.9800
C(6A)-H(6AA)	0.9300	C(17A)-C(20A)	1.517(3)
C(7)-C(8)	1.521(3)	C(17A)-H(17B)	0.9800
C(7)-H(7A)	0.9700	C(18)-H(18A)	0.9600
C(7)-H(7B)	0.9700	C(18)-H(18B)	0.9600
C(7A)-C(8A)	1.527(3)	C(18)-H(18C)	0.9600
C(7A)-H(7AA)	0.9700	C(18A)-H(18D)	0.9600
C(7A)-H(7AB)	0.9700	C(18A)-H(18E)	0.9600
C(8)-C(14)	1.526(2)	C(18A)-H(18F)	0.9600
C(8)-C(9)	1.536(2)	C(19)-H(19A)	0.9600
C(8)-H(8A)	0.9800	C(19)-H(19B)	0.9600
C(8A)-C(14A)	1.526(2)	C(19)-H(19C)	0.9600
C(8A)-C(9A)	1.539(2)	C(19A)-H(19D)	0.9600
C(8A)-H(8AA)	0.9800	C(19A)-H(19E)	0.9600
C(9)-C(11)	1.532(3)	C(19A)-H(19F)	0.9600
C(9)-C(10)	1.557(2)	C(20)-C(21)	1.493(3)
C(9)-H(9A)	0.9800	C(20A)-C(21A)	1.494(3)
C(9A)-C(11A)	1.537(2)	C(21)-H(21A)	0.9600
C(9A)-C(10A)	1.554(2)	C(21)-H(21B)	0.9600
C(9A)-H(9AA)	0.9800	C(21)-H(21C)	0.9600
C(10)-C(18)	1.543(3)	C(21A)-H(21D)	0.9600
C(10A)-C(18A)	1.541(2)	C(21A)-H(21E)	0.9600
C(11)-C(12)	1.535(2)	C(21A)-H(21F)	0.9600
C(2)-C(1)-C(10)	114.32(15)	C(12A)-C(11A)-H(11D)	108.7

C(2)-C(1)-H(1A)	108.7	C(9A)-C(11A)-H(11D)	108.7
C(10)-C(1)-H(1A)	108.7	H(11C)-C(11A)-H(11D)	107.6
C(2)-C(1)-H(1B)	108.7	C(13)-C(12)-C(11)	110.88(15)
C(10)-C(1)-H(1B)	108.7	C(13)-C(12)-H(12A)	109.5
H(1A)-C(1)-H(1B)	107.6	C(11)-C(12)-H(12A)	109.5
C(2A)-C(1A)-C(10A)	113.91(17)	C(13)-C(12)-H(12B)	109.5
C(2A)-C(1A)-H(1AA)	108.8	C(11)-C(12)-H(12B)	109.5
C(10A)-C(1A)-H(1AA)	108.8	H(12A)-C(12)-H(12B)	108.1
C(2A)-C(1A)-H(1AB)	108.8	C(13A)-C(12A)-C(11A)	111.51(16)
C(10A)-C(1A)-H(1AB)	108.8	C(13A)-C(12A)-H(12C)	109.3
H(1AA)-C(1A)-H(1AB)	107.7	C(11A)-C(12A)-H(12C)	109.3
C(3)-C(2)-C(1)	108.66(16)	C(13A)-C(12A)-H(12D)	109.3
C(3)-C(2)-H(2A)	110.0	C(11A)-C(12A)-H(12D)	109.3
C(1)-C(2)-H(2A)	110.0	H(12C)-C(12A)-H(12D)	108.0
C(3)-C(2)-H(2B)	110.0	C(12)-C(13)-C(19)	111.54(17)
C(1)-C(2)-H(2B)	110.0	C(12)-C(13)-C(14)	106.93(14)
H(2A)-C(2)-H(2B)	108.3	C(19)-C(13)-C(14)	112.87(14)
C(3A)-C(2A)-C(1A)	108.9(2)	C(12)-C(13)-C(17)	116.00(14)
C(3A)-C(2A)-H(2AA)	109.9	C(19)-C(13)-C(17)	109.24(14)
C(1A)-C(2A)-H(2AB)	109.9	C(14)-C(13)-C(17)	99.78(14)
C(3A)-C(2A)-H(2AB)	109.9	C(12A)-C(13A)-C(19A)	110.91(15)
C(1A)-C(2A)-H(2AB)	109.9	C(12A)-C(13A)-C(14A)	107.20(14)
H(2AA)-C(2A)-H(2AB)	108.3	C(19A)-C(13A)-C(14A)	113.36(15)
F(1)-C(3)-C(2)	109.28(17)	C(12A)-C(13A)-C(17A)	116.44(15)
F(1)-C(3)-C(4)	109.04(16)	C(19A)-C(13A)-C(17A)	109.59(15)
C(2)-C(3)-C(4)	111.84(17)	C(14A)-C(13A)-C(17A)	98.89(14)
F(1)-C(3)-H(3A)	108.9	C(15)-C(14)-C(8)	119.40(14)
C(2)-C(3)-H(3A)	108.9	C(15)-C(14)-C(13)	104.58(14)
C(4)-C(3)-H(3A)	108.9	C(8)-C(14)-C(13)	114.14(14)

F(1A)-C(3A)-C(2A)	109.5(2)	C(15)-C(14)-H(14A)	105.9
F(1A)-C(3A)-C(4A)	109.0(2)	C(8)-C(14)-H(14A)	105.9
C(2A)-C(3A)-C(4A)	111.40(18)	C(13)-C(14)-H(14A)	105.9
F(1A)-C(3A)-H(3AA)	109.0	C(15A)-C(14A)-C(8A)	119.55(15)
C(2A)-C(3A)-H(3AA)	109.0	C(15A)-C(14A)-C(13A)	104.74(15)
C(4A)-C(3A)-H(3AA)	109.0	C(8A)-C(14A)-C(13A)	114.64(13)
C(3)-C(4)-C(5)	110.81(15)	C(15A)-C(14A)-H(14B)	105.6
C(3)-C(4)-H(4A)	109.5	C(8A)-C(14A)-H(14B)	105.6
C(5)-C(4)-H(4A)	109.5	C(13A)-C(14A)-H(14B)	105.6
C(3)-C(4)-H(4B)	109.5	C(14)-C(15)-C(16)	103.95(15)
C(5)-C(4)-H(4B)	109.5	C(14)-C(15)-H(15A)	111.0
H(4A)-C(4)-H(4B)	108.1	C(16)-C(15)-H(15A)	111.0
C(3A)-C(4A)-C(5A)	110.09(18)	C(14)-C(15)-H(15B)	111.0
C(3A)-C(4A)-H(4AA)	109.6	C(16)-C(15)-H(15B)	111.0
C(5A)-C(4A)-H(4AA)	109.6	H(15A)-C(15)-H(15B)	109.0
C(3A)-C(4A)-H(4AB)	109.6	C(14A)-C(15A)-C(16A)	104.57(17)
C(5A)-C(4A)-H(4AB)	109.6	C(14A)-C(15A)-H(15C)	110.8
H(4AA)-C(4A)-H(4AB)	108.2	C(16A)-C(15A)-H(15C)	110.8
C(6)-C(5)-C(4)	120.62(17)	C(14A)-C(15A)-H(15D)	110.8
C(6)-C(5)-C(10)	123.26(16)	C(16A)-C(15A)-H(15D)	110.8
C(4)-C(5)-C(10)	116.12(16)	H(15C)-C(15A)-H(15D)	108.9
C(6A)-C(5A)-C(4A)	120.86(18)	C(15)-C(16)-C(17)	106.78(15)
C(6A)-C(5A)-C(10A)	122.89(17)	C(15)-C(16)-H(16A)	110.4
C(4A)-C(5A)-C(10A)	116.25(17)	C(17)-C(16)-H(16A)	110.4
C(5)-C(6)-C(7)	124.79(17)	C(15)-C(16)-H(16B)	110.4
C(5)-C(6)-H(6A)	117.6	C(17)-C(16)-H(16B)	110.4
C(7)-C(6)-H(6A)	117.6	H(16A)-C(16)-H(16B)	108.6
C(5A)-C(6A)-C(7A)	125.21(17)	C(15A)-C(16A)-C(17A)	106.35(16)
C(5A)-C(6A)-H(6AA)	117.4	C(15A)-C(16A)-H(16C)	110.5

C(7A)-C(6A)-H(6AA)	117.4	C(17A)-C(16A)-H(16C)	110.5
C(6)-C(7)-C(8)	113.08(16)	C(15A)-C(16A)-H(16D)	110.5
C(6)-C(7)-H(7A)	109.0	C(17A)-C(16A)-H(16D)	110.5
C(8)-C(7)-H(7A)	109.0	H(16C)-C(16A)-H(16D)	108.6
C(6)-C(7)-H(7B)	109.0	C(20)-C(17)-C(16)	113.64(15)
C(8)-C(7)-H(7B)	109.0	C(20)-C(17)-C(13)	115.86(16)
H(7A)-C(7)-H(7B)	107.8	C(16)-C(17)-C(13)	104.70(14)
C(6A)-C(7A)-C(8A)	113.71(15)	C(20)-C(17)-H(17A)	107.4
C(6A)-C(7A)-H(7AA)	108.8	C(16)-C(17)-H(17A)	107.4
C(8A)-C(7A)-H(7AA)	108.8	C(13)-C(17)-H(17A)	107.4
C(6A)-C(7A)-H(7AB)	108.8	C(20A)-C(17A)-C(16A)	113.95(17)
C(8A)-C(7A)-H(7AB)	108.8	C(20A)-C(17A)-C(13A)	116.43(15)
H(7AA)-C(7A)-H(7AB)	107.7	C(16A)-C(17A)-C(13A)	104.15(15)
C(7)-C(8)-C(14)	111.06(15)	C(20A)-C(17A)-H(17B)	107.3
C(7)-C(8)-C(9)	109.88(14)	C(16A)-C(17A)-H(17B)	107.3
C(14)-C(8)-C(9)	109.44(13)	C(13A)-C(17A)-H(17B)	107.3
C(7)-C(8)-H(8A)	108.8	C(10)-C(18)-H(18A)	109.5
C(14)-C(8)-H(8A)	108.8	C(10)-C(18)-H(18B)	109.5
C(9)-C(8)-H(8A)	108.8	H(18A)-C(18)-H(18B)	109.5
C(14A)-C(8A)-C(7A)	110.78(14)	C(10)-C(18)-H(18C)	109.5
C(14A)-C(8A)-C(9A)	108.56(14)	H(18A)-C(18)-H(18C)	109.5
C(7A)-C(8A)-C(9A)	110.06(15)	H(18B)-C(18)-H(18C)	109.5
C(14A)-C(8A)-H(8AA)	109.1	C(10A)-C(18A)-H(18D)	109.5
C(7A)-C(8A)-H(8AA)	109.1	C(10A)-C(18A)-H(18E)	109.5
C(9A)-C(8A)-H(8AA)	109.1	H(18D)-C(18A)-H(18E)	109.5
C(11)-C(9)-C(8)	112.19(14)	C(10A)-C(18A)-H(18F)	109.5
C(11)-C(9)-C(10)	112.73(14)	H(18D)-C(18A)-H(18F)	109.5
C(8)-C(9)-C(10)	112.76(13)	H(18E)-C(18A)-H(18F)	109.5
C(11)-C(9)-H(9A)	106.2	C(13)-C(19)-H(19A)	109.5

C(8)-C(9)-H(9A)	106.2	C(13)-C(19)-H(19B)	109.5
C(10)-C(9)-H(9A)	106.2	H(19A)-C(19)-H(19B)	109.5
C(11A)-C(9A)-C(8A)	111.40(14)	C(13)-C(19)-H(19C)	109.5
C(11A)-C(9A)-C(10A)	113.06(14)	H(19A)-C(19)-H(19C)	109.5
C(8A)-C(9A)-C(10A)	113.28(14)	H(19B)-C(19)-H(19C)	109.5
C(11A)-C(9A)-H(9AA)	106.1	C(13A)-C(19A)-H(19D)	109.5
C(8A)-C(9A)-H(9AA)	106.1	C(13A)-C(19A)-H(19E)	109.5
C(10A)-C(9A)-H(9AA)	106.1	H(19D)-C(19A)-H(19E)	109.5
C(5)-C(10)-C(18)	108.56(14)	C(13A)-C(19A)-H(19F)	109.5
C(5)-C(10)-C(1)	108.81(14)	H(19D)-C(19A)-H(19F)	109.5
C(18)-C(10)-C(1)	109.33(16)	H(19E)-C(19A)-H(19F)	109.5
C(5)-C(10)-C(9)	110.09(14)	O(1)-C(20)-C(21)	119.85(19)
C(18)-C(10)-C(9)	111.87(14)	O(1)-C(20)-C(17)	122.17(19)
C(1)-C(10)-C(9)	108.12(13)	C(21)-C(20)-C(17)	117.97(17)
C(5A)-C(10A)-C(18A)	108.47(15)	O(1A)-C(20A)-C(21A)	120.0(2)
C(5A)-C(10A)-C(1A)	109.00(15)	O(1A)-C(20A)-C(17A)	122.1(2)
C(18A)-C(10A)-C(1A)	109.61(17)	C(21A)-C(20A)-C(17A)	117.96(19)
C(5A)-C(10A)-C(9A)	110.14(14)	C(20)-C(21)-H(21A)	109.5
C(18A)-C(10A)-C(9A)	112.03(14)	C(20)-C(21)-H(21B)	109.5
C(1A)-C(10A)-C(9A)	107.54(14)	H(21A)-C(21)-H(21B)	109.5
C(9)-C(11)-C(12)	114.17(16)	C(20)-C(21)-H(21C)	109.5
C(9)-C(11)-H(11A)	108.7	H(21A)-C(21)-H(21C)	109.5
C(12)-C(11)-H(11A)	108.7	H(21B)-C(21)-H(21C)	109.5
C(9)-C(11)-H(11B)	108.7	C(20A)-C(21A)-H(21D)	109.5
C(12)-C(11)-H(11B)	108.7	C(20A)-C(21A)-H(21E)	109.5
H(11A)-C(11)-H(11B)	107.6	H(21D)-C(21A)-H(21E)	109.5
C(12A)-C(11A)-C(9A)	114.19(15)	C(20A)-C(21A)-H(21F)	109.5
C(12A)-C(11A)-H(11C)	108.7	H(21D)-C(21A)-H(21F)	109.5
C(9A)-C(11A)-H(11C)	108.7	H(21E)-C(21A)-H(21F)	109.5

Supplementary Table 11. Torsion angles [^{o}] for **3r**

C(10)-C(1)-C(2)-C(3)	-57.4(2)
C(10A)-C(1A)-C(2A)-C(3A)	-57.8(2)
C(1)-C(2)-C(3)-F(1)	-179.60(15)
C(1)-C(2)-C(3)-C(4)	59.6(2)
C(1A)-C(2A)-C(3A)-F(1A)	-178.41(18)
C(1A)-C(2A)-C(3A)-C(4A)	61.0(2)
F(1)-C(3)-C(4)-C(5)	-177.66(16)
C(2)-C(3)-C(4)-C(5)	-56.7(2)
F(1A)-C(3A)-C(4A)-C(5A)	-178.64(16)
C(2A)-C(3A)-C(4A)-C(5A)	-57.7(2)
C(3)-C(4)-C(5)-C(6)	-129.1(2)
C(3)-C(4)-C(5)-C(10)	51.1(2)
C(3A)-C(4A)-C(5A)-C(6A)	-129.3(2)
C(3A)-C(4A)-C(5A)-C(10A)	51.3(2)
C(4)-C(5)-C(6)-C(7)	-179.45(18)
C(10)-C(5)-C(6)-C(7)	0.4(3)
C(4A)-C(5A)-C(6A)-C(7A)	-179.1(2)
C(10A)-C(5A)-C(6A)-C(7A)	0.2(3)
C(5)-C(6)-C(7)-C(8)	13.9(3)
C(5A)-C(6A)-C(7A)-C(8A)	12.1(3)
C(6)-C(7)-C(8)-C(14)	-163.67(15)
C(6)-C(7)-C(8)-C(9)	-42.4(2)
C(6A)-C(7A)-C(8A)-C(14A)	-159.83(17)
C(6A)-C(7A)-C(8A)-C(9A)	-39.8(2)
C(7)-C(8)-C(9)-C(11)	-171.71(15)
C(14)-C(8)-C(9)-C(11)	-49.51(19)
C(7)-C(8)-C(9)-C(10)	59.71(19)

C(14)-C(8)-C(9)-C(10)	-178.09(14)
C(14A)-C(8A)-C(9A)-C(11A)	-51.77(18)
C(7A)-C(8A)-C(9A)-C(11A)	-173.17(15)
C(14A)-C(8A)-C(9A)-C(10A)	179.43(12)
C(7A)-C(8A)-C(9A)-C(10A)	58.03(18)
C(6)-C(5)-C(10)-C(18)	-107.8(2)
C(4)-C(5)-C(10)-C(18)	72.03(19)
C(6)-C(5)-C(10)-C(1)	133.32(19)
C(4)-C(5)-C(10)-C(1)	-46.87(19)
C(6)-C(5)-C(10)-C(9)	15.0(2)
C(4)-C(5)-C(10)-C(9)	-165.20(14)
C(2)-C(1)-C(10)-C(5)	50.1(2)
C(2)-C(1)-C(10)-C(18)	-68.3(2)
C(2)-C(1)-C(10)-C(9)	169.64(16)
C(11)-C(9)-C(10)-C(5)	-173.09(15)
C(8)-C(9)-C(10)-C(5)	-44.79(18)
C(11)-C(9)-C(10)-C(18)	-52.3(2)
C(8)-C(9)-C(10)-C(18)	76.0(2)
C(11)-C(9)-C(10)-C(1)	68.17(19)
C(8)-C(9)-C(10)-C(1)	-163.53(15)
C(6A)-C(5A)-C(10A)-C(18A)	-106.7(2)
C(4A)-C(5A)-C(10A)-C(18A)	72.6(2)
C(6A)-C(5A)-C(10A)-C(1A)	134.0(2)
C(4A)-C(5A)-C(10A)-C(1A)	-46.7(2)
C(6A)-C(5A)-C(10A)-C(9A)	16.2(2)
C(4A)-C(5A)-C(10A)-C(9A)	-164.50(16)
C(2A)-C(1A)-C(10A)-C(5A)	49.5(2)
C(2A)-C(1A)-C(10A)-C(18A)	-69.1(2)
C(2A)-C(1A)-C(10A)-C(9A)	168.87(17)

C(11A)-C(9A)-C(10A)-C(5A)	-173.18(15)
C(8A)-C(9A)-C(10A)-C(5A)	-45.23(18)
C(11A)-C(9A)-C(10A)-C(18A)	-52.3(2)
C(8A)-C(9A)-C(10A)-C(18A)	75.60(19)
C(11A)-C(9A)-C(10A)-C(1A)	68.17(19)
C(8A)-C(9A)-C(10A)-C(1A)	-163.89(16)
C(8)-C(9)-C(11)-C(12)	49.9(2)
C(10)-C(9)-C(11)-C(12)	178.48(15)
C(8A)-C(9A)-C(11A)-C(12A)	51.7(2)
C(10A)-C(9A)-C(11A)-C(12A)	-179.40(15)
C(9)-C(11)-C(12)-C(13)	-54.3(2)
C(9A)-C(11A)-C(12A)-C(13A)	-53.9(2)
C(11)-C(12)-C(13)-C(19)	-66.7(2)
C(11)-C(12)-C(13)-C(14)	57.1(2)
C(11)-C(12)-C(13)-C(17)	167.34(16)
C(11A)-C(12A)-C(13A)-C(19A)	-69.23(19)
C(11A)-C(12A)-C(13A)-C(14A)	55.00(18)
C(11A)-C(12A)-C(13A)-C(17A)	164.56(15)
C(7)-C(8)-C(14)-C(15)	-56.1(2)
C(9)-C(8)-C(14)-C(15)	-177.58(16)
C(7)-C(8)-C(14)-C(13)	179.22(15)
C(9)-C(8)-C(14)-C(13)	57.73(18)
C(12)-C(13)-C(14)-C(15)	166.33(15)
C(19)-C(13)-C(14)-C(15)	-70.65(18)
C(17)-C(13)-C(14)-C(15)	45.17(16)
C(12)-C(13)-C(14)-C(8)	-61.41(18)
C(19)-C(13)-C(14)-C(8)	61.61(19)
C(17)-C(13)-C(14)-C(8)	177.43(13)
C(7A)-C(8A)-C(14A)-C(15A)	-54.6(2)

C(9A)-C(8A)-C(14A)-C(15A)	-175.54(15)
C(7A)-C(8A)-C(14A)-C(13A)	179.83(16)
C(9A)-C(8A)-C(14A)-C(13A)	58.87(18)
C(12A)-C(13A)-C(14A)-C(15A)	166.72(15)
C(19A)-C(13A)-C(14A)-C(15A)	-70.55(19)
C(17A)-C(13A)-C(14A)-C(15A)	45.38(17)
C(12A)-C(13A)-C(14A)-C(8A)	-60.29(19)
C(19A)-C(13A)-C(14A)-C(8A)	62.4(2)
C(17A)-C(13A)-C(14A)-C(8A)	178.37(14)
C(8)-C(14)-C(15)-C(16)	-165.33(16)
C(13)-C(14)-C(15)-C(16)	-36.17(19)
C(8A)-C(14A)-C(15A)-C(16A)	-162.69(16)
C(13A)-C(14A)-C(15A)-C(16A)	-32.53(19)
C(14)-C(15)-C(16)-C(17)	12.0(2)
C(14A)-C(15A)-C(16A)-C(17A)	5.8(2)
C(15)-C(16)-C(17)-C(20)	143.17(16)
C(15)-C(16)-C(17)-C(13)	15.8(2)
C(12)-C(13)-C(17)-C(20)	82.8(2)
C(19)-C(13)-C(17)-C(20)	-44.2(2)
C(14)-C(13)-C(17)-C(20)	-162.78(14)
C(12)-C(13)-C(17)-C(16)	-151.15(17)
C(19)-C(13)-C(17)-C(16)	81.78(18)
C(14)-C(13)-C(17)-C(16)	-36.77(17)
C(15A)-C(16A)-C(17A)-C(20A)	150.36(17)
C(15A)-C(16A)-C(17A)-C(13A)	22.5(2)
C(12A)-C(13A)-C(17A)-C(20A)	78.3(2)
C(19A)-C(13A)-C(17A)-C(20A)	-48.6(2)
C(14A)-C(13A)-C(17A)-C(20A)	-167.35(17)
C(12A)-C(13A)-C(17A)-C(16A)	-155.32(16)

C(19A)-C(13A)-C(17A)-C(16A)	77.80(19)
C(14A)-C(13A)-C(17A)-C(16A)	-40.99(18)
C(16)-C(17)-C(20)-O(1)	-9.1(3)
C(13)-C(17)-C(20)-O(1)	112.3(2)
C(16)-C(17)-C(20)-C(21)	169.97(19)
C(13)-C(17)-C(20)-C(21)	-68.7(2)
C(16A)-C(17A)-C(20A)-O(1A)	-6.5(3)
C(13A)-C(17A)-C(20A)-O(1A)	114.8(2)
C(16A)-C(17A)-C(20A)-C(21A)	171.73(19)
C(13A)-C(17A)-C(20A)-C(21A)	-67.0(2)

Supplementary Table 12. Crystal data and structure refinement for **3t**

Empirical formula	$C_{19}H_{29}FO$	
Formula weight	292.42	
Temperature	296(2) K	
Wavelength	1.54178 Å	
Crystal system, space group	Monoclinic, P 21	
Unit cell dimensions	a = 10.608(2) Å	$\alpha = 90^{o}$
	b = 5.962(2) Å	$\beta = 100.12^{o}$
	c = 13.638(3) Å	$\gamma = 90^{o}$
Volume	849.1(4) $Å^{3}$	
Z, Calculated density	2, 1.144 Mg/m^{3}	
Absorption coefficient	0.596 mm^{-1}	
F(000)	320	
Crystal size	0.29 × 0.22 × 0.18 mm^{3}	
Theta range for data collection	3.29 to 66.87^{o}	
Limiting indices	-12<=h<=7, -5<=k<=6, -16<=l<=16	
Reflections collected / unique	4167 / 2322 [R(int) = 0.0216]	
Completeness to theta = 67.39	95.9 %	
Absorption correction	Semi-empirical from equivalents	
Max. and min. transmission	0.7529 and 0.6434	
Refinement method	Full-matrix least-squares on F^{2}	
Data / restraints / parameters	2322 / 1 / 191	
Goodness-of-fit on F^{2}	1.073	
Final R indices [I>2sigma(I)]	R1 = 0.0410, wR2 = 0.1196	
R indices (all data)	R1 = 0.0417, wR2 = 0.1208	
Absolute structure parameter	0.0(2)	
Extinction coefficient	0.014(2)	
Largest diff. peak and hole	0.240 and -0.177 e.$Å^{-3}$	

Supplementary Table 13. Atomic coordinates ($\times 10^4$) and equivalent isotropic displacement parameters ($Å^2 \times 10^3$) for **3t**

	x	y	z	U(eq)
F(1)	-3557(2)	322(3)	359(1)	106(1)
O(1)	4928(1)	2155(4)	3789(1)	89(1)
C(1)	-1339(2)	3300(4)	462(1)	60(1)
C(2)	-2693(2)	3711(5)	-99(1)	72(1)
C(3)	-3692(2)	2683(5)	416(2)	74(1)
C(4)	-3527(2)	3354(5)	1494(2)	73(1)
C(5)	-2166(1)	2914(4)	2049(1)	53(1)
C(6)	-2016(2)	3445(5)	3151(1)	77(1)
C(7)	-699(2)	2771(5)	3705(1)	68(1)
C(8)	387(1)	3764(3)	3244(1)	47(1)
C(9)	188(1)	3232(3)	2116(1)	42(1)
C(10)	-1125(2)	4067(3)	1558(1)	45(1)
C(11)	1344(2)	3994(4)	1654(1)	61(1)
C(12)	2628(2)	3139(4)	2210(1)	64(1)
C(13)	2810(2)	3779(3)	3301(1)	52(1)
C(14)	1680(1)	2851(3)	3738(1)	46(1)
C(15)	2117(2)	3064(5)	4867(1)	71(1)
C(16)	3537(2)	2394(5)	4999(1)	69(1)
C(17)	3915(2)	2702(4)	3997(1)	60(1)
C(18)	-1218(2)	6624(4)	1586(2)	69(1)
C(19)	3007(2)	6316(4)	3446(2)	86(1)
H(1A)	-740	4086	123	72
H(1B)	-1152	1710	442	72
H(2A)	-2842	5313	-164	86
H(2B)	-2774	3086	-764	86

H(3A)	-4545	3126	72	89
H(4A)	-4129	2521	1812	88
H(4B)	-3721	4937	1537	88
H(5A)	-2024	1296	2003	64
H(6A)	-2668	2652	3432	93
H(6B)	-2141	5040	3237	93
H(7A)	-630	1149	3705	82
H(7B)	-607	3263	4391	82
H(8A)	388	5396	3331	56
H(9A)	164	1593	2062	50
H(11A)	1228	3473	971	73
H(11B)	1364	5621	1642	73
H(12A)	2662	1520	2150	77
H(12B)	3316	3773	1915	77
H(14A)	1655	1238	3596	55
H(15A)	1643	2055	5226	85
H(15B)	2019	4588	5090	85
H(16A)	4052	3342	5492	83
H(16B)	3651	844	5213	83
H(18A)	-2040	7091	1233	103
H(18B)	-557	7269	1276	103
H(18C)	-1115	7119	2265	103
H(19A)	3724	6786	3154	129
H(19B)	3170	6657	4145	129
H(19C)	2251	7093	3131	129

Supplementary Table 14. Bond lengths [Å] and angles [^{o}] for **3t**

F(1)-C(3)	1.419(3)	C(9)-C(10)	1.547(2)
O(1)-C(17)	1.203(2)	C(9)-H(9A)	0.9800
C(1)-C(2)	1.524(3)	C(10)-C(18)	1.529(3)
C(1)-C(10)	1.541(2)	C(11)-C(12)	1.525(3)
C(1)-H(1A)	0.9700	C(11)-H(11A)	0.9700
C(1)-H(1B)	0.9700	C(11)-H(11B)	0.9700
C(2)-C(3)	1.500(3)	C(12)-C(13)	1.514(3)
C(2)-H(2A)	0.9700	C(12)-H(12A)	0.9700
C(2)-H(2B)	0.9700	C(12)-H(12B)	0.9700
C(3)-C(4)	1.504(3)	C(13)-C(17)	1.516(3)
C(4)-C(5)	0.9800	C(13)-C(14)	1.533(2)
C(4)-H(4A)	1.530(2)	C(13)-C(19)	1.535(3)
C(4)-H(4B)	0.9700	C(14)-C(15)	1.533(2)
C(3)-H(3A)	0.9700	C(14)-H(14A)	0.9800
C(5)-C(6)	1.516(3)	C(15)-C(16)	1.537(3)
C(5)-C(10)	1.550(2)	C(15)-H(15A)	0.9700
C(5)-H(5A)	0.9800	C(15)-H(15B)	0.9700
C(6)-C(7)	1.521(3)	C(16)-C(17)	1.502(3)
C(6)-H(6A)	0.9700	C(16)-H(16A)	0.9700
C(6)-H(6B)	0.9700	C(16)-H(16B)	0.9700
C(7)-C(8)	1.525(2)	C(18)-H(18A)	0.9600
C(7)-H(7A)	0.9700	C(18)-H(18B)	0.9600
C(7)-H(7B)	0.9700	C(18)-H(18C)	0.9600
C(8)-C(14)	1.519(2)	C(19)-H(19A)	0.9600
C(8)-C(9)	1.549(2)	C(19)-H(19B)	0.9600
C(8)-H(8A)	0.9800	C(19)-H(19C)	0.9600
C(9)-C(11)	1.543(2)		

C(2)-C(1)-C(10)	113.88(15)	C(1)-C(10)-C(9)	110.37(13)
C(2)-C(1)-H(1A)	108.8	C(18)-C(10)-C(5)	112.21(16)
C(10)-C(1)-H(1A)	108.8	C(1)-C(10)-C(5)	107.03(15)
C(2)-C(1)-H(1B)	108.8	C(9)-C(10)-C(5)	107.02(13)
C(10)-C(1)-H(1B)	108.8	C(12)-C(11)-C(9)	113.78(15)
H(1A)-C(1)-H(1B)	107.7	C(12)-C(11)-H(11A)	108.8
C(3)-C(2)-C(1)	112.33(17)	C(9)-C(11)-H(11A)	108.8
C(3)-C(2)-H(2A)	109.1	C(12)-C(11)-H(11B)	108.8
C(1)-C(2)-H(2A)	109.1	C(9)-C(11)-H(11B)	108.8
C(3)-C(2)-H(2B)	109.1	H(11A)-C(11)-H(11B)	107.7
C(1)-C(2)-H(2B)	109.1	C(13)-C(12)-C(11)	110.66(16)
H(2A)-C(2)-H(2B)	107.9	C(13)-C(12)-H(12A)	109.5
F(1)-C(3)-C(2)	107.0(2)	C(11)-C(12)-H(12A)	109.5
F(1)-C(3)-C(4)	108.8(2)	C(13)-C(12)-H(12B)	109.5
C(2)-C(3)-C(4)	112.25(19)	C(11)-C(12)-H(12B)	109.5
F(1)-C(3)-H(3A)	109.6	H(12A)-C(12)-H(12B)	108.1
C(2)-C(3)-H(3A)	109.6	C(12)-C(13)-C(17)	117.38(17)
C(4)-C(3)-H(3A)	109.6	C(12)-C(13)-C(14)	108.51(14)
C(3)-C(4)-C(5)	112.07(16)	C(17)-C(13)-C(14)	100.05(14)
C(3)-C(4)-H(4A)	109.2	C(12)-C(13)-C(19)	111.50(19)
C(5)-C(4)-H(4A)	109.2	C(17)-C(13)-C(19)	105.27(16)
C(3)-C(4)-H(4B)	109.2	C(14)-C(13)-C(19)	113.81(17)
C(5)-C(4)-H(4B)	109.2	C(8)-C(14)-C(15)	120.41(14)
H(4A)-C(4)-H(4B)	107.9	C(8)-C(14)-C(13)	113.86(14)
C(6)-C(5)-C(4)	112.33(15)	C(15)-C(14)-C(13)	103.97(14)
C(6)-C(5)-C(10)	112.16(16)	C(8)-C(14)-H(14A)	105.8
C(4)-C(5)-C(10)	113.06(16)	C(15)-C(14)-H(14A)	105.8
C(6)-C(5)-H(5A)	106.2	C(13)-C(14)-H(14A)	105.8

C(4)-C(5)-H(5A)	106.2	C(14)-C(15)-C(16)	102.36(14)
C(10)-C(5)-H(5A)	106.2	C(14)-C(15)-H(15A)	111.3
C(5)-C(6)-C(7)	111.32(15)	C(16)-C(15)-H(15A)	111.3
C(5)-C(6)-H(6A)	109.4	C(14)-C(15)-H(15B)	111.3
C(7)-C(6)-H(6A)	109.4	C(16)-C(15)-H(15B)	111.3
C(5)-C(6)-H(6B)	109.4	H(15A)-C(15)-H(15B)	109.2
C(7)-C(6)-H(6B)	109.4	C(17)-C(16)-C(15)	105.92(14)
H(6A)-C(6)-H(6B)	108.0	C(17)-C(16)-H(16A)	110.6
C(6)-C(7)-C(8)	112.80(17)	C(15)-C(16)-H(16A)	110.6
C(6)-C(7)-H(7A)	109.0	C(17)-C(16)-H(16B)	110.6
C(8)-C(7)-H(7A)	109.0	C(15)-C(16)-H(16B)	110.6
C(6)-C(7)-H(7B)	109.0	H(16A)-C(16)-H(16B)	108.7
C(8)-C(7)-H(7B)	109.0	O(1)-C(17)-C(16)	125.09(19)
H(7A)-C(7)-H(7B)	107.8	O(1)-C(17)-C(13)	126.18(18)
C(14)-C(8)-C(7)	111.48(15)	C(16)-C(17)-C(13)	108.73(14)
C(14)-C(8)-C(9)	108.79(12)	C(10)-C(18)-H(18A)	109.5
C(7)-C(8)-C(9)	110.42(14)	C(10)-C(18)-H(18B)	109.5
C(14)-C(8)-H(8A)	108.7	H(18A)-C(18)-H(18B)	109.5
C(7)-C(8)-H(8A)	108.7	C(10)-C(18)-H(18C)	109.5
C(9)-C(8)-H(8A)	108.7	H(18A)-C(18)-H(18C)	109.5
C(11)-C(9)-C(10)	114.60(14)	H(18B)-C(18)-H(18C)	109.5
C(11)-C(9)-C(8)	111.45(14)	C(13)-C(19)-H(19A)	109.5
C(10)-C(9)-C(8)	112.12(12)	C(13)-C(19)-H(19B)	109.5
C(11)-C(9)-H(9A)	106.0	H(19A)-C(19)-H(19B)	109.5
C(10)-C(9)-H(9A)	106.0	C(13)-C(19)-H(19C)	109.5
C(8)-C(9)-H(9A)	106.0	H(19A)-C(19)-H(19C)	109.5
C(18)-C(10)-C(1)	108.70(16)	H(19B)-C(19)-H(19C)	109.5
C(18)-C(10)-C(9)	111.43(15)		

Supplementary Table 15. Torsion angles [^{o}] for **3t**

C(10)-C(1)-C(2)-C(3)	-54.5(3)
C(1)-C(2)-C(3)-F(1)	-67.5(2)
C(1)-C(2)-C(3)-C(4)	51.8(3)
F(1)-C(3)-C(4)-C(5)	65.8(3)
C(2)-C(3)-C(4)-C(5)	-52.5(3)
C(3)-C(4)-C(5)-C(6)	-176.3(2)
C(3)-C(4)-C(5)-C(10)	55.5(3)
C(4)-C(5)-C(6)-C(7)	174.2(2)
C(10)-C(5)-C(6)-C(7)	-57.2(3)
C(5)-C(6)-C(7)-C(8)	53.3(3)
C(6)-C(7)-C(8)-C(14)	-173.48(18)
C(6)-C(7)-C(8)-C(9)	-52.4(3)
C(14)-C(8)-C(9)-C(11)	-51.2(2)
C(7)-C(8)-C(9)-C(11)	-173.84(17)
C(14)-C(8)-C(9)-C(10)	178.84(13)
C(7)-C(8)-C(9)-C(10)	56.2(2)
C(2)-C(1)-C(10)-C(18)	-67.1(2)
C(2)-C(1)-C(10)-C(9)	170.38(16)
C(2)-C(1)-C(10)-C(5)	54.3(2)
C(11)-C(9)-C(10)-C(18)	-63.6(2)
C(8)-C(9)-C(10)-C(18)	64.73(19)
C(11)-C(9)-C(10)-C(1)	57.3(2)
C(8)-C(9)-C(10)-C(1)	-174.40(14)
C(11)-C(9)-C(10)-C(5)	173.40(15)
C(8)-C(9)-C(10)-C(5)	-58.28(19)
C(6)-C(5)-C(10)-C(18)	-63.7(2)
C(4)-C(5)-C(10)-C(18)	64.5(2)

C(6)-C(5)-C(10)-C(1)	177.11(17)
C(4)-C(5)-C(10)-C(1)	-54.6(2)
C(6)-C(5)-C(10)-C(9)	58.8(2)
C(4)-C(5)-C(10)-C(9)	-172.95(15)
C(10)-C(9)-C(11)-C(12)	-179.56(15)
C(8)-C(9)-C(11)-C(12)	51.8(2)
C(9)-C(11)-C(12)-C(13)	-54.7(3)
C(11)-C(12)-C(13)-C(17)	168.98(16)
C(11)-C(12)-C(13)-C(14)	56.6(2)
C(11)-C(12)-C(13)-C(19)	-69.5(2)
C(7)-C(8)-C(14)-C(15)	-55.9(2)
C(9)-C(8)-C(14)-C(15)	-177.92(17)
C(7)-C(8)-C(14)-C(13)	179.62(15)
C(9)-C(8)-C(14)-C(13)	57.6(2)
C(12)-C(13)-C(14)-C(8)	-60.8(2)
C(17)-C(13)-C(14)-C(8)	175.71(14)
C(19)-C(13)-C(14)-C(8)	64.0(2)
C(12)-C(13)-C(14)-C(15)	166.33(17)
C(17)-C(13)-C(14)-C(15)	42.8(2)
C(19)-C(13)-C(14)-C(15)	-68.9(2)
C(8)-C(14)-C(15)-C(16)	-168.79(18)
C(13)-C(14)-C(15)-C(16)	-39.8(2)
C(14)-C(15)-C(16)-C(17)	20.5(2)
C(15)-C(16)-C(17)-O(1)	-173.0(2)
C(15)-C(16)-C(17)-C(13)	6.2(2)
C(12)-C(13)-C(17)-O(1)	32.1(3)
C(14)-C(13)-C(17)-O(1)	149.2(2)
C(19)-C(13)-C(17)-O(1)	-92.6(3)
C(12)-C(13)-C(17)-C(16)	-147.15(19)

C(14)-C(13)-C(17)-C(16)	-30.1(2)
C(19)-C(13)-C(17)-C(16)	88.2(2)

Supplementary Table 16. Crystal data and structure refinement for **7j**

Empirical formula	$C_{21}H_{21}FO_2$	
Formula weight	324.38	
Temperature	296(2) K	
Wavelength	1.54178 Å	
Crystal system, space group	Monoclinic, P 21/n	
Unit cell dimensions	a = 6.2779(13) Å	$\alpha = 90^o$
	b = 16.762(3) Å	$\beta = 96.73(3)^o$
	c = 16.993(3) Å	$\gamma = 90^o$
Volume	1775.9(6) $Å^3$	
Z, Calculated density	4, 1.213 Mg/m^3	
Absorption coefficient	0.677 mm^{-1}	
F(000)	688	
Crystal size	0.36 × 0.21 × 0.18 mm^3	
Theta range for data collection	5.24 to 65.99^o	
Limiting indices	-7<=h<=6, -19<=k<=19, -19<=l<=20	
Reflections collected / unique	11772 / 2954 [R(int) = 0.0217]	
Completeness to theta = 67.39	95.3 %	
Absorption correction	Semi-empirical from equivalents	
Max. and min. transmission	0.7529 and 0.6298	
Refinement method	Full-matrix least-squares on F^2	
Data / restraints / parameters	2954 / 0 / 227	
Goodness-of-fit on F^2	1.062	
Final R indices [I>2sigma(I)]	R1 = 0.0561, wR2 = 0.1616	

R indices (all data)	R1 = 0.0574, wR2 = 0.1629
Extinction coefficient	0.0113(10)
Largest diff. peak and hole	0.310 and -0.162 e.Å^{-3}

Supplementary Table 17. Atomic coordinates ($\times 10^4$) and equivalent isotropic displacement parameters (Å^2 $\times 10^3$) for **3r**

	x	y	z	U(eq)
F(1)	-2147(7)	8362(3)	3535(2)	210(2)
F(2)	4364(17)	9165(7)	4817(7)	251(8)
O(1)	1041(3)	8080(1)	4726(1)	70(1)
O(2)	2857(3)	7140(1)	4163(1)	86(1)
C(1)	1344(4)	8632(1)	4093(1)	70(1)
C(2)	-769(5)	8928(2)	3777(2)	120(1)
C(3)	-615(7)	9557(3)	3137(2)	146(2)
C(4)	775(6)	10219(2)	3440(2)	111(1)
C(5)	2925(7)	9923(3)	3752(3)	153(2)
C(6)	2811(6)	9276(2)	4384(2)	124(1)
C(7)	1818(3)	7342(1)	4677(1)	58(1)
C(8)	1231(3)	6815(1)	5324(1)	54(1)
C(9)	2115(3)	6090(1)	5351(1)	59(1)
C(10)	-181(3)	7124(1)	5902(1)	52(1)
C(11)	-2247(3)	7388(1)	5665(1)	67(1)
C(12)	-3528(4)	7647(2)	6226(2)	81(1)
C(13)	-2752(4)	7649(1)	7018(2)	82(1)
C(14)	-717(4)	7396(1)	7255(1)	71(1)
C(15)	576(3)	7138(1)	6702(1)	60(1)
C(16)	1834(3)	5401(1)	5857(1)	56(1)
C(17)	-7(3)	5246(1)	6214(1)	63(1)

C(18)	-182(4)	4552(1)	6640(1)	74(1)
C(19)	1468(4)	4006(1)	6723(1)	75(1)
C(20)	3295(4)	4146(1)	6378(1)	74(1)
C(21)	3468(4)	4835(1)	5949(1)	68(1)
H(1A)	1965	8343	3673	84
H(2A)	-1351	9199	4216	143
H(3A)	-43	9315	2688	176
H(3B)	-2036	9759	2957	176
H(4A)	137	10492	3858	134
H(4B)	903	10599	3018	134
H(5A)	3786	10365	3979	183
H(5B)	3623	9707	3319	183
H(6A)	1931	9551	4740	148
H(9A)	3080	6008	4983	71
H(11A)	-2774	7392	5130	80
H(12A)	-4919	7820	6066	97
H(13A)	-3618	7824	7391	98
H(14A)	-196	7397	7791	86
H(15A)	1970	6972	6867	71
H(17A)	-1128	5611	6165	76
H(18A)	-1425	4453	6872	88
H(19A)	1340	3542	7014	90
H(20A)	4412	3778	6433	89
H(21A)	4710	4925	5713	81

Supplementary Table 18. Bond lengths [Å] and angles [^{o}] for **7j**

F(1)-C(2)	1.317(4)	C(9)-H(9A)	0.9300
F(2)-C(6)	1.165(7)	C(10)-C(11)	1.385(3)

O(1)-C(7)	1.335(2)	C(10)-C(15)	1.386(3)
O(1)-C(1)	1.449(2)	C(11)-C(12)	1.387(3)
O(2)-C(7)	1.199(2)	C(11)-H(11A)	0.9300
C(1)-C(2)	1.458(4)	C(12)-C(13)	1.376(4)
C(1)-C(6)	1.467(4)	C(12)-H(12A)	0.9300
C(1)-H(1A)	0.9800	C(13)-C(14)	1.362(4)
C(2)-C(3)	1.526(4)	C(13)-H(13A)	0.9300
C(2)-H(2A)	0.9800	C(14)-C(15)	1.382(3)
C(3)-C(4)	1.468(5)	C(14)-H(14A)	0.9300
C(3)-H(3A)	0.9700	C(15)-H(15A)	0.9300
C(3)-H(3B)	0.9700	C(16)-C(17)	1.391(3)
C(4)-C(5)	1.477(5)	C(16)-C(21)	1.392(3)
C(4)-H(4A)	0.9700	C(17)-C(18)	1.380(3)
C(4)-H(4B)	0.9700	C(17)-H(17A)	0.9300
C(5)-C(6)	1.534(4)	C(18)-C(19)	1.377(3)
C(5)-H(5A)	0.9700	C(18)-H(18A)	0.9300
C(5)-H(5B)	0.9700	C(19)-C(20)	1.369(3)
C(6)-H(6A)	0.9800	C(19)-H(19A)	0.9300
C(7)-C(8)	1.489(2)	C(20)-C(21)	1.377(3)
C(8)-C(9)	1.335(3)	C(20)-H(20A)	0.9300
C(8)-C(10)	1.491(2)	C(21)-H(21A)	0.9300
C(9)-C(16)	1.464(3)		

C(7)-O(1)-C(1)	117.67(14)	C(9)-C(8)-C(10)	124.76(16)
O(1)-C(1)-C(2)	107.29(19)	C(7)-C(8)-C(10)	119.87(15)
O(1)-C(1)-C(6)	110.60(19)	C(8)-C(9)-C(16)	131.36(17)
C(2)-C(1)-C(6)	112.4(2)	C(8)-C(9)-H(9A)	114.3
O(1)-C(1)-H(1A)	108.8	C(16)-C(9)-H(9A)	114.3
C(2)-C(1)-H(1A)	108.8	C(11)-C(10)-C(15)	118.75(17)

C(6)-C(1)-H(1A)	108.8	C(11)-C(10)-C(8)	121.91(16)
F(1)-C(2)-C(1)	113.9(4)	C(15)-C(10)-C(8)	119.33(17)
F(1)-C(2)-C(3)	111.7(3)	C(10)-C(11)-C(12)	120.0(2)
C(1)-C(2)-C(3)	111.5(3)	C(10)-C(11)-H(11A)	120.0
F(1)-C(2)-H(2A)	106.4	C(12)-C(11)-H(11A)	120.0
C(1)-C(2)-H(2A)	106.4	C(13)-C(12)-C(11)	120.3(2)
C(3)-C(2)-H(2A)	106.4	C(13)-C(12)-H(12A)	119.8
C(4)-C(3)-C(2)	110.9(3)	C(11)-C(12)-H(12A)	119.8
C(4)-C(3)-H(3A)	109.5	C(14)-C(13)-C(12)	120.1(2)
C(2)-C(3)-H(3A)	109.5	C(14)-C(13)-H(13A)	120.0
C(4)-C(3)-H(3B)	109.5	C(12)-C(13)-H(13A)	120.0
C(2)-C(3)-H(3B)	109.5	C(13)-C(14)-C(15)	120.1(2)
H(3A)-C(3)-H(3B)	108.0	C(13)-C(14)-H(14A)	120.0
C(3)-C(4)-C(5)	110.6(3)	C(15)-C(14)-H(14A)	120.0
C(3)-C(4)-H(4A)	109.5	C(14)-C(15)-C(10)	120.8(2)
C(5)-C(4)-H(4A)	109.5	C(14)-C(15)-H(15A)	119.6
C(3)-C(4)-H(4B)	109.5	C(10)-C(15)-H(15A)	119.6
C(5)-C(4)-H(4B)	109.5	C(17)-C(16)-C(21)	117.57(18)
H(4A)-C(4)-H(4B)	108.1	C(17)-C(16)-C(9)	124.58(17)
C(4)-C(5)-C(6)	111.9(3)	C(21)-C(16)-C(9)	117.73(17)
C(4)-C(5)-H(5A)	109.2	C(18)-C(17)-C(16)	120.5(2)
C(6)-C(5)-H(5A)	109.2	C(18)-C(17)-H(17A)	119.8
C(4)-C(5)-H(5B)	109.2	C(16)-C(17)-H(17A)	119.8
C(6)-C(5)-H(5B)	109.2	C(19)-C(18)-C(17)	120.6(2)
H(5A)-C(5)-H(5B)	107.9	C(19)-C(18)-H(18A)	119.7
F(2)-C(6)-C(1)	122.7(6)	C(17)-C(18)-H(18A)	119.7
F(2)-C(6)-C(5)	116.8(5)	C(20)-C(19)-C(18)	120.0(2)
C(1)-C(6)-C(5)	111.3(3)	C(20)-C(19)-H(19A)	120.0
F(2)-C(6)-H(6A)	100.2	C(18)-C(19)-H(19A)	120.0

C(1)-C(6)-H(6A)	100.2	C(19)-C(20)-C(21)	119.5(2)
C(5)-C(6)-H(6A)	100.2	C(19)-C(20)-H(20A)	120.3
O(2)-C(7)-O(1)	122.74(17)	C(21)-C(20)-H(20A)	120.3
O(2)-C(7)-C(8)	124.98(17)	C(20)-C(21)-C(16)	121.86(19)
O(1)-C(7)-C(8)	112.28(15)	C(20)-C(21)-H(21A)	119.1
C(9)-C(8)-C(7)	115.32(16)	C(16)-C(21)-H(21A)	119.1

Supplementary Table 19. Torsion angles [^{o}] for **7j**

C(7)-O(1)-C(1)-C(2)	124.3(3)
C(7)-O(1)-C(1)-C(6)	-112.7(3)
O(1)-C(1)-C(2)-F(1)	-56.0(4)
C(6)-C(1)-C(2)-F(1)	-177.8(3)
O(1)-C(1)-C(2)-C(3)	176.5(3)
C(6)-C(1)-C(2)-C(3)	54.7(4)
F(1)-C(2)-C(3)-C(4)	174.8(5)
C(1)-C(2)-C(3)-C(4)	-56.5(5)
C(2)-C(3)-C(4)-C(5)	56.6(5)
C(3)-C(4)-C(5)-C(6)	-55.3(5)
O(1)-C(1)-C(6)-F(2)	41.7(10)
C(2)-C(1)-C(6)-F(2)	161.6(10)
O(1)-C(1)-C(6)-C(5)	-172.7(3)
C(2)-C(1)-C(6)-C(5)	-52.8(4)
C(4)-C(5)-C(6)-F(2)	-159.1(10)
C(4)-C(5)-C(6)-C(1)	53.1(5)
C(1)-O(1)-C(7)-O(2)	4.8(3)
C(1)-O(1)-C(7)-C(8)	-174.87(17)
O(2)-C(7)-C(8)-C(9)	6.0(3)
O(1)-C(7)-C(8)-C(9)	-174.31(18)

O(2)-C(7)-C(8)-C(10)	-176.4(2)
O(1)-C(7)-C(8)-C(10)	3.3(3)
C(7)-C(8)-C(9)-C(16)	-175.80(19)
C(10)-C(8)-C(9)-C(16)	6.7(4)
C(9)-C(8)-C(10)-C(11)	-122.2(2)
C(7)-C(8)-C(10)-C(11)	60.4(3)
C(9)-C(8)-C(10)-C(15)	56.9(3)
C(7)-C(8)-C(10)-C(15)	-120.42(19)
C(15)-C(10)-C(11)-C(12)	-1.2(3)
C(8)-C(10)-C(11)-C(12)	177.97(19)
C(10)-C(11)-C(12)-C(13)	0.6(4)
C(11)-C(12)-C(13)-C(14)	-0.1(4)
C(12)-C(13)-C(14)-C(15)	0.2(3)
C(13)-C(14)-C(15)-C(10)	-0.8(3)
C(11)-C(10)-C(15)-C(14)	1.3(3)
C(8)-C(10)-C(15)-C(14)	-177.87(17)
C(8)-C(9)-C(16)-C(17)	26.5(3)
C(8)-C(9)-C(16)-C(21)	-157.7(2)
C(21)-C(16)-C(17)-C(18)	-0.1(3)
C(9)-C(16)-C(17)-C(18)	175.68(18)
C(16)-C(17)-C(18)-C(19)	0.5(3)
C(17)-C(18)-C(19)-C(20)	-0.4(3)
C(18)-C(19)-C(20)-C(21)	0.0(4)
C(19)-C(20)-C(21)-C(16)	0.4(3)
C(17)-C(16)-C(21)-C(20)	-0.3(3)
C(9)-C(16)-C(21)-C(20)	-176.39(19)

Supplementary Methods

General Considerations

Unless otherwise mentioned, solvents and reagents were purchased from commercial sources and used as received. Toluene was distilled from sodium/benzophenone and stored over MS 4Å. THF was obtained through distillation over sodium/benzophenone. DCM (dichloromethane), CH_3CN and triethylamine were obtained through distillation over CaH_2. DCE (1,2-dichloroethane), PhCN and chlorobenzene were distilled from CaH_2 and stored over MS 4Å. The water was used after distillation. All the melting points were uncorrected. ^{1}H NMR spectra were recorded at 400 MHz or 300 MHz. ^{13}C NMR spectra were recorded at 100 MHz. ^{19}F NMR spectra were recorded at 376 MHz or 282 MHz. ^{1}H NMR chemical shifts were determined relative to internal $(CH_3)_4Si$ (TMS) at δ 0.0 or to the signal of the residual protonated solvent: $CDCl_3$ δ 7.26. ^{13}C NMR chemical shifts were determined relative to $CDCl_3$ at δ 77.0. ^{19}F NMR chemical shifts were determined relative to external $CFCl_3$ at δ 0.0. Data for ^{1}H, ^{13}C and ^{19}F NMR were recorded as follows: chemical shift (δ, ppm), multiplicity (s = singlet, d = doublet, t = triplet, m = multiplet, q = quartet, br = broad). Mass spectra were obtained on a mass spectrometer. High-resolution mass data were recorded on a high-resolution mass spectrometer in the EI or ESI mode.

1. Synthesis of CpFluors 1.

1.1. Synthesis of 1,2-Diarylethynes SI-1.

Diarylethyne **SI-1a** was commercially available. Diarylethynes **SI-1b, SI-1c, SI-1d, SI-1e, SI-1f, SI-1g, SI-1h** and **SI-1i** were synthesized according to the following procedures.[1]

1.1a. Synthesis of 1,2-Bis(2-methoxyphenyl)ethyne (SI-1b)

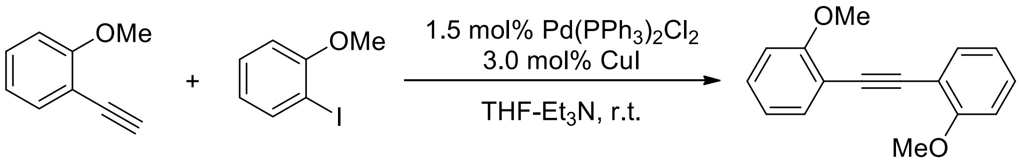

SI-2b **SI-3b** **SI-1b**

A 3-necked 250-mL round bottomed flask (pre dried by hot air gun under vacuo) fitted with magnetic stirrer was evacuated/N_2 filled (3 times), then 1-iodo-2-methoxybenzene (**SI-3b**, 11.70 g, 50 mmol, 1.0 equiv.), $Pd(PPh_3)_2Cl_2$ (526.4 mg, 0.75 mmol, 1.5 mol%), CuI (285.7 mg, 1.5 mmol, 3.0 mol%), THF (40 mL) and Et_3N (100 mL) were added, a solution of 1-ethynyl-2-methoxybenzene[2] (**SI-2b**, 7.27 g, 55 mmol, 1.1 equiv.) in THF (20 mL) was added dropwise by syringe over 75 min at room temperature. After the reaction was complete as monitored by TLC, evaporate the solvent, CH_2Cl_2 (250 mL) was added to dissolve the product, the precipitate was removed by filtration through a pad of celite, the filtrate was washed with water (150 mL×3), dried over $MgSO_4$. After remove the solvent and recrystallized from anhydrous ethyl alcohol for several times, 1,2-bis(2-methoxyphenyl)ethyne (**SI-1b**) was got as white solid (8.53 g, 72%). **^{1}H NMR** ($CDCl_3$, 300 MHz) δ = 7.53 (dd, J = 7.5, 1.3 Hz, 2H), 7.29 (td, J = 7.4, 1.5 Hz, 2H), 6.96–6.88 (m, 4H), 3.92 (s, 6H).[3]

1.1b. Synthesis of 1,2-Bis(4-methoxyphenyl)ethyne (SI-1c)

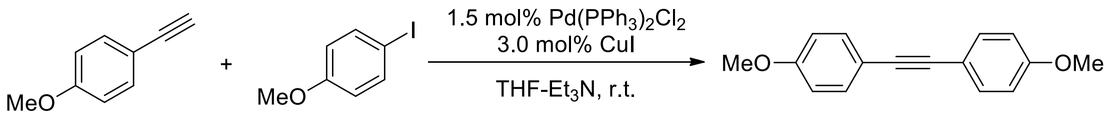

SI-2c **SI-3c** **SI-1c**

A 3-necked 250-mL round bottomed flask (pre dried by hot air gun under vacuo) fitted with magnetic stirrer was evacuated/N_2 filled (3 times), then 1-iodo-4-methoxybenzene (**SI-3c**, 11.70 g, 50 mmol, 1.0 equiv.), $Pd(PPh_3)_2Cl_2$ (526.4 mg, 0.75 mmol, 1.5 mol%), CuI (285.7 mg, 1.5 mmol, 3.0 mol%), THF (40 mL) and Et_3N (100 mL) were added, a solution of 1-ethynyl-4-methoxybenzene (**SI-2c**, 6.81 g, 51.5 mmol, 1.03 equiv.) in THF (20 mL) was added dropwise by syringe over 75 min

at room temperature. After the reaction was complete as monitored by TLC, evaporate the solvent, CH_2Cl_2 (250 mL) was added to dissolve the product, the precipitate was removed by filtration through a pad of celite, the filtrate was washed with water (150 mL×3), dried over $MgSO_4$. After remove the solvent and recrystallized from anhydrous ethyl alcohol for several times, 1,2-bis(4-methoxyphenyl)ethyne (**SI-1c**) was got as white solid (9.65 g, 81%). **^{1}H NMR** ($CDCl_3$, 400 MHz) δ = 7.46 (d, *J* = 8.7 Hz, 4H), 6.87 (d, *J* = 8.7 Hz, 4H), 3.82 (s, 6H).[1]

1.1c. Synthesis of 1,2-Bis(3,4-dimethoxyphenyl)ethyne (SI-1d)

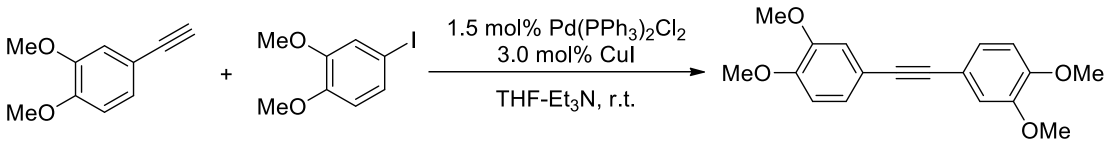

A 3-necked 250-mL round bottomed flask (pre dried by hot air gun under vacuo) fitted with magnetic stirrer was evacuated/N_2 filled (3 times), then 4-iodo-1,2-dimethoxybenzene[4] (**SI-3d**, 10.56 g, 40 mmol, 1.0 equiv.), $Pd(PPh_3)_2Cl_2$ (842 mg, 1.2 mmol, 3 mol%), CuI (229 mg, 1.2 mmol, 3.0 mol%), THF (40 mL) and Et_3N (100 mL) were added, a solution of 4-ethynyl-1,2-dimethoxybenzene[5] (**SI-2d**, 7.14 g, 44 mmol, 1.1 equiv.) in THF (20 mL) was added dropwise by syringe over 40 min at room temperature. After the reaction was complete as monitored by TLC, the precipitate was collected by filtration, then washed with ether and water, use ethyl acetate (500 mL) to dissolve the crude product, add activated carbon (~ 1.0 g), reflux for 1 h, the precipitate was filtered through a pad of celite when the solution was hot, after the filtrate was evaporated and the residue was recrystallized from anhydrous ethyl alcohol for several times, 1,2-bis(3,4-dimethoxyphenyl)ethyne (**SI-1d**) was got as acicular crystal (9.04 g, 76%). **^{1}H NMR** ($CDCl_3$, 300 MHz) δ = 7.13 (d, *J* = 8.4 Hz, 2H), 7.03 (s, 2H), 6.84 (d, *J* = 8.4 Hz, 2H), 3.91 (s, 12H).[6]

1.1d. Synthesis of 1,2-Bis(2,4-dimethoxyphenyl)ethyne (SI-1e)

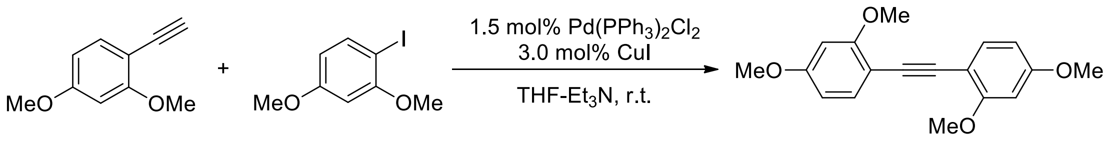

SI-2e SI-3e SI-1e

A 3-necked 250-mL round bottomed flask (pre dried by hot air gun under vacuo) fitted with magnetic stirrer was evacuated/N_2 filled (3 times), then 1-iodo-2,4-dimethoxybenzene[7] (**SI-3e**, 13.20 g, 50 mmol, 1.0 equiv.), $Pd(PPh_3)_2Cl_2$ (526.4 mg, 0.75 mmol, 1.5 mol%), CuI (285.7 mg, 1.5 mmol, 3.0 mol%), THF (40 mL) and Et_3N (100 mL) were added, a solution of 1-ethynyl-2,4-dimethoxybenzene[8] (**SI-2e**, 8.92 g, 55 mmol, 1.1 equiv.) in THF (20 mL) was added dropwise by syringe over 75 min at room temperature. After the reaction was complete as monitored by TLC, the precipitate was collected by filtration, then washed with ether and water, use ethyl acetate (450 mL) to dissolve the crude product, add activated carbon (~ 1.0 g), reflux for 45 min, the precipitate was filtered through a pad of celite when the solution was hot, the filtrate was concentrated to 150 mL, then place the solution for several hours, after filtration and washed with ether, 1,2-bis(2,4-dimethoxyphenyl)ethyne was got as plate crystal (12.44 g, 83%). m.p. 158–160 ^{o}C. **IR (KBr):** 3083, 3002, 2937, 2836, 1864, 1612, 1571, 1514, 1471, 1444, 1417, 1319, 1301, 1281, 1244, 1212, 1166, 1127, 1046, 1030, 927, 826, 786, 720, 633, 604, 560, 497 cm^{-1}; **^{1}H NMR** ($CDCl_3$, 300 MHz) δ = 7.42 (d, J = 8.1 Hz, 2H), 6.48–6.45 (m, 4H), 3.89 (s, 6H), 3.82 (s, 6H); **^{13}C NMR** ($CDCl_3$, 100 MHz) δ = 160.9, 160.8, 134.0, 105.7, 104.7, 98.4, 88.2, 55.8, 55.3; **MS** (**EI**, *m/z*, %): 298 (M^+, 100.00); **HRMS** (**EI**): Calcd. For $C_{18}H_{18}O_4$: 298.1205; Found: 298.1207.

1.1e. Synthesis of 1,2-Bis(4-methoxy-3,5-dimethylphenyl)ethyne (SI-1f)

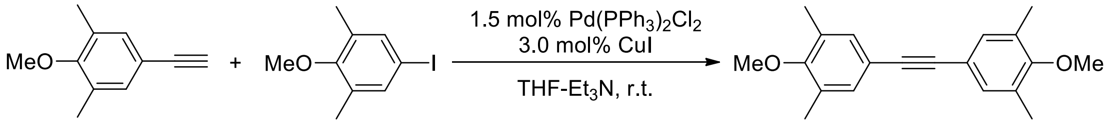

SI-2f SI-3f SI-1f

A 3-necked 250-mL round bottomed flask (pre dried by hot air gun under vacuo)

fitted with magnetic stirrer was evacuated/N_2 filled (3 times), then 5-iodo-2-methoxy-1,3-dimethylbenzene[9] (**SI-3f**, 10.47 g, 39.9 mmol, 1.0 equiv.), $Pd(PPh_3)_2Cl_2$ (526.4 mg, 0.75 mmol, 1.9 mol%), CuI (285.7 mg, 1.5 mmol, 3.8 mol%), THF (40 mL) and Et_3N (100 mL) were added, a solution of 5-ethynyl-2-methoxy-1,3-dimethylbenzene[10] (**SI-2f**, 7.04 g, 43.9 mmol, 1.1 equiv.) in THF (20 mL) was added dropwise by syringe over 75 min at room temperature. After the reaction was complete as monitored by TLC, evaporate the solvent, CH_2Cl_2 (350 mL) was added to dissolve the product, the precipitate was removed by filtration through a pad of celite, the filtrate was washed with water (200 mL×3), dried over $MgSO_4$. After remove the solvent, use ethyl acetate (200 mL) to dissolve the crude product, add activated carbon (~ 1.0 g), reflux for 1 h, the precipitate was filtered through a pad of celite when the solution was hot, the filtrate was concentrated to 20 mL, then place the solution for several hours, after filtration and washed with ether, the crude product was given as hoar solid, further recrystallized from anhydrous ethyl alcohol, 1,2-bis(4-methoxy-3,5-dimethylphenyl)ethyne (**SI-1f**) was got as acicular crystal (10.17 g, yield 86%). m.p. 145–147 ^oC. **IR (KBr):** 2946, 2857, 1755, 1484, 1420, 1352, 1229, 1159, 1038, 1002, 881, 766, 617, 493 cm^{-1}; **^{1}H NMR** ($CDCl_3$, 300 MHz) δ = 7.18 (s, 4H), 3.73 (s, 6H), 2.28 (s, 12H); **^{13}C NMR** ($CDCl_3$, 100 MHz) δ = 157.1, 132.0, 131.0, 118.7, 88.2, 59.7, 15.9; **MS** (**EI**, *m/z*, %): 294 (M^+, 85.23), 279 (100.00); **HRMS** (**EI**): Calcd. For $C_{20}H_{22}O_2$: 294.1620; Found: 294.1623.

1.1f. Synthesis of 1,2-Di(naphthalen-1-yl)ethyne (SI-1g)

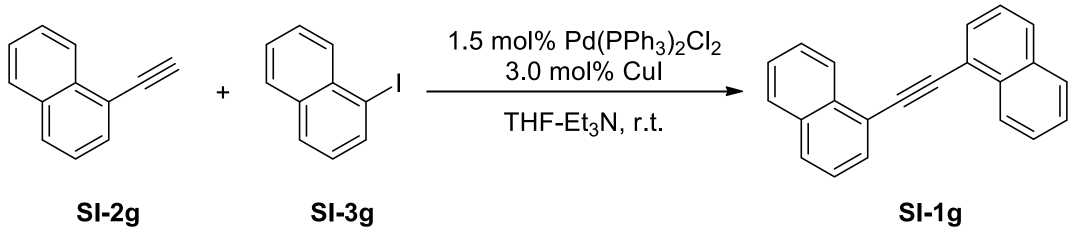

A 3-necked 250-mL round bottomed flask (pre dried by hot air gun under vacuo) fitted with magnetic stirrer was evacuated/N_2 filled (3 times), then 1-iodonaphthalene (**SI-3g**, 8.32 g, 32.7 mmol, 1.0 equiv.), $Pd(PPh_3)_2Cl_2$ (343.9 mg, 0.49 mmol, 1.5

mol%), CuI (186.6 mg, 0.98 mmol, 3.0 mol%), THF (30 mL) and Et_3N (70 mL) were added, a solution of 1-ethynylnaphthalene (**SI-2g**, 5.48 g, 36 mmol, 1.1 equiv.) in THF (10 mL) was added dropwise by syringe over 75 min at room temperature. After the reaction was complete as monitored by TLC, evaporate the solvent, CH_2Cl_2 (250 mL) was added to dissolve the product, the precipitate was removed by filtration through a pad of celite, the filtrate was washed with water (150 mL×3), dried over $MgSO_4$. After remove the solvent, purification by silica-gel chromatography (eluant: petroleum ether/ethyl acetate = 10/1) and further recrystallized from anhydrous ethyl alcohol, 1,2-di(naphthalen-1-yl)ethyne (**SI-1g**) was got as yellow plate crystal (6.75 g, yield 74%). **^{1}H NMR** ($CDCl_3$, 400 MHz) δ = 8.57 (d, *J* = 8.4 Hz, 2H), 7.92–7.88 (m, 6H), 7.66–7.62 (m, 2H), 7.59–7.55 (m, 2H), 7.52 (t, *J* = 7.6 Hz, 2H).[11]

1.1g. Synthesis of 1,2-Di(naphthalen-2-yl)ethyne (SI-1h)

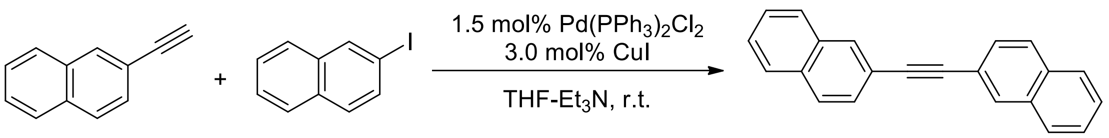

A 3-necked 250-mL round bottomed flask (pre dried by hot air gun under vacuo) fitted with magnetic stirrer was evacuated/N_2 filled (3 times), then 2-iodonaphthalene (**SI-3h**, 11.43 g, 45 mmol, 1.0 equiv.), $Pd(PPh_3)_2Cl_2$ (473.8 mg, 0.675 mmol, 1.5 mol%), CuI (257.1 mg, 1.35 mmol, 3.0 mol%), THF (35 mL) and Et_3N (90 mL) were added, a solution of 2-ethynylnaphthalene (**SI-2h**, 7.53 g, 49.5 mmol, 1.1 equiv.) in THF (15 mL) was added dropwise by syringe over 75 min at room temperature. After the reaction was complete as monitored by TLC, the precipitate was collected by filtration, then washed with water, use toluene (400 mL) to dissolve the crude product, add activated carbon (~ 1.0 g), reflux for 45 min, the precipitate was filtered through a pad of celite when the solution was hot, the filtrate was concentrated and recrystallized from toluene for several times, 1,2-di(naphthalen-2-yl)ethyne (**SI-1h**) was got as white plate crystal (11.15 g, yield 89%). **^{1}H NMR** ($CDCl_3$, 400 MHz) δ =

8.11 (s, 2H), 7.86–7.83 (m, 6H), 7.63 (dd, J = 8.5, 1.5 Hz, 2H), 7.52–7.50 (m, 4H).[11]

1.1h. Synthesis of 1,2-Bis(4-methoxynaphthalen-1-yl)ethyne (SI-1i)

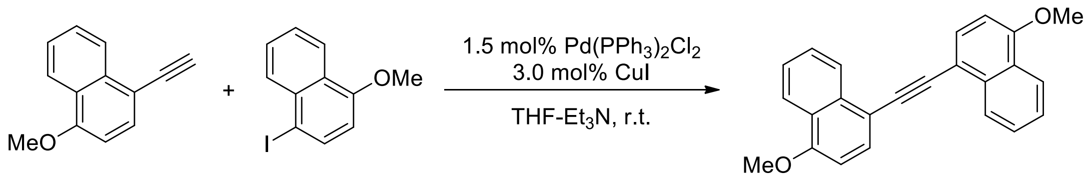

A 3-necked 1.0-L round bottomed flask (pre dried by hot air gun under vacuo) fitted with magnetic stirrer was evacuated/N_2 filled (3 times), then 1-iodo-4-methoxynaphthalene[12] (67.04 g, 236 mmol, 1.0 equiv.), $Pd(PPh_3)_2Cl_2$ (2.485 g, 3.54 mmol, 1.5 mol%), CuI (1.348 g, 7.08 mmol, 3.0 mol%), THF (180 mL) and Et_3N (470 mL) were added, a solution of 1-ethynyl-4-methoxynaphthalene[13] (47.41 g, 260 mmol, 1.1 equiv.) in THF (80 mL) was added dropwise by syringe over 75 min at room temperature. After the reaction was complete as monitored by TLC, the precipitate was collected by filtration, then washed with ether, recrystallized from toluene for several times, 1,2-bis(4-methoxynaphthalen-1-yl)ethyne was got as white acicular crystal (64.34 g, yield 81%). m.p. 201–203 ^{o}C. **IR (KBr):** 3050, 3010, 2962, 2936, 2836, 1579, 1510, 1461, 1421, 1393, 1373, 1326, 1300, 1255, 1240, 1098, 1063, 1023, 1006, 831, 814, 767, 712, 608 cm^{-1}; **^{1}H NMR** ($CDCl_3$, 300 MHz) δ = 8.52 (d, J = 8.4 Hz, 2H), 8.32 (d, J = 8.1 Hz, 2H), 7.80 (d, J = 7.8 Hz, 2H), 7.65 (t, J = 7.2 Hz, 2H), 7.55 (t, J = 7.4 Hz, 2H), 6.85 (d, J = 8.1 Hz, 2H), 4.06 (s, 6H); **^{13}C NMR** ($CDCl_3$, 100 MHz) δ = 155.8, 134.2, 130.8, 127.3, 126.2, 125.7, 125.4, 122.3, 113.6, 103.7, 91.0, 55.6; **MS** (**EI**, *m/z*, %): 338 (M^+, 100.00), 323 (82.96); **HRMS** (**EI**): Calcd. For $C_{24}H_{18}O_2$: 338.1307; Found: 338.1304.

1.2. Preparation of $TMSCF_2Cl$

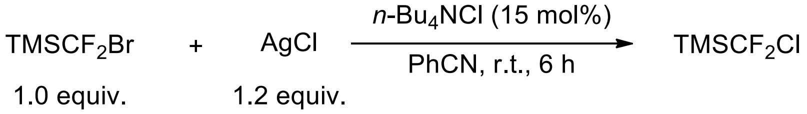

(Chlorodifluoromethyl)trimethylsilane ($TMSCF_2Cl$) was prepared according to a reported method.[14]

To a 150-mL oven-dried round bottomed flask, AgCl (13.76 g, 96 mmol, 1.2 equiv.), anhydrous *n*-Bu_4NCl (3.335 g, 12 mmol, 0.15 equiv.), PhCN (40 mL) and $TMSCF_2Br$ (16.25 g, 80 mmol, 1.0 equiv.) were successively added. After the reaction mixture was stirred at room temperature for 6 h, the desired product and other low boiling point substances were evaporated and condensed into a cold trap at –196 ^{o}C. The mixture in the cold trap was collected, then subjected to fractional distillation using a 15-cm column packed with glass helices to afford pure $TMSCF_2Cl$ (collected at 80~82 ^{o}C) as colorless liquid, yield: 10.46 g (82% based on $TMSCF_2Br$). **^{1}H NMR** ($CDCl_3$, 400 MHz) δ = 0.30 (s, 9H); **^{19}F NMR** ($CDCl_3$, 376 MHz) δ = –62.9 (s, 2F). The characterization data are consistent with previous report.[15]

1.3. Synthesis of CpFluors 1 via [2+1] Cycloaddition Reaction

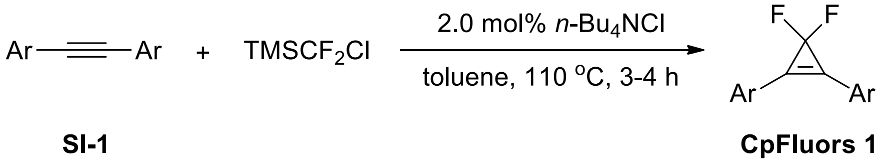

1.3a. Synthesis of (3,3-Difluorocycloprop-1-ene-1,2-diyl)dibenzene (1a)

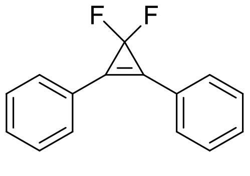

A 250-mL sealed round bottomed flask (pre dried by hot air gun under vacuo) fitted with magnetic stirrer was evacuated/N_2 filled (3 times), then 1,2-diphenylethyne (3.565 g, 20 mmol, 1.0 equiv.), *n*-Bu_4NCl (111.2 mg, 0.4 mmol, 2%), anhydrous toluene (80 mL) and $TMSCF_2Cl$ (4.760 g, 30 mmol, 1.5 equiv.) were successively added, then put the sealed flask into 110 ^{o}C oil bath, the mixture was stirred for 4 h, after cooled to room temperature, remove toluene and volatile materials under

reduced pressure, the residue was dissolved by a mixture solvent of Et_2O (60 mL) and Et_3N (3 mL), then washed with 10% Na_2CO_3 (20 mL × 1), the aqueous phase was extracted with Et_2O (20 mL × 1), the combined organic layer was washed with 2% Na_2CO_3 (40 mL × 1), dried over anhydrous K_2CO_3. After remove the solvent and recrystallized from mixture solvent of hexane (10 mL) and Et_3N (1.0 mL), (3,3-difluorocycloprop-1-ene-1,2-diyl)dibenzene was got as white solid (3.962 g, 87%). ^{1}**H NMR** ($CDCl_3$, 300 MHz) δ = 7.82–7.79 (m, 4H), 7.55–7.52 (m, 6H). ^{19}**F NMR** ($CDCl_3$, 282 MHz) δ = −112.5 (s, 2F); **MS** (**EI**, *m/z*, %): 228 (M^+, 64.61), 178 (100.00).[15]

1.3b. Synthesis of 4,4'-(3,3-Difluorocycloprop-1-ene-1,2-diyl)bis(methoxybenzene) (1c)

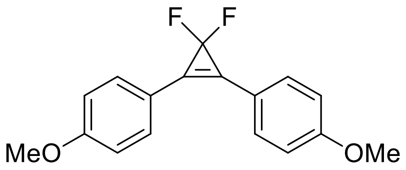

A 100-mL sealed round bottomed flask (pre dried by hot air gun under vacuo) fitted with magnetic stirrer was evacuated/N_2 filled (3 times), then 1,2-bis(4-methoxyphenyl)ethyne (1.906 g, 8.0 mmol, 1.0 equiv.), *n*-Bu_4NCl (44.5 mg, 0.16mol, 2%), anhydrous toluene (30 mL) and $TMSCF_2Cl$ (1.904 g, 12.0 mmol, 1.5 equiv.) were successively added, then put the sealed flask into 110 ^{o}C oil bath, the mixture was stirred for 4 h, after cooled to room temperature, remove toluene and volatile materials under reduced pressure, the residue was dissolved by a mixture solvent of Et_2O (70 mL) and Et_3N (10 mL), then washed with 10% Na_2CO_3 (60 mL × 3), dried over anhydrous K_2CO_3, and concentrated to dryness, the residue was washed with little mixture solvent of Et_2O/Et_3N (10/1), 4,4'-(3,3-difluorocycloprop-1-ene-1,2-diyl)bis(methoxybenzene) was got as white solid (2.082 g, 90%). m.p. 112−114 ^{o}C. **IR (KBr):** 2969, 2842, 1847, 1783, 1601, 1571, 1519, 1504, 1421, 1376, 1304, 1251, 1170, 1030, 962, 825, 622, 565, 544, 512, 501 cm^{-1}; ^{1}**H NMR** ($CDCl_3$, 400 MHz) δ = 7.69 (d, *J* = 8.0 Hz, 4H), 7.01 (d, *J* = 8.8 Hz, 4H), 3.86 (s, 6H); ^{19}**F NMR** ($CDCl_3$, 376

MHz) δ = –112.0 (s, 2F); **^{13}C NMR** ($CDCl_3$, 100 MHz) δ = 161.4, 131.8, 119.9 (t, *J* = 10.6 Hz), 117.3, 114.6, 102.6 (t, *J* = 268.7 Hz), 55.4; **MS** (**EI**, *m/z*, %): 288 (M^+, 100.00), 257 (82.22); **HRMS** (**EI**): Calcd. For $C_{17}H_{14}O_2F_2$: 288.0962; Found: 288.0966.

1.3c. General Procedures for the Synthesis of 3,3-Difluorocycloprop-1- enes 1b, 1d, 1e, 1f, 1g and 1h.

A 250-mL sealed round bottomed flask (pre dried by hot air gun under vacuo) fitted with magnetic stirrer was evacuated/N_2 filled (3 times), then 1,2-diarylethyne (20 mmol, 1.0 equiv.), *n*-Bu_4NCl (111.2 mg, 0.4 mmol, 2%), anhydrous toluene (80 mL) and $TMSCF_2Cl$ (4.760 g, 30 mmol, 1.5 equiv.) were successively added, then put the sealed flask into 110 ^{o}C oil bath, the mixture was stirred for 3-4 h, after cooled to room temperature, remove toluene and volatile materials under reduced pressure, the residue was recrystallized from appropriate solvent, 1,2-diaryl-3,3-difluorocycloprop-1-enes was got as solid.

2,2'-(3,3-difluorocycloprop-1-ene-1,2-diyl)bis(methoxybenzene) (1b)

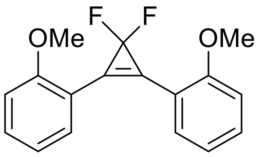

After recrystallized from anhydrous toluene, **1b** was got as white solid (5.26 g, 91%). m.p. 125–127 ^{o}C. **IR (KBr):** 3010, 2967, 2937, 2837, 1765, 1597, 1494, 1485, 1466, 1438, 1361, 1319, 1272, 1241, 1183, 1168, 1161, 1040, 1027, 1001, 979, 835, 762 cm^{-1}; **^{1}H NMR** ($CDCl_3$, 400 MHz) δ = 7.83 (dd, *J* = 7.6, 1.6 Hz, 2H), 7.46–7.42 (m, 2H), 7.07 (td, *J* = 7.6, 0.8 Hz, 2H), 7.00 (d, *J* = 8.4 Hz, 2H), 3.98 (s, 6H); **^{19}F NMR** ($CDCl_3$, 376 MHz) δ = –107.9 (s, 2F); **^{13}C NMR** ($CDCl_3$, 100 MHz) δ = 158.6, 132.4, 132.3, 120.7, 118.3 (t, *J* = 10.6 Hz), 114.6, 111.1, 101.8 (t, *J* = 267.4 Hz), 55.6; **MS** (**EI**, *m/z*, %): 288 (M^+, 5.12), 273 (100.00); **HRMS** (**EI**): Calcd. For $C_{17}H_{14}O_2F_2$: 288.0962; Found: 288.0964; **Elem. Anal.**: Calcd. For $C_{17}H_{14}O_2F_2$: C, 70.83; H, 4.89;

F, 13.18; Found: C, 70.86; H, 4.94; F, 13.24.

4,4'-(3,3-difluorocycloprop-1-ene-1,2-diyl)bis(1,2-dimethoxybenzene) (1d)

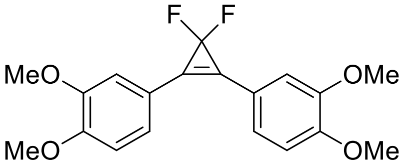

After washed with mixed solvent of DCM/Et_3N (2/1), **1d** was got as white solid (6.86 g, 98%). m.p. 218–220 ^oC. **IR (KBr):** 3016, 2964, 2942, 2842, 1604, 1585, 1516, 1452, 1362, 1334, 1296, 1274, 1251, 1227, 1142, 1023, 970, 841, 811, 794, 764 cm^{-1}; **^{1}H NMR** ($CDCl_3$, 400 MHz) δ = 7.36 (dd, *J* = 8.4, 2.0 Hz, 2H), 7.18 (d, *J* = 1.6 Hz, 2H), 6.98 (d, *J* = 8.0 Hz, 2H), 3.96 (s, 6H), 3.95 (s, 6H); **^{19}F NMR** ($CDCl_3$, 376 MHz) δ = –111.6 (s, 2F); **^{13}C NMR** ($CDCl_3$, 100 MHz) δ = 151.2, 149.3, 123.8, 120.5 (t, *J* = 10.8 Hz), 117.5, 112.5, 111.4, 102.6 (t, *J* = 268.6 Hz), 56.00, 55.98; **MS** (**EI**, *m/z*, %): 348 (M^+, 100.00), 317 (98.39); **HRMS** (**EI**): Calcd. For $C_{19}H_{18}O_4F_2$: 348.1173; Found: 348.1172.

4,4'-(3,3-difluorocycloprop-1-ene-1,2-diyl)bis(1,3-dimethoxybenzene) (1e)

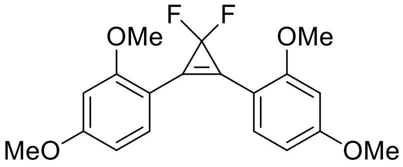

After recrystallized from anhydrous toluene, **1e** was got as white solid (6.27 g, 90%). m.p. 145-147 ^oC. **IR (KBr):** 2982, 2952, 2839, 1610, 1574, 1509, 1471, 1358, 1312, 1286, 1241, 1210, 1167, 1137, 1024, 981, 827, 818 cm^{-1}; **^{1}H NMR** ($CDCl_3$, 400 MHz) δ = 7.71 (d, *J* = 8.4 Hz, 2H), 6.58 (dd, *J* = 8.4, 2.4 Hz, 2H), 6.51 (d, *J* = 2.4 Hz, 2H), 3.94 (s, 6H), 3.85 (s, 6H); **^{19}F NMR** ($CDCl_3$, 376 MHz) δ = –107.8 (s, 2F); **^{13}C NMR** ($CDCl_3$, 100 MHz) δ = 163.0, 159.9, 133.2, 115.1 (t, *J* = 10.3 Hz), 108.0, 105.3, 102.2 (t, *J* = 266.9 Hz), 98.3, 55.6, 55.5; **MS** (**EI**, *m/z*, %): 348 (M^+, 14.27), 149 (100.00); **HRMS** (**EI**): Calcd. For $C_{19}H_{18}O_4F_2$: 348.1173; Found: 348.1171.

5,5'-(3,3-difluorocycloprop-1-ene-1,2-diyl)bis(2-methoxy-1,3-dimethylbenzene) (1f)

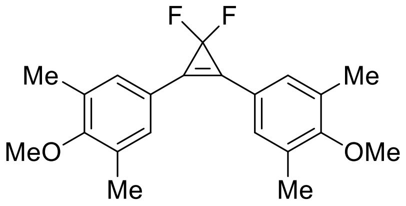

After recrystallized from mixture solvent of petroleum ether/Et_3N (10/1), **1f** was got as colorless solid (6.36 g, 92%). m.p. 143–145 °C. **IR (KBr):** 2926, 2827, 1766, 1602, 1488, 1454, 1385, 1374, 1288, 1266, 1250, 1212, 1169, 1117, 1100, 1016, 996, 975, 926, 874, 796, 760 cm^{-1}; **^{1}H NMR** ($CDCl_3$, 400 MHz) δ = 7.43 (s, 4H), 3.78 (s, 6H), 2.38 (s, 12H); **^{19}F NMR** ($CDCl_3$, 376 MHz) δ = –112.3 (s, 2F); **^{13}C NMR** ($CDCl_3$, 100 MHz) δ = 159.2, 132.0, 130.9, 121.2 (t, *J* = 10.6 Hz), 120.2, 102.6 (t, *J* = 268.8 Hz), 59.7, 16.1; **MS** (**EI**, *m/z*, %): 344 (M^+, 75.32), 329 (100.00); **HRMS** (**EI**): Calcd. For $C_{21}H_{22}O_2F_2$: 344.1588; Found: 344.1585; **Elem. Anal.**: Calcd. For $C_{21}H_{22}O_2F_2$: C, 73.24; H, 6.44; F, 11.03; Found: C, 73.26; H, 6.48; F, 10.96.

1,1'-(3,3-difluorocycloprop-1-ene-1,2-diyl)dinaphthalene (1g)

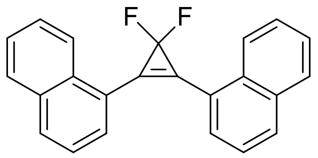

After recrystallized from anhydrous hexane, **1g** was got as yellowgreen solid (5.34 g, 81%). m.p. 121–123 °C. **IR (KBr):** 3050, 1755, 1509, 1351, 1272, 1211, 988, 972, 859, 848, 796, 771 cm^{-1}; **^{1}H NMR** ($CDCl_3$, 400 MHz) δ = 8.43 (d, *J* = 8.4 Hz, 2H), 8.12 (dd, *J* = 7.2, 0.8 Hz, 2H), 8.03 (d, *J* = 8.4 Hz, 2H), 7.96 (d, *J* = 8.4 Hz, 2H), 7.74–7.70 (m, 2H), 7.63 (t, *J* = 7.8 Hz, 4H); **^{19}F NMR** ($CDCl_3$, 376 MHz) δ = –101.1 (s, 2F); **^{13}C NMR** ($CDCl_3$, 100 MHz) δ = 133.7, 131.8, 131.4, 130.0, 128.6, 127.9, 126.8, 125.4, 125.3, 122.5, 121.9 (t, *J* = 10.9 Hz), 102.3 (t, *J* = 271.6 Hz); **MS** (**EI**, *m/z*, %): 328 (M^+, 32.58), 307 (100.00); **HRMS** (**EI**): Calcd. For $C_{23}H_{14}F_2$: 328.1064; Found: 328.1067.

2,2'-(3,3-difluorocycloprop-1-ene-1,2-diyl)dinaphthalene (1h)

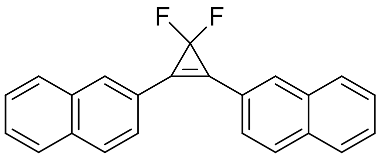

After recrystallized from anhydrous toluene, **1h** was got as light brown solid (5.72 g, 87%). m.p. 156–157 ^{o}C. **IR (KBr):** 3059, 1772, 1352, 1287, 1266, 1021, 1010, 982, 950, 905, 869, 858, 827, 818, 762, 749 cm^{-1}; ^{1}**H NMR** ($CDCl_3$, 400 MHz) δ = 8.29 (s, 2H), 7.98−7.95 (m, 4H), 7.90−7.87 (m, 4H), 7.61−7.56 (m, 4H); ^{19}**F NMR** ($CDCl_3$, 376 MHz) δ = −112.2 (s, 2F); ^{13}**C NMR** ($CDCl_3$, 100 MHz) δ = 134.2, 133.1, 131.0, 129.0, 128.7, 127.9, 127.8, 127.0, 126.4, 123.2 (t, *J* = 10.3 Hz), 121.9, 102.5 (t, *J* = 269.9 Hz); **MS** (**EI**, *m/z*, %): 328 (M^{+}, 100.00), 278 (69.55), 276 (54.29); **HRMS** (**EI**): Calcd. For $C_{23}H_{14}F_2$: 328.1064; Found: 328.1059.

1.3d. Synthesis of 4,4'-(3,3-Difluorocycloprop-1-ene-1,2-diyl)bis(1-methoxynaphthalene) (1i)

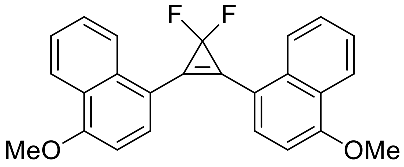

A 150-mL sealed round bottomed flask (pre dried by hot air gun under vacuo) fitted with magnetic stirrer was evacuated/N_2 filled (3 times), then 1,2-bis(4-methoxynaphthalen-1-yl)ethyne (3.384 g, 10 mmol, 1.0 equiv.), *n*-Bu_4NCl (55.6 mg, 0.2 mmol, 2%), anhydrous toluene (40 mL) and $TMSCF_2Cl$ (2.380 g, 15 mmol, 1.5 equiv.) were successively added, then put the sealed flask into 110 ^{o}C oil bath, the mixture was stirred for 4 h, after cooled to room temperature, remove toluene and volatile materials under reduced pressure, anhydrous KF (581 mg) and CH_3CN (40 mL) were added under dry N_2 atmosphere, the mixture was further stirred at 70 ^{o}C for 24 h, after cooled to room temperature, the precipitate was collected by filtration, washed with anhydrous CH_3CN, after recrystallized (the insoluble matter was removed by hot filtration) from anhydrous mixture solvent of toluene (40 mL) and Et_3N (2 mL), **1i** was got as bright yellow solid (3.21 g, 83%). m.p. 210–212 ^{o}C.

IR (KBr): 3004, 2960, 2937, 2903, 2835, 1578, 1514, 1464, 1440, 1359, 1261, 1244, 1209, 1098, 1089, 1006, 960, 947, 817, 804, 760, 599 cm^{-1}; **^{1}H NMR** ($CDCl_3$, 400 MHz) δ = 8.38–8.35 (m, 4H), 8.03 (d, *J* = 8.0 Hz, 2H), 7.73–7.69 (m, 2H), 7.62–7.58 (m, 2H), 6.95 (d, *J* = 8.4 Hz, 2H) 4.09 (s, 6H); **^{19}F NMR** ($CDCl_3$, 376 MHz) δ = −100.9 (s, 2F); **^{13}C NMR** ($CDCl_3$, 100 MHz) δ = 158.0, 132.5, 130.9, 128.2, 126.0, 125.7, 125.2, 122.5, 119.0 (t, *J* = 10.8 Hz), 115.4, 103.8, 102.8 (t, *J* = 269.9 Hz), 55.8; **MS** (**EI**, *m/z*, %): 388 (M^+, 100.00), 337 (85.49), 387 (83.58); **HRMS** (**EI**): Calcd. For $C_{25}H_{18}O_2F_2$: 388.1275; Found: 388.1278.

2. Optimization on the Structure of CpFluors 1 for Deoxyfluorination of 3-Phenylpropan-1-ol (2a).

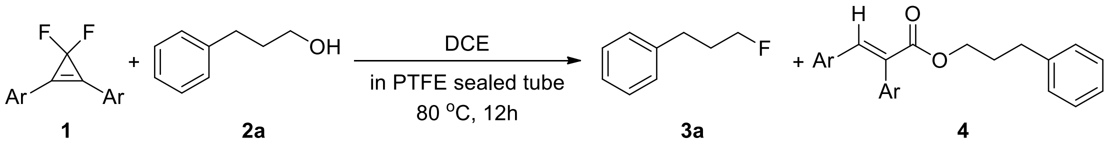

General Procedures:

To a dry polytetrafluoroethene (PTFE) sealed tube, 3-phenylpropan-1-ol (**2a**, 68.1 mg, 0.5 mmol), reagent **1** (0.6 mmol) and solvent DCE (3.5 mL) were sequentially added. The sealed tube was immersed in an oil bath at 80 °C, and the mixture was stirred for 12 h, after cooled to room temperature (yield of **3a** was determined by ^{19}F NMR using $PhCF_3$ as an internal standard and was given in the Main Text, Figure 2b), the mixture was concentrated under reduced pressure and the residue was purified by flash chromatography on silica gel to give the corresponding ester product **4**.

(*E*)-3-Phenylpropyl 2,3-diphenylacrylate (4a)

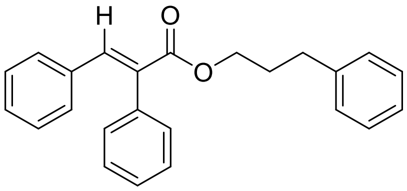

Performed on 0.5 mmol scale, eluted with hexane/Et_2O = 8/1 to give **4a** (68.4 mg, 40% yield). Colorless oil. **IR (film):** 3059, 3026, 2954, 1709, 1601, 1496, 1448, 1244, 1207, 1171, 1021, 776, 750, 710, 697 cm^{-1}; ^{1}**H NMR** ($CDCl_3$, 400 MHz) δ = 7.83 (s, 1H), 7.40–7.35 (m, 3H), 7.27–7.22 (m, 4H), 7.20–7.10 (m, 6H), 7.06 (d, *J* = 7.1 Hz, 2H), 4.21 (t, *J* = 6.4 Hz, 2H), 2.62 (t, *J* = 7.4 Hz, 2H), 1.99–1.92 (m, 2H); ^{13}**C NMR** ($CDCl_3$, 100 MHz) δ = 167.6, 141.1, 140.1, 136.0, 134.5, 132.7, 130.5, 129.6, 128.9, 128.5, 128.33, 128.32, 128.1, 127.7, 125.9, 64.2, 32.0, 30.2; **MS** (**EI**, *m/z*, %): 342 (M^+, 4.60), 224 (100.00); **HRMS** (**EI**): Calcd. For $C_{24}H_{22}O_2$: 342.1620; Found: 342.1623.

(*E*)-3-Phenylpropyl 2,3-bis(2-methoxyphenyl)acrylate (4b)

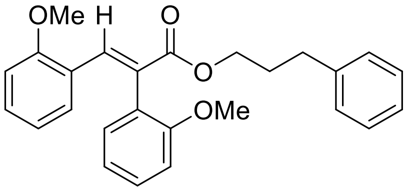

Performed on 0.5 mmol scale, eluted with PE/EA = 4/1 to give **4b** (77.9 mg, 39% yield). Colorless oil. **IR (film):** 3026, 2954, 2837, 1709, 1597, 1493, 1484, 1463, 1436, 1293, 1250, 1162, 1113, 1051, 1028, 753, 701 cm^{-1}; ^{1}**H NMR** ($CDCl_3$, 400 MHz) δ = 8.14 (s, 1H), 7.30–7.23 (m, 3H), 7.17–7.09 (m, 4H), 7.00 (dd, *J* = 7.5, 1.7 Hz, 1H), 6.91 (d, *J* = 7.7 Hz, 1H), 6.87–6.81 (m, 2H), 6.78 (dd, *J* = 7.7, 1.6 Hz, 1H), 6.60–6.56 (m, 1H), 4.18 (t, *J* = 6.4 Hz, 2H), 3.82 (s, 3H), 3.73 (s, 3H), 2.60 (t, *J* = 7.6 Hz, 2H), 1.94–1.90 (m, 2H); ^{13}**C NMR** ($CDCl_3$, 100 MHz) δ = 168.0, 158.0, 157.6, 141.4, 135.1, 130.9, 130.0, 129.94, 129.91, 129.1, 128.4, 128.3, 125.8, 125.7, 124.1, 120.7, 119.9, 110.7, 110.4, 63.7, 55.4, 31.9, 30.3; **MS** (**EI**, *m/z*, %): 402 (M^+, 22.27), 91 (100.00); **HRMS** (**EI**): Calcd. For $C_{26}H_{26}O_4$: 402.1831; Found: 402.1832.

(*E*)-3-Phenylpropyl 2,3-bis(4-methoxyphenyl)acrylate (4c)

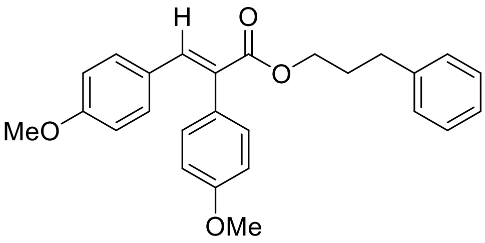

Performed on 0.5 mmol scale, eluted with PE/EA = 6/1 to give **4c** (45.3 mg, 23% yield). Colorless oil. **IR (film):** 3026, 2955, 2837, 1705, 1604, 1509, 1289, 1250, 1167, 1030, 834, 701 cm^{-1}; ^{1}**H NMR** ($CDCl_3$, 400 MHz) δ = 7.76 (s, 1H), 7.27 (t, *J* = 7.4 Hz, 2H), 7.19–7.12 (m, 5H), 7.04 (d, *J* = 8.4 Hz, 2H), 6.93 (d, *J* = 8.8 Hz, 2H), 6.70 (d, *J* = 8.8 Hz, 2H), 4.19 (t, *J* = 6.4 Hz, 2H), 3.84 (s, 3H), 3.75 (s, 3H), 2.63 (t, *J* = 7.7 Hz, 2H), 2.00–1.93 (m, 2H); ^{13}**C NMR** ($CDCl_3$, 100 MHz) δ = 168.2, 160.1, 159.0, 141.3, 139.7, 132.3, 131.0, 129.9, 128.5, 128.40, 128.36, 127.4, 125.9, 114.1, 113.6, 64.1, 55.16, 55.15, 32.1, 30.3; **MS** (**EI**, *m/z*, %): 402 (M^+, 64.28), 284 (100.00); **HRMS** (**EI**): Calcd. For $C_{26}H_{26}O_4$: 402.1831; Found: 402.1837.

(*E*)-3-Phenylpropyl 2,3-bis(3,4-dimethoxyphenyl)acrylate (4d)

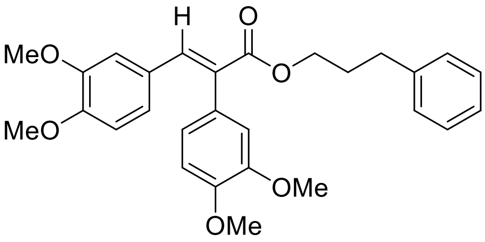

Performed on 0.5 mmol scale, eluted with hexane/EA = 4/1 to give **4d** (50.9 mg, 22% yield). Light yellow solid. M.p. 81–83 °C. **IR (KBr):** 3002, 2958, 2939, 2839, 1702, 1620, 1597, 1582, 1519, 1466, 1418, 1335, 1316, 1252, 1231, 1197, 1186, 1139, 1022, 864, 823, 759, 701 cm^{-1}; ^{1}**H NMR** ($CDCl_3$, 400 MHz) δ = 7.75 (s, 1H), 7.29–7.26 (m, 2H), 7.20–7.13 (m, 3H), 6.92 (d, *J* = 8.0 Hz, 1H), 6.84–6.80 (m, 3H), 6.73 (d, *J* = 8.0 Hz, 1H), 6.56 (d, *J* = 2.0 Hz, 1H), 4.22 (t, *J* = 6.5 Hz, 2H), 3.90 (s, 3H), 3.85 (s, 3H), 3.82 (s, 3H), 3.49 (s, 3H), 2.66 (t, *J* = 7.6 Hz, 2H), 2.02–1.95 (m, 2H); ^{13}**C NMR** ($CDCl_3$, 100 MHz) δ = 168.0, 149.9, 149.2, 148.5, 148.1, 141.1, 140.0, 129.9, 128.9, 128.3, 127.4, 125.9, 125.2, 122.2, 112.9, 112.4, 111.4, 110.4, 64.1, 55.9, 55.7, 55.2, 32.0, 30.2; **MS** (**EI**, *m/z*, %): 462 (M^+, 57.33), 91 (100.00); **HRMS** (**EI**): Calcd. For

$C_{28}H_{30}O_6$: 462.2042; Found: 462.2038.

(*E*)-3-Phenylpropyl 2,3-bis(2,4-dimethoxyphenyl)acrylate (4e) (contains about 20% *Z*-isomoer)

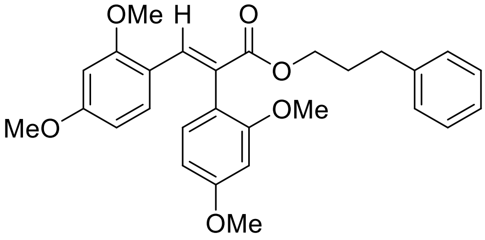

Performed on 0.5 mmol scale, eluted with PE/EA = 3/1 to give **4e** (32.7 mg, 14% yield). Colorless oil. **IR (film):** 3392, 3061, 3001, 2940, 2838, 1704, 1605, 1578, 1498, 1464, 1438, 1418, 1308, 1261, 1236, 1209, 1159, 1123, 1031, 936, 833, 803, 749, 701 cm^{-1}; ^{1}**H NMR** ($CDCl_3$, 400 MHz) δ = 8.09 (s, 1H), 7.28–7.24 (m, 2H), 7.19–7.12 (m, 3H), 6.95 (d, *J* = 8.8 Hz, 1H), 6.78 (d, *J* = 8.8 Hz, 1H), 6.52 (d, *J* = 2.0 Hz, 1H), 6.43 (s, 1H), 6.39 (d, *J* = 2.4 Hz, 1H), 6.18 (dd, *J* = 8.7, 2.3 Hz, 1H), 4.17 (t, *J* = 6.4 Hz, 2H), 3.84 (s, 3H), 3.82 (s, 3H), 3.75 (s, 3H), 3.73 (s, 3H), 2.63 (t, *J* = 7.6 Hz, 2H), 1.97–1.90 (m, 2H); ^{13}**C NMR** ($CDCl_3$, 100 MHz) δ = 168.6, 161.5, 160,6, 159.6, 158.6, 141.5, 134.3, 131.4, 130.8, 128.4, 128.3, 127.1, 125.8, 118.7, 117.2, 104.7, 104.5, 98.8, 97.9, 63.7, 55.5, 55.3, 55.2, 32.0, 30.4; **MS** (**EI**, *m/z*, %): 462 (M^+, 52.67), 91 (100.00); **HRMS** (**EI**): Calcd. For $C_{28}H_{30}O_6$: 462.2042; Found: 462.2047.

(*E*)-3-Phenylpropyl 2,3-bis(4-methoxy-3,5-dimethylphenyl)acrylate (4f)

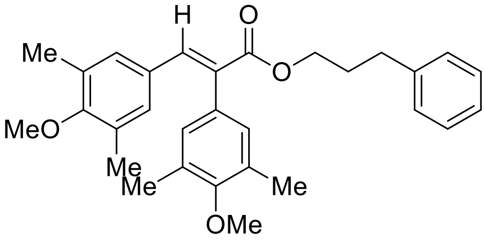

Performed on 0.5 mmol scale, eluted with PE/EA = 6/1 to give **4f** (74.2 mg, 32% yield). Colorless oil. **IR (film):** 3026, 2949, 2826, 1708, 1485, 1455, 1235, 1151, 1136, 1014, 877, 746, 700 cm^{-1}; ^{1}**H NMR** ($CDCl_3$, 400 MHz) δ = 7.66 (s, 1H), 7.26 (t, *J* = 7.4 Hz, 2H), 7.19–7.12 (m, 3H), 6.88 (s, 2H), 6.73 (s, 2H), 4.20 (t, *J* = 6.4 Hz, 2H),

3.75 (s, 3H), 3.66 (s, 3H), 2.66 (t, *J* = 7.6 Hz, 2H), 2.27 (s, 6H), 2.09 (s, 6H), 2.02–1.95 (m, 2H); **^{13}C NMR** ($CDCl_3$, 100 MHz) δ = 168.1, 157.8, 156.7, 141.2, 139.5, 131.65, 131.56, 131.0, 130.9, 130.4, 130.2, 129.9, 128.4, 128.3, 125.9, 64.0, 59.8, 59.5, 32.0, 30.3, 16.00, 15.98; **MS** (**EI**, *m/z*, %): 458 (M^+, 39.84), 91 (100.00); **HRMS** (**EI**): Calcd. For $C_{30}H_{34}O_4$: 458.2457; Found: 458.2453.

(*E*)-3-Phenylpropyl 2,3-di(naphthalen-1-yl)acrylate (4g)

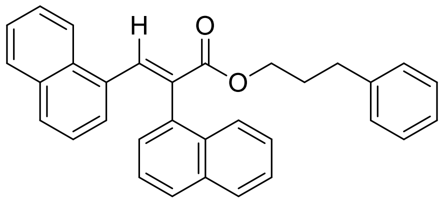

Performed on 0.5 mmol scale, eluted with hexane/Et_2O = 8/1 to give **4g** (68.6 mg, 31% yield). Light brown oil. **IR (film):** 3402, 3059, 2953, 2858, 1709, 1273, 1236, 1210, 1158, 789, 778, 737, 700 cm^{-1}; **^{1}H NMR** ($CDCl_3$, 400 MHz) δ = 8.83 (s, 1H), 8.25 (d, *J* = 8.4 Hz, 1H), 7.91–7.89 (m, 1H), 7.86–7.83 (m, 1H), 7.76 (d, *J* = 8.4 Hz, 2H), 7.57 (t, *J* = 7.6 Hz, 2H), 7.50–7.42 (m, 3H), 7.27 (t, *J* = 7.6 Hz, 1H), 7.20–7.15 (m, 3H), 7.11 (t, *J* = 7.2 Hz, 1H), 6.92–6.84 (m, 4H), 4.20 (t, *J* = 6.0 Hz, 1H), 4.12 (t, *J* = 6.0 Hz, 1H), 2.31 (t, *J* = 7.8 Hz, 2H), 1.82–1.75 (m, 2H); **^{13}C NMR** ($CDCl_3$, 100 MHz) δ = 167.9, 141.0, 139.8, 133.8, 133.7, 133.4, 133.3, 132.6, 132.0, 131.6, 129.0, 128.6, 128.5, 128.3, 128.2, 128.1, 127.5, 127.2, 126.5, 126.3, 125.9. 125.8, 125.5, 125.2, 125.0, 123.9, 64.1, 31.7, 30.1; **MS** (**EI**, *m/z*, %): 442 (M^+, 22.08), 279 (100.00); **HRMS** (**EI**): Calcd. For $C_{32}H_{26}O_2$: 442.1933; Found: 442.1930.

(*E*)-3-Phenylpropyl 2,3-di(naphthalen-2-yl)acrylate (4h)

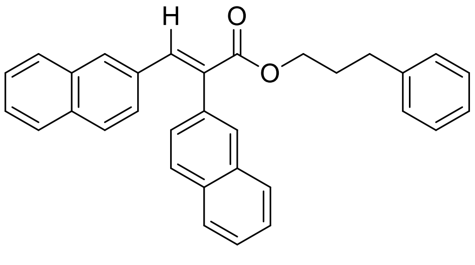

Performed on 0.5 mmol scale, eluted with hexane/Et_2O = 8/1 to give **4h** (86.4 mg, 39% yield). Light yellow solid. M.p. 91–93 ^{o}C. **IR (KBr):** 3055, 3024, 2952, 2886,

2855, 1710, 1616, 1592, 1270, 1233, 1182, 1154, 1120, 1031, 958, 863, 822, 806, 743, 699, 475 cm^{-1}; **^{1}H NMR** ($CDCl_3$, 400 MHz) δ = 8.09 (s, 1H), 7.84 (dd, *J* = 8.2, 3.5 Hz, 2H), 7.78 (s, 1H), 7.75–7.72 (m, 2H), 7.63–7.58 (m, 2H), 7.49–7.41 (m, 2H), 7.40–7.32 (m, 4H), 7.23–7.19 (m, 2H), 7.15–7.12 (m, 1H), 7.06–7.04 (m, 2H), 6.98 (dd, *J* = 8.7, 1.7 Hz, 1H), 4.24 (t, *J* = 6.4 Hz, 2H), 2.66 (t, *J* = 7.6 Hz, 2H), 1.98–1.91 (m, 2H); **^{13}C NMR** ($CDCl_3$, 100 MHz) δ = 167.8, 141.1, 140.5, 133.5, 133.4, 133.3, 132.9, 132.8, 132.6, 132.3, 131.7, 129.0, 128.4, 128.35, 128.33, 128.1, 127.9, 127.7, 127.6, 127.5, 127.0, 126.7, 126.22, 126.18, 126.0, 125.9, 64.4, 32.1, 30.2; **MS** (**EI**, *m/z*, %): 442 (M^+, 24.44), 279 (100.00); **HRMS** (**EI**): Calcd. For $C_{32}H_{26}O_2$: 442.1933; Found: 442.1935.

(*E*)-3-Phenylpropyl 2,3-bis(4-methoxynaphthalen-1-yl)acrylate (4i) (contains about 20% *Z*-isomoer)

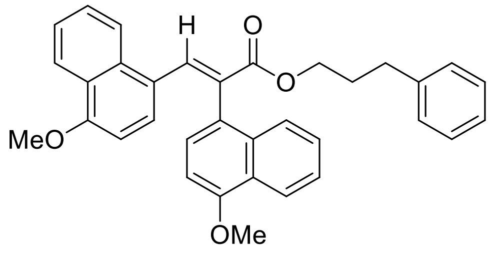

Performed on 0.5 mmol scale, eluted with PE/EA = 4/1 to give **4i** (9.4 mg, 4% yield). Light brown oil. **IR (film):** 3062, 3025, 3003, 2955, 2839, 1707, 1621, 1583, 1512, 1462, 1423, 1377, 1321, 1284, 1267, 1241, 1209, 1159, 1095, 1067, 1044, 1026, 819, 791, 765, 737, 713, 701 cm^{-1}; **^{1}H NMR** ($CDCl_3$, 400 MHz) δ = 8.77 (s, 1H), 8.33–8.31 (m, 1H), 8.24 (d, *J* = 9.6 Hz, 2H), 7.83–7.81 (m, 1H), 7.63–7.59 (m, 1H), 7.54–7.45 (m, 3H), 7.21 (t, *J* = 7.4 Hz, 2H), 7.16–7.11 (m, 2H), 6.93 (d, *J* = 7.2 Hz, 2H), 6.84 (d, *J* = 8.4 Hz, 1H), 6.69 (d, *J* = 8.0 Hz, 1H), 6.30 (d, *J* = 8.4 Hz, 1H), 4.20 (t, *J* = 6.0 Hz, 1H), 4.13 (t, *J* = 6.0 Hz, 1H), 3.97 (s, 3H), 3.81 (s, 3H), 2.38 (t, *J* = 7.6 Hz, 2H), 1.86–1.78 (m, 2H); **^{13}C NMR** ($CDCl_3$, 100 MHz) δ = 168.6, 156.0, 155.1, 141.2, 139.2, 133.5, 133.2, 131.2, 128.4, 128.3, 127.5, 127.1, 126.8, 126.6, 125.8, 125.7, 125.3, 125.24, 125.16, 125.10, 123.9, 123.5, 122.5, 122.3, 103.7, 103.4, 64.0, 55.42, 55.36, 31.9, 30.2; **MS** (**EI**, *m/z*, %): 502 (M^+, 36.67), 91 (100.00); **HRMS** (**EI**):

Calcd. For $C_{34}H_{30}O_4$: 502.2144; Found: 502.2147.

3. Synthesis of Monoalcohols 2 and Diols 6q-6t.

Monoalcohols **2d**,[16] **2g**,[1,17] **2j**,[18,19] **2k**,[18,19] and **2l**[18,19] were synthesized according to the reported procedures. Monoalcohols **2n**, **2p** and **2q**, as well as diols **6q-6t** were synthesized according to the following procedures. The other alcohols **2** and **6a-6p** are commercially available.

3a. Synthesis of (*R*)-4-(3-Hydroxypyrrolidine-1-carbonyl)benzaldehyde (2n)

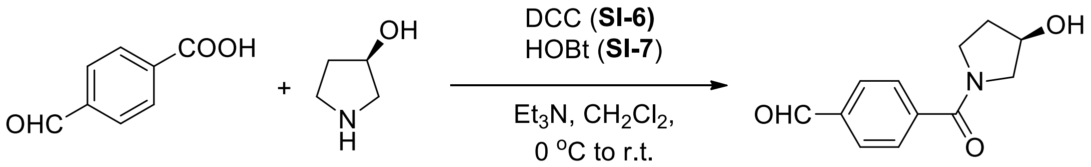

SI-4 **SI-5** **2n**

(*R*)-4-(3-Hydroxypyrrolidine-1-carbonyl)benzaldehyde (**2n**) was prepared according to reference with modifications.[20] 4-formylbenzoic acid (**SI-4**, 1.501 g, 10.0 mmol, 1.0 equiv.) was dissolved in CH_2Cl_2 (15 mL) and dicyclohexylcarbodiimide (**SI-6**, 2.682 g, 13.0 mmol, 1.3 equiv.), 1-hydroxybenzotriazole (**SI-7**, 1.757 g, 13.0 mmol, 1.3 equiv.) and triethylamine (4.2 mL, 30.0 mmol, 3.0 equiv.) were sequentially added. The reaction mixture was cooled at 0 ℃ and a solution of (*R*)-pyrrolidin-3-ol (**SI-5**, 958.3 mg, 11.0 mmol, 1.1 equiv.) in CH_2Cl_2 (10 mL) was added. The reaction mixture was stirred at 0 ℃ for 30 minutes and then warmed to room temperature and stirred for 20 hours. CH_2Cl_2 was then removed under reduced pressure. Water was added and the solution was extracted ten times with EtOAc. The combined organic extracts were dried (Na_2SO_4) and concentrated under reduced pressure to obtain a crude oil that was purified by column chromatography on silica gel (CH_2Cl_2/MeOH 20:1 v/v) to obtain the title compound **2n** as a white solid (807.4 mg, 37%, ee 99.4%). The enantiomeric excess was determined by chiral HPLC, ID (4.6 mm × 150 mm, 3 μm), Hexane/IPA = 60/40 (v/v), 0.7 mL/min, λ = 214 nm, tR

(major) = 11.09 min, tR (minor) = 7.26 min. M.p. 143–145 °C. **IR (KBr):** 3358, 2982, 2937, 2839, 1698, 1612, 1569, 1507, 1448, 1409, 1318, 1297, 1275, 1204, 1100, 998, 974, 868, 856, 835, 754, 711 cm^{-1}; **^{1}H NMR** ($CDCl_3$, 400 MHz) [mixture of 2 rotamers] δ = 10.034 [10.029] (s, 1H), 7.91 [7.90] (d, *J* = 8.4 Hz, 2H), 7.66 [7.62] (d, *J* = 8.4 Hz, 2H), 4.56 [4.44] (brs, 1H), 3.82–3.50 [3.52 (dd, *J* = 11.4, 4.2 Hz)] (m, 3H), 3.43–3.34 (m, 1H), 2.86 [2.79] (d, *J* = 2.8 Hz, 1H), 2.12–1.96 (m, 2H); **^{13}C NMR** ($CDCl_3$, 100 MHz) [mixture of 2 rotamers] δ = 191.58 [191.60], 168.7 [168.8], 142.1 [142.2], 137.03 [136.96], 129.7, 127.7, 69.3 [70.6], 54.8 [57.1], 47.2 [44.3], 34.5 [32.8]; **MS** (**EI**, *m/z*, %): 219 (M^+, 39.25), 133 (100.00); **HRMS** (**EI**): Calcd. For $C_{12}H_{13}NO_3$: 219.0895; Found: 219.0894.

3b. Synthesis of (*R*)-1-(4-Bromophenylsulfonyl)pyrrolidin-3-ol (2p)

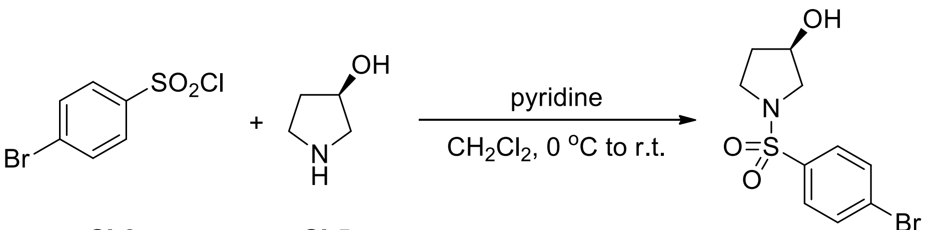

SI-8 **SI-5** **2p**

(*R*)-Pyrrolidin-3-ol (**SI-5**, 871.2 mg, 10.0 mmol, 1.0 equiv.) was dissolved in CH_2Cl_2 (10 mL) and pyridine (1.2 mL, 15.0 mmol, 1.5 equiv.) were added. The reaction mixture was cooled at 0 °C and a solution of 4-bromobenzene-1-sulfonyl chloride (**SI-8**, 2.555 g, 10.0 mmol, 1.0 equiv.) in CH_2Cl_2 (10 mL) was added dropwise by syringe over 30 min. The reaction mixture was stirred at 0 °C for 2 h and then warmed to room temperature and stirred for 10 hours. Water was added and the solution was extracted with CH_2Cl_2 (20 mL × 2). The combined organic layer was washed with water (30 mL × 1) and brine (20 mL × 1), dried over Na_2SO_4. Then remove the solvent, after purification by silica-gel chromatography (eluant: petroleum ether/ethyl acetate = 1/1) give the title compound **2p** as white solid (2.346 g, yield 77%, ee 99.7%). The enantiomeric excess was determined by chiral HPLC, AD-H (4.6 mm × 250 mm, 5 μm), Hexane/IPA = 70/30 (v/v), 0.7 mL/min, λ = 214 nm, tR

(major) = 11.23 min, tR (minor) = 8.82 min. M.p. 103–105 °C. **IR (KBr):** 3498, 3087, 2946, 2878, 1573, 1471, 1388, 1328, 1176, 1150, 1113, 1092, 1067, 1007, 828, 794, 741, 705, 614, 581 cm^{-1}; **^{1}H NMR** ($CDCl_3$, 400 MHz) [mixture of 2 rotamers] δ = 7.72–7.65 (m, 4H), 4.43–4.39 (m, 1H), 3.44–3.32 (m, 3H), 3.26 [3.23] (t, *J* = 1.6 Hz, 1H), 2.00–1.92 (m, 1H), 1.89–1.82 (m, 1H), 1.60 (s, 1H); **^{13}C NMR** ($CDCl_3$, 100 MHz) δ = 135.7, 132.3, 128.9, 127.7, 70.4, 56.1, 46.0, 34.1; **MS** (**EI**, *m/z*, %): 305 (M^+, 6.19), 86 (100.00); **HRMS** (**EI**): Calcd. For $C_{10}H_{12}NO_3SBr$: 304.9721; Found: 304.9719.

3c. Synthesis of (*S*)-5-(Naphthalen-1-yl)pentan-2-ol (2q)

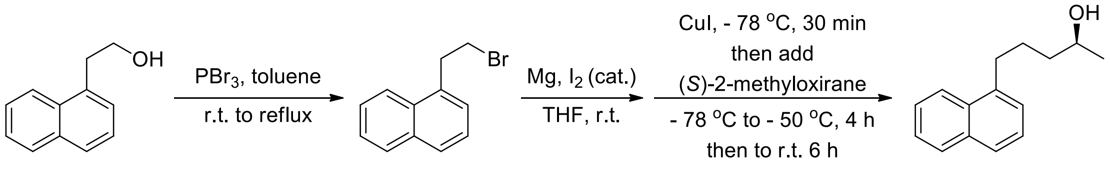

2f **SI-9** **2q**

Step 1: 2-(Naphthalen-1-yl)ethanol (**2f**, 17.22 g, 100 mmol, 1.0 equiv.) was dissolved in toluene (80 mL), then PBr_3 (4.7 mL, 50 mmol, 0.5 equiv.) was added dropwise over 10 min, the reaction mixture was further stirred under reflux for 5 h. After the reaction mixture was cooled to room temperature, a solution of $NaHCO_3$ and $Na_2S_2O_3$ (1:1 *w*/*w*) was added, the mixture was filtered through a pad of Celite and the Celite was washed with ether. The filtrate was extracted with ether (50 mL × 2), The combined organic layer was washed with water (100 mL × 2), dried over $MgSO_4$. After remove the solvent give 1-(2-bromoethyl)naphthalene (**SI-9**) as light yellow liquid (21.36 g, yield 91%), **^{1}H NMR** ($CDCl_3$, 400 MHz) δ = 8.01 (d, *J* = 8.4 Hz, 1H), 7.89 (d, *J* = 8.8 Hz, 1H), 7.80 (d, *J* = 8.0 Hz, 1H), 7.58–7.49 (m, 2H), 7.44 (t, *J* = 7.4 Hz, 1H), 7.39 (d, *J* = 7.2 Hz, 1H), 3.73–3.62 (m, 4H). The characterization data are consistent with previous report.[21]

Step 2: A 3-necked 100-mL round bottomed flask (pre dried by hot air gun under vacuo) fitted with magnetic stirrer was evacuated/N_2 filled (3 times), and then

magnesium turnings (612.6 g, 25.2 mmol, 1.4 equiv.), THF (30 mL) and a grain of iodine were successively added. At first, about 2 mL solution of 1-(2-bromoethyl)naphthalene (**SI-9**, 5.925 g, 25.2 mmol, 1.4 equiv.) in THF (10 mL) was added to the mixture by syringe, after the reaction was initiated, the solution of 1-(2-bromoethyl)naphthalene was added dropwise, and keep the reaction mixture slightly boiling, after about 15 min, 1-(2-bromoethyl)naphthalene was added up, the mixture was further stirred at room temperature until the magnesium turnings disappeared. A 3-necked 100-mL round bottomed flask (pre dried by hot air gun under vacuo) fitted with magnetic stirrer was evacuated/N_2 filled (3 times), and then CuI (480 mg, 2.52 mmol, 0.14 equiv.) was added, After cooled to -78 ^{o}C, the freshly prepared Grignard reagent was added by syringe over 15 min. then the reaction mixture was stirred for additional 15 min. then a solution of (*S*)-2-methyloxirane (1.045 g, 18 mmol, 1.0 equiv.) in THF (10 mL) was added dropwise by syringe over 1 h. After the (*S*)-2-methyloxirane was added up, the reaction mixture was warmed to -50 ^{o}C and stirred for 4 h. then warmed to room temperature and stirred for 6 hours. After the reaction was complete, 50 mL HCl (about 1.0 M) was added, the mixture was filtered through a pad of Celite and the Celite was washed with ether. The filtrate was extracted with ether (80 mL × 2). The combined organic layer was washed with water (100 mL × 2), dried over $MgSO_4$. Then remove the solvent, after purification by silica-gel chromatography (eluant: petroleum ether/ethyl acetate = 10/1) give the title compound **2q** as colorless oil liquid (3.41 g, yield 88%, ee 96.1%). The enantiomeric excess was determined by chiral HPLC, Lux 5u Cellulose-3 (4.6 mm × 250 mm), Hexane/IPA = 90/10 (v/v), 0.7 mL/min, λ = 214 nm, tR (major) = 11.127 min, tR (minor) = 12.027 min. **IR (film):** 3363, 3045, 3006, 2964, 2935, 2866, 1596, 1509, 1463, 1396, 1374, 1128, 1088, 778 cm^{-1}; **^{1}H NMR** ($CDCl_3$, 400 MHz) δ = 8.05 (d, *J* = 8.4 Hz, 1H), 7.86 (dd, *J* = 7.8, 1.2 Hz, 1H), 7.72 (d, *J* = 8.4 Hz, 1H), 7.54–7.46 (m, 2H), 7.40 (t, *J* = 7.6 Hz, 1H), 7.33 (d, *J* = 6.4 Hz, 1H), 3.88–3.82 (m, 1H), 3.11 (t, *J* = 7.6 Hz, 2H), 1.96–1.74 (m, 2H), 1.67–1.53 (m, 2H), 1.33 (d, *J* = 4.4 Hz, 1H), 1.20 (d, *J* = 6.0 Hz, 3H); **^{13}C NMR** ($CDCl_3$, 100 MHz) δ = 138.4, 133.8, 131.7, 128.6, 126.4, 125.8, 125.6, 125.4, 125.3, 123.7, 67.7, 39.1, 32.8, 26.8, 23.4; **MS** (**EI**, *m/z*, %): 214

(M$^+$, 32.62), 154 (100.00); **HRMS** (**EI**): Calcd. For $C_{15}H_{18}O$: 214.1358; Found: 214.1362.

3d. Synthesis of 2-(4-(benzyloxy)phenyl)ethanol (6q)

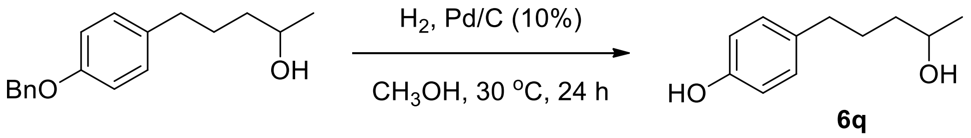

The starting material 5-(4-(benzyloxy)phenyl)pentan-2-ol was prepared according to reported procedures for the preparation of **2q**.

A solution of 5-(4-(benzyloxy)phenyl)pentan-2-ol (1.352 g, 5.0 mmol) in MeOH (30 mL) was degassed and flushed with N_2 three times. After that 10% Pd/C (135.2 mg, 10 w%) was added and the solution was degassed again with N_2. A balloon of hydrogen was attached and the reaction mixture was stirred at 30 ^{o}C for 24 hours. The mixture was filtered through a pad of Celite and the Celite was washed with MeOH. The filtrate was concentrated to dryness, the residue was purified by flash chromatography on silica gel (PE/EA = 2/1, v/v) to give compound **6q** as colorless liquid (892.6 mg, 99%). **IR (film):** 3330, 3020, 2967, 2934, 2858, 1613, 1597, 1515, 1452, 1375, 1240, 1172, 1127, 1109, 939, 830, 767, 558 cm^{-1}; ^{1}**H NMR** ($CDCl_3$, 400 MHz) δ = 7.03 (dt, *J* = 8.8, 2.8 Hz, 2H), 6.74 (dt, *J* = 8.4, 2.4 Hz, 2H), 5.06 (brs, 1H), 3.87–3.79 (m, 1H), 2.56 (t, *J* = 7.4 Hz, 2H), 1.77–1.42 (m, 4H), 1.40 (brs, 1H), 1.19 (d, *J* = 6.4 Hz, 3H); ^{13}**C NMR** ($CDCl_3$, 100 MHz) δ = 153.8, 134.3, 129.4, 115.2, 68.3, 38.7, 34.9, 27.7, 23.4; **MS** (**EI**, *m/z*, %): 180 (M$^+$, 17.49), 120 (100.00); **HRMS** (**EI**): Calcd. For $C_{11}H_{16}O_2$: 180.1150; Found: 180.1158.

3e. Synthesis of *N*-(2-hydroxyethyl)-*N*-(4- (hydroxymethyl)phenyl)benzamide (6r)

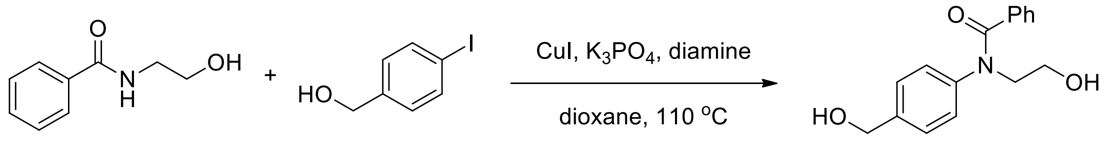

Compound **6r** was prepared according to reported procedures.[27] A Schlenk tube was charged with CuI (23.8 mg, 0.125 mmol, 0.05 equiv.), *N*-(2-hydroxyethyl)benzamide[27] (495.6 mg, 3.0 mmol, 1.2 equiv.), K_3PO_4 (1.061 g, 5.0 mmol, 2.0 equiv.), evacuated and backfilled with argon. Racemic *trans*-1,2-cyclohexanediamine (28.5 mg, 0.25 mmol, 0.1 equiv.), (4-iodophenyl)methanol (585.1 mg, 2.5 mmol, 1.0 equiv.) and dioxane (10.0 mL) were added under argon. The Schlenk tube was sealed and the reaction mixture was stirred at 110 °C for 36 hours. The resulting suspension was allowed to reach room temperature. Water was added and the solution was extracted ten times with EtOAc. The combined organic extracts were dried (Na_2SO_4) and concentrated under reduced pressure to obtain a crude oil that was purified by flash chromatography on silica gel (EtOAc/Petroleum Ether = 2/1, v/v) to obtain *N*-(2-hydroxyethyl)-*N*-(4- (hydroxymethyl)phenyl)benzamide (**6r**) as a white solid (212 mg, 31%). m.p. 63–65 °C. **IR (KBr):** 3302, 3066, 3031, 2937, 2879, 1641, 1612, 1579, 1541, 1515, 1318, 1306, 1252, 1214, 1175, 1054, 1006, 928, 831, 808, 717, 692, 657 cm^{-1}; **^{1}H NMR** ($CDCl_3$, 400 MHz) δ = 7.79–7.77 (m, 2H), 7.53–7.49 (m, 1H), 7.44 (t, *J* = 7.4 Hz, 2H), 7.30 (d, *J* = 8.8 Hz, 2H), 6.91 (d, *J* = 8.4 Hz, 2H), 6.61 (brs, 1H), 4.63 (d, *J* = 5.6 Hz, 2H), 4.16 (t, *J* = 5.2 Hz, 2H), 3.88 (q, *J* = 5.2 Hz, 2H), 1.61 (t, *J* = 6.0 Hz, 1H); **^{13}C NMR** ($CDCl_3$, 100 MHz) δ = 167.6, 158.1, 134.4, 133.8, 131.6, 128.7, 128.6, 127.0, 114.6, 67.0, 65.0, 39.5; **MS** (**EI**, *m/z*, %): 271 (M^+, 0.39), 148 (100.00); **HRMS** (**EI**): Calcd. For $C_{16}H_{17}NO_3$: 271.1208; Found: 271.1206.

3f. Synthesis of *N*-(2-hydroxyethyl)-4-(hydroxymethyl)-*N*-methylbenzamide (6s)

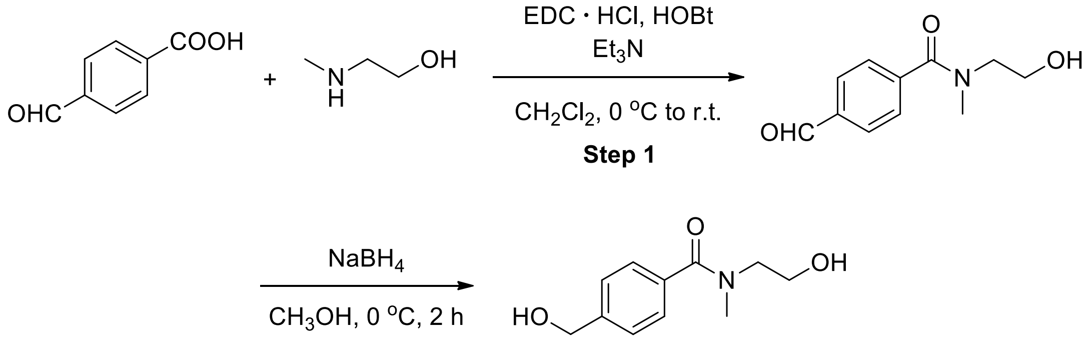

6s

Step 1: 4-Formyl-*N*-(2-hydroxyethyl)-*N*-methylbenzamide was prepared according to reported procedures.[27]

Step 2: To a 50-mL round bottomed flask, 4-formyl-*N*-(2-hydroxyethyl)-*N*-methylbenzamide (1.243 g, 6.0 mmol, 1.0 equiv.) was dissolved in CH_3OH (25 mL). After the solution was cooled to 0 ^{o}C, $NaBH_4$ (136.2 mg, 3.6 mmol, 0.6 equiv.) was added, and the reaction mixture was stirred at 0 ^{o}C for 2 hours. The mixture was concentrated to dryness, the residue was purified by flash chromatography on silica gel (CH_2Cl_2/MeOH = 15/1, v/v) to obtain *N*-(2-hydroxyethyl)-4-(hydroxymethyl)-*N*-methylbenzamide (**6s**) as light yellow liquid (778.6 mg, 62%). **IR (film):** 3373, 2933, 2881, 1608, 1568, 1518, 1487, 1456, 1406, 1083, 1050, 1017, 860, 836, 758, 625 cm ^{1}; **^{1}H NMR** (DMSO-d6, 400 MHz) [mixture of 2 rotamers] δ = 7.35 (s, 4H), 5.26 (t, *J* = 5.4 Hz, 1H), 4.79 (t, *J* = 5.4 Hz, 1H), 4.52 (d, *J* = 5.6 Hz, 2H), 3.62–3.60 (m, 1H), 3.49–3.46 (m, 2H), 3.28–3.26 (m, 1H), 2.96 [2.94] (s, 3H); **^{13}C NMR** (DMSO-d6, 100 MHz) [mixture of 2 rotamers] δ = 171.0 [170.2], 143.4 [143.7], 135.3, 126.7, 126.1, 62.5, 58.1 [58.5], 52.6 [49.6], 32.5 [38.3]; **MS** (**EI**, *m/z*, %): 209 (M^+, 3.1), 135 (100.00); **HRMS** (**EI**): Calcd. For $C_{11}H_{15}NO_3$: 209.1052; Found: 209.1056.

3g. Synthesis of (*R*)-(4-(hydroxymethyl)phenyl)(3-hydroxypyrrolidin-1-yl)methanone (6t)

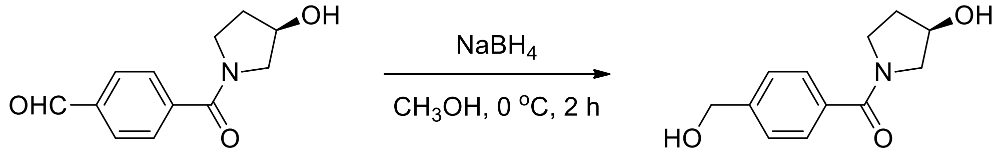

2n **6t**

To a 25-mL round bottomed flask, compound **2n** (252.1 mg, 1.15 mmol, 1.0 equiv.) was dissolved in CH_3OH (4.0 mL). After the solution was cooled to 0 ^{o}C, $NaBH_4$ (26.1 mg, 0.69 mmol, 0.6 equiv.) was added, and the reaction mixture was stirred at 0 ^{o}C for 2 hours. The mixture was concentrated to dryness, the residue was purified by flash chromatography on silica gel (CH_2Cl_2/MeOH = 20/1, v/v) to obtain compound **6t** as white solid (211.3 mg, 83%). m.p. 116–118 ^{o}C. **IR (KBr):** 3382, 2981, 2890, 1608, 1567, 1455, 1435, 1321, 1219, 1174, 1102, 1050, 1020, 1000, 978, 869, 836, 754 cm^{-1}; ^{1}**H NMR** (DMSO-d6, 400 MHz) [mixture of 2 rotamers] δ = 7.465 [7.474] (d, *J* = 8.0 Hz, 2H), 7.36 (d, *J* = 7.6 Hz, 2H), 5.28 (t, *J* = 5.6 Hz, 1H), 4.93 [5.01] (d, *J* = 3.2 Hz, 1H), 4.53 (d, *J* = 6.4 Hz, 2H), 4.25–4.19 [4.34–4.29] (m, 1H), 3.61–3.35 (m, 3H), 3.18 [3.41–3.37 (m, 1H)] (d, *J* = 10.8 Hz, 1H), 1.98–1.85 (m, 1H), 1.85–1.73 (m, 1H); ^{13}**C NMR** (DMSO-d6, 100 MHz) [mixture of 2 rotamers] δ = 168.5 [168.4], 144.3 [144.2], 135.33 [135.29], 127.03 [126.95], 126.0, 69.4 [68.0], 62.5, 57.1 [54.3], 44.0 [46.8], 32.2 [34.3]; **MS** (**EI**, *m/z*, %): 221 (M^{+}, 29.76), 135 (100.00); **HRMS** (**EI**): Calcd. For $C_{12}H_{15}NO_3$: 221.1052; Found: 221.1049.

3h. Synthesis of 4-(4-(3-hydroxypropoxy)phenyl)butan-2-ol (6u)

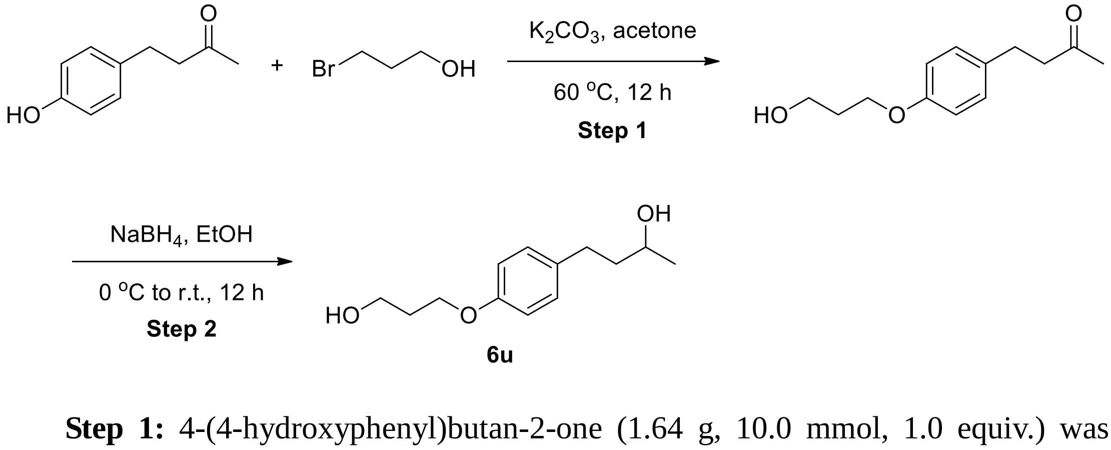

6u

Step 1: 4-(4-hydroxyphenyl)butan-2-one (1.64 g, 10.0 mmol, 1.0 equiv.) was dissolved in acetone (20 ml) and 3-bromopropan-1-ol (1.38 g, 0.9 ml, 10 mmol, 1.0

equiv.), K_2CO_3 (1.38 g, 10.0 mmol, 1.0 equiv.) were sequentially added. The reaction mixture was warmed to 60 °C for 12 h. After cooling, the solid was filtered off and water was added and the solution was extracted with EtOAc (50 mL × 20). The combined organic extracts were dried (Na_2SO_4) and concentrated under reduced pressure to obtain a crude oil that was purified by column chromatography on silica gel (EtOAc/Petroleum Ether = 1/2, v/v) to obtain 4-(4-(3-hydroxypropoxy)phenyl) butan-2-one as a colourless oil (1.32 g, 60 %). **^{1}H NMR** ($CDCl_3$, 400 MHz) δ = 7.05 (d, *J* = 8.5 Hz, 2H), 6.79 (d, *J* = 8.5 Hz, 2H), 4.04 (t, *J* = 6.0 Hz, 2H), 3.80 (t, *J* = 6.0 Hz, 2H), 2.79 (t, *J* = 7.2 Hz, 2H), 2.68 (t, *J* = 7.0 Hz, 2H), 2.58 (s, 1H), 2.09 (s, 3H), 1.99 (p, *J* = 6.0 Hz, 2H).

Step 2: 4-(4-(3-hydroxypropoxy)phenyl)butan-2-one (1.26 g, 5.67 mmol, 1.0 equiv.) was dissolved in EtOH (20 ml), the mixture was cooled to 0 °C followed by addition of $NaBH_4$ (215 mg, 5.67 mmol, 1.0 equiv.). The reaction mixture was stirred at 0 °C for 30 minutes and then warmed to room temperature and stirred for 12 hours. Water was added and the solution was extracted with EtOAc (50 mL × 20). The combined organic extracts were dried (Na_2SO_4) and concentrated under reduced pressure to obtain 4-(4-(3-hydroxypropoxy)phenyl)butan-2-ol (**6u**) as a white solid (1.21 g, 95 %). **^{1}H NMR** ($CDCl_3$, 500 MHz) δ = 7.08 (d, *J* = 8.5 Hz, 2H), 6.80 (d, *J* = 9.0 Hz, 2H), 4.05 (t, *J* = 6.0 Hz, 2H), 3.81–3.75 (m, 3H), 2.69–2.63 (m, 1H), 2.61–2.55 (m, 3H), 1.99 (p, *J* = 6.0 Hz, 2H), 1.77–1.64 (m, 2H), 1.19 (d, *J* = 6.0 Hz, 3H); **^{13}C NMR** ($CDCl_3$, 100 MHz) δ =156.9, 134.3, 129.2, 114.4, 67.3, 65.6, 60.1, 40.9, 31.9, 31.1, 23.4; **MS** (**EI**, *m/z*, %): 224 (M^+, 0.51), 107 (100.00); **HRMS** (**EI**): Calcd. For $C_{13}H_{20}O_3$: 224.1412; Found: 224.1417.

4. Deoxyfluorination of Monoalcohols 2 Using CpFluor 1i or 1c.

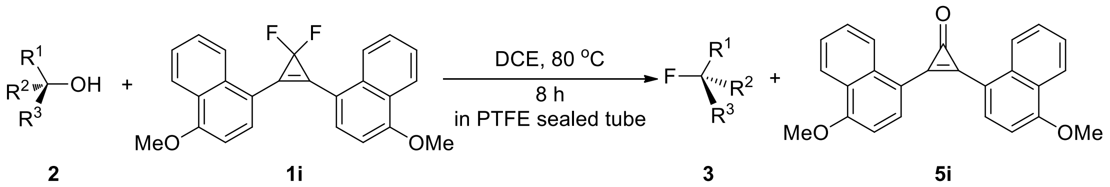

General Procedures:

To a dried polytetrafluoroethene (PTFE) sealed tube, alcohols **2** (0.2 mmol or 0.4 mmol, 1.0 equiv.), CpFluor **1i** (0.22 mmol or 0.44 mmol, 1.1 equiv.) and solvent DCE (1.5 mL or 3.0 mL) were sequentially added. The tube was sealed and immersed in an oil bath at 80 °C. After being stirred for 8 hours, the reaction mixture was cooled to room temperature and precipitated with ether (6 mL). The precipitate (mainly 2,3-bis(4-methoxynaphthalen-1-yl)cycloprop-2-enone) was filtered and washed with ether. The filtrate was concentrated under reduced pressure and the residue was purified by flash chromatography on silica gel to give the corresponding alkyl fluoride **3**. (Deoxyfluorination of alcohols **2** using CpFluor **1c** was performed similarly.)

1-Fluorooctadecane (3b)[22]

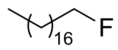

Performed on 0.2 mmol scale, eluted with hexane to give **3b** (51.3 mg, 93% yield; 74% yield when **1c** was used instead of **1i**). White solid. M.p. 28–29 °C. **^{1}H NMR** ($CDCl_3$, 400 MHz) δ = 4.43 (dt, *J* = 47.2, 6.4 Hz, 2H), 1.75–1.62 (m, 2H), 1.44–1.26 (m, 30H), 0.89 (t, *J* = 6.6 Hz, 3H); **^{19}F NMR** ($CDCl_3$, 376 MHz) δ = –218.0 (m, 1F); **^{13}C NMR** ($CDCl_3$, 100 MHz) δ = 84.2 (d, *J* = 163.1 Hz), 32.0, 30.4 (d, *J* = 18.5 Hz), 29.72, 29.70, 29.68, 29.59, 29.55, 29.4, 29.3, 25.2 (d, *J* = 5.4 Hz), 22.7, 14.1; **MS (EI**, *m/z*, %): 272 (M^+, 1.79), 57 (100.00).

1-Bromo-12-fluorododecane (3c)

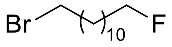

Performed on 0.2 mmol scale, eluted with hexane to give **3c** (47.3 mg, 89% yield). Colorless liquid. **IR (film):** 2926, 2855, 1465, 1390, 1254, 1007, 722, 646, 563 cm^{-1};

^{1}H NMR ($CDCl_3$, 400 MHz) δ = 4.43 (dt, *J* = 47.2, 6.2 Hz, 2H), 3.40 (t, *J* = 6.8 Hz, 2H), 1.88–1.81 (m, 2H), 1.75–1.62 (m, 2H), 1.43–1.28 (m, 16H); **^{19}F NMR** ($CDCl_3$, 376 MHz) δ = –218.0 (m, 1F); **^{13}C NMR** ($CDCl_3$, 100 MHz) δ = 84.2 (d, *J* = 163.4 Hz), 34.0, 32.8, 30.4 (d, *J* = 19.6 Hz), 29.5, 29.4, 29.2, 28.7, 28.1, 25.1 (d, *J* = 5.3 Hz); **MS** (**EI**, *m/z*, %): 266 (M^+, 0.76), 137 (100.00), 135 (96.15); **HRMS** (**EI**): Calcd. For $C_{12}H_{24}FBr$: 266.1045; Found: 266.1050.

4-(3-Fluoropropyl)-1,1'-biphenyl (3d)

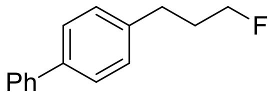

Performed on 0.4 mmol scale, eluted with hexane/ethyl acetate = 100/1 to give **3d** (73.9 mg, 86% yield). Colorless liquid. **IR (film):** 3056, 3028, 2963, 2861, 1487, 1449, 1410, 1389, 1076, 1063, 1029, 1008, 906, 845, 762, 731, 698 cm^{-1}; **^{1}H NMR** ($CDCl_3$, 400 MHz) δ = 7.60–7.58 (m, 2H), 7.54 (d, *J* = 8.0 Hz, 2H), 7.46–7.42 (m, 2H), 7.34 (tt, *J* = 7.4, 1.4 Hz, 1H), 7.29 (d, *J* = 8.0 Hz, 2H), 4.50 (dt, *J* = 48.0, 5.8 Hz, 2H), 2.80 (t, *J* = 7.8 Hz, 2H), 2.12–1.99 (m, 2H); **^{19}F NMR** ($CDCl_3$, 376 MHz) δ = –219.8 (m, 1F); **^{13}C NMR** ($CDCl_3$, 100 MHz) δ = 140.9, 140.2, 139.0, 128.9, 128.7, 127.1, 127.0, 126.9, 83.0 (d, *J* = 164.0 Hz), 31.9 (d, *J* = 19.6 Hz), 30.9 (d, *J* = 5.3 Hz); **MS** (**EI**, *m/z*, %): 214 (M^+, 35.46), 167 (100.00); **HRMS** (**EI**): Calcd. For $C_{15}H_{15}F$: 214.1158; Found: 214.1155.

1-(Fluoromethyl)-4-(methylsulfonyl)benzene (3e)

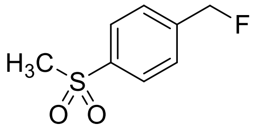

Performed on 0.4 mmol scale, eluted with hexane/Et_2O = 1/4 to give **3e** (73.1 mg, 97% yield). Light yellow solid. M.p. 56–58 ^oC. **IR (KBr):** 3100, 3078, 3013, 3001, 2946, 2921, 2886, 1602, 1456, 1411, 1377, 1305, 1209, 1183, 1145, 1089, 1028, 1011, 969, 952, 814, 763, 656, 551, 527, 448 cm^{-1}; **^{1}H NMR** ($CDCl_3$, 400 MHz) δ = 7.98 (d, *J* = 7.6 Hz, 2H), 7.56 (d, *J* = 8.4 Hz, 2H), 5.49 (d, *J* = 46.8 Hz, 2H), 3.06 (s, 3H); **^{19}F**

NMR ($CDCl_3$, 376 MHz) δ = –215.1 (t, *J* = 46.2 Hz, 1F); ^{13}**C NMR** ($CDCl_3$, 100 MHz) δ = 142.3 (d, *J* = 17.4 Hz), 140.4 (d, *J* = 2.1 Hz), 127.6, 127.2 (d, *J* = 6.9 Hz), 83.0 (d, *J* = 169.3 Hz), 44.4; **MS** (**EI**, *m/z*, %): 188 (M^+, 36.13), 109 (100.00); **HRMS** (**EI**): Calcd. For $C_8H_9O_2FS$: 188.0307; Found: 188.0302.

1-(2-Fluoroethyl)naphthalene (3f)

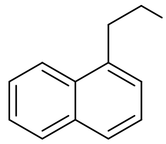

Performed on 0.4 mmol scale, eluted with hexane/Et_2O = 100/1 to give **3f** (53.1 mg, 76% yield). Colorless liquid. **IR (film):** 3048, 2968, 2901, 1597, 1511, 1397, 1046, 1020, 1005, 798, 777 cm^{-1}; ^{1}**H NMR** ($CDCl_3$, 400 MHz) δ = 8.04 (d, *J* = 8.4 Hz, 1H), 7.89 (dd, *J* = 8.6, 2.0 Hz, 1H), 7.79 (d, *J* = 7.6 Hz, 1H), 7.58–7.49 (m, 2H), 7.46–7.40 (m, 2H), 4.78 (dt, *J* = 47.2, 6.8 Hz, 2H), 3.52 (dt, *J* = 20.4, 6.6 Hz, 2H); ^{19}**F NMR** ($CDCl_3$, 376 MHz) δ = –213.4 (tt, *J* = 46.2, 19.9 Hz, 1F); ^{13}**C NMR** ($CDCl_3$, 100 MHz) δ = 133.9, 132.8 (d, *J* = 7.4 Hz), 132.0, 128.9, 127.5, 127.1, 126.1, 125.6, 125.5, 123.3, 83.5 (d, *J* = 169.3 Hz), 33.8 (d, *J* = 21.1 Hz); **MS** (**EI**, *m/z*, %): 174 (M^+, 35.13), 141 (100.00); **HRMS** (**EI**): Calcd. For $C_{12}H_{11}F$: 174.0845; Found: 174.0844.

2-(5-Fluoropentyl)naphthalene (3g)

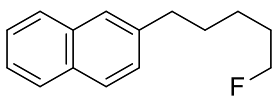

Performed on 0.4 mmol scale, eluted with hexane/Et_2O = 50/1 to give **3g** (74.4 mg, 86% yield). Colorless liquid. **IR (film):** 3052, 3020, 2936, 2858, 1633, 1601, 1508, 1462, 1062, 1040, 982, 961, 892, 855, 818, 748, 476 cm^{-1}; ^{1}**H NMR** ($CDCl_3$, 400 MHz) δ = 7.83–7.78 (m, 3H), 7.63 (s, 1H), 7.49–7.41 (m, 2H), 7.34 (dd, *J* = 8.8, 1.6 Hz, 1H), 4.46 (dt, *J* = 47.2, 6.2 Hz, 2H), 2.81 (t, *J* = 7.6 Hz, 2H), 1.83–1.69 (m, 4H), 1.54–1.46 (m, 2H); ^{19}**F NMR** ($CDCl_3$, 376 MHz) δ = –218.0 (m, 1F); ^{13}**C NMR** ($CDCl_3$, 100 MHz) δ = 139.9, 133.6, 132.0, 127.8, 127.6, 127.4, 127.3, 126.3, 125.8, 125.1, 84.0 (d, *J* = 163.2 Hz), 35.9, 30.9, 30.3 (d, *J* = 18.9 Hz), 24.9 (d, *J* = 5.5 Hz);

MS (**EI**, *m/z*, %): 216 (M^+, 30.80), 141 (100.00); **HRMS** (**EI**): Calcd. For $C_{15}H_{17}F$: 216.1314; Found: 216.1318.

1-Fluoroadamantane (3i)[23]

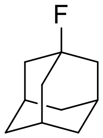

Performed on 0.5 mmol scale, eluted with hexane/CH_2Cl_2 = 20/1 to give **3i** (63.8 mg, 83% yield). White solid. **^{1}H NMR** ($CDCl_3$, 400 MHz) δ = 2.22 (s, 3H), 1.89−1.87 (m, 6H), 1.62 (s, 6H); **^{19}F NMR** ($CDCl_3$, 376 MHz) δ = −128.5 (m, 1F); **MS** (**EI**, *m/z*, %): 154 (M^+, 97.41), 97 (100.00).

1-(3-Fluorobutyl)-4-methoxybenzene (3j)

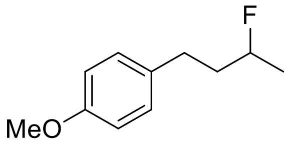

Performed on 0.4 mmol scale, eluted with hexane/ethyl acetate = 100/1 to give **3j** (62.8 mg, 86% yield). Colorless liquid. **IR (film):** 2979, 2936, 2836, 1613, 1514, 1465, 1385, 1301, 1247, 1178, 1133, 1061, 1037, 853, 828 cm^{-1}; **^{1}H NMR** ($CDCl_3$, 400 MHz) δ = 7.12 (d, *J* = 8.4 Hz, 2H), 6.84 (d, *J* = 8.4 Hz, 2H), 4.76–4.56 (dm, *J* = 48.6 Hz, 1H), 3.80 (s, 3H), 2.78–2.71 (m, 1H), 2.68–2.61 (m, 1H), 2.03–1.90 (m, 1H), 1.87−1.71 (m, 1H), 1.35 (dd, *J* = 24.2, 6.2 Hz, 3H); **^{19}F NMR** ($CDCl_3$, 376 MHz) δ = −174.2 (m, 1F); **^{13}C NMR** ($CDCl_3$, 100 MHz) δ = 157.8, 133.5, 129.3, 113.8, 90.0 (d, *J* = 164.1 Hz), 55.2, 38.9 (d, *J* = 20.6 Hz), 30.4 (d, *J* = 4.5 Hz), 21.0 (d, *J* = 22.1 Hz); **MS** (**EI**, *m/z*, %): 182 (M^+, 23.13), 121 (100.00); **HRMS** (**EI**): Calcd. For $C_{11}H_{15}OF$: 182.1107; Found: 182.1108.

1-(3-Fluorobutyl)-3-methylbenzene (3k)

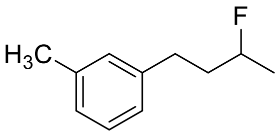

Performed on 0.4 mmol scale, eluted with hexane/ethyl acetate = 100/1 to give **3k** (53.6 mg, 81% yield; 74% yield when **1c** was used instead of **1i**). Colorless liquid. **IR (film):** 3019, 2979, 2934, 2864, 1610, 1590, 1489, 1453, 1385, 1345, 1133, 1064, 928, 878, 840, 783, 699 cm^{-1}; ^{1}**H NMR** ($CDCl_3$, 400 MHz) δ = 7.20 (t, *J* = 7.8 Hz, 1H), 7.04–7.00 (m, 3H), 4.77–4.58 (dm, *J* = 49.2 Hz, 1H), 2.81–2.74 (m, 1H), 2.70–2.63 (m, 1H), 2.35 (s, 3H), 2.05–1.92 (m, 1H), 1.90–1.74 (m, 1H), 1.36 (dd, *J* = 23.6, 6.4 Hz, 3H); ^{19}**F NMR** ($CDCl_3$, 376 MHz) δ = –174.2 (m, 1F); ^{13}**C NMR** ($CDCl_3$, 100 MHz) δ = 141.4, 137.9, 129.2, 128.3, 126.6, 125.4, 89.9 (d, *J* 164.1 = Hz), 38.7 (d, *J* = 19.9 Hz), 31.2 (d, *J* = 3.8 Hz), 21.3, 20.9 (d, *J* = 22.6 Hz); **MS** (**EI**, *m/z*, %): 166 (M^+, 83.35), 106 (100.00); **HRMS** (**EI**): Calcd. For $C_{11}H_{15}F$: 166.1158; Found: 166.1161.

2-(3-Fluorobutyl)naphthalene (3l)

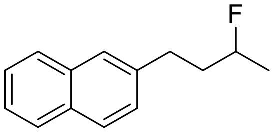

Performed on 0.4 mmol scale, eluted with hexane/ethyl acetate = 100/1 to give **3l** (71.1 mg, 88% yield). Colorless liquid. **IR (film):** 3053, 3021, 2978, 2935, 2863, 1634, 1601, 1508, 1448, 1385, 1344, 1132, 1068, 1019, 960, 925, 895, 856, 838, 818, 746, 475 cm^{-1}; ^{1}**H NMR** ($CDCl_3$, 400 MHz) δ = 7.88–7.83 (m, 3H), 7.70 (s, 1H), 7.55–7.47 (m, 2H), 7.40 (dd, *J* = 8.2, 1.4 Hz, 1H), 4.85–4.66 (dm, *J* = 48.8 Hz, 1H), 3.06–2.99 (m, 1H), 2.96–2.88 (m, 1H), 2.20–2.07 (m, 1H), 2.04–1.88 (m, 1H), 1.42 (dd, *J* = 24.0, 6.0 Hz, 3H); ^{19}**F NMR** ($CDCl_3$, 376 MHz) δ = –174.1 (m, 1F); ^{13}**C NMR** ($CDCl_3$, 100 MHz) δ = 138.9, 133.6, 132.0, 128.0, 127.6, 127.4, 127.2, 126.5, 125.9, 125.2, 90.0 (d, *J* = 164.1 Hz), 38.5 (d, *J* = 20.8 Hz), 31.5 (d, *J* = 4.5 Hz), 21.0 (d, *J* = 23.0 Hz); **MS** (**EI**, *m/z*, %): 202 (M^+, 42.23), 141 (100.00), 142 (85.93); **HRMS** (**EI**): Calcd. For $C_{14}H_{15}F$: 202.1158; Found: 202.1156.

(4*S*,6*S*)-4-Fluoro-6-methyl-5,6-dihydro-4*H*-thieno[2,3-*b*]thiopyran 7,7-dioxide (*trans*-3m)

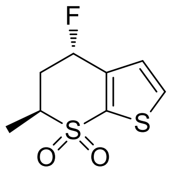

Performed on 0.2 mmol scale, eluted with hexane/Et_2O = 1/1 to give **3m** [43.7 mg, 99% total yield (dr 6.9/1)]. The major isomer was isolated by preparative HPLC, Kaseisorb LC ODS 2000 (10.0 mm × 250 mm, 5 μm), Acetonitrile/Water = 20/80 (v/v), 4.0 mL/min, λ = 220 nm. White solid. M.p. 76–79 ^{o}C. **IR (KBr):** 3120, 3112, 2943, 1312, 1281, 1267, 1140, 1118, 1044, 1026, 941, 842, 829, 756, 725, 690, 673, 608, 547, 526, 506 cm^{-1}; **^{1}H NMR** ($CDCl_3$, 400 MHz) δ = 7.63 (d, *J* = 4.8 Hz, 1H), 7.13 (d, *J* = 5.2 Hz, 1H), 5.61 (dt, *J* = 48.8, 3.0 Hz, 1H), 3.81–3.73 (m, 1H), 2.79–2.55 (m, 2H), 1.54 (d, *J* = 7.6 Hz, 3H); **^{19}F NMR** ($CDCl_3$, 376 MHz) δ = –161.6 (ddd, *J* = 48.9, 38.2, 14.7 Hz, 1F); **^{13}C NMR** ($CDCl_3$, 100 MHz) δ = 139.4 (d, *J* = 19.0 Hz), 139.1 (d, *J* = 5.8 Hz), 131.0 (d, *J* = 2.2 Hz), 127.6, 81.3 (d, *J* = 169.0 Hz), 52.1, 36.4 (d, *J* = 21.8 Hz), 10.3; **MS** (**EI**, *m/z*, %): 220 (M^+, 100.00), 114 (99.17), 106 (79.52); **HRMS** (**EI**): Calcd. For $C_8H_9O_2FS_2$: 220.0028; Found: 220.0030.

(4*R*,6*S*)-4-Fluoro-6-methyl-5,6-dihydro-4*H*-thieno[2,3-*b*]thiopyran 7,7-dioxide (*cis*-3m)

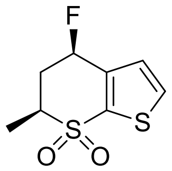

The minor isomer was isolated by preparative HPLC, Kaseisorb LC ODS 2000 (10.0 mm × 250 mm, 5 μm), Acetonitrile/Water = 20/80 (v/v), 4.0 mL/min, λ = 220 nm. White solid. M.p. 111–114 ^{o}C. **IR (KBr):** 3133, 3121, 2983, 2936, 1453, 1412, 1318, 1303, 1277, 1260, 1139, 1048, 1009, 963, 896, 763, 707, 678, 608, 566, 520, 510, 491 cm^{-1}; **^{1}H NMR** ($CDCl_3$, 400 MHz) δ = 7.64 (d, *J* = 4.4 Hz, 1H), 7.16 (d, *J* = 4.8 Hz, 1H), 5.68 (ddd, *J* = 49.1, 7.6, 5.5 Hz, 1H), 3.51–3.43 (m, 1H), 2.83–2.65 (m, 2H), 1.57 (dd, *J* = 6.0, 0.8 Hz, 3H); **^{19}F NMR** ($CDCl_3$, 376 MHz) δ = –167.2 (dt, *J* = 47.4, 14.6 Hz, 1F); **^{13}C NMR** ($CDCl_3$, 100 MHz) δ = 141.0 (d, *J* = 13.7 Hz), 140.8, 131.2

(d, J = 2.0 Hz), 126.4 (d, J = 2.3 Hz), 83.6 (d, J = 174.4 Hz), 55.3 (d, J = 5.9 Hz), 35.4 (d, J = 20.7 Hz), 12.3; **MS** (**EI**, *m/z*, %): 220 (M^+, 94.71), 114 (100.00); **HRMS** (**EI**): Calcd. For $C_8H_9O_2FS_2$: 220.0028; Found: 220.0027.

(*S*)-4-(3-Fluoropyrrolidine-1-carbonyl)benzaldehyde (3n)

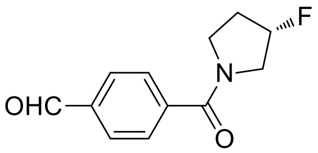

Prepared from alcohol **2n** (ee 99.4%) on 0.4 mmol scale, eluted with CH_2Cl_2/ethyl acetate = 2/1 to give **3n** (60.2 mg, 68% yield, ee 95.5%). The enantiomeric excess was determined by chiral HPLC, ID (4.6 mm × 150 mm, 3 μm), Hexane/IPA = 80/20 (v/v), 0.7 mL/min, λ = 214 nm, t_R (major) = 28.53 min, t_R (minor) = 36.90 min. Light yellow liquid. **IR (film):** 3475, 2985, 2892, 1702, 1628, 1570, 1508, 1432, 1208, 967, 845, 831, 785, 757 cm^{-1}; ^{1}**H NMR** ($CDCl_3$, 400 MHz) [mixture of 2 rotamers] δ = 10.02 (s, 1H), 7.91 (d, J = 8.0 Hz, 2H), 7.66 [7.62] (d, J = 8.0 Hz, 2H), 5.32 [5.19] (d, J = 51.2 Hz, 1H), 3.98–3.75 (m, 2H), 3.70–3.47 (m, 2H), 2.38–2.21 (m, 1H), 2.17–1.90 (m, 1H); ^{19}**F NMR** ($CDCl_3$, 376 MHz) [mixture of 2 rotamers] δ = −177.5 [−178.3] (m, 1F); ^{13}**C NMR** ($CDCl_3$, 100 MHz) [mixture of 2 rotamers] δ = 191.4, 168.4 [168.7], 141.8 [142.0], 137.14 [137.07], 129.6 [129.7], 127.7 [127.6], 91.3 [92.3] (d, J = 175.3 Hz), 52.9 [55.3] (J = 23.5 Hz), 46.8 [43.9], 33.0 [30.9] (d, J = 20.6 Hz); **MS** (**EI**, *m/z*, %): 221 (M^+, 43.82), 133 (100.00); **HRMS** (**EI**): Calcd. For $C_{12}H_{12}NO_2F$: 221.0852; Found: 221.0853.

(*R*)-Benzyl 3-fluoropyrrolidine-1-carboxylate (3o)

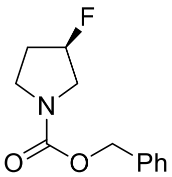

Prepared from alcohol **2o** (ee > 99.9%) on 0.2 mmol scale, eluted with hexane/ethyl acetate = 5/1 to give **3o** (29.8 mg, 67% yield, ee 95.0%). The enantiomeric excess was determined by chiral HPLC, Lux 5u Amylose-2 (4.6 mm × 250 mm), Hexane/IPA =

80/20 (v/v), 0.7 mL/min, λ = 214 nm, t_R (major) = 18.96 min, t_R (minor) = 16.25 min. Light yellow liquid. **IR (film):** 3501, 3033, 2956, 2891, 1705, 1421, 1355, 1209, 1113, 956, 859, 769, 754, 699 cm^{-1}; ^{1}**H NMR** ($CDCl_3$, 400 MHz) [mixture of 2 rotamers] δ = 7.37–7.29 (m, 5H), 5.29–5.15 (m, 3H), 3.83–3.46 (m, 4H), 2.29–2.19 (m, 1H), 2.09–1.87 (m, 1H); ^{19}**F NMR** ($CDCl_3$, 376 MHz) [mixture of 2 rotamers] δ = −177.2 [−177.6] (m, 1F); ^{13}**C NMR** ($CDCl_3$, 100 MHz) [mixture of 2 rotamers] δ = 154.8 [154.7], 136.69 [136.66], 128.4, 127.94, 127.86, 92.8 [92.0] (d, *J* = 176.0 Hz), 66.9, 52.8 [52.5] (d, *J* = 22.9 Hz), 43.9 [43.6], 32.4 [31.6] (d, *J* = 20.8 Hz); **MS** (**EI**, *m/z*, %): 223 (M^+, 17.65), 91 (100.00); **HRMS** (**EI**): Calcd. For $C_{12}H_{14}NO_2F$: 223.1009; Found: 223.1006.

(*S*)-1-((4-Bromophenyl)sulfonyl)-3-fluoropyrrolidine (3p)

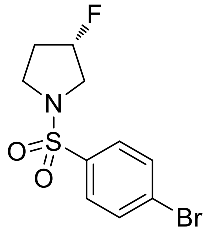

Prepared from alcohol **2p** (ee 99.7%) on 0.3 mmol scale, eluted with hexane/ethyl acetate = 5/1 to give **3p** (44.3 mg, 48% yield, ee 96.9%). The enantiomeric excess was determined by chiral HPLC, Lux 5u Amylose-2 (4.6 mm × 250 mm), Hexane/IPA = 80/20 (v/v), 0.7 mL/min, λ = 214 nm, t_R (major) = 25.577 min, t_R (minor) = 27.527 min. (69% yield when using **1c**). White solid. M.p. 115–118 °C. **IR (KBr):** 3094, 2990, 2954, 2886, 1573, 1473, 1390, 1340, 1276, 1233, 1218, 1167, 1125, 1092, 1077, 1067, 1030, 1008, 957, 856, 829, 796, 741, 705, 617, 583 cm^{-1}; ^{1}**H NMR** ($CDCl_3$, 400 MHz) δ = 7.71–7.66 (m, 4H), 5.21–5.08 (dm, *J* = 52.2 Hz, 1H), 3.58–3.53 (m, 2H), 3.48–3.47 (m, 1H), 3.29–3.23 (m, 1H), 2.21–2.11 (m, 1H), 2.06–1.86 (m, 1H); ^{19}**F NMR** ($CDCl_3$, 376 MHz) δ = −176.0 (m, 1F); ^{13}**C NMR** ($CDCl_3$, 100 MHz) δ = 135.7, 132.4, 128.9, 127.9, 92.0 (d, *J* = 178.7 Hz), 54.4 (d, *J* = 23.5 Hz), 45.9, 32.5 (d, *J* = 20.7 Hz); **MS** (**EI**, *m/z*, %): 307 (M^+, 13.22), 121 (100.00); **HRMS** (**EI**): Calcd. For $C_{10}H_{11}NO_2FSBr$: 306.9678; Found: 306.9680.

(*R*)-1-(4-fluoropentyl)naphthalene (3q)

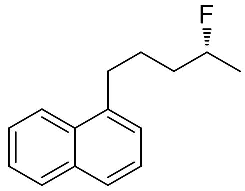

Prepared from alcohol **2n** (ee 96.1%) on 0.4 mmol scale, eluted with hexane/Et_2O = 50/1 to give **3q** (69.7 mg, 80% yield, ee 92.8%). The enantiomeric excess was determined by chiral HPLC, OD-H (4.6 mm × 250 mm, 5 μm), Hexane/IPA = 95/5 (v/v), 0.7 mL/min, λ = 214 nm, t_R (major) = 7.92 min, t_R (minor) = 7.49 min. Colorless liquid. **IR (film):** 3046, 2977, 2938, 2868, 1597, 1510, 1463, 1386, 1133, 1087, 925, 814, 799, 790, 778 cm^{-1}; ^{1}**H NMR** ($CDCl_3$, 400 MHz) δ = 8.08 (d, *J* = 8.0 Hz, 1H), 7.90 (dd, *J* = 8.6, 2.0 Hz, 1H), 7.76 (d, *J* = 8.4 Hz, 1H), 7.58–7.49 (m, 2H), 7.44 (t, *J* = 7.6 Hz, 1H), 7.36 (d, *J* = 7.2 Hz, 1H), 4.83–4.63 (dm, *J* = 48.8 Hz, 1H), 3.15 (t, *J* = 7.6 Hz, 2H), 2.04–1.61 (m, 4H), 1.36 (dd, *J* = 24.0, 6.4 Hz, 4H); ^{19}**F NMR** ($CDCl_3$, 376 MHz) δ = –172.5 (m, 1F); ^{13}**C NMR** ($CDCl_3$, 100 MHz) δ = 138.2, 133.9, 131.8, 128.8, 126.6, 125.9, 125.7, 125.5, 125.4, 123.7, 90.8 (d, *J* = 164.1 Hz), 36.8 (d, *J* = 21.2 Hz), 32.7, 26.2 (d, *J* = 4.8 Hz), 21.0 (d, *J* = 22.5 Hz); **MS** (**EI**, *m/z*, %): 216 (M^+, 26.94), 141 (100.00); **HRMS** (**EI**): Calcd. For $C_{15}H_{17}F$: 216.1314; Found: 216.1313.

1-((3*S*,8*S*,9*S*,10*R*,13*S*,14*S*,17*S*)-3-Fluoro-10,13-dimethyl-2,3,4,7,8,9,10,11,12,13,14,15,16,17-tetradecahydro-1*H*-cyclopenta[*a*]phenanthren-17-yl)ethanone (3r)[24]

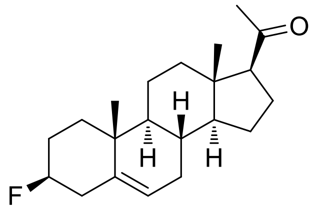

Performed on 0.2 mmol scale, eluted with hexane/Et_2O = 10/1 to give **3r** (61.2 mg, 96% yield; 83% yield when **1c** was used instead of **1i**). White solid. M.p. 157–159 °C. ^{1}**H NMR** ($CDCl_3$, 400 MHz) δ = 5.38 (d, *J* = 4.4 Hz, 1H), 4.47–4.26 (dm, *J* = 50.4 Hz, 1H), 2.52 (t, *J* = 9.0 Hz, 1H), 2.43 (d, *J* = 7.2 Hz, 2H), 2.20–2.11 (m, 4H), 2.05–1.97

(m, 3H), 1.90–1.85 (m, 1H), 1.74–1.39 (m, 8H), 1.27–0.93 (m, 7H), 0.62 (s, 3H); **^{19}F NMR** ($CDCl_3$, 376 MHz) δ = –168.0 (dm, *J* = 50.8 Hz, 1F); **^{13}C NMR** ($CDCl_3$, 100 MHz) δ = 209.3, 139.3 (d, *J* = 12.1 Hz), 122.7, 92.6 (d, *J* = 173.6 Hz), 63.6, 56.8, 49.8 (d, *J* = 1.7 Hz), 43.9, 39.3 (d, *J* = 18.7 Hz), 38.7, 36.5 (d, *J* = 1.5 Hz), 36.3 (d, *J* = 10.4 Hz), 31.8, 31.7, 31.5, 28.7 (d, *J* = 17.6 Hz), 24.4, 22.8, 21.1, 19.2, 13.2; **MS** (**ESI**): *m/z* 319.0 ($M+H^+$); **HRMS** (**ESI**): Calcd. For $C_{21}H_{32}OF^+$: 319.2432; Found: 319.2427.

(3*S*,8*S*,9*S*,10*R*,13*R*,14*S*,17*R*)-3-Fluoro-10,13-dimethyl-17-((*R*)-6-methylheptan-2-yl)-2,3,4,7,8,9,10,11,12,13,14,15,16,17-tetradecahydro-1*H*-cyclopenta[*a*]phenanthrene (3s)[25]

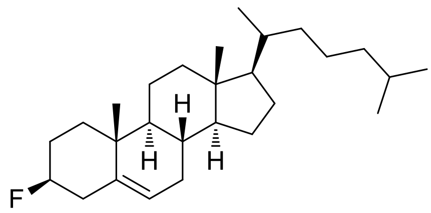

Performed on 0.2 mmol scale, eluted with hexane/CH_2Cl_2 = 100/1 to give **3s** (70.6 mg, 91% yield). White solid. M.p. 91–92 °C. **^{1}H NMR** ($CDCl_3$, 400 MHz) δ = 5.39 (d, *J* = 4.4 Hz, 1H), 4.49–4.28 (dm, *J* = 50.4 Hz, 1H), 2.44 (t, *J* = 7.0 Hz, 2H), 2.03–1.96 (m, 3H), 1.90–1.78 (m, 2H), 1.75–1.63 (m, 1H), 1.61–0.95 (m, 23H), 0.91 (d, *J* = 6.4 Hz, 3H), 0.86 (dd, *J* = 6.8, 1.6 Hz, 6H), 0.68 (s, 3H); **^{19}F NMR** ($CDCl_3$, 376 MHz) δ = –167.8 (dm, *J* = 50.0 Hz, 1F); **^{13}C NMR** ($CDCl_3$, 100 MHz) δ = 139.4 (d, *J* = 12.6 Hz), 123.0, 92.9 (d, *J* = 172.7 Hz), 56.7, 56.2, 50.0 (d, *J* = 2.3 Hz), 42.3, 39.7, 39.5, 39.4 (d, *J* = 19.1 Hz), 36.5 (d, *J* = 1.6 Hz), 36.3 (d, *J* = 11.3 Hz), 36.2, 35.8, 31.92, 31.86, 28.8 (d, *J* = 17.4 Hz), 28.2, 28.0, 24.3, 23.8, 22.8, 22.6, 21.1, 19.3, 18.7, 11.8; **MS** (**EI**, *m/z*, %): 388 (M^+, 1.47), 88 (100.00); **HRMS** (**EI**): Calcd. For $C_{27}H_{45}F$: 388.3505; Found: 388.3504.

(3*R*,5*S*,8*R*,9*S*,10*S*,13*S*,14*S*)-3-Fluoro-10,13-dimethyltetradecahydro-1*H*-cyclopenta[*a*]phenanthren-17(2*H*)-one (3t)[20]

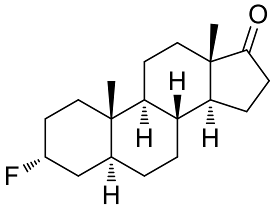

Performed on 0.2 mmol scale, eluted with hexane/Et_2O = 10/1 to give **3t** (42.0 mg, 72% yield). White solid. M.p. 112–115 °C. **^{1}H NMR** ($CDCl_3$, 400 MHz) δ = 4.80 (d, *J* = 48.8 Hz, 1H), 2.42 (dd, *J* = 19.2, 8.5 Hz, 1H), 2.10–2.01 (m, 1H), 1.96–1.84 (m, 2H), 1.81–1.77 (m, 2H), 1.70–1.15 (m, 15H), 1.01 (qd, *J* = 13.0, 4.6 Hz, 1H), 0.85 (s, 3H), 0.80 (s, 3H); **^{19}F NMR** ($CDCl_3$, 376 MHz) δ = –181.2 (m, 1F); **^{13}C NMR** ($CDCl_3$, 100 MHz) δ = 221.2, 89.2 (d, *J* = 165.7 Hz), 54.1, 51.3, 47.7, 39.3, 35.75, 35.73, 34.9, 33.7 (d, *J* = 21.3 Hz), 32.3, 31.4, 30.6, 27.9, 26.9 (d, *J* = 22.2 Hz), 21.6, 19.9, 13.7, 11.0 (d, *J* = 1.5 Hz); **MS** (**ESI**): *m/z* 293.1 ($M+H^+$); **HRMS** (**ESI**): Calcd. For $C_{19}H_{30}OF^+$: 293.2275; Found: 293.2271.

3-(3-Fluoropropyl)pyridine (3h)[26]

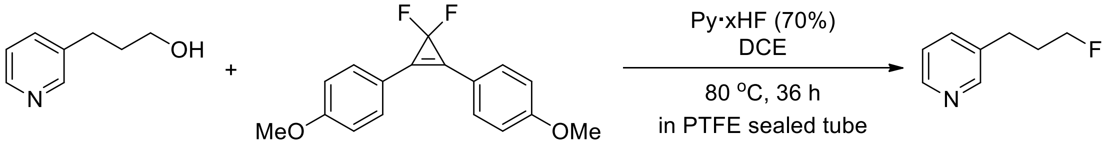

2h **1c** **3h**

To a dry PTFE sealed tube, 3-(pyridin-3-yl)propan-1-ol (**2h**, 68.6 mg, 64 μL, 0.5 mmol, 1.0 equiv.), CpFluor **1c** (432.4 mg, 1.5 mmol, 3.0 equiv.) and solvent DCE (7.0 mL) were sequentially added, then 70% Py•xHF (94.5 mg, ~3.3 mmol HF, 6.6 equiv.) was added. The sealed tube was immersed in an oil bath at 80 °C, and the mixture was stirred for 36 h at 80 °C. After cooled to room temperature, 10 mL of 20% K_2CO_3 aqueous solution was added, extracted with CH_2Cl_2 (30 mL × 3), the organic layer was combined, dried over Na_2SO_4. After remove the solvent, 60 mL ether was added, stirred for about 5 min, the precipitate was filtered, eluting with ether. The filtrate was concentrated under reduced pressure and the residue was purified by flash chromatography on silica gel (eluent: hexane/Et_2O/Et_3N = 30/10/1) to give the 3-(3-fluoropropyl)pyridine as colorless liquid (37.8 mg, 54% yield). **^{1}H NMR** ($CDCl_3$,

400 MHz) δ = 8.46–8.43 (m, 2H), 7.49 (dt, J = 8.0, 1.6 Hz, 1H), 7.20 (dd, J = 7.8, 4.6 Hz, 1H), 4.44 (dt, J = 47.2, 5.8 Hz, 2H), 2.73 (t, J = 8.0 Hz, 2H), 2.05–1.92 (m, 2H); **^{19}F NMR** ($CDCl_3$, 376 MHz) δ = –220.5 (tt, J = 47.2, 25.4 Hz, 1F); **^{13}C NMR** ($CDCl_3$, 100 MHz) δ = 149.9, 147.6, 136.3, 135.8, 123.3, 82.6 (d, J = 165.4 Hz), 31.6 (d, J = 20.5 Hz), 28.5 (d, J = 5.2 Hz); **MS** (**EI**, *m/z*, %): 139 (M^+, 60.16), 92 (100.00); **HRMS** (**EI**): Calcd. For $C_8H_{10}NF$: 139.0797; Found: 139.0793.

2,3-Bis(4-methoxynaphthalen-1-yl)cycloprop-2-enone (5i)

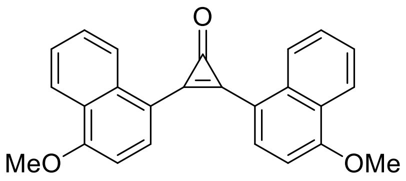

Bright yellow solid. M.p. 261–263 ^{o}C. **IR (KBr):** 3054, 3006, 2939, 2840, 1843, 1573, 1512, 1462, 1431, 1376, 1349, 1329, 1319, 1282, 1264, 1246, 1095, 1086, 805, 769, 598, 502 cm^{-1}; **^{1}H NMR** ($CDCl_3$, 400 MHz) δ = 8.87 (d, J = 8.4 Hz, 2H), 8.31 (d, J = 8.4 Hz, 2H), 8.19 (d, J = 8.4 Hz, 2H), 7.83–7.79 (m, 2H), 7.62–7.58 (m, 2H), 6.94 (d, J = 7.6 Hz, 2H), 4.10 (s, 6H); **^{13}C NMR** ($CDCl_3$, 100 MHz) δ = 159.5, 155.0, 142.3, 134.5, 131.5, 129.0, 126.5, 126.4, 125.6, 122.2, 115.6, 103.5, 55.9; **MS (EI**, *m/z*, %): 338 (100.00), 323 (85.57), 339 (27.06), 324 (22.01), 126 (20.44); **HRMS** (**EI**): Calcd. For $C_{25}H_{18}O_3$: 366.1256; Found: 366.1258.

5. Deoxyfluorination of 1,2- and 1,3-Diols 6a-6p with CpFluor 1a or 1i

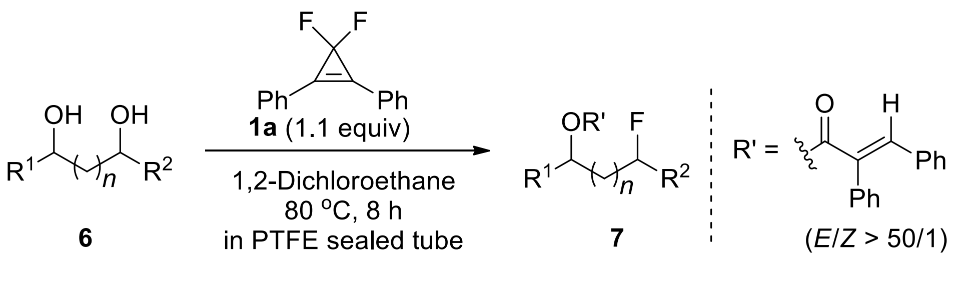

General procedures:

To a dry PTFE sealed tube, 1,2- or 1,3-diols **6** (0.5 mmol), CpFluor **1a** (125.5 mg, 0.55 mmol) and solvent DCE (3.0 mL) were sequentially added. The sealed tube was immersed in an oil bath at 80 °C, and the mixture was stirred for 8 h, after cooled to room temperature, the mixture was concentrated under reduced pressure and the residue was purified by flash chromatography on silica gel to give the corresponding monofluorination product **7**.

(*E*)-2-Fluoroethyl 2,3-diphenylacrylate (7a)

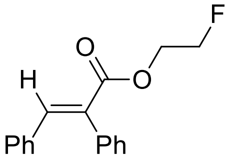

Performed on 0.5 mmol scale, eluted with hexane/Et_2O = 8/1 to give **7a** (133.9 mg, 99% yield). White solid. M.p. 67–68 °C. **IR (KBr):** 3083, 3051, 3024, 2960, 1702, 1624, 1494, 1446, 1383, 1321, 1291, 1238, 1177, 1080, 1042, 974, 940, 876, 845, 786, 777, 761, 753, 710, 689, 601 cm^{-1}; **^{1}H NMR** ($CDCl_3$, 400 MHz) δ = 7.89 (s, 1H), 7.39–7.34 (m, 3H), 7.25–7.13 (m, 5H), 7.05 (d, *J* = 7.6 Hz, 2H), 4.64 (dt, *J* = 47.6, 4.0 Hz, 2H), 4.46 (dt, *J* = 28.8, 4.0 Hz, 2H); **^{19}F NMR** ($CDCl_3$, 376 MHz) δ = –224.5 (tt, *J* = 46.8, 28.6 Hz, 1F); **^{13}C NMR** ($CDCl_3$, 100 MHz) δ = 167.3, 140.8, 135.4, 134.3, 131.9, 130.5, 129.7, 129.0, 128.5, 128.1, 127.8, 81.2 (d, *J* = 170.6 Hz), 63.9 (d, *J* = 19.3 Hz); **MS** (**EI**, *m/z*, %): 270 (M^+, 30.90), 179 (100.00); **HRMS** (**EI**): Calcd. For $C_{17}H_{15}O_2F$: 270.1056; Found: 270.1055.

(*R*,*E*)-1-Fluoropropan-2-yl 2,3-diphenylacrylate (7b1) and (*S*,*E*)-2-fluoropropyl 2,3-diphenylacrylate (7b2)

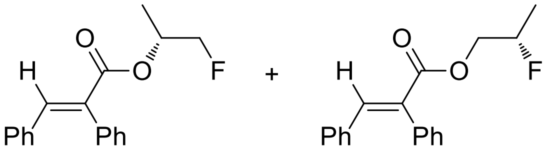

Performed on 0.5 mmol scale, eluted with hexane/Et_2O = 8/1 to give **7b** [141.2 mg, 99% total yield (an inseparable mixture, 1.2/1)]. Colorless liquid. **IR (film):** 3083, 3053, 3025, 3000, 2961, 1699, 1622, 1494, 1446, 1396, 1381, 1318, 1290, 1239, 1213,

1175, 1118, 1075, 1045, 1028, 1012, 974, 957, 936, 828, 777, 756, 710, 690 cm^{-1}; ^{1}**H NMR** ($CDCl_3$, 400 MHz) δ = 7.85 [7.88] (s, 1H), 7.36–7.31 (m, 3H), 7.23–7.11 (m, 5H), 7.05–7.03 (m, 2H), 5.31–5.19 (dm, *J* = 20.0 Hz, 1H), [4.95–4.76 (dm, *J* = 48.8 Hz, 1H)], 4.52–4.19 (m, 2H), [1.30 (dd, *J* = 23.2, 6.4 Hz, 3H)], 1.29 (dd, *J* = 6.8, 1.2 Hz, 3H); ^{19}**F NMR** ($CDCl_3$, 376 MHz) δ = –228.2 (td, *J* = 47.3, 19.9 Hz, 1F) [–180.5 (m, 1F)]; ^{13}**C NMR** ($CDCl_3$, 100 MHz) δ = 167.0 [167.3], 140.5 [140.8], 135.6 [135.5], 134.4 [134.5], 132.4 [132.0], 130.5 [130.6], 129.74 [129.67], 129.0 [129.1], 128.4 [128.5], 128.08 [128.11], 127.7 [127.8], 84.4 (d, *J* = 173.4 Hz) [87.7 (d, *J* = 169.6 Hz)], 69.7 (d, *J* = 19.8 Hz) [67.1 (d, *J* = 21.5 Hz)], 15.1 (d, *J* = 7.0 Hz) [17.1 (d, *J* = 22.0 Hz)]; **MS** (**EI**, *m/z*, %): 284 (M^+, 35.16), 179 (100.00); **HRMS** (**EI**): Calcd. For $C_{18}H_{17}O_2F$: 284.1213; Found: 284.1216, 284.1210.

(*E*)-1-Fluorooctan-2-yl 2,3-diphenylacrylate (7c1) and (*E*)-2-fluorooctyl 2,3-diphenylacrylate (7c2)

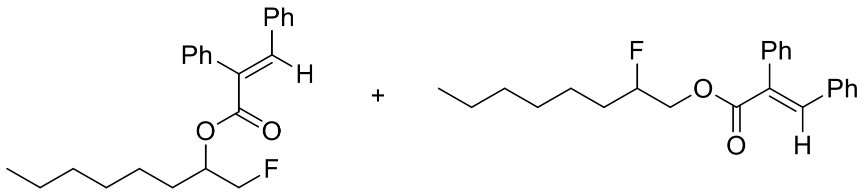

Performed on 0.5 mmol scale, eluted with hexane/Et_2O = 8/1 to give **7c** [126.7 mg, 71% total yield (an inseparable mixture, 1.7/1)]. Colorless liquid. **IR (film):** 3057, 3026, 2955, 2929, 2858, 1713, 1625, 1493, 1448, 1380, 1240, 1207, 1171, 1078, 1033, 776, 710, 692 cm^{-1}; ^{1}**H NMR** ($CDCl_3$, 400 MHz) δ = 7.85 [7.88] (s, 1H), 7.37–7.31 (m, 3H), 7.23–7.11 (m, 5H), 7.05 (d, *J* = 7.6 Hz, 2H), 5.23–5.12 (dm, *J* = 21.2 Hz, 1H), [4.77–4.59 (dm, *J* = 49.6 Hz, 1H)], 4.57–4.21 (m, 2H), 1.70–1.27 (m, 10H), 0.89–0.86 (m, 3H); ^{19}**F NMR** ($CDCl_3$, 376 MHz) δ = –230.0 (td, *J* = 47.4, 21.1 Hz, 1F) [–186.7 (m, 1F)]; ^{13}**C NMR** ($CDCl_3$, 100 MHz) δ = 167.2 [167.4], 140.5 [140.8], 135.7 [135.6], 134.5 [134.4], 132.5 [132.1], 130.56 [130.60], 129.7, 129.0 [129.1], 128.47 [128.52], 128.11 [128.13], 127.7 [127.8], 83.5 (d, *J* = 172.2 Hz) [91.2 (d, *J* = 171.7 Hz)], 73.1 (d, *J* = 19.4 Hz) [66.3 (d, *J* = 22.8 Hz)], 31.5, [31.3 (d, *J* = 20.3 Hz)], 29.5 (d, *J* = 6.4 Hz), 28.9, 25.0, [24.6 (d, *J* = 4.6 Hz)], 22.4 [22.5], 14.0; **MS** (**EI**,

m/z, %): 354 (M^+, 22.42), 179 (100.00); **HRMS** (**EI**): Calcd. For $C_{23}H_{27}O_2F$: 354.1995; Found: 354.1997, 354.2000.

(*E*)-1-(Allyloxy)-3-fluoropropan-2-yl 2,3-diphenylacrylate (7d1)

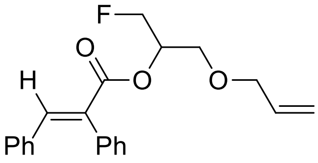

Performed on 0.5 mmol scale, eluted with hexane/Et_2O = 10/1 to give **7d1** (149.4 mg, 88% yield). Colorless liquid. **IR (film):** 3057, 3025, 2917, 1713, 1622, 1448, 1240, 1208, 1171, 1100, 1030, 1005, 930, 776, 711, 693 cm^{-1}; ^{1}**H NMR** ($CDCl_3$, 400 MHz) δ = 7.87 (s, 1H), 7.37–7.33 (m, 3H), 7.24–7.14 (m, 5H), 7.06–7.04 (m, 2H), 5.90–5.80 (m, 1H), 5.34–5.17 (m, 3H), 4.72–4.53 (dm, *J* = 47.2 Hz, 2H), 3.98 (d, *J* = 5.2 Hz, 2H), 3.66 (dd, *J* = 5.8, 1.4 Hz, 2H); ^{19}**F NMR** ($CDCl_3$, 376 MHz) δ = –233.0 (td, *J* = 47.2, 21.7 Hz, 1F); ^{13}**C NMR** ($CDCl_3$, 100 MHz) δ = 166.8, 140.9, 135.4, 134.4, 134.1, 132.0, 130.5, 129.7, 129.0, 128.4, 128.1, 127.7, 117.1, 81.5 (d, *J* = 170.9 Hz), 72.1, 71.8 (d, *J* = 19.3 Hz), 67.1 (d, *J* = 6.8 Hz); **MS** (**EI**, *m/z*, %): 340 (M^+, 1.20), 179 (100.00); **HRMS** (**EI**): Calcd. For $C_{21}H_{21}O_3F$: 340.1475; Found: 340.1476.

(*E*)-3-(allyloxy)-2-fluoropropyl 2,3-diphenylacrylate (7d2)

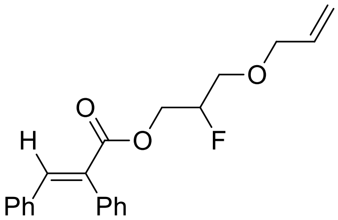

Performed on 0.5 mmol scale, eluted with hexane/Et_2O = 10/1 to give **7d2** (16.5 mg, 10% yield). Colorless liquid. **IR (film):** 3057, 3025, 2951, 2861, 1714, 1624, 1493, 1448, 1239, 1207, 1172, 1106, 1079, 1042, 1025, 931, 775, 712, 693 cm^{-1}; ^{1}**H NMR** ($CDCl_3$, 400 MHz) δ = 7.87 (s, 1H), 7.37–7.35 (m, 3H), 7.23–7.14 (m, 5H), 7.06 (d, *J* = 7.2 Hz, 2H), 5.91–5.82 (m, 1H), 5.29–5.18 (m, 2H), 4.92–4.75 (dm, *J* = 48.0 Hz, 1H), 4.52–4.34 (m, 2H), 3.99 (d, *J* = 5.6 Hz, 2H), 3.58 (dd, *J* = 21.4, 4.6 Hz, 2H); ^{19}**F NMR** ($CDCl_3$, 376 MHz) δ = –194.0 (dm, *J* = 48.5 Hz, 1F); ^{13}**C NMR** ($CDCl_3$, 100

MHz) δ = 167.3, 141.0, 135.6, 134.4, 134.1, 132.0, 130.7, 129.7, 129.2, 128.6, 128.2, 127.9, 117.6, 89.8 (d, *J* = 173.8 Hz), 72.5, 68.7 (d, *J* = 23.0 Hz), 63.9 (d, *J* = 23.2 Hz); **MS** (**EI**, *m/z*, %): 340 (M^+, 15.13), 179 (100.00); **HRMS** (**EI**): Calcd. For $C_{21}H_{21}O_3F$: 340.1475; Found: 340.1471.

(*E*)-1-Fluoro-3-phenoxypropan-2-yl 2,3-diphenylacrylate (7e1)

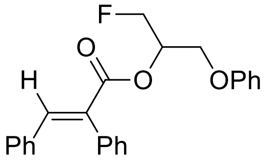

Performed on 0.5 mmol scale, eluted with hexane/Et_2O = 8/1 to give **7e1** (162.1 mg, 86% yield). White solid. M.p. 58–60 ^oC. **IR (KBr):** 3058, 2958, 1712, 1621, 1599, 1588, 1496, 1448, 1320, 1292, 1235, 1169, 1103, 1079, 1050, 1005, 776, 755, 711, 691 cm^{-1}; ^{1}**H NMR** ($CDCl_3$, 400 MHz) δ = 7.87 (s, 1H), 7.36–7.33 (m, 3H), 7.31–7.26 (m, 2H), 7.23–7.13 (m, 5H), 7.05 (d, *J* = 7.2 Hz, 2H), 6.98 (t, *J* = 7.2 Hz, 1H), 6.94–6.91 (m, 2H), 5.52–5.42 (dm, *J* = 22.4 Hz, 1H), 4.73 (dd, *J* = 47.0, 3.8 Hz, 2H), 4.21 (dd, *J* = 5.4, 1.0 Hz, 2H); ^{19}**F NMR** ($CDCl_3$, 376 MHz) δ = –233.2 (td, *J* = 47.1, 21.8 Hz, 1F); ^{13}**C NMR** ($CDCl_3$, 100 MHz) δ = 166.9, 158.2, 141.2, 135.3, 134.3, 131.8, 130.6, 129.8, 129.5, 129.1, 128.5, 128.1, 127.8, 121.3, 114.6, 81.2 (d, *J* = 171.8 Hz), 71.2 (d, *J* = 19.7 Hz), 64.9 (d, *J* = 6.8 Hz); **MS** (**EI**, *m/z*, %): 376 (M^+, 4.84), 283 (100.00), 179 (95.62); **HRMS** (**EI**): Calcd. For $C_{24}H_{21}O_3F$: 376.1475; Found: 376.1473.

(*E*)-2-Fluoro-3-phenoxypropyl 2,3-diphenylacrylate (7e2)

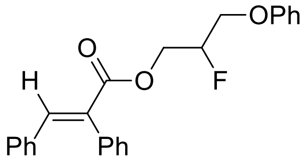

Performed on 0.5 mmol scale, eluted with hexane/Et_2O = 8/1 to give **7e2** (14.2 mg, 8% yield). White solid. M.p. 69–70 ^oC. **IR (KBr):** 3058, 2953, 1713, 1624, 1600, 1589, 1496, 1448, 1291, 1239, 1207, 1171, 1106, 1079, 1053, 1026, 775, 755, 712, 692 cm^{-1}; ^{1}**H NMR** ($CDCl_3$, 400 MHz) δ = 7.88 (s, 1H), 7.36–7.34 (m, 3H),

7.31–7.25 (m, 2H), 7.23–7.14 (m, 5H), 7.05 (d, *J* = 6.8 Hz, 2H), 6.98 (t, *J* = 7.4 Hz, 1H), 6.86–6.84 (m, 2H), 5.11–4.94 (dm, *J* = 47.6 Hz, 1H), 4.64–4.44 (m, 2H), 4.08 (dd, *J* = 20.0, 5.2 Hz, 2H); **^{19}F NMR** ($CDCl_3$, 376 MHz) δ = –194.6 (m, 1F); **^{13}C NMR** ($CDCl_3$, 100 MHz) δ = 167.3, 158.1, 141.2, 135.6, 134.3, 131.8, 130.7, 129.7, 129.5, 129.3, 128.7, 128.2, 127.9, 121.4, 114.6, 88.9 (d, *J* = 175.5 Hz), 66.5 (d, *J* = 24.7 Hz), 63.7 (d, *J* = 23.6 Hz); **MS** (**EI**, *m/z*, %): 376 (M^+, 11.72), 179 (100.00); **HRMS** (**EI**): Calcd. For $C_{24}H_{21}O_3F$: 376.1475; Found: 376.1478.

(*E*)-1-Fluoro-3-(4-methoxyphenoxy)propan-2-yl 2,3-diphenylacrylate (7f1)

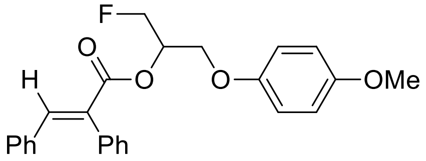

Performed on 0.5 mmol scale, eluted with hexane/Et_2O = 6/1 to give **7f1** (183.7 mg, 90% yield). Colorless liquid. **IR (film):** 3056, 3025, 2998, 2955, 2834, 1712, 1620, 1508, 1464, 1447, 1320, 1290, 1230, 1170, 1103, 1036, 1005, 825, 777, 750, 712, 693 cm^{-1}; **^{1}H NMR** ($CDCl_3$, 400 MHz) δ = 7.86 (s, 1H), 7.36–7.34 (m, 3H), 7.23–7.14 (m, 5H), 7.05 (d, *J* = 6.8 Hz, 2H), 6.87–6.81 (m, 4H), 5.49–5.39 (dm, *J* = 22.0 Hz, 1H), 4.72 (dd, *J* = 47.0, 4.2 Hz, 2H), 4.17 (dd, *J* = 5.6, 0.8 Hz, 2H), 3.76 (s, 3H); **^{19}F NMR** ($CDCl_3$, 376 MHz) δ = –233.1 (td, *J* = 47.1, 21.7 Hz, 1F); **^{13}C NMR** ($CDCl_3$, 100 MHz) δ = 166.8, 154.2, 152.3, 141.1, 135.3, 134.3, 131.8, 130.6, 129.7, 129.1, 128.5, 128.1, 127.8, 115.7, 114.6, 81.3 (d, *J* = 172.2 Hz), 71.3 (d, *J* = 18.7 Hz), 65.8 (d, *J* = 6.7 Hz), 55.5; **MS** (**EI**, *m/z*, %): 406 (M^+, 1.05), 283 (100.00), 179 (69.12); **HRMS** (**EI**): Calcd. For $C_{25}H_{23}O_4F$: 406.1580; Found: 406.1583.

(*E*)-2-Fluoro-3-(4-methoxyphenoxy)propyl 2,3-diphenylacrylate (7f2)

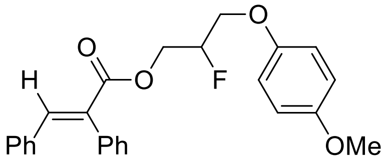

Performed on 0.5 mmol scale, eluted with hexane/Et_2O = 6/1 to give **7f2** (18.1 mg, 9% yield). White solid. M.p. 86–87 ^{o}C. **IR (KBr):** 3082, 3054, 3005, 2953, 2937,

2837, 1719, 1625, 1514, 1454, 1444, 1269, 1243, 1208, 1172, 1094, 1034, 1019, 871, 834, 791, 747, 713, 696, 689, 503, 484 cm^{-1}; **^{1}H NMR** ($CDCl_3$, 400 MHz) δ = 7.87 (s, 1H), 7.36–7.34 (m, 3H), 7.23–7.13 (m, 5H), 7.06–7.04 (m, 2H), 6.84–6.78 (m, 4H), 5.08–4.91 (dm, *J* = 48.0 Hz, 1H), 4.62–4.42 (m, 2H), 4.03 (dd, *J* = 20.0, 4.8 Hz, 2H), 3.77 (s, 3H); **^{19}F NMR** ($CDCl_3$, 376 MHz) δ = –194.6 (m, 1F); **^{13}C NMR** ($CDCl_3$, 100 MHz) δ = 167.3, 154.3, 152.3, 141.2, 135.6, 134.3, 131.8, 130.7, 129.7, 129.3, 128.7, 128.2, 127.9, 115.7, 114.6, 88.9 (d, *J* = 175.3 Hz), 67.4 (d, *J* = 24.8 Hz), 63.7 (d, *J* = 23.6 Hz), 55.7; **MS** (**EI**, *m/z*, %): 406 (M^+, 6.00), 283 (100.00), 179 (84.37); **HRMS** (**EI**): Calcd. For $C_{25}H_{23}O_4F$: 406.1580; Found: 406.1582.

(*R*,*E*)-1-Chloro-3-fluoropropan-2-yl 2,3-diphenylacrylate (7g1) and (*S*,*E*)-3-chloro-2-fluoropropyl 2,3-diphenylacrylate (7g2)

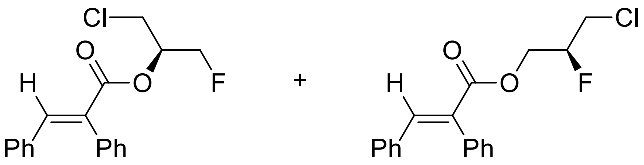

Performed on 0.5 mmol scale, eluted with hexane/Et_2O = 8/1 to give **7g** [154.8 mg, 97% total yield (an inseparable mixture, 15.8/1)]. White solid. **IR (KBr):** 3081, 3061, 3022, 3003, 2963, 1717, 1629, 1492, 1445, 1430, 1374, 1341, 1314, 1249, 1202, 1171, 1131, 1063, 1022, 986, 934, 896, 778, 764, 752, 706, 690, 626, 612, 575, 462 cm^{-1}; **^{1}H NMR** ($CDCl_3$, 400 MHz) δ = 7.89 (s, 1H), 7.35–7.31 (m, 3H), 7.23–7.11 (m, 5H), 7.05 (d, *J* = 7.2 Hz, 2H), 5.34–5.24 (dm, *J* = 19.6 Hz, 1H), 4.71–4.51 (dm, *J* = 46.8 Hz, 2H), 3.74 (ddd, *J* = 11.6, 6.1, 0.9 Hz, 1H), 3.67 (ddd, *J* = 11.6, 5.4, 1.1 Hz, 1H); **^{19}F NMR** ($CDCl_3$, 376 MHz) δ = –233.9 (td, *J* = 46.8, 20.1 Hz, 1F) [–188.2 (m, 1F)]; **^{13}C NMR** ($CDCl_3$, 100 MHz) δ = 166.5, 141.5, 135.1, 134.2, 131.5, 130.6, 129.7, 129.3, 128.5, 128.1, 127.9, 80.8 (d, *J* = 173.2 Hz), 71.8 (d, *J* = 21.0 Hz), 40.9 (d, *J* = 6.9 Hz); **MS** (**EI**, *m/z*, %): 318 (M^+, 12.11), 179 (100.00); **HRMS** (**EI**): Calcd. For $C_{18}H_{16}O_2FCl$: 318.0823; Found: 318.0821, 318.0817.

(*E*)-4-Fluorohexa-1,5-dien-3-yl 2,3-diphenylacrylate (7h)

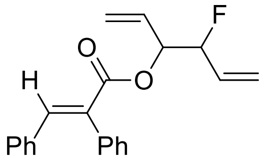

Prepared from a 1/1 mixture of *meso*- and *dl*-hexa-1,5-diene-3,4-diol (**6h**) on 0.5 mmol scale, eluted with hexane/Et_2O = 8/1 to gvie **7h** [117.7 mg,. 73% yield (a 1/1 mixture of 2 pairs of inseparable diastereoisomers)]. Colorless liquid. **IR (film):** 3084, 3057, 3026, 2924, 1716, 1625, 1493, 1447, 1237, 1207, 1169, 1035, 1014, 987, 938, 777, 710, 692 cm^{-1}; ^{1}**H NMR** ($CDCl_3$, 400 MHz) δ = 7.88 (s, 1H), 7.36–7.33 (m, 3H), 7.23–7.11 (m, 5H), 7.05 (d, *J* = 7.6 Hz, 2H), 5.86–5.71 (m, 2H), 5.59–5.53 (m, 1H), 5.41–5.28 (m, 4H), 4.95 (dm, *J* = 48.0 Hz, 1H) [4.91 (dt, *J* = 47.2, 5.8 Hz, 1H)]; ^{19}**F NMR** ($CDCl_3$, 376 MHz) δ = –187.6 (dt, *J* = 47.8, 15.4 Hz, 1F) [–187.9 (ddd, *J* = 47.4, 17.8, 14.4 Hz, 1F)]; ^{13}**C NMR** ($CDCl_3$, 100 MHz) δ = 166.49 [166.46], 140.83 [140.88], 135.54 [135.56], 134.39 [134.37], 132.17 [132.14], 131.5 [131.8] (d, *J* = 19.9 Hz), 130.7 [131.2] (d, *J* = 5.9 Hz), 130.6, 129.7, 129.13 [129.11], 128.5, 128.1, 127.7, 119.6 [120.0] (d, *J* = 12.4 Hz), 119.5 [119.8], 92.9 [92.3] (d, *J* = 176.2 Hz), 75.5 [75.2] (d, *J* = 52.4 Hz); **MS** (**EI**, *m/z*, %): 322 (M^+, 9.42), 179 (100.00); **HRMS** (**EI**): Calcd. For $C_{21}H_{19}O_2F$: 322.1369; Found: 322.1366.

(*E*)-(2*R*,3*S*)-3-fluorobutan-2-yl 2,3-diphenylacrylate (7i)

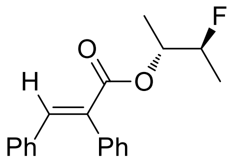

Prepared from (*R*,*R*)-butane-2,3-diol (**6i**) on 0.5 mmol scale, eluted with hexane/Et_2O = 8/1 to give **7i** (148.6 mg, 99% yield). Colorless liquid. **IR (film):** 3057, 3026, 2987, 2941, 1711, 1625, 1448, 1385, 1244, 1208, 1172, 1105, 1082, 1013, 1001, 774, 710, 692 cm^{-1}; ^{1}**H NMR** ($CDCl_3$, 400 MHz) δ = 7.84 (s, 1H), 7.38–7.32 (m, 3H), 7.23–7.14 (m, 5H), 7.06 (d, *J* = 7.6 Hz, 2H), 5.13–5.03 (m, 1H), 4.75–4.57 (dm, *J* = 48.0 Hz, 1H), 1.28 (d, *J* = 6.8 Hz, 3H), 1.25 (dd, *J* = 23.6, 6.8 Hz, 3H); ^{19}**F NMR** ($CDCl_3$, 376 MHz) δ = –184.2 (m, 1F); ^{13}**C NMR** ($CDCl_3$, 100 MHz) δ = 166.9, 140.4, 135.7, 134.5, 132.5, 130.5, 129.7, 129.0, 128.4, 128.1, 127.7, 90.7 (d, *J* = 171.7

Hz), 72.5 (d, J = 22.8 Hz), 16.0 (d, J = 22.6 Hz), 14.4 (d, J = 7.2 Hz); **MS** (**EI**, *m/z*, %): 298 (M^+, 31.82), 179 (100.00); **HRMS** (**EI**): Calcd. For $C_{19}H_{19}O_2F$: 298.1369; Found: 298.1371.

(*E*)-(*trans*)-2-Fluorocyclohexyl 2,3-diphenylacrylate (7j)

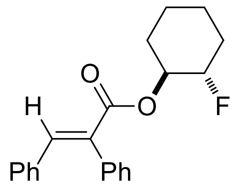

Prepared from *syn*-cyclohexane-1,2-diol (**6j**) on 0.5 mmol scale, eluted with hexane/Et_2O = 8/1 to give **7j** (151.9 mg, 94% yield). White solid. M.p. 72−73 °C. **IR (KBr):** 3064, 3030, 2930, 2864, 1709, 1622, 1492, 1448, 1359, 1278, 1250, 1209, 1173, 1053, 1038, 1026, 1001, 992, 923, 784, 774, 759, 750, 712, 696, 687 cm^{-1}; **^{1}H NMR** ($CDCl_3$, 400 MHz) δ = 7.85 (s, 1H), 7.36−7.31 (m, 3H), 7.23−7.10 (m, 5H), 7.05−7.03 (m, 2H), 5.06−4.98 (m, 1H), 4.52−4.34 (dm, J = 50.0 Hz, 1H), 2.09−1.97 (m, 2H), 1.72−1.69 (m, 1H), 1.64−1.52 (m, 2H), 1.43−1.21 (m, 3H); **^{19}F NMR** ($CDCl_3$, 376 MHz) δ = −181.8 (d, J = 34.6 Hz, 1F); **^{13}C NMR** ($CDCl_3$, 100 MHz) δ = 166.9, 140.3, 135.7, 134.5, 132.5, 130.5, 129.7, 128.9, 128.4, 128.1, 127.6, 91.5 (d, J = 177.2 Hz), 74.3 (d, J = 20.6 Hz), 30.0 (d, J = 18.8 Hz), 29.0 (d, J = 4.7 Hz), 22.7 (d, J = 1.9 Hz), 22.4 (d, J = 9.2 Hz); **MS** (**EI**, *m/z*, %): 324 (M^+, 5.42), 179 (100.00); **HRMS** (**EI**): Calcd. For $C_{21}H_{21}O_2F$: 324.1526; Found: 324.1525.

(*E*)-3-Fluoropropyl 2,3-diphenylacrylate (7k)

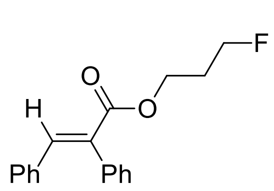

Performed on 0.5 mmol scale, eluted with hexane/Et_2O = 8/1 to give **7k** (134.7 mg, 95% yield). Colorless liquid. **IR (film):** 3057, 3025, 2970, 2906, 1712, 1625, 1493, 1448, 1243, 1207, 1171, 1048, 777, 711, 692 cm^{-1}; **^{1}H NMR** ($CDCl_3$, 400 MHz) δ = 7.84 (s, 1H), 7.39−7.34 (m, 3H), 7.22−7.13 (m, 5H), 7.06−7.04 (m, 2H), 4.46 (dt, J = 46.8, 5.8 Hz, 2H), 4.33 (t, J = 6.4 Hz, 2H), 2.04 (dm, J = 25.2 Hz, 2H); **^{19}F NMR**

(CDCl$_3$, 376 MHz) δ = –222.2 (tt, *J* = 46.6, 25.6 Hz, 1F); ^{13}**C NMR** (CDCl$_3$, 100 MHz) δ = 167.5, 140.4, 135.7, 134.4, 132.4, 130.5, 129.6, 129.0, 128.5, 128.1, 127.7, 80.5 (d, *J* = 165.0 Hz), 60.9 (d, *J* = 5.0 Hz), 29.6 (d, *J* = 20.2 Hz); **MS** (**EI**, *m/z*, %): 284 (M^+, 24.17), 179 (100.00); **HRMS** (**EI**): Calcd. For $C_{18}H_{17}O_2F$: 284.1213; Found: 284.1215.

(*E*)-3-Fluoro-2,2-dimethylpropyl 2,3-diphenylacrylate (7l)

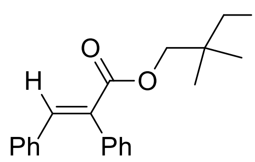

Performed on 0.5 mmol scale, eluted with hexane/Et_2O = 5/1 to give **7l** (153.1 mg, 98% yield). White solid. M.p. 60–63 ^{o}C. **IR (KBr):** 3081, 3054, 2967, 2892, 1712, 1626, 1597, 1493, 1475, 1447, 1370, 1292, 1250, 1210, 1173, 1078, 1020, 1000, 974, 783, 766, 709, 693 cm^{-1}; ^{1}**H NMR** (CDCl$_3$, 400 MHz) δ = 7.86 (s, 1H), 7.37–7.32 (m, 3H), 7.21–7.11 (m, 5H), 7.06 (d, *J* = 7.2 Hz, 2H), 4.00 (s, 2H), 3.98 (d, *J* = 47.2 Hz, 2H), 0.86 (d, *J* = 1.2 Hz, 6H); ^{19}**F NMR** (CDCl$_3$, 376 MHz) δ = –226.5 (t, *J* = 47.8 Hz, 1F); ^{13}**C NMR** (CDCl$_3$, 100 MHz) δ = 167.4, 140.3, 136.0, 134.4, 132.4, 130.6, 129.4, 129.0, 128.5, 128.1, 127.6, 87.8 (d, *J* = 173.0 Hz), 69.0 (d, *J* = 5.0 Hz), 35.6 (d, *J* = 17.2 Hz), 20.4 (d, *J* = 5.3 Hz); **MS** (**EI**, *m/z*, %): 312 (M^+, 30.53), 179 (100.00); **HRMS** (**EI**): Calcd. For $C_{20}H_{21}O_2F$: 312.1526; Found: 312.1530.

(*E*)-3-Bromo-2-(bromomethyl)-2-(fluoromethyl)propyl 2,3-diphenylacrylate (7m)

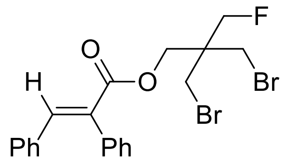

Performed on 0.5 mmol scale, eluted with hexane/Et_2O = 8/1 to give **7m** (228.3 mg, 97% yield). White solid. M.p. 74–75 ^{o}C. **IR (KBr):** 3083, 3028, 2980, 2961, 1716, 1628, 1596, 1464, 1447, 1266, 1242, 1206, 1170, 1065, 1031, 1016, 973, 781, 774, 760, 711, 700, 689, 610 cm^{-1}; ^{1}**H NMR** (CDCl$_3$, 400 MHz) δ = 7.87 (s, 1H), 7.43–7.37 (m, 3H), 7.24–7.16 (m, 5H), 7.09 (d, *J* = 7.2 Hz, 2H), 4.27 (d, *J* = 46.8 Hz,

2H), 4.23 (d, *J* = 1.6 Hz, 2H), 3.26 (s, 4H); **^{19}F NMR** ($CDCl_3$, 376 MHz) δ = –233.8 (t, *J* = 46.8 Hz, 1F); **^{13}C NMR** ($CDCl_3$, 100 MHz) δ = 166.7, 141.2, 135.9, 134.1, 131.5, 130.7, 129.4, 129.2, 128.7, 128.2, 127.9, 81.9 (d, *J* = 176.4 Hz), 63.2 (d, *J* = 4.7 Hz), 43.5 (d, *J* = 16.8 Hz), 32.2 (d, *J* = 5.4 Hz); **MS** (**EI**, *m/z*, %): 468 (M^+, 4.45), 179 (100.00); **HRMS** (**EI**): Calcd. For $C_{20}H_{19}O_2FBr_2$: 467.9736; Found: 467.9740.

(*E*)-3-Fluoro-2-methyl-2-nitropropyl 2,3-diphenylacrylate (7n)

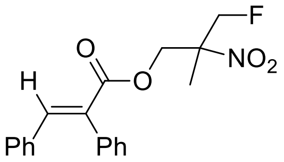

Performed on 0.5 mmol scale, eluted with hexane/Et_2O = 4/1 to give **7n** (48.2 mg, 28% yield). White solid. M.p. 77–80 °C. **IR (KBr):** 3082, 3055, 3024, 2968, 2892, 1716, 1622, 1551, 1493, 1447, 1352, 1313, 1292, 1239, 1213, 1172, 1077, 1026, 776, 759, 711, 690 cm^{-1}; **^{1}H NMR** ($CDCl_3$, 400 MHz) δ = 7.84 (s, 1H), 7.40–7.35 (m, 3H), 7.25–7.14 (m, 5H), 7.07 (d, *J* = 7.2 Hz, 2H), 4.61 (dd, *J* = 11.0, 2.0 Hz, 1H), 4.57 (d, *J* = 46.0 Hz, 2H), 4.54 (dd, *J* = 11.6, 1.2 Hz, 1H), 1.53 (d, *J* = 2.0 Hz, 3H); **^{19}F NMR** ($CDCl_3$, 376 MHz) δ = –228.4 (t, *J* = 46.4 Hz, 1F); **^{13}C NMR** ($CDCl_3$, 100 MHz) δ = 166.4, 141.8, 135.3, 134.0, 131.1, 130.8, 129.5, 129.4, 128.7, 128.3, 128.0, 87.3 (d, *J* = 18.9 Hz), 82.5 (d, *J* = 178.4 Hz), 64.4 (d, *J* = 4.6 Hz), 18.1 (d, *J* = 4.1 Hz); **MS** (**EI**, *m/z*, %): 343 (M^+, 4.55), 179 (100.00); **HRMS** (**EI**): Calcd. For $C_{19}H_{18}NO_4F$: 343.1220; Found: 343.1218.

(*S*,*E*)-4-Fluorobutan-2-yl 2,3-diphenylacrylate (7o1) and (*R*,*E*)-3-fluorobutyl 2,3-diphenylacrylate (7o2)

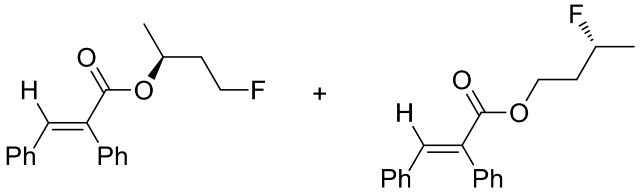

7o1 **7o2**

Performed on 0.5 mmol scale, eluted with hexane/Et_2O = 8/1 to give **7o** [139.8 mg, 94% total yield (an inseparable mixture, 1/5, ee 94.0% & ee 93.2%)]. The

enantiomeric excess was determined by chiral HPLC, IC (4.6 mm × 250 mm, 5 μm), Hexane/IPA = 98/2 (v/v), 0.4 mL/min, λ = 214 nm, t_{R7o1} (major) = 22.377 min, t_{R7o1} (minor) = 20.127 min; t_{R7o2} (major) = 29.677 min, t_{R7o2} (minor) = 31.127 min. Colorless liquid. **IR (film):** 3057, 3025, 2979, 1710, 1625, 1448, 1385, 1246, 1207, 1172, 778, 711, 692 cm^{-1}; **^{1}H NMR** ($CDCl_3$, 400 MHz) δ = 7.82 [7.84] (s, 1H), 7.36–7.33 (m, 3H), 7.21–7.10 (m, 5H), 7.04 (d, *J* = 8.0 Hz, 2H), 5.22–5.14 (m, 1H) [4.78–4.58 (dm, *J* = 48.8 Hz, 1H)], 4.49–4.26 (m, 2H), 2.03–1.79 (m, 2H), 1.32 (d, *J* = 6.4 Hz, 3H) [1.29 (dd, *J* = 23.8, 6.2 Hz, 3H)]; **^{19}F NMR** ($CDCl_3$, 376 MHz) δ = –220.4 (m, 1F) [–175.2 (m, 1F)]; **^{13}C NMR** ($CDCl_3$, 100 MHz) δ = 167.1 [167.5], 140.0 [140.3], 135.8 [135.7], 134.5 [134.4], 132.8 [132.4], 130.48 [130.51], 129.6, 128.9 [129.0], 128.4 [128.5], 128.07 [128.10], 127.6 [127.7], 80.4 (d, *J* = 164.3 Hz) [87.7 (d, *J* = 164.2 Hz)], 68.4 (d, *J* = 5.2 Hz) [61.2 (d, *J* = 5.4 Hz)], 36.4 (d, *J* = 18.6 Hz) [35.8 (d, *J* = 21.0 Hz)], 20.0 [20.9 (d, *J* = 22.5 Hz)]; **MS** (**EI**, *m/z*, %): 298 (M^+, 34.93), 179 (100.00); **HRMS** (**EI**): Calcd. For $C_{19}H_{19}O_2F$: 298.1369; Found: 298.1368.

(*E*)-(3a*R*,5*S*,6*R*,6a*R*)-5-(Fluoromethyl)-2,2-dimethyltetrahydrofuro[2,3-*d*][1,3]dioxol-6-yl 2,3-diphenylacrylate (7p)

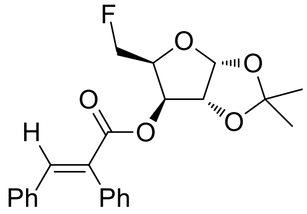

Performed on 0.5 mmol scale, eluted with hexane/Et_2O = 8/1 to give **7p** (192.6 mg, 97% yield). Light yellow liquid. **IR (film):** 3057, 3026, 2990, 1716, 1624, 1448, 1384, 1376, 1240, 1207, 1165, 1074, 1054, 1014, 737, 711, 693 cm^{-1}; **^{1}H NMR** ($CDCl_3$, 400 MHz) δ = 7.83 (s, 1H), 7.38–7.37 (m, 3H), 7.25–7.15 (m, 5H), 7.05 (d, *J* = 7.2 Hz, 2H), 5.89 (d, *J* = 4.4 Hz, 1H), 5.33 (d, *J* = 3.2 Hz, 1H), 4.62–4.54 (m, 2H), 4.51–4.29 (dm, *J* = 46.4 Hz, 2H), 1.53 (s, 3H), 1.32 (s, 3H); **^{19}F NMR** ($CDCl_3$, 376 MHz) δ = –229.9 (td, *J* = 47.0, 15.7 Hz, 1F); **^{13}C NMR** ($CDCl_3$, 100 MHz) δ = 166.5, 141.6, 135.2, 134.0, 131.4, 130.7, 129.43, 129.38, 128.7, 128.2, 128.0, 112.3, 104.8, 83.2,

80.5 (d, J = 168.2 Hz), 77.4 (d, J = 21.5 Hz), 76.8 (d, J = 5.6 Hz), 26.7, 26.2; **MS** (**EI**, *m/z*, %): 398 (M^+, 12.38), 179 (100.00); **HRMS** (**EI**): Calcd. For $C_{23}H_{23}O_5F$: 398.1530; Found: 398.1528.

(*E*)-3-Fluoro-2,2-dimethylpropyl 2,3-bis(4-methoxynaphthalen-1-yl)acrylate (SI-10)

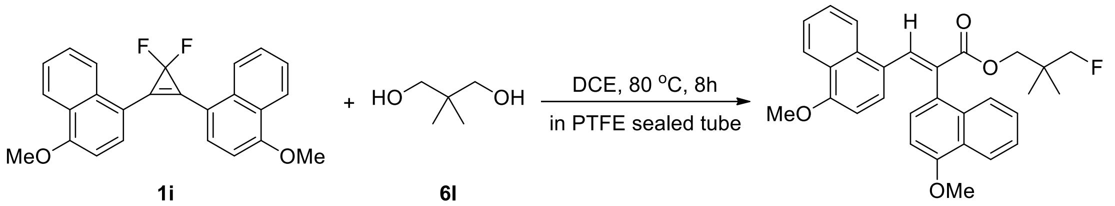

1i (1.1 equiv.) **6l** (1.0 equiv.) **SI-10**, 82%

To a dry PTFE sealed tube, 1,3-diol **6l** (20.8 mg, 0.2 mmol, 1.0 equiv.), CpFluor **1i** (85.5 mg, 0.22 mmol, 1.1 equiv.) and solvent DCE (1.5 mL) were sequentially added. The sealed tube was immersed in an oil bath at 80 ^{o}C, and the mixture was stirred for 8 h, after cooled to room temperature, the mixture was concentrated under reduced pressure and the residue was purified by flash chromatography on silica gel (petroleum ether/ethyl acetate 15:1 v/v) to give the product **SI-10** as yellow solid (77.5 mg, 82% yield). M.p. 65–67 ^{o}C. **IR (film):** 3066, 2966, 2894, 2839, 1709, 1621, 1583, 1511, 1462, 1442, 1423, 1401, 1375, 1329, 1284, 1267, 1242, 1209, 1149, 1095, 1067, 1045, 1026, 997, 969, 919, 818, 793, 764, 737, 713, 601 cm^{-1}; ^{1}**H NMR** ($CDCl_3$, 400 MHz) δ = 8.81 (s, 1H), 8.34–8.31 (m, 1H), 8.27 (d, J = 2.4 Hz, 1H), 8.24 (d, J = 2.0 Hz, 1H), 7.83–7.81 (m, 1H), 7.63 (t, J = 8.4 Hz, 1H), 7.53–7.45 (m, 3H), 7.11 (d, J = 8.0 Hz, 1H), 6.88 (d, J = 8.4 Hz, 1H), 6.68 (d, J = 8.0 Hz, 1H), 6.28 (d, J = 8.8 Hz, 1H), 4.01 (d, J = 10.8 Hz, 1H), 3.97 (s, 3H), 3.92 (d, J = 11.2 Hz, 1H), 3.79 (s, 3H), 3.70 (dq, J = 47.2, 8.0 Hz, 2H), 0.69 (s, 3H), 0.65 (s, 3H); ^{19}**F NMR** ($CDCl_3$, 376 MHz) δ = –226.8 (t, J = 47.5 Hz, 1F); ^{13}**C NMR** ($CDCl_3$, 100 MHz) δ = 168.3, 156.1, 155.0, 139.3, 133.4, 133.2, 130.9, 128.4, 127.3, 127.1, 126.8, 126.5, 125.6, 125.3, 125.24, 125.16, 125.0, 123.7, 123.5, 122.5, 122.3, 103.6, 103.4, 87.8 (d, J = 172.1 Hz), 69.0 (d, J = 4.5 Hz), 55.4, 55.3, 35.5 (d, J = 16.4 Hz), 20.4 (d, J = 5.9 Hz), 20.3 (d, J = 5.4 Hz); **MS** (**EI**, *m/z*, %): 472 (M^+, 100.00), 339 (37.31), 473 (31.26); **HRMS** (**EI**): Calcd. For $C_{30}H_{29}O_4F$: 472.2050; Found: 472.2043.

6. Selective Deoxyfluorination of Diols 6q-6t .

6.1 Using CpFluor 1c.

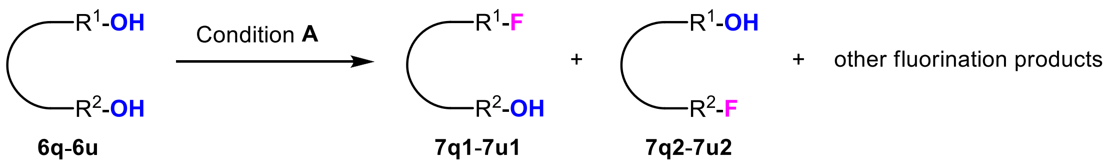

Condition A: CpFluor **1c** (1.1 equiv), 1,2-dichloroethane (0.13 M), 80 °C, 8 h.

Method:

To a dried polytetrafluoroethene (PTFE) sealed tube, alcohols **6q-6u** (0.6 mmol, 1.0 equiv.), CpFluor **1c** (190.3 mg, 0.66 mmol, 1.1 equiv.) and solvent 1,2-dichloroethane (4.5 mL) were sequentially added. The sealed tube was immersed in an oil bath at 80 °C, and the mixture was stirred for 8 hours at 80 °C. After cooling to room temperature, the mixture was analyzed by ^{19}F NMR spectroscopy with $PhCF_3$ as an internal standard (see Supplementary Figures 201-205); then the mixture was concentrated under reduced pressure and the residue was purified by flash chromatography on silica gel to give the corresponding alkyl fluoride.

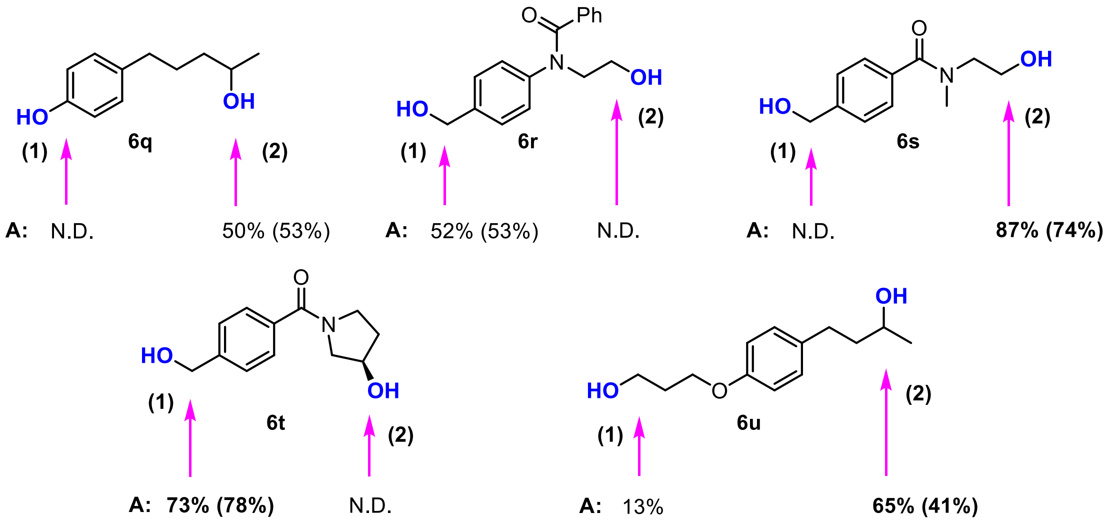

4-(4-fluoropentyl)phenol (7q2)

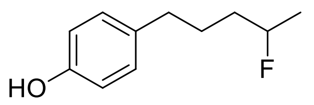

Performed with **6q** on 0.6 mmol scale, eluted with hexane/ethyl acetate = 10/1 to give 4-(4-fluoropentyl)phenol (57.8 mg, 53% yield). Light yellow liquid. **IR (film):** 3538, 3384, 3021, 2979, 2939, 2862, 1613, 1598, 1515, 1446, 1386, 1340, 1225, 1172, 1133, 1111, 925, 828, 558 cm^{-1}; ^{1}**H NMR** ($CDCl_3$, 400 MHz) δ = 7.05 (d, *J* = 8.0 Hz, 2H), 6.76 (d, *J* = 8.4 Hz, 2H), 4.80 (s, 1H), 4.77–4.57 (dm, *J* = 49.2 Hz, 1H), 2.58 (t, *J* = 7.4 Hz, 2H), 1.81–1.48 (m, 4H), 1.31 (dd, *J* = 24.4, 6.0 Hz, 3H); ^{19}**F NMR** ($CDCl_3$, 376 MHz) δ = –172.3 (m, 1F); ^{13}**C NMR** ($CDCl_3$, 100 MHz) δ = 153.5, 134.3, 129.4, 115.1, 91.0 (d, *J* = 163.4 Hz), 36.3 (d, *J* = 20.1 Hz), 34.7, 27.0 (d, *J* = 5.0 Hz), 21.0 (d, *J* = 22.7 Hz); **MS** (**EI**, *m/z*, %): 182 (M^+, 19.47), 107 (100.00); **HRMS** (**EI**): Calcd. For $C_{11}H_{15}OF$: 182.1107; Found: 182.1112.

***N*-(4-(fluoromethyl)phenyl)-*N*-(2-hydroxyethyl)benzamide (7r1)**

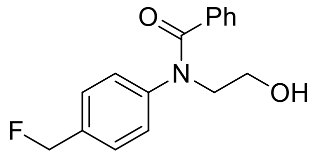

Performed with **6r** on 0.6 mmol scale, eluted with hexane/ethyl acetate = 3/1 to give *N*-(4-(fluoromethyl)phenyl)-*N*-(2-hydroxyethyl)benzamide (87.4 mg, 53% yield) *[Note: The column was eluted previously with hexane/Et_3N (50/1, v/v)]*. White solid. m.p. 88–89 °C. **IR (KBr):** 3446, 3061, 3033, 1634, 1539, 1515, 1415, 1319, 1252, 1178, 1064, 1053, 965, 929, 835, 809, 693, 671, 594 cm^{-1}; ^{1}**H NMR** ($CDCl_3$, 400 MHz) δ = 7.79–7.77 (m, 2H), 7.49 (tt, *J* = 7.4, 2.4 Hz, 1H), 7.41 (t, *J* = 7.4 Hz, 2H), 7.31 (dd, *J* = 8.6, 2.2 Hz, 2H), 6.92 (d, *J* = 8.4 Hz, 2H), 6.79 (brs, 1H), 5.29 (d, *J* = 48.4 Hz, 2H), 4.14 (t, *J* = 5.2 Hz, 2H), 3.86 (q, *J* = 5.2 Hz, 2H); ^{19}**F NMR** ($CDCl_3$, 376 MHz) δ = –199.7 (t, *J* = 48.5 Hz, 1F); ^{13}**C NMR** ($CDCl_3$, 100 MHz) δ = 167.7, 159.0 (d, *J* = 3.9 Hz), 134.2, 131.5, 129.8 (d, *J* = 5.3 Hz), 128.8 (d, *J* = 18.0 Hz), 128.5, 126.9, 114.5 (d, *J* = 2.0 Hz), 84.3 (d, *J* = 164.1 Hz), 66.8, 39.4; **MS** (**EI**, *m/z*, %): 273 (M^+, 0.78), 148 (100.00); **HRMS** (**EI**): Calcd. For $C_{16}H_{16}NO_2F$: 273.1165; Found: 273.1171.

N-(2-fluoroethyl)-4-(hydroxymethyl)-N-methylbenzamide (7s2)

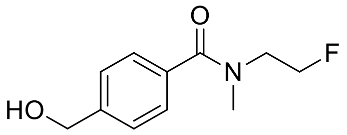

Performed with **6s** on 0.6 mmol scale, eluted with CH_2Cl_2/CH_3OH = 30/1 to give N-(2-fluoroethyl)-4-(hydroxymethyl)-N-methylbenzamide (94.1 mg, 74% yield). Light yellow liquid. **IR (film):** 3393, 2932, 1616, 1569, 1517, 1487, 1456, 1405, 1303, 1079, 1040, 1018, 851, 838, 758, 621, 571, 510 cm^{-1}; ^{1}**H NMR** ($CDCl_3$, 400 MHz) [mixture of 2 rotamers] δ = 7.31–7.27 (m, 4H), 4.69 [4.44] (d, J = 47.6 Hz, 2H), 4.58 (s, 2H), 4.12 (brs, 1H), 3.80 [3.54] (d, J = 27.2 Hz, 2H), 3.05 [3.11] (s, 3H); ^{19}**F NMR** ($CDCl_3$, 376 MHz) [mixture of 2 rotamers] δ = –222.5 [–223.3 (m, 1F)] (tt, J = 47.6, 27.6 Hz, 1F); ^{13}**C NMR** ($CDCl_3$, 100 MHz) [mixture of 2 rotamers] δ = 171.8 [172.5], 143.3 [143.1], 134.3, 126.7, 126.3, 82.6 [80.8] (d, J = 167.6 Hz), 63.81 [63.82], 48.3 [51.1] (d, J = 20.2 Hz), 39.2 [33.1]; **MS** (**EI**, *m/z*, %): 211 (M^+, 11.49), 135 (100.00); **HRMS** (**EI**): Calcd. For $C_{11}H_{14}NO_2F$: 211.1009; Found: 211.1012.

(*R*)-(4-(fluoromethyl)phenyl)(3-hydroxypyrrolidin-1-yl)methanone (7t1)

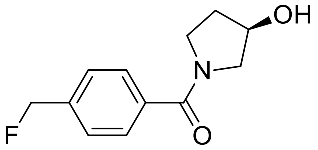

Performed with **6t** on 0.6 mmol scale, eluted with CH_2Cl_2/CH_3OH = 20/1 to give (*R*)-(4-(fluoromethyl)phenyl)(3-hydroxypyrrolidin-1-yl)methanone (104.5 mg, 78% yield). White solid. m.p. 135–137 ^{o}C. **IR (KBr):** 3335, 2983, 2941, 2923, 2882, 1609, 1571, 1518, 1451, 1317, 1295, 1275, 1218, 1173, 1103, 997, 971, 870, 854, 826, 756, 704, 646, 633, 584 cm^{-1}; ^{1}**H NMR** ($CDCl_3$, 400 MHz) [mixture of 2 rotamers] δ = 7.53 [7.50] (d, J = 7.6 Hz, 2H), 7.39–7.37 (m, 2H), 5.39 (d, J = 47.6 Hz, 2H), 4.51 [4.38] (brs, 1H), 3.82–3.50 [3.52 (dd, J = 11.2, 4.4 Hz)] (m, 3H), 3.45–3.36 (m, 1H), 3.25 [3.18] (d, J = 2.0 Hz, 1H), 2.08–1.98 (m, 1H), 1.95–1.92 (m, 1H); ^{19}**F NMR** ($CDCl_3$, 376 MHz) [mixture of 2 rotamers] δ = –210.0 [–209.8] (t, J = 47.4 Hz, 1F); ^{13}**C NMR** ($CDCl_3$, 100 MHz) [mixture of 2 rotamers] δ = 169.5 [169.6], 138.1 [138.0]

(d, J = 17.9 Hz), 136.8 [137.0], 127.4, 127.0 (d, J = 4.8 Hz), 83.9 (d, J = 166.4 Hz), 69.4 [70.7], 54.8 [57.2], 47.3 [44.2], 34.6 [32.8]; **MS** (**EI**, *m/z*, %): 223 (M^+, 27.03), 137 (100.00); **HRMS** (**EI**): Calcd. For $C_{12}H_{14}NO_2F$: 223.1009; Found: 223.1014.

3-(4-(3-fluorobutyl)phenoxy)propan-1-ol (7u2)

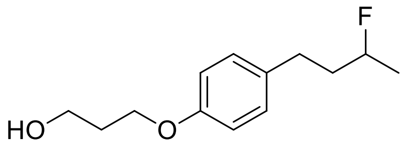

Performed with **6u** on 0.6 mmol scale, eluted with hexane/ethyl acetate = 8/1 to give 3-(4-(3-fluorobutyl)phenoxy)propan-1-ol (55.6 mg, 41% yield). Light yellow liquid. **IR (film):** 3385, 2976, 2935, 2877, 1612, 1512, 1472, 1386, 1298, 1244, 1176, 1132, 1062, 992, 950, 922, 881, 828 cm^{-1}; **^{1}H NMR** ($CDCl_3$, 400 MHz) δ = 7.11 (d, J = 8.8 Hz, 2H), 6.84 (d, J = 8.4 Hz, 2H), 4.75–4.55 (dm, J = 48.8 Hz, 1H), 4.10 (t, J = 6.0 Hz, 2H), 3.85 (t, J = 5.8 Hz, 2H), 2.77–2.70 (m, 1H), 2.67–2.60 (m, 1H), 2.19 (brs, 1H), 2.03 (p, J = 6.0 Hz, 2H), 1.98–1.89 (m, 1H), 1.86–1.70 (m, 1H), 1.34 (dd, J = 24.0, 6.0 Hz, 3H); **^{19}F NMR** ($CDCl_3$, 376 MHz) δ = –174.1 (m, 1F); **^{13}C NMR** ($CDCl_3$, 100 MHz) δ = 157.0, 133.7, 129.3, 114.4, 90.0 (d, J = 163.5 Hz), 65.7, 60.3, 38.8 (d, J = 21.4 Hz), 31.9, 30.3 (d, J = 5.6 Hz), 20.9 (d, J = 22.5 Hz); **MS** (**EI**, *m/z*, %): 226 (M^+, 22.14), 107 (100.00); **HRMS** (**EI**): Calcd. For $C_{13}H_{19}O_2F$: 226.1369; Found: 226.1371.

6.2 Using DAST.

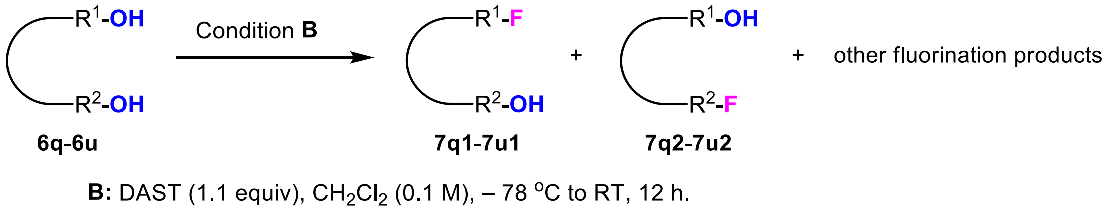

Method:

Experiments were performed according to reported procedures.[28] To a dried

polytetrafluoroethene (PTFE) sealed tube containing alcohols **6q-6u** (0.2 mmol, 1.0 equiv.) and solvent CH_2Cl_2 (0.1 M) was added DAST (for **6q-6s**, 1.5 equiv; for **6t-6u**, 1.1 equiv) at –78 °C. The tube was sealed and warmed to RT. The mixture was stirred for 12 hours at RT; then the mixture was analyzed by ^{19}F NMR spectroscopy with $PhCF_3$ as an internal standard (see Supplementary Figures 206-210).

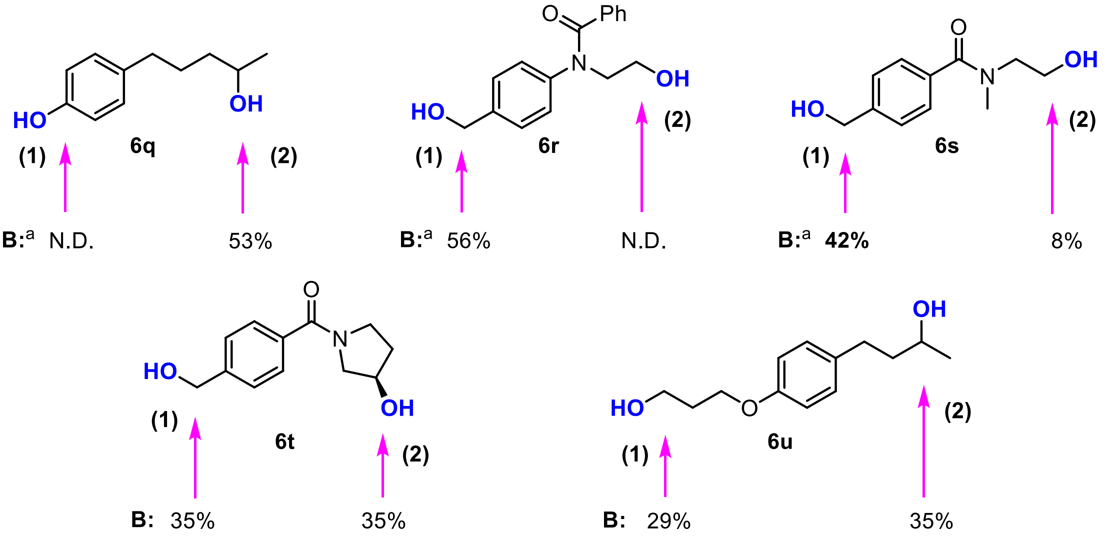

6.3 Using PhenoFluor.

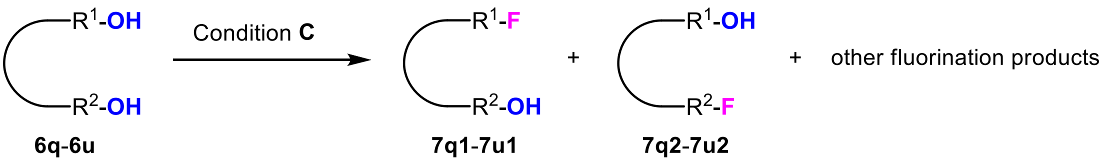

C: PhenoFluor (1.2 equiv), KF (2.0 equiv), DIPEA (2.0 equiv), toluene (0.04 M), 80 °C, 12 h.

Method:

Experiments were performed according to reported procedures.[20] To a dried polytetrafluoroethene (PTFE) sealed tube were added alcohols **6q-6u** (0.2 mmol, 1.0 equiv.), KF (2.0 equiv), and toluene (0.04 M) sequentially. *N*,*N*-Diisopropylethylamine (DIPEA, 2.0 equiv) was added, followed by a toluene solution of PhenoFluor (for **6q** and **6r**, 1.5 equiv; for **6s**, 1.0 equiv; for **6t** and **6u**, 1.5 equiv). The tube was sealed and heated to 80 °C (for **6s**, RT). The mixture was stirred for 12 hours (for **6s**, 48 h); then the mixture was analyzed by ^{19}F NMR

spectroscopy with $PhCF_3$ as an internal standard (see Supplementary Figures 211-215).

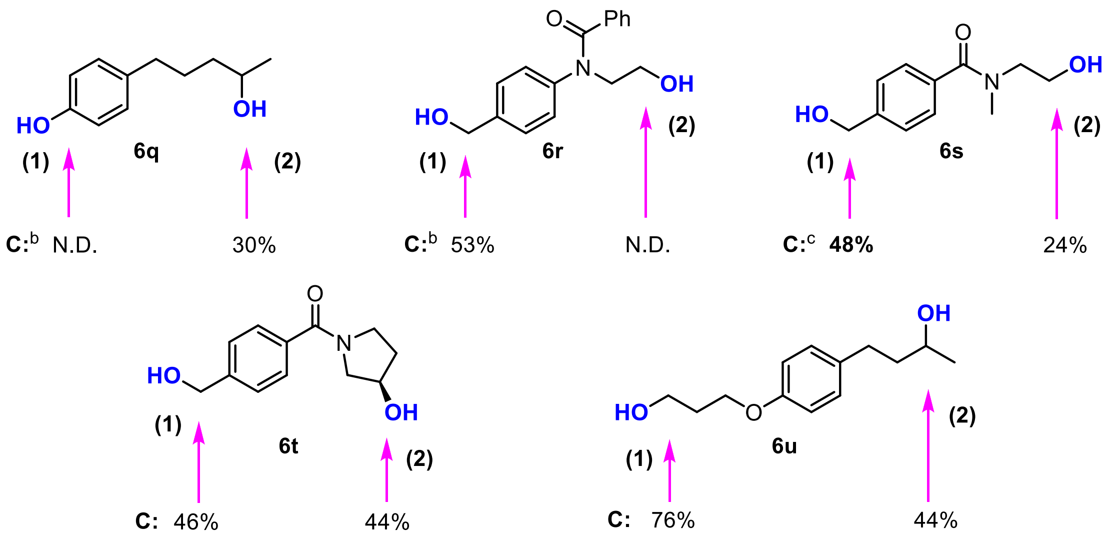

6.4 Using PyFluor.

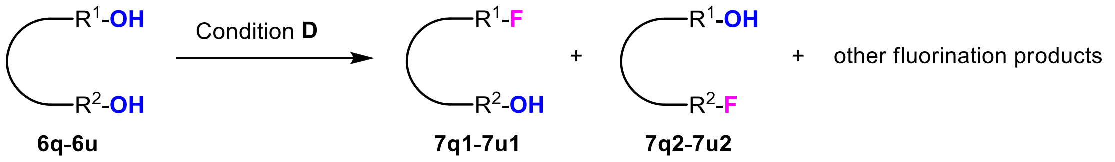

D: PyFluor (1.1 equiv), MTBD (2.0 equiv), toluene (0.5 M), RT, 48 h.

Method:

Experiments were performed according to reported procedures.[26] A PTFE sealed tube was charged sequentially with alcohols **6q-6u** (0.5 mmol, 1.0 equiv.), toluene (1.0 mL, 0.5 M), PyFluor (88.6 mg, 0.55 mmol, 1.1 equiv), and 7-methyl-1,5,7-triazabicyclo[4.4.0]dec-5-ene (MTBD, 153.2 mg, 1.0 mmol, 2.0 equiv). The mixture was stirred at room temperature for 48 hours; then the mixture was analyzed by ^{19}F NMR spectroscopy with $PhCF_3$ as an internal standard (see Supplementary Figures 215-220); then the mixture was concentrated under reduced pressure and the residue was purified by flash chromatography on silica gel to give the corresponding alkyl fluoride

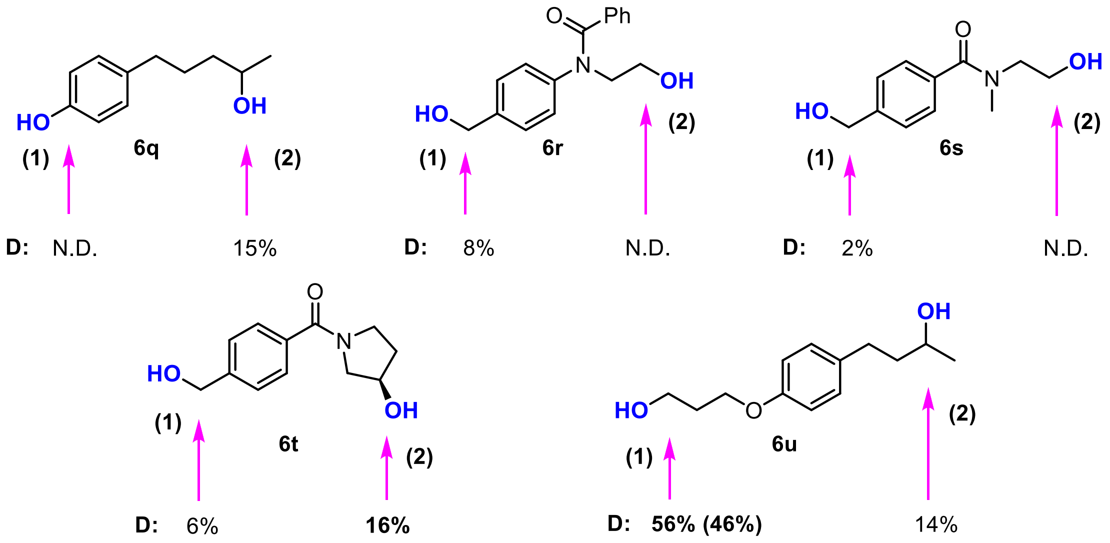

4-[4-(3-fluoropropoxy)phenyl]butan-2-ol (**7u1**)

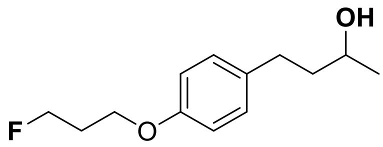

Performed with **6u** on 0.5 mmol scale, eluted with hexane/ethyl acetate = 5/1 (v/v) to give 4-[4-(3-fluoropropoxy)phenyl]butan-2-ol (**7u1**) (51.9 mg, 46%). Light yellow liquid (51.9 mg, 46%). **^{1}H NMR** ($CDCl_3$, 400 MHz) δ = 7.11 (d, J = 8.8 Hz, 2H), 6.83 (d, J = 8.8 Hz, 2H), 4.64 (dt, J = 47.2, 5.6 Hz, 2H), 4.08 (t, J = 6.2 Hz, 2H), 3.86–3.78 (m, 1H), 2.73–2.58 (m, 2H), 2.16 (dp, J = 25.6, 6.0 Hz, 2H), 1.77–1.71 (m, 2H), 1.37 (brs, 1H), 1.22 (d, J = 6.4 Hz, 3H); **^{19}F NMR** ($CDCl_3$, 376 MHz) δ = –222.2 (tt, J = 47.1, 26.0 Hz, 1F); **^{13}C NMR** ($CDCl_3$, 100 MHz) δ = 156.9, 134.3, 129.2, 114.4, 80.7 (d, J = 163.2 Hz), 67.4, 63.4 (d, J = 4.8 Hz), 41.0, 31.1, 30.4 (d, J = 19.4 Hz), 23.5. **MS** (**EI**, *m/z*, %): 226 (M^+, 46.42), 107 (100.00), 179 (84.37); **HRMS** (**EI**): Calcd. For $C_{13}H_{19}O_2F$: 226.1369; Found: 226.1371.

7. Experimental Investigation of Reaction Mechanism

7.1. NMR Study of the Reaction between Monoalcohol 2a and CpFluor 1a

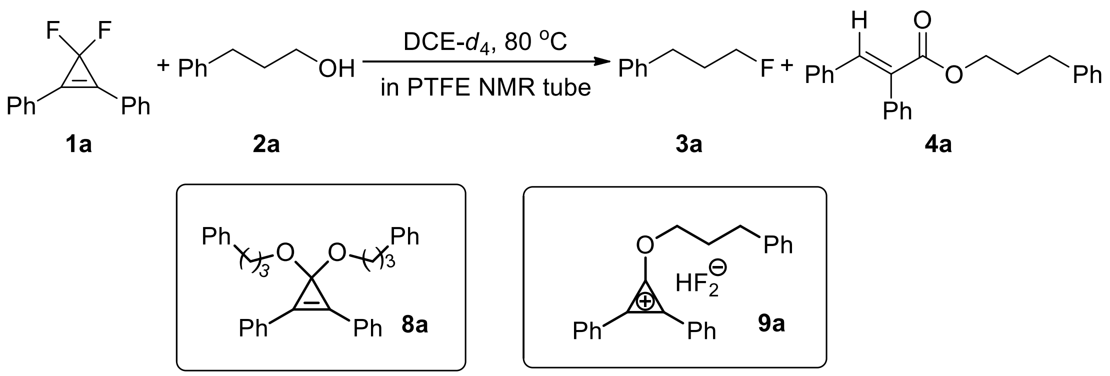

Method:

To a dry PTFE NMR tube (purchased from J&K, product No. PTFE-5MM-KIT), alcohol **2a** (6.8 mg, 0.05 mmol, 1.0 equiv.), CpFluor **1a** (11.4 mg, 0.05 mmol, 1.0 equiv.) and deuterated 1,2-dichloroethane (DCE-d_4) (~ 0.4 mL) were sequentially added, then $PhCF_3$ (9.8 mg) was added as an internal standard. The NMR tube was sealed and immersed in an oil bath at 80 ^{o}C, and the progress of the reaction was monitored by ^{19}F and ^{1}H NMR spectroscopy (at 25 ^{o}C) at the time shown in Supplementary Table 2. The conversion of CpFluor **1a** and the yields of HF and alkyl fluoride **3a** were determined by ^{19}F NMR spectroscopy (using the integration of the CF_3 of added $PhCF_3$ as the standard). The yields of ester **4a**, acetal **8a** (based on **2a**), and alkoxycyclopropenium cation **9a** were determined by ^{1}H NMR spectroscopy (using the total integration of the residual non-deuterated and partially deuterated solvent as the standard). Representational ^{1}H NMR spectra are shown in Supplementary Figure 221.

7.2. Synthesis of Acetal 8a and Its Reaction with Hydrogen Fluoride.

The involvement of acetal **8a** as one of the key intermediates was further confirmed by the ring-opening fluorination of separately prepared **8a** with pyridine hydrogen fluoride complex, which also gave a mixture of alkyl fluoride **3a** and ester **6a**.

Synthesis of Acetal 8a:

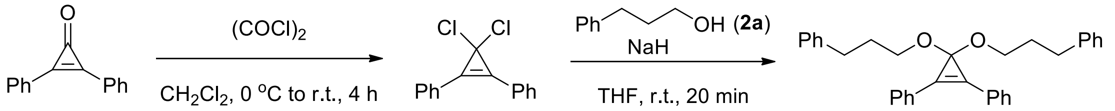

To a 25-mL oven-dried round bottomed flask, 2,3-diphenylcycloprop-2-enone (**5a**, 618.7 mg, 3.0 mmol, 1.0 equiv.) and anhydrous CH_2Cl_2 (10.0 mL) were successively added. The reaction mixture was cooled with ice-water, then oxalyl dichloride (309 μL, 456.9 mg, 3.6 mmol, 1.2 equiv.) was added, after the reaction mixture was stirred for 30 min, remove the cold bath and continue stirring for additional 4 h. The residual oxalyl dichloride and other low boiling point substances were evaporated. After recrystallized from anhydrous hexane, (3,3-dichlorocycloprop-1-ene-1,2-diyl) dibenzene (**12**) was got as white solid (660 mg, 84%).

To an oven-dried reaction tube, 3-phenylpropan-1-ol (**2a**, 163.4 mg, 1.2 mmol, 2.4 equiv.) and 5.0 mL anhydrous THF were successively added, then NaH (95%, 33.3 mg, 1.32 mmol, 2.64 equiv.) was carefully added with stirring, after the reaction mixture was stirred for 20 min, (3,3-dichlorocycloprop-1-ene-1,2-diyl)dibenzene (130.6 mg, 0.5 mmol, 1.0 equiv.) was added and continue stirring for additional 4 h. the precipitate was filtered through a pad of basic Al_2O_3, eluting with Et_3N and Et_2O. The filtrate was concentrated under reduced pressure to obtain a crude oil that was purified by column chromatography on silica gel (petroleum ether/Et_3N 100:1 v/v) to obtain the title compound **8a** as a light yellow liquid (65.3 mg, 28%). *[Note: This product was sensitive to acid, so the column must be eluted previously with petroleum ether/Et_3N (20/1 v/v)].* **IR (film):** 3082, 3061, 3025, 2928, 2863, 1773, 1708, 1602, 1496, 1454, 1447, 1234, 1062, 760, 745, 690 cm^{-1}; **^{1}H NMR** ($CDCl_3$, 400 MHz) δ = 7.75 (d, *J* = 7.2 Hz, 4H), 7.47 (t, *J* = 7.6 Hz, 4H), 7.41 (t, *J* = 7.4 Hz, 2H), 7.20 (t, *J* = 7.2 Hz, 4H), 7.14 (t, *J* = 6.2 Hz, 6H), 3.72 (t, *J* = 6.6 Hz, 4H) 2.69 (t, *J* = 7.6 Hz, 4H), 1.96–1.89 (m, 4H); **^{13}C NMR** ($CDCl_3$, 100 MHz) δ = 142.0, 129.7, 129.4, 128.9, 128.5, 128.2, 127.9, 126.4, 125.6, 86.9, 66.5, 32.4, 31.5; **MS** (**EI**, *m/z*, %): 460

(M^+, 0.85), 91 (100.00); **HRMS** (**EI**): Calcd. For $C_{33}H_{32}O_2$: 460.2402; Found: 460.2396.

Reaction of Acetal 8a with Hydrogen Fluoride:

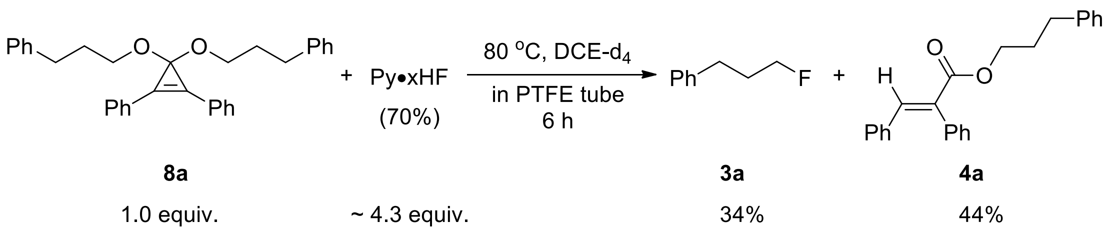

To a dry PTFE NMR tube (purchased from J&K, product No. PTFE-5MM-KIT), compound **8a** (11.5 mg, 0.025 mmol, 1.0 equiv.), $PhCF_3$ (8.3 mg, internal standard) and deuterated 1,2-dichloroethane (DCE-d_4) (~ 0.3 mL) were sequentially added, then Py •xHF (70%, 3.1 mg, contain about 0.11 mmol HF, 4.3 equiv.) was added. The NMR tube was sealed and immersed in an oil bath at 80 °C, and the progress of the reaction was monitored by ^{19}F and ^1H NMR spectroscopy (at 25 °C) The yield of alkyl fluoride **3a** was determined by ^{19}F NMR spectroscopy (using the integration of CF_3 of the added $PhCF_3$ as a standard). The yield of ester **4a** was determined by ^1H NMR spectroscopy (using the integration of CH_2F of the alkyl fluoride **3a** as a standard).

7.3. NMR Study of the Reaction between Monoalcohol 2a and CpFluor 1c

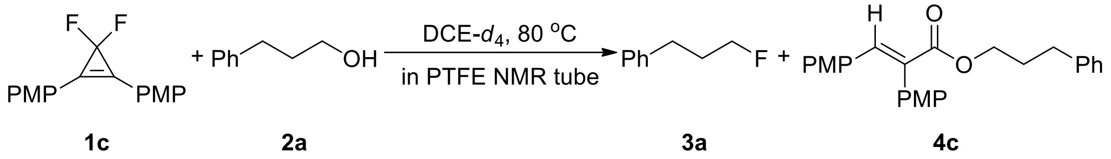

(PMP = *p*-methoxyphenyl)

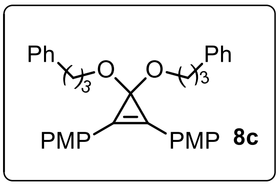

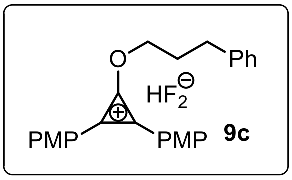

Method:

To a dry PTFE NMR tube (purchased from J&K, product No. PTFE-5MM-KIT), alcohol **2a** (5.4 mg, 0.04 mmol, 1.0 equiv.), CpFluor **1c** (11.5 mg, 0.04 mmol, 1.0 equiv.) and deuterated 1,2-dichloroethane (DCE-d_4) (~ 0.35 mL) were sequentially added, then $PhCF_3$ (10.0 mg) was added as an internal standard. The NMR tube was sealed and immersed in an oil bath at 80 ^{o}C, and the progress of the reaction was monitored by ^{19}F and ^{1}H NMR spectroscopy (at 25 ^{o}C) at the time shown in Supplementary Table 3. The conversion of CpFluor **1c** and the yields of HF and alkyl fluoride **3a** were determined by ^{19}F NMR spectroscopy (using the integration of Cf3 of the added $PhCF_3$ as a standard). The yields of ester **4c**, acetal **8c** (based on **2a**), and alkoxycyclopropenium cation **9c** were determined by ^{1}H NMR spectroscopy (using the total integration of the residual non-deuterated and partially deuterated solvent and the total integration of the aromatic hydrogens as the standard). Representational ^{1}H NMR spectra are shown in Supplementary Figure 222.

Supplementary References

[1] Aikawa, K., Hioki, Y., Shimizu, N. & Mikami, K. Catalytic asymmetric synthesis of stable oxetenes via Lewis acid-promoted [2 + 2] cycloaddition. *J. Am. Chem. Soc.* **133**, 20092−20095 (2011).

[2] Beshai, M., Dhudshia, B., Mills, R. & Thadani, A. N. Terminal alkynes from aldehydes via dehydrohalogenation of (*Z*)-1-iodo-1-alkenes with TBAF. *Tetrahedron Lett.* **49**, 6794–6796 (2008).

[3] Park, K., Bae, G., Moon, J., Choe, J., Song, K. H. & Lee, S. Synthesis of symmetrical and unsymmetrical diarylalkynes from propiolic acid using palladium-catalyzed decarboxylative coupling. *J. Org. Chem.* **75**, 6244–6251 (2010).

[4] Fürstner, A. & Kennedy, J. W. J. Total syntheses of the tylophora alkaloids cryptopleurine, (−)-antofine, (−)-tylophorine, and (−)-ficuseptine C. *Chem. Eur. J.*

12, 7398–7410 (2006).

[5] Ueda, H., Yamaguchi, M., Kameya, H., Sugimoto, K. & Tokuyama, H. Autotandem catalysis: synthesis of pyrroles by gold-catalyzed cascade reaction. *Org. Lett.* **16**, 4948–4951 (2014).

[6] Trosien, S. & Waldvogel, S. R. Synthesis of highly functionalized 9,10-phenanthrenequinones by oxidative coupling using $MoCl_5$. *Org. Lett.* **14**, 2976–2979 (2012).

[7] Ren, Y.-L., Shang, H., Wang, J., Tian, X., Zhao, S., Wang, Q. & Li, F. Nitrogen dioxide-catalyzed electrophilic iodination of arenes. *Adv. Synth. Catal.* **355**, 3437–3442 (2013).

[8] Scannell, R. T. & Stevenson, R. Natural benzofurans. synthesis of medicagol methoxybenzofuran. *J. Heterocyclic Chem.* **17**, 1727–1728 (1980).

[9] Panetta, C. A., Fang, Z. & Mattern, D. L. Iodination of methylated anisoles: unusual aryl methyl replacements and oxidations. *J. Org. Chem.* **60**, 7953–7958 (1995).

[10] Zhao, W., Huang, L., Guan, Y. & Wulff, W. D. Three-component asymmetric catalytic Ugi reaction–concinnity from diversity by substrate-mediated catalyst assembly. *Angew. Chem. Int. Ed.* **53**, 3436–3441 (2014).

[11] Wee, K.-R., Cho, Y.-J., Song, J. K. & Kang, S. O. Multiple photoluminescence from 1,2-dinaphthyl-*ortho*-carborane. *Angew. Chem. Int. Ed.* **52**, 9682–9685 (2013).

[12] Iskra, J., Stavber, S. & Zupan, M. Nonmetal-catalyzed iodination of arenes with iodide and hydrogen peroxide. *Synthesis* 1869–1873 (2004).

[13] Tietze, L. F., Vock, C. A., Krimmelbein, I. K. & Nacke, L. Synthesis of novel structurally simplified estrogen analogues with electron-donating groups in ring A. *Synthesis* 2040–2060 (2009).

[14] Wang, F., Li, L., Ni, C. & Hu, J. Deoxygenative gem-difluoroolefination of carbonyl compounds with (chlorodifluoromethyl)trimethylsilane and triphenyl-phosphine. *Beilstein J. Org. Chem.* **10**, 344–351 (2014).

[15] Wang, F., Zhang, W., Zhu, J., Li, H., Huang, K.-W. & Hu, J. Chloride ion-catalyzed generation of difluorocarbene for efficient preparation of *gem*-difluorinated cyclopropenes and cyclopropanes. *Chem. Commun.* **47**, 2411–2413 (2011).

[16] Wu, H. & Moeller, K. D. Anodic coupling reactions: a sequential cyclization route to the arteannuin ring skeleton. *Org. Lett.* **9**, 4599–4602 (2007).

[17] Nicolaou, K. C., Reingruber, R., Sarlah, D. & Bräse, S. Enantioselective intramolecular Friedel–Crafts-type α-arylation of aldehydes. *J. Am. Chem. Soc.* **131**, 2086–2087 (2009).

[18] Xing, P., Zang, W., Huang, Z.-G., Zhan, Y.-X., Zhu, C.-J. & Jiang, B. A mild method for Indium(III)-catalyzed 1,4-hydrosilylation of α,β-enone esters with triethylsilane and trifluoroacetic acid. *Synlett* **23**, 2269–2273 (2012).

[19] Fukuzawa, S., Fujinami, T., Yamauchi, S. & Sakai, S. 1,2-Regioselective reduction of α,β-unsaturated carbonyl compounds with lithium aluminium hydride in the presence of lanthanoid salts. *J. Chem. Soc., Perkin Trans. 1*, 1929–1932 (1986).

[20] Sladojevich, F., Arlow, S. I., Tang, P. & Ritter, T. Late-stage deoxyfluorination of alcohols with PhenoFluor. *J. Am. Chem. Soc.* **135**, 2470–2473 (2013).

[21] Jadhav, V. H., Kim, J. G., Jeong, H. J. & Kim, D. W. Nucleophilic hydroxylation in water media promoted by a hexa-ethylene glycol-bridged dicationic ionic liquid. *J. Org. Chem.* **80**, 7275–7280 (2015).

[22] Maeda, H., Koide, T., Matsumoto, S. & Ohmori, H. Fluorination of secondary and primary alcohols by thermal decomposition of electrochemically generated alkoxy triphenylphosphonium tetrafluoroborates. *Chem. Pharm. Bull.* **44**, 1480–1483 (1996).

[23] Aoyama, M., Fukuhara, T. & Hara, S. Selective fluorination of adamantanes by an electrochemical method. *J. Org. Chem.* **73**, 4186–4189 (2008).

[24] Knox, L. H., Velarde, E., Berger, S., Cuadriello, D. & Cross, A. D. Steroids. CCXL. The reaction of steroidal alcohols with 2-Chloro-1,1,2-trifluorotriethylamine. *J. Org. Chem.* **29**, 2187–2195 (1964).

[25] Kobayashi, Y., Kumadaki, I., Ohsawa, A., Honda, M. & Hanzawa, Y. Studies on organic fluorine compounds. XVI.: stereochemistry in fluorination of sterols with phenylfluorophosphoranes. *Chem. Pharm. Bull.* **23**, 196–200 (1975).

[26] Nielsen, M. K., Ugaz, C. R., Li, W. & Doyle, A. G. PyFluor: a low-cost, stable, and selective deoxyfluorination reagent. *J. Am. Chem. Soc.* **137**, 9571–9574 (2015).

[27] Klapars, A., Huang, X. & Buchwald S. L. A general and efficient copper catalyst

for the amidation of aryl halides. *J. Am. Chem. Soc.* **124**, 7421–7428 (2002).

[28] Middleton, W. J. New fluorinating reagents. dialkylaminosulfur fluorides. *J. Org. Chem.* **40**, 574–578 (1975)
